# Supplementary material for: Staffing levels and hospital mortality in England: a national panel study using routinely collected data
Source: BMJ Open. 2023 May 17;13(5):e066702. doi: 10.1136/bmjopen-2022-066702 (PMC10193053; doi:10.1136/bmjopen-2022-066702)

Supporting information

Tables

Table S1. NHS digital data sources

Data source	Datasets	Main variables available
NHS workforce data	Medical dataset	Professionally qualified clinical staff
	Non-medical dataset	Professionally qualified clinical staff
		Support to clinical staff
Bed occupancy data	Overnight beds dataset	Available beds
		Occupied beds
		% occupied beds
	Day only beds datasets	Available beds
		Occupied beds
		% occupied beds
ERIC data		Trust profile (e.g. type)
		Strategies and policies
		Finance
		Safety
		Fire and safety
		Business transport
		Medical records
		Key worker accommodation
		Facilities management services
		Areas
		Function and space
		Quality of buildings
		Combined heat and power energy
		Energy
		Water services
		Waste
SHMI data		SHMI value
		SHMI banding
		Number of spells*
		Observed deaths
		Expected deaths

NHS: National Health Service, ERIC: Estates Returns Information Collection, SHMI: Summary Hospital level Mortality Indicator, * spells: total continuous stay of a patient using a hospital bed at an NHS organisation under the care of one or more consultants, or nursing episode or midwife episode.

Table S2. Comparison of goodness-of-fit for model selection

Model	Correlations for trusts	Model degrees of freedom	AIC	BIC	Likelihood-ratio
**OLS**	No	17	8183.5	8255.8	-4074.7
**Poisson**	No	16	10567.6	10635.7	-5267.8
**Negative binomial**	No	17	6692.6	6764.9	-3329.3
**GLS**	Yes	26	7517.0	7627.6	-3732.5
**GLS exchangeable**	Yes	18	7867.5	7944.0	-3915.7
**GLS Toeplitz (2)**	Yes	19	7823.9	7904.7	-3892.9
**GLS AR**	Yes	18	7822.7	7899.2	-3893.3
**Poisson RE**	Yes	14	1997.8	2057.3	-984.9
**Negative binomial RE**	Yes	14	250.3	309.9	-111.2
**Poisson FE**	Yes (dummy)	146	6477.4	7098.2	-3092.7
**Negative binomial FE**	Yes (dummy)	147	6180.5	6805.6	-2943.3
**Negative binomial WBRE**	Yes	22	6418.3	6511.8	-3187.1

AIC: Akaike information criterion, BIC: Bayesian information criterion, OLS: ordinary least squares, GLS: generalised least squares, AR: autoregressive, RE: random effects, FE: fixed effects, WBRE: within-between random effects, (2) tridiagonal 2-Toeplitz matrix

Table S3. Multicollinearity test in the negative binomial random effects model

Variable	GVIF
Beds per medical	3.1
Beds per surgical	3.6
Beds per other medical	6.1
Beds per nurse	3.7
Beds per nurse support	1.7
Beds per AHP	2.0
Beds per AHP support	1.4

Beds per ST&T	2.6
Teaching status	1.8
Trust size	1.1*

GVIF: generalised variance inflation factor; AHP: allied health professionals; ST&T: scientific, therapeutic, and technical; *reported on GVIF^(1/(2*degrees of freedom)) instead of GVIF as variable has 2 degrees of freedom

Figure S1. Spearman correlation between staffing level variables included in the study.

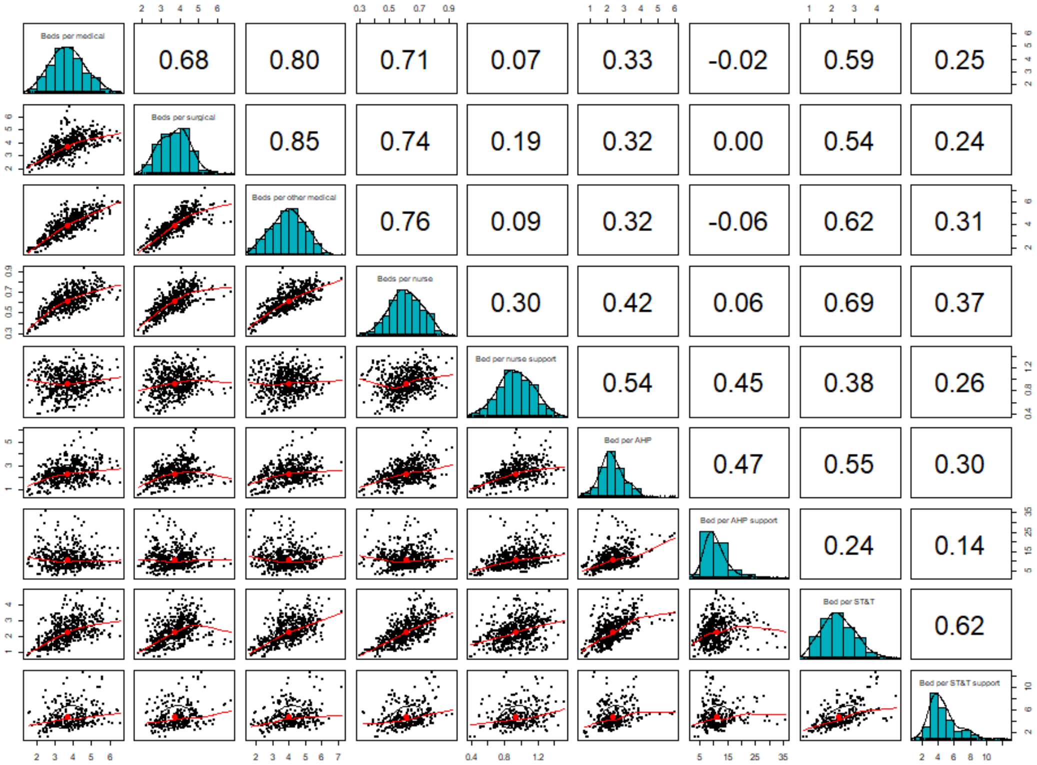

Supplement: Supplementary data [file bmjopen-2022-066702supp001.pdf]
