# Supplementary material for: Staffing levels and hospital mortality in England: a national panel study using routinely collected data
Source: BMJ Open. 2023 May 17;13(5):e066702. doi: 10.1136/bmjopen-2022-066702 (PMC10193053; doi:10.1136/bmjopen-2022-066702)

BMJ Open

BMJ Open is committed to open peer review. As part of this commitment we make the peer review history of every article we publish publicly available.

When an article is published we post the peer reviewers' comments and the authors' responses online. We also post the versions of the paper that were used during peer review. These are the versions that the peer review comments apply to.

The versions of the paper that follow are the versions that were submitted during the peer review process. They are not the versions of record or the final published versions. They should not be cited or distributed as the published version of this manuscript.

BMJ Open is an open access journal and the full, final, typeset and author-corrected version of record of the manuscript is available on our site with no access controls, subscription charges or pay-per-view fees (http://bmjopen.bmj.com).

If you have any questions on BMJ Open's open peer review process please email info.bmjopen@bmj.com

BMJ Open

Staffing levels and hospital mortality in England: a national panel study using routinely collected data

Journal:	*BMJ Open*
Manuscript ID	bmjopen-2022-066702
Article Type:	Original research
Date Submitted by the Author:	18-Jul-2022
Complete List of Authors:	Rubbo, Bruna; University of Southampton, School of Health Sciences<br>Saville, Christina; University of Southampton, School of Health Sciences; National Institute for Health Research Applied Research Collaboration (Wessex), University of Southampton<br>Dall'Ora, Chiara; University of Southampton, School of Health Sciences; National Institute for Health Research Applied Research Collaboration (Wessex), University of Southampton<br>Turner, Lesley; University of Southampton, School of Health Sciences<br>Jones, Jeremy; University of Southampton, School of Health Sciences<br>Ball, Jane; University of Southampton, School of Health Sciences<br>Culliford, David; University of Southampton, Faculty of Medicine; National Institute for Health Research Applied Research Collaboration (Wessex), University of Southampton<br>Griffiths, Peter; University of Southampton, School of Health Sciences; National Institute for Health Research Applied Research Collaboration (Wessex), University of Southampton
Keywords:	HEALTH SERVICES ADMINISTRATION & MANAGEMENT, Organisation of health services < HEALTH SERVICES ADMINISTRATION & MANAGEMENT, Quality in health care < HEALTH SERVICES ADMINISTRATION & MANAGEMENT, Health policy < HEALTH SERVICES ADMINISTRATION & MANAGEMENT

SCHOLARONE™
Manuscripts

I, the Submitting Author has the right to grant and does grant on behalf of all authors of the Work (as defined in the below author licence), an exclusive licence and/or a non-exclusive licence for contributions from authors who are: i) UK Crown employees; ii) where BMJ has agreed a CC-BY licence shall apply, and/or iii) in accordance with the terms applicable for US Federal Government officers or employees acting as part of their official duties; on a worldwide, perpetual, irrevocable, royalty-free basis to BMJ Publishing Group Ltd ("BMJ") its licensees and where the relevant Journal is co-owned by BMJ to the co-owners of the Journal, to publish the Work in this journal and any other BMJ products and to exploit all rights, as set out in our licence.

The Submitting Author accepts and understands that any supply made under these terms is made by BMJ to the Submitting Author unless you are acting as an employee on behalf of your employer or a postgraduate student of an affiliated institution which is paying any applicable article publishing charge ("APC") for Open Access articles. Where the Submitting Author wishes to make the Work available on an Open Access basis (and intends to pay the relevant APC), the terms of reuse of such Open Access shall be governed by a Creative Commons licence – details of these licences and which Creative Commons licence will apply to this Work are set out in our licence referred to above.

Other than as permitted in any relevant BMJ Author's Self Archiving Policies, I confirm this Work has not been accepted for publication elsewhere, is not being considered for publication elsewhere and does not duplicate material already published. I confirm all authors consent to publication of this Work and authorise the granting of this licence.

For peer review only

Staffing levels and hospital mortality in England: a national panel study using routinely collected data

Bruna Rubbo[1], Christina Saville[1,2], Chiara Dall'Ora[1,2], Lesley Turner[1], Jeremy Jones[1], Jane Ball[1], David Culliford[2], Peter Griffiths[1,2]*

[1]University of Southampton, School of Health Sciences, Southampton, UK

[2]National Institute for Health Research Applied Research Collaboration, Wessex

*Corresponding author

peter.griffiths@soton.ac.uk (PG)

Short title: Staffing levels and hospital mortality

For peer review only - http://bmjopen.bmj.com/site/about/guidelines.xhtml

Abstract

Objectives

Examine the association between multiple clinical staff levels and case-mix adjusted patient mortality in English hospitals. Most studies investigating the association between hospital staffing levels and mortality have focused on single professional groups, in particular nursing. However, single staff group studies might overestimate effects or neglect important contributions to patient safety from other staff groups.

Design

Retrospective observational study of routinely available data.

Setting and participants

138 National Health Service hospital trusts that provided general acute adult services in England between 2015 and 2019.

Outcome measure

Standardised mortality rates were derived from the Summary Hospital level Mortality Indicator dataset.

Results

Using a multilevel negative binomial random effects model, hospitals with higher levels of medical and allied healthcare professional (AHP) staff had significantly lower mortality rates (1.04, 95%CI 1.02 to 1.06, and 1.04, 95%CI 1.02 to 1.06, respectively), while those with higher support staff had higher mortality rates (0.85, 95%CI 0.79 to 0.91 for nurse support, and 1.00, 95%CI 0.99 to 1.00 for AHP support), after adjusting for multiple staff groups and hospital characteristics. Estimates of staffing levels on mortality were higher in magnitude between- than within-hospitals, which were not statistically significant in a within-between random effects model.

Conclusions

Considering multiple staff groups simultaneously when examining the association between hospital mortality and clinical staffing levels is crucial. As the main variation was seen between- as opposed to within-hospitals, structural recruitment and retention difficulties coupled with financial constraints could contribute to the effect of staffing levels on hospital mortality.

Article Summary

Strengths and limitations of the study

- We collected data from 138 hospitals over 4 years
- We included numerous clinical staffing groups simultaneously
- We did not have access to data at the ward level, so our associations were at the hospital level only.
- We used occupied beds-per-staff, but this might not be a good measure of staffing levels, as it does not take into account temporary staff or staff absence.

Keywords

Scheduling and staffing; Hospital Mortality; Workforce; Patient Safety; Delivery of Health Care

For peer review only - http://bmjopen.bmj.com/site/about/guidelines.xhtml

Introduction

Cost constraints and workforce shortages put pressure on health systems to find efficient staffing models to meet rising demand for care. In some countries, such as England, this has led to an increased reliance on support workforce to provide nursing care. Whilst reducing the skill mix may create short-term savings, multiple observational studies provide evidence of a risk of higher in-hospital mortality rates when registered nurse staffing levels are lowered and skill mix is diluted (1-7).

Studies focussing on the impact of clinical staff other than nurses on hospital mortality rates are relatively scarce. While some studies on medical staffing levels exist, indicating worse outcomes with lower medical staffing levels (3), most focus on differing models of rostering or deployment e.g. intensive care unit staffing models (8), physician caseload levels (9-11), or the 'weekend effect', whereby worse outcomes for patients admitted over weekends have been associated with having fewer experienced physicians rostered over weekend (12). To our knowledge, no recent study has examined the potential effect of allied healthcare professionals (AHP) staffing levels on clinical outcomes and only a few focused on in-hospital pharmacists (13, 14).

A small number of studies assessed the simultaneous impact of multiple staff groups on hospital mortality, even though in-hospital patient care is delivered by multidisciplinary staff and no single staff group is solely responsible for patient outcomes. Of the studies that did consider multiple staff groups, some found partial attenuation or complete absence of the effect of single staff levels on mortality when adjusted for other professionally qualified staff, indicating that uni-professional staffing estimates are potentially biased by confounding (3, 15).

In the face of enduring staffing shortages in some professional groups it is important to understand the likely consequences on patient care and to identify priorities. A focus on single professional groups risks unintended consequences through neglect of other important contributions to patient safety and might lead to biased estimates. We therefore aimed to examine the associations between staffing levels of multiple staff groups, and case-mix adjusted patient mortality in hospitals.

Methods

Study design

This was a retrospective observational study using routinely available data on clinical healthcare staffing and hospital mortality.

Study setting

We included all 138 National Health Service (NHS) hospital trusts providing general acute adult inpatient services in England between April 2015 and March 2019. Hospital trusts are defined as organisational units within the NHS that service a defined geographical area or that provide a specialised function. One trust can therefore encompass several hospitals.

For peer review only - http://bmjopen.bmj.com/site/about/guidelines.xhtml

Data sources and linkage

We linked four data sources that provide trust-level datasets: a) NHS workforce, which contains detailed information on trust staffing; b) bed availability and occupancy, which contains data on trust-level available and occupied beds; c) Estates Returns Information Collection (ERIC), which contains data on trust organisation and structure; and d) Summary Hospital-level Mortality Indicator (SHMI), which contains data on observed and expected deaths (Table S1). These datasets are openly available and accessible on the NHS Digital platform, along with the data dictionary (16, 17). We linked the datasets using the unique hospital trust ID (*i.e.* “Org code”).

Study outcome

The main study outcome was all-cause mortality, with standardised mortality rates derived from observed and expected deaths in the SHMI dataset (18). This is calculated and reported for all trusts providing acute adult services in NHS England (19). Specialist trusts that do not provide general acute care do not report SHMI and, therefore, were not included in the study.

The SHMI includes all in-hospital deaths and those that occurred within 30-days of discharge from patients admitted to non-specialist acute trusts. SHMI data are derived from the Hospital Episode Statistics (HES) at the level of provider spells (*i.e.* total continuous stay of a patient using a hospital bed at an NHS organisation under the care of one or more consultants, or nursing episode or midwife episode), and the HES-Office of National Statistics (ONS) linked mortality data (Table S1) (20). The latter captures deaths that occur outside of hospital. Data used to calculate the SHMI are submitted by each hospital trust.

SHMI expected deaths is calculated based on individual patient characteristics that can affect the risk of mortality, including the patient’s condition for hospitalisation, underlying conditions (Charlson Comorbidity Index) (21), age, gender, method of admission to hospital, and year of discharge. Logistic regression models estimate the risk for each provider spell, with binary variable as outcome (*i.e.* died or survived). The SHMI model performs well with early validation accounting for 81% of between-hospital variations with a c-statistic (area under the receiver operator curve) of 0.9 (19). The models are based on the preceding three years’ data, with last year data used to calculate the SHMI (22). As SHMI data are published monthly, we used the dataset that contains mortality data from April to March the following year to report on annual hospital mortality level for each hospital trust included in this study.

Study variables

We obtained hospital staffing data by linking the medical and dental workforce dataset with the non-medical workforce dataset. Bed occupancy data are published quarterly as averages, with no estimate of monthly variation. For wards open overnight, occupied bed is defined as when it is occupied at midnight on the day in question; for day-only wards, occupied bed is a bed where at least one day case has taken place during the day. Trust teaching status was derived from the ERIC dataset, which contains variables on trust profile.

Staffing variables are published monthly. We used full-time equivalent (FTE) values to calculate the annual average available staff at each hospital trust from April to March the following year, to align with the SHMI data. FTE data are based on the proportion of time each staff are expected to work in a week, which would correspond to an FTE of 1 (*e.g.* 48

For peer review only - http://bmjopen.bmj.com/site/about/guidelines.xhtml

hours for doctors, 37.5h for nurses). Overtime and out-of-hour work are not recorded in these datasets.

Clinical staff are classified according to occupation codes. We grouped staffing variables by frequency and occupation as listed by NHS England, merging groups that had mean below 3 with others of a similar occupation in order to create 8 groups (*i.e.* medical, surgical, other medical specialties, nurses, support to nurses, AHP, support to AHP, and scientific, therapeutic, and technical (ST&T) staff) (see S1 Appendix for details).

We used the number of general acute occupied beds to calculate the bed-per-staff ratio. Beds assigned to maternity, mental illness, and learning disabilities services were excluded; however, general acute beds represented 96.2% of the total beds available across all hospital trusts included in the study. We calculated the average number of occupied beds overnight or day-only per trust per year, and then divided occupied beds per each staff group to obtain the average bed-per-staff levels for each hospital trust per year.

Teaching affiliation was coded as yes or no according to the trust type recorded in the ERIC dataset. Trust size was calculated based on the number of available general acute beds in each trust. There was a median of 744.2 available general acute beds per trust (interquartile range (IQR) 535.7 to 1009.4, range 143 to 2704). Hospital trusts in the upper tertile (*i.e.* the upper third of trusts, ranked by size) were classified as large, while those in the lower tertile were deemed small trusts, with the remaining classified as medium-sized hospital trusts.

Observations with missing data were removed from the analyses; however, hospital trusts that reported at least one year of complete data were included. There were 540 observations in the linked dataset, of which 519 (96%) contained data on all variables and were therefore included in our analyses. There were 2 hospital trusts that did not report on medical staffing in 2016, with 6 trusts not reporting these variables from 2017 onwards. For other clinical staff, only 1 trust did not report staff numbers in 2016 and 2017, and two trusts in 2018, with no missing data in 2019. One trust did not report the number of occupied general acute beds in 2018. The number of hospital trusts that reported on complete data varied, with 137 included in 2016 and 2017, 135 in 2018, and 130 in 2019.

Statistical analysis

We initially explored the data using descriptive statistics. Continuous variables were described as mean and standard deviation (SD) or median and IQR, depending on the distribution of each variable. Categorical variables were described as frequencies and proportions. Annual mortality rates were calculated by dividing the number of observed deaths by the number of patient spells, across all hospital trusts each year. To explore the relationship between staffing levels and hospital mortality, statistical modelling was conducted at the hospital trust-level using multiple regression models on four years of data, with standardised mortality rates regressed onto hospital level staffing levels (expressed as the number of occupied beds-per-staff). We included expected deaths as an offset, as the number of expected deaths is the number of times the event could have happened (*i.e.* the exposure variable).

Multilevel models were adjusted for trust size and affiliation as a teaching hospital, with trust included as a random effect to adjust for clustering. We considered a range of potential models, with model selection based on minimising the Akaike information criterion (AIC), the Bayesian information criterion (BIC) and the likelihood ratios (see Table S2 for alternative frameworks considered). We report exponentiated coefficients (rate ratios) with 95% confidence intervals (CI) for all estimates obtained from the models.

For peer review only - http://bmjopen.bmj.com/site/about/guidelines.xhtml

The best performing model (referred to as main model) was a negative binomial random effects model that included all 8 clinical staff groups and hospital characteristics (*i.e.* trust size and teaching status). To explore variations between and within hospital trusts, we also constructed a within-between random effects model (WBRE), with trusts as random effect, and staffing and hospital characteristics as fixed effects. These models can differentiate between effects arising from staffing differences between hospital trusts and those that are associated with annual changes of staffing levels within each hospital (23, 24).

We assessed potential collinearity between co-variates using correlation plots and Spearman's correlation coefficient, and the overall multicollinearity for all predictors in the models using generalised variance inflation factors (GVIF). All models had a GVIF<10. As a sensitivity analysis, we reran the models after excluding any variables that had a GVIF above 5 (25).

We performed data linkage, cleaning, coding, and analyses in R statistical software, version 4.0.2 (R Core Team, R Foundation for Statistical Computing, Vienna, Austria), using the lme4 and glmer packages (v1.1-27.1, Bates et al, 2015), and the plm package (v2.4-3, Croissant et al, 2021) (26).

Patient and public involvement

There was no patient and public involvement in this study.

Results

Descriptive statistics

Our final sample consisted of 519 observations of staffing, bed occupancy and mortality variables for the 4-year study period, clustered within 138 hospital trusts. The median number of acute general occupied beds per day was 674.0 (IQR 486.1 to 911.6). The mean annual observed deaths ± SD was 2229 ± 992 (range 525 to 7468), while the expected deaths were 2228 ± 987.2 (range 665 to 7591). Annual mortality rates across all hospital trusts were 3.44% for 2016, 3.46% for 2017, 3.23% for 2018, and 3.31% for 2019. Forty-one (29.7%) hospital trusts were classified as teaching trusts.

Table 1 shows the distribution of staffing variables by their median, IQR, mean, and SD across all hospital trusts. There was considerable variation in staffing levels, with nurse staff levels showing least relative variation (SD 18.0% of the mean) and AHP support most (SD 44.4% of the mean). By contrast, variations within trusts as a percentage of the mean were relatively low, with the within trust variation ranging from 4.9% for nurse staffing to 10.9% for AHP support.

Table 1. Frequency of occupied bed-per-staff variables across 138 hospital trusts

Staffing level variables	Q1	Median	Q3	Mean	SD	SD as % of mean	Mean within-trust SD	Within-trust SD as % of mean
Bed per medical	3.05	3.66	4.30	3.70	0.96	25.9%	0.28	7.6%

For peer review only - http://bmjopen.bmj.com/site/about/guidelines.xhtml

Staffing level variables	Q1	Median	Q3	Mean	SD	SD as % of mean	Mean within-trust SD	Within-trust SD as % of mean
Bed per surgical	3.10	3.79	4.28	3.73	0.81	21.7%	0.22	5.9%
Bed per other medical specialties	3.26	3.96	4.69	3.94	1.01	25.6%	0.27	6.9%
Bed per nurse adult service	0.54	0.61	0.68	0.61	0.11	18.0%	0.03	4.9%
Bed per nurse support	0.80	0.93	1.08	0.93	0.21	22.6%	0.07	7.5%
Bed per AHP	1.88	2.25	2.74	2.36	0.89	37.7%	0.18	7.6%
Bed per AHP support	7.91	10.03	13.01	11.07	4.91	44.4%	1.21	10.9%
Bed per ST&T	1.80	2.24	2.82	2.31	0.75	32.5%	0.16	6.9%

Q1: lower quartile, Q3: upper quartile, SD: standard deviation, AHP: allied healthcare professional, ST&T: scientific, therapeutic, and technical

Nurse staffing levels were strongly correlated with staffing levels in all medical staff groups (rho>0.71). Nurse support staff levels were correlated to RN (rho=0.30), as were AHP and AHP support (rho=0.47).

Staffing levels and hospital mortality

Table 2 shows results for the negative binomial random effect models, adjusted for hospital characteristics. In the single staff group models, hospital trusts that had lower staffing levels (*i.e.* more occupied beds per FTE staff) were associated with higher (standardised) mortality rates for all professionally qualified staff groups. The opposite effect was observed for nursing support and AHP support staff, where hospital trusts with lower staffing levels (*i.e.* more occupied beds per FTE staff) had lower mortality.

Table 2. Exponentiated estimates for hospital mortality from the multilevel negative binomial random effect models, adjusted for hospital characteristics (n = 138)

Predictors	Estimates for single staff models	95% CI	Estimates for multiple staff model	95% CI
Bed per medical	1.05***	1.04 to 1.07	1.04***	1.02 to 1.06
Bed per surgical	1.04***	1.02 to 1.06	0.98	0.96 to 1.01
Bed per other medical specialties	1.05***	1.03 to 1.06	1.03*	1.00 to 1.06
Bed per nurse adult service	1.33***	1.15 to 1.54	1.07	0.88 to 1.31

For peer review only - http://bmjopen.bmj.com/site/about/guidelines.xhtml

Predictors	Estimates for single staff models	95% CI	Estimates for multiple staff model	95% CI
Bed per nurse support	0.92*	0.86 to 0.98	0.85***	0.79 to 0.91
Bed per AHP	1.02*	1.00 to 1.04	1.04***	1.02 to 1.06
Bed per AHP support	0.99*	0.99 to 0.99	1.00**	0.99 to 1.00
Bed per ST&T	1.02**	1.00 to 1.04	0.99	0.98 to 1.05
Teaching (ref not teaching)	0.96*	0.92 to 0.99	1.01	0.98 to 1.05
Medium trust (ref small trust)	1.01	0.97 to 1.05	1.01	0.98 to 1.04
Large trust (ref small trust)	1.00	0.96 to 1.04	1.01	0.98 to 1.05

CI: confidence interval, AHP: allied healthcare professional, ST&T: scientific, therapeutic, and technical, * p<0.05, ** p<0.01, *** p<0.001

In the main multivariable model including all staff groups, associations between medical (1.04, 95%CI 1.02 to 1.06), other medical specialties (1.03, 95%CI 1.00 to 1.06), AHP (1.04, 95%CI 1.02 to 1.06), nurse support (0.85, 95%CI 0.79 to 0.91), and AHP support (1.00, 95%CI 0.99 to 1.00) FTE staffing levels and hospital mortality remained statistically significant, with no change in direction of the effect compared to the single staff models (Table 2). Higher mortality rates were observed in hospital trusts with lower levels of medical and AHP staff. In contrast, hospital trusts with lower support staff levels per occupied bed (*i.e.* nursing support and AHP support) had lower mortality rates. The association with RN staffing was attenuated and no longer statistically significant (1.07, 95%CI 0.88 to 1.31), although the observed effect remained relatively large compared to other associations (Figure S1). Similarly, the association between hospital mortality and ST&T staff, observed in the single staff groups models, were no longer statistically significant in the model adjusted for all staff groups.

Although we noted correlation between staffing variables, our GVIF for all variables was <10 which is generally accepted as indicative of no evidence of multicollinearity (27). The GVIF for beds per other medical specialties was 6.1 (Table S3), and therefore over our threshold of 5, so we ran the negative binomial random effects model omitting this variable as a sensitivity analysis and found similar results (not shown; available from authors upon request).

Within-between hospital trust variability

Between-hospital trust effects from the WBRE model were largely the same as those obtained from the main model (*i.e.* between only). For example, the between-estimate from WBRE for the FTE medical group was 1.04 (95%CI 1.02 to 1.07), similar to the estimates obtained from the main model (1.04, 95%CI 1.02 to 1.06). However, even for staff groups where between-hospital trusts effects were significant, the within-hospital effects were small and not statistically significant. For example, the within-hospital estimate from the WBRE model for the medical group was 0.99 (95%CI 0.97 to 1.01) (Fig 1). Despite potentially providing additional information by decomposing the variability into between- and within-estimates, the WBRE model did not improve fit compared to the main model (Table S2).

For peer review only - http://bmjopen.bmj.com/site/about/guidelines.xhtml

Figure 1. Hospital mortality exponentiated estimates obtained from the within-between random effect model (WBRE).

Discussion

After adjustment for hospital characteristics and including multiple clinical staff groups in our models, we found that higher medical and AHP staffing levels were associated with lower mortality rates in acute hospital trusts in England during the period of 2015 to 2019. In contrast, hospitals with higher support staff per occupied bed had higher mortality rates.

Our study highlights the importance of simultaneously adjusting for multiple clinical staff groups when investigating associations between staffing levels and mortality at the hospital-level. In the single staff group models, significant effects on mortality rates were seen for all staffing groups. However, when adjusted for multiple groups, effects for medical, other medical specialties, AHP, nurse support, and AHP support staffing levels remained statistically significant, but levels of surgical doctors, RN, and ST&T staff were no longer significant.

Our findings are in line with previous research. Griffiths *et al* (15) reported that higher levels of occupied beds per RN and per doctor were associated with increased mortality in the single staff models, while the association was reversed for healthcare support workers. However, there was a substantial attenuation in nurse staffing estimates when medical staffing levels were included. Jarman *et al* (3) showed that the association between RN staffing and mortality was no longer significant when adjusting for doctors-per-bed, with higher levels of physician staffing significantly reducing hospital standardised mortality ratios.

Other studies have shown a reduction in hospital mortality when higher numbers of doctors and nurses per bed were observed (12, 15, 28-33). In our study, although the largest estimate of effect on patient mortality was for nurse staffing, this was not statistically significant in the main model, which was adjusted for all clinical staff groups. Studies have reported that much of the variation in nurse staffing occurs between wards within hospitals (34, 35) and Keogh noted that hospital nurse staffing levels often bore little relationship to staffing available to be deployed on wards in NHS hospitals (36), therefore hospital nurse staffing per bed may be a poor indicator of the staffing levels experienced by inpatients. Griffiths *et al* (15) found that when medical staffing levels were included in models, significant nurse staffing effects were only observed in models using ward-based staffing ratios as opposed to hospital-level staff-per-bed ratios. Longitudinal patient level studies of exposure to variation in nurse staffing confirm that there is an adverse effect of low nurse staffing when measured at this level (37-39).

In our study, high levels of nurse support staff were associated with higher mortality rates. Bond *et al* (28) showed that higher staffing levels of medical residents, RN, pharmacists, and medical technologists resulted in lower mortality rates, while nurse support and administrators had the opposite effect. The reasons for the reverse effect of support staff on mortality are unclear; however, they could include reduction of skill mix by increasing the proportion of support staff in relation to professionally qualified staff, and increased workload of professionally qualified staff due to additional supervisory tasks when support staff levels are higher (40).

To our knowledge, this was the first study to include AHP and AHP support staff levels in analysis. Higher levels of AHP staff had a protective effect at the hospital trust level while more AHP support staff were detrimental to patient mortality, a finding that mirrors those obtained for nursing support staff. While causality cannot be assumed for any of these results, there is a potential that previous findings that have emphasised a link between nurse staffing and patient safety could divert attention from the role that other staff groups may play in delivering quality and preventing avoidable deaths.

For peer review only - http://bmjopen.bmj.com/site/about/guidelines.xhtml

We found that variations between hospital trusts in the 4-year study period were higher in magnitude than within-hospital trusts estimates, which were not statistically significant for any of the clinical staff groups at the hospital level. Structural recruitment and retention difficulties, and financial constraints could be main contributors to inter-hospital trust variations. There was relatively little year on year variation in staffing between trusts. Reduced variability of staffing levels within hospitals might be due to the limited number of funded posts in each organisation and could reflect historic staffing levels. Bjerregaard *et al* (33), using a WBRE model, found staffing levels estimates were significant between wards, while within-ward estimates had no significant effect on hospital mortality.

Strengths and limitations

This was a national study using 4 years of routinely available data. We considered the effects of staffing by professionally qualified and support workers on in-hospital and 30-day mortality rates. The inclusion of numerous clinical staffing groups simultaneously in the models allowed us to estimate the effect of each staffing level on hospital mortality while considering all other clinical staff levels and hospital trust characteristics.

We used a variety of models and selected the one that performed best on our data. The use of different methodological approaches highlighted how different assumptions about the distribution of staffing variables and the nature of staffing between and within hospitals can impact model performance and findings derived from such models. Most studies investigating clinical staffing levels and in-hospital mortality used fixed effects models, which only evaluate within-hospital effects. While these might be useful when estimating the impact of interventions on staffing levels within a hospital, they might not appropriately assess the effect of different staffing levels based on variations between hospitals in hospital-level observational studies. We showed that between-hospital effects are larger and generally dictate the relationship between clinical staffing levels and mortality. In fact, some of the estimates of within-hospital staffing levels had the opposite direction to the between-hospital estimates on hospital mortality.

However, our study was limited by the relatively small sample size, as observations were clustered on 138 hospital trusts. We therefore had to group some of the medical specialties and were unable to unpack relatively heterogenous groups such as AHP. Furthermore, we were unable to explore the effects of grade and experience for medical (*e.g.* junior doctors, level of consultants) and RN (*e.g.* senior nurses) staff.

In our study, beds that were occupied by multiple patients in a single day were counted as one single occupied bed per staff. Studies have shown that workload (*e.g.* number of admissions, discharges, additional tasks) in high volume hospitals can lead to higher mortality rates, even when levels of staffing are similar to those observed in low volume hospitals. Occupied beds-per-staff might not be a good measure of staffing levels, as these do not take into account temporary staff or staff absence due to sickness, maternity leave or long-term leave. The extent to which staff are deployed to deliver services such as ambulatory care or other services not delivered to inpatient beds is likely to vary (41).

Future studies should aim to capture different staffing levels responsible for delivering patient care measured at the patient or ward-level, rather than at the hospital-level.

Although SHMI provides good control for patient risk and we controlled for a number of hospital factors and multiple staff groups, the cross-sectional nature of our analysis means that findings need to be interpreted as demonstrating association but not direct evidence of causation.

For peer review only - http://bmjopen.bmj.com/site/about/guidelines.xhtml

Conclusion

In conclusion, our study highlights the importance of simultaneously considering multiple staff groups when investigating the effect of clinical staffing levels and mortality at the hospital-level. We showed that the number of AHP and AHP support per occupied beds have a significant impact on patient mortality, yet these groups have largely not been included in previous workforce studies, of which the majority focused exclusively on nursing staff. We also found that hospitals with higher levels of medical staffing had lower mortality rates, while higher levels of support workers were associated with higher hospital mortality.

Data Statement

The dataset is available from the ePrints repository, DOI: https://doi.org/10.5258/SOTON/D2055

Authors' contributions

All authors made substantial contributions to the conception or design of the work or the acquisition, analysis, or interpretation of data for the work; drafted the work or revised it critically for important intellectual content; gave Final approval of the version to be published; agree to be accountable for all aspects of the work in ensuring that questions related to the accuracy or integrity of any part of the work are appropriately investigated and resolved.

Conflicts of interest

All authors have completed the ICMJE uniform disclosure form at http://www.icmje.org/disclosure-of-interest/ and declare: all authors had financial support from the NIHR Health Services and Delivery Research for the submitted work; no financial relationships with any organisations that might have an interest in the submitted work in the previous three years; no other relationships or activities that could appear to have influenced the submitted work."

Funding

This study/project is funded by the NIHR [Health and Social Care Delivery Research (NIHR128056)]. The views expressed are those of the author(s) and not necessarily those of the NIHR or the Department of Health and Social Care.

Ethics Approval

We did not need ethics approval as this project relied on publicly available routinely collected data.

For peer review only - http://bmjopen.bmj.com/site/about/guidelines.xhtml

References

1. Kutney-Lee A, Sloane DM, Aiken LH. An increase in the number of nurses with baccalaureate degrees is linked to lower rates of postsurgery mortality. Health Aff (Millwood). 2013;32(3):579-86.
2. Aiken LH, Clarke SP, Cheung RB, Sloane DM, Silber JH. Educational levels of hospital nurses and surgical patient mortality. Jama. 2003;290(12):1617-23.
3. Jarman B, Gault S, Alves B, Hider A, Dolan S, Cook A, et al. Explaining differences in English hospital death rates using routinely collected data. Bmj. 1999;318(7197):1515-20.
4. Kane RL, Shamliyan TA, Mueller C, Duval S, Wilt TJ. The association of registered nurse staffing levels and patient outcomes: systematic review and meta-analysis. Medical Care. 2007;45(12):1195-204.
5. Shekelle PG. Nurse-patient ratios as a patient safety strategy: A systematic review. Annals of Internal Medicine. 2013;158(5 PART 2):404-9.
6. Griffiths P, Ball J, Drennan J, Dall'Ora C, Jones J, Maruotti A, et al. Nurse staffing and patient outcomes: Strengths and limitations of the evidence to inform policy and practice. A review and discussion paper based on evidence reviewed for the National Institute for Health and Care Excellence Safe Staffing guideline development. Int J Nurs Stud. 2016;63:213-25.
7. Blume KS, Dietermann K, Kirchner-Heklau U, Winter V, Fleischer S, Kreidl LM, et al. Staffing levels and nursing-sensitive patient outcomes: Umbrella review and qualitative study. Health Serv Res. 2021;56(5):885-907.
8. Pronovost PJ, Angus DC, Dorman T, Robinson KA, Dremsizov TT, Young TL. Physician staffing patterns and clinical outcomes in critically ill patients: a systematic review. Jama. 2002;288(17):2151-62.
9. Lin HC, Lee HC. Caseload volume-outcome relation for pulmonary embolism treatment: Association between physician and hospital caseload volume and 30-day mortality. Journal of Thrombosis and Haemostasis. 2008;6(10):1707-12.
10. Ong M, Bostrom A, Vidyarthi A, McCulloch C, Auerbach A. House staff team workload and organization effects on patient outcomes in an academic general internal medicine inpatient service. Archives of Internal Medicine. 2007;167(1):47-52.
11. Coit MH, Katz JT, McMahon GT. The effect of workload reduction on the quality of residents' discharge summaries. J Gen Intern Med. 2011;26(1):28-32.
12. Ricciardi R, Nelson J, Roberts PL, Marcello PW, Read TE, Schoetz DJ. Is the presence of medical trainees associated with increased mortality with weekend admission? BMC Medical Education. 2014;14:4.
13. Bond CA, Raehl CL, Franke T. Interrelationships among mortality rates, drug costs, total cost of care, and length of stay in United States hospitals: summary and recommendations for clinical pharmacy services and staffing. Pharmacotherapy:The Journal of Human Pharmacology & Drug Therapy. 2001;21(2):129-41.
14. Lee H, Ryu K, Sohn Y, Kim J, Suh GY, Kim E. Impact on Patient Outcomes of Pharmacist Participation in Multidisciplinary Critical Care Teams: A Systematic Review and Meta-Analysis*. Critical Care Medicine. 2019;47(9).
15. Griffiths P, Ball J, Murrells T, Jones S, Rafferty AM. Registered nurse, healthcare support worker, medical staffing levels and mortality in English hospital trusts: a cross-sectional study. BMJ Open. 2016;6(2):e008751.
16. NHS Digital. National Workforce Data Set (NWD) and NHS occupational codes [Available from: https://digital.nhs.uk/data-and-information/data-collections-and-data-sets/data-sets/national-workforce-data-set-nwd-and-nhs-occupation-codes#top.
17. NHS England. NHS Data Model and Dictionary: NHS Digital; [updated September 2021. Available from: https://datadictionary.nhs.uk/index.html.

For peer review only

18. NHS England. The processing cycle and HES data quality NHS Digital: NHS England; [Available from: https://digital.nhs.uk/data-and-information/data-tools-and-services/data-services/hospital-episode-statistics/the-processing-cycle-and-hes-data-quality.
19. Campbell MJ, Jacques RM, Fotheringham J, Maheswaran R, Nicholl J. Developing a summary hospital mortality index: retrospective analysis in English hospitals over five years. Bmj. 2012;344:e1001.
20. Aylin P, Williams S, Bottle A, Jarman B. Counting hospital activity: spells or episodes? BMJ. 2004;329(7476):1207.
21. Charlson ME, Pompei P, Ales KL, MacKenzie CR. A new method of classifying prognostic comorbidity in longitudinal studies: development and validation. J Chronic Dis. 1987;40(5):373-83.
22. Campbell MJ, Jacques, R. M., Fotheringham, J., Peatson, T., Maheswaran, R., Nicholl, J. An evaluation of the Summary Hospital Mortality Index. The University of Sheffield; 2011 21/04/2011.
23. Bell A, Fairbrother M, Jones K. Fixed and random effects models: making an informed choice. Quality & Quantity. 2019;53:1051-74.
24. Schunck R. Within and between Estimates in Random-Effects Models: Advantages and Drawbacks of Correlated Random Effects and Hybrid Models. The Stata Journal. 2013;13:65 - 76.
25. O’brien RM. A Caution Regarding Rules of Thumb for Variance Inflation Factors. Quality & Quantity. 2007;41(5):673-90.
26. R Core Team. A Language and Enviroment for Statistical Computing. Vienna, Austria: R Foundation for Statistical Computing; 2020.
27. James G WD, Hastie T, Tibshirani R. An Introduction to Statistical Learning: with Applications in R. 1st ed. ed: Springer; 2013. 440 p.
28. Bond CA, Raehl CL, Pitterle ME, Franke T. Health care professional staffing, hospital characteristics, and hospital mortality rates. Pharmacotherapy:The Journal of Human Pharmacology & Drug Therapy. 1999;19(2):130-8.
29. Griffiths P, Jones S, Bottle A. Is "failure to rescue" derived from administrative data in England a nurse sensitive patient safety indicator for surgical care? Observational study. Int J Nurs Stud. 2013;50(2):292-300.
30. Ozdemir BA, Sinha S, Karthikesalingam A, Poloniecki JD, Pearse RM, Grocott MP, et al. Mortality of emergency general surgical patients and associations with hospital structures and processes. British Journal of Anaesthesia. 2016;116(1):54-62.
31. Chung W, Sohn M. The Impact of Nurse Staffing on In-Hospital Mortality of Stroke Patients in Korea. Journal of Cardiovascular Nursing. 2018;33(1):47-54.
32. West E, Barron DN, Harrison D, Rafferty AM, Rowan K, Sanderson C. Nurse staffing, medical staffing and mortality in Intensive Care: An observational study. International Journal of Nursing Studies. 2014;51(5):781-94.
33. Bjerregaard U, Hølge-Hazelton, Bibi, Rud Kristensen, Søren, Rose Olsen, Kim. Nurse staffing and patient outcomes: Analyzing within- and between-variation. DaCHE discussion papers. 2020;3.
34. Griffiths P, Ball J, Bloor K, Böhning D, Briggs J, Dall’Ora C, et al. Nurse staffing levels, missed vital signs and mortality in hospitals: retrospective longitudinal observational study. Health Services and Delivery Research 2018.
35. Van den Heede K, Sermeus W, Diya L, Clarke SP, Lesaffre E, Vleugels A, et al. Nurse staffing and patient outcomes in Belgian acute hospitals: cross-sectional analysis of administrative data. Int J Nurs Stud. 2009;46(7):928-39.
36. Keogh BE. Review into the quality of care and treatment provided by 14 hospitals trusts in England: overview report. NHS England; 2013.
37. Dall’Ora C, Saville C, Rubbo B, Turner LY, Jones J, Griffiths P. Nurse staffing levels and patient outcomes: a systematic review of longitudinal studies. medRxiv. 2021:2021.09.17.21263699.
38. Griffiths P, Maruotti A, Recio Saucedo A, Redfern OC, Ball JE, Briggs J, et al. Nurse staffing, nursing assistants and hospital mortality: retrospective longitudinal cohort study. BMJ Quality & Safety. 2019;28(8):609.

For peer review only - http://bmjopen.bmj.com/site/about/guidelines.xhtml

39. Needleman J, Buerhaus P, Pankratz VS, Leibson CL, Stevens SR, Harris M. Nurse staffing and inpatient hospital mortality. New England Journal of Medicine. 2011;364(11):1037-45.
40. Griffiths P, Maruotti A, Recio Saucedo A, Redfern OC, Ball JE, Briggs J, et al. Nurse staffing, nursing assistants and hospital mortality: Retrospective longitudinal cohort study. BMJ Quality and Safety. 2019;28(8):609-17.
41. Ball J. Nurse staffing levels, care left undone, & patient mortality in acute hospitals [Thesis for doctoral degree]. Stockholm, Sweden: Karolinska Institutet; 2017.

Supporting information captions

Table S1. NHS digital data sources

Table S2. Comparison of goodness-of-fit for model selection

Table S3. Multicollinearity test in the main model (*i.e.* negative binomial random effect)

Figure S1. Hospital mortality estimates obtained from the main model (*i.e.* negative binomial random effect).

For peer review only

For peer review only - http://bmjopen.bmj.com/site/about/guidelines.xhtml

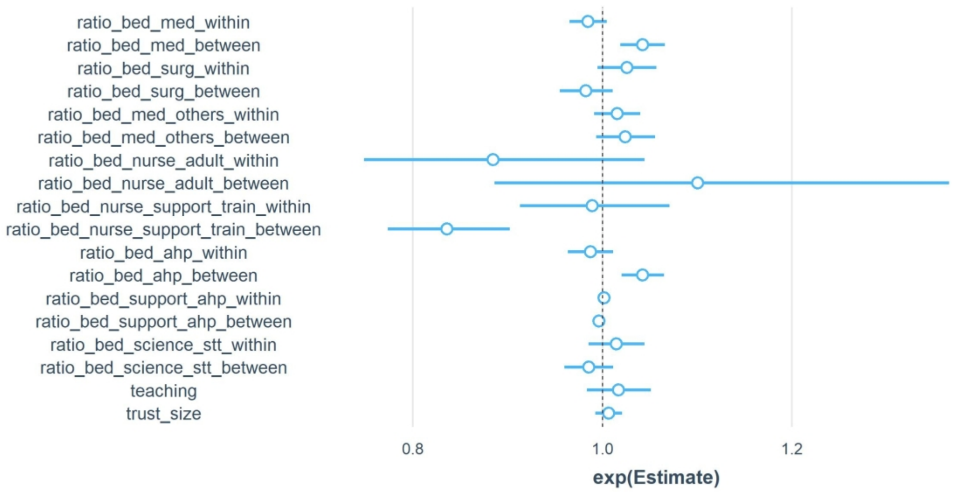

Hospital mortality exponentiated estimates obtained from the within-between random effect model (WBRE).

199x99mm (300 x 300 DPI)

Supporting information

Staff variable groups

General medicine specialist doctors were grouped with clinical oncology doctors to form the general medicine group, surgeons were grouped with obstetricians and gynaecologists to form the surgical group, and all other medical specialties were grouped together to form the other medical group (*i.e.* radiologists, emergency medicine, anaesthetists, and pathologists). We excluded psychiatrists and paediatricians. The nurses' group was composed of nurses in adult services only; we did not include nurses from paediatric services. The nurse support group included support to adult and general nurses, and nurses in training. Staff in the fields of podiatry and chiropody, dietetics, occupational therapy, orthoptics and optics, physiotherapy, radiography, art, music and drama therapy, and speech and language therapy were included in the allied health professional (AHP) group. Scientific, therapeutic, and technical (ST&T) staff group was composed of multi-therapists, applied psychologist, psychological therapist, pharmacists, dentists, operating theatre staff, social services, and other ST&T staff.

Tables and figures

Table S1. NHS digital data sources

Data source	Datasets	Main variables available
NHS workforce data	Medical dataset	Professionally qualified clinical staff
	Non-medical dataset	Professionally qualified clinical staff
		Support to clinical staff
Bed occupancy data	Overnight beds dataset	Available beds
		Occupied beds
		% occupied beds
	Day only beds datasets	Available beds
		Occupied beds
		% occupied beds
ERIC data		Trust profile (e.g. type)
		Strategies and policies
		Finance
		Safety
		Fire and safety

For peer review only

Data source	Datasets	Main variables available
		Business transport
		Medical records
		Key worker accommodation
		Facilities management services
		Areas
		Function and space
		Quality of buildings
		Combined heat and power energy
		Energy
		Water services
		Waste
SHMI data		SHMI value
		SHMI banding
		Number of spells
		Observed deaths
		Expected deaths

NHS: National Health Service, ERIC: Estates Returns Information Collection, SHMI: Summary Hospital level Mortality Indicator

Table S2. Comparison of goodness-of-fit for model selection

**Model**	**Correlations for trusts**	**Model degrees of freedom**	**AIC**	**BIC**	**Likelihood-ratio**
OLS	No	17	8183.5	8255.8	-4074.7
Poisson	No	16	10567.6	10635.7	-5267.8
Negative binomial	No	17	6692.6	6764.9	-3329.3
GLS	Yes	26	7517.0	7627.6	-3732.5
GLS exchangeable	Yes	18	7867.5	7944.0	-3915.7
GLS Toeplitz (2)	Yes	19	7823.9	7904.7	-3892.9
GLS AR	Yes	18	7822.7	7899.2	-3893.3
Poisson RE	Yes	14	1997.8	2057.3	-984.9
**Negative binomial RE**	**Yes**	**14**	**250.3**	**309.9**	**-111.2**
Poisson FE	Yes (dummy)	146	6477.4	7098.2	-3092.7
Negative binomial FE	Yes (dummy)	147	6180.5	6805.6	-2943.3
Negative binomial WBRE	Yes	22	6418.3	6511.8	-3187.1

For peer review only

AIC: Akaike information criterion, BIC: Bayesian information criterion, OLS: ordinary least squares, GLS: generalised least squares, AR: autoregressive, RE: random effects, FE: fixed effects, WBRE: within-between random effects, (2) tridiagonal 2-Toeplitz matrix

Table S3. Multicollinearity test in the negative binomial random effects model

Variable	GVIF
Beds per medical	3.1
Beds per surgical	3.6
Beds per other medical	6.1
Beds per nurse	3.7
Beds per nurse support	1.7
Beds per AHP	2.0
Beds per AHP support	1.4
Beds per ST&T	2.6
Teaching status	1.8
Trust size	1.1*

GVIF: generalised variance inflation factor; AHP: allied health professionals; ST&T: scientific, therapeutic, and technical; *reported on GVIF^(1/(2*Df)) instead of GVIF as variable has 2 degrees of freedom

For peer review only

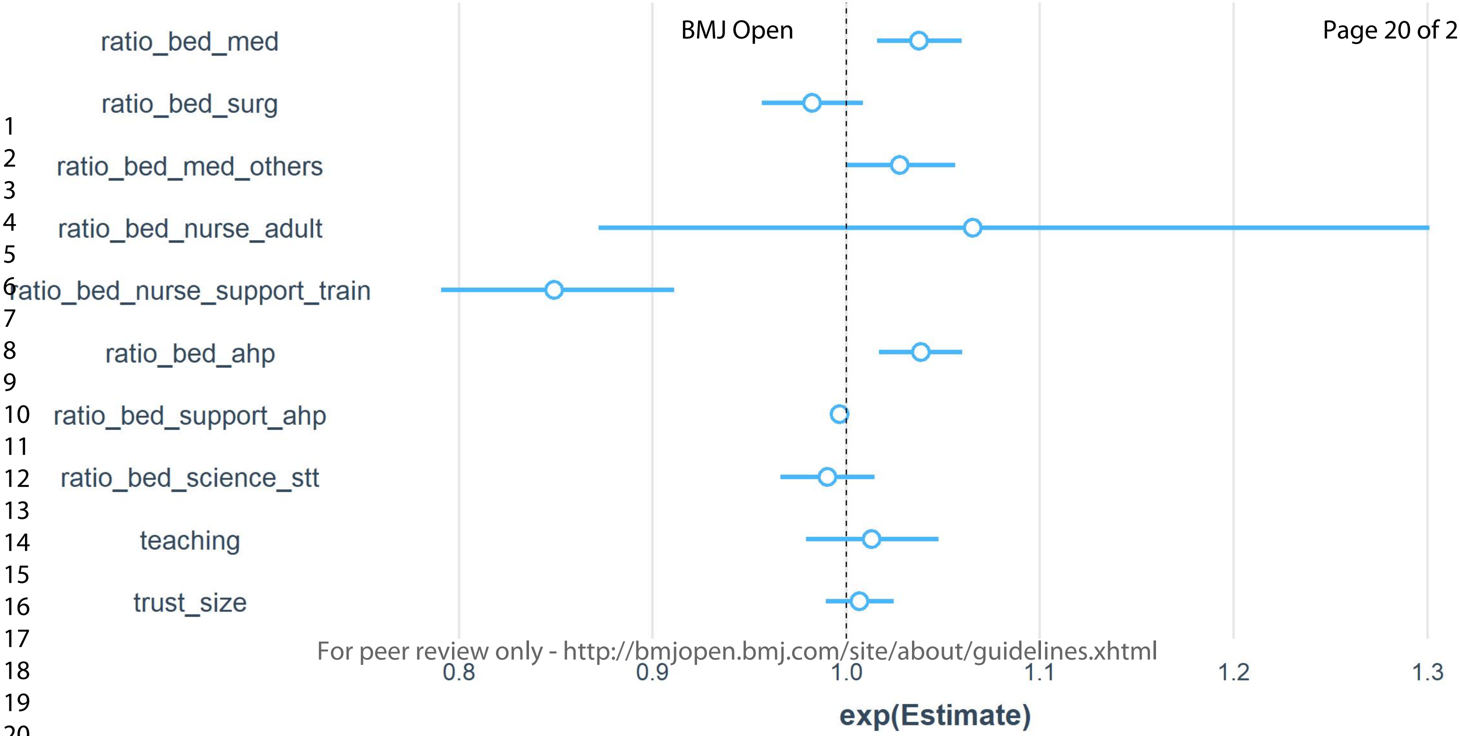

For peer review only - http://bmjopen.bmj.com/site/about/guidelines.xhtml

STROBE Statement—Checklist of items that should be included in reports of ***cross-sectional studies***

	Item No	Recommendation	Page No
**Title and abstract**	1	(*a*) Indicate the study's design with a commonly used term in the title or the abstract	1
		(*b*) Provide in the abstract an informative and balanced summary of what was done and what was found	2
**Introduction**			
Background/rationale	2	Explain the scientific background and rationale for the investigation being reported	3
Objectives	3	State specific objectives, including any prespecified hypotheses	3
**Methods**			
Study design	4	Present key elements of study design early in the paper	3
Setting	5	Describe the setting, locations, and relevant dates, including periods of recruitment, exposure, follow-up, and data collection	3
Participants	6	(*a*) Give the eligibility criteria, and the sources and methods of selection of participants	3
Variables	7	Clearly define all outcomes, exposures, predictors, potential confounders, and effect modifiers. Give diagnostic criteria, if applicable	4-5
Data sources/ measurement	8*	For each variable of interest, give sources of data and details of methods of assessment (measurement). Describe comparability of assessment methods if there is more than one group	4-5
Bias	9	Describe any efforts to address potential sources of bias	3-5
Study size	10	Explain how the study size was arrived at	3
Quantitative variables	11	Explain how quantitative variables were handled in the analyses. If applicable, describe which groupings were chosen and why	3-5
Statistical methods	12	(*a*) Describe all statistical methods, including those used to control for confounding	5-6
		(*b*) Describe any methods used to examine subgroups and interactions	
		(*c*) Explain how missing data were addressed	
		(*d*) If applicable, describe analytical methods taking account of sampling strategy	
		(*e*) Describe any sensitivity analyses	
**Results**			
Participants	13*	(a) Report numbers of individuals at each stage of study—eg numbers potentially eligible, examined for eligibility, confirmed eligible, included in the study, completing follow-up, and analysed	6
		(b) Give reasons for non-participation at each stage	
		(c) Consider use of a flow diagram	
Descriptive data	14*	(a) Give characteristics of study participants (eg demographic, clinical, social) and information on exposures and potential confounders	6
		(b) Indicate number of participants with missing data for each variable of interest	
Outcome data	15*	Report numbers of outcome events or summary measures	6
Main results	16	(*a*) Give unadjusted estimates and, if applicable, confounder-adjusted estimates and their precision (eg, 95% confidence interval). Make clear which confounders were adjusted for and why they were included	7-8

For peer review only - http://bmjopen.bmj.com/site/about/guidelines.xhtml

		(*b*) Report category boundaries when continuous variables were categorized	7-8
		(*c*) If relevant, consider translating estimates of relative risk into absolute risk for a meaningful time period	
Other analyses	17	Report other analyses done—eg analyses of subgroups and interactions, and sensitivity analyses	8
**Discussion**			
Key results	18	Summarise key results with reference to study objectives	8
Limitations	19	Discuss limitations of the study, taking into account sources of potential bias or imprecision. Discuss both direction and magnitude of any potential bias	9-10
Interpretation	20	Give a cautious overall interpretation of results considering objectives, limitations, multiplicity of analyses, results from similar studies, and other relevant evidence	8-10
Generalisability	21	Discuss the generalisability (external validity) of the study results	8-10
**Other information**			
Funding	22	Give the source of funding and the role of the funders for the present study and, if applicable, for the original study on which the present article is based	11

*Give information separately for exposed and unexposed groups.

Note: An Explanation and Elaboration article discusses each checklist item and gives methodological background and published examples of transparent reporting. The STROBE checklist is best used in conjunction with this article (freely available on the Web sites of PLoS Medicine at http://www.plosmedicine.org/, Annals of Internal Medicine at http://www.annals.org/, and Epidemiology at http://www.epidem.com/). Information on the STROBE Initiative is available at www.strobe-statement.org.

For peer review only

Staffing levels and hospital mortality in England: a national panel study using routinely collected data

Journal:	*BMJ Open*
Manuscript ID	bmjopen-2022-066702.R1
Article Type:	Original research
Date Submitted by the Author:	09-Nov-2022
Complete List of Authors:	Rubbo, Bruna; University of Southampton, School of Health Sciences<br>Saville, Christina; University of Southampton, School of Health Sciences; National Institute for Health Research Applied Research Collaboration (Wessex), University of Southampton<br>Dall'Ora, Chiara; University of Southampton, School of Health Sciences; National Institute for Health Research Applied Research Collaboration (Wessex), University of Southampton<br>Turner, Lesley; University of Southampton, School of Health Sciences<br>Jones, Jeremy; University of Southampton, School of Health Sciences<br>Ball, Jane; University of Southampton, School of Health Sciences<br>Culliford, David; University of Southampton, Faculty of Medicine; National Institute for Health Research Applied Research Collaboration (Wessex), University of Southampton<br>Griffiths, Peter; University of Southampton, School of Health Sciences; National Institute for Health Research Applied Research Collaboration (Wessex), University of Southampton
**Primary Subject Heading**:	Health policy
Secondary Subject Heading:	Health services research
Keywords:	Organisation of health services < HEALTH SERVICES ADMINISTRATION & MANAGEMENT, Quality in health care < HEALTH SERVICES ADMINISTRATION & MANAGEMENT, Health policy < HEALTH SERVICES ADMINISTRATION & MANAGEMENT

I, the Submitting Author has the right to grant and does grant on behalf of all authors of the Work (as defined in the below author licence), an exclusive licence and/or a non-exclusive licence for contributions from authors who are: i) UK Crown employees; ii) where BMJ has agreed a CC-BY licence shall apply, and/or iii) in accordance with the terms applicable for US Federal Government officers or employees acting as part of their official duties; on a worldwide, perpetual, irrevocable, royalty-free basis to BMJ Publishing Group Ltd ("BMJ") its licensees and where the relevant Journal is co-owned by BMJ to the co-owners of the Journal, to publish the Work in this journal and any other BMJ products and to exploit all rights, as set out in our licence.

The Submitting Author accepts and understands that any supply made under these terms is made by BMJ to the Submitting Author unless you are acting as an employee on behalf of your employer or a postgraduate student of an affiliated institution which is paying any applicable article publishing charge ("APC") for Open Access articles. Where the Submitting Author wishes to make the Work available on an Open Access basis (and intends to pay the relevant APC), the terms of reuse of such Open Access shall be governed by a Creative Commons licence – details of these licences and which Creative Commons licence will apply to this Work are set out in our licence referred to above.

Other than as permitted in any relevant BMJ Author's Self Archiving Policies, I confirm this Work has not been accepted for publication elsewhere, is not being considered for publication elsewhere and does not duplicate material already published. I confirm all authors consent to publication of this Work and authorise the granting of this licence.

For peer review only

Staffing levels and hospital mortality in England: a national panel study using routinely collected data

Bruna Rubbo[1], Christina Saville[1,2], Chiara Dall'Ora[1,2], Lesley Turner[1], Jeremy Jones[1], Jane Ball[1], David Culliford[2], Peter Griffiths[1,2]*

[1]University of Southampton, School of Health Sciences, Southampton, UK

[2]National Institute for Health Research Applied Research Collaboration, Wessex

*Corresponding author

peter.griffiths@soton.ac.uk (PG)

Short title: Staffing levels and hospital mortality

For peer review only

Abstract

Objectives

Examine the association between multiple clinical staff levels and case-mix adjusted patient mortality in English hospitals. Most studies investigating the association between hospital staffing levels and mortality have focused on single professional groups, in particular nursing. However, single staff group studies might overestimate effects or neglect important contributions to patient safety from other staff groups.

Design

Retrospective observational study of routinely available data.

Setting and participants

138 National Health Service hospital trusts that provided general acute adult services in England between 2015 and 2019.

Outcome measure

Standardised mortality rates were derived from the Summary Hospital level Mortality Indicator dataset, with observed deaths as outcome in our models and expected deaths as offset. Staffing levels were calculated as the ratio of occupied beds per staff group. We developed negative binomial random-effects models with trust as random effects.

Results

Using a multilevel negative binomial random effects model, hospitals with higher levels of medical and allied healthcare professional (AHP) staff had significantly lower mortality rates (1.04, 95%CI 1.02 to 1.06, and 1.04, 95%CI 1.02 to 1.06, respectively), while those with higher support staff had higher mortality rates (0.85, 95%CI 0.79 to 0.91 for nurse support, and 1.00, 95%CI 0.99 to 1.00 for AHP support), after adjusting for multiple staff groups and hospital characteristics. Estimates of staffing levels on mortality were stronger between- than within-hospitals, which were not statistically significant in a within-between random effects model.

Conclusions

Considering multiple staff groups simultaneously when examining the association between hospital mortality and clinical staffing levels is crucial.

Article Summary

Strengths and limitations of the study

- We collected data from 138 hospitals over 4 years
- We included numerous clinical staffing groups simultaneously
- We did not have access to data at the ward level, so our associations were at the hospital level only.
- We used occupied beds-per-full time/contract staff, but this might not be a complete measure of staffing levels, as it does not take into account temporary staff or staff absence.

Keywords

Scheduling and staffing; Hospital Mortality; Workforce; Patient Safety; Delivery of Health Care

For peer review only

Introduction

Cost constraints and a quest for safer hospitals put pressure on health systems to find efficient staffing models to meet rising demand for care. Adequate nurse staffing levels in acute hospitals have important implications for positive outcomes for patients, including reducing patients' risk of death and costs.(1–5) Low registered nurse staffing levels are associated with failure to respond to patient abnormal changes in their physiological vital signs, which is in turn associated with failure to prevent death.(6) This link between registered nurses, failure to detect early deterioration and risk of death has meant the focus of much of the evidence and associated policy is on nurses. Nonetheless, the healthcare workforce is made up of many different professional groups, who must be mobilised to properly respond to or prevent deterioration and staffing levels of other staff groups are also likely to influence the quality and safety of care.

Studies focussing on the impact of clinical staff other than nurses on hospital mortality rates are relatively scarce. There are several studies on the effect of physician staffing levels, and they all indicate increased risk of patient death with lower medical staffing levels.(7–18) In-hospital patient care is delivered by multidisciplinary staff and no single staff group is solely responsible for patient outcomes. However, only a few studies considered multiple staff groups simultaneously, with some finding partial attenuation or complete absence of the effect of single staff levels on mortality when adjusted for other professionally qualified staff.(7,13,14,19) To our knowledge, no recent study has examined the potential effect of allied healthcare professionals (AHP) staffing levels on clinical outcomes, which include staff in the fields of podiatry and chiropody, dietetics, occupational therapy, orthoptics and optics, physiotherapy, radiography, art, music and drama therapy, and speech and language therapy. Only a few studies focused on in-hospital pharmacists.(19,20)

In the face of enduring staffing shortages in some professional groups it is important to understand the likely consequences on patient care and to identify priorities. A focus on single professional groups risks unintended consequences through neglect of other important contributions to patient safety and might lead to biased estimates. We therefore aimed to examine the associations between staffing levels of multiple staff groups, and case-mix adjusted patient mortality in hospitals.

Methods

Study design

This was a retrospective observational study using routinely available data on clinical healthcare staffing and hospital mortality.

Study setting

We included all 138 National Health Service (NHS) hospital trusts providing general acute adult inpatient services in England between April 2015 and March 2019. Hospital trusts are defined as organisational units within the NHS that service a defined geographical area or that provide a specialised function. One trust can therefore encompass several hospitals.

For peer review only - http://bmjopen.bmj.com/site/about/guidelines.xhtml

Data sources and linkage

We linked four data sources that provide trust-level datasets: a) NHS workforce, which contains detailed information on trust staffing; b) bed availability and occupancy, which contains data on trust-level available and occupied beds; c) Estates Returns Information Collection (ERIC), which contains data on trust organisation and structure; and d) Summary Hospital-level Mortality Indicator (SHMI), which contains data on observed and expected deaths (Table S1). These datasets are openly available and accessible on the NHS Digital platform, along with the data dictionary.(21,22) We linked the datasets using the unique hospital trust ID (*i.e.* “Org code”).

The SHMI includes all deaths that occur in hospital or within 30-days of discharge among patients admitted to non-specialist acute trusts. SHMI data are derived from the Hospital Episode Statistics (HES) at the level of provider spells (*i.e.* total continuous stay of a patient using a hospital bed at an NHS organisation under the care of one or more consultants, or nursing episode or midwife episode), and the HES-Office of National Statistics (ONS) linked mortality data (Table S1).(23) The latter captures deaths that occur outside of hospital. Data used to calculate the SHMI are submitted by each hospital trust.

SHMI expected deaths is calculated based on individual patient characteristics that can affect the risk of mortality, including the patient’s condition for hospitalisation, underlying conditions (Charlson Comorbidity Index),(24) age, gender, method of admission to hospital, and year of discharge. Logistic regression models estimate the risk for each provider spell, with binary variable as outcome (*i.e.* died or survived). The SHMI model performs well with early validation accounting for 81% of between-hospital variations.(25) The models are based on the preceding three years’ data, with last year data used to calculate the SHMI (26). As SHMI data are published monthly, we used the dataset that contains mortality data from April to March the following year to report on annual hospital mortality level for each hospital trust included in this study. Additional information, including dataset and reports, can be found at the NHS digital website (https://digital.nhs.uk/data-and-information/publications/ci-hub/summary-hospital-level-mortality-indicator-shmi).

We obtained hospital staffing data by linking the medical and dental workforce dataset (physicians) with the non-medical workforce dataset (all other healthcare workers, including nurses, nurse assistants, physiotherapists, pharmacists, among others). Bed occupancy data are published quarterly as averages, with no estimate of monthly variation. For wards open overnight, occupied bed is defined as when it is occupied at midnight on the day in question; for day-only wards, occupied bed is a bed where at least one day case has taken place during the day. Trust teaching status was derived from the ERIC dataset, which contains variables on trust profile.

Staffing variables are published monthly. We used full-time equivalent (FTE) values to calculate the annual average available staff at each hospital trust from April to March the following year, to align with the SHMI data. FTE data are based on the proportion of time each staff are expected to work in a week, which would correspond to an FTE of 1 (*e.g.* 48 hours for doctors, 37.5h for nurses and AHP). Overtime and out-of-hour work are not recorded in these datasets.

Study outcome

The main study outcome was annual standardised all-cause mortality rate derived from observed and expected deaths in the SHMI dataset.(27) This is calculated and reported for all trusts providing acute adult services in NHS England.(25) Specialist trusts that do not

For peer review only - http://bmjopen.bmj.com/site/about/guidelines.xhtml

provide general acute care do not report SHMI and, therefore, were not included in the study.

Staffing variables

Clinical staff are classified according to occupation codes. We grouped staffing variables by frequency and occupation as listed by NHS England, merging groups that had mean below 3 with others of a similar occupation in order to create 8 groups (*i.e.* medical, surgical, other medical specialties, nurses, support to nurses, AHP, support to AHP, and scientific, therapeutic, and technical (ST&T) staff). General medicine specialist doctors were grouped with clinical oncology doctors to form the general medicine group, surgeons were grouped with obstetricians and gynaecologists to form the surgical group, and all other medical specialties were grouped together to form the other medical group (*i.e.* radiologists, emergency medicine, anaesthetists, and pathologists). We excluded psychiatrists and paediatricians. The nurses' group was composed of nurses in adult services only; we did not include nurses from paediatric services. The nurse support group included support to adult and general nurses, and nurses in training. Scientific, therapeutic, and technical (ST&T) staff group was composed of multi-therapists, applied psychologist, psychological therapist, pharmacists, dentists, operating theatre staff, social services, and other ST&T staff.

We used the number of general acute occupied beds to calculate the bed-per-staff ratio. Beds assigned to maternity, mental illness, and learning disabilities services were excluded; however, general acute beds represented 96.2% of the total beds available across all hospital trusts included in the study. We calculated the average number of occupied beds overnight or day-only per trust per year, and then divided occupied beds per each staff group to obtain the average bed-per-staff levels for each hospital trust per year.

Hospital-level covariates

Teaching affiliation was coded as yes or no according to the trust type recorded in the ERIC dataset. Trust size was calculated based on the number of available general acute beds in each trust.

Statistical analysis

We initially explored the data using descriptive statistics. Continuous variables were described as mean and standard deviation (SD) or median and IQR, depending on the distribution of each variable. Categorical variables were described as frequencies and proportions. Annual mortality rates were calculated by dividing the number of observed deaths by the number of patient spells, across all hospital trusts each year. To explore the relationship between staffing levels and hospital mortality, statistical modelling was conducted at the hospital trust-level using multiple regression models on four years of data, with standardised mortality rates regressed onto hospital level staffing levels (expressed as the number of occupied beds-per-staff). We included expected deaths as an offset, as the number of expected deaths is the number of times the event could have happened (*i.e.* the exposure variable).

Multilevel models were adjusted for trust size and affiliation as a teaching hospital, with trust included as a random effect to adjust for clustering. We considered a range of potential models, with model selection based on theoretical reasoning and model fit (minimising the Akaike information criterion (AIC), the Bayesian information criterion (BIC) and the likelihood ratios (see Table S2 for alternative frameworks considered)). We report exponentiated coefficients (rate ratios) with 95% confidence intervals (CI) for all estimates obtained from the models.

For peer review only - http://bmjopen.bmj.com/site/about/guidelines.xhtml

The best performing model (referred to as main model) was a negative binomial random effects model that included all 8 clinical staff groups and hospital characteristics (*i.e.* trust size and teaching status). To explore variations between and within hospital trusts, we also constructed a within-between random effects model (WBRE), with trusts as random effect, and staffing and hospital characteristics as fixed effects. These models can differentiate between effects arising from staffing differences between hospital trusts and those that are associated with annual changes of staffing levels within each hospital by group-mean centring each trust.(28,29)

We assessed potential collinearity between co-variates using correlation plots and Spearman's correlation coefficient, and the overall multicollinearity for all predictors in the models using generalised variance inflation factors (GVIF). All models had a GVIF<10. As a sensitivity analysis, we reran the models after excluding any variables that had a GVIF above 5.(30)

Observations with missing data were removed from the analyses; however, hospital trusts that reported at least one year of complete data were included.

We performed data linkage, cleaning, coding, and analyses in R statistical software, version 4.0.2 (R Core Team, R Foundation for Statistical Computing, Vienna, Austria), using the lme4 and glmer packages (v1.1-27.1, Bates et al, 2015), and the plm package (v2.4-3, Croissant et al, 2021).(31) The full dataset is available.(32)

Patient and public involvement

There was no patient and public involvement in this study.

Results

Descriptive statistics

There were 540 observations in the linked dataset for the 4-year study period, of which 519 (96%) contained data on all variables and were therefore included in our analyses. There were 2 hospital trusts that did not report on medical staffing in 2016, with 6 trusts not reporting these variables from 2017 onwards. For other clinical staff, only 1 trust did not report staff numbers in 2016 and 2017, and two trusts in 2018, with no missing data in 2019. One trust did not report the number of occupied general acute beds in 2018. The number of hospital trusts that reported on complete data varied, with 137 included in 2016 and 2017, 135 in 2018, and 130 in 2019.

The median number of acute general occupied beds per day was 674.0 (IQR 486.1 to 911.6). The mean annual observed deaths ± SD was 2229 ± 992 (range 525 to 7468), while the expected deaths were 2228 ± 987.2 (range 665 to 7591). Annual mortality rates across all hospital trusts were 3.44% for 2016, 3.46% for 2017, 3.23% for 2018, and 3.31% for 2019. There was a median of 744.2 available general acute beds per trust (interquartile range (IQR) 535.7 to 1009.4, range 143 to 2704). Hospital trusts in the upper tertile (*i.e.* the upper third of trusts, ranked by size) were classified as large, while those in the lower tertile were deemed small trusts, with the remaining classified as medium-sized hospital trusts. Forty-one (29.7%) hospital trusts were classified as teaching trusts.

Table 1 shows the distribution of staffing variables by their median, IQR, mean, and SD across all hospital trusts. There was considerable variation in staffing levels, with nurse staff levels showing least relative variation (SD 18.0% of the mean) and AHP support most (SD 44.4% of the mean). By contrast, variations within trusts as a percentage of the mean were

For peer review only - http://bmjopen.bmj.com/site/about/guidelines.xhtml

relatively low, with the within trust variation ranging from 4.9% for nurse staffing to 10.9% for AHP support.

Table 1. Frequency of occupied bed-per-staff variables across 138 hospital trusts

Staffing level variables	Median (IQR)	Mean (SD)	SD as % of mean	Mean within-trust SD	Within-trust SD as % of mean
Bed per medical	3.66 (3.05 to 4.30)	3.70 (0.96)	25.9%	0.28	7.6%
Bed per surgical	3.79 (3.10 to 4.28)	3.73 (0.81)	21.7%	0.22	5.9%
Bed per other medical specialties	3.96 (3.26 to 4.69)	3.94 (1.01)	25.6%	0.27	6.9%
Bed per nurse adult service	0.61 (0.54 to 0.68)	0.61 (0.11)	18.0%	0.03	4.9%
Bed per nurse support	0.93 (0.80 to 1.08)	0.93 (0.21)	22.6%	0.07	7.5%
Bed per AHP	2.25 (1.88 to 2.74)	2.36 (0.89)	37.7%	0.18	7.6%
Bed per AHP support	10.03 (7.91 to 13.01)	11.07 (4.91)	44.4%	1.21	10.9%
Bed per ST&T	2.24 (1.80 to 2.82)	2.31 (0.75)	32.5%	0.16	6.9%

Q1: lower quartile, Q3: upper quartile, SD: standard deviation, AHP: allied healthcare professional, ST&T: scientific, therapeutic, and technical

Nurse staffing levels were strongly correlated with staffing levels in all medical staff groups (rho>0.71). Nurse support staff levels were correlated to RN (rho=0.30), as were AHP and AHP support (rho=0.47) (Figure S1).

Staffing levels and hospital mortality

Table 2 shows results for the negative binomial random effect models, adjusted for hospital characteristics. In the single staff group models, hospital trusts that had lower staffing levels (*i.e.* more occupied beds per FTE staff) were associated with higher (standardised) mortality rates for all professionally qualified staff groups. The opposite effect was observed for nursing support and AHP support staff, where hospital trusts with lower staffing levels (*i.e.* more occupied beds per FTE staff) had lower mortality.

For peer review only - http://bmjopen.bmj.com/site/about/guidelines.xhtml

Table 2. Exponentiated estimates for hospital mortality from the multilevel negative binomial random effect models, adjusted for hospital characteristics (*n* = 138)

Predictors	Estimates for single staff models	95% CI	Estimates for multiple staff model	95% CI
Bed per medical	1.05***	1.04 to 1.07	1.04***	1.02 to 1.06
Bed per surgical	1.04***	1.02 to 1.06	0.98	0.96 to 1.01
Bed per other medical specialties	1.05***	1.03 to 1.06	1.03*	1.00 to 1.06
Bed per nurse adult service	1.33***	1.15 to 1.54	1.07	0.88 to 1.31
Bed per nurse support	0.92*	0.86 to 0.98	0.85***	0.79 to 0.91
Bed per AHP	1.02*	1.00 to 1.04	1.04***	1.02 to 1.06
Bed per AHP support	0.99*	0.99 to 0.99	1.00**	0.99 to 1.00
Bed per ST&T	1.02**	1.00 to 1.04	0.99	0.98 to 1.05
Teaching (ref not teaching)	0.96*	0.92 to 0.99	1.01	0.98 to 1.05
Medium trust (ref small trust)	1.01	0.97 to 1.05	1.01	0.98 to 1.04
Large trust (ref small trust)	1.00	0.96 to 1.04	1.01	0.98 to 1.05

CI: confidence interval, AHP: allied healthcare professional, ST&T: scientific, therapeutic, and technical, * $p<0.05$, ** $p<0.01$, *** $p<0.001$. Estimates are a ratio of observed to expected deaths.

In the main model including all staff groups, associations between medical (1.04, 95%CI 1.02 to 1.06), other medical specialties (1.03, 95%CI 1.00 to 1.06), AHP (1.04, 95%CI 1.02 to 1.06), nurse support (0.85, 95%CI 0.79 to 0.91), and AHP support (1.00, 95%CI 0.99 to 1.00) FTE staffing levels and hospital mortality remained statistically significant, with no change in direction of the effect compared to the single staff models (Table 2). Higher mortality rates were observed in hospital trusts with lower levels of medical and AHP staff. In contrast, hospital trusts with lower support staff levels per occupied bed (*i.e.* nursing support and AHP support) had lower mortality rates. The association with RN staffing was attenuated and no longer statistically significant (1.07, 95%CI 0.88 to 1.31), although the observed effect remained relatively large compared to other associations (Table S3). Similarly, the association between hospital mortality and ST&T staff, observed in the single staff groups models, were no longer statistically significant in the model adjusted for all staff groups.

Although we noted correlation between staffing variables, our GVIF for all variables was <10. The GVIF for beds per other medical specialties was 6.1 (Table S3), and therefore over our threshold of 5, so we ran the negative binomial random effects model omitting this variable as a sensitivity analysis and found similar results (not shown; available from authors upon request).

Within-between hospital trust variability

Between-hospital trust effects from the WBRE model were largely the same as those obtained from the main model (*i.e.* between only). For example, the between-estimate from WBRE for the FTE medical group was 1.04 (95%CI 1.02 to 1.07), similar to the estimates obtained from the main model (1.04, 95%CI 1.02 to 1.06). However, even for staff groups where between-hospital trusts effects were significant, the within-hospital effects were small and not statistically significant. For example, the within-hospital estimate from the WBRE model for the medical group was 0.99 (95%CI 0.97 to 1.01) (Table 3). Despite potentially providing additional information by decomposing the variability into between- and within-estimates, the WBRE model did not improve fit compared to the main model (Table S2).

For peer review only - http://bmjopen.bmj.com/site/about/guidelines.xhtml

Table 3. Exponentiated estimates for hospital mortality from the multilevel within-between negative binomial random effect models, adjusted for hospital characteristics (*n* = 138).

Predictors	Observed deaths Incidence Rate Ratios	95% confidence intervals	p-values
**Bed per medical**			
Within-hospital	0.98	0.97 – 1.00	0.129
Between-hospital	1.04	1.02 – 1.07	**<0.001**
**Bed per surgical**			
Within-hospital	1.03	0.99 – 1.06	0.106
Between-hospital	0.98	0.95 – 1.01	0.222
**Bed per other medical specialties**			
Within-hospital	1.02	0.99 – 1.04	0.21
Between-hospital	1.02	0.99 – 1.06	0.124
**Bed per nurse**			
Within-hospital	0.88	0.75 – 1.04	0.148
Between-hospital	1.1	0.88 – 1.37	0.393
**Bed per nurse support**			
Within-hospital	0.99	0.91 – 1.07	0.789
Between-hospital	0.84	0.77 – 0.90	**<0.001**
**Bed per AHP**			
Within-hospital	0.99	0.96 – 1.01	0.296
Between-hospital	1.04	1.02 – 1.07	**<0.001**
**Bed per AHP support**			
Within-hospital	1	1.00 – 1.00	0.352
Between-hospital	1	0.99 – 1.00	**0.017**
**Bed per ST&T**			
Within-hospital	1.01	0.99 – 1.04	0.329
Between-hospital	0.99	0.96 – 1.01	0.266
**Teaching hospital**			
Yes	1.02	0.98 – 1.05	0.323
**Trust size (reference: small)**			
Medium	1	0.98 – 1.03	0.706
Large	1.01	0.98 – 1.04	0.381

Discussion

After adjustment for hospital characteristics and including multiple clinical staff groups in our models, we found that higher medical and AHP staffing levels were associated with lower mortality rates in acute hospital trusts in England during the period of 2015 to 2019. In contrast, hospitals with higher support staff per occupied bed had higher mortality rates.

Our study highlights the importance of simultaneously adjusting for multiple clinical staff groups when investigating associations between staffing levels and mortality at the hospital-level. In the single staff group models, significant effects on mortality rates were seen for all staffing groups. However, when adjusted for multiple groups, effects for medical, other medical specialties, AHP, nurse support, and AHP support staffing levels remained statistically significant, but levels of surgical doctors, RN, and ST&T staff were no longer significant.

For peer review only - http://bmjopen.bmj.com/site/about/guidelines.xhtml

Our findings are in line with a previous study by Jarman *et al*,(7) who found that the association between RN staffing and mortality was no longer significant when adjusting for doctors-per-bed, with higher levels of physician staffing significantly reducing hospital standardised mortality ratios.

Nonetheless, other studies have shown a reduction in hospital mortality when both higher numbers of doctors and nurses per bed were observed.(11,13–15,33–36) The fact that the association between nurse staffing and mortality was not significant in the multivariable models should not be taken to indicate an absence of effect to nursing. Previous studies have reported that much of the variation in nurse staffing occurs between wards within hospitals and within wards over time (1,37) and Keogh noted that hospital nurse staffing levels often bore little relationship to staffing available to be deployed on wards in NHS hospitals,(38) therefore hospital nurse staffing per bed may be a poor indicator of the staffing levels experienced by inpatients. Griffiths *et al* (13) found that when medical staffing levels were accounted for, significant nurse staffing effects were only observed in models using ward-based staffing ratios as opposed to hospital-level staff-per-bed ratios. Longitudinal patient level studies of exposure to variation in nurse staffing confirm that there is an adverse effect of low nurse staffing when measured at this level.(2,39,40)

To our knowledge, this was the first study to include AHP and AHP support staff levels in analysis. Higher levels of AHP staff had a protective effect at the hospital trust level while more AHP support staff were detrimental to patient mortality, a finding that mirrors those obtained for nursing support staff. While causality cannot be assumed for any of these results, there is a potential that previous findings that have emphasised a link between nurse staffing and patient safety could divert attention from the role that other staff groups may play in delivering quality and preventing avoidable deaths.

In our study, high levels of support staff were associated with higher mortality rates. This finding appears to be paradoxical: increasing the number of some staff groups might lead to increased mortality, when holding all other staffing variables constant. This cannot be fully explored with the given data. One potential reason might be that raising levels of one staff group might affect the distribution of other staff groups to different departments and wards and this could negatively affect mortality. Another possible reason could be that increasing the levels of one staff group might alter the duties of other staff groups – for example, adding more nurse assistants while holding the number of registered nurses constant might increase the workload for the registered nurses as they are now responsible for the supervision of a larger number of nurse assistants, or create an implicit demand for delegation of work that is not suited to the assistants, which could negatively impact mortality.(39) Unfortunately, due to the fact that we do not have granular in-hospital data on deployment of staff and staff responsibility, we are unable to further investigate this, although this mechanism is consistent with evidence on skill mix in the nursing team which shows a tipping point where adding assistants has a positive effect when the number of assistant is low, but a negative one where the number is high.(39)

We found that variations between hospital trusts in the 4-year study period were higher in magnitude than within-hospital trusts estimates, which were not statistically significant for any of the clinical staff groups at the hospital level. Structural recruitment and retention difficulties, financial constraints, and other non-staffing resources available at each hospital could be main contributors to inter-hospital trust variations. There was relatively little year on year variation in staffing between trusts. Reduced variability of staffing levels within hospitals might be due to the limited number of funded posts in each organisation and could reflect historic staffing levels. Bjerregaard *et al*,(36) using a WBRE model, found staffing levels estimates were significant between wards, while within-ward estimates had no significant effect on hospital mortality. Between hospital effects may reflect structural differences in staffing resource between hospitals while the limited within hospital variation we observed could be largely endogenous, reflecting variation in patient populations and risk.

For peer review only - http://bmjopen.bmj.com/site/about/guidelines.xhtml

Strengths and limitations

This was a national study using 4 years of routinely available data. The inclusion of numerous clinical staffing groups simultaneously in the models allowed us to estimate the effect of each staffing level on hospital mortality while considering hospital trust characteristics.

We used a variety of models and selected the one that performed best on our data. Most studies investigating clinical staffing levels and in-hospital mortality used fixed effects models, which only evaluate within-hospital effects. Such models might not appropriately assess the effect of different staffing levels based on variation between hospitals in hospital-level observational studies.

However, our study was limited by the relatively small sample size, as observations were clustered on 138 hospital trusts. We therefore had to group some of the medical specialties and were unable to unpack relatively heterogenous groups such as AHP. Furthermore, we were unable to explore the effects of grade and experience for medical (*e.g.* junior doctors, level of consultants) and RN (*e.g.* senior nurses) staff.

In our study, beds that were occupied by multiple patients in a single day were counted as one single occupied bed. Studies have shown that workload (*e.g.* number of admissions, discharges, additional tasks) in high volume hospitals can lead to higher mortality rates, even when levels of staffing are similar to those observed in low volume hospitals. Occupied beds-per-staff might not be a good measure of staffing levels, as these do not account for temporary staff or staff absence due to sickness, maternity leave, or long-term leave. The extent to which staff are deployed to deliver services such as ambulatory care or other services not delivered to inpatient beds is likely to vary.(41) Additionally, we could not include overtime and out-of-hour work, as these are not captured in the datasets used for this study.

Future studies should aim to capture different staffing levels responsible for delivering patient care measured at the patient or ward-level, rather than at the hospital-level.

Although SHMI provides good control for patient risk and we controlled for hospital factors and multiple staff groups, the cross-sectional nature of our analysis means that findings need to be interpreted as demonstrating association but not direct evidence of causation.

Conclusion

In conclusion, our study highlights the importance of simultaneously considering multiple staff groups when investigating the effect of clinical staffing levels and mortality at the hospital-level. We showed that the number of AHP and AHP support per occupied beds have a significant impact on patient mortality, yet these groups have largely not been included in previous workforce studies, of which the majority focused exclusively on nursing staff. We also found that hospitals with higher levels of medical staffing had lower mortality rates, while higher levels of support workers were associated with higher hospital mortality.

Data Statement

The dataset is available from the ePrints repository, DOI: https://doi.org/10.5258/SOTON/D2055

Authors' contributions

BR, CS, CDO, LT, JJ, JB, DC, and PG made substantial contributions to the conception or design of the work. BR, JJ, PG acquired the data, BR analysed the data, CS and DC independently checked the data analyses, BR, CS, CDO, LT, JJ, JB, DC, and PG discussed

For peer review only - http://bmjopen.bmj.com/site/about/guidelines.xhtml

the interpretation of data for the work; BR drafted the work, and BR, CS, CDO, LT, JJ, JB, DC, and PG revised it critically for important intellectual content and gave final approval of the version to be published; PG is accountable for all aspects of the work in ensuring that questions related to the accuracy or integrity of any part of the work are appropriately investigated and resolved.

Conflicts of interest

All authors have completed the ICMJE uniform disclosure form at http://www.icmje.org/disclosure-of-interest/ and declare: all authors had financial support from the NIHR Health Services and Delivery Research for the submitted work; no financial relationships with any organisations that might have an interest in the submitted work in the previous three years; no other relationships or activities that could appear to have influenced the submitted work."

Funding

This study/project is funded by the NIHR [Health and Social Care Delivery Research (NIHR128056)]. The views expressed are those of the author(s) and not necessarily those of the NIHR or the Department of Health and Social Care.

Ethics Approval

We did not need ethics approval as this project relied on publicly available routinely collected data.

For peer review only

For peer review only - http://bmjopen.bmj.com/site/about/guidelines.xhtml

References

1 Griffiths P, Ball J, Bloor K, et al. Nurse staffing levels, missed vital signs and mortality in hospitals: retrospective longitudinal observational study. *Health Services and Delivery Research* 2018;6:38

2. Dall'Ora C, Saville C, Rubbo B, et al. Nurse staffing levels and patient outcomes: A systematic review of longitudinal studies. *Int J Nurs Stud*. 2022;134:104311.

3. Dietermann K, Winter V, Schneider U, et al. The impact of nurse staffing levels on nursing-sensitive patient outcomes: a multilevel regression approach. Eur J Health Econ. 2021;22(5):833–46.

4. Kane RL, Shamliyan TA, Mueller C, et al. The association of registered nurse staffing levels and patient outcomes: systematic review and meta-analysis. Med Care. 2007;45(12):1195–204.

5. Winter V, Dietermann K, Schneider U, et al Nurse staffing and patient-perceived quality of nursing care: a cross-sectional analysis of survey and administrative data in German hospitals. *BMJ Open*. 2021;11(11):e051133.

6. Hogan H, Healey F, Neale G, et al. Preventable deaths due to problems in care in English acute hospitals: a retrospective case record review study. *BMJ Qual Saf*. 2012;21(9):737–45.

7. Jarman B, Gault S, Alves B, et al. Explaining differences in English hospital death rates using routinely collected data. *BMJ*. 1999;318(7197):1515–20.

8. Pronovost PJ, Angus DC, Dorman T, et al. Physician staffing patterns and clinical outcomes in critically ill patients: a systematic review. *JAMA*. 2002;288(17):2151–62.

9. Lin HC, Lee HC. Caseload volume-outcome relation for pulmonary embolism treatment: association between physician and hospital caseload volume and 30-day mortality. *J Thromb Haemost*. 2008;6(10):1707–12.

10. Ong M, Bostrom A, Vidyarthi A, et al. House staff team workload and organization effects on patient outcomes in an academic general internal medicine inpatient service. *Arch Intern Med*. 2007;167(1):47–52.

11. Ricciardi R, Nelson J, Roberts PL, et al. Is the presence of medical trainees associated with increased mortality with weekend admission? *BMC Med Educ*. 2014;14:4.

12. Coit MH, Katz JT, McMahon GT. The effect of workload reduction on the quality of residents' discharge summaries. *J Gen Intern Med*. 2011;26(1):28–32.

13. Griffiths P, Ball J, Murrells T, et al. Registered nurse, healthcare support worker, medical staffing levels and mortality in English hospital trusts: a cross-sectional study. *BMJ Open*. 2016;6(2):e008751.

14. Bond CA, Raehl CL, Pitterle ME, et al. Health care professional staffing, hospital characteristics, and hospital mortality rates. *Pharmacotherapy.* 1999;19(2):130–8.

15. West E, Barron DN, Harrison D, et al. Nurse staffing, medical staffing and mortality in Intensive Care: An observational study. *Int J Nurs Stud.* 2014;51(5):781–94.

For peer review only - http://bmjopen.bmj.com/site/about/guidelines.xhtml

16. Dara SI, Afessa B. Intensivist-to-bed ratio: association with outcomes in the medical ICU. *Chest.* 2005;128(2):567–72.

17. Neuraz A, Guérin C, Payet C, et al. Patient Mortality Is Associated With Staff Resources and Workload in the ICU: A Multicenter Observational Study. *Crit Care Med*. 2015;43(8):1587–94.

18. Gershengorn HB, Harrison DA, Garland A, et al. Association of Intensive Care Unit Patient-to-Intensivist Ratios With Hospital Mortality. *JAMA Intern Med*. 2017;177(3):388–96.

19. Lee H, Ryu K, Sohn Y, et al. Impact on Patient Outcomes of Pharmacist Participation in Multidisciplinary Critical Care Teams: A Systematic Review and Meta-Analysis. *Crit Care Med*. 2019;47(9):1243–50.

20. Bond CA, Raehl CL, Franke T. Interrelationships among mortality rates, drug costs, total cost of care, and length of stay in United States hospitals: summary and recommendations for clinical pharmacy services and staffing. *Pharmacotherapy*. 2001;21(2):129–41.

21. [dataset] NHS Digital. National Workforce Data Set (NWD) and NHS occupational codes. [accessed 2022 Nov 02]. Available from: https://digital.nhs.uk/data-and-information/data-collections-and-data-sets/data-sets/national-workforce-data-set-nwd-and-nhs-occupation-codes#top

22. NHS Digital. NHS Data Model and Dictionary. NHS England; [accessed 2022 Nov 02]. Available from: https://datadictionary.nhs.uk/index.html

23. Aylin P, Williams S, Bottle A, Jarman B. Counting hospital activity: spells or episodes? *BMJ*. 2004;329(7476):1207.

24. Charlson ME, Pompei P, Ales KL, et al. A new method of classifying prognostic comorbidity in longitudinal studies: development and validation. *J Chronic Dis.* 1987;40(5):373–83.

25. Campbell MJ, Jacques RM, Fotheringham J, et al. Developing a summary hospital mortality index: retrospective analysis in English hospitals over five years. *BMJ.* 2012;344:e1001.

26. Campbell MJ, Jacques RM, Fotheringham J, et al. An evaluation of the Summary Hospital Mortality Index. University of Sheffield; 2011.

27. NHS Digital. The processing cycle and HES data quality NHS Digital: NHS England. [accessed 2022 Feb 11]. Available from: https://digital.nhs.uk/data-and-information/data-tools-and-services/data-services/hospital-episode-statistics/the-processing-cycle-and-hes-data-quality

28. Bell A, Fairbrother M, Jones K. Fixed and random effects models: making an informed choice. *Qual Quant.* 2019;53(2):1051–74.

29. Schunck R. Within and between Estimates in Random-Effects Models: Advantages and Drawbacks of Correlated Random Effects and Hybrid Models. *Stata J.* 2013;13(1):65–76.

For peer review only - http://bmjopen.bmj.com/site/about/guidelines.xhtml

30. O'brien RM. A Caution Regarding Rules of Thumb for Variance Inflation Factors. *Qual Quant*. 2007;41(5):673–90.

31. R Core Team (2018). R: A language and environment for statistical computing. R Foundation for Statistical Computing, Vienna, Austria. [accessed 2022 Nov 08]. Available online at https://www.R-project.org/.

32. [dataset] Rubbo B, Griffiths P, Saville C, et al. NHS England staffing levels and mortality dataset. University of Southampton; 2021. Available at DOI: 10.5258/SOTON/D2055

33. Griffiths P, Jones S, Bottle A. Is "failure to rescue" derived from administrative data in England a nurse sensitive patient safety indicator for surgical care? Observational study. *Int J Nurs Stud*. 2013;50(2):292–300.

34. Ozdemir BA, Sinha S, Karthikesalingam A, et al. Mortality of emergency general surgical patients and associations with hospital structures and processes. *Br J Anaesth*. 2016;116(1):54–62.

35. Chung W, Sohn M. The Impact of Nurse Staffing on In-Hospital Mortality of Stroke Patients in Korea. *J Cardiovasc Nurs*. 2018;33(1):47–54.

36. Bjerregaard U, Hølge-Hazelton B, Rud Kristensen S, et al. Nurse Staffing and Patient Outcomes: Analyzing Within- and Between-Variation. *DaCHE Discussion Papers.* 2020

37. Van den Heede K, Sermeus W, Diya L, et al. Nurse staffing and patient outcomes in Belgian acute hospitals: cross-sectional analysis of administrative data. *Int J Nurs Stud.* 2009;46(7):928–39.

38. Keogh B. Review into the Quality of Care and Treatment Provided by 14 Hospital Trusts in England: Overview Report. London, UK: National Health Service; 2013 [accessed 2022 Nov 2]. Available from: https://psnet.ahrq.gov/issue/review-quality-care-and-treatment-provided-14-hospital-trusts-england-overview-report

39. Griffiths P, Maruotti A, Recio Saucedo A, et al. Nurse staffing, nursing assistants and hospital mortality: retrospective longitudinal cohort study. *BMJ Qual Saf*. 2019 Aug;28(8):609–17.

40. Needleman J, Buerhaus P, Pankratz VS, et al. Nurse staffing and inpatient hospital mortality. *N Engl J Med.* 2011;364(11):1037–45.

41. Ball J. Nurse staffing, care left undone and patient mortality in acute hospitals. Doctoral thesis. Inst för lärande, informatik, management och etik / Dept of Learning, Informatics, Management and Ethics; 2017 [accessed 2022 Nov 2]. Available from: http://openarchive.ki.se/xmlui/handle/10616/45563

Supporting information captions

Table S1. NHS digital data sources

Table S2. Comparison of goodness-of-fit for model selection

Table S3. Multicollinearity test in the main model (*i.e.* negative binomial random effect)

Figure S1. Spearman correlation between staffing level variables included in the study.

For peer review only

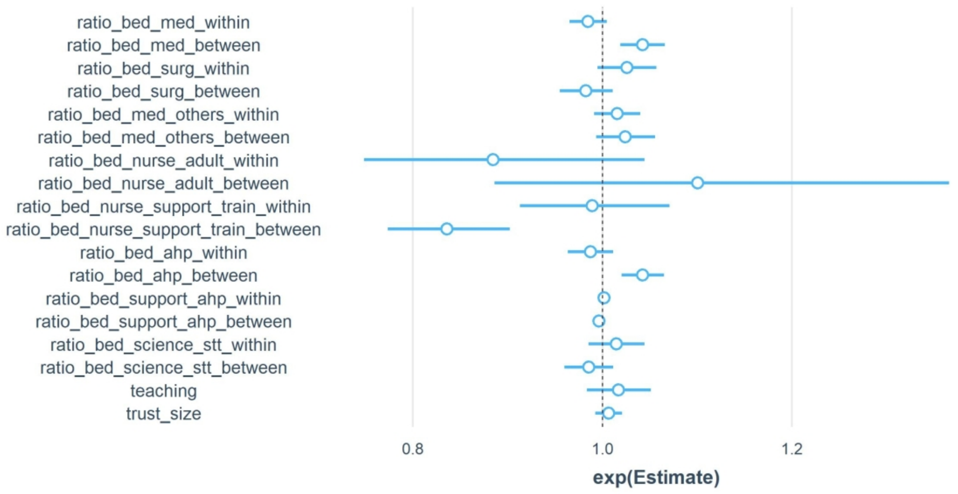

Hospital mortality exponentiated estimates obtained from the within-between random effect model (WBRE).

199x99mm (300 x 300 DPI)

Supporting information

Tables

Table S1. NHS digital data sources

Data source	Datasets	Main variables available
NHS workforce data	Medical dataset	Professionally qualified clinical staff
	Non-medical dataset	Professionally qualified clinical staff
		Support to clinical staff
Bed occupancy data	Overnight beds dataset	Available beds
		Occupied beds
		% occupied beds
	Day only beds datasets	Available beds
		Occupied beds
		% occupied beds
ERIC data		Trust profile (e.g. type)
		Strategies and policies
		Finance
		Safety
		Fire and safety
		Business transport
		Medical records
		Key worker accommodation
		Facilities management services
		Areas
		Function and space
		Quality of buildings
		Combined heat and power energy
		Energy
		Water services
		Waste
SHMI data		SHMI value
		SHMI banding
		Number of spells*
		Observed deaths
		Expected deaths

For peer review only

NHS: National Health Service, ERIC: Estates Returns Information Collection, SHMI: Summary Hospital level Mortality Indicator, * spells: total continuous stay of a patient using a hospital bed at an NHS organisation under the care of one or more consultants, or nursing episode or midwife episode.

Table S2. Comparison of goodness-of-fit for model selection

Model	Correlations for trusts	Model degrees of freedom	AIC	BIC	Likelihood-ratio
**OLS**	No	17	8183.5	8255.8	-4074.7
**Poisson**	No	16	10567.6	10635.7	-5267.8
**Negative binomial**	No	17	6692.6	6764.9	-3329.3
**GLS**	Yes	26	7517.0	7627.6	-3732.5
**GLS exchangeable**	Yes	18	7867.5	7944.0	-3915.7
**GLS Toeplitz (2)**	Yes	19	7823.9	7904.7	-3892.9
**GLS AR**	Yes	18	7822.7	7899.2	-3893.3
**Poisson RE**	Yes	14	1997.8	2057.3	-984.9
**Negative binomial RE**	Yes	14	250.3	309.9	-111.2
**Poisson FE**	Yes (dummy)	146	6477.4	7098.2	-3092.7
**Negative binomial FE**	Yes (dummy)	147	6180.5	6805.6	-2943.3
**Negative binomial WBRE**	Yes	22	6418.3	6511.8	-3187.1

AIC: Akaike information criterion, BIC: Bayesian information criterion, OLS: ordinary least squares, GLS: generalised least squares, AR: autoregressive, RE: random effects, FE: fixed effects, WBRE: within-between random effects, (2) tridiagonal 2-Toeplitz matrix

Table S3. Multicollinearity test in the negative binomial random effects model

Variable	GVIF
Beds per medical	3.1
Beds per surgical	3.6
Beds per other medical	6.1
Beds per nurse	3.7
Beds per nurse support	1.7
Beds per AHP	2.0
Beds per AHP support	1.4

For peer review only

Beds per ST&T	2.6
Teaching status	1.8
Trust size	1.1*

GVIF: generalised variance inflation factor; AHP: allied health professionals; ST&T: scientific, therapeutic, and technical; *reported on GVIF^(1/(2*degrees of freedom)) instead of GVIF as variable has 2 degrees of freedom

Figure S1. Spearman correlation between staffing level variables included in the study.

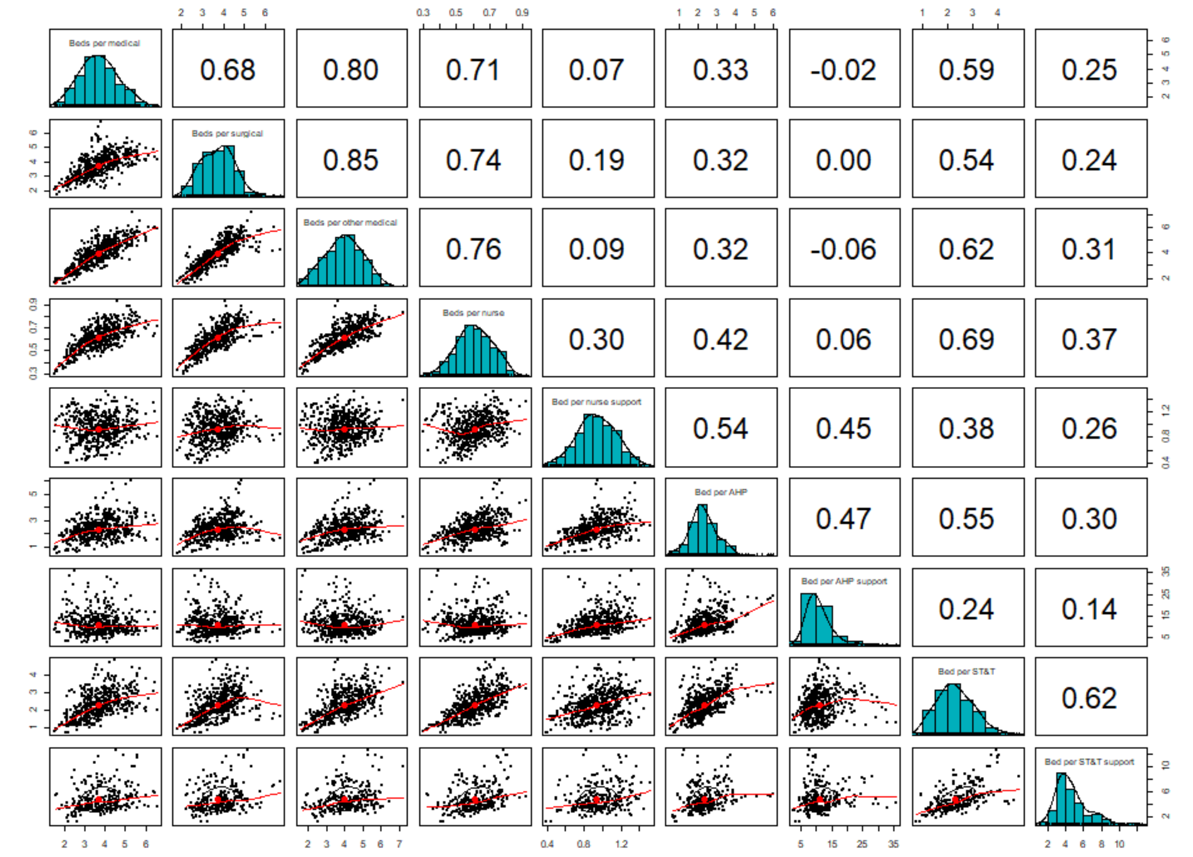

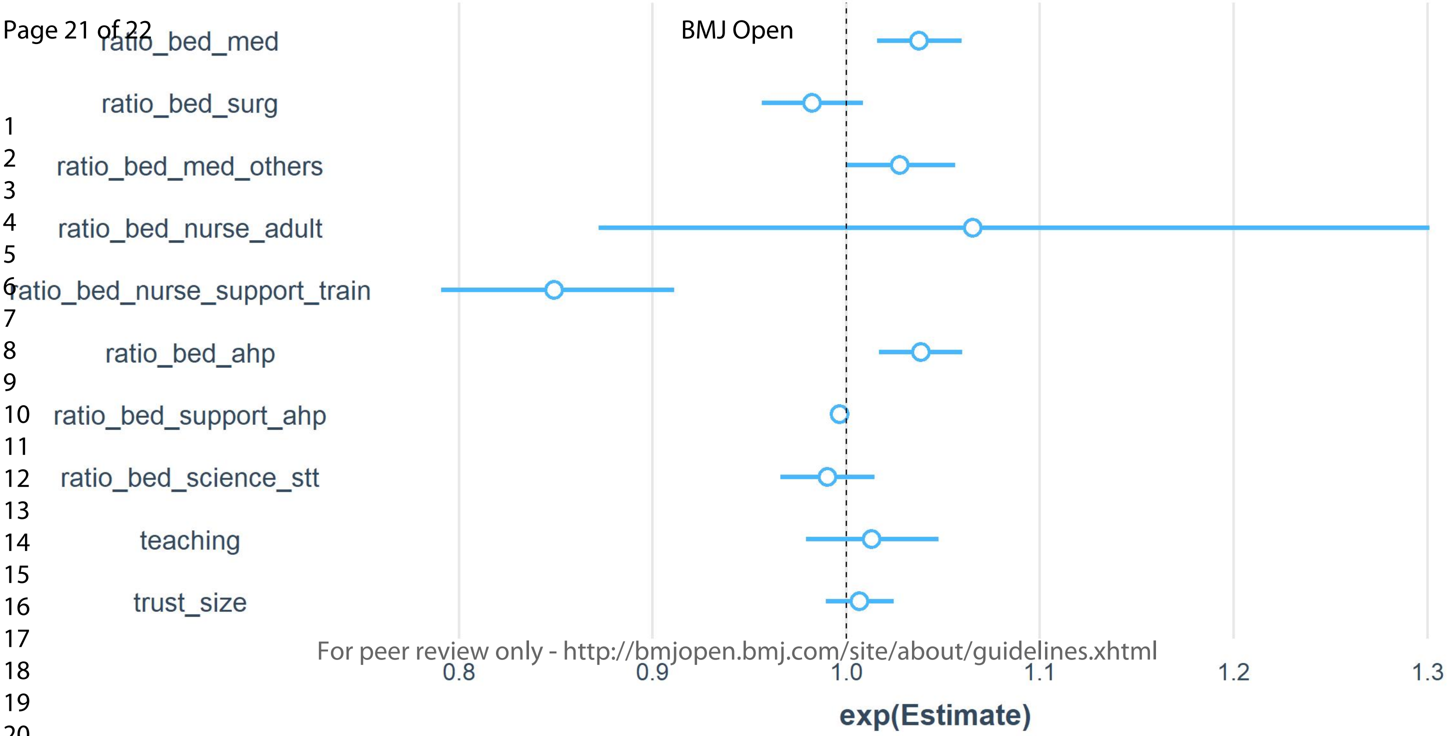
ratio_bed_med
ratio_bed_surg
ratio_bed_med_others
ratio_bed_nurse_adult
ratio_bed_nurse_support_train
ratio_bed_ahp
ratio_bed_support_ahp
ratio_bed_science_stt
teaching
trust_size
0.8
0.9
1.0
1.1
1.2
1.3
exp(Estimate)

STROBE Statement—Checklist of items that should be included in reports of ***cross-sectional studies***

	Item No	Recommendation	Page No
**Title and abstract**	1	(*a*) Indicate the study's design with a commonly used term in the title or the abstract	1
		(*b*) Provide in the abstract an informative and balanced summary of what was done and what was found	2
**Introduction**			
Background/rationale	2	Explain the scientific background and rationale for the investigation being reported	3
Objectives	3	State specific objectives, including any prespecified hypotheses	3
**Methods**			
Study design	4	Present key elements of study design early in the paper	3
Setting	5	Describe the setting, locations, and relevant dates, including periods of recruitment, exposure, follow-up, and data collection	3
Participants	6	(*a*) Give the eligibility criteria, and the sources and methods of selection of participants	3
Variables	7	Clearly define all outcomes, exposures, predictors, potential confounders, and effect modifiers. Give diagnostic criteria, if applicable	4-5
Data sources/ measurement	8*	For each variable of interest, give sources of data and details of methods of assessment (measurement). Describe comparability of assessment methods if there is more than one group	4-5
Bias	9	Describe any efforts to address potential sources of bias	3-5
Study size	10	Explain how the study size was arrived at	3
Quantitative variables	11	Explain how quantitative variables were handled in the analyses. If applicable, describe which groupings were chosen and why	3-5
Statistical methods	12	(*a*) Describe all statistical methods, including those used to control for confounding	5-6
		(*b*) Describe any methods used to examine subgroups and interactions	Not applicable
		(*c*) Explain how missing data were addressed	6
		(*d*) If applicable, describe analytical methods taking account of sampling strategy	Not applicable
		(*e*) Describe any sensitivity analyses	6, 8
**Results**			
Participants	13*	(a) Report numbers of individuals at each stage of study—eg numbers potentially eligible, examined for eligibility, confirmed eligible, included in the study, completing follow-up, and analysed	6
		(b) Give reasons for non-participation at each stage	Not applicable
		(c) Consider use of a flow diagram	Not applicable
Descriptive data	14*	(a) Give characteristics of study participants (eg demographic, clinical, social) and information on exposures and potential confounders	6

For peer review only

		(b) Indicate number of participants with missing data for each variable of interest	6
Outcome data	15*	Report numbers of outcome events or summary measures	6
Main results	16	(*a*) Give unadjusted estimates and, if applicable, confounder-adjusted estimates and their precision (eg, 95% confidence interval). Make clear which confounders were adjusted for and why they were included	7-8
		(*b*) Report category boundaries when continuous variables were categorized	7-8
		(*c*) If relevant, consider translating estimates of relative risk into absolute risk for a meaningful time period	Not applicable
Other analyses	17	Report other analyses done—eg analyses of subgroups and interactions, and sensitivity analyses	8
**Discussion**			
Key results	18	Summarise key results with reference to study objectives	8
Limitations	19	Discuss limitations of the study, taking into account sources of potential bias or imprecision. Discuss both direction and magnitude of any potential bias	9-10
Interpretation	20	Give a cautious overall interpretation of results considering objectives, limitations, multiplicity of analyses, results from similar studies, and other relevant evidence	8-10
Generalisability	21	Discuss the generalisability (external validity) of the study results	8-10
**Other information**			
Funding	22	Give the source of funding and the role of the funders for the present study and, if applicable, for the original study on which the present article is based	11

*Give information separately for exposed and unexposed groups.

Note: An Explanation and Elaboration article discusses each checklist item and gives methodological background and published examples of transparent reporting. The STROBE checklist is best used in conjunction with this article (freely available on the Web sites of PLoS Medicine at http://www.plosmedicine.org/, Annals of Internal Medicine at http://www.annals.org/, and Epidemiology at http://www.epidem.com/). Information on the STROBE Initiative is available at www.strobe-statement.org.

For peer review only

BMJ Open

Staffing levels and hospital mortality in England: a national panel study using routinely collected data

Journal:	*BMJ Open*
Manuscript ID	bmjopen-2022-066702.R2
Article Type:	Original research
Date Submitted by the Author:	17-Jan-2023

Complete List of Authors:	Rubbo, Bruna; University of Southampton, School of Health Sciences<br>Saville, Christina; University of Southampton, School of Health Sciences; National Institute for Health Research Applied Research Collaboration (Wessex), University of Southampton<br>Dall'Ora, Chiara; University of Southampton, School of Health Sciences; National Institute for Health Research Applied Research Collaboration (Wessex), University of Southampton<br>Turner, Lesley; University of Southampton, School of Health Sciences<br>Jones, Jeremy; University of Southampton, School of Health Sciences<br>Ball, Jane; University of Southampton, School of Health Sciences<br>Culliford, David; University of Southampton, Faculty of Medicine; National Institute for Health Research Applied Research Collaboration (Wessex), University of Southampton<br>Griffiths, Peter; University of Southampton, School of Health Sciences; National Institute for Health Research Applied Research Collaboration (Wessex), University of Southampton

**Primary Subject Heading**:	Health policy
Secondary Subject Heading:	Health services research
Keywords:	Organisation of health services < HEALTH SERVICES ADMINISTRATION & MANAGEMENT, Quality in health care < HEALTH SERVICES ADMINISTRATION & MANAGEMENT, Health policy < HEALTH SERVICES ADMINISTRATION & MANAGEMENT

SCHOLARONE™
Manuscripts

I, the Submitting Author has the right to grant and does grant on behalf of all authors of the Work (as defined in the below author licence), an exclusive licence and/or a non-exclusive licence for contributions from authors who are: i) UK Crown employees; ii) where BMJ has agreed a CC-BY licence shall apply, and/or iii) in accordance with the terms applicable for US Federal Government officers or employees acting as part of their official duties; on a worldwide, perpetual, irrevocable, royalty-free basis to BMJ Publishing Group Ltd ("BMJ") its licensees and where the relevant Journal is co-owned by BMJ to the co-owners of the Journal, to publish the Work in this journal and any other BMJ products and to exploit all rights, as set out in our licence.

The Submitting Author accepts and understands that any supply made under these terms is made by BMJ to the Submitting Author unless you are acting as an employee on behalf of your employer or a postgraduate student of an affiliated institution which is paying any applicable article publishing charge ("APC") for Open Access articles. Where the Submitting Author wishes to make the Work available on an Open Access basis (and intends to pay the relevant APC), the terms of reuse of such Open Access shall be governed by a Creative Commons licence – details of these licences and which Creative Commons licence will apply to this Work are set out in our licence referred to above.

Other than as permitted in any relevant BMJ Author's Self Archiving Policies, I confirm this Work has not been accepted for publication elsewhere, is not being considered for publication elsewhere and does not duplicate material already published. I confirm all authors consent to publication of this Work and authorise the granting of this licence.

For peer review only

Staffing levels and hospital mortality in England: a national panel study using routinely collected data

Bruna Rubbo[1], Christina Saville[1,2], Chiara Dall'Ora[1,2], Lesley Turner[1], Jeremy Jones[1], Jane Ball[1], David Culliford[2], Peter Griffiths[1,2]*

[1]University of Southampton, School of Health Sciences, Southampton, UK

[2]National Institute for Health Research Applied Research Collaboration, Wessex

*Corresponding author

peter.griffiths@soton.ac.uk (PG)

Short title: Staffing levels and hospital mortality

For peer review only - http://bmjopen.bmj.com/site/about/guidelines.xhtml

Abstract

Objectives

Examine the association between multiple clinical staff levels and case-mix adjusted patient mortality in English hospitals. Most studies investigating the association between hospital staffing levels and mortality have focused on single professional groups, in particular nursing. However, single staff group studies might overestimate effects or neglect important contributions to patient safety from other staff groups.

Design

Retrospective observational study of routinely available data.

Setting and participants

138 National Health Service hospital trusts that provided general acute adult services in England between 2015 and 2019.

Outcome measure

Standardised mortality rates were derived from the Summary Hospital level Mortality Indicator dataset, with observed deaths as outcome in our models and expected deaths as offset. Staffing levels were calculated as the ratio of occupied beds per staff group. We developed negative binomial random-effects models with trust as random effects.

Results

Hospitals with higher levels of medical and allied healthcare professional (AHP) staff (i.e. podiatry and chiropody, dietetics, occupational therapy, orthoptics and optics, physiotherapy, radiography, art, music and drama therapy, and speech and language therapy) had significantly lower mortality rates (rate ratio: 1.04, 95%CI 1.02 to 1.06, and 1.04, 95%CI 1.02 to 1.06, respectively), while those with higher support staff had higher mortality rates (0.85, 95%CI 0.79 to 0.91 for nurse support, and 1.00, 95%CI 0.99 to 1.00 for AHP support), after adjusting for multiple staff groups and hospital characteristics. Estimates of staffing levels on mortality were stronger between- than within-hospitals, which were not statistically significant in a within-between random effects model.

Conclusions

Considering multiple staff groups simultaneously when examining the association between hospital mortality and clinical staffing levels is crucial.

Article Summary

Strengths and limitations of the study

- We collected data from 138 hospitals over 4 years
- We included numerous clinical staffing groups simultaneously
- We did not have access to data at the ward level, so our associations were at the hospital level only.
- We used occupied beds-per-full time/contract staff, but this might not be a complete measure of staffing levels, as it does not take into account temporary staff or staff absence.

Keywords

Scheduling and staffing; Hospital Mortality; Workforce; Patient Safety; Delivery of Health Care

For peer review only - http://bmjopen.bmj.com/site/about/guidelines.xhtml

Introduction

Cost constraints and a quest for safer hospitals put pressure on health systems to find efficient staffing models to meet rising demand for care. Adequate nurse staffing levels in acute hospitals have important implications for positive outcomes for patients, including reducing patients' risk of death and costs.(1–5) Low registered nurse staffing levels are associated with failure to respond to patient abnormal changes in their physiological vital signs, which is in turn associated with failure to prevent death.(6) This link between registered nurses, failure to detect early deterioration and risk of death has meant the focus of much of the evidence and associated policy is on nurses. Nonetheless, the healthcare workforce is made up of many different professional groups, who must be mobilised to properly respond to or prevent deterioration and staffing levels of other staff groups are also likely to influence the quality and safety of care.

Studies focussing on the impact of clinical staff other than nurses on hospital mortality rates are relatively scarce. There are several studies on the effect of physician staffing levels, and they all indicate increased risk of patient death with lower medical staffing levels.(7–18) In-hospital patient care is delivered by multidisciplinary staff and no single staff group is solely responsible for patient outcomes. However, only a few studies considered multiple staff groups simultaneously, with some finding partial attenuation or complete absence of the effect of single staff levels on mortality when adjusted for other professionally qualified staff.(7,13,14,19) To our knowledge, no recent study has examined the potential effect of allied healthcare professionals (AHP) staffing levels on clinical outcomes, which include staff in the fields of podiatry and chiropody, dietetics, occupational therapy, orthoptics and optics, physiotherapy, radiography, art, music and drama therapy, and speech and language therapy. Only a few studies focused on in-hospital pharmacists.(19,20)

In the face of enduring staffing shortages in some professional groups it is important to understand the likely consequences on patient care and to identify priorities. A focus on single professional groups risks unintended consequences through neglect of other important contributions to patient safety and might lead to biased estimates. We therefore aimed to examine the associations between staffing levels of multiple staff groups, and case-mix adjusted patient mortality in hospitals.

Methods

Study design

This was a retrospective observational study using routinely available data on clinical healthcare staffing and hospital mortality.

Study setting

We included all 138 National Health Service (NHS) hospital trusts providing general acute adult inpatient services in England between April 2015 and March 2019. Hospital trusts are defined as organisational units within the NHS that service a defined geographical area or that provide a specialised function. One trust can therefore encompass several hospitals.

For peer review only - http://bmjopen.bmj.com/site/about/guidelines.xhtml

Data sources and linkage

We linked four data sources that provide trust-level datasets: a) NHS workforce, which contains detailed information on trust staffing; b) bed availability and occupancy, which contains data on trust-level available and occupied beds; c) Estates Returns Information Collection (ERIC), which contains data on trust organisation and structure; and d) Summary Hospital-level Mortality Indicator (SHMI), which contains data on observed and expected deaths (Table S1). These datasets are openly available and accessible on the NHS Digital platform, along with the data dictionary.(21,22) We linked the datasets using the unique hospital trust ID (*i.e.* “Org code”).

Standardised mortality rates derived from the SHMI includes all deaths that occur in hospital or within 30-days of discharge among patients admitted to non-specialist acute trusts in the period of a year. SHMI data are derived from the Hospital Episode Statistics (HES) at the level of provider spells (*i.e.* total continuous stay of a patient using a hospital bed at an NHS organisation under the care of one or more consultants, or nursing episode or midwife episode), and the HES-Office of National Statistics (ONS) linked mortality data (Table S1).(23) The latter captures deaths that occur outside of hospital. Data used to calculate the SHMI are submitted by each hospital trust.

SHMI expected deaths is calculated based on individual patient characteristics that can affect the risk of mortality, including the patient’s condition for hospitalisation, underlying conditions (Charlson Comorbidity Index),(24) age, gender, method of admission to hospital, and year of discharge. Logistic regression models estimate the risk for each provider spell, with binary variable as outcome (*i.e.* died or survived). The SHMI model performs well with early validation accounting for 81% of between-hospital variations.(25) The models are constructed using the preceding three years’ data, with last year data used to calculate the standardised mortality rates (26). As SHMI data are published monthly, we used the dataset that contains mortality data from April to March the following year to report on annual hospital mortality level for each hospital trust included in this study. Additional information, including dataset and reports, can be found at the NHS digital website (https://digital.nhs.uk/data-and-information/publications/ci-hub/summary-hospital-level-mortality-indicator-shmi).

We obtained hospital staffing data by linking the medical and dental workforce dataset (physicians) with the non-medical workforce dataset (all other healthcare workers, including nurses, nurse assistants, physiotherapists, pharmacists, among others). Bed occupancy data are published quarterly as averages, with no estimate of monthly variation. For wards open overnight, occupied bed is defined as when it is occupied at midnight on the day in question; for day-only wards, occupied bed is a bed where at least one day case has taken place during the day. Trust teaching status was derived from the ERIC dataset, which contains variables on trust profile.

Staffing variables are published monthly. We used full-time equivalent (FTE) values to calculate the annual average available staff at each hospital trust from April to March the following year, to align with the SHMI data. FTE data are standardised measures of the workload that allow for the total workforce workload to be expressed in an equivalent number of full-time staff and are based on the proportion of time each staff are expected to work in a week, which would correspond to an FTE of 1 (*e.g.* 48 hours for doctors, 37.5h for nurses and AHP). Overtime and out-of-hour work are not recorded in these datasets.

For peer review only - http://bmjopen.bmj.com/site/about/guidelines.xhtml

Study outcome

The main study outcome was annual standardised all-cause mortality rate derived from observed and expected deaths in the SHMI dataset.(27) This is calculated and reported for all trusts providing acute adult services in NHS England.(25) Specialist trusts that do not provide general acute care do not report SHMI and, therefore, were not included in the study.

Staffing variables

Clinical staff are classified according to occupation codes. We grouped staffing variables by frequency and occupation as listed by NHS England, merging groups that had mean below 3 with others of a similar occupation in order to create 8 groups (*i.e.* medical, surgical, other medical specialties, nurses, support to nurses, AHP, support to AHP, and scientific, therapeutic, and technical (ST&T) staff). General medicine specialist doctors were grouped with clinical oncology doctors to form the general medicine group, surgeons were grouped with obstetricians and gynaecologists to form the surgical group, and all other medical specialties were grouped together to form the other medical group (*i.e.* radiologists, emergency medicine, anaesthetists, and pathologists). We excluded psychiatrists and paediatricians. The nurses' group was composed of nurses in adult services only; we did not include nurses from paediatric services. The nurse support group included support to adult and general nurses, and nurses in training. Scientific, therapeutic, and technical (ST&T) staff group was composed of multi-therapists, applied psychologist, psychological therapist, pharmacists, dentists, operating theatre staff, social services, and other ST&T staff.

We used the number of general acute occupied beds to calculate the bed-per-staff ratio. Beds assigned to maternity, mental illness, and learning disabilities services were excluded; however, general acute beds represented 96.2% of the total beds available across all hospital trusts included in the study. We calculated the average number of occupied beds overnight or day-only per trust per year, and then divided occupied beds per each staff group to obtain the average bed-per-staff levels for each hospital trust per year.

Hospital-level covariates

Teaching affiliation was coded as yes or no according to the trust type recorded in the ERIC dataset. Trust size was calculated based on the number of available general acute beds in each trust.

Statistical analysis

We initially explored the data using descriptive statistics. Continuous variables were described as mean and standard deviation (SD) or median and IQR, depending on the distribution of each variable. Categorical variables were described as frequencies and proportions. Annual mortality rates were calculated by dividing the number of observed deaths by the number of patient spells, across all hospital trusts each year. To explore the relationship between staffing levels and hospital mortality, statistical modelling was conducted at the hospital trust-level using multiple regression models on four years of data, with standardised mortality rates regressed onto hospital level staffing levels (expressed as the number of occupied beds-per-staff). We included expected deaths as an offset, as the number of expected deaths is the number of times the event could have happened (*i.e.* the exposure variable).

Multilevel models were adjusted for trust size and affiliation as a teaching hospital, with trust included as a random effect to adjust for clustering. We considered a range of potential

For peer review only - http://bmjopen.bmj.com/site/about/guidelines.xhtml

models, with model selection based on theoretical reasoning and model fit (minimising the Akaike information criterion (AIC), the Bayesian information criterion (BIC) and the likelihood ratios (see Table S2 for alternative frameworks considered)). We report exponentiated coefficients (rate ratios) with 95% confidence intervals (CI) for all estimates obtained from the models.

The best performing model (referred to as main model) was a negative binomial random effects model that included all 8 clinical staff groups and hospital characteristics (*i.e.* trust size and teaching status). To explore variations between and within hospital trusts, we also constructed a within-between random effects model (WBRE), with trusts as random effect, and staffing and hospital characteristics as fixed effects. These models can differentiate between effects arising from staffing differences between hospital trusts and those that are associated with annual changes of staffing levels within each hospital by group-mean centring each trust.(28,29)

We assessed potential collinearity between co-variates using correlation plots and Spearman's correlation coefficient, and the overall multicollinearity for all predictors in the models using generalised variance inflation factors (GVIF). All models had a GVIF<10. As a sensitivity analysis, we reran the models after excluding any variables that had a GVIF above 5.(30)

Observations with missing data were removed from the analyses; however, hospital trusts that reported at least one year of complete data were included.

We performed data linkage, cleaning, coding, and analyses in R statistical software, version 4.0.2 (R Core Team, R Foundation for Statistical Computing, Vienna, Austria), using the lme4 and glmer packages (v1.1-27.1, Bates et al, 2015), and the plm package (v2.4-3, Croissant et al, 2021).(31) The full dataset is available.(32)

Patient and public involvement

There was no patient and public involvement in this study.

Results

Descriptive statistics

There were 540 observations in the linked dataset for the 4-year study period, of which 519 (96%) contained data on all variables and were therefore included in our analyses. There were 2 hospital trusts that did not report on medical staffing in 2016, with 6 trusts not reporting these variables from 2017 onwards. For other clinical staff, only 1 trust did not report staff numbers in 2016 and 2017, and two trusts in 2018, with no missing data in 2019. One trust did not report the number of occupied general acute beds in 2018. The number of hospital trusts that reported on complete data varied, with 137 included in 2016 and 2017, 135 in 2018, and 130 in 2019.

The median number of acute general occupied beds per day was 674.0 (IQR 486.1 to 911.6). The mean annual observed deaths ± SD was 2229 ± 992 (range 525 to 7468), while the expected deaths were 2228 ± 987.2 (range 665 to 7591). Annual mortality rates across all hospital trusts were 3.44% for 2016, 3.46% for 2017, 3.23% for 2018, and 3.31% for 2019. There was a median of 744.2 available general acute beds per trust (interquartile range (IQR) 535.7 to 1009.4, range 143 to 2704). Hospital trusts in the upper tertile (*i.e.* the upper third of trusts, ranked by size) were classified as large, while those in the lower tertile were deemed small trusts, with the remaining classified as medium-sized hospital trusts. Forty-one (29.7%) hospital trusts were classified as teaching trusts.

For peer review only - http://bmjopen.bmj.com/site/about/guidelines.xhtml

Table 1 shows the distribution of staffing variables by their median, IQR, mean, and SD across all hospital trusts. There was considerable variation in staffing levels, with nurse staff levels showing least relative variation (SD 18.0% of the mean) and AHP support most (SD 44.4% of the mean). By contrast, variations within trusts as a percentage of the mean were relatively low, with the within trust variation ranging from 4.9% for nurse staffing to 10.9% for AHP support.

Table 1. Frequency of occupied bed-per-staff variables across 138 hospital trusts

Staffing level variables	Median (IQR)	Mean (SD)	SD as % of mean	Mean within-trust SD	Within-trust SD as % of mean
Bed per medical	3.66 (3.05 to 4.30)	3.70 (0.96)	25.9%	0.28	7.6%
Bed per surgical	3.79 (3.10 to 4.28)	3.73 (0.81)	21.7%	0.22	5.9%
Bed per other medical specialties	3.96 (3.26 to 4.69)	3.94 (1.01)	25.6%	0.27	6.9%
Bed per nurse adult service	0.61 (0.54 to 0.68)	0.61 (0.11)	18.0%	0.03	4.9%
Bed per nurse support	0.93 (0.80 to 1.08)	0.93 (0.21)	22.6%	0.07	7.5%
Bed per AHP	2.25 (1.88 to 2.74)	2.36 (0.89)	37.7%	0.18	7.6%
Bed per AHP support	10.03 (7.91 to 13.01)	11.07 (4.91)	44.4%	1.21	10.9%
Bed per ST&T	2.24 (1.80 to 2.82)	2.31 (0.75)	32.5%	0.16	6.9%

Q1: lower quartile, Q3: upper quartile, SD: standard deviation, AHP: allied healthcare professional, ST&T: scientific, therapeutic, and technical

All groups of medical staff were highly correlated with each other (range rho=0.68 to 0.85) (Figure S1). Nurse staffing levels were strongly correlated with staffing levels in all medical staff groups (rho>0.71). Nurse support staff levels were correlated to RN (rho=0.30), as were AHP and AHP support (rho=0.47), but neither support staff were correlated to medical staff (0.07 and -0.02, respectively). As the correlations between registered staff and their support were weak to moderate, there does not seem to be a substitution effect between these groups.

Staffing levels and hospital mortality

Table 2 shows results for the negative binomial random effect models, adjusted for hospital characteristics. In the single staff group models, hospital trusts that had lower staffing levels (*i.e.* more occupied beds per FTE staff) were associated with higher (standardised) mortality rates for all professionally qualified staff groups. The opposite effect was observed for

For peer review only - http://bmjopen.bmj.com/site/about/guidelines.xhtml

nursing support and AHP support staff, where hospital trusts with lower staffing levels (*i.e.* more occupied beds per FTE staff) had lower mortality.

Table 2. Exponentiated estimates (rate ratios) for hospital mortality from the multilevel negative binomial random effect models, adjusted for hospital characteristics (*n* = 138)

**Predictors**	**Estimates for single staff models**	**95% CI**	**Estimates for multiple staff model**	**95% CI**
Bed per medical	1.05***	1.04 to 1.07	1.04***	1.02 to 1.06
Bed per surgical	1.04***	1.02 to 1.06	0.98	0.96 to 1.01
Bed per other medical specialties	1.05***	1.03 to 1.06	1.03*	1.00 to 1.06
Bed per nurse adult service	1.33***	1.15 to 1.54	1.07	0.88 to 1.31
Bed per nurse support	0.92*	0.86 to 0.98	0.85***	0.79 to 0.91
Bed per AHP	1.02*	1.00 to 1.04	1.04***	1.02 to 1.06
Bed per AHP support	0.99*	0.99 to 0.99	1.00**	0.99 to 1.00
Bed per ST&T	1.02**	1.00 to 1.04	0.99	0.98 to 1.05
Teaching (ref not teaching)	0.96*	0.92 to 0.99	1.01	0.98 to 1.05
Medium trust (ref small trust)	1.01	0.97 to 1.05	1.01	0.98 to 1.04
Large trust (ref small trust)	1.00	0.96 to 1.04	1.01	0.98 to 1.05

CI: confidence interval, AHP: allied healthcare professional, ST&T: scientific, therapeutic, and technical, * p<0.05, ** p<0.01, *** p<0.001. Estimates are a ratio of observed to expected deaths.

In the main model including all staff groups, associations between medical (rate ratio: 1.04, 95%CI 1.02 to 1.06), other medical specialties (1.03, 95%CI 1.00 to 1.06), AHP (1.04, 95%CI 1.02 to 1.06), nurse support (0.85, 95%CI 0.79 to 0.91), and AHP support (1.00, 95%CI 0.99 to 1.00) FTE staffing levels and hospital mortality remained statistically significant, with no change in direction of the effect compared to the single staff models (Table 2). Higher mortality rates were observed in hospital trusts with lower levels of medical and AHP staff. In contrast, hospital trusts with lower support staff levels per occupied bed (*i.e.* nursing support and AHP support) had lower mortality rates. The association with RN staffing was attenuated and no longer statistically significant (1.07, 95%CI 0.88 to 1.31), although the observed effect remained relatively large compared to other associations (Table S3). Similarly, the association between hospital mortality and surgical and ST&T staff, observed in the single staff groups models, were no longer statistically significant in the model adjusted for all staff groups.

Although we noted correlation between staffing variables, our GVIF for all variables was <10. The GVIF for beds per other medical specialties was 6.1 (Table S3), and therefore over our threshold of 5, so we ran the negative binomial random effects model omitting this variable as a sensitivity analysis and found similar results (not shown; available from authors upon request).

Within-between hospital trust variability

Between-hospital trust effects from the WBRE model were largely the same as those obtained from the main model (*i.e.* between only). For example, the between- rate ratio estimate from WBRE for the FTE medical group was 1.04 (95%CI 1.02 to 1.07), similar to the estimates obtained from the main model (1.04, 95%CI 1.02 to 1.06). However, even for staff groups where between-hospital trusts effects were significant, the within-hospital effects were small and not statistically significant. For example, the within-hospital estimate from the WBRE model for the medical group was 0.99 (95%CI 0.97 to 1.01) (Table 3). Despite potentially providing additional information by decomposing the variability into between- and

within-estimates, the WBRE model did not improve fit compared to the main model (Table S2).

Table 3. Exponentiated estimates (rate ratios) for hospital mortality from the multilevel within-between negative binomial random effect models, adjusted for hospital characteristics (*n* = 138).

	Observed deaths		
**Predictors**	**Incidence Rate Ratios**	**95% confidence intervals**	**p-values**
**Bed per medical**			
Within-hospital	0.98	0.97 – 1.00	0.129
Between-hospital	1.04	1.02 – 1.07	**<0.001**
**Bed per surgical**			
Within-hospital	1.03	0.99 – 1.06	0.106
Between-hospital	0.98	0.95 – 1.01	0.222
**Bed per other medical specialties**			
Within-hospital	1.02	0.99 – 1.04	0.21
Between-hospital	1.02	0.99 – 1.06	0.124
**Bed per nurse**			
Within-hospital	0.88	0.75 – 1.04	0.148
Between-hospital	1.1	0.88 – 1.37	0.393
**Bed per nurse support**			
Within-hospital	0.99	0.91 – 1.07	0.789
Between-hospital	0.84	0.77 – 0.90	**<0.001**
**Bed per AHP**			
Within-hospital	0.99	0.96 – 1.01	0.296
Between-hospital	1.04	1.02 – 1.07	**<0.001**
**Bed per AHP support**			
Within-hospital	1	1.00 – 1.00	0.352
Between-hospital	1	0.99 – 1.00	**0.017**
**Bed per ST&T**			
Within-hospital	1.01	0.99 – 1.04	0.329
Between-hospital	0.99	0.96 – 1.01	0.266
**Teaching hospital**			
Yes	1.02	0.98 – 1.05	0.323
**Trust size (reference: small)**			
Medium	1	0.98 – 1.03	0.706
Large	1.01	0.98 – 1.04	0.381

Discussion

After adjustment for hospital characteristics and including multiple clinical staff groups in our models, we found that higher medical and AHP staffing levels were associated with lower mortality rates in acute hospital trusts in England during the period of 2015 to 2019. In contrast, hospitals with higher support staff per occupied bed had higher mortality rates.

Our study highlights the importance of simultaneously adjusting for multiple clinical staff groups when investigating associations between staffing levels and mortality at the hospital-level. In the single staff group models, significant effects on mortality rates were seen for all

For peer review only

For peer review only - http://bmjopen.bmj.com/site/about/guidelines.xhtml

staffing groups. However, when adjusted for multiple groups, effects for medical, other medical specialties, AHP, nurse support, and AHP support staffing levels remained statistically significant, but levels of surgical doctors, RN, and ST&T staff were no longer significant.

Our findings are in line with a previous study by Jarman *et al*,(7) who found that the association between RN staffing and mortality was no longer significant when adjusting for doctors-per-bed, with higher levels of physician staffing significantly reducing hospital standardised mortality ratios.

Nonetheless, other studies have shown a reduction in hospital mortality when both higher numbers of doctors and nurses per bed were observed.(11,13–15,33–36) The fact that the association between nurse staffing and mortality was not significant in the multivariable models should not be taken to indicate an absence of effect to nursing. Previous studies have reported that much of the variation in nurse staffing occurs between wards within hospitals and within wards over time (1,37) and Keogh noted that hospital nurse staffing levels often bore little relationship to staffing available to be deployed on wards in NHS hospitals,(38) therefore hospital nurse staffing per bed may be a poor indicator of the staffing levels experienced by inpatients. Griffiths *et al* (13) found that when medical staffing levels were accounted for, significant nurse staffing effects were only observed in models using ward-based staffing ratios as opposed to hospital-level staff-per-bed ratios. Longitudinal patient level studies of exposure to variation in nurse staffing confirm that there is an adverse effect of low nurse staffing when measured at this level.(2,39,40)

To our knowledge, this was the first study to include AHP and AHP support staff levels in analysis. Higher levels of AHP staff had a protective effect at the hospital trust level while more AHP support staff were detrimental to patient mortality, a finding that mirrors those obtained for nursing support staff. While causality cannot be assumed for any of these results, there is a potential that previous findings that have emphasised a link between nurse staffing and patient safety could divert attention from the role that other staff groups may play in delivering quality and preventing avoidable deaths.

In our study, high levels of support staff were associated with higher mortality rates. This finding appears to be paradoxical: increasing the number of some staff groups might lead to increased mortality, when holding all other staffing variables constant. This cannot be fully explored with the given data. One potential reason might be that raising levels of one staff group might affect the distribution of other staff groups to different departments and wards and this could negatively affect mortality. Another possible reason could be that increasing the levels of one staff group might alter the duties of other staff groups – for example, adding more nurse assistants while holding the number of registered nurses constant might increase the workload for the registered nurses as they are now responsible for the supervision of a larger number of nurse assistants, or create an implicit demand for delegation of work that is not suited to the assistants, which could negatively impact mortality.(39) Unfortunately, due to the fact that we do not have granular in-hospital data on deployment of staff and staff responsibility, we are unable to further investigate this, although this mechanism is consistent with evidence on skill mix in the nursing team which shows a tipping point where adding assistants has a positive effect when the number of assistant is low, but a negative one where the number is high.(39)

We found that variations between hospital trusts in the 4-year study period were higher in magnitude than within-hospital trusts estimates, which were not statistically significant for any of the clinical staff groups at the hospital level. Structural recruitment and retention difficulties, financial constraints, and other non-staffing resources available at each hospital could be main contributors to inter-hospital trust variations. There was relatively little year on year variation in staffing between trusts. Reduced variability of staffing levels within hospitals might be due to the limited number of funded posts in each organisation and could reflect

For peer review only - http://bmjopen.bmj.com/site/about/guidelines.xhtml

historic staffing levels. Bjerregaard *et al*,(36) using a WBRE model, found staffing levels estimates were significant between wards, while within-ward estimates had no significant effect on hospital mortality. Bjerregaard et al.(36) proposed that between hospital effects may reflect structural differences in staffing resource between hospitals while the limited within hospital variation we observed could be largely endogenous, reflecting change in staff in response to variation in patient populations and risk that is not reflected in the case mix adjustment model. This proposition remains untested.

Strengths and limitations

This was a national study using 4 years of routinely available data. The inclusion of numerous clinical staffing groups simultaneously in the models allowed us to estimate the effect of each staffing level on hospital mortality while considering hospital trust characteristics.

We used a variety of models and selected the one that performed best on our data. Most studies investigating clinical staffing levels and in-hospital mortality used fixed effects models, which only evaluate within-hospital effects. Such models might not appropriately assess the effect of different staffing levels based on variation between hospitals in hospital-level observational studies.

However, our study was limited by the relatively small sample size, as observations were clustered on 138 hospital trusts. We therefore had to group some of the medical specialties and were unable to unpack relatively heterogenous groups such as AHP. Furthermore, we were unable to explore the effects of grade and experience for medical (*e.g.* junior doctors, level of consultants) and RN (*e.g.* senior nurses) staff.

In our study, beds that were occupied by multiple patients in a single day were counted as one single occupied bed. Studies have shown that workload (*e.g.* number of admissions, discharges, additional tasks) in high volume hospitals can lead to higher mortality rates, even when levels of staffing are similar to those observed in low volume hospitals. Occupied beds-per-staff might not be a good measure of staffing levels, as these do not account for temporary staff or staff absence due to sickness, maternity leave, or long-term leave. The extent to which staff are deployed to deliver services such as ambulatory care or other services not delivered to inpatient beds is likely to vary.(41) FTE might not be an accurate representation of actual hours worked by the clinical staff as trusts with similar FTE staff might have a higher or lower rotation of staff responsible for a single bed during a shift, which could affect the quality of care, including mortality rates.(42) Additionally, our bed-to-staff rations might have been overestimated, as we could not include overtime and out-of-hour since these data are not captured in the datasets used for this study.

Future studies should aim to capture different staffing levels responsible for delivering patient care measured at the patient or ward-level, rather than at the hospital-level.

Although SHMI provides good control for patient risk and we controlled for hospital factors and multiple staff groups, the cross-sectional nature of our analysis means that findings need to be interpreted as demonstrating association but not direct evidence of causation.

Conclusion

In conclusion, our study highlights the importance of simultaneously considering multiple staff groups when investigating the effect of clinical staffing levels and mortality at the hospital-level. We showed that the number of AHP and AHP support per occupied beds have a significant impact on patient mortality, yet these groups have largely not been included in previous workforce studies, of which the majority focused exclusively on nursing

For peer review only - http://bmjopen.bmj.com/site/about/guidelines.xhtml

staff. We also found that hospitals with higher levels of medical staffing had lower mortality rates, while higher levels of support workers were associated with higher hospital mortality.

Data Statement

The dataset is available from the ePrints repository, DOI: https://doi.org/10.5258/SOTON/D2055

Authors' contributions

BR, CS, CDO, LT, JJ, JB, DC, and PG made substantial contributions to the conception or design of the work. BR, JJ, PG acquired the data, BR analysed the data, CS and DC independently checked the data analyses, BR, CS, CDO, LT, JJ, JB, DC, and PG discussed the interpretation of data for the work; BR drafted the work, and BR, CS, CDO, LT, JJ, JB, DC, and PG revised it critically for important intellectual content and gave final approval of the version to be published; PG is accountable for all aspects of the work in ensuring that questions related to the accuracy or integrity of any part of the work are appropriately investigated and resolved.

Conflicts of interest

All authors have completed the ICMJE uniform disclosure form at http://www.icmje.org/disclosure-of-interest/ and declare: all authors had financial support from the NIHR Health Services and Delivery Research for the submitted work; no financial relationships with any organisations that might have an interest in the submitted work in the previous three years; no other relationships or activities that could appear to have influenced the submitted work."

Funding

This study/project is funded by the NIHR [Health and Social Care Delivery Research (NIHR128056)]. The views expressed are those of the author(s) and not necessarily those of the NIHR or the Department of Health and Social Care.

Ethics Approval

We did not need ethics approval as this project relied on publicly available routinely collected data.

For peer review only - http://bmjopen.bmj.com/site/about/guidelines.xhtml

References

1 Griffiths P, Ball J, Bloor K, et al. Nurse staffing levels, missed vital signs and mortality in hospitals: retrospective longitudinal observational study. *Health Services and Delivery Research* 2018;6:38

2. Dall'Ora C, Saville C, Rubbo B, et al. Nurse staffing levels and patient outcomes: A systematic review of longitudinal studies. *Int J Nurs Stud*. 2022;134:104311.

3. Dietermann K, Winter V, Schneider U, et al. The impact of nurse staffing levels on nursing-sensitive patient outcomes: a multilevel regression approach. Eur J Health Econ. 2021;22(5):833–46.

4. Kane RL, Shamliyan TA, Mueller C, et al. The association of registered nurse staffing levels and patient outcomes: systematic review and meta-analysis. Med Care. 2007;45(12):1195–204.

5. Winter V, Dietermann K, Schneider U, et al Nurse staffing and patient-perceived quality of nursing care: a cross-sectional analysis of survey and administrative data in German hospitals. *BMJ Open*. 2021;11(11):e051133.

6. Hogan H, Healey F, Neale G, et al. Preventable deaths due to problems in care in English acute hospitals: a retrospective case record review study. *BMJ Qual Saf*. 2012;21(9):737–45.

7. Jarman B, Gault S, Alves B, et al. Explaining differences in English hospital death rates using routinely collected data. *BMJ*. 1999;318(7197):1515–20.

8. Pronovost PJ, Angus DC, Dorman T, et al. Physician staffing patterns and clinical outcomes in critically ill patients: a systematic review. *JAMA*. 2002;288(17):2151–62.

9. Lin HC, Lee HC. Caseload volume-outcome relation for pulmonary embolism treatment: association between physician and hospital caseload volume and 30-day mortality. *J Thromb Haemost*. 2008;6(10):1707–12.

10. Ong M, Bostrom A, Vidyarthi A, et al. House staff team workload and organization effects on patient outcomes in an academic general internal medicine inpatient service. *Arch Intern Med*. 2007;167(1):47–52.

11. Ricciardi R, Nelson J, Roberts PL, et al. Is the presence of medical trainees associated with increased mortality with weekend admission? *BMC Med Educ*. 2014;14:4.

12. Coit MH, Katz JT, McMahon GT. The effect of workload reduction on the quality of residents' discharge summaries. *J Gen Intern Med*. 2011;26(1):28–32.

13. Griffiths P, Ball J, Murrells T, et al. Registered nurse, healthcare support worker, medical staffing levels and mortality in English hospital trusts: a cross-sectional study. *BMJ Open*. 2016;6(2):e008751.

14. Bond CA, Raehl CL, Pitterle ME, et al. Health care professional staffing, hospital characteristics, and hospital mortality rates. *Pharmacotherapy.* 1999;19(2):130–8.

15. West E, Barron DN, Harrison D, et al. Nurse staffing, medical staffing and mortality in Intensive Care: An observational study. *Int J Nurs Stud.* 2014;51(5):781–94.

For peer review only - http://bmjopen.bmj.com/site/about/guidelines.xhtml

16. Dara SI, Afessa B. Intensivist-to-bed ratio: association with outcomes in the medical ICU. *Chest.* 2005;128(2):567–72.

17. Neuraz A, Guérin C, Payet C, et al. Patient Mortality Is Associated With Staff Resources and Workload in the ICU: A Multicenter Observational Study. *Crit Care Med*. 2015;43(8):1587–94.

18. Gershengorn HB, Harrison DA, Garland A, et al. Association of Intensive Care Unit Patient-to-Intensivist Ratios With Hospital Mortality. *JAMA Intern Med*. 2017;177(3):388–96.

19. Lee H, Ryu K, Sohn Y, et al. Impact on Patient Outcomes of Pharmacist Participation in Multidisciplinary Critical Care Teams: A Systematic Review and Meta-Analysis. *Crit Care Med*. 2019;47(9):1243–50.

20. Bond CA, Raehl CL, Franke T. Interrelationships among mortality rates, drug costs, total cost of care, and length of stay in United States hospitals: summary and recommendations for clinical pharmacy services and staffing. *Pharmacotherapy*. 2001;21(2):129–41.

21. [dataset] NHS Digital. National Workforce Data Set (NWD) and NHS occupational codes. [accessed 2022 Nov 02]. Available from: https://digital.nhs.uk/data-and-information/data-collections-and-data-sets/data-sets/national-workforce-data-set-nwd-and-nhs-occupation-codes#top

22. NHS Digital. NHS Data Model and Dictionary. NHS England; [accessed 2022 Nov 02]. Available from: https://datadictionary.nhs.uk/index.html

23. Aylin P, Williams S, Bottle A, Jarman B. Counting hospital activity: spells or episodes? *BMJ*. 2004;329(7476):1207.

24. Charlson ME, Pompei P, Ales KL, et al. A new method of classifying prognostic comorbidity in longitudinal studies: development and validation. *J Chronic Dis.* 1987;40(5):373–83.

25. Campbell MJ, Jacques RM, Fotheringham J, et al. Developing a summary hospital mortality index: retrospective analysis in English hospitals over five years. *BMJ.* 2012;344:e1001.

26. Campbell MJ, Jacques RM, Fotheringham J, et al. An evaluation of the Summary Hospital Mortality Index. University of Sheffield; 2011.

27. NHS Digital. The processing cycle and HES data quality NHS Digital: NHS England. [accessed 2022 Feb 11]. Available from: https://digital.nhs.uk/data-and-information/data-tools-and-services/data-services/hospital-episode-statistics/the-processing-cycle-and-hes-data-quality

28. Bell A, Fairbrother M, Jones K. Fixed and random effects models: making an informed choice. *Qual Quant.* 2019;53(2):1051–74.

29. Schunck R. Within and between Estimates in Random-Effects Models: Advantages and Drawbacks of Correlated Random Effects and Hybrid Models. *Stata J.* 2013;13(1):65–76.

For peer review only

30. O'brien RM. A Caution Regarding Rules of Thumb for Variance Inflation Factors. *Qual Quant*. 2007;41(5):673–90.

31. R Core Team (2018). R: A language and environment for statistical computing. R Foundation for Statistical Computing, Vienna, Austria. [accessed 2022 Nov 08]. Available online at https://www.R-project.org/.

32. [dataset] Rubbo B, Griffiths P, Saville C, et al. NHS England staffing levels and mortality dataset. University of Southampton; 2021. Available at DOI: 10.5258/SOTON/D2055

33. Griffiths P, Jones S, Bottle A. Is "failure to rescue" derived from administrative data in England a nurse sensitive patient safety indicator for surgical care? Observational study. *Int J Nurs Stud*. 2013;50(2):292–300.

34. Ozdemir BA, Sinha S, Karthikesalingam A, et al. Mortality of emergency general surgical patients and associations with hospital structures and processes. *Br J Anaesth*. 2016;116(1):54–62.

35. Chung W, Sohn M. The Impact of Nurse Staffing on In-Hospital Mortality of Stroke Patients in Korea. *J Cardiovasc Nurs*. 2018;33(1):47–54.

36. Bjerregaard U, Hølge-Hazelton B, Rud Kristensen S, et al. Nurse Staffing and Patient Outcomes: Analyzing Within- and Between-Variation. *DaCHE Discussion Papers.* 2020

37. Van den Heede K, Sermeus W, Diya L, et al. Nurse staffing and patient outcomes in Belgian acute hospitals: cross-sectional analysis of administrative data. *Int J Nurs Stud.* 2009;46(7):928–39.

38. Keogh B. Review into the Quality of Care and Treatment Provided by 14 Hospital Trusts in England: Overview Report. London, UK: National Health Service; 2013 [accessed 2022 Nov 2]. Available from: https://psnet.ahrq.gov/issue/review-quality-care-and-treatment-provided-14-hospital-trusts-england-overview-report

39. Griffiths P, Maruotti A, Recio Saucedo A, et al. Nurse staffing, nursing assistants and hospital mortality: retrospective longitudinal cohort study. *BMJ Qual Saf.* 2019;28(8):609–17.

40. Needleman J, Buerhaus P, Pankratz VS, et al. Nurse staffing and inpatient hospital mortality. *N Engl J Med.* 2011;364(11):1037–45.

41. Ball J. Nurse staffing, care left undone and patient mortality in acute hospitals. Doctoral thesis. Inst för lärande, informatik, management och etik / Dept of Learning, Informatics, Management and Ethics; 2017 [accessed 2022 Nov 2]. Available from: http://openarchive.ki.se/xmlui/handle/10616/45563

42. Denson JL, Jensen A, Saag HS. Associations between end-of-rotation resident transition in care and mortality among hospitalized patients. *JAMA* 2016;316(21):2204-2213.

Supporting information captions

Table S1. NHS digital data sources

Table S2. Comparison of goodness-of-fit for model selection

Table S3. Multicollinearity test in the main model (*i.e.* negative binomial random effect)

Figure S1. Spearman correlation between staffing level variables included in the study.

For peer review only - http://bmjopen.bmj.com/site/about/guidelines.xhtml

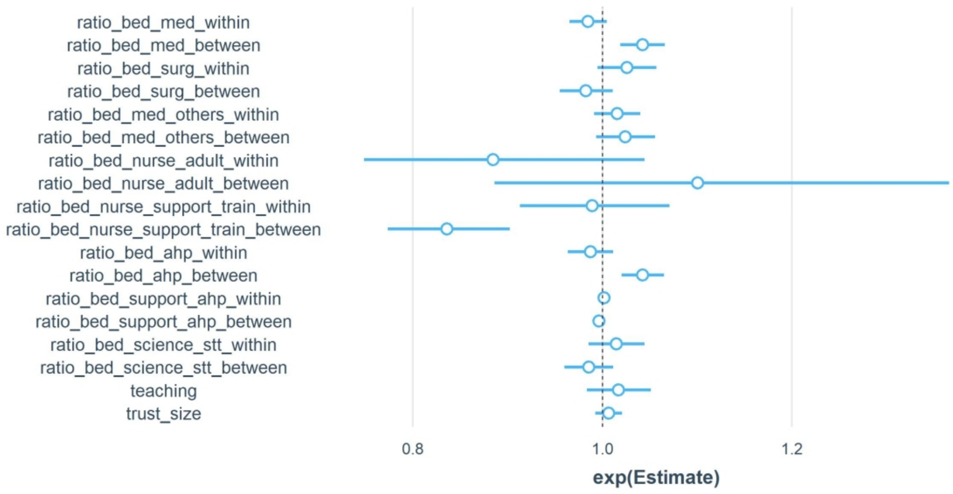

Hospital mortality exponentiated estimates obtained from the within-between random effect model (WBRE).

199x99mm (300 x 300 DPI)

Supporting information

Tables

Table S1. NHS digital data sources

Data source	Datasets	Main variables available
NHS workforce data	Medical dataset	Professionally qualified clinical staff
	Non-medical dataset	Professionally qualified clinical staff
		Support to clinical staff
Bed occupancy data	Overnight beds dataset	Available beds
		Occupied beds
		% occupied beds
	Day only beds datasets	Available beds
		Occupied beds
		% occupied beds
ERIC data		Trust profile (e.g. type)
		Strategies and policies
		Finance
		Safety
		Fire and safety
		Business transport
		Medical records
		Key worker accommodation
		Facilities management services
		Areas
		Function and space
		Quality of buildings
		Combined heat and power energy
		Energy
		Water services
		Waste
SHMI data		SHMI value
		SHMI banding
		Number of spells*
		Observed deaths
		Expected deaths

For peer review only

NHS: National Health Service, ERIC: Estates Returns Information Collection, SHMI: Summary Hospital level Mortality Indicator, * spells: total continuous stay of a patient using a hospital bed at an NHS organisation under the care of one or more consultants, or nursing episode or midwife episode.

Table S2. Comparison of goodness-of-fit for model selection

Model	Correlations for trusts	Model degrees of freedom	AIC	BIC	Likelihood-ratio
**OLS**	No	17	8183.5	8255.8	-4074.7
**Poisson**	No	16	10567.6	10635.7	-5267.8
**Negative binomial**	No	17	6692.6	6764.9	-3329.3
**GLS**	Yes	26	7517.0	7627.6	-3732.5
**GLS exchangeable**	Yes	18	7867.5	7944.0	-3915.7
**GLS Toeplitz (2)**	Yes	19	7823.9	7904.7	-3892.9
**GLS AR**	Yes	18	7822.7	7899.2	-3893.3
**Poisson RE**	Yes	14	1997.8	2057.3	-984.9
**Negative binomial RE**	Yes	14	250.3	309.9	-111.2
**Poisson FE**	Yes (dummy)	146	6477.4	7098.2	-3092.7
**Negative binomial FE**	Yes (dummy)	147	6180.5	6805.6	-2943.3
**Negative binomial WBRE**	Yes	22	6418.3	6511.8	-3187.1

AIC: Akaike information criterion, BIC: Bayesian information criterion, OLS: ordinary least squares, GLS: generalised least squares, AR: autoregressive, RE: random effects, FE: fixed effects, WBRE: within-between random effects, (2) tridiagonal 2-Toeplitz matrix

Table S3. Multicollinearity test in the negative binomial random effects model

Variable	GVIF
Beds per medical	3.1
Beds per surgical	3.6
Beds per other medical	6.1
Beds per nurse	3.7
Beds per nurse support	1.7
Beds per AHP	2.0
Beds per AHP support	1.4

For peer review only

Beds per ST&T	2.6
Teaching status	1.8
Trust size	1.1*

GVIF: generalised variance inflation factor; AHP: allied health professionals; ST&T: scientific, therapeutic, and technical; *reported on GVIF^(1/(2*degrees of freedom)) instead of GVIF as variable has 2 degrees of freedom

Figure S1. Spearman correlation between staffing level variables included in the study.

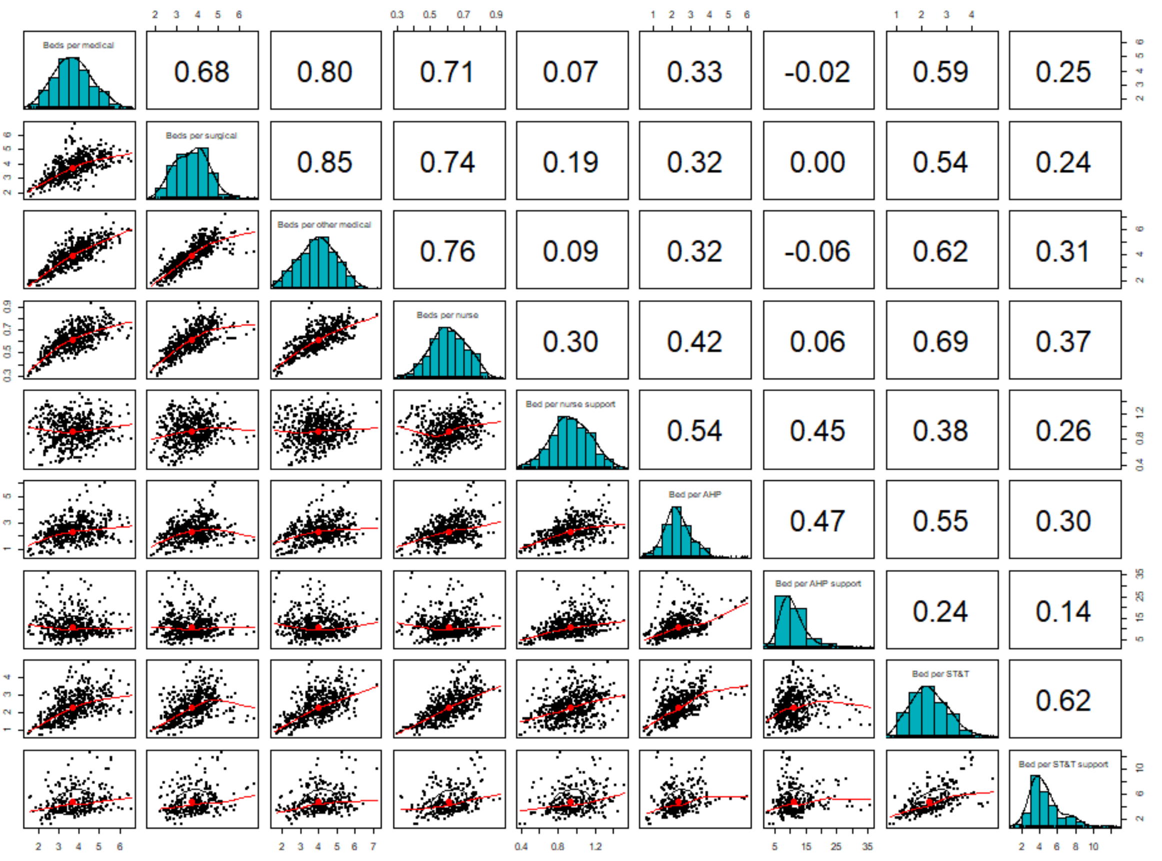

BMJ Open

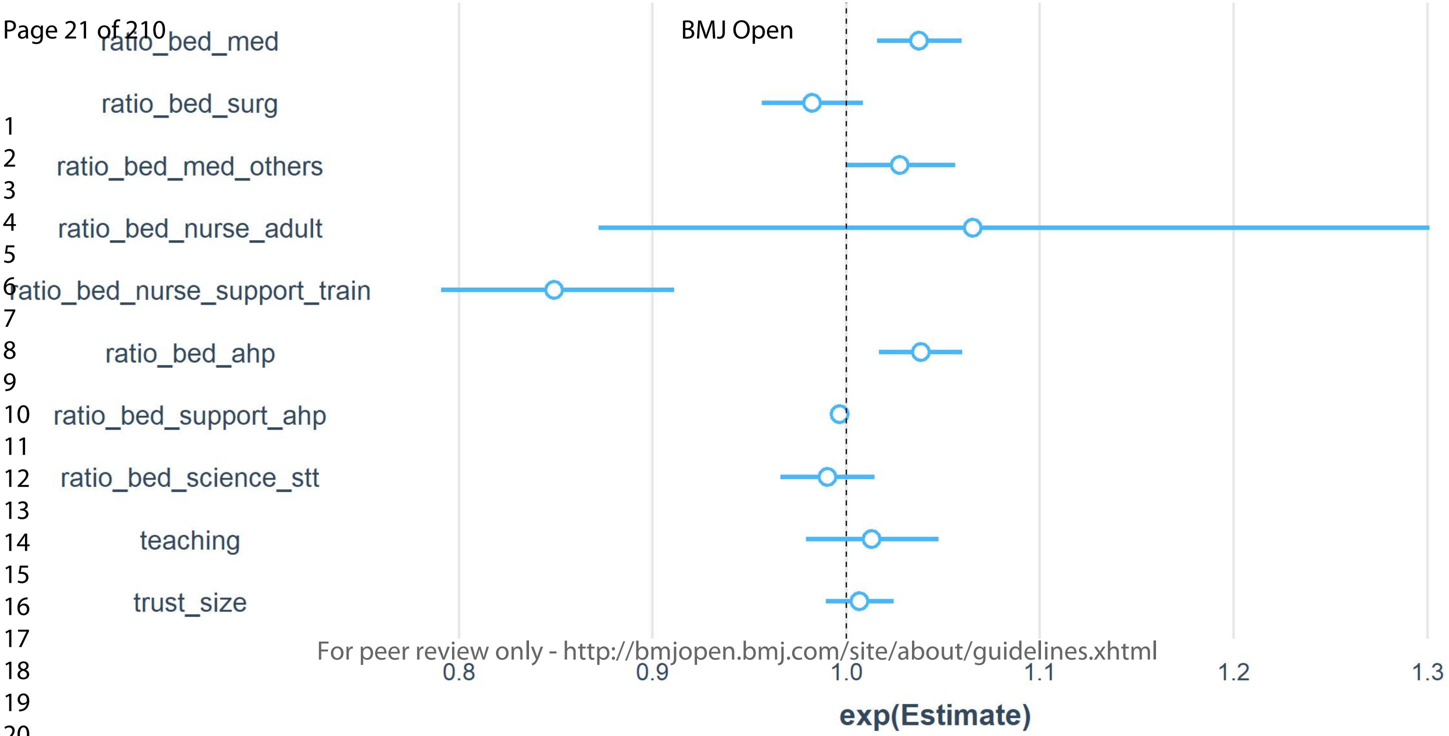

For peer review only - http://bmjopen.bmj.com/site/about/guidelines.xhtml

orgcode	year	wave	aest	onco	dental	em	gen_med	obg
R00	2016	1	56.05	0	8	33.12583	153.2234	29.37652
R00	2017	2	56.18542	0	9.875	35.65656	159.836	28.9803
R00	2018	3	57.43906	0	9.20625	37.83073	157.8064	29.53469
R00	2019	4	63.70833	0	8.5625	36.53271	160.5372	29.55375
R0A	2017	2	302.0568	1	85.00836	121.7625	530.2163	136.3645
R0A	2018	3	320.5534	1.083333	84.99457	129.6084	544.2799	147.3029
R0A	2019	4	345.349	1.25	86.16583	139.2243	575.7461	159.0995
R0B	2018	3	83.886	0	20.85417	56.41459	199.7705	30.38875
R0B	2019	4	85.90921	0	20.91042	61.10417	217.3347	31.26896
R1F	2016	1	23.4015	0	0.95	20.96667	52.04995	13
R1F	2017	2	20.944	0	0.9	20.08333	68.15411	11.5
R1F	2018	3	22.56067	0	0.9	17.93333	72.27033	11.24833
R1F	2019	4	23.31067	0	0.9	20.46667	68.3486	11.24333
R1H	2016	1	306.4837	14.81042	103.8693	182.3187	669.9289	141.6634
R1H	2017	2	331.231	15.55417	104.4089	194.7344	686.235	141.8544
R1H	2018	3	346.1419	18.39167	103.5746	213.5106	715.446	139.8643
R1H	2019	4	370.9894	18.39167	114.7958	213.3492	722.5703	140.9994
R1K	2016	1	118.8167	0.6	42.52334	78.08708	384.4125	66.15151
R1K	2017	2	126.0047	0.6	40.14333	76.13333	411.4606	68.28636
R1K	2018	3	128.8062	0.602083	42.74958	87.68125	425.547	68.16666
R1K	2019	4	133.5969	0.602083	40.2675	108.8089	444.2445	69.725
RA2	2016	1	70.85208	41.9365	28.56989	27.33021	138.7205	31.128
RA2	2017	2	73.22448	42.94622	27.25947	26.60067	138.2988	32.83358
RA2	2018	3	74.5599	42.78729	27.93031	32.76665	133.7064	35.50834
RA2	2019	4	77.61639	42.38624	28.38447	37.88098	139.227	38.74479
RA3	2016	1	18.31667	1	0	13.958	52.66979	6.083334
RA3	2017	2	20.72917	1	0	12.683	54.26667	6.333334
RA3	2018	3	19.04323	0.666667	0	17.608	60.26042	5.25
RA3	2019	4						
RA4	2016	1	36.44667	0	0.058333	19.49167	83.12766	14.66667
RA4	2017	2	38.34875	0	0.1	19.20417	78.98117	15.25
RA4	2018	3	36.955	0	0.025	21.5	83.75765	14.8625
RA4	2019	4	39.34458	0	0	24.16667	83.97288	15.41042
RA7	2016	1	195.405	43.66567	67.84854	36.40053	239.3715	60.14467
RA7	2017	2	197.7366	46.27292	70.55145	41.42104	247.6082	61.42617
RA7	2018	3	200.945	44.91146	73.69695	44.84463	267.9057	64.82542
RA7	2019	4	208.4044	45.70729	78.42262	52.04108	292.9539	69.72591
RA9	2016	1	62.45	3.835417	15.32706	43.94167	124.6981	16.79583
RA9	2017	2	62.30833	2.275	13.6835	37.26563	144.566	19.21406
RA9	2018	3	63.72519	2.895833	14.19167	45.15625	150.9001	21.61615
RA9	2019	4	67.0883	2.991667	16.40833	32.94584	160.0612	23.96354
RAE	2016	1	79.9295	0	22.95378	49.87684	189.1905	41.58021
RAE	2017	2	82.72961	0	21.80191	50.06498	194.1658	38.9275
RAE	2018	3	84.12875	0	25.07212	50.44463	198.6724	39.09687
RAE	2019	4	88.93708	0	25.72378	53.05235	192.2427	41.48938
RAJ	2016	1	58.35833	3.05	8.148487	41.02197	161.758	30.47121
RAJ	2017	2	58.40833	2.666667	6.78182	45.94864	176.8283	32.28787
RAJ	2018	3	62.96875	5.25	7.80682	49.47114	178.2605	32.26222
RAJ	2019	4	77.41807	4.583334	8.93182	58.3026	213.1049	39.06709

For peer review only

RAL	2016	1	200.3969	6.595833	24.3125	78.16137	455.9223	93.30182
RAL	2017	2	209.1594	5.866667	22.00208	77.64116	462.086	90.89093
RAL	2018	3	216.5466	5.416667	22.69375	81.9176	468.5832	94.7331
RAL	2019	4	222.421	6.733333	23.2625	89.01599	472.6579	96.04423
RAP	2016	1	51.875	16.61667	0	47.96349	133.3507	43.5625
RAP	2017	2	53.66667	17.86667	0	50.66778	133.842	48.27292
RAP	2018	3	55.80417	16.74792	0	53.34924	136.9253	45.47813
RAP	2019	4	58.43333	14.61875	0	63.69167	139.454	47.92396
RAS	2016	1	61.25834	0	3	43.725	104.9833	37.83983
RAS	2017	2	60.17083	0	3	41.59167	103.8361	39.54725
RAS	2018	3	59.50938	0	2.686667	45.93958	106.4638	39.949
RAS	2019	4	58.77854	0	2.695	44.85833	114.4132	40.5131
RAX	2016	1	56.23067	2.8	10.3894	32.08458	110.8432	35.13144
RAX	2017	2	56.5686	2.8	11.15531	33.77083	124.1296	37.68229
RAX	2018	3	58.28421	2.8	11.84375	36.92146	129.1772	38.80156
RAX	2019	4	65.93154	2.8	12.37368	46.40677	132.8992	40.06927
RBA	2016	1	63.00671	7.630209	15.18258	24.1083	131.2115	18.61708
RBA	2017	2	63.68482	6.267208	16.23758	21.26042	141.8442	19.69742
RBA	2018	3	66.24634	8.652291	16.60424	24.96269	153.4586	23.92708
RBA	2019	4						
RBD	2016	1	38.825	0.401516	3.633333	25.40833	81.71083	18.94233
RBD	2017	2	40.76944	0.333333	4.041667	28.175	93.00094	18.89492
RBD	2018	3	39.1766	0.666667	3.145	26.84086	92.80698	18.85572
RBD	2019	4	40.41083	1	2.713333	28.41202	92.47287	18.18206
RBK	2016	1	45.68058	0.133333	2.006063	32.00834	110.997	31.2625
RBK	2017	2	45.25475	0.45	2.694698	26.10833	119.2886	32.96667
RBK	2018	3	50.80893	0.4125	3.403409	26.84167	116.0729	31.06875
RBK	2019	4	52.14167	0.1875	3.083333	26.40729	110.2847	34.175
RBL	2016	1	71.74509	0	10.6	44.22424	178.0978	28.69091
RBL	2017	2	68.54583	0	11.20417	43.69924	190.621	30.74091
RBL	2018	3	70.19552	0	11.00417	45.12188	184.7213	31.27997
RBL	2019	4	72.8774	0	11.56667	49.98902	177.2029	29.14716
RBN	2016	1	75.675	0	4.25	46.21125	150.4852	28.64167
RBN	2017	2	75.38541	0.166667	4.8	53.045	155.4005	31.65417
RBN	2018	3	81.04289	0	5.65	55.19066	199.1467	41.11552
RBN	2019	4	77.61486	0.166667	3	58.69119	198.2433	35.15302
RBT	2016	1	38.52917	0	0	27.65458	81.44036	21.2
RBT	2017	2	40.41198	0	0	23.53475	86.85333	21.61719
RBT	2018	3	40.46406	0	0	27.12708	88.1657	22.14167
RBT	2019	4	43.1375	0	0	28.83646	88.45606	24.3
RBZ	2016	1	34.93229	1.65	2.033333	18.59167	67.76807	17.134
RBZ	2017	2	36.45358	1.65	1.783333	17.92708	88.00845	19.453
RBZ	2018	3	41.49588	1.65	5.588367	19.63333	85.87724	19.7895
RBZ	2019	4	46.37004	1.65	7.137251	23.50781	81.69103	20.096
RC1	2016	1	44.24167	7.333334	4.825	24.91667	80.36288	18.3
RC1	2017	2	43.53333	8.4	4.534584	32.32969	85.78125	20.38333
RC1	2018	3	43.94	8.68125	3.7	33.91809	90.44375	21.02344
RC3								
RC9	2016	1	64.65947	0	27.07783	45.23333	141.1459	42.58333
RC9	2017	2	61.36512	0	27.60842	54.44584	147.6735	47.48125

For peer review only

RC9	2018	3	63.329	0	28.48125	50.5375	151.9242	48.97039
RC9	2019	4	78.31475	0.814583	30.50833	56.31469	168.819	51.42994
RCB	2016	1	91.275	11.83333	22.49788	44.86086	204.6702	45.93333
RCB	2017	2	98.6	7.5	22.56815	46.48482	218.2605	42.73333
RCB	2018	3	99.03646	1.25	23.64582	48.90525	233.9064	43.8625
RCB	2019	4	105.9104	1.533333	24.40615	55.914	252.915	44.33542
RCD	2016	1	37.57917	0	1.36591	27.275	87.82261	20.3875
RCD	2017	2	39.37242	0	1.36591	29.41667	92.06877	21.34479
RCD	2018	3	38.46088	0	1.267993	30.96198	92.19634	21.72233
RCD	2019	4	38.92892	0	0.592993	31.94479	93.28851	22.98581
RCF	2016	1	29.17	0	1	22.19448	82.54462	21.90758
RCF	2017	2	28.27983	0	1	24.5974	81.27171	21.36885
RCF	2018	3	30.21371	0	1	25.63646	83.81448	21.15346
RCF	2019	4	29.90267	0	1	27.9599	83.2041	19.40895
RCX	2016	1	50.47933	5.158333	5.873	19.05833	83.5314	17.49383
RCX	2017	2	53.44183	2.845833	5.606333	24.68333	89.31162	24.06193
RCX	2018	3	53.97329	3.504167	5.873	30.75375	109.3997	27.19405
RCX	2019	4	53.6875	3.995833	5.856333	34.16708	123.9652	30.69375
RD1	2016	1	74.375	14.61042	8.70625	47.90271	173.0826	20.36667
RD1	2017	2	75.69583	16.47083	9.066667	37.4825	184.8946	22.59924
RD1	2018	3	76.7524	11.16696	10.185	34.48812	195.746	24.17202
RD1	2019	4	80.23301	10.27265	11.19667	35.86073	195.7948	26.06771
RD3	2016	1	61.54167	16.09167	17.02424	24.14419	123.6139	29.56733
RD3	2017	2	62.31104	15.50208	16.27841	25.56908	129.9648	29.61979
RD3	2018	3	62.76823	16.14375	16.12518	26.97127	131.5882	29.18438
RD3	2019	4	66.88416	17.50833	17.85299	31.13294	135.964	28.16917
RD8	2016	1	47.68333	0.291667	3.458333	27.22713	114.8412	27.98334
RD8	2017	2	47.69167	0	3.366667	32.4938	126.8977	28.94556
RD8	2018	3	47.69584	0	3.27197	37.31463	129.2916	30.01459
RD8	2019	4	48.4125	0	2.383902	36.07713	131.251	28.71667
RDD	2016	1	49.3625	0	3.35	37.45833	191.4276	27.9697
RDD	2017	2	48.80833	0	2.1	43.41667	197.6606	32.575
RDD	2018	3	49.45521	0	2.133333	44.4	199.7161	33.35417
RDD	2019	4						
RDE	2016	1	61.7375	2.258212	3.610603	33.65	112.5378	31.4095
RDE	2017	2	61.27917	6.535417	2.360603	36.41459	106.9778	29.6625
RDE	2018	3	108.0061	29.40636	13.65473	93.28333	187.753	52.60642
RDE	2019	4	129.8947	32.17067	16.46079	115.5766	225.452	63.74796
RDU	2016	1	130.2705	2.333333	8.466666	97.26041	278.4438	69.03333
RDU	2017	2	131.0559	2.5	7.883333	97.2151	292.7857	73.94583
RDU	2018	3	134.5504	2.916667	8.041667	110.012	302.9783	75.3474
RDU	2019	4	134.9448	3	7.783333	116.6828	298.9524	79.94323
RDZ	2016	1	45.85	0	3.219702	32.23727	190.4624	15.02992
RDZ	2017	2	49.91459	0	3.459093	31.99977	201.0549	14.61742
RDZ	2018	3	52.39375	0	3.570077	36.82524	198.5292	13.91951
RDZ	2019	4	55.09317	0	2.86591	44.44018	200.8992	15.4018
RE9	2016	1	26	0	0	16.58333	83.64016	14.93333
RE9	2017	2	25.16667	0	0	16.86667	81.77356	14.96667
RE9	2018	3						
REF	2016	1	86.08	15.78333	14.3597	37.96097	164.3415	28.82092

For peer review only

REF	2017	2	88.815	14.15521	12.82152	43.31717	227.0609	34.455
REF	2018	3	97.79283	12.18021	14.35108	40.70599	212.6059	31.9625
REF	2019	4	102.7426	18.5625	15.46458	37.28071	193.8264	36.51563
REM	2016	1	78.49167	0	23.13228	50.24149	194.7319	0.666667
REM	2017	2	77.82604	0	24.33	42.00303	163.5197	0
REM	2018	3	90.08649	0	27.97167	47.48333	188.1273	1.183333
REM	2019	4	155.8515	0	53.1175	86.84728	340.1711	4.333334
RF4	2016	1	135.7154	15.35	9.591666	56.18333	246.882	55.65
RF4	2017	2	140.1291	15.58333	10.04583	46.4	234.8843	56.48125
RF4	2018	3	137.3853	16.75833	9.025	47.43156	228.6433	56.94452
RF4	2019	4	137.7187	17.16667	8.85	61.115	241.1263	58.74738
RFF	2016	1	37.85161	0	2.866667	33.38921	90.46875	25.67083
RFF	2017	2	35.1303	0	2.45	34.91062	95.44532	25.39583
RFF	2018	3	36.58962	0	2.506792	32.99543	104.5011	24.88333
RFF	2019	4	37.90387	0	1.8813	36.38083	109.6876	26.44583
RFR	2016	1	46.1235	0	15.23125	25.0375	79.39888	25.85337
RFR	2017	2	42.78917	0	16.03479	21.66458	72.20543	25.95704
RFR	2018	3	40.56688	0	16.22173	26.50067	75.4009	24.12661
RFR	2019	4	39.47617	0	15.96457	34.4047	82.30307	25.9964
RFS	2016	1	48.145	0	9.066667	26.3625	92.56084	26.28333
RFS	2017	2	46.895	0	7.2	29.98583	102.145	26.79583
RFS	2018	3	51.345	0	7.400833	29.76583	118.1527	26.85844
RFS	2019	4	57.465	0	6.875834	33.28667	117.7742	29.07365
RGN	2016	1	65.75398	0	14.08333	39.21833	126.9627	27.31667
RGN	2017	2	95.72239	0	15.175	58.19883	193.4043	49.44563
RGN	2018	3	92.86095	2.041667	13.475	65.16155	198.9864	50.92792
RGN	2019	4	93.91649	3.941667	13.26288	72.40466	206.6115	50.4501
RGP	2016	1	41.275	0	1.49727	24.63333	82.22978	18.8
RGP	2017	2	37.88334	0	1.71818	27.275	85.05378	21.25
RGP	2018	3	40.17083	0	2.139538	30.67308	82.85521	20.64323
RGP	2019	4	44.1625	0	2.11923	32.54213	92.33636	22.03142
RGQ	2016	1	59.00367	13.775	14.85151	49.17292	114.1882	24.23042
RGQ	2017	2						
RGQ	2018	3						
RGR	2016	1	44.7625	0	0	20.69924	103.4793	18.94167
RGR	2017	2	45.73854	0	0	24.40909	113.2268	20.60833
RGR	2018	3	46.97031	0	0	23.53542	121.343	21.90625
RGR	2019	4	48.30417	0	0	28.49583	129.3202	24.37917
RGT	2016	1	162.7792	35.64978	21.51167	51.24196	327.2557	43.85517
RGT	2017	2	162.3762	40.9473	22.575	58.01162	359.018	45.73056
RGT	2018	3	179.4107	41.10527	27.93854	62.3079	374.886	47.9035
RGT	2019	4	186.5079	33.9806	26.60649	65.33437	399.2176	51.98479
RH8	2016	1	81.71667	13.49167	16.07458	35.97709	214.7752	26.37275
RH8	2017	2	83.87135	16.54792	15.27156	42.05729	233.2943	31.93629
RH8	2018	3	84.78584	18.61667	15.7849	51.72031	238.2508	31.03894
RH8	2019	4	93.7366	19.26042	15.84792	45.57604	245.5139	35.23629
RHM	2016	1	210.375	25.19375	17.15833	62.57917	362.5858	62.2875
RHM	2017	2	212.35	24.81875	17.69219	62.06562	369.9792	54.92344
RHM	2018	3	219.0417	27.64948	17.575	64.07291	395.7296	51.37448
RHM	2019	4	222.5219	28.73646	18.21042	74.50365	411.6944	51.83958

RHQ	2016	1	166.5333	46.06023	64.87046	54.39469	446.5996	57.58182
RHQ	2017	2	190.4385	44.96966	67.93675	61.15028	469.506	59.14849
RHQ	2018	3	200.1123	46.70816	70.4139	74.75993	486.0018	64.24432
RHQ	2019	4	224.1695	49.80399	76.38525	65.89523	490.2131	64.51515
RHU	2016	1	116.15	31.63125	26.729	43.19584	291.3975	39.94716
RHU	2017	2	117.1259	31.52083	28.87575	48.05521	289.6882	39.90128
RHU	2018	3	114.0091	33.16094	29.23254	49.40437	321.291	40.01177
RHU	2019	4	112.2245	30.52194	27.53546	53.32958	332.5465	41.79825
RHW	2016	1	90.725	21.45833	5.958334	49.00531	182.9681	38.74583
RHW	2017	2	91.90208	19.9	6.308333	49.22027	193.753	40.6125
RHW	2018	3	91.17812	17.33333	6.094584	51.44219	199.7756	39.8427
RHW	2019	4	90.27812	16.75	6.4475	52.86146	192.4432	49.22109
RJ1	2016	1	245.4731	41.00417	91.36166	79.84583	534.1581	87.39243
RJ1	2017	2	254.9398	44.525	96.39523	92.19479	587.3328	93.96117
RJ1	2018	3	281.9419	54.29167	102.0646	106.4766	580.2237	95.31326
RJ1	2019	4	289.1425	57.90625	111.925	100.0344	600.5868	93.74323
RJ2	2016	1	107.2896	0.9	0	69.79375	212.8779	73.49448
RJ2	2017	2	115.3151	0.725	0	77.43125	225.3058	75.77705
RJ2	2018	3	121.7297	0.8875	0	86.69583	231.75	82.89859
RJ2	2019	4	123.1719	1.575	0	91.99937	228.4326	84.11531
RJ6	2016	1	56.42083	0	9.4	36.78542	132.9347	34.46041
RJ6	2017	2	57.10833	0	9.30909	37.65364	140.0358	35.99583
RJ6	2018	3	57.43958	0	10.46402	35.41459	141.3482	39.57865
RJ6	2019	4	60.03646	0	9.917807	41.00469	149.2809	42.05729
RJ7	2016	1	180.8667	2	34.19167	78.99631	355.6003	48.80542
RJ7	2017	2	189.799	2.383333	41.27291	69.73882	379.3764	52.96376
RJ7	2018	3	184.2429	3	42.39917	68.52938	388.4285	52.33363
RJ7	2019	4	179.8208	3	53.19584	73.22548	394.4716	51.90909
RJC	2016	1	43.22892	2.083333	2.09091	26.2	103.9834	21.85983
RJC	2017	2	43.8125	1.833333	1.257577	25.88333	96.98075	20.99042
RJC	2018	3	46.18906	1.666667	1.761743	27.21563	103.7375	22.57548
RJC	2019	4	48.05104	1.666667	2.216667	31.51146	115.4541	23.91662
RJE	2016	1	134.2514	18.90642	22.11381	82.27029	282.441	49.04746
RJE	2017	2	132.6484	17.68333	21.59222	80.63729	306.4102	47.75331
RJE	2018	3	133.8543	18.62972	23.1541	88.99792	322.5295	48.27087
RJE	2019	4	137.8962	19.14827	22.90586	92.09583	336.1371	51.18598
RJF	2016	1	47.56667	2.2	1.350002	23.28333	82.32892	23.57045
RJF	2017	2						
RJF	2018	3						
RJL	2016	1	70.00289	0	0.878783	33.10151	129.5047	44.0088
RJL	2017	2	66.28637	0	0.04545	35.84061	122.1511	46.1471
RJL	2018	3	64.9665	0	0.04545	47.01538	136.5877	47.40052
RJL	2019	4	64.54964	0	0.128783	56.27399	155.5457	46.83692
RJN	2016	1	24.425	0	0.472328	18.96667	71.89438	30.95049
RJN	2017	2	23.45	0	0.50667	20.15313	66.07357	29.03132
RJN	2018	3	24.90833	0	0.097781	24.18125	63.73016	28.33845
RJN	2019	4	25.2	0	0	24.43833	63.85285	21.6875
RJR	2016	1	55.375	0	14.29318	29.25133	110.7431	28.23333
RJR	2017	2	54.67761	0	12.37235	28.76667	111.9412	29.06667
RJR	2018	3	56.56719	0	10.89659	31.02917	115.8739	29.65833

For peer review only

RJR	2019	4	61.88646	0	8.286364	33.58021	115.685	29.89375
RJZ	2016	1	234.0708	2	104.5935	118.1583	509.0765	94.32727
RJZ	2017	2	244.3283	1.416667	105.6794	128.4333	542.2481	97.39584
RJZ	2018	3	268.2871	1.166667	109.1574	135.3614	562.8437	99.10573
RJZ	2019	4	274.4289	2.083333	114.8229	138.3613	570.7501	102.2224
RK5	2016	1	52.32084	0	1.069	28.49565	127.0066	30.45
RK5	2017	2	55.45729	0	1.069	30.72292	140.5839	31.875
RK5	2018	3	55.50604	0	1.06325	37.675	148.7871	33.9099
RK5	2019	4	54.51875	0	1.00575	44	155.724	32.86042
RK9	2016	1	121.7471	22.49333	15.30356	47.38479	197.4907	35.35439
RK9	2017	2	126.5346	22.99563	14.04729	53.20156	247.2842	40.74826
RK9	2018	3	125.4432	26.19792	14.10271	50.70302	240.2687	37.83607
RK9	2019	4	130.2242	25.26542	16.9175	55.62989	249.0176	38.53188
RKB	2016	1	119.9297	18.82803	14.59516	53.85833	263.1738	39.48333
RKB	2017	2	120.9349	19.80094	17.475	62.00104	276.3835	39.12083
RKB	2018	3	127.0432	19.30542	20.2	78.35375	277.4241	41.24219
RKB	2019	4	126.0243	17.16053	19.36667	84.15104	295.4099	42.99167
RKE	2016	1	54.98692	0	0	18.41667	127.1963	39.11065
RKE	2017	2	60.00583	0	0	26.85729	112.089	38.96095
RKE	2018	3	57.30063	0	0	34.97552	104.8696	36.29857
RKE	2019	4	57.35812	0	0	39.36042	107.1449	36.79744
RL4	2016	1	85.02175	13.4125	12.93958	49.93333	234.4717	41.8
RL4	2017	2	89.72824	17.02083	14.9971	51.39917	256.8023	45.08229
RL4	2018	3	95.1983	18.19583	13.95235	55.71312	293.4972	46.61979
RL4	2019	4	101.2841	23.925	14.59667	59.8525	296.9304	52.02438
RLN	2016	1	74.68229	0	20.89924	47.53333	156.8769	24.75833
RLN	2017	2	75.25354	0	21.06667	43.2	154.7685	25.525
RLN	2018	3						
RLQ	2016	1	36.27	0	2.95	14.27667	59.57993	15.5
RLQ	2017	2	38.96938	0	2.866667	17.1975	63.415	16.70833
RLQ	2018	3	40.86636	0	2.108333	18.80917	69.29416	20.68854
RLQ	2019	4	42.62604	0	1.983333	22.73958	76.65526	20.17083
RLT	2016	1	31.08667	0	1.2	12.15	70.0781	20.73788
RLT	2017	2	29.19542	0	1.2	14.7	65.94319	20.65
RLT	2018	3	30.59375	0	1.2	14.85313	67.76146	19.94896
RLT	2019	4	29.41823	0	1.2	13.25833	73.95493	18.53237
RM1	2016	1	93.54583	17.36515	14.25	64.18735	229.5133	49.57659
RM1	2017	2	98.65417	22.00833	14.64773	71.03479	258.6885	52.98576
RM1	2018	3	104.575	22.7625	16.68219	75.75333	267.9467	58.71605
RM1	2019	4	105.0042	23.64167	17.39545	82.26406	284.7962	59.7125
RM2	2016	1	99.20348	0.583333	15.075	47.98188	224.4659	35.85526
RM2	2017	2						
RM3	2016	1	114.1604	0	2.445917	62.19489	279.4623	16.95417
RM3	2017	2	118.1771	0	2.210104	61.33352	308.3342	17.95208
RM3	2018	3	120.8198	0	3.233333	65.14422	326.5655	17.87136
RM3	2019	4	131.5156	0	4.525	68.12704	335.3588	19.75
RMC	2016	1	68.44791	0	1.9755	38.88334	120.0949	42.8375
RMC	2017	2	68.725	0	1.9755	39.82292	121.5635	42.18518
RMC	2018	3	70.83854	0	2.112667	41.46354	115.9706	46.31989
RMC	2019	4	73.06675	0	2.221	40.58096	110.5036	49.714

For peer review only

RMP	2016	1	40.50598	0	3.53	22.16667	81.08167	27.15208
RMP	2017	2	43.26788	0	4.400833	23.81667	79.95424	26.8
RMP	2018	3	44.59872	0	4.22275	28.29292	92.30418	28.93021
RMP	2019	4	43.71247	0	4.362	34.38542	90.18779	31.05313
RN3	2016	1	64.08729	0	6.94012	36.4407	189.1266	26.60145
RN3	2017	2	61.16087	0	6.937145	35.92067	192.742	29.02905
RN3	2018	3	59.87972	0	7.005987	39.4561	205.6559	27.95881
RN3	2019	4	61.7742	0	6.431195	36.75644	223.6947	26.46625
RN5	2016	1	98.53125	7.533333	5.033333	42.05908	178.6222	44.91906
RN5	2017	2	96.64958	9.358334	3.245833	42.49375	185.402	47.42396
RN5	2018	3	99.86906	11.33333	3.0125	41.42083	191.9418	48.81229
RN5	2019	4	100.0865	10.94167	3.1	52.40344	197.0649	48.71198
RN7	2016	1	51.26667	0	0	31.79167	91.09356	33.88334
RN7	2017	2	53.06667	0	0	34.73333	94.76667	34.97709
RN7	2018	3	52.8125	0	0	31.38333	104.1396	35.55
RN7	2019	4	53.93333	0	0	31.02083	109.2458	35.875
RNL	2016	1	48.30625	2.391667	12.01	29.75	88.49917	24.8
RNL	2017	2	52.45833	3.158333	11.09667	31.49167	102.5034	27.2
RNL	2018	3	58.05455	3.559091	13.36364	38.23237	117.9671	35.375
RNL	2019	4						
RNN	2016	1						
RNN	2017	2						
RNN	2018	3						
RNN	2019	4	34.05208	2.475	11.67063	28.51608	89.37759	23.9375
RNQ	2016	1	51.95	0	6.533333	29.16667	118.4063	29.24833
RNQ	2017	2	51.08542	0	6.9	33.925	125.3	33.23333
RNQ	2018	3	51.93542	0.416667	6.091667	39.53959	135.3783	33.71979
RNQ	2019	4	54.9625	1	5.858333	47.61667	139.9085	34.77917
RNS	2016	1	75.14648	10.97588	18.70833	33.025	122.1244	33.91071
RNS	2017	2	78.52179	12.77281	18.625	38.29085	122.0165	34.35646
RNS	2018	3	77.94117	11.09896	17.875	45.25344	133.8442	35.34023
RNS	2019	4	82.01121	11.77917	16.02917	48.05125	145.8263	35.05492
RNZ	2016	1	46.3875	0	9.91136	21.55	79.63724	18.34167
RNZ	2017	2	46.4375	0	9.803026	20.73333	87.93146	21.58333
RNZ	2018	3	49.74948	0	12.63901	23.88073	91.55992	21.39583
RNZ	2019	4	50.0625	0	14.31553	24.77868	92.15708	23.575
RP5	2016	1	78.44318	0	7.8975	40.76875	190.243	30.70833
RP5	2017	2	69.99526	0	7.78675	48.89167	193.325	30.77917
RP5	2018	3	64.90787	0	7.772667	49.3974	203.0541	28.66302
RP5	2019	4	64.58245	0	7.606	53.62083	213.9069	33.30927
RPA	2016	1	62.07711	0	4.633333	37.54167	150.2921	42.33958
RPA	2017	2	64.95825	0	4.5	42.83542	158.7665	39.58333
RPA	2018	3	71.45019	0	3.791667	52.93906	145.9664	39.57708
RPA	2019	4	75.56875	0	3.5	59.67292	129.5933	42.31385
RQ6	2016	1	102.394	0	48.64924	49.00455	302.3664	2.666667
RQ6	2017	2	114.9177	0	51.54091	55.3858	301.1214	1.833333
RQ6	2018	3	111.6865	0	46.98016	57.51091	296.0392	2
RQ6	2019	4						
RQ8	2016	1	111.3839	1.2	11.345	32.03333	125.5748	22.30583
RQ8	2017	2	110.64	1.908333	16.91667	35.3625	131.8054	26.198

For peer review only

RQ8	2018	3	113.7166	0	14.91667	39.11771	131.1163	26.71517
RQ8	2019	4						
RQM	2016	1	117.866	7.158333	7.133333	79.52917	299.0949	93.75338
RQM	2017	2	125.6956	7.533333	6.804167	81.82652	326.0594	95.31879
RQM	2018	3	134.0506	8.454166	6.744167	89.00177	335.8099	98.96886
RQM	2019	4	144.6141	8.116667	6.564167	100.107	350.5117	103.1225
RQQ	2016	1						
RQW	2016	1	53.6625	1.97727	10.26136	54.78959	93.92017	20.5375
RQW	2017	2	52.38438	2.386363	10.26969	56.24792	113.2316	28.59479
RQW	2018	3	48.36875	2.966667	11.04053	44.25156	122.4298	28.04375
RQW	2019	4	51.77083	2.675	11.96761	43.94115	124.8825	30.45417
RQX	2016	1	53.26667	0	6.616667	40.59833	131.3397	52.17437
RQX	2017	2	54.35417	0	5.866667	44.5216	133.1501	53.89166
RQX	2018	3	54.69062	0	6.133333	42.01341	130.5806	59.13588
RQX	2019	4	55.00625	0	5.266667	46.00018	130.454	61.93771
RR1	2016	1	129.3599	1	3	69.92225	401.4728	79.48438
RR1	2017	2						
RR7	2016	1	45.14583	0.008333	0	25.00583	103.2896	25.955
RR7	2017	2	49.10104	0.05625	0	31.65971	103.4667	27.36667
RR7	2018	3	46.39219	0	0	30.17304	118.2253	30.14175
RR7	2019	4	46.85625	0	0	29.3595	129.6694	32.60071
RR8	2016	1	234.6685	26.43736	96.19991	92.86289	499.1016	84.74091
RR8	2017	2	250.1548	33.63136	96.06458	110.8853	505.2443	90.92671
RR8	2018	3	259.1913	35.51458	96.27448	122.229	532.7408	89.33968
RR8	2019	4	270.5147	38.52083	94.17604	119.7005	549.2219	89.84953
RRF	2016	1	63.24924	0	4.210987	24.41667	106.5839	25.325
RRF	2017	2	64.75985	0	4.511153	31.64063	127.2966	26.06667
RRF	2018	3	63.78243	0	4.55082	37.5901	121.1668	30.25312
RRF	2019	4	66.69309	0	4.30082	37.58333	118.1557	32.00521
RRK	2016	1	179.8438	47.95	29.25932	43.6	371.3226	13.67266
RRK	2017	2	196.9408	45	30.87849	49.02852	409.0855	29.78559
RRK	2018	3	320.7498	45.95833	34.39536	122.5691	755.8887	99.57109
RRK	2019	4	333.7638	45.525	34.56724	129.3491	790.5596	95.11091
RRV	2016	1	214.0836	19.21306	96.93443	45.04917	317.0301	81.03849
RRV	2017	2	222.0728	20.83011	100.6694	41.51437	336.4005	91.34104
RRV	2018	3	227.4887	20.6325	104.435	37.85916	363.1531	97.61138
RRV	2019	4	230.421	25.83438	108.459	45.27917	383.0187	107.0713
RTD	2016	1	238.425	32.08333	95.25674	46.18333	374.9288	62.54167
RTD	2017	2	231.5865	30.43333	96.64374	47.92292	384.6585	61.02083
RTD	2018	3	240.0891	31.29583	98.75565	53.88854	380.3898	62.38229
RTD	2019	4	248.7276	30.62917	108.0472	54.31355	402.8938	63.90156
RTE	2016	1	113.6979	19.38433	18.74022	48.88334	228.7802	33.86023
RTE	2017	2	116.5366	17.63367	18.96787	48.65833	241.3423	33.83888
RTE	2018	3	120.9087	17.61642	22.66794	42.31562	256.1603	33.24591
RTE	2019	4	124.0461	19.041	24.40731	51.75365	268.4768	34.90556
RTF	2016	1	74.035	0	10.86591	89.20417	231.8506	29.775
RTF	2017	2	71.02312	0	9.694016	92.80458	225.2101	29.375
RTF	2018	3	66.9508	0	9.362904	83.70734	208.682	26.22292
RTF	2019	4	69.62503	0	10.4679	87.88271	215.7467	28.69115
RTG	2016	1	116.8549	26.45833	24.375	62.39792	236.6034	50.40833

For peer review only

RTG	2017	2	121.0737	30.21667	25.12333	70.53059	251.3338	52.19427
RTG	2018	3	159.9411	32.05833	26.5947	96.82732	329.7264	73.85301
RTG	2019	4	174.6979	33.45	28.80341	104.6206	370.769	82.91331
RTH	2016	1	213.1906	33.67667	28.76152	62.62146	409.3517	65.62917
RTH	2017	2	217.7545	36.031	28.0589	74.84181	402.3894	70.41511
RTH	2018	3	221.435	36.79696	27.00253	78.26046	417.9164	67.31999
RTH	2019	4	225.6746	39.42728	32.14442	85.67573	444.2602	73.30315
RTK	2016	1	67.26766	0	10.55	37.72504	141.3588	28.31785
RTK	2017	2	68.08906	0	10.8365	42.48919	144.1845	32.06453
RTK	2018	3	69.19599	0	11.93775	39.38125	140.4989	33.06802
RTK	2019	4	69.15639	0	12.86667	39.66042	150.0568	36.81771
RTP	2016	1	71.41712	0	10.30426	43.73667	159.4066	35.87636
RTP	2017	2	74.12993	0	10.96967	46.18396	177.9179	35.72879
RTP	2018	3	72.43025	0	10.02898	45.96979	178.3931	36.60005
RTP	2019	4	72.75642	0	9.55967	51.69584	172.7689	37.63334
RTR	2016	1	129.2201	16.35	23.31667	44.25518	236.0802	40.925
RTR	2017	2	130.7539	19.17292	24.50833	40.33911	261.4756	39.32046
RTR	2018	3	135.9714	19.75586	25.57863	39.90674	282.0538	43.07302
RTR	2019	4	135.9697	19.72853	28.73729	41.72517	297.6287	45.35049
RTX	2016	1	54.88396	0	7.619267	34.19584	114.3304	39.10417
RTX	2017	2	57.28158	0	7.819267	33.69167	128.0984	36.563
RTX	2018	3	61.09444	0	7.7643	37.78802	148.2303	37.18727
RTX	2019	4	66.27093	0	9.934067	46.93229	171.2887	41.80883
RVJ	2016	1	144.4375	0	0.583333	39.86167	243.4593	41.35683
RVJ	2017	2	145.0684	0	0	39.43051	248.1377	39.88792
RVJ	2018	3	151.2705	0	0	43.15972	257.3058	39.55625
RVJ	2019	4	156.3935	0	0	47.88363	278.6808	43.14067
RVR	2016	1	89.44242	0	3.2	52.50265	186.1453	50.71883
RVR	2017	2	92.22102	0	3.65	52.85104	208.0873	53.7756
RVR	2018	3	98.83369	0	3.85	59.21907	229.5811	56.85511
RVR	2019	4	101.7924	0	4.066667	67.45959	244.943	59.09225
RVV	2016	1	120.9313	0	22.01667	51.75	299.3833	54.17917
RVV	2017	2	126.5075	0	21.8	45.01875	304.4175	56.83092
RVV	2018	3	133.9389	0	23.45067	55.04115	295.8384	58.74798
RVV	2019	4	150.7578	0	24.91867	67.85416	302.601	62.84054
RVW	2016	1	54.72358	0	0	38.39091	149.4677	30.975
RVW	2017	2	53.62347	0	0.5	38.63258	150.2198	33.37292
RVW	2018	3	56.58648	0	0.473958	38.29775	145.8203	29.99427
RVW	2019	4	53.96198	0	0.5	40.90099	144.2896	34
RVY	2016	1	40.64167	0	1.853333	28.00833	84.97702	26.10417
RVY	2017	2	40.30833	0	2.136667	28.575	83.39462	23.70833
RVY	2018	3	39.23895	0	2.161667	31.38958	82.84895	25.74167
RVY	2019	4	36.49828	0	2.045	33.97917	84.01268	24.72656
RW3	2016	1	223.4638	1	67.54186	75.80417	297.4166	96.91834
RW3	2017	2						
RW6	2016	1	135.1927	0.016667	24.21146	88.89053	276.3285	86.61042
RW6	2017	2	156.2648	0	23.8125	102.9729	277.8135	87.59688
RW6	2018	3	143.9831	0	26.07708	99.72954	257.6224	79.76771
RW6	2019	4	132.476	0	23.90833	102.7585	262.4704	78.55469
RWA	2016	1	120.9383	20.20833	24.57966	50.6	303.33	38.27083

For peer review only

RWA	2017	2	118.7854	12.36146	23.98648	52.52604	312.401	39.79531
RWA	2018	3	98.10304	11.68281	24.85939	53.98802	354.7523	46.80261
RWA	2019	4	104.2738	7.583334	28.71091	55.64667	382.7568	49.23875
RWD	2016	1	119.0986	14.9715	16.20733	53.03333	208.9376	44.16667
RWD	2017	2	123.219	13.90625	16.57412	53.05909	211.6468	44.27917
RWD	2018	3	118.5316	14.67292	18.13236	57.42708	208.6225	47.28233
RWD	2019	4	119.7181	12.0625	14.96843	72.43855	210.5489	46.93047
RWE	2016	1	245.0354	30.88333	18.24167	122.8625	491.6226	123.7167
RWE	2017	2	248.1474	27.25	17.23333	124.4156	519.3574	102.1672
RWE	2018	3	256.1781	29.88958	18.85	127.6141	529.7058	101.9501
RWE	2019	4	271.1443	25.45208	20.1	137.1656	546.1966	100.6295
RWF	2016	1	72.93996	22.36667	0	49.375	190.3531	45.4185
RWF	2017	2	78.93183	17.3625	0	42.82813	203.9954	42.56267
RWF	2018	3	83.41973	18.66125	0	37.78959	208.6493	45.52875
RWF	2019	4	86.19614	20.20521	0	38.36666	207.7078	51.89
RWG	2016	1	62.13617	2.916667	4.933333	33.86666	179.62	36.29546
RWG	2017	2	66.56966	2.583333	4.85	38.66354	202.2466	41.05133
RWG	2018	3	69.21904	2.666667	4.9	40.72686	207.3629	39.49628
RWG	2019	4	67.37233	2	4.9	43.63487	216.0864	41.1589
RWH	2016	1	81.65166	49.91768	1.600187	44.54167	197.0144	38.40583
RWH	2017	2	81.06256	50.1778	1.985603	46.45858	203.1054	38.8625
RWH	2018	3	87.14989	46.75015	1.856815	41.17542	210.4644	41.72075
RWH	2019	4	90.1719	48.08612	1.81136	50.61063	212.9288	45.91858
RWJ	2016	1	56.95833	0	3.3	28.21875	129.7319	25.65833
RWJ	2017	2	58.27483	0	4	33.54375	141.6332	29.30833
RWJ	2018	3	59.54479	0	3.474583	34.84823	144.8705	32.60584
RWJ	2019	4	63.45344	0	3.775	34.56083	147.48	30.97
RWP	2016	1	89.99408	10.22083	18.69583	39.85482	141.4256	42.3554
RWP	2017	2	92.3452	9.920834	17.69583	41.19441	155.4723	48.36969
RWP	2018	3	91.7167	9.655209	19.57396	50.07849	154.9678	46.80088
RWP	2019	4	98.46451	9.519792	19.33019	48.78193	175.4674	48.70453
RWW	2016	1	51.4117	0	3.50682	29.53258	111.8735	27.4185
RWW	2017	2	51.52743	0	3.84432	30.20188	104.8273	24.58802
RWW	2018	3	53.40263	0.083333	4.052653	31.11689	103.6463	27.33738
RWW	2019	4	59.32259	0.583333	4.48182	29.0151	106.5459	28.81346
RWY	2016	1	67.35416	1.083333	2.854167	33.89238	154.8838	37.44375
RWY	2017	2	70.90104	0	3.193667	31.20938	169.9258	37.55625
RWY	2018	3	71.80561	0	3.249117	41.29448	174.5843	40.03021
RWY	2019	4	70.56678	0	2.92355	49.05667	181.5638	41.025
RX1	2016	1	224.0996	35.63958	30.125	58.10333	431.5653	98.58542
RX1	2017	2	232.3559	41.14687	35.28542	60.09323	459.3053	99.16458
RX1	2018	3	242.4742	43.74688	32.00302	66.70873	469.4211	106.8068
RX1	2019	4	250.2624	46.74521	34.76584	73.13788	500.6596	115.0995
RXC	2016	1	74.70834	2.083333	12.39015	35.519	149.7908	32.05679
RXC	2017	2	76.85	2.166667	11.91667	41.20052	161.328	33.82027
RXC	2018	3	77.63561	2.15	11.23333	49.13	171.8957	31.24942
RXC	2019	4	80.47319	3.2	11.72083	53.30792	180.134	32.33698
RXF	2016	1	82.59409	0	16.6	62.15	211.1439	44.71224
RXF	2017	2	84.70116	0	15.31458	60.64375	222.3618	45.85942
RXF	2018	3	88.43862	0.083333	17.44167	63.54896	233.2315	49.70208

For peer review only

RXF	2019	4	90.06355	0.666667	18.83167	64.51458	225.7402	52.91557
RXH	2016	1	146.444	27.44167	17.09583	81.72166	354.2038	53.80306
RXH	2017	2	154.9571	26.39675	16.55867	95.06058	353.6995	53.95031
RXH	2018	3	151.3433	28.49758	15.02667	101.2198	342.647	51.89296
RXH	2019	4	156.1558	29.19133	15.60667	105.4219	333.9421	52.05042
RXK	2016	1	85.1125	0	0.1	52.50834	219.2018	54.25
RXK	2017	2	86.4625	0	0.1	56.32854	216.2653	50.50469
RXK	2018	3	85.14427	0	0.075	63.36979	213.4981	51.58646
RXK	2019	4	90.17709	0	0	78.39479	229.1572	51.71198
RXL	2016	1	64.69167	0	1.291667	36.71312	153.606	31.0495
RXL	2017	2	62.85938	0	2.083333	39.96458	157.9049	31.06567
RXL	2018	3	64.52802	0	3.961668	45.46042	174.5514	30.17725
RXL	2019	4	69.1125	0.083333	5.023337	50.21875	172.5288	30.6225
RXN	2016	1	120.3333	22.33333	18.34667	45.59083	200.3276	33.2
RXN	2017	2	126.6277	26.31617	19.78889	52.84396	192.0099	36.06354
RXN	2018	3	134.6108	29.66108	18.92986	64.51511	185.0823	35.65833
RXN	2019	4	141.3852	30.49742	17.43473	63.22298	202.3992	36.05833
RXP	2016	1	88.28385	0	1.7	50.72833	207.4805	44.40879
RXP	2017	2	88.93864	0	1.745833	45.3925	204.172	45.27362
RXP	2018	3	89.64532	0	2.654389	50.99896	207.2259	52.3599
RXP	2019	4	89.90421	0	8.371779	53.12083	210.1679	57.10625
RXQ	2016	1	83.84087	4.433333	8.231063	34.14	183.3661	41.42083
RXQ	2017	2	85.38743	4.033333	9.18177	34.50834	188.7792	42.93222
RXQ	2018	3	91.6507	3.37815	8.833942	36.62935	185.1588	43.12421
RXQ	2019	4	95.09125	1.632817	9.253166	43.67927	187.2656	48.14726
RXR	2016	1	92.12292	2	20.46515	49.73333	165.2967	53
RXR	2017	2	95.25052	2.666667	21.24015	46.55833	174.936	51.55521
RXR	2018	3	94.89452	1	21.54716	55.21323	189.8661	52.96262
RXR	2019	4	96.22781	0	22.38902	67.45448	201.9952	55.05052
RXW	2016	1	77.01334	12.725	13.95845	35.6	127.9044	35.21667
RXW	2017	2	73.89667	12.25	13.45533	34.44688	132.2797	35.25
RXW	2018	3	76.37951	12.26667	13.49595	43.78333	142.9065	36.46442
RXW	2019	4	76.5279	12.61667	14.5897	57.3375	160.7818	39.09506
RYJ	2016	1	210.1444	10.75417	1.091667	126.2701	545.058	99.15374
RYJ	2017	2	219.5588	9.550756	1.158333	118.9013	562.9307	105.974
RYJ	2018	3	246.4839	11.33636	0.947917	100.1198	562.6566	110.4535
RYJ	2019	4	254.5548	13.58333	0.683333	99.26283	548.2219	116.8041
RYR	2016	1	92.42992	4.033333	21.14849	46.6	238.611	57.72
RYR	2017	2	97.03573	1.666667	20.65682	46.25834	238.5493	56.5575
RYR	2018	3	97.10584	0	19.98315	56.2499	226.58	57.06646
RYR	2019	4	98.2076	0	21.66458	61.06521	220.5903	58.60938

For peer review only

paeds	path	phm_chs	psychiatryg	radio	surgical	availableto	availablege	availablelea
31.65313	24.33333	0.015152	2.833333	18.04167	120.3903	917	871	0
32.48438	21.81667	0	2.666667	17.5375	120.4667	856.25	812.75	0
33.76042	18.03333	0	3	20.06365	121.9198	801.25	789.25	33
32.24844	14.8	0	3	20.76104	126.25	822.25	772.75	0
213.662	112.4238	26.13306	28.99792	92.49167	554.8575	2214	2042.5	0
217.3627	117.5304	23.20166	30.42865	89.04417	583.5899	2271.5	2135.5	36.5
235.6098	116.6691	11.49167	28.46979	91.06406	626.7385	2279.25	2113.75	0
52.2125	15.5625	11.03151	9.933333	27.0625	168.8923			
55.27396	16.12708	10.92111	10.3125	28.46458	180.4101	1135.5	1081.75	3.75
11.994	5.483333	2.433562	19.75	6.16725	48.38334	378.5	298.5	0
10.17292	8.075	1.778155	16.225	5.566667	50.01667	355.75	282.75	0
12.84792	8.541667	1.053383	16.34167	5.970833	51.79167	337.75	282	9.5
15.23333	8.183333	1.016783	14.73333	4.945833	50.42083	330.75	272.5	0
183.2802	89.62813	22.51905	17.68333	99.62417	430.0833	2169.25	1961.25	0
180.2698	92.05	8.15	12.36042	106.6563	441.9174	2043.25	1831.25	0
191.9912	93.21094	5.203333	15.16667	110.0672	469.334	1976.25	1826.25	56.25
211.99	94.92136	2.72	17.15	114.1315	477.153	2035.25	1829.75	0
87.45417	45.19773	14.42833	11.83333	72.43601	206.9144	1240.75	1171.75	0
81.76667	44.25	4.933333	10	75.89359	210.2064	1394.5	1324	0
84.70052	41.87519	2.675	9.7	75.34628	216.365	1373	1336.25	31.25
79.31094	40.04588	2.275	10.18333	76.51667	223.9691	1394.25	1326.25	0
24.05833	26.81667	0	3	15.27317	123.7195	595	535.25	0
23.77396	26.88333	0	3	15.805	122.4643	508.25	446.25	0
25.59583	27.46667	0.083333	2.9	17.90917	126.6174	506.5	447.75	3.25
26.1875	29.66667	0	3	18.08417	135.1841	523	461	0
5.85	2.916667	0	5.008333	4.5	47.57917	288.75	288.75	0
5	4	0	4.491667	5.9	52.39583	269.75	269.75	0
3.233333	3.666667	0	3.875	6.591667	53.48333	263.5	266.5	3
						272.25	272.25	0
15.70833	3.866667	0	0.333333	8.005553	60.15417	398.75	375.75	0
16.60833	3.6	0	2.333333	8.236107	60.05833	407	384	0
17.00885	3.875	0	2.333333	8.92037	58.18333	388.5	378	12.5
17.25781	4.233333	0	2.416667	8.933333	64.25	411.5	387.5	0
123.2883	34.61666	10.2987	6.583334	64.29583	211.9466	955.5	879.25	0
120.2281	36.34306	10.84322	7.55	69.33334	213.3238	950.25	874.75	0
130.5382	33.65701	10.51187	8.469271	71.47904	215.7289	925.25	886	35
132.3325	34.69552	6.494444	8.61875	76.40804	231.0748	968.75	892.75	0
25.95833	13.27333	6.230303	2.816667	14.52167	86.84099	428.75	399.75	0
26.83552	11.46583	5.502779	5.033333	16.06167	89.42849	388	368	0
32.17698	12.02354	5.205558	4.254687	16.46604	93.56755	398.25	378.25	0
31.77344	15.73292	5.583337	2.733333	18.59583	106.0623	409.25	389.25	0
59.6825	23.55833	1.95455	0	21.19167	147.139	815.25	752.5	0
60.4939	22.26875	1.871216	0	21.79167	144.8987	844	791.75	0
62.6354	22.25625	0.310607	2.565104	22.64375	144.2672	787.25	751.25	25.75
64.62996	21.01354	0	4.558333	23.89167	149.8511	769.25	693.25	0
32.95779	11.16667	0	0	17.90833	122.4424	583.25	556.5	0
35.38844	12	0	2.75	19.68333	124.6674	599	568	0
38.74844	14.78333	0	6.916667	21.04583	133.8949	598	574.25	7.25
44.18715	17.40976	0	6.95	26.03183	164.3195	609	578	0

For peer review only

100.3181	101.7426	0	13.57292	70.93143	301.8864	1159.25	1075.75	0
101.0818	106.2378	0	15.91458	72.42424	301.9987	1132.5	1047.5	1.5
99.59173	106.3475	0	16.61667	75.07272	304.2069	1192.75	1135.5	33.25
104.0171	109.4372	0	16.36667	74.33167	315.5378	1231.5	1135.75	0
48.75834	21.95833	1	6.583334	10	92.69692	536.75	487.75	0
49.98542	22.075	1	6.766667	10.08333	92.80101	592.5	543.5	0
57.17417	21.58542	1	7.833334	11.14167	96.63409	570.5	540.5	19
54.32281	18.06875	0.916667	6.583334	11.78125	96.11742	589	545.5	0
37.71788	14.28308	0	3.733333	15.0325	83.28659	586.5	525.75	0
41.02687	10.21633	0	4.2	15.62	86.73458	577.25	508.75	0
41.92292	9.716666	0	3.883333	14.13542	90.02417	549.75	500.25	21.25
43.39531	9.558333	0	2.733333	14.7625	90.2325	555.75	484.75	0
34.22917	14.96667	0	4.666667	17.36458	99.62544	505.75	447	0
35.30312	15.3	0.333333	4.85	18.53333	99.54023	505.25	445.75	0
37.21	15.56458	1	4.316667	18.1375	102.6124	466.25	423.75	14.75
43.10156	16.45042	1	5.191667	17.93333	109.2893	490.75	431.75	0
29.28713	21.525	0	1.833333	15.76	120.92	577.75	537	0
30.53032	22.74167	0	2.5	15.79167	117.7387	570.75	531.5	0
32.71431	23.67667	0	1.75	16.76542	125.8759	661.75	643.75	20.75
						696.25	651.5	0
24.15	10.50833	0	3.416667	12.01667	78.40089	377	342	0
24.10938	10.54219	0	3.833333	10.98344	74.57463	366.75	333.25	0
23.74167	10.54844	0	3.916667	11.51805	78.86263	350	328.5	9.5
25.76719	12.55799	0	4.25	12.64522	79.49796	368	337	0
36.33115	12.325	23.21042	0.666667	5.75	78.40434	556.5	523.75	0
40.00636	12.78333	6.677084	0.666667	5.408333	76.81362	527.25	495	0
41.28583	9.919167	0	0.583333	6.283333	80.27159	508.25	483.25	5.5
41.44003	6.3675	0	0.166667	6.866667	84.81667	529.75	499	0
42.7	21.2	1	0	26.1375	125.6985	795	763	0
41.37917	21.45	0.25	1	26.15	124.7568	812.5	780.5	0
45.30656	20.6625	0	3.083333	27.73854	125.7268	824.75	803.5	10.75
52.40531	19.93333	0	2.633333	27.96042	124.5157	824.75	792.75	0
35.31667	27.00682	22.34167	5.25	22.45	156.335	754.25	717.25	0
38.46667	27.75	49.51042	5.666667	22.5	166.3035	760.75	724	0
45.26812	32.49166	56.7349	6.883333	24.27146	179.4842	751.5	726.75	12
40.25573	29.3	41.1	3.554688	22.70542	172.7446	769.25	732.25	0
28.35	5.808333	0	0.333333	11.63333	91.5875	617	590	0
30.30261	5.016667	0	0	12.2	90.98333	590.25	563.25	0
29.79896	6.5	0	0.333333	13.45833	95.22188	583.75	555.5	7.75
31.04792	4.916667	0	0.833333	13.91667	99.68958	576	540	0
16.44813	10.175	7.132501	0	5.24	67.00042	336	322	0
19.93844	10.12083	4.95	0	5.760833	64.94218	332.5	318.25	0
19.5025	9.552083	1.025	0.083333	6.619167	68.54896	323	322.75	12.75
20.1275	9.3	0	2.166667	7.583334	74.03333	346	333	0
18.8875	9.570833	0	3.833333	10.68333	79.21667	432.75	404	0
21.42917	11.00417	0	4.25	10.85	83.3	435.25	411.5	0
21.94448	10.05417	0	3.416667	10.83333	88.59324	432.75	423	7.5
59.86013	14.167	0.583333	4	20.525	106.5616	722.75	642.25	0
63.8139	15.47283	0.916667	5.25	22.50704	119.8848	771.75	690.75	0

For peer review only

65.52142	17.039	0.833333	4.083334	20.44648	122.1037	758.5	694	16
68.74581	18.14	0.083333	4.552084	23.25817	142.4112	784.25	704.25	0
48.075	20.0875	0	9.25	25.24167	183.6455	1255	1200.25	0
47.12083	23.40417	0	8.166667	24.22917	178.4517	1238.75	1189.5	0
52.99635	24.93333	0	9.216666	24.06458	179.4942	1147.5	1145.25	49.5
53.19479	24.25833	0	9.25	23.46458	192.4375	1216.5	1164.5	0
21.84583	14.89325	13.72473	1.241667	8.583333	68.96451	359	331	0
23.19656	14.54617	13.44584	1	8.75	71.35663	356.25	328.25	0
23.45794	15.80846	11.71609	1	8.935416	71.42501	340.75	319.75	6.25
25.14946	16.82388	11.42556	1	9.475	73.85301	360.5	332.5	0
18.02917	10.23333	0.013638	3.166667	8.925	45.52725	424.25	384.25	0
19.45729	8.633333	0	3	8.7	48.67792	432.75	392.75	0
19.97812	8.258333	0	4.216667	8.902083	51.71302	366.75	339.5	12.75
19.07708	5.083334	0	5.004167	7.960417	54.88177	411.75	373	0
23.39965	3.485417	0	4.916667	9.04	81.43542	569.5	540.75	0
25.88712	2.666667	0	4.666667	9.195833	84.05104	590.75	562.5	0
29.11618	3.416667	0	4.083334	10.91875	89.83229	568.5	568.5	26.75
32.92327	3.916667	0	4.166667	10.14583	92.11667	616.25	586.25	0
29.04792	19.8375	0	2.166667	18.34242	116.2308	718.5	698	0
30.20852	20.81375	0	5.75	19.89659	115.6679	715	692.5	0
31.52694	23.49625	0	5.75	21.28416	123.093	687	678.5	15
32.8495	23.28708	0	6.083334	21.57848	122.8585	700.25	673	0
39.74925	17.14	3.625	5.583334	16.1375	69.81341	660.25	598.75	0
40.06771	18.23167	3.816667	4.833334	15.27083	69.27983	653	593	0
38.305	18.06917	4.825	4.666667	15	66.19635	617.75	580.75	22.5
44.05531	18.415	1.266667	5.5	16.025	73.1956	653.25	593.75	0
30.42222	15.93333	0	0	17.2	93.1	576.25	522.75	0
29.825	16.58958	0	0	18.89167	96.29166	562.75	509.75	0
38.75625	16.604	0	1.083333	20.29479	90.84973	598.75	565.5	19.75
36.59375	17.17867	0	0.333333	19.92188	95.21709	621.5	568.5	0
31.97583	16.75	0	6	25.58333	129.2349	745.25	691.25	0
36.59458	19.41667	0	6.666667	26.06667	137.4561	777.75	723.5	0
37.79979	18.71667	0	7.083334	25.825	137.8215	753.25	718.25	20
						789	734	0
31.83333	14.4875	7.470667	6.033333	16.82439	130.1211	701.75	667.75	0
35.48073	14.00833	9.829001	4.283333	16.55019	131.5377	690	656	0
63.80642	28.36698	4.6175	5.833334	26.94614	237.2485	1219.75	1193.75	36.5
72.77383	32.97479	3.333333	5.083334	33.42235	274.8694	1390.5	1318.5	0
78.5	40.48409	0	8.966666	40.99791	250.9667	1483.75	1411.5	0
76.32917	39.47652	0	10.07917	40.57784	257.4847	1484	1408.75	0
88.29636	33.67841	0	9.256771	43.17159	264.6255	1459	1414.75	29.5
86.67604	33.55568	0	10.53177	42.17159	281.1451	1492	1415.5	0
0	20.905	0	4	16.83333	99.8775	715.25	712.25	0
0.916667	19.15542	0	3.5	17.34583	104.4676	708.75	705.75	0
1	21.81181	0	3.916667	18.4375	108.0453	674.75	708	36.25
1	22.04225	0	3.666667	17.85417	118.6891	721.5	718.5	0
12.75833	1	11.88626	3.833333	7.283333	33.03333	391.25	360.25	7
12.85	1	12.32692	4.166667	8.516666	35.75	393	362	7
						380	362.5	19.5
44.32084	24.56833	0.333333	5.583334	24.23333	137.0598	869.75	824.75	0

For peer review only

47.51967	26.59333	0	2.25	24.15	138.8728	845.75	799.5	0
49.36456	26.24646	0	7.602083	25.375	147.2914	820.75	795.25	23.5
51.06414	25.73083	0	7.354167	26.69167	153.9219	841.5	792.5	0
0	12.48333	0	2.633333	32.9	157.0287	842.75	842.75	0
0.083333	9.775	0	14.75	34.96667	185.1469	879.75	879.75	0
1	14.9625	0.374243	10.5875	41.92135	206.5139	905.25	938.25	33
3.083333	50.15417	2.361364	9.911041	74.80104	314.4003	1404.75	1404.75	0
64.93833	30.23333	0	5.833334	34.10792	202.4504	1059.75	994.5	0
60.83	28.69167	0	6.083334	33.48233	198.4315	1060.25	998.25	0
63.78342	30.0401	0	8.3	28.31525	209.1766	1038	996.25	20.5
67.23115	31.375	0	7.920729	29.775	222.1883	1072.5	1010.25	0
33.075	13.72502	0	2	8.293758	67.14868	479.75	428.5	0
32.365	14.58333	0	2	7.747525	73.28857	472.5	425.25	0
29.86583	13.08906	0.533333	2.266667	7.049175	75.787	486.75	465	19.5
38.75	11.80104	0.8	3.733333	7.25	75.55833	509.5	470.5	0
25.699	9.645833	13.56796	6.25	6.216667	88.87746	555.75	532	0
25.44121	8.960417	13.94162	6.333334	6.466667	87.70664	555.25	531.25	0
19.84928	10.15417	12.16318	7.166667	8.708333	85.26659	508.25	508.75	24.25
28.53016	12.8	9.976664	8.333333	9.4	85.36218	508.5	484.5	0
33.36666	11.35083	0	11.46667	11.30667	76.60333	527	496	0
36.835	14.465	0	9.533334	11.22333	84.12042	517	486.75	0
36.90667	12.34667	0	9.233334	11.96167	89.6475	522.75	491.75	0
37.34344	8.600833	0	9.6	12.5025	87.3675	553.25	522	0
31.43333	13.30083	0.583333	4.25	10.43125	122.0144	726	659	0
35.05448	16.64333	0.666667	5.583334	16.26698	188.4999	989.25	885.75	0
39.43594	18.36333	5.530729	5.216667	17.84881	193.0204	991.75	914.25	26.75
55.43021	20.25875	3.599479	5.25	17.21667	196.1014	1135.75	1026.75	0
18.06667	8.85	3.266667	0	8.5	74.75833	532	490.25	0
20.43073	8	2.233333	2.097917	8.666667	75.75833	553.75	511.75	0
22.1	5.964063	1.65	1.75	9.25	76.73906	511.75	490.5	18
22.475	6.8125	1.05	1.916667	8.333333	79.80417	539.5	501.5	0
26.73925	19.01458	1.333333	3.166667	11.85833	123.1504	678.75	640.75	0
						703.75	665.75	0
						744	706	0
21.00417	14.29375	8.266666	3.333333	11.44281	84.962	492.25	451	0
26.54635	14.50833	9.470834	3.166667	11.97677	88.48008	495	455	0
30.87969	16.075	8.741667	3	11.95875	93.79286	464	428.75	0
32.87969	17.29583	7.3375	3.666667	13.19417	96.59871	474.5	430.5	0
101.451	88.17641	0.333333	5.666667	64.74986	275.1983	1230	1172.25	0
106.5898	93.86967	0	6	69.65408	287.4764	1215.25	1160.5	0
117.5852	104.429	0	6.5	70.74823	297.3232	1152.75	1144.75	47.75
121.841	102.9972	0	6.747917	73.18214	319.6253	1192.5	1135.5	0
38.4425	29.50625	0.704042	0	17.62667	157.3317	1022.75	972.75	0
40.15823	31.51042	1.166668	3.6	16.71708	166.2487	1037	986.75	0
48.13208	33.82865	0.716667	3.941667	18.73917	172.6841	956	950.5	42.5
45.18261	34.10521	0	4.316667	21.77167	181.4175	998	950	0
140.4729	62.66667	0	6.866667	76.01667	311.4688	1255.25	1189.75	0
160.1595	66.82604	0	8.05	77.90833	304.151	1252.25	1188.25	0
170.8674	73.73438	0	10.5	84.33281	308.5443	1245	1205	23
182.975	76.80261	0	14.44479	88.10208	313.9958	1228.25	1172	0

32.74166	84.34805	12.76804	1	72.2125	320.4985	1802.75	1715.25	0
28.4	84.4465	12.643	3.333333	73.93229	334.6927	1774.75	1691.25	0
19.79583	93.25083	11.32323	4.333334	77.48802	342.789	1663.75	1637.75	55.5
36.84583	97.32377	5.597673	3.333333	81.43073	342.0807	1635.5	1551.75	0
50.75927	35.38958	1	4.25	41.63725	182.7553	1116.25	1065	0
50.20573	36.24166	1	4.583334	43.68934	187.6281	1102.75	1054.5	0
50.31904	35.81875	1.333333	4.863646	44.38725	185.316	1073.25	1043.75	16.75
49.34848	34.43833	0.666667	7.343958	41.96225	194.4429	1103	1056.5	0
48.34167	27.3	1	7.25	19.35542	157.6458	838.75	791	0
47.29281	26.13333	1.041667	6.383333	20.37	158.2	807.75	758.5	0
48.25396	24.46667	1.133333	5.35	19.05875	158.925	747.75	733	32.5
48.14854	25.0125	1.383333	7.25	18.68375	165.5942	734	677.75	0
218.2375	74.87917	32.175	0	65.63333	322.7277	1221.25	1123.25	0
240.905	78.59167	30.18333	0	77.0575	339.8389	1236	1138	0
260.631	79.425	29.20833	0	89.13709	367.3954	1239.25	1178.25	37
290.5313	85.5625	13.75833	0	95.33229	372.0698	1304.25	1206.25	0
97.22166	32.4	0	8.583333	18.22917	134.1201	1069.5	976	0
102.0541	34.09167	0.666667	9.333333	15.875	146.0945	1055.5	961.5	0
103.8179	33.80812	0.833333	11.05	15.9125	149.6469	1020.75	949	22.25
106.6589	34.62989	1	11.01667	20.2	150.7623	1085.25	991.25	0
42.475	13.43333	0	0	15.41667	82.76458	562.5	501.5	0
46.1661	12.03333	0.1	0.5	20	87.23542	579	518	0
50.50396	11.55833	0.233333	0.666667	20.11667	96.33805	555	503.75	9.75
49.03594	12.3	0	1.166667	19.025	95.84097	560.25	499.25	0
100.3	60.3125	8.560439	7.716667	79.225	228.4269	1033	970	0
105.6545	53.15104	7.65	12.49167	79.05052	236.3396	1035.75	969.75	0
111.2872	53.07552	3.231771	15.4474	84.35833	252.7425	959.25	912.25	20
115.7978	47.44792	0	13.0625	86.93229	267.5015	997	928.25	0
31.12667	11.44583	0	0	10.35	70.76969	580.75	540.75	0
31.37292	11.6	0	0	10.1	75.75198	571	531	0
31.32875	12.68333	0	0	8.178021	80.24757	542.25	520.75	18.5
33.33387	12.85	0	0	11.78365	82.50394	571.5	531.5	0
80.18479	51.11808	6.166667	5.083334	58.74508	256.3202	1486.25	1424.25	0
77.33223	53.30179	3.666667	5	60.17179	259.3467	1552	1488	0
80.21732	53.11463	2.5	4.393229	67.31185	270.3792	1552.75	1530.5	41.75
83.85431	53.29175	2.333333	4.894792	71.12647	280.4277	1623.5	1559.25	0
23.475	5.583334	0	5.916667	6.470833	81.84091	560.5	517.25	0
						442.25	422	0
						396	380	0
47.75314	7.779192	6.166667	8.416667	13.02967	129.1312	904.5	833.25	0
50.78592	7.658033	6.233333	7.333334	10.11746	125.4427	889	818	0
53.07028	7.3997	6.066667	8.066667	9.927292	134.5837	846.5	790.75	14.5
53.76934	7.033033	6.4	10.01667	10.07425	150.511	829.75	759.5	0
20.46667	3.55	3.06	0.333333	12.13333	47.95114	400	372	0
20.37292	4.316667	3.268333	0	11.48333	48.21364	383.25	355.25	0
18.97969	4.3375	3.31	0.333333	11.19167	49.65038	373.75	356.5	10.5
19.92552	3.970833	2.826667	0.583333	11.46667	50.74167	387.5	359.5	0
29.83122	15.95833	0	0.666667	20.06667	103.5826	573.75	511.75	0
29.76455	15.85833	0	2	20.88125	109.5845	588.5	530	0
32.50414	14.91667	0	2.333333	20.97708	112.5519	555	503.75	3.75

For peer review only

32.16768	14.91667	0	2	23.04781	121.7157	538.25	485.75	0
145.0734	126.8686	35.90445	8	74.28125	396.4744	1557.25	1437.25	0
157.9318	135.15	31.81666	9.25	62.55625	393.2777	1623.5	1504.5	0
161.6266	132.8203	31.36667	7.808333	77.27292	405.4603	1542.5	1474	50
165.7266	137.2666	23.35	0.833333	84.6	424.4386	1637.75	1519.25	0
39.10052	12.35	1.666667	3.916667	9.883333	101.6125	692.75	644.75	0
39.75369	14.46667	1.666667	4.166667	12.25833	106.1	680.75	632.75	0
38.83969	14.15	2	4.916667	14.55833	107.7759	696.5	661.25	12.5
39.62292	14.08333	2	4.166667	12.45	116.2839	702.75	654.75	0
47.46417	43.055	0	2.233333	93.75	229.4333	1006.75	943.75	0
53.12177	46.59667	0	3.766667	94.40479	227.7619	997	936	0
56.95021	45.73509	0	4.936458	95.13917	231.5461	969	929	18.5
59.2525	49.53192	0	6.322917	96.70677	246.7976	1006	945	0
51.94683	37.89333	0.916667	9.641666	35.80416	248.4383	1231	1161.25	0
51.60406	37.73416	0.666667	8.183333	39.72083	261.2442	1225.5	1155.5	0
54.79562	39.07325	1	8.828646	45.32084	262.7731	1207.25	1157.5	20.25
59.43198	38.8979	1	9.400521	49.00417	271.128	1239.75	1169.75	0
58.57331	10.69167	30.76608	8.466666	18.27576	71.61094	388.5	325.25	0
56.48114	12.76667	35.50478	12.03333	17.63617	67.21467	386.5	323.25	0
57.88802	14.55	34.13722	11.35	19.84659	70.9568	387	333.5	11.75
59.00573	15	36.69611	11.3	20.85492	72.73333	380.5	315	0
60.59583	34.28025	6.939643	0	24.79583	177.922	1003.75	967.75	0
60.71875	37.8245	9.26222	4.666667	27.70625	178.8872	959.5	923.5	0
65.10177	47.55938	6.679997	6.333334	29.0674	185.9727	943	935.75	28.75
66.37552	71.06458	7.76333	7.083334	31.98448	204.8393	983.75	947.75	0
47.01667	17.57083	0.651897	5.633333	17.72917	152.9328	760.25	726.25	0
46.85833	16.49167	0.27273	5.966667	17.77917	151.8043	763.75	729.75	0
						755.5	721.5	0
24.63625	9.5	13.69344	2.666667	9.7	75.2125	298.25	281.25	0
25.3875	9.9	13.36025	2.666667	9.95	78.9	296.5	279.5	0
25.66198	9.85	11.30901	2.308333	11.25	80.44791	291.5	278	3.5
27.62396	9.8125	9.639202	3.088021	12.45	87.00208	291.5	274.5	0
10.49167	5.75	7.09233	0	6.933333	53.31333	337.5	322.25	0
11.58333	5.5	5.873997	0	8.916667	50.41	327.75	312.25	0
10.48333	5.833334	6.628664	0	9.025	51.65729	333.75	322	3
11.65	5.616667	7.738663	0	10.20833	55.87708	335	320.5	0
52.53788	55.684	12.83333	4.65	73.98333	225.0116	1202	1113	0
58.72917	55.95833	1.666667	5.083334	78.61667	234.9081	1121	1040.5	0
60.99115	57.42917	1.583333	3.583333	81.52917	248.6953	1091	1044.75	35.5
62.48646	58.05833	1.666667	3.5	84.40417	259.3092	1159.5	1071.25	0
30.71344	27.84475	0	2.833333	39.1	231.4512	846	784.25	0
						815	757	0
14.01813	33.3775	10.78044	0.333333	38.375	173.0822	758.75	758.75	0
13.61875	33.46084	8.34191	0.333333	38.22833	172.1452	809.75	809.75	0
14.89167	35.73771	6.10227	0.833333	41.40375	197.924	819.25	826.75	7.5
21.225	36.67761	4.7	0.666667	44.82875	211.9953	827.75	827.75	0
50.95417	14.59167	5.539667	9.666667	16.38125	106.8807	724.25	640.25	0
52.06667	14.75417	5.828358	9.016666	17.075	105.5577	720.25	636.25	0
50.15025	13.96667	5.48775	5.833334	17.73125	111.417	697.25	627.5	11.25
52.75275	14.53417	4.65475	5.166667	21.90908	115.6425	712	628	0

For peer review only

28.75	3.9	0	2.416667	5.304167	63.17833	531.25	502.25	0
30.11667	3.216667	0	3.666667	3.35	65.10858	496.75	467.75	0
29.41719	3.216667	0	4.066667	3.625	65.51117	487	469.25	11.5
31.16302	4.3	0	4.183333	4.358333	69.64033	497.5	468.25	0
29.3	17.97424	12.00309	3.833333	12.90625	116.8116	622.75	592	0
31.67917	17.84508	15.46773	3.833333	13.36083	117.4668	672.5	641.5	0
30.425	17.9706	12.16112	3.666667	13.93093	119.9048	663	651.5	19
30.63333	16.64976	10.67681	3.833333	14.80092	125.0663	710.75	680.25	0
50.82086	32.84583	11.51667	11.70417	21.7307	166.6031	946.75	860	4
52.54069	32.99792	13.66667	11.99583	23.42958	169.2558	891.5	808.5	4
54.89969	32.18847	14.71823	12.35969	24.29261	171.9308	849	782.75	17.5
56.69506	31.13229	14.83906	11.97844	24.31511	170.5924	888	806.25	4
27.64167	11.58333	0	2.333333	16.06667	77.62576	607.5	569.5	0
30.09042	12.08333	0	2.333333	16.58333	81.92368	621.75	586	0
30.88582	12.94583	0	2.75	17.775	84.42083	597.25	577	15.75
33.83612	11.5375	0	2.533333	20.65833	87.52273	600	563.75	0
14.7	7.683333	0	3.25	13.5	95.08891	686.5	646.5	0
19.84167	11.89167	0	2.5	11.975	93.35799	662.25	622.25	0
21.21527	13.29091	0	2.636364	14.1	103.4144	641.25	623.25	22
						687.5	647	0
						145	0	9.5
						143.25	0	6
						144.25	0	6
24.03833	7.533333	17.20723	31.04042	9.125	62.04292	435.5	345	3
32.08258	16.78333	7.333334	0	13.17083	100.2583	684.25	635.25	0
34.27424	18.84167	2.666667	0	12.46667	98.35833	691	642	0
37.96667	18.83125	0	1.083333	13.22917	95.465	655.75	616.25	9.5
36.26061	18.22917	0	0	11.42083	107.3283	629.5	580.5	0
44.18217	15.28333	0	0	13.86667	122.7572	858.5	798.5	0
44.58236	13.65906	0	0	14.14167	124.0356	873.5	813.5	0
46.1785	12.75771	0	0	16.67292	124.4671	887.5	845.5	18
46.52271	14.28542	0	0	16.70833	123.1358	899.5	834.75	0
18.60833	15.71799	0.283333	2.916667	10.94167	99.86803	573.5	549.5	0
20.28437	15.44508	0.4	4.077083	12.80833	104.8139	585	561	0
22.5624	16.40341	0.366667	4.002083	13.66042	106.3456	545.25	540.5	19.5
21.3099	14.57008	0.25	4.95	13.325	108.1364	547.25	523.25	0
39.39167	13.68708	0.18182	0.979167	9.841666	128.4981	1031.75	947	0
33.25417	16.10875	0.204547	1	11.2125	129.0923	958.25	875.25	0
33.45142	16.59708	0.281894	0.833333	11.175	138.5849	916.75	861	27.75
45.49004	14.93583	0.51091	1	10.675	140.4301	915.75	838.5	0
59.075	13.71667	0	10.58333	9.125	117.7985	553.25	506.5	0
63.28333	11.85	0	10.25	10.6	117.9561	532	486	0
57.43427	12.2045	0	11.08594	21.15833	118.252	508.75	466.25	3.5
52.00771	12.64367	0	9.871875	20.95833	112.9378	511	465	0
5.666667	65.23959	0	0.1	49.41667	205.3767	929.75	929.75	0
4	64.78883	0	0.083333	49.19917	199.6893	899.75	899.75	0
4	62.59764	4.16529	0.002273	46.91137	196.1106	832.5	863.5	31
						888.5	888.5	0
25.16733	19.33576	0	4.666667	12.51767	164.9061	597.5	542.5	0
30.81783	18.21909	0	4.333334	15.33079	168.5393	620	565	0

35.10666	18.83751	0	4.916667	14.23138	160.4567	625.25	582.25	12.25
						635	579.5	0
127.0133	12.39091	1	6.333334	42.24417	187.9939	991.5	817.5	0
134.1029	11.3	1.25	4	43.87667	182.716	1019.75	838.5	0
139.0399	12.84229	2	5.266667	44.50114	191.5208	948	840.25	25.25
137.6189	14.1625	2	2.75	46.21261	193.6023	994	860.5	0
						264	227	0
27.907	19.945	0	1.666667	15.57708	99.1031	591.5	528.25	0
32.01983	20.74986	0.025	1.666667	17.575	101.1774	650	581.75	0
34.46667	23.21625	0.1	2	17.42083	104.7493	556.5	514.5	24.5
34.62396	24.73083	0.1	2	16.95833	102.3717	583.25	513.25	0
63.41708	9.133333	4.35	6.1	10.93333	57.98556	439.75	394.25	0
64.73729	8.825	4.708334	5.333334	10.75	56.87179	396.5	350.5	0
67.20315	9.627084	3.475	5.166667	11.91667	59.78574	333	292.5	3.25
70.35704	9.314584	3.325	5.416667	11.38333	57.97771	317.75	271.75	0
80.57459	49.08133	6.613225	8.211042	39.28019	241.081	1537.5	1433.5	0
						1584	1467.25	0
24.91278	23.1	0	13.62304	16.01875	68.3803	618.75	567.5	0
24.87471	21.8	0	14.6085	17.725	73.1228	622.25	572.25	0
24.37019	21.475	0	13.84006	17.68817	73.62517	578	553.75	21.5
26.95888	23.06667	0	14.96725	17.59358	73.19183	585.25	537.75	0
179.0434	109.8327	1.9	16.83333	95.57227	433.3868	2011.75	1874.25	0
184.4014	115.1811	1.766667	17	102.4347	440.1325	2093.25	1950.75	0
190.6936	118.2318	1	17.5	104.0719	435.9254	2051.75	1962.5	55.75
199.993	121.4125	1	17	113.0604	454.724	2089	1944.5	0
30.35625	7.25	0	0	18.98333	184.1208	629.25	601.75	0
29.89063	7	0.0115	5.166667	19.40833	186.7667	610.5	582.5	0
28.86042	6.833334	0	6.583334	20.61667	187.4552	572	574.75	30.75
30.27083	6.81875	0	5.833334	19.68021	175.5355	612.25	584.25	0
0	51.0375	0	4.333334	60.775	289.0894	1173.5	1173.5	0
7.441667	59.95167	0.534	6.639583	66.04792	319.537	1169.25	1169.25	0
80.33868	113.7062	6.191333	11.80833	103.1187	535.4528	2737.5	2663	41.5
83.46365	118.4216	6.773	12.01667	107.7948	554.811	2810.25	2704	0
67.45698	114.7948	2.053031	9.233334	84.84583	203.0143	1071	987.5	0
67.70323	118.4802	1.59091	12.06667	95.09917	213.3043	1078.5	994.5	0
68.8175	122.054	2.060607	13	100.5355	233.6066	1052	1011.25	43
68.51959	127.5663	1.5	12.6	103.1343	243.4732	1089.25	993	0
168.7417	92.29166	7.75	6.383333	67.00833	412.7667	1786.5	1705.5	0
169.6511	98.12083	7.361458	2.916667	66.22916	416.2634	1807.25	1724.25	0
170.2063	102.3198	6.748958	3.333333	71.06458	418.4019	1757	1723	61.25
173.1885	102.3063	0.616667	4	75.59583	431.6732	1810.75	1714.75	0
57.0625	32.34792	1	0	26.41333	184.2353	1076	1030	0
50.06494	34.05938	0.75	0	25.95958	184.1247	1092.25	1046.25	0
51.9328	35.84842	0.05	0.666667	27.78361	192.8477	1038.25	1014.25	21
51.18548	35.54314	0.2	4.833334	29.08632	195.4676	1063.75	1018	0
26.84167	25.82183	3.93275	17.38333	17.37083	100.4604	1146	1057.25	0
26.69167	20.47392	3.758333	17.08438	17.4	100.2431	1084	1004	0
30.15833	21.10358	1.985	13.89792	17.16198	115.0385	1022.25	976.75	28
29.68958	21.64717	1.69	13.18854	17.79167	110.681	991	928.25	0
50.93779	24.58333	0	9	31.70208	206.225	1162.25	1090.25	0

For peer review only

52.86904	25.65	0	9	36.11979	204.61	1149.25	1076.75	0
74.3221	28.82604	0	12.5	52.18104	275.2604	1452	1392.75	26.25
80.91704	32.01979	0	15.33333	100.8597	310.9868	1680.75	1582.5	0
161.701	86.77747	6.258333	14.98333	88.79375	416.4966	1441.5	1340.5	0
168.187	84.5125	1.908333	10.53333	93.40434	435.9766	1352.25	1257.75	0
167.1726	87.75584	1.925	14.1	96.7605	435.9406	1200	1163.75	52.25
177.7219	91.96667	0.991667	14.325	97.45025	447.7464	1226.5	1140.5	0
60.83104	12.24167	0	12.1	19.24553	118.2418	604.75	551.75	0
58.94969	10.5	2	11.42708	19.28011	126.3198	578.25	525	0
60.29131	11.16667	1.5	11.59844	19.09053	128.8932	577	538.25	13.25
59.73517	12.33333	0	9.989583	18.36969	129.3592	585.5	532.5	0
40.02114	17.125	0	12.78833	16.92102	118.1618	733.25	691.25	0
44.87065	19.0875	0	14.435	17.37878	118.2783	748.25	706.75	0
44.64869	18.39167	0	12.57573	21.39337	120.7972	741.5	714.75	14.75
44.86031	18.31667	0	8.620833	20.81212	119.6951	778.75	737.5	0
58.60591	31.92017	4.6	0	29.955	246.1957	1102.5	1049.25	0
59.54299	34.67877	4.166667	0	30.67792	251.228	1080	1029	0
58.77282	37.16117	0.8	0	30.63021	254.1516	1015.25	1002.25	34.5
61.53073	34.47473	0	0	32.30042	262.5197	1024.75	974.75	0
37.33067	16.25833	0	5.75	17.7375	132.3508	848	793	0
33.99367	15.45417	0	5.166667	17.63333	137.7875	832	781.25	0
37.7607	17.8375	5.783339	7.683333	21.32279	138.4252	773.75	766.75	31.25
43.77345	19.50833	0.916667	5.666667	26.416	141.8307	876.5	832	0
27.104	58.97883	0	10.425	32.15833	263.2792	1055	983.25	0
28.06417	63.10217	0	9.516666	35.43333	267.6848	1051.75	985	0
27.49542	64.28008	0	9.904166	34.7215	276.5395	958.75	917	25.25
28.22667	67.10646	0	14.50833	35.127	282.6401	952	885	0
67.245	30.75833	3.333333	7	20.025	120.8892	1051.75	954.75	0
74.66042	29.90833	2.616667	7.666667	21.2375	122.2188	1054.25	957.25	0
73.52917	30.65833	0.05	5.916667	20.70833	133.1328	994	934.5	36
80.63125	30.825	0	6	21.95417	141.1661	1015	920	0
80.54063	34.50834	0	19.81667	40.98617	201.8123	1109.75	1052	0
78.50823	34.90833	0	20.00573	43.33617	199.8139	1089	1032.25	0
82.54	36.8894	0	19.09531	50.06513	209.6042	1070.75	1045.5	12.25
84.60573	36.57652	0	20.19479	52.38617	231.9316	1096	1058.5	0
47.39834	11.444	5.75	6.166667	19.16667	100.6583	739.5	693.5	0
50.54558	12.78333	5.233333	2.333333	20.58333	102.9625	738.75	692.75	0
47.30471	13.84583	4.983333	4.727083	19.7875	98.61108	674	648.5	20.75
46.04927	13.28333	5.566667	6.189583	21.88333	97.99157	683.75	637.75	0
27.7	2	0	0.666667	6.083334	66.33352	496.5	454.5	0
24.35167	1.833333	0	0.333333	7	59.48901	486.25	445.5	0
30.03917	1	0	0.166667	6.833334	65.69584	452.5	420.25	6.75
31.19333	1	0	1.333333	5.733333	68.93259	482.75	454.5	0
177.9909	71.93333	28.985	20.50833	47.15625	321.7277	1355.5	1241.5	0
						1323.5	1210	0
88.65833	35.1	1.075	9.983334	36.77917	205.8507	1400	1267.25	0
100.2313	36.8375	0.475	42.24414	34.49104	212.7986	1328	1198.25	0
98.69944	33.275	0.083333	7.903125	30.92854	201.1556	1308.25	1215	29.25
101.1998	26.69896	0	3.166667	29.60206	209.4267	1352.5	1230	0
51.50021	35.34167	0	0	47.78833	257.9691	1241.5	1130.75	0

For peer review only

50.38667	30.475	0	0	54.37844	252.2634	1218.75	1101.5	0
48.57906	30.89583	0	0	58.22094	248.2081	1175	1105.25	32.5
55.98208	34.55416	0	0	59.1774	271.0893	1206	1130	0
59.55333	25.27778	1	16.08333	21.28333	225.654	1407.5	1250.5	0
58.97985	23.03434	0.666667	13.41667	19.12717	219.4146	1392.75	1250.5	0
52.90417	21.49583	0	12.41667	19.50623	212.7321	1426.75	1339.25	50.75
55.85717	22.14167	0.083333	9.833333	19.90467	209.8398	1470.25	1321.75	0
97.22646	86.58334	0	1.583333	82.08958	358.5763	1808.75	1668.5	0
124.687	85.72083	0.083333	0.583333	91.80271	380.0126	1831.75	1693	0
135.0119	92.06563	0	0	90.72656	379.035	1823.25	1751.25	54.25
150.4923	93.34323	0	0	76.99167	386.8727	1907	1783	0
42.275	35.21833	0	14.38333	23.09833	166.0693	899.25	852.75	0
40.48416	40.87083	0	14.85	22.535	159.7119	913	863.25	0
34.68333	39.74509	0	14.91667	22.39243	152.2178	824.25	796.75	20
40.14167	35.82796	0	14.36667	24.8657	167.6117	831.25	784.25	0
41.67083	27.4625	0	7.95	17.15	126.7604	812.25	740.5	0
47.475	26.85	0	6	19.78333	134.1643	821.25	749.5	0
49.60256	23.8505	0	1.825521	19.16875	137.6317	813.25	759.25	18
51.93067	23.12425	0	7.893229	21.76875	142.0335	862	790.25	0
50.06635	19.13729	2	7.133333	26.5	162.3872	759.5	711.5	0
52.14264	19	0.666667	3.966667	27.22163	170.7838	795.5	729.5	0
52.48179	18.92058	1	4.020834	29.04157	179.4429	742	712.75	39
55.3265	19.08183	0.666667	4	29.97103	188.7605	795.75	720.75	0
28.95	20.74167	14.675	0.833333	16.32409	114.9837	771	732.25	0
31.11667	20.05	0	7.333334	17.53742	115.7841	751.25	712	0
30.9049	17.24104	0	3.75	18.84167	114.6559	705.5	676.25	10
29.53604	19.25833	0	4.333334	17.88553	116.8325	696.75	655	0
38.75545	24.34167	0	0	26.86307	136.2348	824.75	754.25	0
39.45237	26.89583	0	0	26.76458	136.5241	824.75	759	0
40.66435	27.48229	0	0	24.63845	147.3705	822.75	763.5	4.75
41.31018	26.58125	0	0	25.37628	159.6312	866.25	800.25	0
22.43333	14.48333	0	0	19.64167	95.02966	687	647	0
24.76854	14.35	0	0	22.0125	96.33109	667.5	627.5	0
27.15229	13.6375	2.416667	0	22.4047	90.5829	632.5	609.25	16.75
28.35635	15.16667	7.166667	0	21.82088	99.26922	648.25	608.25	0
42.03646	21.26	0	5	16.01833	127.4827	851	802	0
42.30667	20.76938	0	5.333334	15.74	128.1859	835.25	786.75	0
39.95175	21.6975	0	8.139063	15.47417	124.4291	765	732	14.75
40.28513	20.46833	0	10.44375	13.93729	135.3468	756.5	707.75	0
184.4719	80.16333	4.6125	12.34375	73.4375	358.3055	1817.25	1705.5	0
185.9207	78.16167	3.8625	11.65104	76.15208	359.1154	1787	1675.75	0
188.2728	83.84125	3.259896	12.22552	79.25918	378.7517	1749.5	1686.75	45.5
196.4894	91.1316	0.866667	12.21771	61.98554	399.2352	1855.25	1746.25	0
42.65	14.39167	8.994904	3.283333	28.41667	134.5171	793.75	763.5	0
45.62942	12.69583	8.830307	1.7	28.09583	135.2833	800	768.5	0
48.14154	12.3	8.083333	2.625333	30.30365	135.5373	765.75	740.25	6
48.44171	13.15	8.416667	2	32.79792	137.255	774.5	742.75	0
53.90931	26.09358	8.400006	4.333334	26.34545	183.9805	1295.25	1236	0
58.53448	27.025	7.800007	5.083334	27.42878	188.0769	1295.5	1233	0
58.36125	25.69792	7.966849	7.159375	29.50416	195.1151	1222.5	1222.5	59

For peer review only

55.59698	25.62083	8.283903	4.666667	31.30076	197.122	1282.75	1224	0
72.85219	33.20618	0	9.766666	48.61157	254.7581	953	880	0
77.15385	33.0375	0	20.43333	49.93473	242.9443	943.25	871.25	0
78.41377	34.78125	0	18.64167	55.25581	252.476	961	906.75	17.75
81.02665	36.27083	0	7.2	54.57821	259.2644	1001	930	0
55.55	26.81799	1.933333	5	38.11206	191.2379	836.75	774.75	0
56.90427	26.60185	2	4.666667	39.03122	185.0349	783	721.25	0
59.10848	20.91591	2	5	37.86095	186.3607	730.5	681	12.5
63.88458	16.69924	2	4.666667	36.95004	212.8315	773.75	711.75	0
29.723	22.65	16.5596	2.15	20.91083	114.1409	871.75	831.25	0
28.198	20.35	15.44369	1	20.40883	119.0534	802.75	765	0
31.27925	22.50833	15.83445	1.583333	18.48933	120.9893	754.25	733.5	17
29.62613	22.7801	15.63889	2	21.81783	122.3003	743	705	0
49.26667	22.03258	0	9.083333	36.23333	194.0279	942.25	880.25	0
52.78125	21.29333	0	7	40.86771	202.0712	938.25	876	0
52.98385	22.54167	0	7.566667	44.47133	211.9663	879.25	832.25	15
57.73146	23.90958	0	6.683333	45.51708	218.0554	899.5	837.75	0
58.41459	18.63333	7.939442	2	14.263	155.5667	1174.5	1105.25	0
57.59521	17.475	8.677218	1.708333	11.46575	162.6104	1085	1016.75	0
57.90314	17.37417	7.173054	4.510417	14.62708	160.2199	1053.5	1007	22.5
64.20989	18.27125	2.144167	7.86875	15.04375	161.0131	1037	968.25	0
47.81667	31.17083	0.443	5.833334	15.7	151.3407	791.25	734.25	0
46.475	29.52773	0.421	5.666667	16.92768	157.3767	764.75	707.75	0
43.69271	29.97001	0.385733	6.139583	18.12254	163.3758	707.75	656	5.25
52.01823	31.10257	0.035067	8.75	19.03658	165.232	644	587	0
76.6125	21.85417	22	17.06667	25.91667	159.5833	1109.5	1033.5	0
81.9375	21.72083	21.66667	17.91667	26.25	162.7583	1115.75	1040.25	0
83.25185	21.36667	10.71667	17.25	25.9605	165.3181	1092.25	1053.75	36.5
84.72742	21.01667	2.5	19.575	27.11883	175.3021	1135.75	1061.5	0
53.37333	18.69667	0	0.266667	19.20227	141.9658	807.5	755	0
52.56354	20.03271	0	0	19.89144	142.1778	785	748	0
53.80417	20.49417	0	0	19.67693	149.0204	734	704.25	7.25
53.20313	20.57583	0	0	20.32262	155.5036	782.75	745.75	0
133.6431	96.53182	0	7.416667	102.6014	271.3837	1410.75	1242.5	0
139.2013	101.6402	0	12.43333	104.6235	287.5405	1385.5	1218.25	0
144.8596	107.1897	0	12.91667	116.2222	330.9642	1348.5	1245	51.25
145.5851	117.4064	0	13	120.3035	362.6232	1461.5	1290.75	0
46.005	23.61917	0	15.28333	23.95	135.0606	1056.75	996.5	0
48.20581	25.46667	0	12.9	24.93333	135.6208	1067.25	1006.75	0
49.07915	26.72917	0	13.83542	25.10104	138.3469	1049.25	1006	16.75
51.34696	26.225	0	13.90833	24.68333	147.5	1061.75	1001.75	0

For peer review only

availablem	availablem	occupiedto	occupiedge	occupiedle	occupiedm	occupiedm	ratio_gen_	ratio_surgic
46	0	779	754	0	25.5	0	0.203214	0.159669
43.5	0	760.75	733.25	0	27	0	0.217983	0.164291
45.75	0.75	729.5	733.25	30.25	27.25	0.75	0.215215	0.166273
49.5	0	745.25	717.75	0	27.75	0	0.223667	0.175897
158.5	13	1973.5	1849	0	114	11	0.286758	0.300085
159.75	12.75	1991	1895.75	28.5	112.5	11	0.287105	0.307841
154	12	1988.25	1863	0	114	11.25	0.309042	0.336414
50	0	930	909	3.25	18.25	0	0.239092	0.198471
23	57	314.75	263	0	8.25	43.25	0.197909	0.183967
23	50	298	250.75	0	8	39.25	0.271801	0.199468
17	48.25	292.75	250.75	7.75	6.75	43	0.288217	0.206547
15.75	42.5	290.25	248.25	0	6.5	35.25	0.275322	0.203105
208	0	1958.5	1824.75	0	134	0	0.367135	0.235694
212	0	1806.5	1681.5	0	125	0	0.408109	0.262811
206.5	0	1774.5	1699.5	41.75	116.75	0	0.420974	0.27616
205.5	0	1859.25	1741.25	0	117.75	0	0.414972	0.274029
69	0	1135.25	1090.5	0	44.75	0	0.35251	0.189743
70.5	0	1284.25	1236.25	0	48	0	0.33283	0.170035
68	0	1298.75	1288.25	31.25	42	0	0.33033	0.167953
68	0	1279.25	1239	0	40.5	0	0.358551	0.180766
59.75	0	462	441	0	21	0	0.314559	0.280543
62	0	402.25	380.25	0	21.75	0	0.363705	0.322063
62	0	408.25	390.5	2.75	20.75	0	0.342398	0.324244
62	0	412.5	392.25	0	20.5	0	0.354945	0.344638
0	0	286.75	286.75	0	0	0	0.183678	0.165926
0	0	256	256	0	0	0	0.211979	0.204671
0	0	249.5	251.25	1.75	0	0	0.239842	0.212869
0	0	265.25	265.25	0	0	0		
23	0	359.5	348.25	0	11	0	0.238701	0.172733
23	0	356.5	346	0	10.5	0	0.228269	0.173579
23	0	331.5	333.5	12.5	10.25	0	0.251147	0.174463
24	0	358.25	348	0	10.75	0	0.241301	0.184626
76.25	0	831.5	784.25	0	47.25	0	0.305223	0.270254
76	0	833.25	785.5	0	48	0	0.315224	0.271577
75	1	798.75	776.25	26	49	1	0.345128	0.277912
76	0	843.5	796	0	47.75	0	0.368032	0.290295
29	0	350.25	338	0	12	0	0.368929	0.256926
20	0	326	317	0	8.75	0	0.456044	0.282109
20	0	340	330	0	10	0	0.457273	0.283538
20	0	349.75	339.75	0	9.75	0	0.471115	0.312178
63	0	590.25	579	0	11.25	0	0.326754	0.254126
52.25	0	606.25	597.25	0	9.75	0	0.3251	0.24261
61.75	0	698	682	20.25	35.75	0	0.291309	0.211535
76	0	647.25	595	0	52.25	0	0.323097	0.251851
26.75	0	503.25	492.25	0	11	0	0.328609	0.24874
31	0	513.25	500	0	13.5	0	0.353657	0.249335
31	0	510.25	503.25	6.75	14	0	0.354219	0.26606
31	0	521.25	506.5	0	14.75	0	0.42074	0.324421

For peer review only

77.25	6	1024.5	977	0	46	0.75	0.466655	0.308993
77	6	1029.75	981.5	0	46.5	1.25	0.470796	0.307691
87.5	2.5	1012.25	998	28.75	40.75	2.5	0.469522	0.304817
93.75	2	988	950	0	36	2	0.497535	0.332145
49.25	0	497.25	458.75	0	38.5	0	0.290683	0.202064
49	0	523	485	0	38	0	0.275963	0.191342
49	0	510.5	486	14.5	38.75	0	0.281739	0.198836
43.25	0	547.5	508.25	0	39.25	0	0.274381	0.189114
60.75	0	494.75	472.75	0	21.75	0	0.222069	0.176175
68.5	0	481.75	462.5	0	19.5	0	0.22451	0.187534
70.75	0	454.75	453	16	17.75	0	0.235019	0.198729
71	0	454.5	440.5	0	14	0	0.259735	0.204841
58.75	0	467	414.75	0	52.5	0	0.267253	0.240206
59.25	0	472.75	425	0	47.75	0	0.29207	0.234212
58.25	1	428.5	398.5	14.75	45.5	1	0.324159	0.257497
59	0	452.5	405.5	0	47	0	0.327741	0.269517
40.75	0	475	456	0	19.25	0	0.287745	0.265175
39.25	0	490	470	0	19.75	0	0.301796	0.250508
38.5	0	505.25	502.5	17.75	21	0	0.30539	0.250499
44.75	0	582.75	560.75	0	22.25	0		
35	0	308.25	296	0	12.25	0	0.27605	0.264868
33.5	0	305.25	292.5	0	12.5	0	0.317952	0.254956
31	0	298	294.25	8	11.5	0	0.315402	0.268012
30.75	0	313.25	301.5	0	11.5	0	0.306709	0.263675
32.75	0	482.25	460.25	0	21.75	0	0.241167	0.170352
32.25	0	456.75	438.25	0	18.5	0	0.272193	0.175274
30.5	0	441.25	430.25	4.25	15.5	0	0.26978	0.18657
31	0	458.5	440.75	0	17.5	0	0.25022	0.192437
32	0	678.5	658.5	0	20	0	0.27046	0.190886
32	0	720.5	699.75	0	20.75	0	0.272413	0.178288
32	0	743	732.25	9.25	20	0	0.252265	0.171699
32	0	738	717	0	21	0	0.247145	0.173662
37	0	698.25	665.75	0	32	0	0.226039	0.234825
37	0	718.75	689.75	0	29	0	0.2253	0.241107
37	0	706.25	690.5	11.75	27.25	0	0.288409	0.259934
37	0	726.5	701	0	25.25	0	0.282801	0.246426
27	0	522.75	502.5	0	20.25	0	0.16207	0.182264
27	0	504.5	484.75	0	20	0	0.179171	0.187691
36	0	504	492.5	7.25	18.75	0	0.179017	0.193344
36	0	523.75	505.5	0	18.75	0	0.174987	0.19721
14	0	296.75	285.25	0	10.75	0	0.237574	0.234883
14	0	277.25	269.75	0	7	0	0.326259	0.24075
13.5	0.5	248.25	251.5	9.25	6	0	0.34146	0.27256
13	0	267.5	261.5	0	6.25	0	0.312394	0.28311
28.75	0	412	391	0	21.25	0	0.205532	0.2026
23.75	0	423	401.75	0	21	0	0.213519	0.207343
16.75	0	425.75	417	7	15.75	0	0.216891	0.212454
80.5	0	650	601.25	0	48.5	0	0.234754	0.177233
80.75	0	675	625.75	0	49	0	0.235994	0.191586

For peer review only

81	0	665.75	625.25	12	53	0	0.242981	0.195288
80.25	0	690	637	0	53.25	0	0.265022	0.223565
54.75	0	1148	1114.25	0	33.75	0	0.183684	0.164815
49.25	0	1119.25	1086.75	0	32.25	0	0.200838	0.164207
52.5	0.75	1071.25	1088.75	49.5	32.75	0.75	0.214839	0.164863
52	0	1115	1083.75	0	31	0	0.23337	0.177566
28	0	299.75	285.5	0	14.25	0	0.30761	0.241557
28	0	301.25	288.5	0	13	0	0.319129	0.247337
27.25	0	287.5	280.5	4.75	12	0	0.328686	0.254635
28	0	293.75	282.5	0	11.25	0	0.330225	0.261427
40	0	364.5	348.75	0	15.75	0	0.236687	0.130544
40	0	365.5	349.25	0	16.25	0	0.232704	0.139378
40	0	316.25	312	12	16.25	0	0.268636	0.165747
38.75	0	353.25	337.5	0	15.5	0	0.246531	0.162613
28	0	516.75	495.25	0	20.75	0	0.168665	0.164433
27.75	0	533.25	513.25	0	19.5	0	0.174012	0.163762
28	1.25	500.25	507	26.75	21.25	1.25	0.215778	0.177184
30	0	535.25	513.75	0	22	0	0.241295	0.179303
20	0	662	642	0	20	0	0.269599	0.181045
22.5	0	636	614	0	22.5	0	0.301131	0.188384
23.75	0	646.5	635	11.75	23.75	0	0.308261	0.193847
27.5	0	670.25	643	0	27.5	0	0.304502	0.191071
61	0	603.25	570.75	0	32.75	0	0.216581	0.122319
59.5	0	563.5	532	0	31.5	0	0.244295	0.130225
59	0	550.5	544.75	22.5	28.5	0	0.241557	0.121517
59	0	591.75	564	0	28	0	0.241071	0.129779
53.25	0	530	495.25	0	34.75	0	0.231885	0.187986
53	0	537.75	500.5	0	37.25	0	0.253542	0.192391
53	0	534.75	517	19	37	0	0.250081	0.175725
53	0	563	530	0	32.75	0	0.247643	0.179655
54	0	714.5	682.75	0	32.25	0	0.280377	0.189286
54.25	0	747	715	0	32	0	0.276448	0.192246
55	0	718.75	707.25	20	31.75	0	0.282384	0.19487
55	0	733.25	702.75	0	30.75	0		
34	0	611.25	591.75	0	19.5	0	0.190178	0.219892
34	0	612.5	595.5	0	17.25	0	0.179644	0.220886
62.5	0	1076	1072.25	30.25	34	0	0.175102	0.221262
72	0	1234.5	1193.75	0	41	0	0.18886	0.230257
72.75	0	1376.25	1314.25	0	62.25	0	0.211865	0.190958
75.5	0	1363.5	1303.75	0	60.25	0	0.224572	0.197495
73.5	0	1317	1287.5	25.75	55.25	0	0.235323	0.205534
76.5	0	1334.75	1276.5	0	58.25	0	0.234197	0.220247
3	0	671.75	671	0	1	0	0.283849	0.148849
3	0	671.75	670.75	0	1	0	0.299746	0.155747
3	0	631.5	667.25	36.25	0.25	0	0.297533	0.161926
3	0	669.75	669.5	0	0.25	0	0.300073	0.17728
24	0	317.25	307.25	4	6	0	0.272222	0.107513
24	0	303	294	4.5	4.25	0	0.278141	0.121599
24	0	287.5	285	11	3.75	0		
45	0	720.5	695	0	25.5	0	0.236463	0.197208

For peer review only

46.25	0	715.25	689	0	26.25	0	0.329551	0.201557
49	0	694.75	687.75	20.5	27.75	0	0.309133	0.214164
49.25	0	719.5	692.5	0	27	0	0.279894	0.22227
0	0	784	784	0	0	0	0.248383	0.200292
0	0	818.5	818.5	0	0	0	0.19978	0.226203
0	0	835	863	28	0	0	0.217992	0.239298
0	0	1287	1287	0	0	0	0.264313	0.244289
64.75	0	984.25	930.25	0	53.75	0	0.265393	0.21763
62	0	936.5	882.25	0	54	0	0.266233	0.224915
61.75	0	909.25	866.75	17.25	60	0	0.263794	0.241334
62	0	926.25	867	0	59	0	0.278116	0.256273
51.25	0	405.25	384.25	0	21	0	0.235442	0.174753
47.25	0	428.75	407.25	0	21.75	0	0.234365	0.17996
41.25	0	405.25	401.75	16.75	20.75	0	0.260115	0.188642
39	0	430.25	411	0	19.75	0	0.26688	0.18384
23.75	0	491.25	474.75	0	16.5	0	0.167244	0.187209
24	0	493.25	474.5	0	19	0	0.152172	0.18484
23.75	0	452.75	452.5	17.25	17.75	0	0.166632	0.188434
24	0	457	439.25	0	17.75	0	0.187372	0.194336
31	0	478.5	461.5	0	17	0	0.200565	0.165988
30.25	0	457.75	440	0	18	0	0.232148	0.191183
31	0	440.5	422.5	0	18.25	0	0.279651	0.212183
31.25	0	476.25	458.25	0	18	0	0.257009	0.190655
67	0	605	571.5	0	33.25	0	0.222157	0.213499
103.75	0	812	764.75	0	47.75	0	0.252899	0.246486
104	0	805	780.75	20	44.75	0	0.254866	0.247224
109	0	911	866.5	0	44.5	0	0.238444	0.226314
42	0	448.75	432.75	0	16	0	0.190017	0.172752
42	0	453.25	436	0	17.25	0	0.195077	0.173758
39.25	0	432	433.5	18	16.75	0	0.191131	0.177022
38	0	470.5	454	0	16.5	0	0.203384	0.17578
38	0	611.25	587.75	0	23.75	0	0.19428	0.209529
38	0	652	626.75	0	25	0		
38	0	676	650	0	27	0		
41.5	0	386.25	373.5	0	12.75	0	0.277053	0.227475
39.75	0	392	374	0	18	0	0.302745	0.236578
35.75	0	369.25	358	0	11	0	0.338947	0.261991
44.25	0	369.75	358	0	11.5	0	0.36123	0.269829
57.75	0	1040.5	1010.5	0	29.75	0	0.323855	0.272339
54.75	0	1057.75	1027	0	30.75	0	0.349579	0.279919
55.5	0	1032	1032.25	34.75	34.5	0	0.363174	0.288034
57	0	1055.25	1017	0	38.25	0	0.392544	0.314283
50	0	856	834.75	0	21.25	0	0.257293	0.188478
50.25	0	853	832.5	0	20.75	0	0.280233	0.199698
48	0	798.75	810.75	31.75	19.75	0	0.293865	0.212993
48	0	833.75	813	0	20.5	0	0.301985	0.223146
65.5	0	1152.25	1120.25	0	32.25	0	0.323665	0.278035
64	0	1122.5	1090.25	0	32.25	0	0.339353	0.278974
63	0	1091.25	1082.25	21.5	31	0	0.365655	0.285095
56	0	1105	1073	0	31.75	0	0.383685	0.292634

For peer review only

87.5	0	1646	1582.5	0	63.75	0	0.282211	0.202527
83.5	0	1653.75	1586.5	0	68	0	0.295938	0.210963
82.75	0.75	1525.5	1507.5	43	61	0.75	0.322389	0.227389
83.5	0	1528.5	1462.25	0	66.5	0	0.335246	0.233941
51	0	1049.25	1011	0	38.25	0	0.288227	0.180767
47.75	0	1047.5	1009.25	0	38	0	0.287033	0.185908
45.75	0	1029.5	1009.75	15	34.75	0	0.318189	0.183527
46	0	1046.75	1012.5	0	34.5	0	0.328441	0.192042
47.5	0	693.75	668.5	0	25.5	0	0.273699	0.23582
49.25	0	672.25	648.25	0	24	0	0.298886	0.244042
47	0	608.25	613.5	28	22.5	0	0.325633	0.259046
56.5	0	638.5	609.25	0	29	0	0.315869	0.2718
98	0	978.25	926.25	0	52	0	0.576689	0.348424
98	0	1014.5	963.5	0	50.75	0	0.609583	0.352713
98	0	1000.25	981.25	27.75	47	0	0.591311	0.374416
98	0	1009.75	965.25	0	44.75	0	0.622209	0.385465
94	0	986.5	902	0	84	0	0.236007	0.148692
94	0	973.75	897.75	0	76.5	0	0.250967	0.162734
94	0	955.75	897.5	19.75	78.25	0	0.258217	0.166738
94	0	983	905.75	0	77	0	0.252203	0.16645
61	0	531.5	487.75	0	43.75	0	0.272547	0.169686
61	0	530.5	490.25	0	40	0	0.285642	0.177941
61	0	507.75	481	9.25	36	0	0.293863	0.200287
61	0	517.75	480	0	38.25	0	0.311002	0.199669
63	0	949	902	0	47	0	0.394235	0.253245
66	0	957.5	912.5	0	45.25	0	0.415755	0.259002
67	0	868.5	849.5	18	37.25	0	0.457244	0.297519
69	0	872.75	838.75	0	34	0	0.470309	0.318929
40	0	513	480.75	0	32.5	0	0.216294	0.147207
40	0	518.25	482.5	0	35.75	0	0.200996	0.156999
40	0	476.75	461.5	18.5	33.5	0	0.224783	0.173884
40	0	487.25	450.75	0	36.75	0	0.256138	0.183037
62	0	1396.5	1343.5	0	53	0	0.210228	0.190785
64	0	1447	1391	0	56	0	0.22028	0.186446
64	0	1397.75	1384	41.75	55.25	0	0.233042	0.195361
64.25	0	1429	1374.5	0	54.5	0	0.244552	0.204022
43	0	481.75	460	0	22	0	0.178976	0.177915
20.25	0	389.25	373.5	0	16.25	0		
16	0	339	323	0	16	0		
71.25	0	701.25	669.25	0	32.25	0	0.193507	0.192949
71	0	690.75	659.25	0	31.25	0	0.185288	0.190281
70.5	0	710.25	689	8.5	29.75	0	0.19824	0.195332
70.25	0	721	690	0	31	0	0.225428	0.218132
28	0	346.5	334.25	0	12.25	0	0.215092	0.143459
28	0	340.25	328.5	0	12	0	0.201137	0.146769
28	0	329.75	330	10.5	10.25	0	0.193122	0.150456
28	0	344.5	334.75	0	10	0	0.190748	0.151581
62	0	524	498.5	0	25.75	0	0.222153	0.207789
58.5	0	541.75	516	0	25.75	0	0.21694	0.212373
55.25	0	514.5	496	3.75	22.25	0	0.233617	0.226919

For peer review only

52.5	0	493.5	472.5	0	21.25	0	0.244836	0.257599
120	0	1455.25	1370.25	0	85.25	0	0.371521	0.289345
119	0	1512	1426.25	0	85.5	0	0.380191	0.275742
118.25	0	1451.5	1416	44.75	80.25	0	0.397488	0.286342
118.5	0	1545.75	1466.25	0	79.5	0	0.389258	0.289472
48	0	608	580.75	0	27.25	0	0.218694	0.174968
48	0	581.75	554.75	0	27	0	0.253419	0.191257
47.75	0	588.5	575.5	11.5	24.5	0	0.258535	0.187274
48	0	595	568	0	27	0	0.274162	0.204725
63	0	875.5	843.25	0	32.5	0	0.234202	0.272082
61	0	879	846	0	33	0	0.292298	0.269222
58.25	0	864.75	845	12.75	32.75	0	0.284342	0.274019
61	0	878.5	847.25	0	31.5	0	0.293913	0.291293
69.75	0	1150.75	1140.25	0	10.75	0	0.230804	0.217881
70	0	1142.25	1131.5	0	10.75	0	0.244263	0.230883
70	0	1132	1142	20.25	10.25	0	0.242928	0.230099
70	0	1149.25	1143	0	6.25	0	0.258451	0.237207
51	12	321	278.5	0	30.75	11.75	0.456719	0.257131
51.25	12	330	286.25	0	32	11.5	0.391577	0.234811
53.25	12	315	287	11.25	27.5	11.75	0.365399	0.247236
54	11.5	316.25	276.75	0	29.25	10.5	0.387154	0.262812
36	0	918	884.25	0	34	0	0.265164	0.201212
36	0	896.5	862.75	0	33.5	0	0.297655	0.207345
36	0	877.25	872.75	28.75	33.25	0	0.33629	0.213088
36	0	912.75	879.5	0	33.25	0	0.337613	0.232904
34	0	623.75	607.5	0	16	0	0.258234	0.251741
34	0	610	594	0	16.25	0	0.260553	0.255563
34	0	610	594.5	0	15.25	0		
17	0	271.75	258.25	0	13.75	0	0.230706	0.291239
17	0	258	248	0	10.25	0	0.255706	0.318145
17	0	255.5	249.5	3.25	9.25	0	0.277732	0.322437
17	0	253.25	244.75	0	8.75	0	0.313198	0.355473
15.25	0	308.75	294.75	0	14.25	0	0.237754	0.180876
15.5	0	304.25	289.75	0	14.5	0	0.227587	0.173978
14.75	0	314.25	303	3	14.25	0	0.223635	0.170486
14.5	0	326.5	312.5	0	14	0	0.236656	0.178807
89	0	1078.5	1035.25	0	43	0	0.221698	0.21735
80.25	0	1007	967.5	0	39.25	0	0.267378	0.242799
81.75	0	979.75	980.75	35.5	34.5	0	0.273206	0.253577
88.25	0	1061.75	1026.75	0	35.75	0	0.277376	0.252553
61.75	0	719.25	686	0	33.5	0	0.32721	0.337392
58	0	698	664	0	34	0		
0	0	699.75	699.75	0	0	0	0.399374	0.247349
0	0	736.75	736.75	0	0	0	0.418506	0.233655
0	0	749.25	755	5.75	0	0	0.432537	0.262151
0	0	761.75	761.75	0	0	0	0.440248	0.2783
84	0	603.25	554.5	0	49.5	0	0.216582	0.192752
84	0	601.5	553	0	48.5	0	0.219825	0.190882
82.5	1.5	577.25	537.75	8.75	49.5	1	0.215659	0.207191
84	0	592.5	535.25	0	56.5	0	0.206452	0.216053

For peer review only

29	0	486.25	465.75	0	20.5	0	0.174088	0.135649
29	0	450	429.5	0	20.5	0	0.186157	0.151592
29	0	421.25	411.25	9.25	19.5	0	0.224448	0.159298
29	0	419.25	400.25	0	19	0	0.225329	0.173992
31	0	587.25	565.5	0	22.5	0	0.334441	0.206563
30	0	635	613	0	21.25	0	0.314424	0.191626
30	0	617.25	615	19	20.5	0	0.3344	0.194967
30	0	666.5	645.75	0	20.25	0	0.346411	0.193676
82.25	0	787.75	750.75	0.5	36.75	0	0.237925	0.221916
79.25	0	768	733.5	0	34.25	0	0.252764	0.230751
76.25	0.75	741.75	721.5	12.25	32.75	0.5	0.266032	0.238296
77.75	0	764.25	729	0.5	34.75	0	0.270322	0.234009
38	0	578.75	549	0	29.75	0	0.165926	0.141395
36	0	562.25	531.75	0	31	0	0.178217	0.154064
36	0	547.5	530.25	13.5	31.25	0	0.196397	0.159209
36	0	545.75	512.75	0	33.25	0	0.213059	0.170693
40	0	603.25	583	0	20	0	0.1518	0.163103
40	0	583.5	565	0	18.25	0	0.181422	0.165235
40	0	563	566.75	22	18.25	0	0.208147	0.182469
40.5	0	611.5	593	0	18.5	0		
0	135.5	118.75	0	4.25	0	115		
0	137.25	122	0	5	0	117.25		
0	138.25	130	0	5.25	0	125.25		
20	67.5	390.25	319.5	2.25	9.75	58.75	0.279742	0.194188
49	0	659.5	626.75	0	32.5	0	0.188921	0.159965
49	0	665.25	632.75	0	32.75	0	0.198025	0.155446
49	0	619.75	596	8.75	33	0	0.227145	0.160176
49	0	587	555	0	32.25	0	0.252087	0.193384
60	0	770.5	732	0	38.25	0	0.166837	0.167701
60	0	791.75	754.25	0	37.75	0	0.161772	0.164449
60	0	757	737.75	17	36.25	0	0.181422	0.168712
64.75	0	774.25	742	0	32.5	0	0.196531	0.165951
24	0	494.25	480.25	0	14	0	0.165825	0.20795
24	0	501.5	486.5	0	15	0	0.180743	0.215445
24.25	0	486.75	491.25	19.5	15.25	0	0.186382	0.21648
24	0	506.75	491.5	0	15.25	0	0.187502	0.220013
84.75	0	852.75	814.75	0	38.5	0	0.233499	0.157715
83	0	808.5	771.25	0	37	0	0.250664	0.167381
83.5	0	767	755.5	25	36.5	0	0.268768	0.183435
77.25	0	818	778.5	0	39.75	0	0.274768	0.180386
46.75	0	521.5	496	0	25.75	0	0.303008	0.237497
46	0	484.25	457.5	0	26.5	0	0.347031	0.257827
46	0	475	451.25	3.5	27.25	0	0.323471	0.262054
46	0	461.25	437	0	24.25	0	0.296552	0.258439
0	0	872.5	872.5	0	0	0	0.346552	0.235389
0	0	833.25	833.25	0	0	0	0.361382	0.239651
0	0	781.75	812.75	31	0	0	0.364244	0.241293
0	0	819.5	819.5	0	0	0		
55	0	533.75	506.5	0	27.25	0	0.247927	0.32558
55	0	569	532.75	0	36.25	0	0.247406	0.316357

55	0	571.25	541.75	7.5	36.75	0	0.242024	0.296182
55.5	0	544.75	509.25	0	35.25	0		
174.5	0	904.25	766	0	138	0	0.390463	0.245423
181.25	0	928.75	787.25	0	141.5	0	0.414175	0.232094
133.75	0.5	840	768.25	22.25	94.25	0.5	0.43711	0.249295
133.25	0	835	766	0	68.75	0	0.457587	0.252744
37.25	0	216.5	203.5	0	13.25	0		
63.25	0	545.75	499.5	0	46.25	0	0.188028	0.198405
68.25	0	569.25	530	0	39.5	0	0.213645	0.190901
68.5	2	472	457.75	19.5	34.75	1	0.26746	0.228835
70	0	496	461.75	0	34	0	0.270455	0.221704
45.75	0	345.25	314.75	0	30.5	0	0.417283	0.184227
46	0	314.25	284.25	0	30.75	0	0.468426	0.200077
45	1.25	259.25	230.5	1.75	31.5	1.25	0.56651	0.259374
46	0	247.25	214	0	33	0	0.609598	0.270924
104	0	1426.25	1351	0	75.75	0	0.297167	0.178446
116.75	0	1512.25	1429.75	0	82.5	0		
16	35.25	571	529	0	12.75	29.5	0.195254	0.129263
16	34	569	530	0	12.25	27	0.19522	0.137968
16	29.75	533.75	520.25	19	12	20.25	0.227247	0.141519
16	31.5	536	498.5	0	12.5	25	0.260119	0.146824
137.75	0	1809.75	1747.75	0	62.5	0	0.285568	0.247968
142.5	0	1866	1806	0	60.25	0	0.279759	0.243706
145.25	0	1803	1786.25	42.25	59.25	0	0.298245	0.244045
144.75	0	1822.25	1766.75	0	55.5	0	0.310866	0.257379
28	0	523.25	502.25	0	21.25	0	0.212213	0.366592
28	0	501	479.75	0	21.25	0	0.265339	0.3893
28	0	479	481.75	21.75	19	0	0.251514	0.389113
28	0	497.75	480	0	18	0	0.246158	0.365699
0	0	1135	1135	0	0	0	0.327156	0.254704
0	0	1140.25	1140.25	0	0	0	0.358768	0.280234
116	0	2619.75	2580.75	40	79	0	0.292895	0.20748
106.25	0	2649.25	2576	0	73	0	0.306894	0.215377
71.75	12	895.5	835.75	0	52.25	7.5	0.379336	0.242913
72.25	12	857.5	798.5	0	51.5	7.75	0.421291	0.267131
71.75	12	802.5	776.5	33.25	51.25	8	0.467679	0.300846
84.5	12	843.25	785.5	0	50.25	7.75	0.487611	0.30996
81	0	1499.75	1456.25	0	43.5	0	0.257462	0.283445
83	0	1455.25	1413.5	0	41.75	0	0.272132	0.294491
95.25	0	1385.25	1382	42.5	45.75	0	0.275246	0.302751
96	0	1391	1347	0	44.25	0	0.299105	0.32047
46	0	995.5	956.5	0	39	0	0.239185	0.192614
46	0	977.5	934.5	0	43	0	0.258258	0.19703
45	0	951	934.25	20.5	37.75	0	0.274188	0.20642
45.75	0	971.25	934.5	0	36.75	0	0.287295	0.209168
45.5	42.75	927.5	876.25	0	23.25	28	0.264594	0.114648
38	42	865	827	0	20.75	17.25	0.272322	0.121213
36	37.75	809.25	787.75	21.75	21.25	21.5	0.264909	0.146034
34.75	27.75	833	794.25	0	19.25	19.25	0.271636	0.139353
72	0	1019	978	0	41.25	0	0.241926	0.210864

For peer review only

72.25	0	991	948.25	0	42.75	0	0.26505	0.215776
85.5	0	1255	1222.75	23.5	55.5	0	0.26966	0.225116
98.25	0	1394.25	1337.25	0	56.75	0	0.277262	0.232557
101	0	1260.25	1169.75	0	90.75	0	0.349948	0.356056
94.5	0	1208.25	1122.75	0	85.75	0	0.358396	0.388311
88.5	0	1093.25	1052.25	40.75	81.5	0	0.397165	0.414294
86	0	1111	1030.5	0	80.5	0	0.431111	0.434494
53	0	506	478.25	0	27.5	0	0.295575	0.247239
53	0	496.25	468	0	28	0	0.308087	0.269914
51.75	0	485.5	468.5	9.5	26.5	0	0.299891	0.275119
53	0	517	490.75	0	26.5	0	0.30577	0.263595
42	0	659.25	632.75	0	26.75	0	0.251927	0.186743
41.5	0	672.75	644.5	0	27.75	0	0.276056	0.183519
41.25	0	668.25	652.5	13.25	28.75	0	0.273399	0.18513
41.25	0	721.75	693.5	0	28	0	0.249126	0.172596
53.25	0	932.5	906.75	0	25.75	0	0.260359	0.271514
51	0	913.75	887	0	26.5	0	0.294786	0.283233
49.25	2	855.75	849.75	18.5	24.5	0.5	0.331926	0.29909
50	0	856.5	829.75	0	26.25	0	0.358697	0.316384
55	0	765	736	0	29	0	0.15534	0.179824
51	0	703	682.25	0	20.5	0	0.187759	0.20196
38.25	0	659.25	671.5	31.25	18.75	0	0.220745	0.206143
44.75	0	737	713.5	0	23.25	0	0.240068	0.198782
57.25	14.5	989.5	937	0	44.5	7.75	0.259829	0.280981
54.75	12	977.25	931	0	40.75	5	0.266528	0.287524
55	12	885.5	853.5	19.25	45	6.25	0.301471	0.324006
55	12	871.5	825	0	39.5	7.25	0.337795	0.342594
97	0	856.75	815.5	0	41.25	0	0.228259	0.148239
97	0	880.5	839.25	0	41	0	0.247944	0.145629
95.5	0	815.75	813.75	36	37.75	0	0.282127	0.163604
95	0	807.5	772.25	0	35.25	0	0.317181	0.182799
57.75	0	1018.75	978.75	0	39	0	0.305883	0.206194
56.75	0	970.5	938.75	0	31.25	0	0.32428	0.212851
40	2.5	979.5	969.25	9.75	20	0.25	0.305224	0.216254
37.5	0	969.5	944.5	0	25	0	0.320382	0.24556
46	0	650.75	635	0	16	0	0.235382	0.158517
46	0	616.25	602	0	14.25	0	0.249535	0.171034
46.25	0	574.25	580.25	20.75	14.5	0	0.251306	0.169946
46.5	0	590.25	578.75	0	11.5	0	0.249312	0.169316
42	0	379	362.75	0	16	0	0.234258	0.182863
40.75	0	395	379.25	0	15.75	0	0.219894	0.15686
39	0	382.5	373.5	4.5	13.75	0	0.221818	0.175892
28	0	392.75	380.25	0	12.5	0	0.220941	0.181282
101.5	12.5	1227.25	1134.25	0	82	11.25	0.262214	0.283648
101	13	1172.5	1080.5	0	81.5	10.5		
132.75	0	1174.5	1094.25	0	80	0	0.252528	0.18812
129.5	0	1158	1077.5	0	80.5	0	0.257832	0.197493
122.25	0	1134.5	1083.5	26.75	77.75	0	0.237769	0.185654
122.5	0	1200.5	1122.75	0	77.75	0	0.233775	0.18653
111	0	1022.5	1013.75	0	8.75	0	0.299216	0.25447

For peer review only

117.25	0	1010	997.5	0	13	0	0.313184	0.252896
102.25	0	983	989.25	22.25	16.5	0	0.358607	0.250905
76	0	1012.75	996.5	0	16.75	0	0.384101	0.272041
157	0	1188.25	1056	0	132	0	0.197858	0.213688
142.25	0	1190.25	1071.5	0	118.75	0	0.197524	0.204773
138.25	0	1313	1226.75	37.25	123.5	0	0.170061	0.173411
148.25	0	1345	1229.25	0	116	0	0.171282	0.170706
140.25	0	1535.5	1442.25	0	93.5	0	0.340872	0.248623
138.75	0	1535.25	1449.25	0	85.5	0	0.358363	0.262213
126.25	0	1508.25	1518	34.75	25.25	0	0.34895	0.249694
124	0	1632.5	1557.25	0	75	0	0.350744	0.248433
46.5	0	840.25	811	0	29.25	0	0.234714	0.204771
49.75	0	832.25	803.25	0	29.25	0	0.253962	0.198832
47.25	0	760.75	752	19.25	28.25	0	0.277459	0.202417
47	0	768.75	739.75	0	29	0	0.280781	0.226579
72	0	692.25	674.5	0	17.75	0	0.266301	0.187932
72	0	692.75	673.5	0	19.25	0	0.300292	0.199205
72	0	679.25	668.25	14.75	25.75	0	0.310307	0.205958
72	0	682.25	658.75	0	23.5	0	0.328025	0.215611
48	0	649.5	613.25	0	36.25	0	0.321263	0.264798
65.5	0	715.25	689.75	0	25.75	0	0.294462	0.247602
68.25	0	680.5	675.25	35.5	40.75	0	0.311684	0.265743
75	0	707.75	661.25	0	46.75	0	0.32201	0.28546
38.5	0	676	654.25	0	21.75	0	0.198291	0.175749
39.5	0	656.25	634	0	21.75	0	0.223396	0.182625
39.25	0	621.75	610.25	10	21.25	0	0.237395	0.187883
41.75	0	614	593.5	0	20.25	0	0.248492	0.196853
70.5	0	733	694.75	0	38.25	0	0.203563	0.196092
65.75	0	731.5	692.25	0	39	0	0.22459	0.197218
64	0	742.25	716.75	4.5	30.25	0	0.216209	0.205609
66	0	764.75	726	0	38.75	0	0.241691	0.219878
40	0	600.5	578	0	22.5	0	0.193553	0.164411
40	0	582.25	560.75	0	21.75	0	0.186941	0.17179
40	0	545.25	543.25	16.75	18.75	0	0.190789	0.166743
40	0	555.75	536.5	0	19.75	0	0.198594	0.185031
49	0	735.75	707.75	0	28	0	0.21884	0.180124
48.5	0	725	695.75	0	29.25	0	0.244234	0.184241
48	0	660.5	643	12.5	30.25	0	0.271515	0.193513
48.75	0	652.75	622.75	0	30	0	0.291552	0.217337
111	0	1528.25	1453.75	0	75	0	0.296863	0.24647
110.75	0	1573.5	1492.25	0	81.25	0	0.307794	0.240654
108.5	0.25	1513	1479.25	42.5	77	0.25	0.317337	0.256043
109	0	1592.25	1515.5	0	77	0	0.330359	0.263435
30.25	0	712	687.25	0	25	0	0.217957	0.195732
31.25	0	708	685	0	23.25	0	0.235515	0.197494
31.5	0	668.75	649.75	3	21.75	0	0.264557	0.208599
32	0	681.5	657	0	24.75	0	0.274177	0.208912
59.5	0	1160	1119.5	0	40.5	0	0.188606	0.164342
62.5	0	1175.75	1137.75	0	38.25	0	0.19544	0.165306
59	0	1045	1045.75	40	39.5	0	0.223028	0.186579

For peer review only

59	0	1067.5	1028	0	39	0	0.219592	0.191753
73	0	858.75	801.75	0	57.25	0	0.441788	0.317752
72	0	844.5	787	0	57.25	0	0.449428	0.308697
72	0	852.25	795.5	7.75	64.5	0	0.430732	0.31738
71	0	886	825.75	0	60	0	0.404411	0.313974
62	0	740.5	697.75	0	43	0	0.314155	0.274078
61.75	0	690.5	648	0	42.75	0	0.333743	0.285548
62	0	638.75	611	12.5	39.75	0	0.349424	0.305009
62	0	682.5	645.25	0	37.75	0	0.355145	0.329843
40.5	0	787.5	764.25	0	23.25	0	0.200989	0.14935
37.75	0	758.5	736.5	0	21.75	0	0.214399	0.161648
37.75	0	657.75	645.75	11	22.75	0	0.270308	0.187362
38	0	649.5	629.75	0	20	0	0.273964	0.194205
62	0	880.25	844	0	36.25	0	0.237355	0.229891
62.25	0	857.5	821.75	0	35.75	0	0.23366	0.245904
62	0	811	787	12.75	36.75	0	0.235174	0.269335
61.5	0	835	800.75	0	34.5	0	0.252762	0.272314
69.25	0	968.25	930.5	0	37.5	0	0.222977	0.167186
68.25	0	896.75	858.25	0	38.25	0	0.237893	0.189467
69	0	868.5	853.5	22.5	37.5	0	0.242795	0.187721
68.75	0	867.75	830.25	0	37.5	0	0.253138	0.193933
57	0	728	689.25	0	38.5	0	0.266037	0.219573
57	0	695	657.75	0	37	0	0.287007	0.239265
57.25	0	655	623.5	5.25	36.75	0	0.296967	0.26203
57	0	612.25	575.75	0	36.25	0	0.325255	0.286986
76.25	0	982.75	942.25	0	40.5	0	0.175428	0.169364
75.5	0	993.75	953.75	0	40.25	0	0.183419	0.170651
75	0	956	952	35	38.75	0	0.199439	0.173654
73.75	0	995.5	955.25	0	40.75	0	0.211458	0.183514
52.5	0	714.75	685.25	0	30	0	0.186654	0.207174
37	0	712.5	688.75	0	23.5	0	0.192058	0.206429
37	0	670.5	653.25	6.25	23.75	0	0.218762	0.228122
37	0	697.5	675.75	0	21.5	0	0.237931	0.23012
168	0	1139.5	1033.25	0	107	0	0.527518	0.262651
167.5	0	1104	996.5	0	107.5	0	0.564908	0.28855
161	6.75	1114.25	1060.25	38.75	99.25	6.25	0.530683	0.312157
171	0	1184.25	1073.5	0	110.75	0	0.510686	0.337795
60.25	0	995.5	962.75	0	32.5	0	0.247843	0.140286
60.25	0	993	959.25	0	33.75	0	0.248683	0.141382
60	0	966.75	943.75	14.5	37.75	0	0.240085	0.146593
60	0	969.5	940.5	0	28.75	0	0.234546	0.156831

For peer review only

ratio_obg_	ratio_path_	ratio_aest_	ratio_phm_	ratio_paed	ratio_em_	ratio_radio	ratio_denta	ratio_onco_
0.038961	0.032272	0.074337	2.01E-05	0.04198	0.043933	0.023928	0.01061	0
0.039523	0.029753	0.076625	0	0.044302	0.048628	0.023917	0.013467	0
0.040279	0.024594	0.078335	0	0.046042	0.051593	0.027363	0.012555	0
0.041176	0.02062	0.088761	0	0.04493	0.050899	0.028925	0.01193	0
0.07375	0.060803	0.163362	0.014134	0.115555	0.065853	0.050023	0.045975	0.000541
0.077702	0.061997	0.169091	0.012239	0.114658	0.068368	0.04697	0.044834	0.000571
0.0854	0.062624	0.185373	0.006168	0.126468	0.074731	0.04888	0.046251	0.000671
0.034399	0.017742	0.09451	0.012014	0.060807	0.067221	0.031314	0.023004	0
0.04943	0.020849	0.088979	0.009253	0.045605	0.079721	0.02345	0.003612	0
0.045862	0.032203	0.083525	0.007091	0.04057	0.080093	0.0222	0.003589	0
0.044859	0.034064	0.089973	0.004201	0.051238	0.071519	0.023812	0.003589	0
0.04529	0.032964	0.0939	0.004096	0.061363	0.082444	0.019923	0.003625	0
0.077634	0.049118	0.167959	0.012341	0.100441	0.099914	0.054596	0.056922	0.008116
0.084362	0.054743	0.196985	0.004847	0.107208	0.11581	0.063429	0.062093	0.00925
0.082297	0.054846	0.203673	0.003062	0.112969	0.125631	0.064764	0.060944	0.010822
0.080976	0.054513	0.213059	0.001562	0.121746	0.122526	0.065546	0.065927	0.010562
0.060662	0.041447	0.108956	0.013231	0.080196	0.071607	0.066425	0.038994	0.00055
0.055237	0.035794	0.101925	0.003991	0.066141	0.061584	0.06139	0.032472	0.000485
0.052914	0.032505	0.099985	0.002076	0.065749	0.068062	0.058487	0.033184	0.000467
0.056275	0.032321	0.107826	0.001836	0.064012	0.08782	0.061757	0.0325	0.000486
0.070585	0.060809	0.160662	0	0.054554	0.061973	0.034633	0.064784	0.095094
0.086347	0.070699	0.192569	0	0.062522	0.069956	0.041565	0.071688	0.112942
0.09093	0.070337	0.190934	0.000213	0.065546	0.083909	0.045862	0.071524	0.109571
0.098776	0.075632	0.197875	0	0.066762	0.096574	0.046104	0.072363	0.108059
0.021215	0.010171	0.063877	0	0.020401	0.048677	0.015693	0	0.003487
0.02474	0.015625	0.080973	0	0.019531	0.049543	0.023047	0	0.003906
0.020896	0.014594	0.075794	0	0.012869	0.070082	0.026235	0	0.002653
0.042115	0.011103	0.104657	0	0.045106	0.05597	0.022988	0.000168	0
0.044075	0.010405	0.110835	0	0.048001	0.055503	0.023804	0.000289	0
0.044565	0.011619	0.11081	0	0.051001	0.064468	0.026748	7.50E-05	0
0.044283	0.012165	0.113059	0	0.049591	0.069444	0.02567	0	0
0.076691	0.04414	0.249162	0.013132	0.157205	0.046414	0.081984	0.086514	0.055678
0.0782	0.046267	0.251733	0.013804	0.153059	0.052732	0.088266	0.089817	0.058909
0.083511	0.043358	0.258866	0.013542	0.168165	0.057771	0.092083	0.09494	0.057857
0.087595	0.043587	0.261815	0.008159	0.166247	0.065378	0.09599	0.098521	0.057421
0.049692	0.03927	0.184763	0.018433	0.0768	0.130005	0.042964	0.045346	0.011347
0.060612	0.03617	0.196556	0.017359	0.084655	0.117557	0.050668	0.043166	0.007177
0.065503	0.036435	0.193107	0.015774	0.097506	0.136837	0.049897	0.043005	0.008775
0.070533	0.046307	0.197464	0.016434	0.09352	0.096971	0.054734	0.048295	0.008805
0.071814	0.040688	0.138048	0.003376	0.103079	0.086143	0.0366	0.039644	0
0.065178	0.037285	0.138518	0.003133	0.101287	0.083826	0.036487	0.036504	0
0.057327	0.032634	0.123356	0.000455	0.091841	0.073966	0.033202	0.036763	0
0.06973	0.035317	0.149474	0	0.108622	0.089164	0.040154	0.043233	0
0.061902	0.022685	0.118554	0	0.066953	0.083336	0.036381	0.016554	0.006196
0.064576	0.024	0.116817	0	0.070777	0.091897	0.039367	0.013564	0.005333
0.064108	0.029376	0.125124	0	0.076996	0.098303	0.04182	0.015513	0.010432
0.077131	0.034373	0.152849	0	0.08724	0.115109	0.051396	0.017634	0.009049

For peer review only

0.095498	0.104138	0.205115	0	0.10268	0.080001	0.072601	0.024885	0.006751
0.092604	0.10824	0.213102	0	0.102987	0.079105	0.073789	0.022417	0.005977
0.094923	0.106561	0.216981	0	0.099791	0.082082	0.075223	0.022739	0.005428
0.101099	0.115197	0.234127	0	0.109492	0.093701	0.078244	0.024487	0.007088
0.094959	0.047866	0.113079	0.00218	0.106285	0.104553	0.021798	0	0.036222
0.099532	0.045515	0.110653	0.002062	0.103063	0.10447	0.02079	0	0.036838
0.093576	0.044414	0.114823	0.002058	0.117642	0.109772	0.022925	0	0.034461
0.094292	0.035551	0.11497	0.001804	0.106882	0.125316	0.02318	0	0.028763
0.080042	0.030213	0.129579	0	0.079784	0.092491	0.031798	0.006346	0
0.085508	0.022089	0.130099	0	0.088707	0.089928	0.033773	0.006486	0
0.088188	0.02145	0.131367	0	0.092545	0.101412	0.031204	0.005931	0
0.091971	0.021699	0.133436	0	0.098514	0.101835	0.033513	0.006118	0
0.084705	0.036086	0.135577	0	0.08253	0.077359	0.041868	0.02505	0.006751
0.088664	0.036	0.133103	0.000784	0.083066	0.079461	0.043608	0.026248	0.006588
0.097369	0.039058	0.146259	0.002509	0.093375	0.092651	0.045514	0.029721	0.007026
0.098814	0.040568	0.162593	0.002466	0.106292	0.114443	0.044225	0.030515	0.006905
0.040827	0.047204	0.138173	0	0.064226	0.052869	0.034561	0.033295	0.016733
0.041909	0.048387	0.1355	0	0.064958	0.045235	0.033599	0.034548	0.013334
0.047616	0.047118	0.131834	0	0.065103	0.049677	0.033364	0.033043	0.017218
0.063994	0.035501	0.131166	0	0.081588	0.085839	0.040597	0.012275	0.001356
0.064598	0.036042	0.139383	0	0.082425	0.096325	0.03755	0.013818	0.00114
0.064081	0.035849	0.133141	0	0.080685	0.091218	0.039144	0.010688	0.002266
0.060305	0.041652	0.134033	0	0.085463	0.094236	0.041941	0.008999	0.003317
0.067925	0.026779	0.099252	0.05043	0.078938	0.069546	0.012493	0.004359	0.00029
0.075223	0.029169	0.103262	0.015236	0.091287	0.059574	0.012341	0.006149	0.001027
0.072211	0.023054	0.118092	0	0.095958	0.062386	0.014604	0.00791	0.000959
0.077538	0.014447	0.118302	0	0.094022	0.059914	0.01558	0.006996	0.000425
0.04357	0.032194	0.108952	0.001519	0.064844	0.067159	0.039692	0.016097	0
0.043931	0.030654	0.097958	0.000357	0.059134	0.06245	0.03737	0.016012	0
0.042718	0.028218	0.095863	0	0.061873	0.061621	0.037881	0.015028	0
0.040652	0.027801	0.101642	0	0.07309	0.06972	0.038996	0.016132	0
0.043022	0.040566	0.113669	0.033559	0.053048	0.069412	0.033721	0.006384	0
0.045892	0.040232	0.109294	0.07178	0.055769	0.076905	0.032621	0.006959	0.000242
0.059545	0.047055	0.117368	0.082165	0.065558	0.079929	0.035151	0.008182	0
0.050147	0.041797	0.11072	0.058631	0.057426	0.083725	0.03239	0.00428	0.000238
0.042189	0.011559	0.076675	0	0.056418	0.055034	0.023151	0	0
0.044595	0.010349	0.083367	0	0.062512	0.04855	0.025168	0	0
0.044958	0.013198	0.082161	0	0.060505	0.05508	0.027327	0	0
0.048071	0.009726	0.085336	0	0.06142	0.057045	0.02753	0	0
0.060067	0.03567	0.122462	0.025004	0.057662	0.065177	0.01837	0.007128	0.005784
0.072115	0.037519	0.135138	0.01835	0.073915	0.066458	0.021356	0.006611	0.006117
0.078686	0.03798	0.164994	0.004076	0.077545	0.078065	0.026319	0.02222	0.006561
0.076849	0.035564	0.177323	0	0.076969	0.089896	0.028999	0.027294	0.00631
0.046803	0.024478	0.11315	0	0.048306	0.063725	0.027323	0.01234	0.018755
0.050736	0.027391	0.108359	0	0.05334	0.080472	0.027007	0.011287	0.020909
0.050416	0.024111	0.105372	0	0.052625	0.081338	0.025979	0.008873	0.020818
0.070825	0.023563	0.107542	0.00097	0.099559	0.075232	0.034137	0.045036	0
0.075879	0.024727	0.098067	0.001465	0.10198	0.087009	0.035968	0.044121	0

0.078321	0.027251	0.101286	0.001333	0.104792	0.080828	0.032701	0.045552	0
0.080738	0.028477	0.122943	0.000131	0.107921	0.088406	0.036512	0.047894	0.001279
0.041224	0.018028	0.081916	0	0.043146	0.040261	0.022654	0.020191	0.01062
0.039322	0.021536	0.090729	0	0.043359	0.042774	0.022295	0.020767	0.006901
0.040287	0.022901	0.090963	0	0.048676	0.044919	0.022103	0.021718	0.001148
0.040909	0.022384	0.097726	0	0.049084	0.051593	0.021651	0.02252	0.001415
0.07141	0.052166	0.131626	0.048073	0.076518	0.095534	0.030064	0.004784	0
0.073985	0.05042	0.136473	0.046606	0.080404	0.101964	0.030329	0.004735	0
0.077441	0.056358	0.137115	0.041769	0.083629	0.110381	0.031855	0.00452	0
0.081366	0.059554	0.137801	0.040444	0.089025	0.113079	0.03354	0.002099	0
0.062817	0.029343	0.083642	3.91E-05	0.051697	0.06364	0.025591	0.002867	0
0.061185	0.02472	0.080973	0	0.055712	0.070429	0.024911	0.002863	0
0.0678	0.026469	0.096839	0	0.064032	0.082168	0.028532	0.003205	0
0.057508	0.015062	0.0886	0	0.056525	0.082844	0.023586	0.002963	0
0.035323	0.007038	0.101927	0	0.047248	0.038482	0.018253	0.011859	0.010416
0.046882	0.005196	0.104124	0	0.050438	0.048092	0.017917	0.010923	0.005545
0.053637	0.006739	0.106456	0	0.057428	0.060658	0.021536	0.011584	0.006912
0.059745	0.007624	0.104501	0	0.064084	0.066505	0.019749	0.011399	0.007778
0.031724	0.0309	0.115849	0	0.045246	0.074615	0.028571	0.013561	0.022758
0.036807	0.033899	0.123283	0	0.0492	0.061046	0.032405	0.014767	0.026825
0.038066	0.037002	0.12087	0	0.049649	0.054312	0.033518	0.016039	0.017586
0.040541	0.036216	0.124779	0	0.051088	0.055771	0.033559	0.017413	0.015976
0.051804	0.030031	0.107826	0.006351	0.069644	0.042303	0.028274	0.029828	0.028194
0.055676	0.03427	0.117126	0.007174	0.075315	0.048062	0.028705	0.030599	0.029139
0.053574	0.03317	0.115224	0.008857	0.070317	0.049511	0.027536	0.029601	0.029635
0.049945	0.032651	0.118589	0.002246	0.078112	0.0552	0.028413	0.031654	0.031043
0.056503	0.032172	0.096281	0	0.061428	0.054977	0.03473	0.006983	0.000589
0.057833	0.033146	0.095288	0	0.05959	0.064923	0.037746	0.006727	0
0.058055	0.032116	0.092255	0	0.074964	0.072175	0.039255	0.006329	0
0.054182	0.032413	0.091344	0	0.069045	0.06807	0.037588	0.004498	0
0.040966	0.024533	0.0723	0	0.046834	0.054864	0.037471	0.004907	0
0.045559	0.027156	0.068263	0	0.051181	0.060723	0.036457	0.002937	0
0.04716	0.026464	0.069926	0	0.053446	0.062778	0.036515	0.003016	0

0.053079	0.024482	0.10433	0.012625	0.053795	0.056865	0.028432	0.006102	0.003816
0.049811	0.023524	0.102904	0.016505	0.059581	0.06115	0.027792	0.003964	0.010975
0.049062	0.026456	0.100728	0.004306	0.059507	0.086998	0.02513	0.012735	0.027425
0.053401	0.027623	0.108812	0.002792	0.060962	0.096818	0.027998	0.013789	0.026949
0.052527	0.030804	0.099122	0	0.05973	0.074005	0.031195	0.006442	0.001775
0.056718	0.030279	0.100522	0	0.058546	0.074566	0.031124	0.006047	0.001918
0.058522	0.026158	0.104505	0	0.06858	0.085446	0.033531	0.006246	0.002265
0.062627	0.026287	0.105715	0	0.067901	0.091408	0.033037	0.006097	0.00235
0.022399	0.031155	0.068331	0	0	0.048044	0.025087	0.004798	0
0.021793	0.028558	0.074416	0	0.001367	0.047707	0.02586	0.005157	0
0.020861	0.032689	0.078522	0	0.001499	0.05519	0.027632	0.00535	0
0.023005	0.032923	0.08229	0	0.001494	0.066378	0.026668	0.004281	0
0.048603	0.003255	0.084622	0.038686	0.041524	0.053973	0.023705	0	0
0.050907	0.003401	0.085601	0.041928	0.043707	0.05737	0.028968	0	0

0.041469	0.03535	0.123856	0.00048	0.063771	0.05462	0.034868	0.020661	0.02271

0.050007	0.038597	0.128904	0	0.068969	0.06287	0.035051	0.018609	0.020545
0.046474	0.038163	0.142192	0	0.071777	0.059187	0.036896	0.020867	0.01771
0.05273	0.037156	0.148365	0	0.073739	0.053835	0.038544	0.022332	0.026805
0.00085	0.015923	0.100117	0	0	0.064084	0.041964	0.029505	0
0	0.011943	0.095084	0	0.000102	0.051317	0.04272	0.029725	0
0.001371	0.017338	0.104388	0.000434	0.001159	0.055021	0.048576	0.032412	0
0.003367	0.03897	0.121097	0.001835	0.002396	0.06748	0.05812	0.041272	0
0.059823	0.0325	0.145891	0	0.069807	0.060396	0.036665	0.010311	0.016501
0.06402	0.032521	0.158832	0	0.068949	0.052593	0.037951	0.011387	0.017663
0.065699	0.034658	0.158506	0	0.073589	0.054723	0.032668	0.010412	0.019335
0.067759	0.036188	0.158845	0	0.077545	0.07049	0.034343	0.010208	0.0198
0.066808	0.035719	0.098508	0	0.086077	0.086894	0.021584	0.00746	0
0.062359	0.035809	0.086262	0	0.079472	0.085723	0.019024	0.006016	0
0.061937	0.03258	0.091076	0.001328	0.074339	0.082129	0.017546	0.00624	0
0.064345	0.028713	0.092224	0.001946	0.094282	0.088518	0.01764	0.004577	0
0.054457	0.020318	0.097153	0.028579	0.054132	0.052738	0.013095	0.032083	0
0.054704	0.018884	0.090177	0.029382	0.053617	0.045658	0.013628	0.033793	0
0.053318	0.02244	0.089651	0.02688	0.043866	0.058565	0.019245	0.035849	0
0.059184	0.029141	0.089872	0.022713	0.064952	0.078326	0.0214	0.036345	0
0.056952	0.024596	0.104323	0	0.0723	0.057124	0.0245	0.019646	0
0.0609	0.032875	0.10658	0	0.083716	0.06815	0.025508	0.016364	0
0.06357	0.029223	0.121527	0	0.087353	0.070452	0.028312	0.017517	0
0.063445	0.018769	0.125401	0	0.081491	0.072639	0.027283	0.015005	0
0.047798	0.023274	0.115055	0.001021	0.055001	0.068624	0.018252	0.024643	0
0.064656	0.021763	0.125168	0.000872	0.045838	0.076102	0.021271	0.019843	0
0.065229	0.02352	0.118938	0.007084	0.05051	0.08346	0.022861	0.017259	0.002615
0.058223	0.02338	0.108386	0.004154	0.06397	0.08356	0.019869	0.015306	0.004549
0.043443	0.020451	0.095378	0.007549	0.041749	0.056923	0.019642	0.00346	0
0.048739	0.018349	0.086888	0.005122	0.046859	0.062557	0.019878	0.003941	0
0.04762	0.013758	0.092666	0.003806	0.05098	0.070757	0.021338	0.004935	0
0.048527	0.015006	0.097274	0.002313	0.049504	0.071679	0.018355	0.004668	0
0.041226	0.032351	0.100389	0.002269	0.045494	0.083663	0.020176	0.025268	0.023437

0.050714	0.03827	0.119846	0.022133	0.056236	0.05542	0.030637	0	0
0.055102	0.038792	0.122296	0.025323	0.07098	0.065265	0.032023	0	0
0.061191	0.044902	0.131202	0.024418	0.086256	0.065741	0.033404	0	0
0.068098	0.048312	0.134928	0.020496	0.091843	0.079597	0.036855	0	0
0.043399	0.08726	0.161088	0.00033	0.100397	0.05071	0.064077	0.021288	0.035279
0.044528	0.091402	0.158107	0	0.103788	0.056486	0.067823	0.021982	0.039871
0.046407	0.101166	0.173805	0	0.113912	0.060361	0.068538	0.027066	0.039821
0.051116	0.101276	0.18339	0	0.119804	0.064242	0.071959	0.026162	0.033413
0.031594	0.035347	0.097894	0.000843	0.046053	0.043099	0.021116	0.019257	0.016163
0.038362	0.03785	0.100746	0.001401	0.048238	0.050519	0.020081	0.018344	0.019877
0.038284	0.041725	0.104577	0.000884	0.059367	0.063793	0.023113	0.019469	0.022962
0.043341	0.04195	0.115297	0	0.055575	0.056059	0.026779	0.019493	0.023691
0.055601	0.05594	0.187793	0	0.125394	0.055862	0.067857	0.015317	0.022489
0.050377	0.061294	0.194772	0	0.146902	0.056928	0.071459	0.016228	0.022764
0.04747	0.068131	0.202395	0	0.157882	0.059203	0.077924	0.016239	0.025548
0.048313	0.071577	0.207383	0	0.170527	0.069435	0.082108	0.016971	0.026781

For peer review only

0.036387	0.053301	0.105234	0.008068	0.02069	0.034373	0.045632	0.040992	0.029106
0.037282	0.053228	0.120037	0.007969	0.017901	0.038544	0.046601	0.042822	0.028345
0.042616	0.061858	0.132744	0.007511	0.013132	0.049592	0.051402	0.046709	0.030984
0.04412	0.066558	0.153305	0.003828	0.025198	0.045064	0.055689	0.052238	0.03406
0.039513	0.035005	0.114886	0.000989	0.050207	0.042726	0.041184	0.026438	0.031287
0.039536	0.03591	0.116052	0.000991	0.049746	0.047615	0.043289	0.028611	0.031232
0.039625	0.035473	0.112908	0.00132	0.049833	0.048927	0.043959	0.02895	0.032841
0.041282	0.034013	0.110839	0.000658	0.048739	0.052671	0.041444	0.027196	0.030145
0.057959	0.040838	0.135714	0.001496	0.072314	0.073306	0.028954	0.008913	0.032099
0.062649	0.040314	0.14177	0.001607	0.072955	0.075928	0.031423	0.009731	0.030698
0.064943	0.03988	0.14862	0.001847	0.078654	0.08385	0.031066	0.009934	0.028253
0.08079	0.041055	0.148179	0.002271	0.079029	0.086765	0.030667	0.010583	0.027493
0.094351	0.080841	0.265018	0.034737	0.235614	0.086203	0.070859	0.098636	0.044269
0.097521	0.081569	0.264598	0.031327	0.250031	0.095687	0.079977	0.100047	0.046212
0.097135	0.080943	0.287329	0.029766	0.265611	0.108511	0.09084	0.104015	0.055329
0.097118	0.088643	0.299552	0.014254	0.300991	0.103636	0.098764	0.115954	0.059991
0.081479	0.03592	0.118946	0	0.107785	0.077377	0.02021	0	0.000998
0.084408	0.037975	0.128449	0.000743	0.113678	0.08625	0.017683	0	0.000808
0.092366	0.037669	0.135632	0.000929	0.115675	0.096597	0.01773	0	0.000989
0.092868	0.038233	0.135989	0.001104	0.117758	0.101573	0.022302	0	0.001739
0.070652	0.027541	0.115676	0	0.087084	0.075419	0.031608	0.019272	0
0.073423	0.024545	0.116488	0.000204	0.094168	0.076805	0.040796	0.018988	0
0.082284	0.02403	0.119417	0.000485	0.104998	0.073627	0.041823	0.021755	0
0.087619	0.025625	0.125076	0	0.102158	0.085426	0.039635	0.020662	0
0.054108	0.066865	0.200517	0.009491	0.111197	0.087579	0.087833	0.037907	0.002217
0.058042	0.058248	0.207999	0.008384	0.115786	0.076426	0.086631	0.045231	0.002612
0.061605	0.062479	0.216884	0.003804	0.131003	0.08067	0.099304	0.049911	0.003531
0.061889	0.05657	0.214391	0	0.13806	0.087303	0.103645	0.063423	0.003577
0.04547	0.023808	0.08992	0	0.064746	0.054498	0.021529	0.004349	0.004334
0.043503	0.024041	0.090803	0	0.065022	0.053644	0.020933	0.002606	0.0038
0.048918	0.027483	0.100085	0	0.067885	0.058972	0.017721	0.003817	0.003611
0.05306	0.028508	0.106602	0	0.073952	0.069909	0.026142	0.004918	0.003698
0.036507	0.038048	0.099927	0.00459	0.059684	0.061236	0.043725	0.01646	0.014073
0.03433	0.038319	0.095362	0.002636	0.055595	0.057971	0.043258	0.015523	0.012713
0.034878	0.038378	0.096716	0.001806	0.05796	0.064305	0.048636	0.01673	0.013461
0.03724	0.038772	0.100325	0.001698	0.061007	0.067003	0.051747	0.016665	0.013931
0.05124	0.012138	0.103406	0	0.051033	0.050616	0.014067	0.002935	0.004783

0.065758	0.011624	0.104599	0.009214	0.071353	0.049461	0.019469	0.001313	0
0.069999	0.011616	0.100548	0.009455	0.077036	0.054366	0.015347	6.89E-05	0
0.068796	0.01074	0.094291	0.008805	0.077025	0.068237	0.014408	6.60E-05	0
0.06788	0.010193	0.09355	0.009275	0.077927	0.081557	0.0146	0.000187	0
0.092597	0.010621	0.073074	0.009155	0.061232	0.056744	0.0363	0.001413	0
0.088375	0.013141	0.071385	0.009949	0.062018	0.061349	0.034957	0.001542	0
0.085874	0.013144	0.07548	0.01003	0.057514	0.073277	0.033914	0.000296	0
0.064787	0.011862	0.07528	0.008444	0.059524	0.073005	0.034254	0	0
0.056637	0.032013	0.111083	0	0.059842	0.058679	0.040254	0.028672	0
0.056331	0.030733	0.105964	0	0.057683	0.055749	0.040468	0.023977	0
0.059795	0.030074	0.114047	0	0.065533	0.062559	0.042293	0.021969	0

For peer review only

0.063267	0.03157	0.130977	0	0.06808	0.071069	0.048778	0.017537	0
0.068839	0.092588	0.170823	0.026203	0.105874	0.086231	0.05421	0.076332	0.00146
0.068288	0.094759	0.171308	0.022308	0.110732	0.09005	0.043861	0.074096	0.000993
0.06999	0.0938	0.189468	0.022152	0.114143	0.095594	0.054571	0.077089	0.000824
0.069717	0.093617	0.187164	0.015925	0.113027	0.094364	0.057698	0.078311	0.001421
0.052432	0.021266	0.090092	0.00287	0.067328	0.049067	0.017018	0.001841	0
0.057458	0.026078	0.099968	0.003004	0.071661	0.055382	0.022097	0.001927	0
0.058922	0.024587	0.096448	0.003475	0.067489	0.065465	0.025297	0.001848	0
0.057853	0.024795	0.095984	0.003521	0.069759	0.077465	0.021919	0.001771	0
0.041926	0.051058	0.144378	0	0.056287	0.056193	0.111177	0.018148	0.026675
0.048166	0.055079	0.149568	0	0.062792	0.062886	0.11159	0.016604	0.027182
0.044776	0.054124	0.148454	0	0.067397	0.060004	0.112591	0.01669	0.031003
0.045479	0.058462	0.153702	0	0.069935	0.065659	0.114142	0.019968	0.02982
0.034627	0.033232	0.105178	0.000804	0.045557	0.047234	0.0314	0.0128	0.016512
0.034574	0.033349	0.10688	0.000589	0.045607	0.054795	0.035105	0.015444	0.0175
0.036114	0.034215	0.111246	0.000876	0.047982	0.068611	0.039685	0.017688	0.016905
0.037613	0.034031	0.110257	0.000875	0.051996	0.073623	0.042873	0.016944	0.015014
0.140433	0.03839	0.19744	0.110471	0.210317	0.066128	0.065622	0	0
0.136108	0.0446	0.209627	0.124034	0.197314	0.093825	0.061611	0	0
0.126476	0.050697	0.199654	0.118945	0.2017	0.121866	0.069152	0	0
0.132963	0.054201	0.207256	0.132597	0.213209	0.142224	0.075357	0	0
0.047272	0.038768	0.096151	0.007848	0.068528	0.05647	0.028042	0.014633	0.015168
0.052254	0.043842	0.104003	0.010736	0.070378	0.059576	0.032114	0.017383	0.019729
0.053417	0.054494	0.109079	0.007654	0.074594	0.063836	0.033306	0.015987	0.020849
0.059152	0.080801	0.115161	0.008827	0.07547	0.068053	0.036367	0.016597	0.027203
0.040754	0.028923	0.122934	0.001073	0.077394	0.078244	0.029184	0.034402	0
0.042971	0.027764	0.126689	0.000459	0.078886	0.072727	0.029931	0.035466	0
0.060019	0.036786	0.140445	0.053024	0.095397	0.055282	0.037561	0.011423	0
0.067372	0.039919	0.157135	0.053872	0.102369	0.069345	0.040121	0.011559	0
0.08292	0.039479	0.163793	0.045327	0.102854	0.075387	0.04509	0.00845	0
0.082414	0.040092	0.174162	0.039384	0.112866	0.092909	0.050868	0.008104	0
0.070358	0.019508	0.105468	0.024062	0.035595	0.041221	0.023523	0.004071	0
0.071268	0.018982	0.100761	0.020273	0.039977	0.050733	0.030774	0.004142	0
0.065838	0.019252	0.100969	0.021877	0.034598	0.04902	0.029785	0.00396	0
0.059304	0.017973	0.094138	0.024764	0.03728	0.042427	0.032667	0.00384	0
0.047889	0.053788	0.090361	0.012396	0.050749	0.062002	0.071464	0.013765	0.016774
0.054766	0.057838	0.101968	0.001723	0.060702	0.073421	0.081258	0.01514	0.022748
0.059869	0.058556	0.106628	0.001614	0.062188	0.07724	0.083129	0.01701	0.023209
0.058157	0.056546	0.102268	0.001623	0.060858	0.080121	0.082205	0.016942	0.023026
0.052267	0.04059	0.144611	0	0.044772	0.069944	0.056997	0.021975	0.00085
0.024229	0.047699	0.163145	0.015406	0.020033	0.088882	0.054841	0.003495	0
0.024367	0.045417	0.160403	0.011323	0.018485	0.083249	0.051888	0.003	0
0.023671	0.047335	0.160026	0.008082	0.019724	0.086284	0.054839	0.004283	0
0.025927	0.048149	0.172649	0.00617	0.027863	0.089435	0.05885	0.00594	0
0.077254	0.026315	0.123441	0.00999	0.091892	0.070123	0.029542	0.003563	0
0.076284	0.02668	0.124277	0.01054	0.094153	0.072013	0.030877	0.003572	0
0.086136	0.025972	0.131731	0.010205	0.093259	0.077106	0.032973	0.003929	0
0.09288	0.027154	0.13651	0.008696	0.098557	0.075817	0.040932	0.004149	0

For peer review only

0.058298	0.008374	0.086969	0	0.061728	0.047593	0.011388	0.007579	0
0.062398	0.007489	0.10074	0	0.07012	0.055452	0.0078	0.010246	0
0.070347	0.007822	0.108447	0	0.071531	0.068797	0.008815	0.010268	0
0.077584	0.010743	0.109213	0	0.077859	0.08591	0.010889	0.010898	0
0.047041	0.031785	0.113329	0.021226	0.051813	0.06444	0.022823	0.012273	0
0.047356	0.029111	0.099773	0.025233	0.051679	0.058598	0.021796	0.011317	0
0.045461	0.02922	0.097365	0.019774	0.049472	0.064156	0.022652	0.011392	0
0.040985	0.025784	0.095663	0.016534	0.047438	0.056921	0.022921	0.009959	0
0.059832	0.043751	0.131244	0.01534	0.067693	0.056023	0.028945	0.006704	0.010034
0.064654	0.044987	0.131765	0.018632	0.07163	0.057933	0.031942	0.004425	0.012758
0.067654	0.044613	0.138419	0.020399	0.076091	0.057409	0.03367	0.004175	0.015708
0.06682	0.042705	0.137293	0.020355	0.077771	0.071884	0.033354	0.004252	0.015009
0.061718	0.021099	0.093382	0	0.050349	0.057908	0.029265	0	0
0.065777	0.022724	0.099796	0	0.056588	0.065319	0.031186	0	0
0.067044	0.024415	0.099599	0	0.058248	0.059186	0.033522	0	0
0.069966	0.022501	0.105184	0	0.06599	0.060499	0.040289	0	0
0.042539	0.013179	0.082858	0	0.025214	0.051029	0.023156	0.0206	0.004102
0.048142	0.021047	0.092847	0	0.035118	0.055737	0.021195	0.01964	0.00559
0.062417	0.023451	0.102434	0	0.037433	0.067459	0.024879	0.023579	0.00628

0.074922	0.023579	0.106579	0.053857	0.075237	0.089252	0.02856	0.036528	0.007746
0.046667	0.026778	0.082888	0.011701	0.051189	0.046536	0.021014	0.010424	0
0.052522	0.029777	0.080736	0.004214	0.054167	0.053615	0.019702	0.010905	0
0.056577	0.031596	0.08714	0	0.063702	0.066342	0.022197	0.010221	0.000699
0.062665	0.032845	0.099032	0	0.065334	0.085796	0.020578	0.010556	0.001802
0.046326	0.020879	0.102659	0	0.060358	0.045116	0.018944	0.025558	0.014994
0.04555	0.018109	0.104106	0	0.059108	0.050767	0.018749	0.024693	0.016934
0.047903	0.017293	0.105647	0	0.062594	0.06134	0.0226	0.024229	0.015044
0.047244	0.019253	0.110527	0	0.062699	0.064759	0.022518	0.021603	0.015875
0.038192	0.032729	0.09659	0.00059	0.038747	0.044872	0.022783	0.020638	0
0.044365	0.031747	0.095452	0.000822	0.041695	0.042617	0.026328	0.02015	0
0.043554	0.033391	0.101271	0.000746	0.045929	0.048612	0.027807	0.025728	0
0.047965	0.029644	0.101857	0.000509	0.043357	0.050414	0.027111	0.029126	0
0.03769	0.016799	0.096279	0.000223	0.048348	0.050038	0.012079	0.009693	0
0.039908	0.020887	0.090756	0.000265	0.043117	0.063393	0.014538	0.010096	0
0.037939	0.021968	0.085914	0.000373	0.044277	0.065384	0.014792	0.010288	0
0.042786	0.019185	0.082958	0.000656	0.058433	0.068877	0.013712	0.00977	0
0.085362	0.027655	0.125155	0	0.119103	0.075689	0.018397	0.009341	0
0.086521	0.025902	0.141985	0	0.138324	0.093629	0.023169	0.009836	0
0.087705	0.027046	0.158338	0	0.127278	0.117316	0.046888	0.008403	0
0.096828	0.028933	0.172926	0	0.119011	0.136551	0.04796	0.008009	0
0.003056	0.074773	0.117357	0	0.006495	0.056166	0.056638	0.055758	0
0.0022	0.077754	0.137915	0	0.0048	0.06647	0.059045	0.061855	0
0.002461	0.07702	0.137418	0.005125	0.004922	0.070761	0.057719	0.057804	0

0.044039	0.038175	0.219909	0	0.049689	0.063244	0.024714	0.022399	0.002369
0.049175	0.034198	0.207677	0	0.057847	0.066377	0.028777	0.031753	0.003582

For peer review only

0.049313	0.034772	0.209906	0	0.064802	0.072206	0.026269	0.027534	0
0.122393	0.016176	0.153872	0.001305	0.165814	0.103824	0.055149	0.009312	0.009345
0.121078	0.014354	0.159664	0.001588	0.170343	0.10394	0.055734	0.008643	0.009569
0.128824	0.016716	0.174488	0.002603	0.180983	0.11585	0.057925	0.008779	0.011004
0.134625	0.018489	0.188791	0.002611	0.179659	0.130688	0.06033	0.008569	0.010596
0.041116	0.03993	0.107432	0	0.05587	0.109689	0.031185	0.020543	0.003958
0.053952	0.039151	0.098838	4.72E-05	0.060415	0.106128	0.03316	0.019377	0.004503
0.061264	0.050718	0.105666	0.000218	0.075296	0.096672	0.038058	0.024119	0.006481
0.065954	0.053559	0.112119	0.000217	0.074984	0.095162	0.036726	0.025918	0.005793
0.165764	0.029018	0.169235	0.01382	0.201484	0.128986	0.034737	0.021022	0
0.189592	0.031047	0.19122	0.016564	0.227748	0.156628	0.037819	0.020639	0
0.256555	0.041766	0.23727	0.015076	0.291554	0.182271	0.051699	0.026609	0
0.289429	0.043526	0.257039	0.015537	0.328771	0.214954	0.053193	0.024611	0
0.058834	0.03633	0.095751	0.004895	0.059641	0.051756	0.029075	0.002221	0.00074
0.049064	0.043667	0.085342	0	0.047094	0.04727	0.030281	0	1.58E-05
0.051635	0.041132	0.092643	0	0.046933	0.059735	0.033443	0	0.000106
0.057937	0.041278	0.089173	0	0.046843	0.057997	0.033999	0	0
0.065398	0.046272	0.093994	0	0.05408	0.058896	0.035293	0	0
0.048486	0.062842	0.134269	0.001087	0.102442	0.053133	0.054683	0.055042	0.015127
0.050347	0.063777	0.138513	0.000978	0.102105	0.061398	0.056719	0.053192	0.018622
0.050015	0.06619	0.145104	0.00056	0.106756	0.068428	0.058263	0.053898	0.019882
0.050856	0.068721	0.153114	0.000566	0.113198	0.067752	0.063993	0.053305	0.021803
0.050423	0.014435	0.125932	0	0.060441	0.048615	0.037797	0.008384	0
0.054334	0.014591	0.134987	2.40E-05	0.062305	0.065952	0.040455	0.009403	0
0.062798	0.014184	0.132397	0	0.059907	0.078028	0.042795	0.009446	0
0.066678	0.014206	0.138944	0	0.063064	0.078299	0.041	0.00896	0
0.012046	0.044967	0.158453	0	0	0.038414	0.053546	0.025779	0.042247
0.026122	0.052578	0.172717	0.000468	0.006526	0.042998	0.057924	0.02708	0.039465
0.038582	0.044059	0.124286	0.002399	0.03113	0.047494	0.039957	0.013328	0.017808
0.036922	0.045971	0.129567	0.002629	0.0324	0.050213	0.041846	0.013419	0.017673
0.096965	0.137355	0.256157	0.002457	0.080714	0.053903	0.101521	0.115985	0.022989
0.114391	0.148378	0.278112	0.001992	0.084788	0.05199	0.119097	0.126073	0.026087
0.125707	0.157185	0.292967	0.002654	0.088625	0.048756	0.129473	0.134495	0.026571
0.13631	0.162401	0.293343	0.00191	0.087231	0.057644	0.131298	0.138076	0.032889
0.042947	0.063376	0.163725	0.005322	0.115874	0.031714	0.046014	0.065412	0.022031
0.04317	0.069417	0.163839	0.005208	0.120022	0.033904	0.046855	0.068372	0.02153
0.045139	0.074037	0.173726	0.004883	0.123159	0.038993	0.051422	0.071458	0.022645
0.04744	0.075951	0.184653	0.000458	0.128574	0.040322	0.056122	0.080213	0.022739
0.0354	0.033819	0.118869	0.001045	0.059658	0.051106	0.027615	0.019592	0.020266
0.036211	0.036447	0.124705	0.000803	0.053574	0.052069	0.027779	0.020297	0.01887
0.035586	0.038371	0.129418	5.35E-05	0.055588	0.045294	0.029739	0.024263	0.018856
0.037352	0.038034	0.132741	0.000214	0.054773	0.055381	0.031125	0.026118	0.020376
0.03398	0.029469	0.084491	0.004488	0.030632	0.101802	0.019824	0.0124	0
0.03552	0.024757	0.08588	0.004545	0.032275	0.112218	0.02104	0.011722	0
0.033288	0.02679	0.08499	0.00252	0.038284	0.106261	0.021786	0.011886	0
0.036124	0.027255	0.087661	0.002128	0.037381	0.110649	0.022401	0.01318	0
0.051542	0.025136	0.119484	0	0.052084	0.063802	0.032415	0.024923	0.027054

For peer review only

0.055043	0.02705	0.127681	0	0.055754	0.07438	0.038091	0.026494	0.031866
0.060399	0.023575	0.130804	0	0.060783	0.079188	0.042675	0.02175	0.026218
0.062003	0.023945	0.13064	0	0.06051	0.078236	0.075423	0.021539	0.025014
0.056105	0.074185	0.182253	0.00535	0.138236	0.053534	0.075908	0.024588	0.02879
0.062717	0.075273	0.193947	0.0017	0.149799	0.066659	0.083192	0.024991	0.032092
0.063977	0.083398	0.21044	0.001829	0.158872	0.074374	0.091956	0.025662	0.03497
0.071134	0.089245	0.218995	0.000962	0.172462	0.08314	0.094566	0.031193	0.03826
0.059211	0.025597	0.140654	0	0.127195	0.078881	0.040242	0.02206	0
0.068514	0.022436	0.145489	0.004274	0.125961	0.090789	0.041197	0.023155	0
0.070583	0.023835	0.147697	0.003202	0.12869	0.084058	0.040748	0.025481	0
0.075023	0.025132	0.14092	0	0.121722	0.080816	0.037432	0.026218	0
0.056699	0.027064	0.112868	0	0.06325	0.069122	0.026742	0.016285	0
0.055436	0.029616	0.115019	0	0.069621	0.071659	0.026965	0.01702	0
0.056092	0.028186	0.111004	0	0.068427	0.070452	0.032787	0.01537	0
0.054266	0.026412	0.104912	0	0.064687	0.074543	0.03001	0.013785	0
0.045134	0.035203	0.142509	0.005073	0.064633	0.048806	0.033036	0.025715	0.018031
0.04433	0.039097	0.147411	0.004697	0.067129	0.045478	0.034586	0.027631	0.021615
0.050689	0.043732	0.160013	0.000941	0.069165	0.046963	0.036046	0.030101	0.023249
0.054656	0.041548	0.163868	0	0.074156	0.050286	0.038928	0.034634	0.023776
0.053131	0.02209	0.074571	0	0.050721	0.046462	0.0241	0.010352	0
0.053592	0.022652	0.08396	0	0.049826	0.049383	0.025846	0.011461	0
0.055379	0.026564	0.090982	0.008613	0.056233	0.056274	0.031754	0.011563	0
0.058597	0.027342	0.092881	0.001285	0.06135	0.065778	0.037023	0.013923	0
0.044137	0.062944	0.154149	0	0.028926	0.042542	0.034321	0.000623	0
0.042844	0.067779	0.15582	0	0.030144	0.042353	0.038059	0	0
0.046346	0.075314	0.177235	0	0.032215	0.050568	0.040681	0	0
0.052292	0.081341	0.189568	0	0.034214	0.058041	0.042578	0	0
0.062194	0.037717	0.109678	0.004087	0.082459	0.064381	0.024555	0.003924	0
0.064076	0.035637	0.109885	0.003118	0.088961	0.062974	0.025305	0.004349	0
0.069868	0.037675	0.121455	6.14E-05	0.090358	0.072773	0.025448	0.004731	0
0.07652	0.039916	0.131813	0	0.104411	0.087355	0.028429	0.005266	0
0.055355	0.035258	0.123557	0	0.082289	0.052874	0.041876	0.022495	0
0.060539	0.037186	0.134762	0	0.083631	0.047956	0.046164	0.023222	0
0.060612	0.03806	0.138188	0	0.085159	0.056787	0.051653	0.024195	0
0.066533	0.038726	0.159617	0	0.089577	0.071841	0.055464	0.026383	0
0.04878	0.018022	0.086179	0.009055	0.074643	0.060458	0.030184	0	0
0.055437	0.021235	0.089076	0.008693	0.083963	0.064174	0.034192	0.000831	0
0.051692	0.023862	0.097521	0.008588	0.081525	0.066002	0.034102	0.000817	0
0.058747	0.022952	0.093239	0.009618	0.079567	0.070671	0.037811	0.000864	0
0.071962	0.005513	0.112038	0	0.076361	0.077211	0.01677	0.005109	0
0.062514	0.004834	0.106284	0	0.06421	0.075346	0.018457	0.005634	0
0.06892	0.002677	0.105057	0	0.080426	0.084042	0.018295	0.005788	0
0.065027	0.00263	0.095985	0	0.082034	0.08936	0.015078	0.005378	0
0.085447	0.063419	0.197015	0.025554	0.156924	0.066832	0.041575	0.059548	0.000882
0.07915	0.032077	0.123548	0.000982	0.081022	0.081234	0.033611	0.022126	1.52E-05
0.081296	0.034188	0.145025	0.000441	0.093022	0.095567	0.03201	0.0221	0
0.07362	0.030711	0.132887	7.69E-05	0.091093	0.092044	0.028545	0.024067	0
0.069966	0.02378	0.117992	0	0.090136	0.091524	0.026366	0.021294	0
0.037752	0.034862	0.119298	0	0.050802	0.049914	0.04714	0.024246	0.019934

0.039895	0.030551	0.119083	0	0.050513	0.052658	0.054515	0.024047	0.012392
0.047311	0.031232	0.099169	0	0.049107	0.054575	0.058854	0.02513	0.01181
0.049412	0.034676	0.10464	0	0.056179	0.055842	0.059385	0.028812	0.00761
0.041824	0.023937	0.112783	0.000947	0.056395	0.050221	0.020155	0.015348	0.014178
0.041324	0.021497	0.114997	0.000622	0.055044	0.049519	0.017851	0.015468	0.012978
0.038543	0.017523	0.096622	0	0.043125	0.046812	0.015901	0.014781	0.011961
0.038178	0.018012	0.097391	6.78E-05	0.04544	0.058929	0.016193	0.012177	0.009813
0.08578	0.060034	0.169898	0	0.067413	0.085188	0.056918	0.012648	0.021413
0.070497	0.059148	0.171225	5.75E-05	0.086036	0.085848	0.063345	0.011891	0.018803
0.067161	0.060649	0.16876	0	0.088941	0.084067	0.059767	0.012418	0.01969
0.06462	0.059941	0.174117	0	0.09664	0.088082	0.049441	0.012907	0.016344
0.056003	0.043426	0.089938	0	0.052127	0.060882	0.028481	0	0.027579
0.052988	0.050882	0.098266	0	0.0504	0.053319	0.028055	0	0.021615
0.060544	0.052853	0.11093	0	0.046121	0.050252	0.029777	0	0.024815
0.070145	0.048433	0.116521	0	0.054264	0.051864	0.033614	0	0.027314
0.053811	0.040715	0.092122	0	0.06178	0.05021	0.025426	0.007314	0.004324
0.060952	0.039866	0.098841	0	0.07049	0.057407	0.029374	0.007201	0.003836
0.059104	0.035691	0.103583	0	0.074228	0.060946	0.028685	0.007333	0.003991
0.06248	0.035103	0.102273	0	0.078832	0.066239	0.033046	0.007438	0.003036
0.062627	0.031206	0.133146	0.003261	0.081641	0.072632	0.043212	0.002609	0.081399
0.056343	0.027546	0.117525	0.000967	0.075596	0.067356	0.039466	0.002879	0.072748
0.061786	0.02802	0.129063	0.001481	0.077722	0.060978	0.043009	0.00275	0.069234
0.069442	0.028857	0.136366	0.001008	0.08367	0.076538	0.045325	0.002739	0.07272
0.039218	0.031703	0.087059	0.02243	0.044249	0.043131	0.024951	0.005044	0
0.046228	0.031625	0.091916	0	0.04908	0.052908	0.027662	0.006309	0
0.05343	0.028252	0.097574	0	0.050643	0.057105	0.030875	0.005694	0
0.052182	0.032449	0.106914	0	0.049766	0.058232	0.030136	0.006361	0
0.060965	0.035037	0.129534	0	0.055783	0.057366	0.038666	0.02691	0.014712
0.069873	0.038853	0.133399	0	0.056992	0.059508	0.038663	0.025563	0.014331
0.065296	0.038343	0.127962	0	0.056734	0.069869	0.034375	0.027309	0.013471
0.067086	0.036613	0.135626	0	0.056901	0.067193	0.034954	0.026626	0.013113
0.047437	0.025058	0.088948	0	0.038812	0.051094	0.033982	0.006067	0
0.043848	0.025591	0.09189	0	0.04417	0.05386	0.039255	0.006856	0
0.050322	0.025104	0.098302	0.004449	0.049981	0.057279	0.041242	0.00746	0.000153
0.053706	0.02827	0.110573	0.013358	0.052854	0.054082	0.040673	0.008354	0.001087
0.052905	0.030039	0.095167	0	0.059395	0.047887	0.022633	0.004033	0.001531
0.05398	0.029852	0.101906	0	0.060807	0.044857	0.022623	0.00459	0
0.062255	0.033744	0.111673	0	0.062133	0.064222	0.024066	0.005053	0
0.065877	0.032868	0.113315	0	0.064689	0.078774	0.02238	0.004695	0
0.067815	0.055142	0.154153	0.003173	0.126894	0.039968	0.050516	0.020722	0.024516
0.066453	0.052378	0.155708	0.002588	0.124591	0.04027	0.051032	0.023646	0.027574
0.072203	0.056678	0.163917	0.002204	0.127276	0.045096	0.053581	0.021635	0.029574
0.075948	0.060133	0.165135	0.000572	0.129653	0.04826	0.040901	0.02294	0.030845
0.046645	0.020941	0.108706	0.013088	0.062059	0.051683	0.041348	0.018029	0.003031
0.049373	0.018534	0.11219	0.012891	0.066612	0.060147	0.041016	0.017397	0.003163
0.048095	0.01893	0.119485	0.012441	0.074092	0.075614	0.046639	0.017289	0.003309
0.049219	0.020015	0.122486	0.012811	0.073732	0.081138	0.049921	0.01784	0.004871
0.039939	0.023308	0.073778	0.007503	0.048155	0.055516	0.023533	0.014828	0
0.040307	0.023753	0.074446	0.006856	0.051448	0.053301	0.024108	0.01346	0
0.047528	0.024574	0.08457	0.007618	0.055808	0.060769	0.028213	0.016679	7.97E-05

For peer review only

0.051474	0.024923	0.08761	0.008058	0.054083	0.062757	0.030448	0.018319	0.000649
0.067107	0.041417	0.182655	0	0.090866	0.101929	0.060632	0.021323	0.034227
0.068552	0.041979	0.196896	0	0.098035	0.120789	0.063449	0.02104	0.033541
0.065233	0.043723	0.190249	0	0.098572	0.12724	0.06946	0.01889	0.035823
0.063034	0.043925	0.189108	0	0.098125	0.127668	0.066095	0.0189	0.035351
0.07775	0.038435	0.121981	0.002771	0.079613	0.075254	0.054621	0.000143	0
0.077939	0.041052	0.13343	0.003086	0.087815	0.086927	0.060233	0.000154	0
0.08443	0.034232	0.139352	0.003273	0.096741	0.103715	0.061966	0.000123	0
0.080143	0.02588	0.139755	0.0031	0.099007	0.121495	0.057265	0	0
0.040627	0.029637	0.084647	0.021668	0.038892	0.048038	0.027361	0.00169	0
0.04218	0.027631	0.085349	0.020969	0.038286	0.054263	0.027711	0.002829	0
0.046732	0.034856	0.099927	0.024521	0.048439	0.070399	0.028632	0.006135	0
0.048626	0.036173	0.109746	0.024833	0.047044	0.079744	0.034645	0.007977	0.000132
0.039336	0.026105	0.142575	0	0.058373	0.054018	0.04293	0.021738	0.026461
0.043886	0.025912	0.154095	0	0.06423	0.064307	0.049733	0.024081	0.032025
0.045309	0.028643	0.171043	0	0.067324	0.081976	0.056507	0.024053	0.037689
0.045031	0.029859	0.176566	0	0.072097	0.078955	0.056843	0.021773	0.038086
0.047726	0.020025	0.094878	0.008532	0.062778	0.054517	0.015328	0.001827	0
0.052751	0.020361	0.103628	0.01011	0.067108	0.05289	0.013359	0.002034	0
0.061347	0.020356	0.105033	0.008404	0.067842	0.059753	0.017138	0.00311	0
0.068782	0.022007	0.108286	0.002583	0.077338	0.063982	0.01812	0.010083	0
0.060096	0.045224	0.121641	0.000643	0.069375	0.049532	0.022778	0.011942	0.006432
0.065271	0.044892	0.129817	0.00064	0.070658	0.052464	0.025736	0.013959	0.006132
0.069165	0.048067	0.146994	0.000619	0.070077	0.058748	0.029066	0.014168	0.005418
0.083625	0.054021	0.165161	6.09E-05	0.090349	0.075865	0.033064	0.016071	0.002836
0.056248	0.023194	0.097769	0.023348	0.081308	0.052781	0.027505	0.021719	0.002123
0.054055	0.022774	0.099869	0.022717	0.085911	0.048816	0.027523	0.02227	0.002796
0.055633	0.022444	0.099679	0.011257	0.087449	0.057997	0.027269	0.022634	0.00105
0.057629	0.022001	0.100736	0.002617	0.088697	0.070614	0.028389	0.023438	0
0.051392	0.027284	0.112387	0	0.077889	0.051952	0.028022	0.02037	0.01857
0.05118	0.029086	0.107291	0	0.076317	0.050014	0.02888	0.019536	0.017786
0.05582	0.031373	0.116922	0	0.082364	0.067024	0.030122	0.02066	0.018778
0.057854	0.030449	0.113249	0	0.078732	0.08485	0.030074	0.02159	0.018671
0.095963	0.093425	0.203382	0	0.129342	0.122207	0.0993	0.001057	0.010408
0.106346	0.101997	0.22033	0	0.13969	0.119319	0.104991	0.001162	0.009584
0.104177	0.101099	0.232477	0	0.136628	0.09443	0.109618	0.000894	0.010692
0.108807	0.109368	0.237126	0	0.135617	0.092467	0.112067	0.000637	0.012653
0.059953	0.024533	0.096006	0	0.047785	0.048403	0.024877	0.021967	0.004189
0.05896	0.026549	0.101158	0	0.050254	0.048223	0.025993	0.021534	0.001737
0.060468	0.028322	0.102894	0	0.052004	0.059603	0.026597	0.021174	0
0.062317	0.027884	0.104421	0	0.054595	0.064928	0.026245	0.023035	0

For peer review only

ratio_bed_	ratio_bed_	ratio_bed_	ratio_bed_	ratio_bed_	ratio_bed_	ratio_bed_	ratio_bed_	ratio_bed_
4.920919	6.262961	25.66676	30.9863	13.45227	49763.5	23.82071	22.76169	41.79215
4.587515	6.086746	25.30167	33.60963	13.05054		22.57239	20.56424	41.81041
4.646518	6.0142	24.82674	40.66081	12.7657		21.71922	19.38239	36.5462
4.470925	5.685149	24.28626	48.49662	11.26619		22.25689	19.64678	34.57196
3.487256	3.332387	13.55925	16.44669	6.121365	70.7533	8.653857	15.1853	19.99099
3.483043	3.248428	12.86974	16.12986	5.913991	81.70753	8.721598	14.62675	21.29
3.235801	2.972531	11.70966	15.96824	5.394543	162.1175	7.907143	13.38129	20.45813
4.182489	5.038522	29.07036	56.36481	10.58094	83.23332	16.44536	14.87624	31.93442
5.052839	5.435756	20.23077	47.96353	11.2386	108.0721	21.92763	12.54372	42.64462
3.679162	5.013329	21.80435	31.05263	11.9724	141.017	24.64878	12.48548	45.04491
3.469612	4.841513	22.29219	29.3561	11.11448	238.0425	19.51678	13.98234	41.99581
3.632115	4.92356	22.07975	30.33605	10.64963	244.1523	16.2965	12.12948	50.19377
2.723797	4.242783	12.88089	20.35912	5.953824	81.03139	9.956067	10.00857	18.31634
2.450327	3.80501	11.8537	18.26725	5.076518	206.319	9.327686	8.634839	15.7656
2.375441	3.621089	12.15106	18.23284	4.909836	326.6176	8.851971	7.959792	15.44057
2.4098	3.649249	12.34934	18.34413	4.693531	640.1655	8.213831	8.161504	15.25653
2.836796	5.270295	16.48488	24.12732	9.178005	75.58045	12.46939	13.96518	15.05467
3.00454	5.881126	18.10391	27.93785	9.811143	250.5912	15.11924	16.23796	16.28926
3.02728	5.954059	18.89853	30.76404	10.00146	481.5888	15.20947	14.69242	17.09773
2.789005	5.532013	17.76981	30.93952	9.274169	544.6154	15.62206	11.38694	16.19255
3.179054	3.564515	14.16731	16.445	6.224235		18.33045	16.13599	28.87417
2.749482	3.104987	11.58113	14.14445	5.192936		15.99439	14.29476	24.05884
2.920579	3.084094	10.99742	14.21723	5.2374	4686	15.25639	11.91761	21.80448
2.817341	2.901599	10.12394	13.22191	5.0537		14.97852	10.3548	21.69025
5.444297	6.026797	47.13699	98.31429	15.65514		49.01709	20.54377	63.72222
4.717445	4.885885	40.42105	64	12.34975		51.2	20.1845	43.38983
4.169404	4.697725	47.85714	68.52273	13.19367		77.70618	14.26908	38.11631
4.18934	5.789291	23.74432	90.06465	9.555058		22.16976	17.86661	43.50105
4.380791	5.761066	22.68852	96.11112	9.022458		20.83291	18.01692	42.01014
3.981726	5.731882	22.43902	86.06451	9.024489		19.60744	15.51163	37.38634
4.144195	5.416342	22.58213	82.20473	8.844928		20.16478	14.4	38.95522
3.276288	3.700225	13.03939	22.65527	4.013459	76.15042	6.361105	21.54502	12.19752
3.17235	3.682195	12.78771	21.61348	3.972457	72.44161	6.533416	18.96379	11.32933
2.897475	3.598265	11.97447	23.06355	3.862998	73.84512	5.946535	17.30976	10.85983
2.717152	3.444773	11.41613	22.94245	3.819498	122.5663	6.01515	15.29561	10.41775
2.710547	3.892171	20.12404	25.46459	5.41233	54.25097	13.02087	7.692016	23.27557
2.19277	3.544732	16.49833	27.64736	5.087602	57.60725	11.8127	8.506499	19.73643
2.186877	3.526864	15.26637	27.44616	5.178486	63.39378	10.25578	7.307959	20.04125
2.122625	3.203305	14.17779	21.59485	5.064222	60.85071	10.69289	10.31238	18.27022
3.060407	3.935055	13.92489	24.57729	7.243884	296.2319	9.701336	11.6086	27.32206
3.07598	4.121845	15.34262	26.8201	7.219302	319.1775	9.872897	11.9295	27.40727
3.432787	4.727339	17.44385	30.64308	8.106623	2195.703	10.88841	13.51978	30.11869
3.095047	3.970609	14.34102	28.31508	6.690123		9.206257	11.21534	24.90408
3.043127	4.020257	16.15459	44.08209	8.434957		14.93577	11.99967	27.4872
2.827601	4.010671	15.48569	41.66667	8.560422		14.12891	10.88172	25.4022
2.823115	3.758546	15.59874	34.04171	7.99206		12.98762	10.1726	23.9121
2.376764	3.082411	12.96488	29.09288	6.5424		11.46261	8.687434	19.45695

2.142909	3.236317	10.47139	9.602666	4.875325		9.739024	12.49978	13.77387
2.124064	3.250014	10.79866	9.238707	4.692594		9.709956	12.64149	13.55209
2.129825	3.280662	10.53486	9.384334	4.608708		10.02091	12.18298	13.29378
2.009911	3.010733	9.891276	8.680777	4.27118		9.133113	10.67224	12.78056
3.440178	4.948924	10.53085	20.89184	8.843373	458.75	9.408649	9.564568	45.875
3.623676	5.226236	10.04704	21.97055	9.037267	485	9.70283	9.572159	48.09917
3.54938	5.029281	10.68646	22.5152	8.709027	486	8.500342	9.109784	43.62005
3.644571	5.287803	10.60534	28.12868	8.697947	554.4545	9.356106	7.979851	43.14058
4.503096	5.676184	12.49345	33.0986	7.717318		12.53385	10.81189	31.44853
4.454135	5.33236	11.69487	45.27065	7.686448		11.2731	11.12002	29.60947
4.25497	5.031982	11.33946	46.62093	7.612246		10.80555	9.860777	32.04716
3.85008	4.881833	10.87303	46.08544	7.494232		10.15087	9.819803	29.83912
3.741773	4.163093	11.80567	27.71158	7.375868		12.11686	12.92677	23.88482
3.423841	4.26963	11.27851	27.77778	7.513001	1275	12.03859	12.58483	22.93165
3.084909	3.883545	10.27021	25.603	6.837186	398.5	10.70949	10.79318	21.97105
3.051185	3.710336	10.11997	24.64983	6.150319	405.5	9.408011	8.737949	22.61153
3.475304	3.771088	24.49363	21.18467	7.237324		15.56998	18.91465	28.93401
3.313496	3.99189	23.861	20.66691	7.380095		15.39453	22.1068	29.76253
3.274498	3.992029	21.00131	21.22343	7.585325		15.36025	20.13005	29.97241
3.622531	3.775467	15.62638	28.16812	7.623954		12.25673	11.64972	24.63246
3.14513	3.922245	15.48035	27.74567	7.174491		12.13221	10.38154	26.631
3.170559	3.731171	15.60534	27.89513	7.510861		12.39382	10.96277	25.54686
3.260416	3.79255	16.58228	24.00863	7.460871		11.70093	10.61171	23.84301
4.14651	5.870211	14.72211	37.3428	10.0754	19.82946	12.66819	14.37907	80.04348
3.673862	5.705368	13.29373	34.28292	9.684067	65.63495	10.95451	16.78583	81.03236
3.706723	5.359929	13.84832	43.37562	8.468		10.42125	16.02918	68.4748
3.996476	5.196502	12.89685	69.21869	8.452932		10.63585	16.69047	64.18689
3.697407	5.238727	22.95152	31.06132	9.17833	658.5	15.42155	14.89002	25.19369
3.670897	5.608912	22.76283	32.62238	10.2085	2799	16.91069	16.01286	26.75908
3.964081	5.824138	23.40955	35.4386	10.43158		16.16212	16.22827	26.39829
4.04621	5.758312	24.59931	35.9699	9.838442		13.68182	14.34315	25.64339
4.424022	4.258483	23.24411	24.65118	8.797489	29.79858	18.85087	14.40667	29.65479
4.438532	4.147536	21.79018	24.85586	9.149648	13.93141	17.93111	13.00311	30.65556
3.467294	3.847136	16.79414	21.2516	8.520181	12.17064	15.25356	12.51118	28.44905
3.536059	4.058014	19.94139	23.92492	9.031776	17.05596	17.41367	11.94387	30.87369
6.170159	5.486557	23.70283	86.51363	13.04207		17.72487	18.17059	43.19484
5.581248	5.327899	22.42429	96.62791	11.99521		15.99698	20.5972	39.73361
5.586073	5.172131	22.24313	75.76923	12.17129		16.52742	18.15529	36.59443
5.714702	5.07074	20.80247	102.8136	11.71834		16.28129	17.52989	36.32335
4.20921	4.257436	16.64819	28.0344	8.165797	39.99298	17.3424	15.3429	54.43702
3.065047	4.153695	13.86676	26.65294	7.399821	54.49495	13.52914	15.04707	46.82482
2.928599	3.668911	12.70876	26.32933	6.060844	245.3659	12.89578	12.80985	37.99572
3.201086	3.532193	13.01254	28.11828	5.639417		12.99218	11.12396	34.48352
4.865431	4.93583	21.36612	40.85329	8.837823		20.70152	15.69231	36.59906
4.683425	4.822929	19.70973	36.5089	9.22856		18.74781	12.42666	37.02765
4.610601	4.706905	19.83501	41.47534	9.490214		19.0025	12.29433	38.49231
4.259778	5.642279	14.11937	42.44018	9.298716	1030.714	10.04425	13.29219	29.29355
4.23739	5.219595	13.17889	40.44185	10.19716	682.6364	9.805858	11.49307	27.80241

For peer review only

4.115541	5.120646	12.76792	36.69523	9.873044	750.3	9.542682	12.372	30.57984
3.773272	4.472964	12.38578	35.11577	8.133844	7644	9.266019	11.31144	27.38823
5.444124	6.067394	24.25798	55.46982	12.20761		23.17733	24.83791	44.14328
4.979143	6.089883	25.43097	46.43404	11.02181		23.06305	23.3786	44.85297
4.65464	6.065657	24.82189	43.66644	10.99343		20.54387	22.26244	45.24284
4.285037	5.631697	24.44434	44.67537	10.2327		20.37324	19.38244	46.18663
3.250871	4.139811	14.00368	19.16976	7.597295	20.80187	13.06885	10.46746	33.26214
3.133527	4.043072	13.51618	19.83341	7.327465	21.45644	12.43719	9.807365	32.97143
3.04242	3.927196	12.91298	17.74367	7.293126	23.94144	11.95757	9.059499	31.39193
3.02824	3.825166	12.29019	16.79161	7.256816	24.72528	11.23285	8.843382	29.8153
4.224988	7.660247	15.91915	34.0798	11.95578	25572.87	19.34366	15.71337	39.07563
4.297313	7.174711	16.34389	40.45367	12.34979		17.94957	14.19866	40.14368
3.722507	6.033297	14.74936	37.78002	10.32644		15.61708	12.17017	35.04798
4.056291	6.149583	17.38888	66.39344	11.28662		17.69138	12.07086	42.39728
5.928908	6.081506	28.30998	142.0921	9.810946		21.16485	25.98601	54.78429
5.746733	6.106409	21.33037	192.4688	9.6039		19.82646	20.79338	55.81332
4.634384	5.64385	18.64378	148.3902	9.393535		17.413	16.48579	46.43389
4.14431	5.577167	16.73794	131.1702	9.569266		15.60446	15.0364	50.63655
3.709212	5.523494	31.5221	32.36295	8.631933		22.10141	13.40217	35.00083
3.320812	5.308301	27.16905	29.49973	8.111411		20.32539	16.38098	30.85956
3.244	5.158702	26.27005	27.02559	8.273357		20.14151	18.41213	29.83439
3.284051	5.233664	24.66653	27.61187	8.014157		19.57412	17.93048	29.7982
4.617201	8.175364	19.3034	33.2993	9.274204	157.4483	14.35876	23.63923	35.36793
4.093417	7.679002	17.96096	29.18	8.537812	139.3886	13.27753	20.80638	34.83765
4.13981	8.229305	18.66581	30.14804	8.678754	112.9016	14.22138	20.19742	36.31667
4.148158	7.705381	20.02189	30.62721	8.432489	445.2632	12.80209	18.11586	35.19501
4.312476	5.319549	17.69803	31.08264	10.38623		16.27922	18.18958	28.7936
3.944123	5.19775	17.29108	30.16954	10.4945		16.78122	15.40294	26.49316
3.998712	5.690716	17.22496	31.13708	10.83952		13.33978	13.85515	25.47452
4.038064	5.566228	18.45618	30.85222	10.94759		14.48335	14.69075	26.60392
3.566622	5.283018	24.41035	40.76119	13.83135		21.35206	18.22692	26.6873
3.617311	5.201662	21.94935	36.82404	14.64914		19.53841	16.46833	27.42967
3.541277	5.131639	21.20425	37.78718	14.30082		18.71042	15.92905	27.38625
5.258235	4.547688	18.83984	40.84556	9.584936	79.2098	18.58901	17.58544	35.17214
5.566573	4.527218	20.07585	42.51041	9.717821	60.58602	16.78376	16.35334	35.98146
5.710961	4.519523	20.38249	37.79923	9.927684	232.2144	16.80474	11.49455	39.79235
5.29492	4.342971	18.72609	36.2019	9.190134	358.125	16.40356	10.32865	35.71712
4.719984	5.236751	19.0379	32.46337	10.08863		16.74204	13.51269	32.05651
4.452916	5.063407	17.63115	33.02597	9.948048		17.08063	13.41098	32.1296
4.249479	4.865366	17.08752	38.22924	9.568907		14.58158	11.70327	29.82285
4.269911	4.540361	15.96758	38.04125	9.459424		14.72725	10.93992	30.26919
3.523005	6.71823	44.64427	32.09758	14.63468			20.81442	39.86139
3.336154	6.420654	45.88702	35.01621	13.43796		731.7273	20.96109	38.66923
3.360967	6.175653	47.93633	30.59122	12.7353		667.25	18.11937	36.18983
3.332517	5.640788	43.46895	30.37349	12.15214		669.5	15.0652	37.49825
3.673475	9.301211	20.57478	307.25	11.81731	25.84917	24.0823	18.52764	42.18536
3.595294	8.223776	19.64365	294	11.68212	23.85025	22.87938	17.43083	34.52055
4.229	5.070777	24.11443	28.28845	8.073885	2085	15.68111	18.30828	28.6795

For peer review only

3.034429	4.961374	19.9971	25.90875	7.757699		14.49926	15.90593	28.53002
3.234859	4.669315	21.5174	26.20354	7.032724		13.93206	16.89555	27.10345
3.572784	4.499036	18.96449	26.91324	6.740143		13.56138	18.57529	25.94443
4.026048	4.992717	1176	62.80374	9.988321			15.60463	23.82979
5.005513	4.420815		83.73402	10.51705		9822	19.48669	23.40801
4.587319	4.178896	729.2958	57.67753	9.579682	2305.992	863	18.1748	20.58617
3.78339	4.093507	297	25.66088	8.257861	545.0239	417.4054	14.81912	17.20564
3.767994	4.594952	16.71608	30.76902	6.854416		14.32513	16.5574	27.27373
3.756105	4.446119	15.62023	30.74935	6.295979		14.50353	19.01401	26.34972
3.79084	4.143628	15.22096	28.8531	6.308897		13.58896	18.2737	30.61071
3.595626	3.902095	14.75811	27.63347	6.29544		12.89581	14.18637	29.11839
4.247323	5.722376	14.96835	27.99632	10.15148		11.61754	11.50821	46.33002
4.266841	5.556801	16.0361	27.92571	11.59256		12.58304	11.66551	52.56517
3.844457	5.301041	16.14535	30.69357	10.97989	753.2813	13.45183	12.17593	56.99249
3.747007	5.439506	15.5412	34.82744	10.84322	513.75	10.60645	11.29716	56.68966
5.979303	5.341624	18.36318	49.21814	10.29302	34.99052	18.47348	18.96156	76.36729
6.571528	5.410081	18.28021	52.95513	11.08926	34.03479	18.65085	21.90211	73.37629
6.001255	5.306885	18.75523	44.56299	11.15442	37.20244	22.7968	17.07504	51.96172
5.336982	5.145722	16.89657	34.31641	11.12697	44.02774	15.39599	12.76715	46.72872
4.98591	6.024542	17.55866	40.65781	9.585627		13.83117	17.50593	40.81663
4.307602	5.230597	16.42046	30.41825	9.382664		11.94516	14.6736	39.20404
3.575881	4.712904	15.73063	34.21976	8.228649		11.4478	14.19413	35.32116
3.890921	5.245085	15.7617	53.27972	7.974419		12.27123	13.76677	36.65267
4.501324	4.683874	20.92129	42.96723	8.69149	979.7143	18.18134	14.57227	54.7873
3.954153	4.057031	15.46649	45.94933	7.989249	1147.125	21.81604	13.1403	47.01241
3.923636	4.04491	15.33049	42.51679	8.407732	141.1658	19.79793	11.98176	43.74242
4.193861	4.418632	17.17539	42.77164	9.226281	240.7293	15.63227	11.96746	50.32914
5.262692	5.788652	23.01862	48.8983	10.48456	132.4745	23.95295	17.56766	50.91177
5.126168	5.755142	20.51765	54.5	11.50902	195.2239	21.3404	15.98533	50.30769
5.232019	5.649014	20.99962	72.68536	10.79141	262.7273	19.61539	14.13291	46.86486
4.916806	5.688926	20.60694	66.6422	10.28022	432.381	20.20022	13.95115	54.48
5.147205	4.77262	24.2567	30.91049	9.961246	440.8125	21.9808	11.95272	49.5643

3.609418	4.396083	19.71843	26.1303	8.344038	45.18145	17.78219	18.04414	32.64059
3.303105	4.22694	18.148	25.77829	8.176911	39.48966	14.08856	15.32216	31.22713
2.950316	3.816922	16.34237	22.27061	7.621836	40.95329	11.59338	15.21112	29.93624
2.768323	3.706054	14.68467	20.69863	7.411369	48.79046	10.88818	12.56324	27.1332
3.0878	3.671897	23.04176	11.45998	6.207794	3031.5	9.960474	19.72017	15.60621
2.860581	3.572468	22.45763	10.9407	6.324818		9.635068	17.70335	14.74429
2.753504	3.471811	21.54853	9.88471	5.75356		8.778743	16.56692	14.59047
2.547483	3.18185	19.56341	9.874056	5.452852		8.346943	15.56608	13.89683
3.886621	5.305671	31.65199	28.29062	10.21518	1185.653	21.71425	23.20227	47.35722
3.568454	5.007559	26.06752	26.41983	9.925916	713.5704	20.7305	19.79443	49.79936
3.402927	4.694989	26.12042	23.96638	9.562327	1131.279	16.84428	15.67566	43.265
3.311421	4.481377	23.0728	23.838	8.67324		17.99365	17.83832	37.34211
3.089613	3.596669	17.98515	17.87633	5.325015		7.974847	17.90133	14.7369
2.946787	3.584568	19.85036	16.31475	5.134213		6.807277	17.56608	13.99401
2.734822	3.5076	21.06591	14.67769	4.940841		6.333859	16.89091	12.83308
2.606302	3.417243	20.69847	13.97088	4.821998		5.864189	14.40198	12.17905

For peer review only

3.543443	4.937621	27.48263	18.76155	9.502602	123.9423	48.33291	29.09291	21.91449
3.379083	4.740169	26.82233	18.78705	8.330771	125.4845	55.86268	25.94428	21.45882
3.101841	4.397749	23.46511	16.16608	7.533269	133.1334	76.15239	20.16455	19.45462
2.982887	4.274576	22.66522	15.02459	6.522966	261.2246	39.68563	22.19053	17.95698
3.469487	5.531988	25.30843	28.56773	8.704262	1011	19.91754	23.40504	24.28114
3.483919	5.378993	25.29367	27.84778	8.616795	1009.25	20.10229	21.00189	23.10061
3.14279	5.448801	25.23632	28.19054	8.856751	757.3125	20.06696	20.43847	22.74865
3.044687	5.207184	24.2235	29.40038	9.022094	1518.75	20.51735	18.98571	24.12883
3.653643	4.240518	17.25347	24.48718	7.368421	668.5	13.82865	13.64138	34.53814
3.345755	4.097661	15.96183	24.80549	7.053703	622.32	13.70716	13.17039	31.82376
3.070946	3.860312	15.39805	25.07493	6.728588	541.3236	12.71398	11.92601	32.18994
3.165869	3.679175	12.37783	24.35782	6.74859	440.4217	12.65355	11.52541	32.60855
1.734037	2.870067	10.59875	12.36993	3.773326	28.78788	4.244229	11.60048	14.11249
1.640467	2.835167	10.25424	12.25957	3.779324	31.92159	3.999502	10.4507	12.50365
1.691158	2.670829	10.295	12.35442	3.480327	33.59486	3.764901	9.215643	11.00833
1.607178	2.594271	10.29674	11.28123	3.33832	70.15748	3.322362	9.649183	10.12511
4.237171	6.725317	12.27303	27.83951	8.407153		9.277767	12.92379	49.48114
3.984585	6.144995	11.84726	26.33341	7.78519	1346.625	8.796808	11.59416	56.55118
3.872709	5.997451	10.82648	26.54687	7.372893	1077	8.644943	10.35229	56.4022
3.965064	6.007801	10.76796	26.15515	7.353546	905.75	8.492028	9.845176	44.83911
3.669094	5.893221	14.15392	36.30893	8.644857		11.48323	13.25933	31.63784
3.500891	5.619851	13.61963	40.741	8.584561	4902.5	10.61926	13.01999	24.5125
3.402944	4.992835	12.15302	41.615	8.374016	2061.429	9.524006	13.58198	23.91052
3.215416	5.008297	11.413	39.02439	7.995142		9.788739	11.70598	25.22996
2.536555	3.948747	18.48155	14.95544	4.987099	105.3684	8.993021	11.41826	11.3853
2.405263	3.86097	17.22876	17.16806	4.807719	119.281	8.636638	13.08453	11.54325
2.187018	3.361129	16.23239	16.0055	4.61076	262.859	7.6334	12.39614	10.07014
2.126262	3.135496	16.15806	17.67728	4.664367		7.243231	11.45435	9.648314
4.623334	6.793162	21.99239	42.00219	11.12103		15.44496	18.34924	46.44928
4.975214	6.36947	22.98668	41.59483	11.01284		15.37951	18.64134	47.77228
4.448731	5.750953	20.44253	36.38633	9.991543		14.73088	16.95717	56.43175
3.90415	5.463375	18.84672	35.07782	9.38065		13.52228	14.30432	38.25217
4.756745	5.241491	27.39184	26.28228	10.00734	217.8649	16.75505	16.33032	22.87
4.539667	5.363478	29.12887	26.09669	10.48637	379.3637	17.98733	17.25008	23.11714
4.291081	5.118737	28.67154	26.05685	10.3396	553.6	17.25313	15.55093	20.56101
4.089106	4.901441	26.85305	25.79199	9.967639	589.0714	16.39153	14.92467	19.32473
5.587344	5.620661	19.51596	82.38806	9.670638		19.59531	19.75662	71.08822
5.167768	5.182715	15.20719	86.03079	9.56032	108.527	14.01479	20.21811	51.36356
5.397005	5.255389	14.28584	86.08607	9.945484	105.762	12.98096	18.39394	65.15965
5.04438	5.11949	14.53571	93.11189	10.60547	113.5714	12.98278	14.65478	69.40463
4.435997	4.584383	14.73197	98.10845	10.68945	107.8125	12.83259	12.26144	68.49145
4.649181	6.970637	10.79951	94.15493	13.68475	109.232	16.33143	17.62302	27.54808
4.971731	6.813425	11.31537	76.10039	14.00853	100.5099	16.12435	16.3002	28.60668
5.178083	6.646475	11.64496	76.08069	13.24858	99.69788	17.38701	13.64694	29.48623
5.242523	6.597142	15.43516	84.3022	13.28373	118.4257	16.80006	13.69774	29.19331
4.501411	4.812586	17.65643	31.2376	9.002257		16.71068	17.04196	24.84219
4.609562	4.708697	17.75229	32.5381	9.437137		17.33606	17.93743	24.71116
4.280515	4.406857	16.7238	33.2514	8.768334		15.2596	15.98496	23.64485

For peer review only

4.084366	3.881997	15.80598	31.67598	7.63495		14.68866	14.07079	20.50086
2.691639	3.456087	14.52655	10.80055	5.853998	38.1638	9.445216	11.59673	18.44678
2.630254	3.626573	14.64385	10.55309	5.837434	44.82714	9.030799	11.10498	22.79948
2.515796	3.492328	14.28777	10.66102	5.277927	45.14346	8.760936	10.46089	18.32466
2.568988	3.454563	14.34373	10.68177	5.342914	62.79443	8.847405	10.59725	17.33156
4.572596	5.71534	19.07225	47.02429	11.09979	348.45	14.85274	20.38031	58.76054
3.946041	5.228558	17.40392	38.34678	10.00319	332.85	13.95468	18.05656	45.25493
3.867944	5.339782	16.97145	40.67138	10.36824	287.75	14.81732	15.27538	39.53062
3.647478	4.884599	17.28523	40.33136	10.41843	284	14.33514	12.90909	45.62249
4.269822	3.67536	23.85135	19.58542	6.926244		17.76603	17.79579	8.994667
3.421165	3.714406	20.76162	18.15581	6.685919		15.92567	15.90179	8.96141
3.516896	3.64938	22.33319	18.47597	6.736115		14.83752	16.66567	8.881726
3.40237	3.432975	21.98829	17.10513	6.506089		14.29898	15.23012	8.761021
4.332688	4.589672	28.87927	30.09105	9.507656	1243.909	21.95033	21.17128	31.84685
4.093948	4.331197	28.92321	29.98609	9.356276	1697.25	21.92657	18.24969	28.48631
4.116441	4.345955	27.69009	29.22716	8.989066	1142	20.84108	14.57492	25.19812
3.8692	4.215721	26.58655	29.38462	9.069678	1143	19.23207	13.58272	23.32455
2.189529	3.889071	7.120822	26.04833	5.064841	9.052178	4.754725	15.12217	15.23877
2.553775	4.258743	7.347101	22.42167	4.77037	8.062294	5.068063	10.65819	16.23084
2.736732	4.044715	7.906648	19.72509	5.008671	8.407246	4.957848	8.205739	14.46092
2.582951	3.804995	7.520903	18.45	4.824948	7.541671	4.690223	7.031176	13.27025
3.771244	4.969875	21.15431	25.79474	10.40028	127.4201	14.59259	17.70861	35.66123
3.359589	4.822871	19.13723	22.80929	9.615145	93.14722	14.20896	16.78529	31.13918
2.973623	4.692893	18.72059	18.35075	9.167706	130.6513	13.40593	15.66507	30.02505
2.961974	4.293611	16.90554	12.37607	8.683495	113.289	13.25037	14.69446	27.49771
3.872464	3.972334	24.53719	34.57434	8.134458	931.8962	12.92095	12.78051	34.26557
3.837989	3.912934	23.2713	36.01819	7.893316	2177.978	12.67651	13.75	33.40989

4.334514	3.433605	16.66129	27.18421	7.12021	18.8594	10.48252	18.08896	26.62371
3.910747	3.143219	14.84289	25.05051	6.363972	18.56252	9.768587	14.4207	24.92462
3.600592	3.101385	12.05982	25.32995	6.105267	22.06205	9.722555	13.26481	22.17778
3.192867	2.813151	12.13386	24.94268	5.741795	25.39111	8.860063	10.76317	19.65863
4.206021	5.528636	14.21312	51.26087	9.481557	41.55898	28.09373	24.25926	42.51202
4.393933	5.747868	14.03148	52.68182	9.924503	49.32757	25.01439	19.71088	32.49533
4.471569	5.865581	15.18876	51.94286	9.903984	45.71057	28.90302	20.39975	33.57341
4.225547	5.592633	16.86238	55.63798	10.62267	40.38165	26.82403	23.57008	30.61225
4.510632	4.600875	20.88183	18.59152	11.06677	80.66883	19.70483	16.12857	13.99302
3.74002	4.118633	18.25962	17.28965	9.806986	580.5	16.47393	13.62009	12.30655
3.660243	3.943581	16.70327	17.07756	9.378436	619.4211	16.0802	12.94663	12.02944
3.60521	3.959559	17.19489	17.6848	9.778183	616.05	16.43156	12.48115	12.16468
3.056144	2.963908	19.13248	24.6366	6.91508		22.3355	14.29707	17.54476

2.503916	4.042877	41.27304	20.96472	6.129533	64.90921	49.91752	11.25093	18.23453
2.389453	4.279817	41.03981	22.01828	6.234288	88.31909	54.09821	12.01219	19.27235
2.31194	3.814596	42.24638	21.12614	6.248976	123.7245	50.6995	11.58967	18.23506
2.271448	3.59324	38.56962	20.76881	5.792088	162.0745	35.88928	11.18132	16.99244
4.617183	5.188026	12.94427	38.00114	8.10105	100.0963	10.88233	14.26061	33.84967
4.549065	5.238842	13.10887	37.48094	8.046562	94.88094	10.621	13.88648	32.38653
4.63695	4.826462	11.60948	38.50239	7.591207	97.99098	10.72278	12.96923	30.32781
4.843735	4.628488	10.76658	36.82702	7.325493	114.9901	10.14639	13.18968	24.43051

For peer review only

5.744208	7.371989	17.15338	119.4231	11.4983		16.2	21.01128	87.80833
5.371823	6.596673	16.02612	133.5233	9.926532		14.26121	18.03359	128.2089
4.455378	6.277556	14.21525	127.8497	9.221117		13.97992	14.53544	113.4483
4.437962	5.747388	12.8892	93.0814	9.156426		12.84375	11.64011	91.83556
2.990061	4.841128	21.25824	31.46169	8.823902	47.11289	19.30034	15.51836	43.81598
3.180417	5.218496	21.11678	34.35121	10.02275	39.63091	19.35026	17.06538	45.88037
2.990432	5.129071	21.99664	34.22257	10.27059	50.57099	20.21364	15.58694	44.14636
2.886747	5.163263	24.399	38.78433	10.45339	60.48153	21.07998	17.56835	43.62906
4.203004	4.506218	16.71339	22.85678	7.61941	65.18813	14.77248	17.84989	34.54789
3.956267	4.333678	15.46687	22.22868	7.589272	53.67073	13.96061	17.26136	31.30657
3.758952	4.196457	14.78111	22.41486	7.22446	49.02084	13.14215	17.41877	29.70039
3.699288	4.273344	14.96552	23.4162	7.283703	49.12709	12.85826	13.9113	29.98136
6.026771	7.072395	16.20266	47.39568	10.70871		19.86132	17.26868	34.17012
5.61115	6.490798	15.20281	44.0069	10.02041		17.67174	15.3095	32.06533
5.091724	6.281033	14.91561	40.95913	10.04024		17.16807	16.89591	29.83122
4.693543	5.858479	14.29268	44.44204	9.507108		15.15392	16.52922	24.82049
6.587633	6.131105	23.50806	75.87853	12.06883		39.65986	19.59664	43.18518
5.512013	6.051973	20.77206	47.51226	10.77045		28.47543	17.94125	47.18163
4.804306	5.480376	16.0212	42.64193	9.762371		26.71425	14.82383	40.19503
3.574721	5.149661	13.34726	42.4115	9.382686	18.56777	13.29127	11.2042	35.0137
5.293217	6.251351	21.42857	37.34359	12.06449	85.46591	19.53553	21.48857	47.58621
5.049881	6.43311	19.03962	33.58249	12.38612	237.2813	18.46139	18.65144	50.75535
4.402477	6.243126	17.67508	31.64952	11.47579		15.69798	15.0735	45.05197
3.966878	5.171048	15.95783	30.44572	10.09779		15.30587	11.65558	48.5954
5.99389	5.962989	21.5861	47.89531	9.740975		16.56777	22.16503	52.78846
6.181541	6.080916	21.95366	55.21975	9.605614		16.91813	19.69792	53.3353
5.512003	5.92727	20.87564	57.82779	9.465473		15.97605	16.30263	44.24841
5.088246	6.025866	21.16679	51.94108	9.047544		15.9492	15.44185	44.40898
6.03047	4.808847	26.18355	30.55416	10.353	1695	25.80833	22.28538	43.89185
5.532719	4.64156	22.54054	31.49871	10.47645	1216.25	23.98398	23.46463	37.98309
5.365339	4.619373	22.96008	29.94804	9.874476	1339.773	21.77295	20.57098	35.96157
5.333285	4.545185	20.84836	33.73352	9.817728	1966	23.0644	19.8356	36.88555
4.282682	6.340561	26.53189	59.52693	10.3865	4481.08	20.68331	19.98467	82.78577
3.989397	5.974408	25.05753	47.87771	11.0186	3770.533	23.19258	15.77467	68.78484
3.720683	5.451533	26.358	45.52005	11.63958	2680.084	22.58499	15.29433	67.60626
3.639434	5.543683	23.37187	52.12297	12.05436	1523.752	17.11364	14.51861	72.9274
3.300239	4.21058	11.71481	36.16039	7.990062		8.396107	13.21199	54.35616
2.88159	3.878563	11.5579	38.60759	7.042985		7.229392	10.68041	43.16038
3.091465	3.816004	11.4018	36.97407	6.315589		7.856807	8.523952	21.32729
3.372087	3.869386	10.32759	34.56276	5.782814		8.4026	7.323255	20.8509
2.885572	4.24829	327.1875	13.37378	8.521009		153.9706	17.80447	17.65599
2.767156	4.172732	454.5	12.86101	7.250839		208.3125	15.04447	16.93626
2.745413	4.144346	406.375	12.98372	7.277064	195.1245	203.1875	14.1321	17.32523
4.033452	3.071445	22.70707	26.19499	4.547335		20.1253	15.81165	40.46281
4.041944	3.160985	20.33552	29.24131	4.815167		17.28707	15.06539	34.75032

For peer review only

4.131827	3.3763	20.27874	28.75911	4.764036		15.43155	13.84923	38.0673
2.56106	4.0746	8.170372	61.81952	6.498904	766	6.030867	9.631686	18.13268
2.414438	4.3086	8.259127	69.66814	6.263147	629.8	5.870493	9.620965	17.94234
2.287753	4.011313	7.762543	59.82188	5.731045	384.125	5.525393	8.631851	17.2636
2.185376	3.956565	7.428059	54.08649	5.296857	383	5.566096	7.651814	16.57557
5.318347	5.040206	24.32136	25.04387	9.308176		17.89873	9.116696	32.06634
4.680672	5.238327	18.53484	25.54234	10.11752	21200	16.55224	9.422571	30.15647
3.738877	4.369957	16.32271	19.71679	9.463755	4577.5	13.28095	10.34427	26.27601
3.697477	4.510525	15.16213	18.67102	8.919115	4617.5	13.33614	10.50837	27.2285
2.396457	5.428076	6.032656	34.46168	5.908949	72.35632	4.963174	7.752782	28.78811
2.134809	4.998084	5.274471	32.20963	5.22959	60.37168	4.390823	6.384542	26.44186
1.765194	3.855434	3.897803	23.94287	4.214616	66.33093	3.429899	5.486344	19.34266
1.640425	3.691074	3.455084	22.97473	3.890467	64.3609	3.041629	4.652156	18.79941
3.36511	5.603925	16.99705	27.52574	10.44373	204.2876	16.76707	19.32146	34.39393
5.121523	7.736146	20.38143	22.90043	11.71758		21.23409	21.15506	33.0238
5.122423	7.248082	19.36663	24.31193	10.79407		21.30678	16.74052	29.90127
4.400497	7.066198	17.26011	24.22584	11.21417		21.34781	17.24221	29.41232
3.844393	6.810869	15.29108	21.61127	10.63892		18.49113	16.97917	28.33419
3.501792	4.032772	20.62463	15.91284	7.44774	919.8684	9.761601	18.82076	18.28721
3.574508	4.10331	19.86215	15.67966	7.219531	1022.264	9.793855	16.2871	17.63074
3.352944	4.097605	19.99391	15.10804	6.891629	1786.25	9.367122	14.61397	17.16362
3.216823	3.885324	19.66343	14.55163	6.531069	1766.75	8.83406	14.75975	15.6266
4.71225	2.727828	19.83218	69.27586	7.940807		16.54519	20.56997	26.45742
3.768758	2.568713	18.40473	68.53571	7.40814	41717.39	16.05018	15.16247	24.71876
3.975924	2.569947	15.92398	70.5	7.553021		16.69241	12.81587	23.36702
4.062437	2.734489	14.99756	70.39414	7.197148		15.85685	12.77162	24.38999
3.056642	3.926122	83.01238	22.23855	6.311034			26.03211	18.67544
2.787314	3.568445	38.28193	19.01949	5.789811	2135.3	153.2251	23.25687	17.26398
3.414193	4.819753	25.91867	22.69666	8.045991	416.8327	32.12338	21.05547	25.02698
3.258451	4.643022	27.08417	21.7528	7.718033	380.3337	30.86374	19.9151	23.89726
2.636185	4.116705	10.313	7.280383	3.90385	407.0811	12.38938	18.55195	9.850219
2.373659	3.743478	8.741962	6.739523	3.595668	501.914	11.79412	19.2343	8.396499
2.138217	3.323964	7.955015	6.36194	3.413356	376.8308	11.28347	20.51022	7.723641
2.050814	3.226228	7.336232	6.157585	3.408978	523.6667	11.46388	17.34794	7.616281
3.884071	3.528022	23.28448	15.77878	6.107791	187.9032	8.630055	31.53194	21.73237
3.674688	3.395687	23.16422	14.40571	6.103552	192.0136	8.331808	29.49528	21.34256
3.633115	3.303045	22.15372	13.50667	5.756198	204.7723	8.119561	25.64553	19.4471
3.343313	3.120416	21.0793	13.16635	5.415563	2184.324	7.777651	24.80044	17.81844
4.18087	5.191731	28.24848	29.56914	8.412643	956.5	16.76232	19.567	36.21277
3.872094	5.075367	27.61616	27.43738	8.01894	1246	18.66576	19.20534	35.99827
3.647131	4.844496	28.1012	26.06112	7.726903	18685	17.98959	22.07813	33.62594
3.480747	4.780844	26.77224	26.29199	7.533489	4672.5	18.25713	18.0567	32.12851
3.779374	8.722346	29.42905	33.93446	11.83562	222.8085	32.64514	9.822971	50.44375
3.672127	8.249946	28.15319	40.39286	11.64409	220.0443	30.98345	8.911198	47.52874
3.774882	6.84771	30.04052	37.32779	11.7661	396.8514	26.12048	9.410765	45.90088
3.6814	7.176028	27.68276	36.69072	11.40754	469.9704	26.75181	9.037614	44.64169
4.133499	4.742393	19.40155	39.78305	8.369352		19.19989	15.6736	30.84971

For peer review only

3.772871	4.634426	18.1677	36.96881	7.832008		17.93583	13.44452	26.25292
3.708378	4.442157	16.55654	42.41824	7.645003		16.45204	12.62815	23.43284
3.606694	4.300023	16.12829	41.76323	7.654642		16.52618	12.7819	13.25852
2.857568	2.808547	17.82363	13.47988	5.486875	186.9108	7.234029	18.6797	13.17379
2.790208	2.575253	15.94473	13.28502	5.156037	588.3406	6.675606	15.00164	12.02032
2.517848	2.413746	15.63057	11.99066	4.751958	546.6234	6.294394	13.44549	10.87479
2.319587	2.301526	14.05806	11.20515	4.566309	1039.16	5.798385	12.02791	10.57463
3.383234	4.044676	16.88864	39.06739	7.109657		7.86194	12.67726	24.84993
3.245841	3.704882	14.59557	44.57143	6.873352	234	7.938973	11.01457	24.27372
3.334547	3.634792	14.16777	41.95522	6.770623	312.3333	7.770606	11.89652	24.54097
3.270429	3.793699	13.32918	39.79054	7.096235		8.215428	12.3738	26.7152
3.96941	5.354947	17.63696	36.94891	8.85992		15.81039	14.46727	37.39433
3.622458	5.449014	18.03868	33.76555	8.694194		14.36351	13.95506	37.08545
3.657653	5.401618	17.82785	35.47802	9.008667		14.61409	14.1941	30.50011
4.014032	5.793887	18.42781	37.86169	9.531805		15.4591	13.41501	33.32193
3.840856	3.683045	22.15638	28.40681	7.017098	197.1196	15.47199	20.48912	30.27041
3.392287	3.530658	22.55823	25.57761	6.783735	212.88	14.8968	21.98859	28.91331
3.012723	3.343477	19.72813	22.86661	6.249475	1062.188	14.45821	21.29339	27.74222
2.787869	3.160715	18.29639	24.06835	6.102463		13.48513	19.88608	25.68852
6.437484	5.560981	18.82152	45.26909	13.41011		19.71569	21.52309	41.49401
5.325982	4.951465	18.65958	44.14667	11.91046		20.06992	20.24982	38.69093
4.530113	4.850997	18.05726	37.64541	10.99118	116.1094	17.78304	17.77018	31.49213
4.165483	5.030645	17.06577	36.57411	10.76641	778.3637	16.29983	15.20275	27.01015
3.848692	3.55896	22.65647	15.88706	6.487237		34.57054	23.50629	29.13708
3.751949	3.477971	23.3404	14.75385	6.417662		33.17398	23.61116	26.27469
3.317065	3.086359	21.57687	13.27783	5.642211		31.04154	19.77539	24.58131
2.960377	2.918906	19.12349	12.2939	5.275155		29.22768	17.22927	23.48621
4.380985	6.745846	16.07884	26.51314	9.117598	244.65	12.1273	15.53255	40.72409
4.033163	6.866786	15.60652	28.06074	9.100419	320.7325	11.2409	15.87954	39.51737
3.5445	6.112318	14.3127	26.54254	8.233529	16275	11.06704	13.74135	39.29578
3.152775	5.470504	13.06855	25.05272	7.586517		9.577552	11.4476	35.17556
3.26922	4.849803	18.06506	28.36272	8.093442		12.15225	18.91304	23.88001
3.083758	4.698121	16.5183	26.89186	7.420507		11.95735	20.85242	21.66205
3.276282	4.624192	16.49844	26.27449	7.23651		11.74279	17.60955	19.35978
3.121272	4.072322	15.03011	25.82258	6.265015		11.16355	13.91956	18.02957
4.248409	6.308469	20.5004	55.48759	11.60377	110.4348	13.3971	16.54037	33.13044
4.007461	5.846789	18.03858	47.09257	11.22643	115.0319	11.91004	15.5827	29.24696
3.979213	5.884227	19.34536	41.90791	10.25422	116.4381	12.26622	15.15102	29.32407
4.011031	5.90612	17.02206	43.56964	10.72514	103.9671	12.56806	14.15003	26.44707
4.268801	5.468577	13.89625	181.375	8.925569		13.09567	12.9515	59.63014
4.547656	6.375127	15.99649	206.8636	9.408725		15.57388	13.27209	54.17857
4.508205	5.685291	14.50955	373.5	9.518603		12.43377	11.89885	54.65854
4.526103	5.516259	15.3782	380.25	10.4183		12.1901	11.19068	66.32268
3.813674	3.525497	11.70315	15.76807	5.075766	39.13231	6.372517	14.9629	24.05302
3.959961	5.315745	12.63416	31.17521	8.094002	1017.907	12.34233	12.31009	29.7519
3.878502	5.063472	12.30067	29.25008	6.895349	2268.421	10.75014	10.46392	31.24
4.205768	5.386378	13.58319	32.56199	7.525187	13002	10.97777	10.86438	35.03237
4.277626	5.361065	14.29259	42.0522	8.475118		11.09439	10.9261	37.9281
3.34207	3.929735	26.48884	28.68427	8.382371		19.68439	20.03458	21.21334

3.193011	3.9542	25.06577	32.73175	8.397495		19.7969	18.99058	18.34367
2.788565	3.985567	21.13664	32.01888	10.08379		20.36371	18.32351	16.99131
2.603481	3.675911	20.23813	28.83878	9.556572		17.80034	17.90763	16.8392
5.054142	4.67973	23.90943	41.77583	8.866606	1056	17.732	19.912	49.61629
5.06268	4.883449	24.19874	46.5175	8.695898	1607.25	18.16722	20.19447	56.0198
5.880238	5.766643	25.94521	57.0692	10.34956		23.18815	21.36187	62.89017
5.838312	5.85804	26.19301	55.5175	10.26787	14751	22.00702	16.96956	61.75687
2.933653	4.022156	11.65769	16.65736	5.885884		14.83393	11.73873	17.56922
2.790468	3.813689	14.18508	16.90663	5.840279	17391	11.6231	11.64846	15.78657
2.865742	4.004907	14.88964	16.48824	5.925564		11.24345	11.89524	16.73159
2.85108	4.025225	15.47508	16.68305	5.743252		10.34771	11.35306	20.22622
4.260504	4.883505	17.85616	23.02778	11.11873		19.18391	16.42532	35.11076
3.93759	5.02937	18.87217	19.65338	10.1765		19.84109	18.7552	35.64455
3.604135	4.940289	16.51704	18.92058	9.014653		21.68188	19.89966	33.58277
3.561493	4.413476	14.25612	20.64728	8.582171		18.42848	19.28106	29.74982
3.75515	5.321061	18.58359	24.56076	10.85519		16.18638	19.91634	39.32945
3.330093	5.019964	16.40629	25.0838	10.11722		14.18641	17.41951	34.04381
3.222611	4.855349	16.91932	28.01828	9.654136		13.47209	16.40809	34.86143
3.048549	4.63799	16.00504	28.48741	9.777753		12.68518	15.09687	30.26127
3.112717	3.776467	15.96763	32.04477	7.510563	306.625	12.24875	13.76801	23.14151
3.39602	4.038733	17.74847	36.30263	8.50886	1034.625	13.22814	14.84656	25.33831
3.208381	3.763036	16.18499	35.68864	7.748146	675.25	12.86637	16.39935	23.25116
3.105498	3.503116	14.40049	34.65338	7.333216	991.875	11.95178	13.06544	22.06298
5.043093	5.689939	25.49854	31.54279	11.48647	44.58262	22.59931	23.18494	40.0788
4.476351	5.47571	21.63207	31.62095	10.87948		20.37493	18.90069	36.15126
4.212382	5.322448	18.71598	35.3952	10.24859		19.74606	17.51165	32.38833
4.024273	5.079923	19.16371	30.81783	9.353315		20.09409	17.17262	33.18325
4.912478	5.099651	16.40287	28.5416	7.719953		17.92651	17.43202	25.86265
4.452562	5.070535	14.31165	25.73819	7.496329		17.54647	16.80447	25.86441
4.625153	4.863594	15.31488	26.08043	7.814826		17.62601	14.31253	29.09071
4.13752	4.547984	14.90621	27.31249	7.373215		17.57436	14.88256	28.6094
5.166548	6.082312	21.08066	39.90794	11.24258		25.76523	19.57161	29.42724
5.349274	5.821069	22.80582	39.07666	10.88255		22.63961	18.56672	25.47416
5.241383	5.997269	19.87206	39.83501	10.17272	224.7931	20.00752	17.45836	24.24715
5.035389	5.404495	18.61977	35.37363	9.043773	74.86047	18.91992	18.49037	24.58654
4.569554	5.551732	18.90169	33.29022	10.50789		16.83658	20.88228	44.18375
4.094433	5.427662	18.52555	33.49884	9.812973		16.4454	22.29298	44.20267
3.683034	5.167603	16.06287	29.63475	8.954732		16.09441	15.57109	41.55313
3.429924	4.601144	15.17977	30.42505	8.824974		15.45856	12.6945	44.68228
3.368552	4.057293	14.7461	18.13485	6.48707	315.1762	7.880605	25.02008	19.79574
3.248929	4.15535	15.04822	19.09184	6.422261	386.3431	8.02627	24.83225	19.59566
3.151222	3.905593	13.84978	17.64346	6.10065	453.7722	7.856951	22.17476	18.66345
3.027006	3.796008	13.16687	16.6298	6.055645	1748.654	7.712885	20.72114	24.44925
4.588066	5.109016	21.43852	47.75333	9.199107	76.40438	16.11372	19.3488	24.18475
4.246009	5.063449	20.25413	53.95471	8.913467	77.57375	15.01225	16.626	24.38084
3.779908	4.793882	20.79239	52.8252	8.369227	80.38145	13.49666	13.22512	21.44132
3.647285	4.786711	20.3173	49.96198	8.16421	78.0594	13.56269	12.32462	20.03176
5.302072	6.084884	25.03789	42.90327	13.55424	133.2737	20.76636	18.01287	42.49311
5.116661	6.049386	24.80952	42.09991	13.43252	145.8653	19.43726	18.76121	41.48015
4.483744	5.359657	21.04037	40.69396	11.82459	131.2627	17.91857	16.45582	35.44415

4.553907	5.215045	19.42717	40.1236	11.41416	124.0961	18.49021	15.93438	32.84266
2.263527	3.147104	14.90157	24.1446	5.474789		11.00516	9.810741	16.49299
2.225053	3.239426	14.5875	23.82142	5.078826		10.2004	8.278932	15.76057
2.321631	3.150794	15.32963	22.87152	5.25626		10.1449	7.859134	14.39668
2.472734	3.184973	15.86443	22.76623	5.287989		10.19109	7.832812	15.12966
3.18314	3.648597	12.86175	26.01798	8.197973	360.9052	12.56076	13.28837	18.30785
2.996321	3.502041	12.83049	24.35921	7.494578	324	11.38755	11.50394	16.6021
2.861852	3.278588	11.84419	29.21221	7.176055	305.5	10.33693	9.641818	16.138
2.815753	3.031742	12.47777	38.63948	7.155366	322.625	10.10024	8.230777	17.46277
4.975393	6.695671	24.61392	33.74172	11.81373	46.15149	25.71241	20.81681	36.54804
4.6642	6.186299	23.70784	36.19165	11.71663	47.68938	26.11887	18.42882	36.08731
3.699483	5.337248	21.39857	28.68938	10.00728	40.78134	20.64468	14.20467	34.92554
3.650116	5.149209	20.56494	27.64474	9.111955	40.2682	21.25658	12.54014	28.864
4.2131	4.349891	25.42169	38.3069	7.01385		17.13126	18.51249	23.29347
4.279727	4.066636	22.78617	38.59189	6.489496		15.56898	15.5505	20.10756
4.252163	3.712855	22.07058	34.91312	5.846483		14.85358	12.19869	17.69679
3.95629	3.672232	22.20707	33.49076	5.663605		13.87026	12.66549	17.5923
4.48476	5.981359	20.95306	49.93739	10.53987	117.1997	15.92924	18.34281	65.23873
4.203565	5.277952	18.95695	49.11302	9.649912	98.90842	14.90141	18.90731	74.85337
4.118693	5.327053	16.30064	49.12466	9.520854	118.987	14.74013	16.73564	58.35066
3.950412	5.156412	14.53869	45.44024	9.234829	387.2134	12.93025	15.62946	55.18903
3.758874	4.554293	16.64018	22.11202	8.220931	1555.869	14.41443	20.18893	43.90128
3.48423	4.179462	15.32066	22.27567	7.703125	1562.352	14.15277	19.06061	38.85647
3.36738	3.816356	14.45824	20.80413	6.803004	1616.402	14.27012	17.02187	34.40467
3.07451	3.484495	11.95811	18.51133	6.054711	16418.73	11.06824	13.18131	30.2444
5.700357	5.904439	17.7783	43.11535	10.22818	42.82954	12.29891	18.94605	36.35692
5.451993	5.859915	18.49959	43.90946	10.01307	44.01923	11.63997	20.48505	36.33333
5.014061	5.758594	17.97494	44.55538	10.03219	88.8336	11.43518	17.24225	36.6711
4.729073	5.449165	17.35224	45.45202	9.926963	382.1	11.27439	14.1614	35.2246
5.357519	4.826866	19.45812	36.65092	8.89781		12.83881	19.2486	35.68589
5.206769	4.844286	19.53901	34.38127	9.320448		13.10319	19.99456	34.62545
4.571171	4.383627	17.91472	31.87492	8.552687		12.14125	14.92006	33.19877
4.202902	4.345558	17.28479	32.84193	8.830113		12.70132	11.78548	33.25113
1.89567	3.807341	10.42069	10.70373	4.916858		7.731413	8.182858	10.07052
1.7702	3.465599	9.40325	9.804196	4.538649		7.158699	8.380899	9.524632
1.884364	3.203519	9.599062	9.891338	4.301498		7.319154	10.58981	9.12261
1.958149	2.960373	9.190599	9.143454	4.217167		7.373696	10.81472	8.923267
4.034811	7.128282	16.67966	40.76139	10.416		20.92707	20.65987	40.19833
4.021182	7.073029	16.96062	37.66689	9.885534		19.89905	20.73681	38.4726
4.165196	6.821621	16.53774	35.30787	9.718778		19.22915	16.77781	37.59804
4.26356	6.376271	16.04692	35.86273	9.576652		18.31657	15.40157	38.10263

For peer review only

ratio_bed_	ratio_bed_	shmi_value	shmi_band	spells	observed	expected	po_ll	po_ul
94.25		0.9955	2	78805	2416	2426.966	0.9384	1.0643
74.25317		1.1093	2	69688	2533	2283.408	0.9366	1.0664
79.64698		1.1332	1	61625	2365	2085	0.9337	1.0695
83.82481		1.1468	1	62685	2430	2120	0.9342	1.069
21.7508	1849	0.9478	2	173890	3486	3677.974	0.9498	1.052
22.30437	1749.923	0.9406	2	177505	3285	3495	0.9485	1.0534
21.6211	1490.4	0.9661	2	173410	3460	3580	0.9491	1.0527
		1.0387	2	80165	3070	2955	0.9441	1.0581
43.47116		1.0652	2	79450	3295	3095	0.9454	1.0568
276.8421		1.056	2	17273	946	895.8156	0.8999	1.1076
278.6111		1.0755	2	17348	957	889.8263	0.8996	1.108
278.6111		1.0291	2	17550	845	820	0.8956	1.1126
275.8333		0.9939	2	18655	875	880	0.8992	1.1085
17.56775	123.2072	0.8796	3	155546	3756	4269.981	0.9534	1.0482
16.10495	108.1061	0.8993	2	161833	3826	4254.272	0.9533	1.0483
16.40847	92.40598	0.9058	2	175470	3695	4080	0.9523	1.0493
15.16824	94.67603	0.9263	2	173410	3610	3895	0.9512	1.0505
25.64475	1817.5	0.8122	3	93118	2528	3112.64	0.9455	1.0566
30.7959	2060.417	0.8257	3	95302	2617	3169.444	0.946	1.0561
30.1348	2139.654	0.8375	3	105010	2530	3020	0.9447	1.0575
30.76923	2057.855	0.8487	3	103985	2595	3055	0.945	1.0572
15.43583	10.5159	0.8926	2	45971	1257	1408.267	0.9197	1.0851
13.94928	8.854098	0.7955	3	48116	1169	1469.594	0.9213	1.0833
13.98123	9.126543	0.7981	3	48905	1200	1500	0.9221	1.0824
13.81918	9.254182	0.7872	3	49730	1265	1610	0.9247	1.0795
	286.75	1.0535	2	16649	967	917.9213	0.9011	1.1063
	256	0.9911	2	16448	920	928.303	0.9016	1.1056
	376.875	0.8845	2	16785	780	880	0.899	1.1086
		1.02926	1.8	17097	936.25	909.1971	0.90055	1.10685
5970		0.9972	2	26979	1075	1077.979	0.9085	1.0977
3460		0.9778	2	28330	1114	1139.347	0.9109	1.095
13340		0.9525	2	29380	1035	1090	0.9089	1.0973
		0.8733	3	30565	1045	1200	0.9131	1.0925
11.55883	17.96034	0.9731	2	76248	1690	1736.802	0.9275	1.0764
11.13372	16.97537	1.0274	2	77553	1796	1748.172	0.9277	1.0761
10.533	17.28401	1.0793	2	83035	1765	1635	0.9254	1.0788
10.15013	17.41516	0.9619	2	98670	2545	2645	0.941	1.0615
22.0525	88.12602	0.8309	3	49983	1850	2226.563	0.9358	1.0672
23.16659	139.3407	0.9334	2	46519	1777	1903.709	0.9307	1.0729
23.25308	113.9568	0.9225	2	46315	1680	1825	0.9292	1.0745
20.70594	113.5655	0.992	2	45880	1750	1760	0.928	1.0758
25.2246		0.9577	2	75923	1850	1931.747	0.9312	1.0723
27.39439		0.9375	2	87226	1955	2085.424	0.9337	1.0695
27.20153		0.9199	2	97625	1885	2050	0.9332	1.0701
23.13035		0.9542	2	93540	1850	1940	0.9313	1.0722
60.40999	161.3935	1.1935	1	54495	2468	2067.942	0.9334	1.0698
73.72652	187.5	1.0992	2	56124	2458	2236.216	0.9359	1.0671
64.46287	95.85714	1.0677	2	57660	2235	2095	0.9338	1.0694
56.70737	110.5091	1.0803	2	190305	6510	6025	0.9607	1.0405

For peer review only

40.18509	148.1238	0.8736	3	98735	2758	3157.188	0.9459	1.0562
44.60941	167.3011	0.8496	3	103587	2796	3290.862	0.947	1.055
43.97687	184.2462	0.837	3	105390	2715	3245	0.9466	1.0555
40.83826	141.0891	0.8302	3	111895	2670	3215	0.9464	1.0557
	27.60782	0.8488	3	45508	1214	1430.327	0.9203	1.0844
	27.14552	0.8809	3	46837	1335	1515.449	0.9225	1.082
	29.01853	0.8471	3	49015	1270	1500	0.9221	1.0824
	34.76699	0.8972	2	45930	1310	1460	0.9211	1.0835
157.5833		0.9489	2	38995	992	1045.4	0.9071	1.0993
154.1667		0.9366	2	41351	1082	1155.254	0.9115	1.0943
168.6104		0.9883	2	40020	1035	1050	0.9073	1.0992
163.4508		0.9754	2	40865	1085	1115	0.91	1.0961
39.92051	148.125	0.842	3	54374	1179	1400.294	0.9194	1.0854
38.09846	151.7857	0.8327	3	56838	1247	1497.617	0.922	1.0825
33.64643	142.3214	0.777	3	59135	1140	1465	0.9212	1.0834
32.77119	144.8214	0.7754	3	62305	1200	1545	0.9232	1.0811
30.03443	59.76246	0.9522	2	54622	1760	1848.399	0.9297	1.074
28.94521	74.99352	0.9168	2	58108	1859	2027.785	0.9328	1.0705
30.26335	58.0771	0.9442	2	58740	1840	1950	0.9314	1.072
		0.95702	2	55216	1671.875	1741.539	0.927325	1.076525
81.46789	737.2063	1.1161	2	31234	1143	1024.148	0.9062	1.1004
72.37113	877.5	1.1703	1	30214	1168	997.9934	0.905	1.1017
93.56121	441.375	1.1888	1	29255	1075	905	0.9004	1.1071
111.1179	301.5	1.1289	2	28940	1140	1010	0.9057	1.101
229.4294	3451.875	1.0552	2	44498	1626	1540.939	0.9231	1.0812
162.6342	973.8889	1.107	2	45473	1682	1519.436	0.9226	1.0818
126.4174	1043.03	1.1093	2	45410	1500	1350	0.918	1.087
142.946	2350.667	1.0822	2	51590	1555	1440	0.9205	1.0842
62.12264		0.9561	2	66706	2254	2357.404	0.9376	1.0653
62.45445		0.947	2	67745	2259	2385.442	0.9379	1.0649
66.54298		1.0749	2	65300	2190	2035	0.9329	1.0704
61.98847		1.147	1	64145	2285	1990	0.9322	1.0712
156.6471		1.0277	2	73135	2326	2263.274	0.9363	1.0667
143.6979	4138.5	1.0245	2	76501	2435	2376.657	0.9378	1.065
122.2124		1.036	2	80215	2305	2225	0.9358	1.0673
233.6667	4206	1.0754	2	78130	2445	2275	0.9365	1.0665
		1.0385	2	48857	1564	1505.996	0.9223	1.0822
		1.0439	2	49346	1627	1558.54	0.9235	1.0808
		1.0095	2	51210	1425	1415	0.9198	1.085
		0.9947	2	52235	1555	1560	0.9236	1.0807
140.2869	172.8788	1.0359	2	28114	1084	1046.39	0.9072	1.0993
151.2617	163.4849	1.0677	2	30283	1140	1067.737	0.9081	1.0982
45.0042	152.4242	0.9455	2	31350	965	1025	0.9062	1.1004
36.63876	158.4849	1.0271	2	29295	1005	980	0.9041	1.1028
81.03627	53.31818	1.0609	2	34551	1209	1139.584	0.911	1.095
88.59689	47.82738	1.0134	2	36539	1227	1210.787	0.9135	1.092
112.7027	48.03456	1.1321	2	36495	1270	1120	0.9102	1.0958
					675	786.3953	0.893233	1.115067
22.20451		1.0871	2	75474	1793	1649.348	0.9256	1.0785
22.66519		1.0458	2	77527	1850	1769.042	0.9281	1.0757

For peer review only - http://bmjopen.bmj.com/site/about/guidelines.xhtml

21.95304		1.0304	2	81200	1695	1645	0.9256	1.0785
20.87954	781.9949	1.0337	2	119520	3065	2965	0.9442	1.058
49.52688	94.16197	0.9743	2	86848	3211	3295.618	0.947	1.055
48.15415	144.9	0.9902	2	86990	3193	3224.698	0.9465	1.0556
46.04408	871	0.997	2	89680	3050	3060	0.9451	1.0571
44.4048	706.7935	0.9862	2	92945	3245	3290	0.947	1.0551
209.0182		0.9372	2	26261	940	1003.025	0.9053	1.1015
211.2145		0.9208	2	27046	942	1022.994	0.9062	1.1004
221.2157		0.9294	2	27105	895	965	0.9034	1.1036
476.3966		0.967	2	28230	980	1015	0.9057	1.1009
348.75		0.9594	2	34885	1052	1096.563	0.9093	1.0969
349.25		0.9408	2	32537	1011	1074.575	0.9084	1.0979
312		0.9233	2	33230	1000	1080	0.9087	1.0976
337.5		0.8927	2	32955	1030	1155	0.9115	1.0944
84.32658	96.00969	0.9814	2	46557	1676	1707.82	0.9269	1.0771
91.54825	180.3514	0.9869	2	47400	1796	1819.868	0.9291	1.0746
86.32726	144.6849	1.0136	2	48690	1685	1665	0.9259	1.0781
87.72554	128.5714	1.0147	2	49615	1675	1650	0.9256	1.0785
73.74013	43.94125	1.0181	2	56032	2048	2011.498	0.9325	1.0708
67.72059	37.27802	0.9844	2	57341	1998	2029.704	0.9328	1.0705
62.34659	56.86419	0.9923	2	61455	1900	1915	0.9309	1.0726
57.42781	62.59342	1.0507	2	63300	2080	1980	0.932	1.0714
33.52572	35.46867	0.8345	3	51064	1625	1947.321	0.9314	1.072
32.68132	34.31797	0.872	3	50633	1629	1868.142	0.93	1.0736
33.78257	33.74371	0.9106	2	51655	1590	1750	0.9277	1.0761
31.59134	32.21323	0.8575	3	50340	1550	1810	0.9289	1.0748
143.2048	1698	1.0128	2	42210	1237	1221.364	0.9139	1.0916
148.6634		0.9309	2	44430	1294	1390.113	0.9192	1.0857
158.0088		1.0169	2	50245	1310	1290	0.9161	1.0891
222.3246		1.1748	1	41185	1370	1165	0.912	1.0938
203.806		1.0593	2	55128	2225	2100.405	0.9339	1.0693
340.4762		1.0424	2	69039	2271	2178.66	0.9351	1.068
331.5234		1.0036	2	79520	2120	2115	0.9341	1.0691
		1.00296	2	62410	2061.25	1987.808	0.93195	1.071538
163.8923	262.0436	1.0876	2	56603	2330	2142.342	0.9346	1.0686
252.266	91.1189	1.1088	2	106486	4619	4165.867	0.9528	1.0488
78.52589	36.46321	1.1167	2	109845	4440	3975	0.9517	1.05
72.52081	37.10679	1.0752	2	110015	4400	4090	0.9524	1.0493
155.2264	563.25	0.8935	3	127183	3552	3975.518	0.9517	1.05
165.3806	521.5	0.9214	2	125989	3603	3910.148	0.9513	1.0504
160.1036	441.4286	0.9461	2	130935	3525	3725	0.9501	1.0517
164.0043	425.5	1.0095	2	122025	3740	3705	0.95	1.0518
208.4044		0.9372	2	48988	2047	2184.114	0.9352	1.0679
193.9092		0.9806	2	49162	2146	2188.567	0.9352	1.0678
186.9007		0.9318	2	50170	1990	2135	0.9345	1.0687
233.6082		0.8556	3	51875	2110	2465	0.9389	1.0638
		1.134	1	24017	1141	1006.204	0.9054	1.1013
		1.2321	1	22673	1181	958.4917	0.9032	1.1039
		1.180425	1	23163.25	1151.429	1004.254	0.905214	1.101514
48.39934	44.03379	0.9388	2	69635	2349	2502.047	0.9394	1.0633

For peer review only

53.73779	48.67466	0.9912	2	68974	2577	2599.846	0.9405	1.0621
47.92324	56.46455	0.9509	2	72830	2320	2440	0.9386	1.0641
44.77974	37.3064	0.9123	2	69135	2305	2530	0.9397	1.063
33.89204		1.0579	2	47557	2114	1998.276	0.9323	1.0711
33.64159		1.0002	2	53679	2190	2189.607	0.9353	1.0678
30.85265		0.9351	2	62600	2005	2140	0.9346	1.0686
24.2293		1.0272	2	116195	4195	4085	0.9523	1.0493
96.98523	60.60261	1.046	2	83703	3327	3180.538	0.9461	1.056
87.82248	56.61498	1.0246	2	86815	3310	3230.65	0.9465	1.0556
96.03878	51.72054	0.9638	2	85575	2930	3040	0.9449	1.0573
97.9661	50.50486	1.0141	2	83820	2980	2935	0.9439	1.0583
134.0407		1.0147	2	41484	1495	1473.367	0.9214	1.0832
166.2245		1.0015	2	43239	1522	1519.721	0.9226	1.0818
160.2646		0.993	2	45885	1565	1575	0.9239	1.0803
218.466		1.0345	2	48315	1625	1570	0.9239	1.0804
31.16947		1.1181	2	41047	1537	1374.681	0.9187	1.0862
29.5919		1.0656	2	40127	1457	1367.366	0.9185	1.0864
27.89468		1.127	2	36725	1515	1345	0.9178	1.0872
27.51406		1.1997	1	34435	1490	1245	0.9147	1.0908
50.90073		1.0124	2	50057	1938	1914.17	0.9309	1.0727
61.11111		0.9923	2	50843	1944	1959.011	0.9316	1.0718
57.08817		0.9983	2	51145	1770	1775	0.9282	1.0756
66.64647		0.988	2	51225	1830	1850	0.9297	1.0739
40.57988		1.0737	2	75034	2822	2628.385	0.9408	1.0618
50.39539		1.0636	2	77966	2870	2698.288	0.9416	1.0609
57.94063	382.4082	1.0656	2	81200	2760	2590	0.9404	1.0622
65.33273	219.8309	1.1521	1	82390	3030	2630	0.9408	1.0617
289.026		1.1823	1	31019	1649	1394.777	0.9193	1.0855
253.7569		1.1639	1	31500	1692	1453.705	0.9209	1.0837
202.6138		1.0852	2	33520	1660	1530	0.9229	1.0815
214.2288		1.0894	2	32930	1635	1505	0.9222	1.0823
39.57509	42.66788	0.9918	2	53587	1973	1989.218	0.9321	1.0712
		0.988167	2	52921	1827.333	1801.195	0.928633	1.075117
		0.988167	2	52921	1827.333	1801.195	0.928633	1.075117
		0.9025	2	37188	1447	1603.412	0.9246	1.0796
		0.8685	3	36529	1486	1711.002	0.927	1.077
		0.9082	2	37150	1420	1560	0.9236	1.0807
		0.9353	2	36505	1560	1665	0.926	1.0781
46.97451	28.3452	0.8588	3	74619	2320	2701.502	0.9416	1.0609
45.4928	25.08102	0.8687	3	75061	2355	2711.034	0.9417	1.0608
36.94717	25.11235	0.8513	3	76110	2145	2515	0.9395	1.0631
38.22375	29.92884	0.8575	3	73415	2235	2605	0.9406	1.062
51.92981	61.87153	1.0582	2	61575	2098	1982.533	0.932	1.0714
54.51308	50.30845	1.1278	1	61291	2371	2102.4	0.934	1.0692
51.36239	43.54969	1.0324	2	62905	2165	2100	0.9339	1.0693
51.30012	42.21093	1.0852	2	62975	2350	2165	0.9349	1.0682
65.28898	44.46539	0.9519	2	95440	2964	3113.612	0.9455	1.0566
61.62325	43.92848	0.9348	2	95868	3071	3285.046	0.9469	1.0551
61.57895	39.14179	0.9392	2	96760	2860	3045	0.945	1.0573
58.92232	37.33933	0.8538	3	81560	2200	2575	0.9402	1.0624

For peer review only

24.39477	34.35719	0.9826	2	114317	3725	3791.116	0.9506	1.0512
23.35261	35.27934	0.9617	2	115022	3645	3790.229	0.9506	1.0512
21.40913	32.27488	0.9747	2	114845	3530	3620	0.9494	1.0524
19.1431	29.3601	1.0015	2	115840	3630	3625	0.9495	1.0524
37.82408	31.96206	1.0889	2	81581	3160	2901.999	0.9436	1.0587
34.95147	32.0185	1.0377	2	79217	3107	2994.024	0.9445	1.0578
34.54198	30.44998	1.0211	2	83385	2985	2920	0.9438	1.0585
36.77077	33.17286	1.0501	2	83595	3215	3065	0.9451	1.0571
112.1958	31.1534	1.0274	2	66728	2159	2101.416	0.9339	1.0693
102.7609	32.57538	1.0438	2	67313	2072	1985.128	0.9321	1.0713
100.6632	35.39423	1.0792	2	68795	2010	1860	0.9299	1.0737
94.49399	36.37314	1.1129	2	67945	2190	1970	0.9318	1.0716
10.13828	22.58917	0.7646	3	85307	1491	1950.086	0.9315	1.072
9.995308	21.63953	0.6994	3	90683	1441	2060.296	0.9333	1.07
9.614011	18.07368	0.7126	3	93985	1425	2000	0.9323	1.0711
8.624079	16.66919	0.7595	3	95685	1515	1995	0.9323	1.0711
	1002.222	1.0044	2	88557	2377	2366.518	0.9377	1.0652
	1238.276	0.9652	2	91834	2525	2615.999	0.9407	1.0619
	1011.268	0.958	2	91320	2305	2405	0.9382	1.0646
	575.0794	0.9516	2	91085	2415	2540	0.9398	1.0628
51.8883		0.8855	3	43548	1278	1443.193	0.9206	1.084
52.66358		0.936	2	42528	1345	1437.014	0.9205	1.0842
45.96706		0.91	2	37280	1225	1345	0.9179	1.0872
48.3978		0.9927	2	34915	1315	1325	0.9172	1.0878
26.3807	451	0.8368	3	87335	2003	2393.544	0.938	1.0648
22.10893	382.8671	0.8413	3	89335	2109	2506.892	0.9394	1.0633
20.03577	283.1667	0.8185	3	88960	1930	2355	0.9376	1.0653
15.76721	279.5833	0.886	3	85805	1975	2230	0.9358	1.0672
229.9238	230.76	1.0643	2	33830	1226	1151.942	0.9114	1.0944
383.6744	263.1818	1.0051	2	35117	1293	1286.382	0.9161	1.0892
261.9564	276.9	0.9878	2	36515	1170	1185	0.9126	1.093
203.3458	270.45	0.9746	2	35695	1225	1255	0.915	1.0903
60.7539	71.06053	1.0188	2	128499	4404	4322.67	0.9537	1.0479
64.42136	78.66164	1.0666	2	128542	4507	4225.553	0.9531	1.0485
59.77344	74.28991	1.0115	2	145845	4395	4345	0.9538	1.0478
60.00649	71.78195	0.9719	2	144835	4390	4520	0.9547	1.0468
340.7403	209.0909	0.9901	2	43079	1301	1313.973	0.9169	1.0882
		0.969467	2	42045.33	1301.667	1321.19	0.917083	1.088167
		0.969467	2	42045.33	1301.667	1321.19	0.917083	1.088167
761.5643		1.1368	1	55301	2513	2210.504	0.9356	1.0675
14504.95		1.1732	1	55565	2573	2193.201	0.9353	1.0678
15159.52		1.1578	1	59010	2475	2140	0.9345	1.0686
5357.836		1.1063	2	59075	2450	2215	0.9356	1.0674
707.6645		1.0563	2	20228	939	888.9889	0.8996	1.108
648.351		1.1186	2	19524	982	877.8595	0.8989	1.1088
3374.895		1.1362	2	19715	905	795	0.894	1.1145
		1.1577	1	19635	940	815	0.8952	1.1131
34.87677		1.0992	2	48686	1549	1409.155	0.9197	1.0851
41.70591		1.0364	2	47032	1462	1410.671	0.9197	1.085
45.51883		1.1055	2	48370	1545	1400	0.9194	1.0854

For peer review only

57.0214		1.0752	2	47845	1540	1430	0.9203	1.0844
13.10072	685.125	0.9231	2	109694	3209	3476.205	0.9484	1.0535
13.49601	1006.765	0.9426	2	110748	3235	3431.981	0.9481	1.0539
12.97209	1213.714	0.9421	2	113710	3130	3320	0.9472	1.0548
12.76967	703.8	0.9686	2	109190	3125	3230	0.9465	1.0556
543.2647		0.9875	2	56588	2052	2077.932	0.9336	1.0697
518.9429		0.9878	2	54071	1992	2016.518	0.9326	1.0707
541.265		0.9502	2	56835	1900	1995	0.9323	1.0711
564.7527		0.9655	2	60040	2105	2180	0.9351	1.068
55.10156	37.48888	0.9951	2	72166	2426	2438.041	0.9386	1.0642
60.22513	36.78961	1.0728	2	72439	2639	2459.997	0.9389	1.0639
59.91757	32.25448	1.139	1	72370	2555	2245	0.936	1.067
50.08128	33.53398	1.0761	2	74695	2570	2390	0.938	1.0649
78.12523	60.56131	1.0972	2	96262	2871	2616.667	0.9407	1.0619
64.74964	57.14374	1.1333	1	98181	3063	2702.702	0.9416	1.0609
56.53465	59.15437	1.0973	2	98935	2860	2605	0.9406	1.062
59.01893	66.60635	1.1086	2	97295	2920	2635	0.9409	1.0617
		0.7075	3	27718	548	774.5577	0.8926	1.1161
		0.7573	3	27169	610	805.4418	0.8946	1.1137
		0.7679	3	26060	530	695	0.8867	1.1231
		0.9159	2	23940	610	665	0.8843	1.1258
68.33682	65.92731	1.151	1	69524	2572	2234.669	0.9359	1.0671
57.52781	50.68788	1.2194	1	67757	2663	2183.806	0.9352	1.0679
62.55217	47.96428	1.1749	1	69535	2680	2280	0.9365	1.0664
60.25348	36.76071	1.031	2	70265	2590	2515	0.9395	1.0632
29.06804		1.0126	2	56627	2133	2106.509	0.934	1.0692
28.1962		0.993	2	56653	2030	2044.314	0.933	1.0702
		1.003525	2	57585.25	2023.857	2010.022	0.932486	1.070914
87.54237		1.2123	1	28281	1284	1059.115	0.9077	1.0986
86.51163		1.1211	2	28877	1257	1121.225	0.9102	1.0958
118.3399		1.014	2	29750	1175	1160	0.9117	1.0941
123.4034		1.0481	2	32880	1260	1200	0.9132	1.0924
245.625		1.0816	2	21670	987	912.5237	0.9008	1.1066
241.4583		1.1225	2	21454	1033	920.2723	0.9012	1.1061
252.5		1.1212	2	24180	1090	975	0.9039	1.103
260.4167		1.1593	1	23505	1160	1000	0.9051	1.1016
72.64912	59.61652	1.0752	2	89341	3546	3297.867	0.9471	1.055
66.05121	43.96062	1.0639	2	88397	3604	3387.473	0.9477	1.0542
58.79023	43.08622	1.082	2	90590	3420	3160	0.9459	1.0562
59.02405	43.42968	1.1277	1	88545	3630	3215	0.9464	1.0557
45.50581	1176	0.955	2	65778	1968	2060.799	0.9333	1.07
		0.98	2	64465.67	1800.833	1849	0.9295	1.074067
286.0891		0.9197	2	48011	1889	2053.853	0.9332	1.0701
333.3553		0.9	2	49091	1878	2086.731	0.9337	1.0695
233.5052		0.956	2	50050	1745	1825	0.9292	1.0744
168.3425		0.9272	2	49630	1690	1825	0.9292	1.0745
280.6885		1.0808	2	64950	1792	1658.05	0.9258	1.0782
279.9291		1.0865	2	60918	1782	1640.107	0.9254	1.0787
254.5361		1.1629	1	60800	1740	1495	0.922	1.0825
240.995		1.1719	1	60080	1725	1475	0.9214	1.0831

For peer review only

131.9405		1.0876	2	33940	1402	1289.085	0.9161	1.0891
97.59515		1.1501	1	33645	1564	1359.883	0.9183	1.0867
97.38915		1.2058	1	33485	1385	1150	0.9113	1.0946
91.75837		1.0566	2	36395	1420	1345	0.9178	1.0872
81.48274		0.9501	2	57317	1748	1839.897	0.9295	1.0742
88.36488		0.9084	2	61217	1862	2049.711	0.9331	1.0702
87.78207		0.8856	3	63630	1680	1895	0.9305	1.073
100.409		0.9891	2	61590	1875	1895	0.9305	1.073
149.1556	99.65708	1.0466	2	62439	1995	1906.136	0.9307	1.0728
225.982	78.37934	1.0058	2	62356	2128	2115.796	0.9342	1.069
239.5021	63.66177	1.0097	2	64550	2025	2005	0.9324	1.071
235.1613	66.62605	1.0313	2	68725	2245	2175	0.9351	1.068
		1.0792	2	54270	1497	1387.183	0.9191	1.0858
		1.0871	2	55792	1631	1500.318	0.9221	1.0824
		1.0575	2	57880	1510	1430	0.9202	1.0845
		1.0708	2	57230	1535	1430	0.9203	1.0844
48.54288	243.7631	0.9553	2	46533	1815	1899.985	0.9306	1.0729
50.91619	178.8918	0.983	2	46879	1930	1963.352	0.9317	1.0717
42.40986	159.2401	0.9499	2	46720	1770	1860	0.9299	1.0737
		0.9787	2	46991.4	1769.75	1761.038	0.92775	1.076088
		1.0206	2	46180	1935	1895	0.9305	1.073
		1.0206	2	46180	1935	1895	0.9305	1.073
		1.0206	2	46180	1935	1895	0.9305	1.073
27.37643	129.0909	1.0206	2	46180	1935	1895	0.9305	1.073
95.93112		1.0459	2	52059	1672	1598.611	0.9245	1.0797
91.7029		1.0885	2	51001	1735	1593.997	0.9244	1.0798
97.83858	1430.4	1.0688	2	52645	1735	1625	0.9251	1.0791
94.73684	555	1.0128	2	49845	1685	1665	0.926	1.0781
39.12695	66.69172	0.9889	2	70717	1924	1945.511	0.9314	1.0721
40.49664	59.05121	1.0036	2	69912	2020	2012.725	0.9325	1.0708
41.27273	66.4702	0.9894	2	74075	1900	1920	0.931	1.0725
46.29062	62.99257	1.0011	2	74195	2130	2125	0.9343	1.0689
48.4545		1.0567	2	35048	1104	1044.798	0.9071	1.0993
49.62753		1.0581	2	36403	1099	1038.619	0.9068	1.0996
38.86775		1.0139	2	37400	1030	1015	0.9058	1.1009
34.33335		1.006	2	38355	1030	1025	0.9062	1.1004
103.1656		1.0361	2	75992	2878	2777.73	0.9424	1.06
99.04646		1.0302	2	73594	2820	2737.443	0.942	1.0605
97.19959		1.0618	2	73675	2655	2500	0.9393	1.0634
102.3534		1.0961	2	72625	2830	2585	0.9403	1.0623
107.0504		1.0621	2	54275	1885	1774.838	0.9283	1.0755
101.6667		1.0676	2	57670	2028	1899.577	0.9306	1.073
119.011		1.0934	2	56060	1905	1740	0.9276	1.0763
124.8571		1.0875	2	56160	1945	1790	0.9286	1.0752
17.9345		1.0473	2	45593	2037	1944.991	0.9314	1.0721
16.16677		1.0502	2	48332	2111	2010.15	0.9325	1.0709
17.29985		1.0751	2	50410	1945	1810	0.929	1.0748
		1.0438	2	48767	1959.625	1860.907	0.929788	1.073863
44.64522	422.0833	1.0755	2	55814	1935	1799.153	0.9287	1.075
31.49261	279.1703	1.0973	2	57253	2102	1915.662	0.9309	1.0726

For peer review only

36.31844		1.1331	1	55915	2055	1815	0.929	1.0747
		1.08104	1.8	55481	1864.375	1725.922	0.927113	1.076788
107.3832	107.0082	0.8357	3	89719	1613	1930.131	0.9311	1.0724
115.7012	104.5022	0.7941	3	92147	1607	2023.623	0.9327	1.0706
113.9133	90.87235	0.7665	3	94100	1570	2050	0.9332	1.0701
116.6942	94.37372	0.7723	3	97610	1660	2150	0.9347	1.0684
		1.03345	2	22900.5	733.2	733.6453	0.88966	1.11942
48.67776	252.621	1.0791	2	45974	1545	1431.696	0.9203	1.0844
51.60816	222.0953	1.1366	1	47193	1626	1430.538	0.9203	1.0844
41.46088	154.2978	1.1336	1	54740	1600	1410	0.9197	1.0851
38.58331	172.6168	1.0745	2	56335	1630	1515	0.9225	1.082
47.56927		0.8103	3	43763	569	702.2488	0.8874	1.1222
48.45171		0.7764	3	44120	550	708.4386	0.8879	1.1216
37.58152		0.7708	3	45740	525	680	0.8858	1.1242
40.63291		0.7718	3	44980	530	690	0.8864	1.1234
450.3333	1351	0.9586	2	186823	4574	4771.676	0.9559	1.0455
		0.967267	2	180617.7	4416.334	4364.927	0.9539	1.047717
	63480	1.0021	2	34649	1517	1513.87	0.9225	1.082
	9422.223	1.0315	2	33298	1555	1507.506	0.9223	1.0822
		1.0484	2	32970	1400	1335	0.9176	1.0875
		1.0596	2	32585	1480	1395	0.9193	1.0855
18.1679	66.10911	0.9673	2	131817	4153	4293.326	0.9535	1.0481
18.79985	53.69989	1.0585	2	125839	4275	4038.651	0.9521	1.0496
18.55372	50.29624	1.0775	2	125170	4100	3805	0.9507	1.0511
18.76008	45.86479	1.0646	2	119635	4105	3855	0.951	1.0508
119.2713		1.2035	1	40087	1838	1527.171	0.9228	1.0816
106.3475		1.1345	1	45358	1906	1680.063	0.9263	1.0777
105.8601		1.1434	1	47295	1760	1540	0.9231	1.0813
111.6066		1.1814	1	45385	1775	1505	0.9222	1.0823
38.79106	23.67049	1.0434	2	65756	2718	2604.913	0.9405	1.062
36.92701	25.33889	0.9839	2	256713	7468	7590.561	0.9649	1.036
75.03192	56.15413	0.9822	2	274835	7240	7375	0.9644	1.0365
74.52143	56.58429	0.9825	2	263360	7445	7575	0.9649	1.036
8.621808	43.49905	0.7771	3	72191	1185	1524.961	0.9227	1.0817
7.931907	38.33394	0.7357	3	70535	1167	1586.257	0.9242	1.08
7.435247	37.6348	0.7635	3	70355	1145	1500	0.9221	1.0824
7.242372	30.40523	0.6851	3	117990	1335	1945	0.9314	1.072
15.28763	45.38961	0.9519	2	103163	2674	2809.223	0.9427	1.0597
14.62588	46.44578	0.9359	2	100773	2596	2773.944	0.9424	1.0601
13.99414	44.15923	0.9644	2	102560	2510	2605	0.9405	1.0621
12.46678	43.97769	0.9791	2	115585	2665	2720	0.9418	1.0607
51.03995	49.34397	1.1148	2	87856	3188	2859.719	0.9432	1.0591
49.26753	52.99522	1.0723	2	90593	3091	2882.613	0.9434	1.0589
41.2146	53.03292	1.0462	2	90035	2900	2770	0.9423	1.0601
38.2877	49.0783	1.08	2	92955	3105	2875	0.9433	1.059
80.64214		1.0169	2	66964	3105	3053.331	0.945	1.0572
85.31036		1.0147	2	70892	3263	3215.711	0.9464	1.0557
84.13523		1.0273	2	74510	3085	3005	0.9446	1.0577
75.87479		1.0071	2	84145	3280	3255	0.9467	1.0553
40.12308	36.96378	1.0181	2	88088	3323	3263.779	0.9468	1.0553

For peer review only

37.7438	31.38169	0.9661	2	130260	4672	4836.155	0.9562	1.0452
45.97721	38.14141	0.9657	2	130825	4375	4530	0.9547	1.0468
46.42679	39.97758	0.8922	2	135780	4655	5220	0.9578	1.0435
40.67067	34.73473	0.9357	2	118570	3257	3480.939	0.9484	1.0535
40.01405	31.16067	0.9202	2	115220	3205	3483.013	0.9485	1.0535
38.96858	28.59611	0.9153	2	118520	3095	3380	0.9477	1.0543
32.05845	26.13673	0.9137	2	119520	3230	3535	0.9488	1.0531
45.33175		0.9885	2	42579	1839	1860.442	0.9299	1.0737
43.18737		0.9453	2	42039	1734	1834.372	0.9294	1.0743
39.24525		1.0031	2	41810	1670	1665	0.9259	1.0781
38.14119		0.9695	2	44750	1650	1700	0.9268	1.0772
61.40666		0.9491	2	49238	1929	2032.39	0.9329	1.0705
58.75289		0.955	2	51756	1996	2089.997	0.9338	1.0695
65.06146		0.9787	2	53500	1905	1945	0.9314	1.072
72.54435		0.9522	2	53835	1910	2010	0.9325	1.0709
38.88849	55.45872	1.0377	2	89029	2911	2805.346	0.9427	1.0597
36.19177	46.26318	1.1068	2	87930	2914	2632.806	0.9409	1.0617
33.2211	43.01256	1.0779	2	86825	2640	2450	0.9387	1.064
28.87363	42.05837	1.1299	1	83915	2685	2380	0.9378	1.065
96.59722		1.0541	2	50749	1910	1811.895	0.929	1.0747
87.25243		0.9856	2	49305	1795	1821.213	0.9291	1.0745
86.48558		1.0024	2	52110	1805	1800	0.9287	1.075
71.82356		1.0126	2	51030	1890	1865	0.93	1.0736
1606.286		0.9719	2	81268	2291	2357.27	0.9376	1.0653
		0.9419	2	83788	2367	2513.071	0.9395	1.0632
		0.9194	2	89165	2250	2445	0.9387	1.0641
		0.8901	2	91680	2280	2565	0.9401	1.0625
254.8438		0.9656	2	62464	1976	2046.409	0.9331	1.0702
229.9315		0.9524	2	63612	1989	2088.355	0.9337	1.0695
211.3636		0.9848	2	62900	1920	1950	0.9315	1.0719
189.8975		0.9155	2	61965	1810	1975	0.9319	1.0715
44.45496		1.0087	2	106314	4203	4166.912	0.9528	1.0488
43.06193		1.0233	2	105724	4208	4112.182	0.9525	1.0491
41.33145		1.0901	2	108120	4015	3685	0.9499	1.052
37.90331		1.0687	2	108850	4085	3820	0.9508	1.051
		1.0942	2	59266	2095	1914.576	0.9309	1.0727
1204		1.0485	2	57945	2121	2022.924	0.9327	1.0706
1224.264		0.9711	2	60840	2015	2075	0.9335	1.0697
1157.5		0.9734	2	58655	2040	2095	0.9339	1.0693
195.7284		1.1869	1	32311	1392	1172.779	0.9122	1.0936
177.4961		1.18	1	30094	1381	1170.356	0.9121	1.0937
172.7833		1.0191	2	37530	1340	1315	0.9169	1.0883
185.9413		1.0377	2	39205	1440	1390	0.9191	1.0857
16.79329	1134.25	1.1152	2	106097	1752	1571.025	0.9238	1.0804
		1.0438	2	104864.3	1804.333	1722.348	0.927033	1.07675
45.19554	65655	1.0738	2	114699	3931	3660.974	0.9497	1.0521
45.24934		0.9967	2	122002	3959	3972.078	0.9517	1.05
41.54989		1.0162	2	130730	3785	3725	0.9501	1.0517
46.96061		1.0628	2	126570	3985	3750	0.9503	1.0515
41.24345	50.16495	1.102	2	83586	3505	3180.653	0.9461	1.056

For peer review only

41.58593	80.69436	1.0825	2	85112	3454	3190.757	0.9462	1.0559
39.79381	84.67567	1.0667	2	85055	3335	3125	0.9457	1.0565
34.70806	131.4066	1.043	2	84070	3405	3265	0.9468	1.0553
65.15572	70.53401	1.1257	1	83270	3760	3340.018	0.9474	1.0546
64.649	77.05169	1.1544	1	81182	3761	3258.099	0.9467	1.0553
67.6553	83.60642	1.0991	2	79920	3490	3175	0.946	1.0561
82.12285	101.9067	1.0756	2	82225	3605	3355	0.9475	1.0545
79.0635	46.69995	1.0116	2	150993	4587	4534.6	0.9547	1.0467
84.09574	53.18349	0.9477	2	157584	4444	4689.059	0.9555	1.046
80.5305	50.78692	0.9989	2	157045	4440	4445	0.9543	1.0472
77.47513	61.1836	0.9577	2	157790	4505	4700	0.9555	1.0459
	36.25932	1.0717	2	65303	2420	2258.01	0.9362	1.0668
	46.2635	1.0371	2	70304	2482	2393.126	0.938	1.0648
	40.29741	1.0224	2	76215	2355	2300	0.9368	1.0661
	36.61185	0.9989	2	77880	2375	2375	0.9378	1.065
136.723	231.2571	0.9194	2	62488	2041	2220.041	0.9357	1.0673
138.866	260.7097	0.9645	2	61361	2133	2211.598	0.9356	1.0675
136.3776	250.5938	0.9855	2	61080	2085	2115	0.9341	1.0691
134.4388	329.375	1.0056	2	65130	2170	2155	0.9348	1.0683
383.2365	12.28523	1.0269	2	68374	2437	2373.18	0.9378	1.0651
347.3755	13.74612	1.0538	2	65979	2556	2425.564	0.9384	1.0643
363.6603	14.4438	0.9151	2	67445	2200	2405	0.9382	1.0646
365.0572	13.75137	0.9017	2	67750	2230	2475	0.939	1.0637
198.2576		0.9371	2	57165	1785	1904.76	0.9307	1.0728
158.5		0.974	2	57926	1925	1976.446	0.9319	1.0715
175.6326		0.9549	2	57170	1725	1805	0.9289	1.0749
157.2185		0.9987	2	54615	1810	1810	0.929	1.0747
37.16069	67.97391	1.0667	2	70634	2901	2719.614	0.9418	1.0607
39.11938	69.77741	1.0584	2	71079	2954	2790.974	0.9425	1.0599
36.61753	74.23454	1.144	1	73490	2840	2480	0.9391	1.0636
37.55783	76.26218	1.0428	2	75520	2935	2815	0.9428	1.0596
164.8217		1.0554	2	50401	1464	1387.188	0.9191	1.0858
145.8646		1.0768	2	46827	1448	1344.695	0.9178	1.0872
134.048	6519	1.1043	2	44615	1350	1225	0.914	1.0915
119.7058	919.7143	1.071	2	49635	1400	1305	0.9166	1.0885
247.9708	653.3077	1.0547	2	66121	2355	2232.762	0.9359	1.0671
217.853		0.9898	2	69415	2571	2597.476	0.9405	1.0621
197.9		1.0041	2	71195	2315	2305	0.9369	1.0661
213.0116		0.9718	2	70310	2260	2325	0.9371	1.0658
48.25726	40.79032	0.9421	2	132559	4156	4411.475	0.9541	1.0474
42.29084	36.26642	1.0445	2	134121	4520	4327.608	0.9537	1.0479
46.2222	33.81384	1.0782	2	137110	4380	4065	0.9522	1.0494
43.59165	32.42044	1.0311	2	139775	4520	4385	0.954	1.0476
55.46743	329.88	1.111	2	53027	2639	2375.332	0.9378	1.065
57.48252	316.1538	1.0134	2	57722	2675	2639.728	0.9409	1.0616
57.84125	302.2093	1.1994	1	62155	2475	2065	0.9334	1.0699
56.05404	205.3125	0.9743	2	63990	2670	2740	0.942	1.0605
67.43976		0.9936	2	99556	3109	3128.904	0.9457	1.0565
74.29193		0.97	2	95992	2999	3091.91	0.9453	1.0568
59.957	12549	1.0313	2	94515	2910	2820	0.9428	1.0596

For peer review only

54.5889	1542	1.063	2	91705	3000	2825	0.9429	1.0595
46.89739	29.21652	0.9884	2	70983	2491	2520.29	0.9396	1.0631
47.52798	29.81428	0.971	2	70740	2378	2449.024	0.9387	1.064
52.93922	27.91465	0.9998	2	68980	2320	2320	0.9371	1.0658
52.91008	28.28751	1.0135	2	69580	2295	2265	0.9363	1.0667
6977.5		1.0174	2	68071	2065	2029.585	0.9328	1.0705
6480		1.1408	1	68473	2173	1904.724	0.9307	1.0728
8146.667		1.0557	2	70215	2035	1930	0.9311	1.0724
		1.0903	2	68990	2160	1980	0.932	1.0714
591.6774		1.1539	1	57077	2437	2112.01	0.9341	1.0691
353.52		1.1125	2	56004	2407	2163.613	0.9349	1.0682
162.9995		1.1525	1	54465	2345	2035	0.9329	1.0704
125.3649	7557	1.0952	2	55810	2400	2195	0.9353	1.0678
46.0029	37.79105	1.0344	2	76369	2347	2268.897	0.9364	1.0666
41.52583	31.22605	1.0315	2	75510	2432	2357.822	0.9376	1.0653
41.57452	26.53308	0.9482	2	79450	2180	2300	0.9368	1.0661
45.92846	26.25632	0.9941	2	77575	2470	2485	0.9391	1.0636
547.353		1.0522	2	82604	3049	2897.82	0.9436	1.0587
491.5991		1.0611	2	81834	3094	2915.729	0.9437	1.0586
321.5429		1.112	2	82480	2990	2690	0.9415	1.061
99.17247		1.1322	1	78065	3075	2715	0.9417	1.0607
83.73766	155.4699	0.9316	2	56533	1555	1669.173	0.9261	1.078
71.63651	163.0785	1.016	2	57412	1684	1657.51	0.9258	1.0783
70.58005	184.5685	1.0036	2	63735	1600	1595	0.9244	1.0798
62.22194	352.6115	1.0447	2	62000	1665	1590	0.9243	1.0799
46.04168	471.125	1.0474	2	79129	2716	2593.148	0.9404	1.0622
44.90316	357.6563	1.0626	2	79659	2780	2616.252	0.9407	1.0619
44.18216	952	1.0479	2	82820	2680	2560	0.94	1.0626
42.66601		1.069	2	90650	2780	2600	0.9405	1.0621
49.09211	53.85069	1.0154	2	68114	2723	2681.784	0.9414	1.0611
51.1879	56.22449	1.0263	2	66784	2871	2797.561	0.9426	1.0598
48.4034	53.25408	0.9994	2	71445	2675	2675	0.9413	1.0612
46.31691	53.5601	1.0304	2	73325	2790	2705	0.9417	1.0608
946.4885	96.07904	0.7554	3	109071	2273	3009.068	0.9446	1.0576
860.2878	104.3373	0.7413	3	108220	2233	3012.465	0.9446	1.0576
1118.506	93.52647	0.7069	3	108285	2090	2955	0.9441	1.0582
1570.976	79.03068	0.7024	3	110080	2205	3135	0.9457	1.0564
45.52335	238.6984	0.954	2	76236	3204	3358.5	0.9475	1.0545
46.43745	575.55	0.9653	2	76228	3175	3289.137	0.947	1.0551
47.2273		0.9693	2	79290	2925	3020	0.9447	1.0575
43.41187		1.0166	2	80330	3225	3170	0.946	1.0561

od_ll	od_ul	nurse_adul	nurse_chilc	nurse_com	nurse_disal	nurse_men	healthvisit	nurse_train
0.8942	1.1183	932.4735	75.83111	188.0797	1	0	0	3.666667
0.8913	1.1219	944.3829	78.435	201.1061	1	0	0	2
0.8877	1.1266	961.2322	81.72056	209.6478	1.25	0	0	0.5
0.886	1.1287	1021.677	83.92001	200.0278	2.833333	0	0	0
0.8938	1.1188	4143.338	619.8026	341.2685	20.98667	12.29333	154.2582	13.63333
0.8905	1.123	4202.046	629.2242	451.4888	20.52	13.27666	146.7735	15.41667
0.8887	1.1252	4396.167	688.5308	566.9121	23.83056	15.42555	171.1857	16.25
0.8897	1.124	1649.176	145.5822	499.8895	19.32667	24.60666	30.28223	0
0.8881	1.126	1708.03	139.0739	499.2981	22.22334	24.59416	29.36834	0.75
0.884	1.1312	429.5805	24.62333	96.55447	12.73333	112.3461	28.76457	0
0.8816	1.1342	438.8506	27.68667	85.39228	14.73333	115.0672	30.53836	0
0.8776	1.1394	410.5658	30.30611	87.94506	13.30222	120.3044	26.86635	0
0.8771	1.1401	403.8178	32.39667	98.00707	11.29222	118.775	23.49884	0.416667
0.897	1.1148	3431.573	368.293	113.0697	3.833333	0	9.3626	35.91333
0.8944	1.1181	3668.558	391.7219	42.92243	1	0	3.816667	41.08333
0.8911	1.1223	3775.643	398.3775	40.40014	1	0	4.2	35.58333
0.8891	1.1248	3883.037	380.2681	41.76471	1	0	3.333333	27.06667
0.8956	1.1166	1991.669	85.7115	292.7107	2.833333	0.333333	126.3462	7.055554
0.8932	1.1196	1990.997	77.89763	265.7312	4.166667	0	93.59532	4.75
0.8898	1.1238	1974.926	77.58269	248.8729	5.391114	0	37.45596	6
0.8881	1.1261	2136.352	81.74735	160.3366	1.356668	0	5.773333	2.916667
0.8897	1.1239	890.2658	42.49035	0	0	0	0	0
0.8878	1.1264	886.3051	42.2308	0	0	0	0	0
0.885	1.1299	864.4276	45.18627	91.78166	0	0	0	0
0.8839	1.1313	917.0704	44.65743	93.20367	0	0	0	0
0.8844	1.1308	344.9744	6.03272	0.90667	0	5.995549	0	0
0.8823	1.1334	321.3402	5.635561	1.00667	0	4.019997	0	0
0.8787	1.1381	328.2142	6.503335	1.440003	0	4.294719	0	0
0.885087	1.129962	339.6904	7.213939	2.215761	0	6.343332	0	0
0.8866	1.1279	446.3426	17.85888	0.25	0	0	0	0
0.885	1.1299	444.5055	17.96222	0	0	0	0	0
0.8816	1.1344	467.1368	21.10755	0	0	0	0	0
0.881	1.135	522.7692	20.94488	0	0	0	0	0
0.8917	1.1214	1392.379	689.0553	20.21259	1.3	8.007733	0	0
0.8894	1.1244	1414.557	706.7736	21.93858	1.4	9.636198	0	0
0.8858	1.1289	1430.195	720.2882	21.95687	1.333333	11.34607	0	0
0.8873	1.127	1488.884	749.6544	21.15342	1.4	10.16681	0	0
0.8936	1.119	807.5548	39.74015	186.6664	1.46667	5.583334	51.78833	3.247777
0.89	1.1235	784.1836	45.62144	181.9368	1.46667	9.39993	42.21555	0.958333
0.8867	1.1278	759.1271	61.64485	180.3309	1.46667	10.19167	37.04667	0
0.8847	1.1304	773.2507	107.8992	173.7233	1.51667	0	26.78333	0
0.8926	1.1203	1072.12	153.0661	72.99334	0	0	0	0.75
0.8907	1.1227	1058.155	152.9163	75.69443	0	0	0	1.553333
0.8875	1.1267	1059.065	153.9884	71.08444	0	0	0	0.43
0.8854	1.1295	1073.398	150.3061	65.58556	0	0	0	0.75
0.8931	1.1197	871.0342	64.62598	1.166667	0	0	0	0
0.8912	1.1221	871.4481	66.5341	0.914447	0	0	0	0
0.8877	1.1265	885.331	62.2461	0.914447	0	0	0	0
0.8904	1.1231	1137.082	66.79851	1	0.044444	0	0	0

For peer review only

0.8957	1.1165	2151.279	149.97	6.05	1	0	0	22.925
0.8934	1.1194	2148.051	145.5433	9.019723	1	0	0	25
0.8901	1.1234	2114.332	146.2445	18.91145	1	0	0	20
0.8883	1.1258	2193.783	148.0377	17.4477	1	8.983337	0	14.75
0.8899	1.1237	764.7282	110.9055	0	0.4	0	0	0
0.8881	1.126	796.5917	105.6812	18.91761	0.4	0	0	14.33333
0.885	1.1299	787.608	100.8678	19.81761	0.266667	0	0	11.08333
0.8831	1.1324	807.5683	94.5967	19.98983	0	0	0	8.666667
0.8862	1.1284	633.3256	63.23221	0	0	0	0	0
0.8852	1.1297	634.1189	63.74555	0	0	0	0	0
0.8811	1.135	665.7098	67.88666	0	0	0.083333	0	0.416667
0.8802	1.1361	688.2246	71.57889	0	0	1.416667	0	0.25
0.8897	1.124	604.3442	85.88109	4.95667	0	0	0	4.333334
0.888	1.1261	635.0224	91.23303	5.953336	0	0	0	4.416667
0.8848	1.1302	690.3579	87.28359	6.215556	0	0	0	5.75
0.8836	1.1318	771.5969	89.48537	1.92667	0	0	0	2.666667
0.8923	1.1208	855.8978	77.828	0	0	0	1	0
0.8905	1.1229	837.7005	78.40379	1.406667	0	0	1	0
0.8872	1.1272	849.7393	79.23319	2.454777	0	0	0.8	0
0.892637	1.12035	965.6409	83.32649	5.802823	0	0	1.090909	0
0.8859	1.1288	509.0468	41.88127	0	0.4	0	0	0
0.8833	1.1321	509.2713	38.9012	0	0.6	0	0	0
0.8791	1.1376	511.3606	38.8253	0	0.65	0	0	0
0.879	1.1377	529.5251	38.2068	1.37222	0.8	0	0	0
0.8906	1.1228	593.3972	67.49415	237.2323	0	4.90333	60.65955	7.916667
0.8881	1.126	599.4334	75.56342	240.3317	0	7.143333	55.12639	1.778888
0.884	1.1312	610.4744	76.38509	241.7111	0	6.711114	53.64665	1.036954
0.8829	1.1326	594.9573	84.64896	248.0253	0	7.463331	47.75444	2.056089
0.894	1.1185	1243.727	96.72438	5.300007	0	0	0	0
0.8916	1.1216	1197.903	109.9077	3.443336	0	0	0	0
0.8875	1.1268	1191.036	117.6999	0	0	0	0	0
0.8856	1.1292	1201.773	121.5899	0	0	0	0	0
0.8938	1.1189	1111.031	91.10308	0	0	0	0	0
0.8916	1.1216	1169.127	90.97529	0	0	0	0	0
0.8881	1.126	1215.814	98.75364	0	0	0	0	0
0.8865	1.1281	1270.029	100.7999	8.15	0	0	0	0
0.8904	1.1231	692.2691	60.41388	80.61196	0	0	0	0
0.8884	1.1257	675.4976	64.89831	155.2552	0	0	0	0
0.8844	1.1307	645.8008	64.78165	168.446	0	0	0	0
0.8837	1.1316	687.4832	62.28331	176.6411	0	0	0	0
0.8862	1.1284	517.5748	44.90324	270.1842	0	0.9	0	0
0.8842	1.131	453.5316	44.88032	158.6995	0	0	0	0
0.8808	1.1354	429.2428	47.6812	152.0787	0	0	0	0
0.8786	1.1382	445.3337	48.209	160.6599	0	0	0	0
0.8873	1.127	623.5169	52.52622	0	0	0	0	0
0.8857	1.129	620.283	55.79511	0	0	0	0	0
0.8819	1.1339	629.4073	50.03022	0	0	0	0	0
0.881033	1.1348	635.3941	48.6086	0	0	0	0	0
0.8913	1.122	813.2935	168.7883	0	0	0	0	4.75
0.8895	1.1243	841.2559	177.8853	0	0	0	0	6.833334

For peer review only

0.8858	1.1289	880.7965	180.0742	0	0	0	0	7
0.8879	1.1262	1004.967	184.3675	0	0	0	0	4.833334
0.8959	1.1162	1429.217	85.01666	224.6841	0	0	0	0
0.8933	1.1195	1433.93	88.00221	214.5344	0.1	0	0	0
0.8899	1.1238	1397.072	84.89333	153.4824	0.6	0	0	0
0.8884	1.1256	1408.298	86.76444	145.3189	0.816667	0	0	0
0.8856	1.1291	510.0408	32.60161	246.5277	0	0	262.7362	5.958334
0.8836	1.1317	509.5757	32.6382	258.4661	0	0	270.1708	6.875
0.88	1.1364	527.6488	30.29973	329.8589	0.476667	0	369.8703	12.75
0.879	1.1376	529.3516	31.55377	364.4937	2.10667	0	378.2928	10.04167
0.8868	1.1276	461.2608	42.35166	61.22777	0	0.8	0	0
0.8843	1.1309	478.7017	44.71666	54.76999	0	0.8	0	0
0.8815	1.1344	471.155	45.35666	61.365	0	0.8	0	0
0.8806	1.1356	450.703	48.16444	81.09796	0	1.433333	0	0
0.8916	1.1216	655.3884	65.16668	0	0	3.416667	0	3.25
0.8897	1.124	641.8792	64.84	0	0	2.816663	0	3.416667
0.8859	1.1288	629.3221	65.81778	0	0	3.766663	0	2
0.8841	1.1311	677.8806	65.09389	0	0	3.316666	0	0.583333
0.8929	1.1199	981.6195	79.42887	0	0	0	0	0
0.8905	1.1229	964.4091	84.1411	0	0	0	0	0
0.887	1.1273	947.7358	79.39166	0	0	0	0	0
0.8855	1.1293	990.4032	80.03056	0	0	0	0	0
0.8927	1.1202	796.6229	57.56	0	0	0	0	0
0.8899	1.1237	786.6083	59.02	0	0	0	0	0
0.8863	1.1282	748.269	65.50611	0.5	0	0	0	0
0.8849	1.1301	776.4399	67.95778	1	0	0	0	0
0.8882	1.1259	610.5399	88.15833	0	0	0	0	0
0.8872	1.1271	636.7124	82.41	0	0	0	0	0
0.8835	1.1319	661.7094	83.91387	0	0	0	0	0
0.8807	1.1354	666.944	85.02264	0	0	0	0	0
0.8932	1.1195	1012.691	91.01663	0	0	0	0	0
0.891	1.1223	1022.564	94.55718	0	0	0	0	0
0.8877	1.1265	1032.273	98.73107	0	0	0	0	0
0.89365	1.118963	1106.15	95.8424	0	0	0	0	0
0.8934	1.1194	862.4828	97.78243	1.966667	0	0	0	0
0.8943	1.1182	856.7575	102.8125	3.435555	0	0	0	0
0.891	1.1224	1622.504	133.7295	141.0722	0	0	0	0
0.8893	1.1245	2014.437	152.422	188.5114	0	0	0	0
0.8967	1.1152	1828.192	135.5909	8.415001	0	0	0.8	0.243333
0.8941	1.1185	1871.832	141.0819	32.21667	0	0	0.8	1.166667
0.8907	1.1227	1915.51	140.5197	28.52334	0	0	0.833333	0.666667
0.8889	1.125	2078.584	146.819	24.50767	0	0	1	1
0.8935	1.1192	1083.768	0.833333	10.86	0	0	0	0
0.8911	1.1223	1079.53	3.75	9.658893	0	0	0	0
0.8878	1.1264	1075.72	1.2	9.143336	0	0	0	0
0.8869	1.1275	1102.234	1.083333	9.47667	0	0	0	0
0.8857	1.1291	403.6502	46.25464	622.9471	26.11667	27.0961	170.0906	1.866889
0.8827	1.1328	388.6399	39.53132	536.0984	20.98889	28.34166	162.5489	2.219866
0.887329	1.127071	385.7677	42.36299	507.4027	21.00075	24.51407	70.33926	0.776117
0.8944	1.1181	1007.468	105.2935	9.085534	2.706667	0.3	3.668889	0

For peer review only

0.8921	1.1209	995.7218	102.3829	5.980556	2.706664	0.475555	3	0
0.8887	1.1253	1017.17	100.4395	5.985556	4.293332	0.676665	2.333333	0
0.8871	1.1273	1071.545	102.1862	3.51	5.058333	0.85333	3	0
0.8929	1.12	1082.589	0	0	0	0	0	0
0.8911	1.1223	1119.299	0	0	0	0	0	0
0.8878	1.1263	1156.404	0	0	0	0	0	0
0.8892	1.1245	2139.738	2.5	17.74667	0	0	0	0
0.8957	1.1164	1489.785	141.1291	1	0	0	0	20.83333
0.8933	1.1195	1474.212	136.7794	1	0.416667	0.833333	0	22
0.8898	1.1238	1481.259	137.693	1	1.166667	2.333333	0	24.25
0.8879	1.1263	1485.129	146.4651	0.75	2.25	3.209997	0	17.25
0.8902	1.1233	648.2442	72.09444	0	0	0.516667	0	0
0.8881	1.126	660.9995	74.74999	0	0	0.783333	0	0
0.8855	1.1294	696.6334	78.69278	0	0	1.216667	0	0
0.8837	1.1316	706.1877	76.04778	0	0.25	1	0	0
0.8895	1.1242	677.6792	67.67038	230.0498	0.583333	0	54.64143	0.75
0.8871	1.1273	665.3066	69.22242	240.5007	1	0	51.31857	1.333333
0.8839	1.1313	653.7396	72.58953	245.5061	1	0	47.03112	1.333333
0.8814	1.1345	665.2485	85.11832	230.9401	1	0	43.00945	1.083333
0.8925	1.1204	703.8709	68.18	12.42083	0	1	0	0.416667
0.8903	1.1233	685.1783	70.80666	24.365	0	0.97	0	0
0.8865	1.1281	719.91	73.00584	37.2925	0	0.91	0	0
0.885	1.1299	740.265	73.85333	27.08417	0	0.91	0	0
0.8947	1.1177	1017.46	64.98109	0	0.083333	0	0	0
0.8923	1.1206	1395.08	65.05543	0	1	0	0	0
0.889	1.1249	1407.235	67.20664	0	0.166667	0	0	0
0.8873	1.127	1495.809	131.4829	3.8	0	0	0	0
0.8896	1.124	651.6268	55.11328	0	0	0	0	0
0.8877	1.1265	664.6072	55.19502	0	0	0	0	0
0.8852	1.1297	660.2942	54.69335	0	0	0	0	0
0.8833	1.1321	664.1058	55.90177	0	0	0	0	0
0.8928	1.12	864.6028	38.63564	76.0911	0	0	0	0
0.894467	1.117883	896.7827	38.55109	133.2041	0	0	0	0
0.894467	1.117883	911.5754	36.00887	191.3931	0	0	0	0
0.891	1.1223	693.8419	42.42666	19.01779	0.583333	0	0	0
0.8892	1.1246	665.8073	46.58834	70.09524	1	0	1.633333	0
0.8854	1.1295	633.7678	45.18388	124.0483	1	0	2.8	0
0.8842	1.1309	694.2846	50.03332	132.4695	1	0	3.168889	0
0.8949	1.1175	2183.178	321.7554	0	0	0	0	8.683333
0.8924	1.1206	2167.898	331.2856	0.2	0	0	0	2.433333
0.8888	1.1251	2346.611	338.114	0.8	0	0	0	0
0.8872	1.1271	2574.149	355.6903	0.8	0	0	0	0
0.8928	1.1201	1414.677	8.085	123.1561	0	0.9	0	0
0.8908	1.1226	1399.061	9.102222	243.7633	0	1.8	0	0
0.8877	1.1265	1408.278	14.51832	252.8989	0	1.8	0	0
0.8861	1.1285	1502.834	50.73051	255.7341	0	1.666667	0	0
0.8956	1.1165	2343.556	417.2282	0	0	0	0	0
0.8933	1.1194	2341.257	429.7074	0	0	0	0	0
0.8898	1.1238	2326.906	439.6442	0	0	0	0	0
0.8872	1.1272	2435.625	447.5938	0	0	0	0	0

For peer review only

0.8965	1.1154	2954.054	115.2689	384.0978	0	0	0	0.5
0.894	1.1186	2980.61	117.8329	392.6725	0	0	0	0.083333
0.8906	1.1228	2940.78	113.815	397.1276	0	0	0	0
0.8888	1.1251	2958.596	104.4233	411.8233	1	0	0	0
0.8953	1.117	1763.843	136.874	0	0	0	0	0
0.8929	1.1199	1709.408	136.5305	0	0	0	0	0
0.8896	1.1241	1671.45	134.4518	0	0	1.583333	0	0
0.8881	1.126	1743.999	166.3559	0	0	0.25	0	0
0.8932	1.1195	1221.729	104.3029	9.741667	0	0	0	4.916667
0.8904	1.1231	1182.532	102.7368	9.241667	0	0	0	3.833333
0.8868	1.1276	1195.775	103.2257	9.325	0	0	0	5.666667
0.8855	1.1293	1253.743	102.2263	7.125	0	0	0	2
0.8927	1.1202	2860.24	561.2591	375.8888	7.3	0	149.6942	3.833333
0.8906	1.1228	2943.402	588.0947	369.2369	9.033334	0	132.4942	1.5
0.8874	1.127	3128.288	653.048	379.2003	9.45	0	123.1486	3
0.8856	1.1292	3214.346	686.9036	395.6739	8.366667	0	114.8605	4.083334
0.894	1.1185	1329.278	173.9125	162.4227	0	0	68.30031	4.666667
0.8922	1.1209	1283.234	171.4398	119.6061	0	0	64.23372	1.833333
0.8886	1.1254	1310.932	170.794	117.6561	0	0.416667	58.37645	1.5
0.8871	1.1273	1435.551	177.2726	114.4366	0	1.416667	52.43145	0.75
0.89	1.1236	650.1386	79.0311	134.9455	6.616667	0	42.87667	5.083334
0.8876	1.1267	636.1207	83.55888	135.3457	6.6	0	47.50222	9.333333
0.8839	1.1313	617.7535	84.68416	141.7124	5.983333	0	50.85667	10.5
0.8821	1.1337	631.2926	89.20146	149.9111	6.15	1.516667	55.08111	9.75
0.8941	1.1184	2092.353	182.1847	195.0748	4.583334	0	55.74729	0
0.8919	1.1212	2039.46	189.4915	148.6676	4.833334	0	42.83158	0
0.8884	1.1256	2028.583	191.9389	69.90372	4.5	0	2.25	0
0.8863	1.1283	2010.969	208.5983	32.13445	1.5	0	1	0
0.8875	1.1268	613.4317	35.49112	343.7901	0.730043	0	112.4495	1.333333
0.8864	1.1281	598.4916	35.01889	341.9697	2.12013	0	123.2439	0.592133
0.8826	1.133	582.9486	40.80055	372.2428	1.815909	0.166667	168.1067	0.166667
0.8815	1.1344	580.256	39.24833	384.0548	0.886667	1	185.6946	1
0.897	1.1148	2437.319	169.5113	1.706667	0	0.5	0	3.083333
0.8944	1.1181	2438.483	175.2088	0.266667	0	1.166667	0	3.75
0.8913	1.122	2423.786	181.6485	0	0	2.916667	0	0.666667
0.8896	1.1241	2423.294	186.7937	0	0.066667	5.13	0	0
0.889	1.1249	688.6455	47.76889	0.383333	0	0	0	0
0.891567	1.121767	656.7566	49.34667	0	0	0	0	0
0.891567	1.121767	657.6688	50.01333	0	0	0	0	0
0.8936	1.1191	1009.15	116.8842	135.8993	0.166667	0	34.45223	5
0.8911	1.1222	1013.551	113.8568	132.1672	1	0	11.94111	1.083333
0.8878	1.1264	1009.467	116.7366	133.3483	1	0.083333	0.464447	2.166667
0.8863	1.1283	1024.92	123.5659	134.405	1	0.833333	0.54667	2.416667
0.8839	1.1314	421.6009	25.74666	214.92	0.4	1	22.00167	0
0.8814	1.1345	421.4428	25.48834	134.7213	0	1	10.205	0
0.8771	1.1401	425.7275	27.37555	131.3829	0	0.583333	0	0
0.8759	1.1417	412.5057	27.97276	113.6915	0	0	0	0
0.8897	1.1239	776.8864	65.75332	1	0	0	0	0
0.8874	1.1269	743.0048	61.30778	0.88	0	0	0	0
0.8843	1.1308	740.1496	62.46668	0.57	0	0	0	0

For peer review only

0.8829	1.1327	757.8053	64.49857	0	0	0	0	0
0.8961	1.1159	2955.457	322.5715	3.866667	0	0.886665	0	8.75
0.8935	1.1191	3135.21	327.9695	4.75	0	1	0	8.25
0.8902	1.1233	3412.541	381.0605	4.583334	0	0.7	0	9.583333
0.8883	1.1257	3422.06	365.8611	3.833333	0.75	0.666667	0	4.666667
0.8932	1.1196	893.7457	59.48054	20.87889	0	0	0	1.333333
0.8905	1.123	923.543	69.52354	2.99333	0	0	0	0.083333
0.8873	1.127	925.2714	70.18632	2.49333	0.45	0	0	0
0.8862	1.1284	961.5796	71.83152	2.49333	0.6	0	0	0
0.8942	1.1183	1320.541	132.7235	4.233333	0	0	0	0
0.8918	1.1213	1370	137.5256	5.45	1.4	0	0	0
0.8881	1.126	1411.412	139.0835	5.716667	2.4	0	0	0
0.8868	1.1277	1499.594	146.9656	6.066667	3.35	0.453335	0	0
0.8947	1.1177	1712.27	171.6185	6.280004	0	0.31111	0	1.816667
0.8924	1.1206	1767.11	174.2457	7.58333	0	0	0	1.5
0.889	1.1248	1859.428	183.3054	7.96111	0	0	0	1.166667
0.8873	1.127	1952.296	185.5717	10.66667	0	0.533333	0	0.25
0.8816	1.1343	518.3049	105.7076	247.785	11.43056	11.16667	114.8489	8.583333
0.8801	1.1363	488.3384	101.5545	218.7212	8.356667	9.416667	112.6634	9.333333
0.8748	1.1432	494.2938	93.17681	211.8238	5.815	8.208333	109.305	7.166667
0.8724	1.1462	511.0234	95.89186	209.8502	7.39	8.791667	102.4145	5.666667
0.8937	1.119	1436.991	117.5066	256.3607	0	0	60.21	5.166667
0.891	1.1223	1465.272	119.5278	260.9845	0	0	58.88222	0.75
0.8882	1.1258	1486.883	133.5013	249.834	0	0	59.42722	0.083333
0.8871	1.1273	1557.492	143.5095	252.7569	3	1.916667	60.79222	2.083333
0.8933	1.1195	1231.063	96.59308	10.73889	0	0	0	0
0.8906	1.1229	1228.143	97.03388	10.81667	0	0	0	0
0.894757	1.117757	1235.75	100.6126	8.911118	0	0	0	0
0.8864	1.1282	512.3878	43.66211	98.59136	2.855556	0	33.81332	0.583333
0.8848	1.1302	514.3804	44.61266	90.33167	0.58	0	31.36443	1
0.8823	1.1334	507.8645	45.63543	93.17722	0.04	0	24.99333	0.416667
0.8811	1.135	544.8638	44.85931	100.5278	0	0	26.64333	0
0.8843	1.1309	450.5826	31.28157	9.311547	0	0	0	0
0.8821	1.1336	458.2209	33.25224	7.266664	0	0	0	0
0.8801	1.1362	475.2287	34.83112	3.516665	0	0	0	0
0.8789	1.1378	498.3619	35.03	1	0	0	0	0
0.8959	1.1162	1526.421	131.3622	0	0	0	0	11.08333
0.8935	1.1192	1600.55	145.5386	0	0	0	0	11.41667
0.89	1.1236	1678.088	145.8872	0	0	0	0	6.833334
0.8883	1.1258	1774.747	156.095	0	0	0	0	2.083333
0.8931	1.1197	1461.351	98.54997	130.6184	0	0	0	0
0.894733	1.117583	1469.174	103.7444	128.8445	0	0	0	0
0.8931	1.1197	1421.578	24.44556	246.9392	7.316667	1	63.44778	1.333333
0.8907	1.1227	1422.911	28.36222	258.0294	9.596666	1.75	59.14111	1.083333
0.8867	1.1278	1467.88	25.84889	276.5801	9.533334	2	54.87723	0.833333
0.8849	1.13	1561.289	33.26944	574.1403	10.65	1.901111	108.9174	2.333333
0.8913	1.1219	892.6392	127.4301	295.5939	15.66667	3.883333	71.16444	8.094445
0.8888	1.1251	924.7579	130.3874	290.6352	19.63333	5	70.24778	5.5
0.885	1.13	972.1911	139.5204	275.8503	21.6	0	67.24445	4.166667
0.8831	1.1323	1000.569	165.5945	291.777	20.15	0	65.47334	8.416667

For peer review only

0.8888	1.1251	593.8639	41.10223	162.6577	9.78667	0.813514	56.14887	1.416667
0.887	1.1274	607.7961	40.29001	166.0469	10.58667	0	57.18665	1.5
0.8822	1.1335	610.2389	43.02	165.2152	10.62	0	57.18554	1.416667
0.8823	1.1335	592.2289	45.70445	161.9699	10.70889	0	60.07998	1.833333
0.8922	1.1208	849.585	44.07093	255.3348	13.36	0	0	0
0.8906	1.1228	877.3741	41.97609	343.2059	14.87666	0	0	0
0.887	1.1274	873.759	43.5348	128.5008	0.083333	0	0	0
0.8852	1.1297	946.0252	52.78702	142.2697	0	0	0	0
0.8925	1.1205	1131.449	113.1868	7.323843	0	0	0	0
0.8908	1.1226	1163.328	113.6123	6.012337	0	0	0	0
0.8874	1.1269	1163.312	108.9001	5.349939	0	0.25	0	0
0.8862	1.1284	1240.709	106.1997	6.203566	0	0.826667	0	0
0.8896	1.1241	728.7282	80.06832	0.583333	0	0	0	0
0.888	1.1261	732.8505	80.90394	0	0	0	0	0
0.8845	1.1305	746.2473	80.75495	0	0	0	0	0
0.8829	1.1327	741.1624	80.83046	0	0	0	0	0
0.8925	1.1205	932.3214	57.71112	0	0	0	0	0
0.8903	1.1232	917.8276	61.48779	0	0	0	0	0
0.8868	1.1276	899.7362	60.31834	0	0	0	0	0
0.892663	1.120175	879.6503	61.40934	0	0	0	0	0
0.8852	1.1297	167.7567	15.65756	491.7146	39.79445	248.315	90.7305	0
0.8852	1.1297	156.3467	50.48033	448.5836	45.17223	262.8245	78.25467	0
0.8852	1.1297	117.7269	57.60254	282.1128	42.62	275.6717	74.21556	0
0.8852	1.1297	607.4229	94.44069	285.5564	22.42333	131.5244	67.85778	0
0.891	1.1223	824.6348	50.97277	0	0	0	0	0
0.8886	1.1254	846.4594	48.17389	0	0	0	0	0
0.8857	1.129	825.6768	53.62333	0	0	0	0	0
0.8842	1.131	849.5391	62.34554	0	0	0	0	0
0.8927	1.1203	964.0291	99.75534	9.96	1	0	0	0
0.8905	1.123	979.4166	105.2193	8.74667	1	0	0	0
0.8871	1.1273	1009.439	105.2554	7.568889	1	0	0	0
0.886	1.1286	1035.292	110.2926	7.9	1	0	0	0
0.8862	1.1284	643.3936	24.1422	13.40057	0	0	0	0
0.8838	1.1314	609.0319	25.67221	13.64783	0	0	0	0
0.8807	1.1355	616.1137	28.20443	13.62111	0	0	0	0
0.8791	1.1375	696.9608	35.05666	13.78111	0	0	0	0
0.895	1.1173	1289.359	108.5357	0	0	1	0	0
0.8924	1.1205	1266.688	111.7104	0	0	1	0	0
0.8888	1.1251	1255.033	108.6671	0	0	1	0	0
0.8872	1.1271	1271.186	102.1014	0	0	1	0	0
0.8919	1.1212	767.916	77.258	29.51136	5.579134	0	0	1.583333
0.89	1.1236	792.5388	79.28059	24.15628	4.75	0.75	0	0.916667
0.8863	1.1283	830.232	69.51041	6.180277	2.766667	0.916667	0	0
0.8848	1.1302	888.1129	125.6404	0.897443	3.093334	0	0	0
0.8927	1.1203	1742.074	0.333333	1.166667	0	0	0	0
0.8905	1.123	1646.155	0.666667	22.76667	0	0	0	0
0.8866	1.1279	1601.998	2.5	27.25444	0	0	0	0
0.89325	1.119613	1597.999	4	29.1	0	0	0	0
0.892	1.121	1010.965	68.44713	0	0	0	0	0
0.8901	1.1235	997.9113	68.35053	0	0	0	0	0

For peer review only

0.8866	1.1279	966.573	72.97564	0	0	0	0	0
0.892525	1.120475	1012.911	72.28302	0	0	0	0	0
0.8926	1.1203	1230.6	267.2507	12.34778	1.866667	1	1	4.166667
0.8905	1.123	1250.884	260.2879	13.49222	1.3	1	0.8	5.416667
0.8875	1.1267	1384.63	268.5284	15.52667	0.8	1	0.825557	7.043334
0.8861	1.1285	1517.622	282.9721	4.571109	0.8	1	1.13667	3.223333
0.8834	1.13206	396.3409	0	0	1.090909	0	0	0
0.8899	1.1237	562.6371	64.95	0.75	0	0	0	0
0.8875	1.1267	572.5908	66.90859	1.178333	0.45	0	0	0
0.8844	1.1307	579.5645	70.4944	1.966667	1	0	0	0
0.8834	1.132	664.9507	75.47956	2.033333	0.5	0	0	0
0.8798	1.1366	572.2294	114.3737	130.2895	0.416667	0	70.28445	14.33333
0.8779	1.1391	579.7982	114.7166	129.2261	0	0	71.91444	16.5
0.8744	1.1436	605.8823	121.1516	132.3284	0.25	0	73.73888	14.91833
0.8731	1.1453	647.3892	138.0541	129.8038	0.916667	0	71.58833	10.5
0.8974	1.1144	2178.899	198.4442	153.3098	2.766667	2.666667	41.86499	17
0.8998	1.111517	2149.013	193.4675	140.4783	3.785455	3.818182	21.08242	14.63636
0.8905	1.123	738.6011	33.79333	93.92922	0	51.12667	0	3.333333
0.888	1.1261	747.8984	36.66444	182.9615	0	50.42111	0	0.251126
0.8839	1.1314	749.2594	45.3989	184.864	0	52.87778	0	0.5
0.8826	1.133	755.8593	44.59001	179.7175	0	55.51444	0	0
0.897	1.1148	2777.694	563.3627	0.74667	0	0	0	0.333333
0.8942	1.1183	2831.115	561.9739	0.124445	0	0	0	0.416667
0.8908	1.1226	2844.283	579.4337	0	0	0.458333	0	0.083333
0.889	1.1248	2895.603	606.6335	0	0	0.5	0	1
0.8906	1.1229	965.5936	40.95333	46.85334	0	0	0	4.3
0.889	1.1248	935.5695	41.60111	45.44889	0	0	0	2.5
0.8853	1.1296	927.0773	42.16778	45.02889	0	0	0	2.5
0.8833	1.1321	937.9495	82.89722	242.3412	9.783334	0	65.19058	1.833333
0.8946	1.1178	2190.586	0	58.20187	0	0	0	0
0.896	1.1161	2404.021	15.82246	81.33336	0.35	0.333333	0.156111	1.333333
0.8927	1.1202	4383.703	182.9562	195.5612	4.516667	3.333333	0.25	8.916667
0.8909	1.1225	4408.709	187.9505	187.1434	5.516667	3.666667	0	6.25
0.8905	1.1229	2125.895	157.2745	0	0	1	0	6.416667
0.8885	1.1255	2098.578	148.0135	0	0	1	0	7.916667
0.885	1.1299	2185.762	158.9197	0	0	1	0	3.25
0.8854	1.1294	2310.329	166.8023	0	0	1.333333	0	3.833333
0.8951	1.1172	2579.123	669.9942	258.8777	0	3.966667	58.62388	6.416667
0.8925	1.1204	2696.033	702.4392	289.0109	0	3.1	63.71333	5
0.889	1.1248	2716.577	715.7698	279.0839	0	3.3	64.20555	4.583334
0.8875	1.1268	2763.9	726.9374	264.5944	1.366667	2.705554	60.48014	2.833333
0.8952	1.1171	1476.88	141.0635	2	2.45	0.666667	0	0
0.8927	1.1202	1523.606	149.7429	2	2.8	0	0	0
0.8894	1.1244	1546.771	147.674	2.4	2.8	0	0	0
0.8878	1.1264	1541.531	151.5109	2.8	2.466667	0	0	0
0.8955	1.1167	1249.046	61.72363	525.8792	19.25167	68.61166	115.0048	0
0.8932	1.1195	1260.567	64.13297	482.0697	23.08223	69.64668	72.34278	0
0.8898	1.1239	1261.037	61.9405	477.6875	23.21779	62.2589	70.26389	0
0.8883	1.1257	1284.333	64.21699	492.3493	24.71779	58.73779	69.99	0
0.8958	1.1163	1776.043	178.5991	9.669998	3.366667	0	0	1.25

For peer review only

0.8948	1.1175	1838.797	178.3191	0	3.717778	0	0	0
0.8914	1.1218	2311.823	205.9585	0.2	4.293336	0	0	0
0.89	1.1236	2618.936	216.3485	0.966667	2.756664	0	0	0
0.8961	1.1159	2803.265	322.6111	1.3	0	0	0	0
0.8936	1.1191	2670.202	324.986	3.6	0	0	0	0
0.8903	1.1232	2587.213	326.363	4.166667	3.733333	1	0	0
0.8887	1.1252	2648.878	334.1324	1.333333	7.366667	2.333333	0	0
0.8923	1.1207	740.9286	100.8982	0	0	0	0	0
0.8898	1.1239	710.7043	102.4373	10.15	0	0	0	0
0.8859	1.1288	650.854	103.4351	9.205552	0	0	0	0
0.8844	1.1307	679.8958	105.073	8.71666	0.55	0	0	0
0.893	1.1198	780.2678	78.40716	0	0	0	0	0
0.8907	1.1227	786.5336	76.90313	0	0	0	0	0
0.8872	1.1272	845.3033	82.78992	0	0	0	0	0
0.8856	1.1291	1047.349	87.79513	0	0	0.266667	0	0
0.8951	1.1172	1752.082	186.0492	262.1019	1	0	3.608889	6.524444
0.8922	1.1208	1801.636	194.0905	223.9605	0	0	0	5.62
0.8887	1.1253	1805.545	199.0978	211.4566	0	0	0	4.666667
0.8867	1.1277	1803.377	219.8283	207.3061	0.22	0	0	0.583333
0.8921	1.121	1138.825	101.3212	0	0	0	0	0
0.8897	1.124	1106.111	99.30907	0	1.373333	0	0	0
0.8866	1.128	1108.51	106.3852	259.3355	2.16	0.48	0	0
0.8851	1.1298	1197.648	109.4193	319.9392	2	0.64	0	0
0.894	1.1185	1638.403	73.3588	1.858333	0.286667	0.675	0	0
0.8919	1.1212	1639.048	69.68385	0.533333	0	0	0	0
0.8887	1.1253	1586.795	68.03442	0.75	0	0	0	0
0.8872	1.1272	1617.038	77.71941	1.653335	0	0	0	0
0.893	1.1198	1112.593	90.27065	10.84498	0	0	0	5
0.8907	1.1227	1082.231	90.83812	10.28494	0	0	0	8.166667
0.8872	1.1272	1091.666	89.63179	10.40941	0	0	0	8.75
0.8855	1.1293	1134.068	103.1721	72.43516	0.6	0	0	5.583334
0.8969	1.115	1732.71	73.26987	0.083333	0	0	0	0
0.8943	1.1182	1676.597	72.72431	0	0	0	0	0
0.8907	1.1227	1682.383	69.36575	0	0	0	0	0
0.889	1.1249	1730.819	96.80993	0	0	0	0	0
0.8925	1.1204	837.3277	94.50886	220.8084	0.666	0.733333	62.96778	3.583333
0.8905	1.123	892.6312	93.64056	212.3815	0	0.9	43.51444	4.75
0.8876	1.1266	906.2018	79.79056	196.8422	0	0.4	0.625	2.75
0.8859	1.1288	900.2044	75.89223	197.9306	0	0.4	0	0.333333
0.8877	1.1265	568.3622	66.82111	207.39	0	0	0.24	0
0.8853	1.1295	550.92	70.16667	39.48834	0	0	0.24	0
0.8837	1.1316	546.9495	71.42944	23.425	0	0	0.08	0
0.8826	1.1331	561.6867	73.83889	21.49001	0	0.333333	0	0
0.8908	1.1226	2498.143	518.0199	223.4365	27.41667	7.734446	157.5847	11.69111
0.89415	1.11845	2567.274	496.166	198.8931	22.36889	13.01555	153.4682	8.466666
0.8964	1.1156	1872.962	201.0867	230.6528	1	6	0	4.083334
0.8942	1.1184	1930.831	204.6867	257.7439	2.5	4.25	0	1.833333
0.8907	1.1227	1978.418	206.5956	169.2172	2.083333	1.416667	0	2.916667
0.8889	1.1249	2074.674	215.0222	150.9822	2	0.666667	0	3.416667
0.8957	1.1164	1758.222	136.9	2.583333	0	0.133333	0	0

0.8932	1.1196	1755.077	143.5734	1.666667	0	1.568889	0	0
0.89	1.1236	1782.158	140.64	2.816667	0	1.25333	0	0
0.8884	1.1257	1812.18	137.8084	4.685556	0	0	0	0
0.896	1.1161	1524.683	110.3157	24.13278	0.8	0	0	1.166667
0.8933	1.1194	1534.934	106.1568	21.94722	0.8	0	0	0
0.89	1.1236	1498.749	105.1906	25.50476	0.4	0	0	0
0.8885	1.1255	1475.584	108.7584	33.68188	0	0	0	0
0.8972	1.1146	2661.4	391.9324	44.94446	0	0	0	0.25
0.8947	1.1176	2621.555	403.7183	42.00114	0	0	0	0
0.8914	1.1219	2694.811	401.9143	40.94921	0	0	0	0
0.8897	1.124	2844.259	406.1089	43.9781	0	0	0	0
0.8937	1.1189	1148.264	85.75201	0.53778	0	0.066667	0	0
0.8916	1.1215	1068.637	93.6227	1.61334	0	0.8	0	0
0.8883	1.1258	1059.077	94.0149	1.61334	0	0.8	0	0
0.8867	1.1278	1138.213	100.0701	1.61334	0	0.599997	0	0
0.8936	1.119	972.4022	94.1759	0	0	0	0	7.833334
0.8911	1.1222	964.114	94.65011	0	0	0	0	8.333333
0.8877	1.1265	1034.079	98.24717	0	0	0	0	7.25
0.8861	1.1285	1123.638	96.73172	0	0	0	0	4.166667
0.8941	1.1185	1182.847	112.7374	1.8	0	0	1.3	6.166667
0.8917	1.1214	1239.428	111.9016	1.8	0	0	0.966667	7.75
0.8886	1.1254	1237.781	123.9484	1.768889	0	0	0.8	2.333333
0.887	1.1275	1280.891	122.7409	2.05333	0	0	0.8	5.5
0.8925	1.1205	897.755	89.00889	202.3796	0	0	66.04335	7.77333
0.8903	1.1232	901.3892	89.15222	204.4966	0	0	62.43834	3.583333
0.8866	1.1279	886.4808	83.91611	221.3888	0	0	64.6542	2.083333
0.8849	1.1301	853.3253	83.57778	234.3043	0	0	64.69087	1
0.8949	1.1175	1300.755	85.68909	23.71334	0	0	0	0
0.8925	1.1204	1309.912	84.38979	24.65278	0	0	0	0
0.8888	1.1252	1304.193	89.2096	26.61111	0	0	0	0
0.8877	1.1266	1329.467	86.40834	30.30223	0.945555	0	0	0
0.8896	1.1241	741.3198	71.40317	3.70667	0	0	0	0
0.8869	1.1275	750.4463	78.22595	0.617778	0	0	0	0
0.8829	1.1326	754.3488	77.48702	0	0	0	0	0
0.882	1.1338	760.3403	75.16036	0	0	0	0	0
0.8937	1.119	1151.926	98.19381	163.6231	0	0.083333	47.98999	0
0.8921	1.1209	1119.037	112.2333	158.4024	0	0.083333	10.24277	0
0.8883	1.1257	1091.317	110.1097	158.0956	0	0	0	0
0.8866	1.1279	1106.083	105.6062	153.586	0	0	0	0
0.8971	1.1147	2922.493	464.3519	24.25	0	0	0	0
0.8945	1.118	2981.982	464.1899	24.82668	0	0	0	0
0.891	1.1223	3089.791	462.7984	19.49389	0	0	0	0
0.8895	1.1242	3221.623	478.5122	8.859446	0	0	0	0
0.8941	1.1185	1238.434	44.64011	244.8625	0	0	108.7844	0.666667
0.8922	1.1208	1247.41	52.76714	255.966	0	0	102.8339	0
0.8876	1.1267	1248.091	53.59477	258.4616	0	0	98.65945	0
0.8875	1.1267	1258.846	53.93095	267.5393	0	0	97.97889	0
0.8956	1.1165	1307.331	120.9111	225.0453	5.221108	0	90.40669	1
0.8931	1.1197	1287.389	128.6108	194.5852	7.485553	0	0.93333	0
0.8895	1.1243	1292.968	129.78	190.9218	8.402219	0	2.281666	0

For peer review only

0.8877	1.1265	1293.796	150.6839	178.416	8.86555	0.166667	2.3	0
0.8944	1.118	1858.268	238.2539	0	0	1	0	0.416667
0.8918	1.1214	1787.575	242.6292	0	0	0.583333	0	0
0.8884	1.1257	1787.194	238.5697	0	0	0	0	0
0.8864	1.1281	1749.919	230.0777	0	0	0	0	0
0.893	1.1198	1210.028	112.8135	230.7546	0	0	76.58333	16.58333
0.8901	1.1235	1200.852	113.3852	248.8928	0	0	80.16111	15.83333
0.8871	1.1273	1201.917	111.3832	250.8196	0	0	76.91	13.16667
0.8855	1.1293	1237.299	112.6556	274.4541	0	0	77.57889	9
0.8933	1.1195	1312.798	74.27165	455.3968	4.781942	1.2	110.5424	5.427777
0.891	1.1224	1212.283	70.84619	525.4671	4.356669	1.2	99.11132	3.777777
0.8875	1.1268	1201.727	72.1693	475.2332	4.405558	0.8	95.47887	3
0.8862	1.1284	1193.543	87.25848	410.8459	4.277779	0.8	40.88222	3.166667
0.8938	1.1189	1557.353	141.6112	0	0	2.476667	0	5.333334
0.8915	1.1216	1577.139	144.9114	0	0.6	4.641667	0	0.916667
0.8883	1.1258	1586.602	152.6111	0	1.766667	4.7	0	1.916667
0.887	1.1274	1601.815	155.7422	0	1.15	5.250008	0	3.666667
0.8952	1.117	1296.53	120.025	486.8828	0	0	19.42103	10.04167
0.8928	1.1201	1277.867	117.3689	429.1179	0	0	5.65	7.833334
0.8892	1.1246	1258.247	120.5361	443.3112	0	0	4.45	6.25
0.8875	1.1268	1243.473	126.92	456.4894	0	0	1.533333	2.486667
0.8914	1.1219	1060.995	88.88321	261.8248	7.375266	0	90.1035	8.070311
0.8889	1.125	1033.4	95.33599	253.085	6.719388	0	84.54555	0.916667
0.8856	1.1292	1015.693	90.72778	250.069	6.756331	0	81.43389	0
0.8838	1.1314	1042.935	98.25795	250.8398	7.554619	0	79.03445	1
0.8946	1.1178	1520.385	178.3317	237.4273	0	22.00001	0	0
0.8922	1.1209	1543.267	207.6356	248.0545	0	25.01111	0.666667	0
0.8889	1.125	1511.124	212.6703	252.0689	0	25.73667	0	4.583334
0.8872	1.1271	1552.227	212.4311	256.4278	0	31.15334	2.266667	9.416667
0.8948	1.1176	1068.087	106.5426	0	0	0	0	3.666667
0.8925	1.1204	1058.289	110.9139	0	0	0	0	0.9
0.8892	1.1246	1059.241	109.9205	0	0	0	0	0.15
0.8875	1.1268	1099.073	111.4488	0	0	0	0	0
0.8954	1.1168	2654.643	226.2218	1.666667	0	0	2.25	0
0.8929	1.1199	2773.671	243.5196	2.1	0	0	2.833333	0
0.8897	1.124	2848.693	257.7408	2.666667	0	0	3	0
0.8882	1.1259	2935.933	272.8171	1.916667	0	0	2.583333	0
0.896	1.1161	1340.869	94.50665	0	0	0	0	1.166667
0.8934	1.1194	1326.245	96.47	0	0	0	0	1.416667
0.8898	1.1239	1347.336	92.21524	0	0	0	0	1
0.8882	1.1258	1388.742	95.50246	0	0	0	0	0.416667

For peer review only

midwives	_ambulanc	ahp	science	stt	nurse_learr	nurse_supp	_clericaladr	_estatesma
142.2406	6.666667	269.1992	108.2098	145.6988	18.41667	763.7194	351.8616	0
146.6983	7.5	280.5536	108.6601	155.7028	2.666667	782.8587	368.5199	0
145.33	8.66	290.24	75.64115	158.4362	0.416667	765.0219	399.8952	0
138.7145	12.29889	303.5316	45.58503	166.9424	0	832.9024	432.8218	0
469.0958	0	997.3667	767.5034	759.6329	4.333334	2343.113	1869.628	2.5
466.2428	0	1078.646	819.074	813.9198	0.583333	2466.029	1924.489	1.75
466.4116	0.166667	1231.314	870.5703	893.8073	0	2623.683	2052.866	2.333333
165.1105	0	482.1222	77.26276	372.9046	1.333333	1102.197	636.2934	6.69333
155.758	0	484.6164	74.66553	398.4096	0.833333	1133.829	646.9388	6.69333
41.42888	56.71915	151.1415	43.12734	123.4274	0	366.9131	219.4376	0
42.64833	60.05027	153.1443	40.84012	120.5396	0	366.3778	219.5406	0
44.04777	63.67916	141.7732	44.47512	117.3497	0.114998	378.9298	236.4773	0
50.20443	61.34009	141.2874	49.72857	118.2517	10.15333	414.5788	248.63	0
538.4142	0	782.2802	578.1521	462.7652	1.083333	1558.152	1226.335	0
543.855	0.5	775.8034	583.4042	427.3003	0.416667	1513.371	1161.674	0
538.7268	1.666667	796.6235	596.1769	462.9954	0	1518.533	1135.823	0
517.3402	1.783333	797.2732	595.0442	529.9218	0	1566.201	1137.627	0
152.0599	9.167223	680.9853	75.13113	290.7444	22.25	1036.934	821.8963	0
142.4769	8.60389	698.7165	87.08835	290.0716	34.16333	1045.37	761.6727	0
133.1341	4.596667	697.7691	76.95349	300.5754	38.40778	1079.881	824.3312	0.083333
101.7442	2	578.7072	64.24738	302.5265	39.55333	1085.845	954.388	0.833333
88.79625	0.383333	295.1936	161.5962	118.6942	0	315.7201	307.8796	0
86.40154	0.92	289.2721	162.923	118.5803	0	328.2061	318.0009	0
88.14153	1.038889	330.0502	165.3938	124.7237	0	365.3978	364.9553	0
90.62264	2.763337	343.4043	169.3797	140.1579	0	400.3864	401.5157	0
25.6039	0	87.36404	36.02256	44.31057	0	209.3122	189.0457	0
22.81557	0	87.37732	35.03645	49.61779	0	235.5773	188.5533	0
17.75779	0	88.45295	36.11556	41.61723	0	252.5869	131.2228	0
2.856971	0	87.11593	34.48	41.92971	0	249.6824	59.18612	0
55.57534	0.5	96.86406	13.54648	72.25414	0	327.6093	211.9652	0
52.50575	3.319999	102.7149	15.20048	72.06784	0	376.2832	141.0399	0
53.73167	3.246667	104.807	17.0556	73.91881	0	348.6675	138.7078	0
59.51611	5.78	101.1217	18.4767	69.80926	0	349.9902	148.4084	0
152.1053	0	377.0859	258.2192	352.4229	0	1163.059	682.4044	0
147.967	0	392.3057	261.0741	380.1568	0.25	1147.23	704.087	0
159.7122	0	407.2988	254.7984	391.3543	1	1156.685	747.2336	0
181.7445	0	452.6769	265.7946	404.652	0.916667	1183.697	775.1793	0
81.9204	4.683333	382.2892	89.80003	293.8163	0	754.8383	429.5072	0
80.6155	5.573332	383.447	88.70224	331.6454	0	800.0256	364.0199	0
83.13801	6.675556	388.3877	89.51447	346.8828	0	815.7766	392.2187	3
82.96846	6.920002	459.1111	91.71368	358.5883	0	850.9042	467.6177	3
193.5783	0	278.1459	80.53779	176.8293	2.14222	579.146	561.2909	0
191.9534	0	290.2061	81.50999	186.8253	0.983333	596.502	588.0034	0
195.6976	0	292.69	79.87165	197.2703	0.05	637.3433	564.5321	0
202.9548	1.75	292.8428	84.54805	201.687	0	688.0866	546.5259	0
121.5572	0	245.3078	83.80928	116.9228	0	622.982	347.4597	0
129.0138	0	245.6805	87.03041	123.5392	0	650.4188	356.5255	0
126.1366	0	255.0283	92.13582	130.9575	0	682.3807	365.0412	0
155.984	0.151111	296.7901	106.033	144.7764	5.3	840.5948	472.2443	0.696667

For peer review only

283.5129	0.887777	471.4365	233.5085	328.8352	9.244446	963.9343	966.782	0
275.4678	1.15	491.6885	189.1054	331.6098	2.931112	1005.031	908.5391	0
284.5478	0.916667	498.323	157.1958	338.2677	0	1050.645	828.4645	0
311.8512	1.583333	510.9123	180.415	350.5562	0	1109.527	746.0447	0
147.8998	0	142.0289	55.79522	97.09766	3	384.7587	233.563	0
130.0764	0	153.8961	59.02811	108.3377	1.583333	419.6804	247.2499	0
127.9988	0	164.9856	60.38478	115.1277	0	449.3832	271.3048	0
137.9272	0	188.0439	54.91811	113.1632	0	448.2578	289.4996	0
130.1841	0	157.4775	52.97554	76.37474	0	334.0911	216.2245	0
135.7095	0	156.3603	15.77001	79.88363	0	343.1728	218.7442	0.583333
136.4217	0	165.4287	21.21001	74.20753	0.083333	381.3052	222.1433	1
134.7767	0	156.8882	23.69667	79.61142	0	444.0808	229.4693	1
156.1831	0.5	120.9642	48.72835	93.33685	23.61889	327.8599	343.0632	0
162.0542	2	137.889	54.04498	86.29461	22.22556	326.4441	354.7485	0
169.1606	4.083334	143.476	57.25771	89.21601	12.83333	342.7766	347.5433	0
171.9226	3.75	152.821	59.53893	96.70097	6.833334	345.8576	382.7595	0
110.8883	0.166667	210.0028	57.91645	102.7128	0	730.9009	304.8567	0
116.8322	1.333333	220.709	57.8059	110.505	0	787.2493	306.5463	0
120.3938	1.75	245.4438	61.7009	121.2239	0	762.7737	315.6255	0
129.1805	2	262.1172	64.07918	127.6746	0	836.8936	343.9519	0
63.18835	0.333333	123.1238	67.83652	82.26688	4.583334	449.5458	166.9746	0
63.4089	1.395556	127.5549	71.5236	81.10304	5.5	477.2405	143.393	0
64.56112	1.937774	129.8649	71.28487	80.77785	1.083333	493.5092	144.2948	0
67.50056	2.391102	131.1699	71.10686	79.87521	12.90445	519.1229	155.9289	0
151.4622	0	201.4987	76.02419	107.2933	1.397777	598.4225	352.5014	0
143.4033	1.416667	202.7047	77.59121	114.7922	2.666667	561.6977	343.7001	0
140.6793	1.806665	202.9391	68.15308	116.0306	1.5	536.8114	325.0201	0
141.7292	2.774442	210.1234	61.49221	118.51	0.416667	512.1957	320.8738	0
76.59605	0	251.9961	119.7985	184.8846	0	827.3378	544.5746	0
87.39799	0	258.8251	119.9491	193.0313	0	863.4869	546.4145	0
118.18	0	274.0104	121.0474	220.856	0	899.1032	542.7847	0
121.3711	0	287.8189	117.2766	219.7821	0	912.5721	554.9197	0
123.8983	0	209.0323	173.0647	175.7974	0	688.9418	497.4812	0
132.7022	0	230.6347	176.2411	189.5999	0	729.1709	433.9218	0
138.9444	0	245.5641	178.6056	208.6368	0	734.9133	434.6956	0
139.4772	0	257.1228	179.0532	223.0633	0	765.6457	385.7747	0
104.29	0	178.4759	89.10867	100.5054	0	591.5607	373.9493	0
104.0372	0	258.7562	89.80679	127.2493	0	594.028	414.5815	0
103.1072	0	269.5737	88.73423	137.6746	0	598.9271	434.9605	0
103.2467	0	288.6641	87.13457	127.8853	0	634.9822	456.9867	0
66.55019	5.363331	280.8368	63.50254	103.0436	3.583333	550.7749	191.8596	0
62.61372	7.13	202.7152	63.46477	105.816	0.416667	505.4575	166.1958	0
63.52317	9.342219	202.5236	61.79255	112.8462	0.416667	538.9139	173.9506	0
62.94062	9.47555	209.961	61.86654	113.9741	0.166667	581.6401	193.6867	0
92.38801	0	149.0707	15.10944	89.85172	0	332.2473	207.615	0
87.91624	0	155.0541	14.05	92.93435	0	374.5433	220.1017	0
87.79083	0.916667	155.3574	15.90889	94.60724	0	373.4994	246.365	0
87.14633	3.75879	149.43	15.53091	91.88609	0	408.5721	254.9255	0
167.4834	0	174.7791	89.18445	99.47511	0	550.7981	280.5047	0
174.5256	0	181.6515	95.41556	111.4073	0	569.5444	295.268	0

For peer review only

174.5842	0	196.144	93.913	120.7462	0	576.6066	315.7743	0
175.3874	0.658889	229.3353	101.3146	136.0553	0	642.2089	379.6522	0
150.4217	0	511.5622	186.8389	218.2	0	1237.2	595.0693	0
156.6778	0	544.1281	193.9781	224.9245	0	1214.085	557.4517	0
157.1061	0	505.6212	198.7806	236.1708	0	1245.427	525.7684	0
158.9511	0	470.6088	219.1483	248.2898	0	1249.882	549.9639	0
69.63889	2	258.7083	52.30679	121.5475	0	567.807	256.7886	0
68.38666	2.333333	258.9836	52.76122	121.5636	0	574.8659	265.4247	0
69.93499	1.333333	257.3137	54.05663	115.9502	0	618.6915	284.6715	0
69.60221	2.133333	270.1226	52.33051	115.0801	0	654.4874	295.2265	0
80.53667	1.69	173.4951	66.54402	64.83356	3.666667	331.8246	235.2764	0
76.02611	2.653333	174.4358	96.65332	65.55249	2.583333	368.6687	225.9215	0
70.80111	4.797775	188.1599	110.76	68.32536	0.916667	374.6787	221.6705	0
70.97444	2.19611	192.8508	117.4135	76.49702	0	407.8817	219.5123	0
86.72444	2	136.0715	29.70556	95.18843	0	415.2862	273.8505	0
91.16	3.083333	137.3283	28.83333	97.7828	0	437.3984	273.351	0
83.63333	6.099997	138.3643	27.58556	105.9039	0	441.0047	276.1722	0
83.36888	7.07	133.1738	28	108.061	0	505.1082	285.8472	0
150.095	0	238.318	141.8834	124.2166	0.583333	710.2849	310.4082	0
148.2	0	255.911	143.4306	135.5681	0.416667	700.1007	320.7122	0
141.5156	2.976498	266.2357	142.3515	140.7494	0.083333	732.5315	309.115	0
141.9511	1.805558	277.9674	138.266	137.0428	0.083333	768.7236	299.2481	0
122.9815	0	259.4838	106.418	141.2052	0	536.5826	330.3379	0
129.3961	0.102222	257.8376	103.9004	146.1042	1.453333	555.3543	336.9489	0.333333
133.3055	0.86333	266.3949	105.4942	152.0931	3.303333	572.7457	336.4426	0
132.3778	3.411109	268.721	102.1028	153.9213	23.88667	583.488	358.9897	0
115.8274	0	127.2994	75.01514	80.54711	0	434.4253	202.0258	0
103.0916	0	145.5319	71.12226	83.20458	0	424.0078	228.0027	0
107.7472	0	146.3575	72.62282	91.2459	0	441.851	151.9249	0
121.5683	0	152.3182	72.54895	93.2158	0	450.1083	139.6267	0
157.194	0	167.754	52.26222	107.0624	27.17111	721.2306	294.2343	0
153.1192	0	173.4195	48.83231	106.4922	33.31667	743.8654	303.5393	0
152.432	0.666667	182.542	46.22451	114.692	40.42111	724.8883	325.7009	0
157.0774	1.181818	198.1473	53.94	106.0392	59.65818	743.3051	366.4043	0
119.98	0	230.591	62.98888	118.1314	4.375	485.7943	354.8707	0
123.6333	0	224.8243	152.1273	117.8853	20.89111	498.2603	351.8336	0
212.366	2.100002	461.0398	232.6046	246.844	15.77444	1002.407	621.3938	0
241.1207	1.955557	537.1008	253.8259	285.5215	5.617777	1167.795	699.4713	0.166667
267.9047	0	344.2532	149.6347	221.687	27.47555	1221.655	783.7921	0
265.2949	0	376.1959	155.9141	224.4007	20.63666	1269.53	795.334	0
264.0071	0	396.704	169.5996	231.7566	10.30333	1299.394	751.079	0
286.6802	0	414.0527	165.1274	226.8268	9.38666	1288.945	738.9057	0
33.9389	0	233.3076	93.45889	123.3966	0	630.4504	332.9615	0
35.13112	0	240.021	97.11244	134.0033	0	641.4371	308.3531	0
34.46278	0	253.0403	93.72932	137.385	0	663.0313	312.765	0
33.43333	0	257.1415	88.02709	145.165	11.58333	689.284	322.1733	0
54.68277	0	203.3198	16.08053	151.2245	2.464845	409.3409	330.0449	0
52.38026	0	165.0824	15.37386	158.2378	1.136664	398.6803	306.4633	0
51.40163	0	164.9786	15.02942	153.9495	0	379.8855	292.9574	0
134.7044	0	317.7646	151.2385	165.7117	0	606.3982	513.3428	0

132.12	0.25	313.134	158.7138	171.1713	0	599.4775	531.5998	0
136.2622	0	324.5147	162.5894	184.293	0	615.5951	561.3905	0
146.9205	0	308.4159	160.1429	182.7142	0.993333	644.3127	577.6564	0
2.29333	0	299.7001	60.83601	212.0086	0	518.0262	395.1014	0
2.269997	0	308.3994	63.05507	211.3489	6.833334	576.0942	410.6225	0
2.346663	0	318.9281	62.86031	221.451	1	683.9063	415.7461	0
1.84333	0.1	576.0787	221.1371	386.8841	0	1238.159	643.5192	0
257.532	0.916667	335.7696	175.4014	165.7169	28.25	725.782	587.0498	0
272.0499	2.943333	351.2912	190.3204	169.7837	45.75	739.5136	651.1971	0
271.8521	2.833333	347.3978	199.4103	161.9589	35.91667	756.2869	660.3438	0
264.0494	1.083333	356.4498	213.0056	163.7488	27.83333	787.5245	701.8071	0
111.2542	0	141.9933	81.21364	114.188	2.933333	389.4203	266.6765	0
113.085	0	154.4061	84.04974	117.0151	1.6	416.0023	270.8702	0
114.8238	0	165.9557	85.3164	124.5999	1.6	464.871	291.4712	0
117.5237	0	172.4634	89.33529	130.1153	0.8	500.7405	320.9095	0
92.01224	2	281.1358	85.03383	141.7255	1.72778	580.2838	310.7981	0
88.28894	2.5	299.5657	87.22382	148.9707	0.494445	592.5286	318.9049	0
89.45839	5.083334	298.3456	87.47765	145.7902	0	585.1649	321.493	0
92.15447	6	305.4573	96.14401	143.9412	0.038889	613.8124	333.0007	0
92.88416	0	188.8817	83.86667	142.4275	0	583.7158	144.6217	0
93.01167	0	194.9783	84.24917	169.9975	0	639.995	149.2808	0
96.06583	0	206.5467	56.23	166.3842	0	717.3767	140.575	0
100.775	0	214.8442	32.7625	178.6692	0.083333	672.09	97.18083	0
128.3981	0.166667	217.9392	101.818	144.0205	0.25	552.5413	312.1482	17.00667
215.7514	3.356667	284.9593	135.6925	203.1322	0	835.9764	439.562	16.63333
213.8816	3.5	296.8193	144.0663	205.2	0.066667	905.2572	446.5717	15.65
206.2177	4.25	293.1327	146.5951	215.9778	0	961.3323	483.2062	2.8
67.95795	0	120.1583	28.44333	98.32517	8.5	362.6531	209.6838	0
69.824	0.5	122.7634	28.41111	105.195	8.583333	387.9223	207.2694	0
68.82511	0.333333	131.0942	27.46833	111.7473	3.516667	443.1869	206.7963	0
67.04733	0	125.3886	26.20778	115.0456	0.083333	506.7783	214.2881	0
116.9187	1.666667	231.7187	55.22341	165.9957	5.87	479.1669	420.121	0
116.9509	2	260.6164	59.51552	159.0644	5.453333	539.1224	429.0414	0
125.2532	2	296.5496	64.67646	157.6475	5.666667	563.8484	428.2481	0
91.79112	0.416667	160.4921	27.93	109.8767	0	391.3036	293.5393	0
85.44112	0.416667	222.2503	33.03889	125.6306	0	413.8884	319.0926	0
85.70723	0	278.3342	38.87556	152.1489	0	473.1329	334.4265	0
88.02668	0.416667	300.2302	40.35667	159.8571	0	532.2051	350.535	0
162.3755	0	435.6136	552.3267	294.262	0	873.9577	707.5714	3.416663
171.0933	0.105556	457.6331	502.6031	297.0718	0	942.0183	752.2871	7.828886
169.9389	1.406025	475.2855	496.7644	307.7125	0	1115.24	723.0838	9.663333
179.4741	0.873125	506.8082	498.1753	324.2252	0	1173.091	622.5572	13.72445
136.5664	0.4	370.1724	166.2192	145.2861	0	1105.79	486.886	0
138.4958	0.333333	470.6575	175.4838	167.475	0	1285.676	483.0551	0
135.3646	0.142222	498.1216	186.0277	178.1397	0	1339.25	497.2265	0
110.3145	2.269997	511.5811	195.2298	183.2952	0	1327.393	567.5066	0
188.2593	1.833333	484.977	278.7854	318.2008	0	1360.905	814.4683	0
198.8974	1.5	514.514	279.7617	325.7424	0	1497.98	859.837	0
207.9618	7.083334	532.579	292.4489	348.7438	0	1587.969	900.5263	0
210.7274	15.24	540.4081	320.2456	369.7417	0	1744.581	934.1662	0

220.8479	0	761.6074	516.7793	636.3403	7.52	1758.436	1601.692	0
220.8693	0.23	771.6166	531.1768	653.3856	2.566667	1850.657	1700.03	0
220.8385	0.8	795.794	555.1358	671.1384	3.333333	1827.063	1712.592	0
226.5028	0.966667	813.5815	564.2856	675.786	26.33	1950.533	1775.194	0
140.4805	0	295.373	245.2455	286.0503	0.213333	841.7457	500.5942	0.416667
143.516	0	302.6749	245.8052	289.3159	0.993997	910.1838	500.0372	0
146.1021	0	313.3471	245.628	286.1485	0.064442	961.3321	515.5472	0
159.5549	0.583333	327.1561	239.0724	286.5813	0	1018.152	527.795	0
147.9283	0	243.6218	155.0947	105.2314	0	556.4973	498.1131	0
145.5888	0	254.3281	135.714	110.7463	0	580.7756	511.1115	0
152.0161	1.333333	262.5228	140.6716	128.8024	0	581.6034	488.6757	0
155.005	2.416667	282.1129	137.2213	144.6024	0	631.6673	463.8325	0
251.0776	0	931.3017	328.0813	636.2313	8.978334	849.6379	1128.357	0
249.2301	0	1016.127	349.9817	682.6581	11.9725	973.9741	1175.909	0
252.2209	0	1082.22	378.4996	738.5574	10	926.7397	1234.997	0
259.8377	0	1155.174	391.6698	809.9686	7.25	1090.956	1279.429	0
265.33	0	348.6997	140.8043	151.6599	25	746.5242	453.1211	0
275.5855	0	359.9224	138.5628	168.4171	7.416667	750.9345	461.8058	0
279.4804	0	374.1079	143.9529	185.3011	1.75	803.52	490.4086	0
283.0199	0	391.0393	142.7118	219.4935	2.333333	890.4128	502.1419	0
125.8722	7.5	236.9205	24.30993	97.87544	14.25	388.8393	374.5025	0.25
118.1999	5.833334	243.3752	24.24667	98.81444	7.5	407.6624	386.3018	0
113.3666	5.666667	237.5742	26.09611	100.6689	1.583333	436.3223	375.0219	0
121.7861	3.416667	231.1585	27.46278	111.0928	0.166667	471.574	403.134	0
175.1724	0	565.4114	346.0254	283.4998	0	694.8481	824.4421	0
185.2046	0	580.1169	356.3317	307.8557	0	692.743	851.4906	0
185.175	0	594.945	369.0828	348.2819	0	651.1367	814.3543	0
183.4139	0	574.9164	383.4033	357.6532	0	667.7656	803.1061	0
92.21059	0	378.7501	17.33247	105.5948	10.45	580.6892	367.2774	0
94.44103	0.051111	382.0075	15.73192	114.2912	1.1	607.6108	386.802	0
102.6078	1.237772	393.1505	17.48237	119.6124	0.166667	662.9642	419.6885	0
103.6358	2.296667	405.419	18.62668	118.6247	0	693.0912	440.8644	0
230.9878	0	428.4686	276.8104	308.4643	0	1754.748	742.6944	0.08889
227.7278	0	431.3288	276.4538	337.7343	0	1753.675	779.3926	0.26667
218.0433	0	446.3443	278.3902	353.8685	0	1738.452	786.0686	0.488889
216.1326	0	464.2117	279.0507	367.9861	0	1683.559	800.0564	0.8
99.60873	0	125.7865	24.40133	100.8455	11.65	450.7139	225.9418	1
104.9245	0	128.9713	25.03333	106.274	11.58333	457.9481	232.5149	1
104.7	0	131.0308	25.668	108.5861	10.33333	463.5879	228.2454	1
136.4578	9.58556	341.7512	128.783	152.5896	1	743.1285	621.3956	5.416667
143.5328	9.013339	336.0938	125.6732	152.5594	0.416667	709.0079	613.4383	6.75
152.1306	12.00167	348.4203	125.1974	158.3555	0.083333	758.2632	632.1279	5.416667
155.3306	13.93334	350.5252	124.6711	156.1093	0	789.8686	661.0216	4.53
56.93556	1.166667	238.7921	64.88007	67.96678	0	385.1149	280.9022	0
58.94998	2	152.9877	67.8869	72.30079	0	352.2262	261.993	0
58.37222	1.666667	158.4831	64.44834	74.4017	0	356.0876	262.1677	0
57.16777	0.972221	165.3297	64.53334	70.75909	0	364.466	246.2249	0
90.31494	1	185.8669	76.57129	126.3311	0	625.4816	324.566	0
93.93384	1	189.842	80.54716	132.1888	0	637.8688	321.2399	0
92.45439	1	191.2182	82.64454	142.8977	0	656.5751	318.9487	0

For peer review only

94.11939	1	200.4199	81.15231	153.6363	0	750.1657	280.8561	0
368.4194	5.105553	513.7844	275.4227	469.2856	67.5	1049.774	1237.883	0
361.8631	2.446	558.5316	245.2637	489.3012	99.83334	1152.552	1256.65	0.5
376.6115	0	566.0959	243.7871	502.2448	77.25	1199.553	1263.071	1
356.3943	0	590.1807	254.9629	517.1824	41.66667	1230.658	1327.24	0.916667
107.6534	0	188.691	106.5211	133.4676	0	636.6089	425.4244	0
106.176	0	204.3955	106.7061	136.1294	0	702.1096	482.4313	0
104.0594	0	215.5072	110.1476	140.8988	0	709.9578	494.7515	0
107.1328	3.083333	220.0861	118.5561	138.0425	0	730.1226	523.2974	0
130.2948	0	311.9378	228.3534	216.2221	4.333334	869.4228	417.2994	0
128.7098	2.396667	331.9358	233.1992	221.7145	1.25	953.3451	469.3736	0
125.5359	2.453332	351.2704	242.3299	243.056	0	1016.287	486.5998	0
130.0887	0.766665	356.085	245.8998	266.1067	0	1112.421	568.6289	0
176.3233	2.209997	400.3867	307.0723	223.9433	25.40249	999.4285	528.1224	0
177.6253	4.484444	426.0757	323.0519	244.9676	3.845823	1057.732	558.9321	0
175.5901	3.41	440.6136	330.4196	248.3695	1.105778	1092.933	577.5638	0
193.9012	1.583333	431.8807	331.2035	255.1167	3.646667	1133.529	589.1227	0
130.0182	0.916667	395.1307	48.12103	198.813	10.63333	341.2122	286.8522	0
140.7122	0	408.8015	47.23658	198.6501	9.5	347.9209	265.7489	0
144.4172	0	426.9417	44.3288	187.5728	3.799167	365.3493	260.7265	0
147.7615	0	445.2345	43.88325	215.5676	2.620833	397.0185	262.1018	0
164.8869	0	389.2108	235.0045	208.1098	16.66556	1033.041	774.5627	0
172.1159	0	388.2313	245.4961	215.9101	2.333333	1033.117	735.0245	0
182.0557	0	393.0214	341.0886	220.7423	0.583333	1087.016	762.5263	0
194.6804	0	408.7259	445.1007	218.6973	6.054447	1195.868	780.5816	0
106.3689	0	299.9423	72.55444	198.3134	1.5	677.5749	367.9854	1.334447
108.2778	0	323.9842	62.4911	202.8002	2	677.0112	365.2895	2.30667
111.7607	0	323.9721	61.11778	206.6104	1.555556	701.8199	359.0587	4.065183
69.27168	0	197.5288	61.23541	61.1368	0	463.5088	242.7454	1
66.65223	2.216667	197.0545	59.75945	69.34569	0	479.3313	232.0962	1.87667
67.68555	1.85	193.4876	56.51223	79.10901	0	482.8548	218.3858	2.624448
65.45165	3.456667	195.6208	61.3264	87.29012	0	506.2993	239.457	3.349998
74.7618	1.713333	76.58957	10.59556	87.80389	9.916667	268.4389	171.7421	0
77.20235	2.533333	83.42425	8.526673	87.14001	9	259.5589	186.694	0
82.10412	2.306667	85.74147	10.03445	94.32112	0.583333	266.8217	195.0354	0
82.32389	2.556667	85.68591	10.64445	101.0822	0.5	280.5161	198.5567	0
188.7894	4.353334	386.5176	243.0912	255.9249	0	714.6855	693.5698	0
193.6933	2.8	405.3983	246.4961	264.9851	0	754.2027	691.7847	0
192.42	1.25	428.2145	248.078	283.8392	0	806.6691	707.9146	0
188.6472	5.193333	459.9247	252.2261	304.426	0	958.8027	777.7942	0
149.6713	0	323.0218	190.0852	206.2545	5	793.3125	563.6245	0
153.545	0	350.3372	188.6431	216.3917	2.833333	814.4445	616.4289	0
0	1.83	405.0974	188.4789	310.4075	5.312223	1040.17	463.5041	0
0	2.199997	428.0277	193.553	384.8866	1.75	1158.705	501.0041	0
0	3.616663	450.6715	203.9462	410.8798	0.25	1214.987	521.7752	0
0.083333	3.572219	673.156	222.1729	432.9851	0	1364.66	620.6945	0.75
208.0946	0	355.1782	91.82394	136.3894	16.66667	687.9131	528.1124	3.916667
207.9084	0	365.9403	97.52966	137.2351	10.16667	769.8975	541.1563	1.6
199.985	0	379.071	93.70607	131.4079	4	796.3168	558.9562	0.706667
211.1063	0	396.8135	98.86971	136.3514	0.583333	845.8131	584.2959	0.706667

For peer review only

95.72667	0	221.1065	55.73732	50.21361	0	504.9529	285.6735	0.25
95.17667	0	235.7672	48.54032	48.14555	0	585.1903	296.8385	0
97.22222	0	233.0475	50.7461	54.72833	0	605.0566	299.5952	0
100.39	0.083333	248.9446	50.80776	72.05444	0	618.0151	330.8608	0
133.2422	1.696667	355.1933	83.20001	115.9147	2.216667	617.1171	425.2297	0
145.6367	21.67633	397.3279	83.50874	110.2284	1.433333	706.4326	457.9734	0
148.9911	18.93944	216.7011	85.1256	109.6303	1	568.0709	391.5789	0
150.5094	16.80611	232.0241	91.3141	108.7301	0.95	628.2175	418.7075	0
175.4811	4.433328	262.1661	146.9293	150.7471	0	798.4385	366.435	0
175.7425	2.344444	276.1019	144.8758	163.0995	0	869.0425	426.1946	0
170.4684	1.15	281.764	142.0086	165.5314	0	860.2626	429.7052	0
173.8295	0.772222	281.1364	147.4001	168.8048	0	951.2759	435.1723	0
147.6617	5.406661	153.7912	51	92.27834	0	474.5176	263.1478	0
143.2712	6.732214	164.4843	54.46611	100.7211	0	501.7956	265.1159	0
145.4206	6.063327	177.1247	78.40723	105.4872	0	499.2903	245.0606	0
146.9589	4.248884	177.602	77.18833	104.3906	0	486.9635	241.8543	0
121.7195	3.25	172.5182	96.83787	92.62366	0	439.6015	355.8427	0
122.7442	7.673333	174.3074	86.51397	95.78255	0	455.3004	362.0873	0
122.798	12.77333	174.2246	93.97797	101.8864	0	473.1147	370.9167	0
118.4587	17.072	171.359	94.96693	103.8046	0	482.1232	370.4405	0
0	0	347.0829	2.5	220.9322	2	521.4717	209.2034	0
0	0	357.1746	2	195.2265	1	504.5327	220.3708	0
0.5	0	321.556	2	205.0684	0	451.8233	217.1169	0
69.80522	11.44	413.9548	59.06078	210.3231	0.583333	648.0206	389.747	0.253333
97.78389	6.91	104.3035	90.4976	88.46474	0	656.8336	196.9301	0
105.1633	5.97	109.4621	92.79849	94.78338	0	679.8983	192.2275	0
117.3744	7.907776	110.3076	97.27537	98.02601	0	605.0422	279.9776	8.511112
123.5895	6.619999	118.5878	92.98741	106.3171	0	606.1583	442.5939	24.57667
160.381	0	187.4492	112.1219	138.0077	4.751111	596.8259	437.4491	0
164.1857	6.45	195.2369	111.9138	144.0805	2.833333	571.6243	454.2016	0
155.6354	9.03	201.5539	107.8827	147.2744	0	578.3384	475.3794	0
155.0304	8.683333	198.4858	115.2588	148.48	0	630.1275	491.1534	2.5
75.51054	0	144.8243	133.7789	88.24056	0	491.2317	167.3307	0
80.94943	0	154.3253	132.8195	93.19112	0	463.3261	190.7144	0
82.57442	0	159.2904	136.695	99.21529	0	480.4788	198.7144	0
83.07828	0.75	172.2306	137.6906	101.5728	0	499.0966	212.4847	0
159.0276	0	307.9445	125.5557	173.1626	7.96	818.3173	481.5934	0
167.8792	0	315.0049	122.3505	177.4983	2.686667	838.5726	486.9192	0.416667
168.3721	0	309.9492	123.4119	175.613	0.9	859.6696	445.5335	1
163.4479	0	308.7404	117.9864	178.1672	0	853.0284	362.6595	1
158.3022	0	126.6156	80.00109	99.08392	17.75	554.7439	428.8915	0
154.0422	0.583333	136.4842	75.54967	87.57391	17.08333	579.7435	455.6939	0
156.1978	1.5	145.9945	43.11411	87.36613	9.583333	504.3284	414.6915	0
155.4606	3.416667	155.4764	40.65245	96.87943	4.083334	504.4429	439.1247	1.277775
0	0	392.4165	291.1756	268.4273	0	699.674	380.9178	0
0	0	408.0548	285.6845	294.4269	0	763.8962	366.5123	0
0	0	405.8634	275.2217	299.044	0	786.5198	358.1493	0
0	0	402.3766	279.548	314.3606	0	807.9488	357.2735	0
134.7761	2	156.8996	95.1878	120.0094	0	542.7271	427.1736	5.738891
131.2879	1.166667	162.5618	101.13	131.7426	0	555.8952	429.7228	6.672224

129.4439	1.77111	159.9592	104.2406	148.8934	0	570.2024	458.0014	6.86667
137.0164	1.81333	163.4795	101.8994	146.0237	0	620.5914	489.4323	7.587879
311.1861	0	248.1567	52.66444	201.6629	0	578.0222	458.417	1.37333
330.5243	0	257.1833	41.26667	209.3223	0	589.8294	548.3832	1.37333
331.2911	0	266.7284	43.92056	205.1207	0	603.6935	532.9675	1.37333
332.987	0	259.6407	41.25	231.7492	0	605.5709	514.1666	1.37333
84.97453	0.363636	56.728	21.16485	65.17576	0	220.8794	150.0145	0
137.2244	1.916667	114.7623	76.40223	68.7559	0	388.7173	386.3098	0
142.9413	1.5	116.9426	80.26057	82.59645	0	389.014	376.6398	0
144.2084	4.393333	119.8622	81.30723	105.8889	0	454.0984	325.3696	0
137.1298	4.943333	119.4994	80.71833	111.815	0	475.602	335.0703	0
189.8156	1	386.6905	85.3289	97.77245	8.916667	357.3665	298.2914	0
194.7261	0.333333	387.8667	81.37779	101.7654	6.583334	361.2373	310.7505	0
192.4522	0	387.0888	79.16946	114.3531	9.75	361.5997	299.6171	0
196.8098	1.32	395.8643	83.00333	139.6374	3.75	357.447	307.8587	0
366.2861	0	519.0839	241.3553	338.6217	22.5	1461.499	734.605	0
364.8579	0	536.3204	230.199	331.5904	20	1502.431	760.1303	0
71.86722	0	194.7286	159.2217	94.60457	0	471.0403	338.5178	0
70.40889	0	240.0421	161.2133	105.7718	0	512.3695	354.0338	0
71.76334	0	244.5184	165.9878	115.7707	0	512.7556	370.0228	0
80.90778	0	253.2198	173.0622	110.3545	0	542.2344	379.9641	0
340.0861	3.75	837.4193	673.0803	707.6306	0	1744.245	1433.238	0
340.6988	3.166667	843.2579	667.9243	788.7919	0	1928.449	1500.533	0
340.5039	4.166667	879.2678	671.5898	795.794	0	2005.141	1507.751	0
353.611	4.25	921.1645	678.1573	846.1845	0	2109.571	1541.656	0
110.9323	0	232.107	35.1488	113.5723	0	923.5895	395.9805	0
109.5261	0	237.2569	34.98714	123.2312	0	916.8227	388.6122	0
110.7733	0	230.5093	35.15163	124.8034	0	924.4687	390.7145	0
110.7939	2.316667	394.8454	38.28777	142.659	1	1027.701	476.7289	0
0	0	421.8426	358.5117	256.4142	0	954.0516	488.7419	0
29.67084	0	489.2418	389.5311	328.9109	1.333333	1062.442	594.7523	0
353.7651	0.916667	1019.856	625.5776	678.5823	11.5	2575.428	1317.655	0
349.1711	1	1047.538	640.3998	716.4262	1.333333	2742.703	1350.422	0
192.5423	1.25	476.1524	222.0621	400.9913	5.583334	634.9067	857.1481	0
198.8812	2.516667	512.4488	225.2034	397.4316	6.666667	669.1052	903.7271	0
188.887	1.291667	533.9238	235.7229	411.4718	9.916667	728.1298	655.1594	0
196.3276	0.666667	577.0567	240.9437	445.4249	5.666667	796.6399	708.0135	0
244.2783	1.25	668.4464	564.3785	512.9372	27.95205	1638.946	985.389	0
247.8394	0.660556	698.5928	577.799	535.2507	14.64722	1656.29	1053.385	0
245.4989	0.5	717.7503	597.7849	556.3637	7.398889	1669.033	1101.944	0
242.2017	0.5	731.6531	606.7368	572.7772	24.08333	1725.297	1121.369	0
227.721	1.1	327.5251	234.6793	200.9946	46.10111	979.6696	505.6885	0
226.6715	1.333333	337.1421	232.861	220.3332	42.59333	956.2694	510.4769	0
224.2896	2	340.5297	237.2437	227.7456	12.25111	1000.978	527.965	0
227.0911	2.25	348.0814	241.7121	224.6953	7.793334	1098.141	571.8854	0
136.1878	0	513.6314	130.0303	387.5433	17.51667	1032.425	710.1429	0
134.9294	0	519.5405	127.4053	415.782	17.66667	996.9677	730.2237	0
130.9483	0	528.6107	132.6849	400.3309	6.883333	978.9583	739.3237	0
144.4945	0	532.9951	133.0745	405.7831	1.833333	1028.97	772.3417	0
216.196	6.75	470.5882	214.8525	176.3922	0	940.5735	891.7868	0

For peer review only

219.2696	8.633333	503.6812	222.6684	189.3489	0	953.9617	911.3022	0
292.4771	6.472221	627.8127	276.4205	361.0603	5.166667	1330.495	1121.02	0.75
324.0552	6.133333	670.3729	295.3095	403.1825	1.666667	1556.29	1328.463	1
282.7763	0	536.3054	511.0147	381.836	0	1185.192	1255.69	0
267.2344	0	552.6923	508.4041	405.0754	0	1286.728	1213.141	0
257.2961	0	558.4335	515.2944	410.6117	0	1378.047	1189.713	0
258.7322	0	639.0409	519.4347	412.6277	0	1418.605	1248.244	0
115.4739	0	182.0594	83.34833	64.28297	3.416667	399.6036	267.2331	0
114.4844	0	200.8584	85.46777	62.41352	6	452.679	253.9305	0
115.5021	0.833333	204.7237	87.32999	74.40964	1	456.0186	253.1498	0
117.9311	2.55	209.6981	88.22705	102.9347	0.166667	492.5199	262.1619	0
129.7366	0	169.9407	80.71174	106.744	20.75	528.2484	302.727	0
130.2408	0	186.026	80.78841	119.593	19.66667	553.7803	324.1759	0
143.2958	0	188.2863	79.87976	127.7817	9.583333	594.0468	343.3379	0
139.5671	2.288887	193.673	79.3578	131.9055	4.083334	633.8441	360.4422	0
187.5878	2.833333	584.7578	209.5109	247.4578	0	1202.753	724.9855	0
186.7517	2.083333	586.0491	209.8886	265.8373	0	1213.646	751.2097	0.166667
185.3967	2	568.2863	205.4044	259.1133	0	1190.522	645.9728	0
185.5598	3.75	572.1383	204.1999	271.4066	0	1228.218	645.02	0
124.3935	0	249.2531	134.5459	71.09657	0	610.57	505.4403	0
143.7945	0.5	254.6401	136.0413	91.02651	0	667.602	513.0264	0
148.4445	2	352.7486	142.722	122.3753	0	773.3754	546.7661	0
152.3351	2	382.6357	144.8381	129.4795	0	781.6047	565.5759	0
212.1917	0	341.1547	343.2288	212.6819	0	973.2591	594.2146	0
221.1983	0	335.6188	343.372	199.5181	0	1000.775	568.8411	0
220.5855	0	357.6208	341.1851	210.0431	0	1004.891	569.8747	0
229.9322	0	384.7301	354.6713	229.465	0	1029.894	582.2463	0
183.1764	0	139.0065	146.6167	122.0775	3.166667	600.5322	474.0127	1
173.7394	0	163.6143	144.6972	126.1206	0	641.6885	461.3999	0.833333
174.7711	10.08333	174.1848	143.9299	138.7706	0	648.9421	479.8164	0.25
177.013	14.17222	308.7114	137.9467	161.8529	0.166667	690.5516	554.7762	0
221.9434	0	448.7227	212.8798	256.6716	0	1021.931	649.1038	0
221.6913	0	439.5626	214.2767	272.5231	0	1060.074	631.1811	0
226.0991	0	463.8579	219.4443	292.504	0	1135.83	646.6582	0
227.7269	0.5	489.5214	230.2981	304.2882	0	1310.158	725.6033	0
111.5206	4.161117	371.807	110.9248	118.5236	0.358889	590.5118	302.7167	0
110.5633	4.186673	384.5369	110.8354	120.3659	0	598.8301	314.5608	0
110.975	4.120007	377.5908	108.3404	116.4454	0	554.5938	335.9783	0
112.5233	4.17834	364.5902	101.8974	117.4699	0	561.5458	353.0906	0
97.2739	0	243.3756	26.78999	99.39986	0	343.3651	301.742	0
100.0178	0	178.6056	25.72332	99.27387	0	313.8633	275.0896	0
103.1837	0	166.4101	28.22332	105.4988	0	316.3428	263.5413	0
99.01222	0	168.8895	26.98666	113.9338	0	341.5117	267.7213	0
301.1934	0	636.1953	573.0902	480.3062	0.416667	1390.132	1103.795	2
303.8333	0	638.7655	575.2227	487.9353	0	1437.667	1218.374	2.5
332.0277	4.666667	519.2808	186.4785	224.7988	3.5	1151.477	825.2252	0
344.8235	6.416667	541.7534	188.595	235.7826	0.583333	1230.189	868.4531	4.433333
343.7471	8.661111	512.9622	187.3917	251.9587	0.083333	1342.918	903.6014	7.543334
330.1689	5.927777	486.9778	188.92	268.3576	0	1422.903	967.8764	8.6
164.9021	1	387.221	269.5819	216.8551	11.58333	949.7974	202.9997	0

156.8438	0.5	400.3571	271.1562	220.3431	1.083333	1001.451	209.2949	0
159.5216	0	417.9783	270.341	243.9847	9.75	969.091	206.1811	0
157.9866	0	428.9988	274.6409	258.6835	26.08333	906.4429	113.7101	0
188.4998	2.5	333.5173	167.7261	212.9113	6.214423	1193.156	519.4705	0
188.4103	4	343.9959	167.7317	209.5568	6.331648	1236.778	556.7375	0
186.8854	2.416667	344.0377	164.1524	208.7726	3.572766	1220.741	585.3731	0
188.8945	3.5	352.1562	172.0481	219.1933	0.839998	1323.433	575.6685	0
335.7351	6.84	556.7655	405.4684	386.091	0	1600.264	1266.25	0
338.7296	8.14	601.1854	425.0706	411.6021	0	1768.942	1303.966	0
340.2924	8.929997	628.7278	428.2054	422.011	0	1841.213	1334.862	0
343.0006	6.604437	640.0179	426.7359	429.4465	0	1929.386	1384.031	0
194.8168	0	357.6825	177.273	140.8115	12.5	593.8062	309.2227	0
195.5434	2.864433	346.453	177.9678	131.4132	12.75	588.1152	292.9772	0
195.5674	3.482221	342.6786	186.9613	129.0815	7.416667	576.4985	302.5812	0
185.9928	10.32334	346.2423	189.5739	140.4352	3.083333	637.0402	346.9576	0
135.9299	0	169.0982	145.4245	115.8896	0	531.1832	451.0259	1.5
138.0623	0	187.5723	152.7308	123.7472	0	578.8489	484.8461	2.75
141.4545	0	193.9974	159.2808	125.2416	0	568.5869	481.5982	2
143.8365	0	207.7901	154.8962	128.9632	0	576.5179	494.5168	0.25
158.5927	0	207.5841	109.8944	163.5093	0	589.9695	157.1262	0
150.2977	0	195.6321	141.2488	160.1443	0	589.2323	148.6933	0
159.066	0	192.7957	145.4594	166.1619	0	603.3377	147.1847	0
159.5477	0	228.5193	147.4193	165.3164	0	611.5324	143.4831	0
116.9733	0.916667	285.5191	91.95331	151.8048	15.16667	706.623	382.0945	0
115.7667	1	293.0276	86.48297	163.755	6.25	757.2507	385.5035	0
118.6567	1.133333	306.4671	86.65153	153.0972	1.75	747.8883	386.0147	0.982663
120.6483	1.75	318.8406	86.49825	156.96	0	767.0911	407.7737	3.543102
206.0204	2.616667	319.4353	171.9998	176.7074	19.66667	838.3082	416.2758	0
207.0054	2.29	329.847	174.7198	178.2367	16.5	856.8987	424.7692	0
207.3118	2.272219	337.195	176.9236	185.1442	5.75	854.2188	430.8402	0
201.8233	2.491109	345.263	175.6037	194.7708	0.5	928.8939	456.1823	0
101.3672	1.5	245.0794	85.82451	142.8501	0	499.8144	364.2693	5.811022
97.11111	0.416667	244.2932	90.11195	137.1396	0	518.8405	375.3471	5.273248
93.48554	0.916667	245.3051	90.83167	136.9694	0	519.6838	376.0694	5.146603
97.10108	1.533333	264.6185	88.53223	144.2903	0	548.6957	387.9988	5.083332
160.6694	0	332.0628	116.2464	175.893	0.416667	735.0625	389.4721	0
181.0237	0	352.2622	107.6996	180.4883	0.833333	704.3717	416.1864	0
169.7964	0	359.4982	108.2107	183.9345	0.4	687.6707	368.1888	0
167.0566	0	374.6352	109.6057	198.5397	0	727.6283	363.3103	0
344.1886	0	614.3196	455.3085	488.5011	0	1438.522	1325.949	2.838888
342.7812	0	628.9933	472.4783	523.3015	0	1495.715	1359.424	1.816667
330.492	0	652.8717	491.2824	542.0547	0	1648.014	1335.535	4.85
339.6931	0	692.9469	535.6874	544.1593	0.333333	1978.094	1549.513	5.25
110.1499	0	346.9928	129.3255	157.4972	22.58333	961.4253	515.8859	0
114.0049	0	380.583	131.0924	163.9681	0.666667	1045.784	566.2045	0
116.0897	0.25	397.7748	136.4099	158.6948	0.583333	1038.361	569.8463	0
124.2903	1	407.7269	142.4973	156.6745	0.916667	1056.973	581.0731	0
214.6726	0	495.2826	138.5611	198.0159	1.966667	1263.353	501.435	0
210.4649	0	518.3181	141.1523	202.9744	1.55	1342.334	508.6936	0
209.9689	2.04	537.11	139.9471	220.3091	1.066667	1454.666	531.3887	0

For peer review only

201.0744	0.083333	537.2531	144.0129	232.3674	0	1535.995	549.7099	0
189.1321	0	348.4128	249.4205	230.0644	34.33556	795.8213	688.6914	0
185.642	0	356.7719	258.1835	232.294	28.76667	874.4203	663.8384	0
191.9194	0	365.2071	264.5144	239.3635	18.5	940.9667	696.3846	0
193.7505	0	367.6969	263.3098	250.8405	10.7	957.8435	723.8781	0
198.38	0.583333	354.4569	235.806	164.1733	0	796.2771	461.0846	0
200.5717	4.102221	370.4624	234.2734	153.0866	0	820.5533	406.1119	0
198.8095	4.496664	365.423	172.2133	145.2355	0	824.9068	366.9505	0
198.8981	3.431111	381.5378	121.1597	155.5705	0	864.5824	374.7228	0
93.11945	0	397.501	182.1439	202.2118	0	948.4147	398.131	0
86.29057	0	428.0193	179.335	220.0409	0.6	1016.91	357.1865	0
84.11781	0	419.4247	183.5843	232.4944	0	1088.209	326.0291	0
83.53558	0	410.7737	183.4574	263.9276	0	1151.372	311.5403	0
145.7317	0	367.8468	210.1112	228.9418	19.25	802.8871	713.9727	0
152.3256	0	382.5822	215.9973	239.9001	14.91667	891.3234	742.8548	0
159.1288	0.333333	405.922	223.646	228.309	15.00555	981.0263	733.2672	0
162.0222	1.216667	428.6928	227.8496	240.0153	0	1106.077	759.5983	0
201.2195	20.65889	353.8579	151.9244	179.6314	0.15	1016.278	543.5181	0
200.8089	15.17501	340.0213	150.2059	176.2098	0	1037.036	535.7911	0
195.7028	17.32001	361.4401	151.0774	181.0066	0	1012.373	524.4423	0
194.2146	17.93556	385.9393	148.7835	188.3721	0	975.4738	533.3757	0
148.6517	0	350.7427	130.0515	145.1267	14.93333	793.6888	493.3241	0
146.63	0	402.1947	122.9004	151.1528	4.25	848.2311	517.5331	0
147.8416	3.091104	407.8449	124.5542	166.3204	5.916667	875.2264	528.5852	0
148.9755	5.957773	400.7355	124.082	180.9902	10.91667	885.8221	528.9163	0
206.8078	0	492.8163	117.6001	184.0954	0	1005.011	690.6718	0
221.2911	0	486.7015	122.1138	201.3145	0	1013.214	669.8126	0
235.0467	0	526.8436	129.6882	223.9381	0	1181.392	639.7466	0
243.8811	0	573.8294	133.0735	254.9222	0	1292.585	703.4111	0
182.8229	0	261.1928	167.0941	162.5788	2.433333	887.2726	418.9246	0
187.4388	0	262.8624	166.6474	166.93	2.016667	886.5416	430.7209	0
194.696	3	269.3794	161.7679	168.1191	3.516667	897.7673	428.5821	0
209.5021	3	283.3083	163.859	172.2026	1.416667	954.3185	446.0536	0
325.3758	0	503.7627	552.4872	222.7533	0	813.3729	858.0479	0
319.2537	0	547.3372	628.2092	222.579	0	893.5981	854.9064	0
325.9618	0	586.2544	651.9371	233.4352	0	910.0862	841.5129	0
329.0431	0	620.621	638.5891	296.7494	0	948.3575	863.2739	0
181.1004	0.666667	343.5126	111.3856	227.3828	33.18666	816.8655	464.7393	0
176.2564	7.571109	349.2293	125.2082	232.7712	30.25	857.6823	478.2402	0
179.1417	4.320002	352.6744	124.6152	210.4474	17.4	853.569	465.8321	0
185.7933	3.028055	354.9094	120.2885	201.8372	10.68333	876.1362	468.5178	0

For peer review only

_ambulanc	_healthcare	v75	v76	support_ah	support_st	v79	_seniormag	v81
0	0	0	0	47.83756	49.95	65.0641	31.69579	194.5968
0	0	0	0	51.83018	53.71666	71.22214	39.86647	205.8266
0	0	0	0	54.23103	58.99667	63.20636	37.93896	215.2558
0	0	0	0	69.02727	59.7911	56.23772	37.71367	227.7274
0	0	0	0	135.0331	307.5307	542.3549	95.25891	1270.19
0	0	0	0	153.5779	315.0265	530.749	100.2571	1411.718
0	0	0	0	172.7251	371.0644	534.079	128.5301	1570.14
0	0	0	0	78.47068	70.66454	61.19388	46.53333	440.3155
0	0	0	0	77.49122	69.33675	62.77301	45.93889	423.9725
40.35667	7.145551	51.21971	0	39.09785	31.04777	27.48667	35.47666	196.881
44.25444	5.106885	57.01723	0	39.68795	33.71222	28.84334	41.11666	207.3352
42.695	5.844667	55.34346	0	41.18496	30.96167	25.18223	37	208.1621
46.15445	0.988	55.55028	3.6	40.44493	34.12	19.82889	34.56904	201.1614
0	0	0	0	117.0089	165.2669	73.35011	39.46333	1479.118
4.34	121.4173	0	0	103.4867	139.995	68.42789	41.08	1555.071
9.346666	218.3918	0	0	105.2983	156.8276	69.4659	43.78	1620.553
153.6529	35.44133	0	29.76689	108.332	131.6147	73.07923	62.70945	1751.112
0	0	0.308332	0	112.5564	88.5177	49.5004	76.75	350.2568
0	0	0	0	112.0337	103.5897	44.9092	74.2	329.7002
0	0	0	0	111.1718	101.6622	43.32142	79.30681	332.7251
0	0	0	0	107.1501	93.72726	40.16142	74.43	264.4685
0	0	0	0	14.91333	23.40925	80.75871	30.31889	235.0221
0	0	0	0	18.86333	23.74667	79.27562	32.73333	246.4105
0	0	0	0	26.19	29.78778	81.48825	39.47223	252.1484
0	0	0	0	37.69395	33.65556	83.74671	43.13278	280.7043
0	0	0	0	20.33963	5.634444	12.87889	5.866667	110.7849
0	0	0	0	20.2821	6.673333	12.76888	4.25	115.1193
0	0	0	0	19.98191	8.158334	13.05889	3	170.6612
0	0	0	0	21.7002	10.70121	12.07636	3.345455	244.7379
0	0	0.8	0	16.56739	23.34938	7.588577	42.72556	142.3619
0	0	0.133333	0	21.65163	26.10565	12.68395	35.35166	148.9513
0	0	0	0	24.98999	36.77556	14.88499	29.81667	142.6554
0	0	0	0	24.67722	36.72501	16.78167	26.59001	154.4554
0	0	0.416667	0	67.07778	118.4255	124.8712	29.40667	445.9677
0	0	0.083333	0	62.31279	120.6228	123.605	33.26715	486.339
0	0	1	0	59.03556	127.2864	126.0604	33.75889	508.6978
0	0	1	0	59.84618	122.3933	125.9468	35.03333	549.9965
0	12.98889	0	0	76.42442	187.2545	59.03334	23.71246	579.0833
0	7.444445	0	0	95.47946	260.8366	51.47333	19.73333	541.4574
0	0	0	0	110.7815	278.9111	52.41443	17.5	543.0197
0	0	0	0	112.4675	283.5133	55.30943	22.55	606.7604
0	0	0	0	56.54489	37.40777	80.14538	13.5	408.7076
0	0	0	0	61.27933	46.64222	74.05593	14.75	474.9865
0	0	0	0	66.05543	57.05111	75.0215	15.41667	518.0295
0	0	0	0	67.52654	69.79569	71.1824	15.51667	576.9069
0	0	0	0	73.51378	80.18246	29.93888	51.30417	252.2038
0	0	0	0	75.49449	82.90873	29.60278	56.54308	240.7039
0	0	0	0	79.55265	87.48611	32.16667	60.06583	241.0961
0	0	0	0	80.29599	110.6334	42.43001	81.0135	284.5895

For peer review only

0	0	0	0	78.64847	119.8261	140.0624	27.38333	576.1822
0	0	0	0	82.1894	128.4481	130.5459	30.74778	586.1495
0	0	0	0	82.69824	128.815	133.083	31.26	625.9869
0	0	0	0	82.10941	110.3529	148.151	25.94666	730.512
0	4	0	0	17.88876	20	18.86974	18.68333	163.8595
0	0.333333	0	0	17.15098	16.33333	19.73252	15.91528	184.7871
0	0	0	0	16.2846	24	23.32252	16.99792	167.1548
0	0	0	0	23.18056	29.23333	22.21085	22.54361	129.0656
0.94	0	0	0	39.19611	26.95334	44.97552	30.79333	230.5834
4.166667	0	0	0	41.73444	18.49333	46.29097	30.5	226.1758
4.476667	0	0	0	40.46555	20.215	43.30921	29.63444	219.2504
5.330002	0	0	0	36.74522	30.05889	44.27475	31.05556	210.1568
0	0	0	0	34.51102	24.25426	37.06572	15.4825	173.8994
0	0	0	0	32.39547	23.38426	39.55315	17.89722	176.5733
0	0	0	0	32.6388	24.39426	35.64779	22.475	192.5596
0	0	0	0	31.05991	32.76093	27.44057	24.19	204.1078
0	0	0	0	36.73733	34.86795	40.13446	13.75	268.6337
0	0	0	0	46.11805	64.70335	43.47169	14.30778	278.9876
0	0	0	0	48.80305	58.54296	48.91597	14.09556	306.545
0	0	0	0	52.47501	65.07318	46.52565	15.05455	338.0895
0	0	0	0	29.25324	11.80067	1.870002	37.11666	123.8602
0	0	0	0	29.56124	23.39178	3.946662	40.16667	123.9024
0	0	0	0	31.9379	23.02622	5.25889	41.72556	119.7674
0	0	0	0	35.79846	22.19733	5.787783	42.08666	132.54
0	0	0	0	56.29411	52.51901	47.64245	22.66	300.6339
0	0	0	0	54.68134	47.68447	51.28486	27.50234	295.373
0	0	0	0	54.20805	47.52889	51.41112	26.2545	295.0769
0	0	0	0	51.43028	42.98167	51.77503	28.49889	287.8951
0	0	0	0	50.58734	46.57946	43.11937	47.23888	223.5873
0	0	0	0	49.85712	48.1089	39.44582	47.7745	253.6903
0	0	0	0	49.54643	55.48667	35.65861	43.65555	288.4518
0	0	0	0	50.53639	64.95834	32.4435	32.41893	295.8234
0	0	0	0	53.566	60.6112	26.12387	18.98666	419.1513
0	0	0	0	62.17211	68.98009	32.0538	35.86997	468.9815
0	0	0	0	63.00211	77.7072	35.45186	48.17648	562.528
0	0	0	0	65.86155	80.10606	33.86298	57.24691	653.9292
0	0	0	0	38.2239	57.43776	45.92511	20.1732	190.2412
0	0	0	0	56.69444	59.26553	55.99627	25.11645	210.4264
0	0	0	0	60.93847	61.3633	52.47672	28.28401	214.1312
0	0	0	0	66.70755	57.6433	50.19157	31.83401	234.8532
0	0	0	0	107.3164	59.62744	16.42112	39.12888	311.7714
0	0	0	0	88.416	57.46453	7.432227	30.24055	203.392
0	0	0	0	92.18314	67.73286	4.383337	27.68889	175.5543
0	0	0	0	107.5513	74.82486	4.648337	25.79277	195.649
0	0	0	0	38.5397	27.20496	37.45947	4.083334	138.4058
0	0	0	0	43.50359	29.06875	38.33779	3.5	139.4047
0	0	0	0	44.04637	33.64517	37.56063	3	149.709
0	0	0	0	44.89783	38.41031	37.26676	4.272728	158.0006
0	0	0	0	32.215	24.22722	43.50554	19.56944	192.1644
0	0	0	0	31.97222	25.19445	45.81165	20.15	213.9322

0	0	0	0	32.19778	35.15667	46.59054	18.81666	231.1906
0	0	0	0	34.29836	40.42889	44.59726	21.85444	247.6556
0	0	0	0	95.35387	166.311	99.13665	45.86611	578.8763
0	0	0	0	100.1822	179.4306	101.691	41.92386	567.0064
0	0	0	0	92.15617	165.8534	107.1348	43.30888	538.0212
0	0	0	0	91.12332	175.6705	107.7301	42.44833	533.9163
0	0	0	0	49.45237	19.68334	37.38983	13.14	203.3513
0	0	0	0	50.99265	17.5089	38.22983	14.45667	207.4832
0	0	0	0	50.87819	13.41111	37.87983	14.60889	226.6148
0	0	0	0	52.5554	16.05556	38.194	13.32334	231.0774
0	0	0	0	49.46288	18.92445	22.97577	13.81667	141.8346
0	0	0	0	52.51774	23.40889	25.87889	14.08333	148.2228
0	0	0	0	49.44243	26.83222	26.16057	15.46667	155.3616
0	0	0	0	51.70776	37.20463	31.47228	27.87222	171.0913
0	0	0	0	51.31702	15.19	35.90055	16.66667	141.2696
0	0	0	0	53.29291	17.87	37.40222	18.71622	142.6235
0	0	0	0	50.9662	21.67333	33.94722	16.25	147.9554
0	0	0	0	51.25462	19.05333	30.65111	16.61667	164.4831
0	0	0	0	51.81922	54.07778	32.84687	30.06307	273.4819
0	0	0	0	58.34066	90.04666	55.95811	30.11109	253.124
0	0	0	0	77.06284	118.6006	70.95914	37.1219	234.6111
0	0	0	0	84.5909	124.6875	70.16869	39.17223	265.4347
0	0	0	0	40.00882	87.25166	32.87474	22.60167	133.0522
0	0	0	0	40.50648	84.19	36.39534	24.40638	143.1433
0	0	0	0	55.76034	81.67333	36.19033	23.85455	144.3435
3.009266	0	1.132223	0.342775	65.17209	85.15278	37.17289	24.16667	147.7528
0	0	0	0	65.66084	23.74111	34.34451	30.07556	178.787
0	0	0	0	73.26489	27.68111	33.17547	30.94167	178.7331
0	0	0	0	70.81628	30.18667	33.09547	28.91333	279.1931
0	0	0	0	68.11462	30.14166	32.00436	27.81666	320.7412
0	0	0	0	58.17667	116.4144	42.54722	25.47565	288.7468
0	0	0	0	56.8589	112.3833	43.46001	23.50394	292.6957
0	0	0	0	67.05557	89.49001	62.72889	27.74889	294.7856
0	0	0	0	51.12122	71.82425	66.53211	34.12875	300.9852
0	0	0	0	54.37112	67.8425	79.32753	27.64868	221.7139
0	0	0.416667	0	55.81612	68.74165	81.84868	24.76667	221.5181
0	0	0.916667	0	117.5105	186.2777	99.3042	38.76036	388.572
0.25	0	0	0	141.8524	227.6717	108.682	52.00333	542.4265
0	5.06666	0	0	74.29829	109.9266	94.90105	30.99	495.1231
0	4.754446	0	0	75.39365	116.1282	97.1283	30.9628	518.5047
0	4.96	0	0	85.51695	117.7126	109.6833	33.36532	528.2781
3.744446	0.826667	0	0	84.99211	111.1995	126.9911	31.81352	526.4653
0	0	0	0	40.503	87.54601	46.25772	20.50666	259.4866
0	0	0	0	41.49833	99.33359	47.3087	21.80666	290.8037
0	0	0	0	42.63445	107.2517	37.61092	25.47603	319.3368
0	0	0	0	41.93611	102.5338	38.39092	28.28333	353.1621
0	0	0	0	28.49528	27.63276	32.66016	32.75879	152.1676
0	0	0	0	24.3356	32.41767	28.29888	25.875	134.7426
0	0	0	0	25.13652	32.96297	27.73614	21.02222	121.6044
0	1.2	0	0	77.44119	88.82889	31.4367	36.91999	399.01

For peer review only

0	0.85	0	0	83.67255	92.38965	26.89033	34.94666	401.9153
0	0.655558	0	0	90.59421	93.25219	27.28589	42.438	392.1852
0.166668	0	0	0	99.76708	129.0782	61.45187	48.859	413.8465
0	0	0	0	57.7711	76.70227	71.95113	37.41081	286.5816
0	0	0	0	61.34611	79.5039	74.17669	39.5518	303.2213
0	0	0	0	61.64001	78.58501	72.44168	45.7205	336.7236
0	0	0	0	102.3065	152.5205	121.1682	113.1927	797.2858
0	0	0	0	64.42011	116.4434	94.6237	28.58333	323.7552
0	0	0	0	66.31735	115.488	98.44106	34.58333	354.3198
0	0	0	0	71.20673	123.0922	95.50632	37.75	352.625
0	0	0	0	71.5506	128.8436	95.9769	37.5	377.5809
0	0	0	0	34.82501	42.31167	28.24333	9.5	170.6472
0	0	0	0	39.32585	50.7889	22.01555	10.66667	171.105
0	0	0	0	49.05127	64.77001	20.98666	13.11333	173.3266
0	0	0	0	53.2296	67.51	26.17833	15.81333	200.7454
0	0	0	0	50.18654	28.22945	93.13781	22.47222	248.9795
0	0	0	0	53.75605	29.61795	120.3058	22.65	232.6494
18.84597	4.193328	0	0	56.19236	31.8932	113.5736	22.60667	231.8293
26.42542	0.797777	0	0	55.70951	37.12641	103.8204	21.97778	240.9869
0	0	0	0	53.91583	41.94833	41.85166	14.73	278.6867
0	0	0	0	54.19667	42.82333	38.405	14.53333	300.1592
0	0	0	0	61.17083	46.45167	40.49667	20.07	344.8375
0	0	0	0	60.66167	43.30084	37.56083	18.73333	289.8442
0	0	0	0	54.938	84.131	20.35674	38.34574	245.2401
0	0	0	0	77.70033	105.9955	36.62697	43.2296	305.3268
0	0	0	0	78.54869	102.5432	32.96238	34.91667	353.1421
0	0	0	0	70.28705	108.6848	32.71616	31.45222	394.3625
0	0	0	0	36.89854	43.76666	18.91329	16.74533	196.3081
0	0	0	0	38.23047	44.15444	19.72666	17.72867	201.8965
0	0	0	0	42.38279	45.73	24.69167	17.64867	207.4711
0	0	0	0	45.77288	54.31778	26.9199	18.332	213.8749
0	0	0	0	79.66457	141.4811	36.49463	17.08333	219.4292
0	0	0	0	80.91428	157.5459	36.96745	20.66667	230.3647
0	0	0	0	67.74854	163.8781	36.13646	21	224.7486
0	0	0	0	47.09501	24.41666	18.78911	20.07132	183.745
0	0	0	0	54.07815	44.07222	18.28911	11	193.792
0	0	0	0	59.21548	56.25666	17.66355	13.85222	210.4593
0	0	0	0	63.91538	63.3311	17.21577	15.21937	216.5414
0	0	0	0	86.40001	47.70223	188.3181	44.73333	470.6223
0	0	0	0	91.96257	50.31778	174.3059	38.21722	541.6065
0	0	0	0	97.92712	57.26334	194.7757	41.09222	572.4136
0	0	0	0	101.9983	77.31166	230.189	44.89528	671.6782
0	0	0	0	87.62674	93.57277	128.3549	24.89667	431.9348
0	0	0	0	124.9725	98.75921	119.0997	31.70278	460.3369
0	0	0	0	131.3848	105.9644	115.1525	37.9689	485.945
0	0	0	0	158.8243	101.6472	93.65778	45.54778	512.0301
0	0	0	0	94.53667	86.43466	1.8	23.08444	450.7956
0	0	0	0	99.26724	85.07242	1.8	26.96445	484.4262
0	0	0	0	82.39668	91.34184	2.751113	25.95334	505.693
0	0	0	0	87.68945	99.90083	2.960003	26.90667	540.034

For peer review only

0	0	0	0	211.6328	130.3064	176.3783	34.35778	616.8843
0	0	0	0	214.071	134.2782	178.8328	37.98889	649.0017
0	0	0	0	216.7739	133.9553	182.6095	39.71667	657.8593
0	0	0	0	223.0194	153.4412	189.1006	38.78778	710.5049
0	0	0	0	108.2322	134.0068	267.0096	15.78667	234.0873
0	0	0	0	117.5611	127.8845	259.1552	40.97222	236.8889
0	0	0	0	113.3383	125.7442	255.0214	54.05991	240.6553
0	0	0	0	114.9315	133.2232	243.2203	58.26667	276.6545
0	5.083334	0	0	50.4243	49.58333	53.94252	44.01666	157.8
0	5.083334	0	0	44.23909	48.82555	52.09927	49.25666	157.7161
0	5.416667	0	0	41.47111	51.33667	52.21907	54.78468	171.845
3.666667	2	0	0	46.00888	52.05156	50.69407	60.54222	184.7042
0	0	0	0	77.75794	115.8921	70.53842	80.30108	1337.404
0	0	0	0	90.46922	131.5352	69.13454	95.96063	1518.591
0	0	0	0	106.9565	140.9983	67.32454	98.22987	1634.233
0	0	0	0	113.2187	156.5858	73.13242	117.607	1792.255
0	4	0	0	71.39611	48.60667	58.38369	35.12167	347.7595
0	3.166667	0	0	77.21074	52.28001	60.99257	34.59454	322.1508
0	2	0	0	81.82031	49.43111	62.94312	32.90667	296.8677
0	0	0	0	90.81932	42.94333	62.21271	31.58806	309.7457
0	0	0	0	54.23882	20.44445	66.41214	19.41889	230.5894
0	0	0	0	53.7094	24.02556	62.15339	20.1	248.8199
0	0	0	0	48.69278	25.02555	53.93031	17.75222	229.6014
0	0	0	0	50.59111	28.85722	48.96066	19.13333	226.3304
0	0	0	0	70.85694	117.6727	136.0335	20.86233	434.0477
0	0	0	0	65.19438	126.2321	141.0371	20.07111	421.5065
0	0	0	0	70.83609	133.382	200.9739	39.75	463.3827
0	0	0	0	66.63459	150.8828	201.6882	47.24167	492.1205
0	0	0	0	62.00429	30.30223	46.25576	9.5	226.307
0	0	0	0	64.94536	33.91223	48.03721	10.08333	234.5871
0	0	0	0	68.46167	44.30754	51.67564	18.25	237.4853
0	0	0	0	79.32246	49.80149	58.12714	18	255.9291
0	0	0	0	153.8608	73.72522	41.05305	39.52889	729.9606
0	0	0	0	162.9737	78.55467	29.92515	37.03333	681.6342
0	0	0	0	168.0554	83.5286	28.19837	31.22111	645.7437
0	0	0	0	174.285	81.53263	21.1947	28.24666	626.223
0	0	0	0	61.48108	27.93	15.58167	27.05	113.5934
0	0	0.055556	0	60.73235	30.2	19.45889	23.17	132.0892
0	0	0	0	59.48791	29.46666	19.38667	21	132.1978
0	0	0	0	100.9553	36.95801	63.27776	48.44	263.9489
0	0	0	0	104.8645	43.85856	65.0472	55.15166	262.3594
0	0	0	0	116.9839	43.45245	65.75498	67.95557	271.3032
0	0	0	0	125.8278	45.02023	67.96053	71.8	303.8967
0	0	0	0	47.88732	25.40833	32.33262	11.75	166.7108
0	0	0	0	31.55692	24.77927	28.50756	11.08333	187.8609
0	0	0	0	31.63104	23.99834	26.055	10.66667	201.5004
0	0	0	0	32.8377	21.60334	25.07633	10.31667	203.8802
0	0	0	0	17.56334	79.5387	51.78281	21.26333	220.8798
0	0	0	0	17.17445	84.87463	56.07084	12.69	237.1627
0	0	0	0	15.68444	80.78655	57.2887	12.48556	239.4742

For peer review only

0	0	0	0	14.475	64.24442	56.06398	14.90111	260.2564
0	0	0	0	74.23582	130.0054	70.79266	31.13391	624.3656
0	0	0	0	75.68459	133.4302	67.02528	45.13992	581.2105
0	0	0	0	83.07035	123.7367	63.42167	40.70667	575.5424
0	0	0	0	92.08025	113.325	64.94555	41.88166	612.8752
0	0	0	0	43.54737	74.59378	23.46352	25.38333	335.0202
0	0	0	0	40.35007	83.48676	37.53684	41.40223	328.834
0	0	0	0	38.68736	95.45954	38.43323	42.84	330.3475
0	0	0	0	37.3728	104.7742	38.93213	48.77556	356.9635
0	0	0	0	82.04607	54.64626	32.03389	16.10333	650.8148
0	0	0	0	104.2837	57.42696	33.29054	16.34555	598.3036
0	0	0	0	81.11107	66.09831	34.44786	18.96666	601.446
0	0	0	0	81.09008	71.77871	37.11111	19.43333	626.9033
0	0	0	0	122.0912	75.06612	56.29187	86.88776	358.0542
0	0	0	0	128.2488	79.96236	57.5733	98.62777	368.8251
0	0	0	0	128.2466	80.84354	56.87842	112.107	358.3431
0	0	0	0	122.3556	72.0074	60.28404	117.0882	388.1248
0	0	0	0	36.13667	35.46121	23.8246	55.28614	314.2331
0	0	0	0	39.72278	55.81288	26.955	61.79166	302.5591
0	0	0	0	40.41723	69.71288	26.05611	61.50361	306.6953
0	0	0	0	41.85416	75.74732	27.12667	71.69778	324.1287
0	0	0	0	133.7902	39.82888	70.79254	20.74167	459.3482
0	0	0	0	144.0925	42.08443	74.94441	20.08333	462.3694
0	0	0	0	155.0705	41.55638	104.703	21.58445	474.8806
0	0	0	0	161.3545	40.66611	157.4725	20.93333	517.4901
0	0	0	0	60.49666	23.87453	37.18666	21.33333	321.3383
0	0	0	0	57.68999	24.75786	31.97666	21.32222	328.3432
0	0	0	0	53.19184	30.51786	30.57407	27.15555	316.2067
0	0	0	0	52.87515	50.86	40.74062	17.75225	224.6135
0	0	0	0	59.21701	49.02	41.31603	20.05	215.6378
0	0	0	0	65.92071	48.36779	40.13789	22.38333	208.8394
0	0	0	0	66.23227	51.96612	41.58357	25.08667	244.2628
0	0	0	0	26.54834	28.09	20.01333	5.920044	192.8578
0	0	0	0	26.07945	49.5089	22.76723	7.68468	175.1189
0	0	0	0	25.67779	51.17335	22.65334	4.51351	171.6668
0	0	0	0	27.58334	49.24779	23.75112	5.167792	176.9101
0	0	0	0	102.5906	135.6356	83.29779	10.91667	430.5028
0	0	0	0	110.6088	117.9997	79.88557	14	475.0791
0	0	0	0	111.6945	121.1847	78.23847	17.66667	530.4459
0	0	0	0	123.6245	118.8008	81.15875	19.63333	590.6913
0	0	0	0	47.71052	30.60453	84.97632	32.9727	433.5767
0	0	0	0	49.34951	33.06009	79.28371	43.58777	462.7661
0	0	0	0	78.04148	117.0023	61.108	33.81837	770.9537
0	0	0	0	100.5637	131.5007	61.63465	46.96783	874.1386
0	0	0	0	112.7564	135.4162	70.94402	63.23417	905.7645
0	0	0	0	148.6843	135.9877	87.51125	71.75213	1088.211
0	0	0	0	68.89877	81.70937	31.48323	23.97556	233.6036
0	0	0	0	65.0859	89.97937	30.0839	26.50667	236.1515
0	0	0	0	62.35565	85.81315	28.62612	33.28334	261.7064
0	0	0	0	66.37628	88.75314	31.62112	42.63445	289.703

For peer review only

0	0	0	0	27.38093	63.98166	28.51045	18.5	262.726
0	0	0	0	32.30196	67.76112	27.95125	21.26667	274.1227
0	0	0	0	33.77626	69.21111	27.45903	28.71667	282.5109
0	0	0	0	38.74737	72.42944	37.5109	30.63333	281.9885
0	0	0	0	43.58463	19.21334	73.04549	18.58333	291.9824
0	0	0	0	89.29504	35.04	78.52247	18.38445	312.1014
0	0	0	0	44.65215	53.63556	54.98709	13.46667	295.4364
0	0	0	0	51.10366	59.64778	56.01442	12.93356	316.9586
0	0	0	0	92.69943	84.88922	47.79066	19.63	271.3798
0	0	0	0	105.31	93.86344	42.68222	21.63	235.1373
0	0	0	0	100.9422	93.35622	39.91444	23.09667	254.374
0.783333	0	0	0	101.3522	98.07603	37.66722	25.48111	283.1674
0	0	0	0	32.90556	61.47889	25.34727	27.41111	161.7324
0	0	0	0	31.66611	64.43	23.23004	24.72333	163.2991
0	0	0	0	38.16	66.68723	20.99111	23.65222	159.6417
0	0	0	0	38.30223	65.29111	20.44666	25.64444	165.0519
0	0	0	0	64.9407	24.24666	33.95823	49.41999	243.3016
0	0	0	0	65.13355	27.74888	35.06715	51.49555	251.2633
0	0.083333	0	0	68.19809	31.39666	35.01044	47.40861	243.1713
0	0	0	0	68.36186	30.504	33.93175	41.52066	223.3669
0	0	0	0	69.703	80.4801	7.825397	32.06167	383.1283
0	0	0	0	63.19535	101.3694	9.760405	37.155	358.4825
0	0	0	0	57.12413	105.515	9.212009	33.26	336.1181
0	0	0	0	98.0241	107.9378	27.01888	50.79972	424.6462
0	0	0	0	25.63889	30.03223	65.64965	29.88093	322.953
0	0	0	0	27.96944	31.72218	65.64697	35.08334	356.3214
0	0	0	0	29.39555	31.22602	61.31417	33.55334	306.3637
0.583333	0	0	0	35.63944	30.49435	58.2311	29.05444	202.2091
0	0	0	0	58.04265	31.77192	60.92794	44.11	178.4031
0	0	0	0	62	44.26136	50.55085	45.24	183.3573
0	0	0	0	64.13384	60.81637	47.26196	47.98222	190.4066
0	0	0	0	73.40774	68.37192	47.18649	56.8554	210.4638
0	0	0	0	50.59	25.02622	19.28778	12.81667	169.7653
0	0	0	0	59.40334	28.35657	22.47	13.48333	160.9198
0	0	0	0	52.80722	34.43935	21.66387	23.9	188.9258
0	0	0	0	59.46445	35.5788	21.05609	30.16667	221.2612
0	0	0	0	113.1878	168.8561	20.54014	57.61167	228.3819
0	0	0	0	113.5372	166.6768	16.9989	61.95208	241.4
0	0	0	0	106.9376	175.6793	16.88112	65.03875	250.6699
0	0	0	0	114.4778	169.6279	16.59945	77.49344	392.8751
0	0	0	0	28.43154	52.92	14.45914	37.776	260.7397
0	0	0	0	34.43	58.95444	11.70111	27.81444	286.6073
0	0	0	0	42.90278	52.64444	8.951112	22.34333	299.9434
0	0	0	0	47.43721	52.91556	22.81778	31.85444	245.4657
0	0	0	0	64.74434	99.06287	98.30178	114.4309	698.9812
0	0	0	0	69.08934	99.94341	93.95507	119.3294	738.3878
0	0	0	0	68.15157	100.7874	93.77927	113.3553	750.1065
0	0	0	0	70.20001	104.1693	89.95224	118.9366	768.9652
0	0	0	0	24.55664	51.585	12.8489	28.56847	173.2787
0	0	0	0	24.45331	59.50333	13.46612	34.76081	176.048

0	0	0	0	28.23887	63.135	13.86333	45.35	169.7807
0	0	0	0	32.95999	70.41212	14.72243	49.03394	160.9477
0	0	0	0	44.63889	60.925	9.75444	77.34751	242.2275
0	0	0	0	38.132	57.37555	4.949998	83.7592	288.9543
0	0	0	0	35.41421	58.49166	5.675556	82.795	326.9298
0	0	0	0	34.39429	58.86056	5.746666	81.23666	405.3261
0	0	0	0	22.14894	17.33994	20.98546	16.79605	87.68097
0	0	0	0	30.97222	45.12555	0	30.67112	127.4576
0	0	0	0	36.06112	44.05555	0	30.38556	148.5164
0	0	0	0	38.58501	23.04111	0	32.71667	171.0559
0	0	0	0	39.14111	24.13999	0	38.415	170.4487
0	0	0	0	43.38124	73.49	22.48	9.333333	206.0711
0	0	0	0	36.37124	75.27334	19.86667	11.08333	192.1942
0	0	0	0	30.55791	90.00057	19.54333	11.5	193.2944
0	0	0	0	30.32736	97.64667	21.58889	11.41667	193.9461
0	6.433333	0	0	135.7573	72.84665	43.25922	28.49657	599.543
0	5.872727	0	0	145.8153	63.44969	40.73866	24.49586	568.4641
0	0	0	0	59.68393	22.73333	93.95258	28.5876	169.9637
0	0	0	0	65.9064	27.89666	91.97578	34.20608	189.2403
0	0	0	0	77.27782	27.70832	91.27354	36.15458	190.2944
0	0	0	0	82.46806	28.40777	93.04444	35.26478	188.845
0	0	0	0	176.8916	201.4064	149.3789	25.76084	891.8038
0	0	0	0	192.0592	204.189	153.8784	25.01576	909.8412
0	0	0	0	197.6977	222.36	151.2298	25.04955	942.7186
0	0	0	0	209.7822	276.9822	154.6512	23.8232	987.3142
0	0	0	0	50.69603	35.91463	32.38379	25.91667	341.435
0	0	0	0	48.40103	37.08296	28.45851	26.06667	335.7356
0	0	0	0	43.84937	40.09525	29.915	31.26556	343.0459
0	0	0	0	72.11287	52.88275	48.59342	33.54445	418.3512
0	0	0	0	94.45332	54.38651	97.93691	17.15	892.9877
0	0.416667	0	0	112.1407	65.12006	108.806	20.14814	975.0002
0	5.833334	0	0	254.4089	130.8165	146.3488	41.21515	1554.352
0	2	0	0	283.3084	133.5867	148.0376	62.22667	1592.959
5	7	0	0	23.27333	68.02884	0	53.185	357.9939
4.5	6.5	0	0	24.02444	87.44881	2.583333	51.09889	383.46
0.333333	1.166667	0	0	29.95277	104.3266	3.333333	49.18056	609.1406
0	0.166667	0	0.833333	35.16945	101.7577	2.75	48.36389	678.2434
0	0	0	0	135.4853	211.8346	132.3483	10.66667	469.1866
0	0	0	0	138.0075	209.214	111.2121	9.166667	490.279
0	0	0	0	151.9796	215.714	102.9889	9.416667	493.861
0	0	0	0	164.5873	236.9351	103.6514	11.5	512.1155
0	0	0	0	78.83677	90.88871	114.8486	21.55357	354.4417
0	0	0	0	72.52361	92.51135	115.2511	24.08611	364.3175
0	0	0	0	66.40879	95.52967	120.6575	29.28333	362.6629
0	0	0	0	78.23502	105.068	121.2319	42.46555	398.0476
0	0	0	0	105.3836	53.8365	35.69445	70.42612	694.3902
0	0	0	0	109.7016	59.47705	33.59111	77.67724	715.8474
0	0	0	0	111.9838	71.43205	33.27222	78.60612	763.4843
0	0	0	0	112.6287	86.42964	29.86111	69.47056	844.5725
0	0	0	0	140.8856	66.66277	16.40223	20.18555	503.2448

For peer review only

For peer review only - http://bmjopen.bmj.com/site/about/guidelines.xhtml

0	0	0	0	147.9434	77.13438	37.82112	19.69666	466.6944
0	0	0	0	206.196	101.8716	57.16768	31.50777	578.7451
0	0	0	0	225.1388	109.5308	62.04934	31.13888	632.2782
0	2.788878	0	0	95.20338	153.8732	113.3235	29.48258	608.6199
0	2.205547	0	0	96.37555	152.1697	110.9532	32.6185	630.6749
0	2.162226	0	0	105.1283	150.1629	108.7917	31.41778	663.8821
1.011117	0.711115	0	0	122.5129	151.9224	116.2628	38.97556	703.4739
0	0	0	0	29.43178	75.19212	26.98097	9.5	323.6409
0	0	0	0	33.33956	77.03977	30.60541	12.83333	317.7664
0	0	0	0	28.91789	55.72221	30.36009	18.94333	330.0499
0	0	0	0	29.81845	36.67333	31.59393	32.84777	332.0933
0	0	0	0	24.4612	16.92627	21.96722	24.14258	147.166
0	0	0	0	25.87333	17.72627	26.44555	28.15485	156.4331
0	0	0	0	30.64444	19.71182	27.77	28.07152	172.1812
0	0	0	0	38.27444	21.77516	29.47445	28.97152	184.9547
0	0.988332	0	0	174.1525	92.06611	108.9645	23.76127	404.5273
0	0.276111	0	0	177.236	98.69611	94.1684	24.54205	447.2958
0	0	0	0	172.3388	90.22889	77.31728	19.58778	440.1973
0	0	0	0	172.2293	100.9235	66.62172	24.27833	451.1906
0	0	0	0	44.16916	61.03699	25.03786	18.55889	294.4796
0	0	0	0	38.53373	63.53111	28.20398	8.282223	331.7328
0	0	0	0	40.78272	79.51499	29.21897	8.333333	367.7333
0	0	0	0	83.59996	79.43276	24.14619	9.5	425.6219
0	0	0	0	121.0996	64.16222	120.4563	29.52114	333.1605
0	0	0	0	124.0851	62.53778	123.3687	30.44343	325.0263
0	0	0	0	125.34	63.24502	121.1783	35.89667	328.8986
0	0	0	0	139.0216	68.05113	127.4173	42.07779	351.4667
0	0	0	0	35.60077	46.43994	6.064422	75.00057	250.4994
0	0	0	0	36.79808	54.7816	6.936844	92.52891	247.7261
75.33529	0	12.28934	1.916667	34.67846	57.88715	8.020886	93.24724	242.3887
99.81863	0	10.2896	3.166667	53.31166	68.06058	7.007153	97.27424	226.5156
0	0	0	0	46.06406	107.0079	142.2153	22.63333	511.3056
0	0	0	0	42.11973	106.849	140.3252	23.11667	510.1187
0	0	0	0	44.78027	122.2642	146.5363	26.19279	549.2673
0	0	0	0	64.4444	128.7511	145.2246	35.8	582.0761
0	2.5	0	0	140.0677	37.79984	9.470008	31.99223	512.8486
0	2.5	0	0	139.235	43.69126	10.97334	33.83	501.2398
0	0	1	0	131.5146	41.86164	11.62223	34.15667	463.0237
0	0	1	0	129.0138	53.74281	11.22223	36.17	453.4724
0	0	0	0	57.58424	70.77056	42.53078	6.791667	195.6053
0	0	0	0	40.54389	72.63335	42.52244	5.416667	140.9799
0	0	0	0	37.7789	73.39279	42.87746	11.18446	117.2301
0	0	0	0	36.4939	77.63445	42.17746	10.41667	160.2119
0	0	0	0	85.27544	272.25	486.027	37.84096	706.4747
0	0	0	0	83.08328	280.9029	455.4683	44.53642	735.0672
0	0	0	0	106.1039	129.7204	29.86177	56.25556	529.1566
0	0	0	0	109.8978	142.4689	27.4711	68.22334	509.9545
0	0	0	0	114.5756	151.6443	27.32499	74.07667	494.2534
0	0	0	0	109.1759	155.9816	34.9711	75.91062	444.3479
0	0	0	0	131.7297	70.01177	92.94062	42.04111	740.8161

For peer review only

0	0	0	0	133.559	69.68056	99.11063	42.94334	765.6706	
0	0	0	0	140.2661	113.3233	97.31133	44.17334	777.0806	
0	0	0	0	156.8724	151.9378	86.30608	42.98779	960.0991	
0	0	0	0	108.1794	54.55599	75.37859	31.3	349.1184	
0	0	0	0	108.3778	57.21033	72.28408	31.45333	354.0865	
0	0	0	0	118.8367	53.32723	75.73769	32.71	366.0487	
0	0	0	0	130.1417	66.25223	76.32029	37.26667	413.3446	
0	0	0	0	128.8466	105.1954	49.51379	28.75	708.2703	
0	0	0	0	136.4625	108.5378	49.01162	29.08333	741.15	
0	0	0	0	134.1926	100.25	60.70321	30.95778	773.2701	
2.433333	0	0	0	144.0682	114.1783	51.24368	29.83333	835.4465	
0	0	0	0	83.44469	43.74505	52.61701	25.43919	418.7217	
0	0	0	0	83.83496	46.54671	49.60212	28.07397	414.5205	
0	0	0	0	84.15014	47.56893	51.07546	31.56222	447.4687	
0	0	0	0	78.06522	47.79615	52.75701	35.20111	488.3879	
0	0	0	0	29.81867	41.93941	47.51489	87.57343	269.5069	
0	0	0	0	34.67889	42.71275	49.25205	93.65169	280.7192	
0	0	0	0	36.18223	76.82052	49.66038	69.89757	299.1769	
0	0	0	0	38.66891	75.12162	48.07581	66.6659	311.59	
0	0	0.4	0	72.02383	34.22969	12.28293	8.5	819.5329	
0	0	0.266667	0	69.8994	46.08964	18.01738	9.666667	856.2064	
0	0	0	0	52.8249	58.68551	18.3496	9.583333	892.7742	
0	0	0	0	56.39217	74.89572	18.98571	11.95	965.0202	
0	0	0	0	45.76331	46.35481	47.75802	26.34874	323.3939	
0	0	0	0	51.41327	48.55209	36.73729	35.83695	309.8821	
0	1.25	0	0	33.90937	74.05547	37.57762	32.14786	314.4261	
0	0	0	0	35.16797	93.46311	41.04103	33.14724	324.0222	
0	0	0	0	77.20094	63.54973	56.93002	29.86444	241.536	
0	0	0	0	83.71034	66.91297	54.80692	33.18667	246.3909	
0	0	0	0	91.45485	68.72952	55.19117	31.86166	244.428	
0	0	0	0	98.73805	75.37976	54.21671	32.32777	267.3149	
0	0	0.333333	0	60.50175	49.36943	48.66372	18.59667	199.7101	
0	0	0.833333	0	58.55793	46.99059	53.86372	26.96333	209.6276	
0	0	1	0	66.71424	47.3989	59.74264	32.26667	224.245	
0	0	0.833333	0	78.37492	44.13556	61.20192	40.37553	246.4898	
0	0	0	0	50.06091	107.7623	42.29934	35.93944	377.3038	
0	0	0	0	61.0054	123.0562	48.85545	34.35111	379.5182	
0	0	0	0	75.11981	118.7041	68.12334	32.4	407.2043	
0	0	0	0	78.90259	122.5033	70.79778	33.57533	415.5682	
0	0	0	0	140.2929	120.3109	27.63149	204.8914	649.2327	
0	0	0	0	141.9238	125.4567	23.07422	219.3089	638.212	
0	0	0	0	146.1095	132.2537	20.15467	82.2537	685.788	
0	0	0	0	149.711	72.91999	23.24277	85.73732	676.8751	
0	0	0	0	73.73018	46.10593	26.43187	21.58333	392.1289	
0	0	0	0	79.73573	43.40481	30.42543	24.05	423.8537	
0	0	0	0	85.46373	40.91097	30.24271	23.50338	416.7659	
0	0	0	0	85.10652	39.10955	28.20878	24.95811	416.8288	
0	0	0	0	109.2278	67.60169	137.5302	29.85352	346.0234	
0	0	0	0	116.4109	73.79401	143.2354	32.49398	363.0845	
0	0	0	0	114.1226	79.47111	145.4559	39.88565	361.9775	

For peer review only

0	0	0	0	121.6677	92.77332	143.4425	42.17676	387.3694
0	0	2.083333	0	48.83046	107.6289	57.76636	26.7534	276.8562
0	0	0	0	54.4499	101.4556	58.77196	21.56001	269.2816
0	0	0	0	55.98323	100.0672	63.81529	18.90112	263.3878
0	0	0	0	52.46713	101.2383	60.4762	20.62558	268.6151
0	57.01	0	0	84.99079	38.84805	44.26734	38.94815	313.5533
0	56.77667	0	0	91.91178	41.74278	39.29957	44.36112	324.0007
0	55.88333	0	0	87.47179	43.63555	31.51736	48.52519	344.4303
4.361111	50.65555	0	0	94.41277	50.78666	22.5566	50.13696	414.9766
0	0	0	0	90.56987	91.35019	62.10039	33.04506	615.0898
0	0	0	0	70.23568	98.42818	63.67228	35.63666	640.0029
0	0	0	0	70.64362	103.3906	64.62336	39.94167	678.0995
0	0	0	0	63.30651	88.73285	62.92613	46.65778	718.0275
0	0	0	0	72.70661	204.9677	76.55474	20.40222	396.6125
0	0	0	0	75.85973	199.6502	80.48068	14.6	418.0368
0	0	0	0	79.82583	178.2426	79.5719	14.2527	414.682
0	0	0	0	77.01495	193.1105	86.45477	15.13333	449.207
0	0	0	0	110.5041	53.19223	21.10814	37.72889	479.2269
0	0	0	0	107.8554	56.33445	14.81547	42.38333	506.3777
0	0	0	0	96.7558	64.85889	14.94379	46.11056	497.1837
0	0	0	0	95.57324	53.96889	15.15546	45.71889	474.7132
0	0	0	0	97.83743	26.73	58.40102	26.2232	276.8402
0	0	0	0	103.063	25.13167	62.35182	29.03889	272.9884
0	0	0	0	105.0776	34.52833	59.4298	32.19672	273.5542
0	0	0	0	108.1164	40.48867	53.51138	34.82946	287.6334
0	0	0	0	147.4866	55.85167	104.4506	65.78889	478.1207
0	0	0	0	150.4254	51.42001	114.8381	69.21939	546.3884
0	0	0	0	120.3337	65.05368	158.2945	63.13409	504.503
1.25	0	0	0	127.5263	52.28667	208.0172	42.90333	449.7567
0	0	0	0	75.23055	73.93667	62.20331	32.91667	276.0133
0	0	0	0	75.28611	70.47424	64.7222	34.68333	292.1239
0	0	0	0	89.30637	73.39346	63.73608	35.98333	303.2318
0	0	0	0	93.47165	76.43402	64.04331	40.3978	321.1809
0	0	0	0	67.03389	40.68852	114.3328	44.00444	771.153
0	0	0	0	73.97334	40.55076	115.6533	48.09667	803.5501
0	0	0	0	74.14111	38.31024	118.3729	54.54889	857.3868
0	0	0	0	81.06611	38.14028	119.0093	57	943.2418
0	0	0	0	101.5421	64.07777	87.64417	24.97334	419.6617
0	0	0	0	102.8277	64.05334	90.71677	29.31901	424.1343
0	0	0	0	105.4229	64.90111	93.67888	34.19018	419.7332
0	0	0	0	109.3394	70.94333	96.2767	29.43473	429.3087

For peer review only

v82	v83	v84	_unknown	_supportto	v87	_estatesma	support_sc	_magers
0	3.618889	0		106.2259		0	106.2259	42.78347
0	4.593337	0		107.73		0	107.73	47.7686
0	4.92667	0		82.17819		0		
0	4.810003	0	2.833333	54.15348	0			
140.6401	57.71912	23.3		330.6587		0	330.6587	226.2585
200.0821	53.39023	24.15		294.6275		0		
280.883	58.97289	30.05667	8.333333	311.6854	0			
456.2948	29.47521	33.32		75.70437		0		
452.9909	28.66409	38.05903	17.5106	79.02659	0			
114.4889	5.3	35.94111		55.46113		0	55.46113	35.20444
111.1578	4.95	35.50213		53.83447		0	53.83447	36.97778
118.4786	4.512222	36.60778		51.35003		0		
116.5669	4.143332	35.22952	41.80222	52.53003	0			
7.466667	46.506	55.71112		290.122		0	290.122	216.5872
8.3	45.64489	54.05222		271.8439		0	271.8439	206.7183
9.633333	40.346	63.06667		252.4943		0		
10.71667	39.52766	66.43333	47.30889	254.8233	0			
24.68712	21.43201	42.88792		60.16996		0	60.16996	123.0487
25.61601	20.17516	54.5468		55.62418		0	55.62418	120.9732
33.36379	15.38738	52.75013		47.61474		0		
35.48489	13.94493	49.4168	5.033333	46.04147	0			
3.166667	5.333334	5		68.78329		0	68.78329	46.07667
3.75	6	4.416667		68.23908		0	68.23908	53.47667
22.38821	9.916667	4.416667		74.00348		0		
23.82838	11.56667	5.583334	3.094443	83.88902	0			
108.1046	4.55	9.485556		23.25977		0	23.25977	2
108.5124	3.774446	9.8		20.752		0	20.752	1.583333
103.8093	4.266667	9.452223		20.93977		0		
98.56723	3.86667	11.23636	7.072727	20.22715	0			
79.18576	3.962223	29.12778		33.07655		0	33.07655	47.28889
93.51469	11.49	31.55		32.31889		0	32.31889	45.38112
115.81	15.2	37.91556		32.10001		0		
111.167	15.48333	42.41333	0.666667	32.9789	0			
316.1325	66.81332	68.77333		120.9807		0.666667	120.9807	50.14196
290.3226	67.65611	66.04		133.2622		0.966667	133.2622	52.92723
287.2604	68.34388	65.24333		138.1317		0.3		
324.4761	73.03999	66.73556	3.276667	140.1899	0			
267.0564	36.68444	68.89614		96.87595		3.638892	96.87595	117.4494
80.48503	34.89	228.7579		26.78962		3.055561	26.78962	89.23778
57.57478	37.67223	245.028		7.25		3.222223		
56.83555	44.07112	266.5658	2.416667	7.833334	2.45			
374.4771	41.05223	38.45833		39.12221		0	39.12221	43.19798
364.7826	41.36556	43.09		37.38332		0	37.38332	48.08081
367.1518	40.51223	42.64		35.30722		0		
368.9518	37.83222	42.19666	12.2	34.91389	0			
53.80555	12.07222	37.85		19.18054		0	19.18054	101.7671
53.59666	12.81389	35.84		19.24498		0	19.24498	100.9229
53.56556	14.16666	33.38		19.97188		0		
100.0361	21.57554	43.60222	4.178886	37.52512	0			

161.2667	40.77533	36.65		167.8193		0	167.8193	63.17556
168.3633	44.47533	34.9		138.6188		0	138.6188	59.64889
183.8544	59.63604	39.63111		105.4244		0		
209.0089	72.65009	32.51667	57.78444	120.2622	0			
38.29001	3.416667	0		42.65603		0	42.65603	48.75666
35.85556	4.916667	0.333333		40.99947		0	40.99947	46.59333
13.01889	5	2		38.95		0		
4.083334	5.833334	4	21.47838	36.68945	0			
203.6665	33.23001	92.29722		60.15056		0	60.15056	53.07278
193.8077	34.24889	89.82999		37.19556		0	37.19556	53.37667
207.4013	36.20167	74.06889		41.01113		0		
213.6854	30.86334	48.23	2	38.87447	0			
28.58445	18.48773	20.08333		30.03859		0	30.03859	56.74556
28.105	17.6044	18.5		38.02194		0	38.02194	56.17612
26.34334	19.38866	13.16667		38.18583		0		
21.89334	20.37	13.83333	1.666667	37.55693	0			
186.0451	22.43666	23.83333		19.556		0	19.556	44.68667
182.2365	26.21666	25.33333		21.13196		0	21.13196	52.63
271.7033	27.70333	35.78333		20.25167		0		
295.3127	29.33333	37.05455	30.35031	25.21909	0			
97.00484	3.883333	17.25222		41.07704		0.333333	41.07704	26.78889
92.4511	4.250001	15.55		40.21544		1	40.21544	41.24445
91.35245	5.169445	17.13333		41.65033		1		
103.858	5.73333	18.33333	5.375556	45.80166	1			
237.8167	11.55547	0.666667		61.95324		0	61.95324	63.78525
234.1065	17.46703	0		57.99775		0	57.99775	58.31925
245.5202	17.04802	0		49.92108		0		
250.4036	16.86622	0	1.76333	41.04107	0			
421.8029	35.77668	43.44		118.247		0	118.247	40.42333
415.635	33.86222	40.72333		122.523		0	122.523	46.78889
418.18	30.56334	42.94444		123.5102		0		
421.8517	30.67334	46.12333	0	121.2545	0			
244.5857	10.57335	0		145.2459		0	145.2459	59.81833
250.299	10.67001	0		147.9028		0	147.9028	86.79778
246.3201	10.05668	0		151.6385		0		
247.5334	7.584461	0	2.9375	149.2575	0			
253.28	29.2164	44.55		81.33391		0	81.33391	37.54389
261.0611	26.87352	45.9		82.28671		0	82.28671	45.95889
256.7328	28.42104	44.8		91.00939		0		
247.8117	30.94881	54.78888	0.166667	93.59396	0			
91.45984	13.23333	17.01667		33.55444		0	33.55444	43.42111
48.51019	12.11889	15.48334		32.84722		0	32.84722	40.85333
47.49514	10.76	16.25		34.32333		0		
49.4907	10.47555	16.5	0	34.00612	0			
120.6777	10.58556	25.26667		40.80601		0	40.80601	40.56334
120.5061	11.85556	25.31667		37.8111		0	37.8111	46.29001
116.7361	12.05	24.48333		38.45888		0		
116.1271	14.27031	32.41213	0	39.59151	0			
72.34055	24.97223	15.75		50.92498		0	50.92498	62.32333
69.42222	28.59111	14.5		56.69221		0	56.69221	72.81667

For peer review only

75.02111	30.78999	16.5		58.65831		0		
88.36937	34.1611	17.92222	13.29056	63.35776	0			
263.9116	38.309	126.5509		142.6588		1	142.6588	88.16202
434.5484	48.71178	121.7058		154.5096		0.96	154.5096	100.1732
458.3243	56.724	118.9108		151.2504		1.376667		
483.6372	72.92844	120.7571	3.383333	147.5031	1.96			
128.5112	18.99	22.5		39.48659		0	39.48659	42.31475
131.2074	20.52333	24.36667		39.42437		0	39.42437	42.8192
124.703	21.99555	23.43333		39.77214		0		
127.8022	22.45666	24.03333	4.569997	38.79214	0			
133.5468	6.168801	26.06		52.85476		0	52.85476	43.08555
127.4645	8.15956	28.06667		93.47112		0	93.47112	43.44111
156.0826	10.31668	34.45		99.84167		0		
199.8153	12.64889	40.08556	3.663619	102.9781	0			
254.7089	9.827783	23.16667		27.984		0	27.984	44.72999
258.7287	12.67778	24.16667		25.34534		0	25.34534	45.90777
262.7529	11.4	23.5		25.81534		0		
275.0679	15.65	21.35	0.416667	26.41256	0			
296.4229	37.63989	32.07333		67.91175		0	67.91175	96.73026
280.7529	40.64889	31.19222		82.90306		0	82.90306	103.2458
273.6151	38.77778	30.39333		84.02593		0		
269.3	42.59222	31.47333	5.30205	86.80781	0			
59.1548	13.52578	26.69444		77.16013		0	77.16013	52.41151
60.01408	12.06956	23.78333		77.3709		0.5	77.3709	49.61678
61.25252	13.37266	25.7		76.2261		1		
63.62809	14.91155	26.95	0.566667	70.36333	1.166667			
168.9709	17.45023	19.16111		71.43889		0	71.43889	55.82726
175.3256	20.9736	20.25		71.5249		0	71.5249	63.35233
179.6999	20.62194	21.25		74.50856		0		
186.2589	18.98305	23.08333	6.89	73.49816	0			
256.5256	29.55722	60.83222		37.04861		0	37.04861	46.34444
286.8292	33.51	43.72333		40.4		0	40.4	44.75
284.7581	31.41023	42.13333		41.71667		0		
268.1823	48.83531	33.49697	1.054545	41.1128	0			
258.842	44.74612	22.13333		9.690003		0	9.690003	74.20667
244.5568	45.02778	22.05		112.2649		0	112.2649	83.26555
270.1984	60.64778	53.50667		140.2213		0		
279.1149	61.84889	64.72556	19.11555	154.9399	0			
380.5833	63.74942	45.08333		193.4565		0	193.4565	105.4991
458.131	67.47288	44.45		189.3164		0	189.3164	103.8631
484.8083	60.50224	45.23333		205.3337		0		
615.188	69.17494	53.45	34.29093	216.4115	0			
202.3559	28.73803	54.50501		72.65556		0	72.65556	43.10722
199.4211	29.4789	53.38556		69.54584		0	69.54584	54.65722
197.6522	32.30834	52.04278		69.07782		0		
196.3414	28.68667	53.29725	5.36001	59.79002	0			
224.1399	37.0982	32.80222		16.6378		0	16.6378	34.12833
217.924	21.75632	31.55		16.22668		0	16.22668	31.90333
212.3955	20.27965	31.24445		15.25484		0		
66.73446	17.08222	39.825		90.01749		0	90.01749	59.93858

For peer review only

63.55335	21.27333	39.41667		95.04529		0	95.04529	60.26525
67.71223	24.14667	44.77945		93.89417		0		
80.00779	36.89444	42.68333	9.624443	98.77194	0			
325.9483	22.81444	47.58333		30.46111		0	30.46111	85.20667
329.3772	18.68999	45.89702		30.14889		0	30.14889	82.87546
336.1993	24.07333	52.26278		28.81722		0		
206.6744	30.13555	265.0921	38.05166	176.383	0			
8	7.013593	0		118.9658		0	118.9658	79.08556
11.53556	8.178041	0		132.0898		0	132.0898	76.88111
12.66667	6.825819	0		128.5405		0		
13.16667	13.93983	0	9.966666	142.0775	0			
34.75001	11.01889	25.65		69.23881		0	69.23881	63.24556
32.02445	12.41111	23.08333		72.88436		0	72.88436	65.49556
93.09573	15.88666	22.31667		76.91213		0		
163.9761	21.97777	24.23187	8.278573	86.02103	0			
24.25695	20.37748	132.2841		99.1936		0	99.1936	42.02252
29.82444	13.9588	132.4222		103.289		0	103.289	34.8297
27.89667	15.6582	135.0553		99.53849		0		
20.25333	21.14334	139.615	2.36333	95.46218	0			
220.3658	3.945	37.56667		77.4975		0	77.4975	37.6825
227.5242	2.618333	37.3525		79.21917		0	79.21917	40.675
241.9717	2.18	34.805		42.38667		0		
63.66083	2.03	5.933333	21.17833	12.60583	0			
37.49276	16.326	40.38435		70.12809		0	70.12809	76.69978
102.5622	28.73089	45.18104		82.36291		0	82.36291	106.8445
94.38667	41.292	43.44662		92.83068		0		
122.8833	34.88733	46.6033	4.784443	99.27456	0			
234.5271	13.18	29.5		20.05554		0	20.05554	29.95667
241.8023	10.94334	30.25		19.66332		0.083333	19.66332	32.96444
247.3705	11.00667	28.33333		16.16166		0		
252.6265	10.76334	28.41667	0.166667	15.97999	0			
0	24.81666	46.17444		9.915869		0	9.915869	43.02334
15.08684	33.25222	43.37222		9.995508		0	9.995508	43.64
29.98767	38.46666	42.65334		10.12773		0		
245.5129	47.7605	31.67778		18.27444		0	18.27444	48.36834
228.6345	50.45383	34.2		20.49999		0	20.49999	66.09834
224.8568	46.55604	33.69167		20.74666		0		
228.3655	46.50604	34.61666	0	25.28777	0			
94.97666	132.153	136.9611		422.652		1.8	422.652	189.6089
92.90889	141.6443	140.9044		332.9929		2.166667	332.9929	193.1755
94.08334	140.5402	146.1856		307.2807		2.666667		
98.63555	152.7037	147.92	79.72222	330.0433	2.083333			
456.4544	74.92167	57.1		89.2356		0	89.2356	81.07332
484.0311	64.53001	52.78333		98.09627		0	98.09627	84.08166
469.4344	64.08001	49.92778		104.2615		0		
480.9888	60.105	57.11556	3.05	112.9256	0			
80.90888	45.07833	75.83334		163.0297		0	163.0297	299.0958
78.37888	45.31833	90.09333		160.9689		0	160.9689	317.7
89.61223	49.99444	95.02111		206.58		0		
72.07889	38.91444	107.9522	0.25	245.3651	0			

For peer review only

1240.564	60.16167	119.6656		272.547		0	272.547	169.6645
1269.94	59.83334	113.6856		273.5716		0	273.5716	180.7816
1290.103	58.7339	110.2417		282.5652		0		
1260.378	60.49223	115.4	27.55388	302.0982	0			
9.55	5.392223	0		213.4123		0	213.4123	137.6278
7.716667	4.9	0		207.2378		0	207.2378	121.3533
6.186667	4.656668	0		195.1217		0		
5.166667	7.785555	0	11.25278	194.6689	0			
353.5504	20.22338	4		95.78415		0	95.78415	71.15286
361.936	20.82014	3		88.96362		0	88.96362	79.29305
365.916	21.29604	1		91.99211		0		
368.1771	22.34335	0	1.233333	89.0527	0			
706.0303	230.6584	139.9167		49.58		0	49.58	271.4735
762.6826	227.1934	146.9356		44.40161		0	44.40161	311.6667
775.2473	235.9141	150.3689		41.63881		0		
801.2689	232.0256	159.1333	13.88333	48.30547	0			
38.02787	11.86667	7.666667		152.9536		0	152.9536	84.00166
41.50448	14.07778	7.833334		152.2095		0	152.2095	88.8
42.5856	13.87	8		156.848		0		
41.58382	14.745	8.75	0	156.7611	0			
23.80334	13.79	109.9601		21.33311		0	21.33311	35.53333
20.05666	13.18556	100.31		22.18088		0	22.18088	37.05555
51.30224	12.38333	66.98113		23.19199		0		
57.52446	13.07111	61.43335	2.15667	27.19533	0			
175.2511	36.72666	52.65		255.4296		0	255.4296	86.99028
185.25	36.51	55.06667		282.5768		0	282.5768	85.61639
215.9672	37.12111	62.71111		280.471		0		
208.0556	38.25889	59.65	27.30916	263.4874	0			
11.25666	28.1439	37.75		7.865401		0	7.865401	76.345
10.16666	26.73066	34.6		7.178318		0	7.178318	79.80407
9.794441	27.92149	38.08667		7.52532		0		
10.41666	29.26204	40.16	9.936579	6.843103	0			
268.1494	51.48112	83.53		266.3456		0	266.3456	170.4843
239.6139	48.05778	82.58		272.2217		0	272.2217	160.3711
243.4094	42.94666	82.16333		271.5639		0		
240.6256	42.94666	82.06333	15.63441	270.6534	0			
253.7354	23.11889	29.41667		18.4		0	18.4	55.04556
248.7123	25.06945	26.83333		16.84		0	16.84	37.33334
256.1634	25.47333	28		16.73334		0		
392.4883	23.31889	83.7168		111.8614		3.48	111.8614	74.13711
388.653	24.21001	81.12927		112.4572		1.416667	112.4572	75.14822
388.1338	24.42001	85.00668		111.3695		1		
385.9879	31.66112	93.64001	3.333333	116.4111	0.833333			
11.69544	3.277777	21.84111		65.64149		0	65.64149	38.56147
8.897208	4.30833	18.07334		63.0466		0	63.0466	40.98
9.786674	3.899998	18.89333		62.10616		0		
10.51334	3.538884	21.85	1.145552	63.04852	0			
205.7532	13.15333	22.18667		44.72217		0	44.72217	44.88889
212.6443	14.20666	19.86389		46.76423		0	46.76423	28.60945
216.8204	16.35999	19.88667		47.34861		0		

For peer review only

197.7388	16.90443	19.145	0.963333	48.25102	0			
9.515555	61.91633	33.75		93.81488		0	93.81488	131.4347
7.5	59.89855	35.11666		90.90167		0	90.90167	132.9807
8.75	59.48078	35.63334		93.19389		0		
6	61.15645	36.05	17.85667	94.04446	0			
185.661	10.48404	9.738889		75.61643		0	75.61643	85.61049
160.8199	6.938892	1.986667		77.57154		0	77.57154	82.87222
152.1246	6.012226	0		82.42398		0		
143.8448	6.062226	0	4.583334	85.21175	0			
2.635557	27.73334	48.30477		205.6778		0	205.6778	105.7093
2.642223	25.56667	47.69746		204.5951		0	204.5951	111.8434
2.470004	27.48	47.37794		205.2648		0		
218.751	36.91889	56.21905	4.55	210.1527	0			
0	5	0		278.3116		0	278.3116	133.74
0	4.5	0		300.8449		0	300.8449	132.9656
0	3.5	0		333.3348		0		
0	1.666667	0	43.70427	340.1074	0			
153.6766	86.32448	9.083333		91.15467		0	91.15467	59.91778
152.3366	84.45133	8.333333		91.88774		0	91.88774	60.04667
146.6222	79.31836	8		89.32819		0		
164.8511	76.66836	7.583334	5.990004	86.75224	0			
358.6843	43.02444	75.56111		156.562		0	156.562	74.23112
359.9551	43.60777	76.66666		153.7537		0	153.7537	75.56556
367.2736	43.20333	77.34444		225.7234		0		
368.3841	55.59828	81.53778	6.626668	312.7706	0			
165.9678	34.43125	67.99223		46.82546		0	46.82546	80.63667
50.15885	13.23112	0.583333		55.06324		0	55.06324	75.73556
243.7425	9.983713	2.222222		53.92584		0		
6.072235	6.083337	9.547778		34.61945		4.33333	34.61945	39.11667
9.366115	5.540561	10.81111		37.29126		1.805554	37.29126	42.72222
26.39779	5.69334	15.00555		36.51693		0		
25.43556	6.551673	15.69333	31.94833	36.77082	0			
123.7966	19.34427	25.22334		10.53666		0	10.53666	29.68
124.195	22.98958	25.03445		10.51667		0	10.51667	34.93778
142.6778	23.83867	25.3		10.69333		0		
152.1711	24.17554	23.88333	10.25	10.69333	0			
75.24834	7.217777	6.071114		193.8227		0	193.8227	47.82192
73.88445	5.829444	6.570004		204.2133		0	204.2133	72.29692
72.95335	7.485561	5.98667		193.2669		0		
76.49224	8.560007	5.653337	39.89223	190.0966	0			
41.30004	11.56	7.516667		87.61659		0	87.61659	77.17222
42.18893	12.89334	6.833334		93.49991		0	93.49991	89.68333
188.1867	13.9525	36.49166		139.5122		0	139.5122	121.0457
178.6361	12.45052	37.65833		154.5889		0	154.5889	132.4675
174.5567	17.20942	38.35		156.5545		0		
172.7662	24.8124	39.59334	10.56167	163.3476	0			
123.3103	39.3919	93.92016		81.53803		0	81.53803	21.29889
237.5108	44.59332	99.21127		76.84614		0	76.84614	24.91445
242.5261	45.665	95.9532		80.09036		0		
242.4866	41.88688	83.87354	6	79.92647	0			

For peer review only

181.9733	26.39223	20.75		41.82221		0	41.82221	55.10333
218.0727	24.08667	19.91667		41.77332		0	41.77332	59.20493
225.9844	23.46001	23.58333		43.77001		0		
230.3061	21.76667	31.75444	16.11386	44.21666	0			
83.59068	25.4928	10.7		96.30274		0	96.30274	57.47333
96.8525	20.42287	3.733333		101.4966		0	101.4966	67.36889
96.45337	17.144	0.4		98.99219		0		
94.97325	15.01622	1.057782	1.344443	100.7494	0			
430.0875	28.0189	59.41666		115.2422		0	115.2422	64.58646
452.2344	32.18668	56.37222		125.6829		0	125.6829	60.24444
454.3733	31.31334	54.91667		122.5896		0		
482.3348	32.68334	56.31667	7.71667	120.3726	0			
0	0	0		38.76625		0	38.76625	51.3889
0	0	0		40.89818		0	40.89818	49.95557
0	0	0		84.991		0		
0	0	0	0	85.90823	0			
47.72755	21.34111	163.6805		73.49558		0	73.49558	37.02333
51.84089	21.07444	170.9204		85.31599		0	85.31599	38.90221
52.032	17.03778	171.3005		78.86325		0		
51.60401	15.41333	175.8953	0	78.03896	0			
51.5	7.193886	91.17266		2.329954		0	2.329954	88.85832
124.5296	7.43333	16.02334		0		0	0	88.13222
114.81	8.217217	16.89001		0.467422		0		
119.0631	18.08666	117.715	2.486667	48.08543	0			
110.2903	6.026653	27.49889		83.19403		0	83.19403	58.63112
98.55356	5.65332	45.93089		87.57716		0	87.57716	64.22668
165.2456	11.26576	46.77		87.51927		1		
158.5311	14.07665	103.3525	5.427779	84.4256	1.583333			
331.8128	18.54334	24.77334		87.71103		0	87.71103	94.79746
341.0553	19.52	33.67667		86.77213		0.5	86.77213	100.0469
347.2076	25.23334	37.56		91.45213		1		
308.7597	28.85889	102.8351	1.575554	93.41546	1.583333			
163.3523	51.46454	37.30222		65.39568		1	65.39568	67.92512
166.7317	49.1825	35.2		61.70609		1	61.70609	75.118
164.8708	47.28944	34.37111		58.49553		1		
166.6725	44.05611	31.47222	3.25	60.70887	0.166667			
479.1271	32.95335	62.45557		147.6143		5.166667	147.6143	82.34021
447.0807	33.32565	55.91056		145.8144		2.25	145.8144	81.23299
377.5837	33.16232	54.76875		150.9013		0		
353.2543	29.55121	56.16459	13.26667	152.1113	0			
265.1744	29.37333	63.11667		71.14542		1	71.14542	52.31444
306.4488	32.33112	62.53333		66.08017		1	66.08017	49.13111
308.6898	26.95222	58.175		28.25271		1		
294.1595	31.55444	49.61667	2.45	26.94493	0.166667			
107.5878	13.79223	7.933333		199.7148		0	199.7148	156.5138
102.9867	11.83334	4.8		204.6877		0	204.6877	164.3279
102.2383	11.02334	6.311111		217.1394		0		
103.8307	10.90667	8.73333	64.11732	237.6709	0			
272.7331	14.70334	95.69333		74.27013		0	74.27013	53.43557
265.2337	19.07667	99.70333		69.77602		0	69.77602	55.98581

For peer review only

264.0637	19.92667	103.5889		77.26		0		
262.9476	24.02061	106.177	7.174543	90.38023	0			
0	2.416667	0		44.92136		0	44.92136	99.41667
0	1.416667	0.416667		37.58775		0	37.58775	116.2644
0	1	1		35.9372		0		
0.083333	1	0.5	37.92389	35.25386	0			
81.04183	10.90327	6.727273		6.164852		0	6.164852	49.94303
185.5212	15.67335	22.86889		53.77499		0	53.77499	52.37
192.2336	13.35334	22.41667		56.7722		0	56.7722	71.25333
226.5006	12.40334	21.33333		63.30166		0		
224.8692	12.56334	25.46111	2.90667	66.80555	0			
20.55944	8.117207	22.93445		89.20166		0	89.20166	46.03667
21.96666	12.59134	20.85444		91.52222		0	91.52222	42.09666
21.92	17.57467	18.81		92.15055		0		
25.16612	16.54134	18.24166	0.383333	95.43333	0			
323.8727	76.55001	110.0356		212.5354		0	212.5354	104.3617
446.2387	65.92121	110.2909		227.499		0	227.499	93.58424
250.6729	20.47333	44.37111		112.7393		0	112.7393	55.99352
260.9061	23.96833	68.13333		122.0467		0	122.0467	67.89778
262.7272	24.45444	81.36667		123.89		0		
284.05	22.55111	81.28333	31.03222	121.3236	0			
1283.467	75.72221	150.1667		335.8978		0	335.8978	91.01111
1281.718	79.98666	145.6667		339.5671		0	339.5671	92.29556
1322.004	83.13778	140.6		354.1958		0		
1352.366	87.33	148.65	4.43	362.7796	0			
293.7972	54.2772	57.29796		28.12227		0	28.12227	67.44279
297.1322	53.35553	56.0524		30.92717		0.233333	30.92717	65.13666
290.3522	49.67886	53.76462		30.52759		0		
293.9444	66.45386	54.42646	6.666652	32.66775	0			
622.6184	33.38268	1.53333		165.3378		0	165.3378	58.74587
690.9894	34.30604	10.95		199.8756		0	199.8756	69.38742
1314.204	87.14547	107.4833		432.0734		0		
1350.653	86.49722	112.04	18.04	442.9695	0			
109.3255	50.05534	3.4		126.8966		0	126.8966	335.5304
112.7839	53.03423	2.9		144.8344		0	144.8344	360.0385
121.4583	55.41523	1.65		151.7939		0		
125.0367	55.08703	5.35	34.33333	170.5522	0			
644.0097	42.68499	154.5333		270.8365		0	270.8365	168.9911
638.4003	43.57721	167.1011		282.3247		0	282.3247	184.6989
643.2083	48.42334	169.8561		274.431		0		
642.336	51.13445	178.2456	25.77166	293.9052	0			
292.9765	35.40423	67.59844		207.6182		0	207.6182	79.95445
275.8561	34.38445	66.18555		201.1352		0	201.1352	84.04889
243.3359	33.33556	61.75444		212.6947		0		
269.3821	27.83445	63.76944	5.166667	215.946	0			
473.6767	25.13334	80.51981		61.86494		1.5	61.86494	146.8087
164.51	13.44222	34.28333		63.84888		0	63.84888	143.0022
38.52002	8.483327	21.33333		64.65332		0		
42.61778	7.227773	19.41667	46.7447	65.49332	0			
117.8974	18.40556	57.80281		213.0851		0	213.0851	101.1176

For peer review only

118.5937	16.54556	62.72448		219.1326		0	219.1326	108.1215
316.648	34.57944	85.90894		254.8409		0		
356.5018	41.19333	85.16338	23.46	294.0262	0			
83.47938	29.13333	47.43333		201.0092		0	201.0092	180.2611
79.25658	32.43333	46.21667		206.7174		0	206.7174	183.4562
111.0236	32.56667	48.06667		211.6267		0		
120.9889	32.35	44.46667	19.21	235.4404	0			
147.2319	25.36146	29.43333		60.77627		2.333333	60.77627	47.77334
149.8189	24.32548	28.91667		65.67412		1.166667	65.67412	47.72278
153.1611	24.71574	52.20778		65.96191		0		
154.3267	25.69716	64.25333	0.850463	79.37212	0			
252.8977	12.05101	15.70334		62.09926		0	62.09926	64.63487
259.4194	12.12668	14.45889		60.3724		0	60.3724	65.56111
261.6283	11.01779	15.31667		61.71407		0		
280.2638	11.22556	19.91667	3.791667	72.37489	0			
181.2678	22.11611	37.00222		133.6		0	133.6	71.73
164.815	16.42	40.34		137.8645		0	137.8645	83.61888
161.3089	12.77667	46.47667		135.3861		0.083333		
158.5723	12.05	45.91333	0	141.3523	0			
395.8055	28.91016	82.29778		65.7741		0	65.7741	131.1633
388.6505	29.80482	84.83		63.68932		0	63.68932	149.5745
392.8116	31.5226	89.6289		68.61322		0		
402.1697	33.8926	87.89778	0	73.40432	0			
472.8266	80.24935	29.75		248.7782		0	248.7782	213.7494
500.7089	65.3659	26.29025		241.591		0	241.591	215.9768
520.2756	67.65514	27.46667		250.5104		0		
523.625	62.37737	26.90001	6.463486	267.2777	0			
47.09667	13.36667	42.55		111.1722		0	111.1722	55.17418
45.02796	13.69723	38.675		109.6963		0	109.6963	63.64779
19.16164	16.29445	39.91667		105.6229		0		
20.44889	16.76667	41.33333	7.083334	108.8879	0.75			
84.53519	36.35746	64.33714		168.2668		0.666667	168.2668	87.67445
86.68686	34.39088	67.25191		159.4839		1	159.4839	90.22556
507.5872	48.7208	66.08334		168.4397		1		
749.3021	57.09773	67.08334	57.3345	173.2847	1			
315.7607	18.88722	100.0635		87.78224		0	87.78224	63.74778
329.1378	20.365	96.9164		86.44893		0	86.44893	62.07556
370.4831	16.19666	61.32		87.20615		0		
369.0479	17.23555	61.24	1.166667	85.49838	0			
249.6026	16.75	36.11334		7.268337		0	7.268337	20.03333
240.9273	16.69556	36.96333		6.024446		0	6.024446	12.93667
235.9867	18.85112	34.88		5.800003		0		
229.2016	16.62556	34.20334	4.083334	5.835556	0			
88.10342	28.39333	17.75		247.8383		0	247.8383	261.5649
88.24231	43.388	17.16667		231.8231		0	231.8231	121.6989
521.1374	82.02119	143.1922		133.8197		0	133.8197	86.13612
527.2001	83.11009	141.1322		139.8496		0	139.8496	85.37222
533.6307	79.52132	281.6532		138.5884		0		
533.7342	70.39971	430.0884	6.052223	148.3328	0			
310.6612	56.25	79.74389		147.7882		0	147.7882	157.0989

For peer review only

315.6268	55.14388	82.64222		151.4857		0	151.4857	164.1889
327.0534	61.65665	82.91889		157.2674		0		
335.2723	66.31443	80.65	22.06111	152.2602	0			
316.4502	35.16556	73.81778		124.8919		0	124.8919	48.54555
321.4411	34.89223	75.36889		122.9078		0	122.9078	59.30111
309.0263	33.9211	71.05222		124.7378		0		
310.5109	33.80111	70.35888	5.236113	126.1595	0.333333			
1085.679	82.27783	97.26778		286.1996		0.333333	286.1996	70.76834
1215.245	79.4772	106.7478		303.3397		0	303.3397	83.96519
1274.723	65.84416	105.4322		332.3396		0		
1223.079	52.7711	95.88722	69.26676	331.2624	0			
459.5869	39.03406	33.91667		120.197		0	120.197	81.95777
437.9157	35.32721	32.75		122.2075		0	122.2075	80.78999
444.7891	35.02555	35.18333		123.2548		0		
435.8452	30.63831	33.01667	1.083333	128.575	0			
22.85222	24.69556	34.17667		104.4433		0	104.4433	56.86666
23.26	29.33001	39.14167		103.9729		0	103.9729	54.16667
23.79222	21.80112	39.98333		101.8418		0		
33.62333	23.53779	40.18333	33.50436	120.8314	0			
233.4684	10.83333	28.21667		30.43333		0	30.43333	47.04091
243.2216	11.36667	25.20833		87.54409		0	87.54409	58.94801
236.1722	10.46778	23.33333		116.6284		0		
251.3664	8.1	19.44444	22.60222	119.9273	0			
256.1772	32.70594	32.89		45.4148		0.083333	45.4148	78.77844
270.1148	21.68928	30.19667		46.18055		0	46.18055	84.13956
288.4095	19.42355	31.09333		50.31444		0		
308.8317	19.05577	30.32333	8.066107	53.55612	0			
128.3977	29.22319	24.08333		89.51057		0	89.51057	66.68389
133.0754	29.74444	28.16667		96.62389		0	96.62389	74.27445
142.5132	26.98777	30.91667		95.33845		0		
152.731	22.58999	30.5	9.291667	95.35655	0			
213.6103	20.25111	42.49		62.72646		0	62.72646	50.46406
204.6719	19.38888	42.61778		60.4387		0	60.4387	52.69833
202.7457	19.94666	47.55889		55.61361		0		
212.9226	20.6661	48.24778	5.583334	58.03109	0			
237.701	47.50557	25.92333		122.4828		0	122.4828	105.0628
249.8049	50.08223	24.93222		125.0268		0	125.0268	117.3806
253.3027	46.84934	29.21667		112.4935		0.5		
250.3374	50.03876	38.33889	6.382221	114.1957	0			
62.28778	24.20667	4.868889		188.7001		0.416667	188.7001	242.7127
750.8021	167.1628	133.2127		177.1417		0	177.1417	255.6046
820.1185	170.4506	120.46		165.7214		0		
755.1199	74.04499	221.0583	151.5072	194.8456	0			
468.016	36.75379	67.79401		108.6052		0	108.6052	87.69222
457.5565	27.48556	66.12734		109.7747		0	109.7747	103.2833
467.3974	32.92222	75.97012		108.7127		0		
434.8635	41.67533	121.2151	0	116.1641	0			
395.9894	53.23653	34.65333		138.5658		0	138.5658	88.24703
424.8809	51.16013	36.80666		153.2343		0	153.2343	95.23967
505.3632	52.81382	37.09		153.379		0		

For peer review only

For peer review only - http://bmjopen.bmj.com/site/about/guidelines.xhtml

521.27	61.09326	36.84	12.15	152.1578	0			
533.4711	105.3968	57.73333		121.4636		0	121.4636	106.1823
532.1532	102.7072	55.44166		122.2663		0	122.2663	114.3579
568.832	104.2256	59.1		123.7796		0		
582.1959	111.2261	58.58611	0.341667	125.3742	0			
405.1673	59.8711	66.06667		154.8945		0	154.8945	95.63
397.5331	58.74022	58.11667		166.9753		0	166.9753	101.3044
393.2906	57.37078	54.68333		125.3996		0		
406.7198	52.76466	52.60555	35.77166	81.53155	0			
218.3996	26.19389	56.3		109.0224		0	109.0224	84.03722
179.391	20.02444	38.66667		101.5209		0	101.5209	68.81334
177.5344	21.15332	40.25		96.99315		0		
176.2255	17.38666	34.08333	15.9589	95.02818	0			
541.4751	64.68371	63.67		140.8711		0	140.8711	80.79467
557.6115	59.9141	64.74889		141.1457		0	141.1457	89.31334
504.8312	61.33	59.65445		143.5731		0		
513.7585	66.01325	60.25667	4.363887	161.0185	0			
244.0442	23.86009	42.69435		100.861		0	100.861	104.7987
238.153	19.51621	22.86657		103.7674		0	103.7674	116.0994
228.6545	18.12045	16.66213		107.7523		0		
257.5061	16.2812	18.16214	11.44667	104.6957	0			
39.21003	18.58787	36.38333		74.91255		0	74.91255	97.13324
34.33447	18.77898	33.75		75.29645		0	75.29645	104.7066
33.04781	17.42176	34.33333		77.49596		0		
36.17242	16.14898	31.25	1.25	73.42811	0			
533.8164	44.77224	25.56667		80.5678		0	80.5678	66.41187
537.9377	37.00668	25.58333		79.2558		0	79.2558	75.02242
538.49	39.08223	29.75		76.27335		0		
554.0689	58.42833	35.67222	9	75.30557	0			
211.221	22.93	44.53333		114.7283		0	114.7283	110.2673
215.5705	24.92	44.29167		120.2978		0	120.2978	119.5144
214.8366	28.68223	46.94222		129.45		0		
229.3894	31.58501	48.31889	0	137.5072	0			
0	9.10333	18.2		225.4502		0	225.4502	340.8121
0	9.985554	14.25		264.8117		0	264.8117	364.8796
0	17.16667	15.58333		275.9868		0		
0	23.83333	17.3	48.42611	284.6882	0			
455.3383	52.52335	42.51667		190.1709		0	190.1709	48.92556
397.2163	53.6903	52.7		195.5408		0	195.5408	49.84
392.2139	52.59511	53.06667		196.719		0		
427.5227	51.66846	56	6.526667	202.8762	0			

For peer review only

v91	ratio_bed_	ratio_bed_	ratio_bed_	ratio_bed_	ratio_bed_	ratio_bed_	ratio_bed_	ratio_bed_a
31.49667	0.808602	9.943149	4.008939	754		205.6364	5.300879	2.8009
45.06333	0.776433	9.348506	3.646085	733.25		366.625	4.998353	2.613583
	0.762823	8.972651	3.497532	586.6		1466.5	5.045414	2.526357
	0.702522	8.552788	3.588251	253.3235			5.174298	2.364664
52.6	0.446259	2.983208	5.418022	88.10356	150.4068	135.6235	3.941626	1.853882
	0.451149	3.012837	4.198886	92.38548	142.7882	122.9676	4.066015	1.757527
	0.423778	2.705761	3.286224	78.17693	120.7737	114.6462	3.994326	1.513017
	0.532192	6.536094	1.820556	40.90294	36.96	1212	5.835978	1.87571
13.67778	0.612225	10.68093	2.723851	20.65445	2.34098		6.348229	1.740091
14.97389	0.571379	9.056706	2.936448	17.01923	2.179161		5.87948	1.637345
	0.610743	8.27391	2.851212	18.85023	2.084296		5.692683	1.76867
	0.614757	7.662826	2.532981	21.98416	2.090087	595.8	4.944782	1.757057
69.50616	0.531753	4.954615	16.13828	476.0217		50.80982	3.38912	2.332604
83.49999	0.458354	4.292586	39.17532	1681.5		40.929	3.091817	2.16743
	0.450122	4.266055	42.06669	1699.5		47.76112	3.154661	2.133379
	0.448425	4.579007	41.69189	1741.25		64.33189	3.365773	2.184007
0.083333	0.547531	12.72291	3.725521	384.8823	3271.5	154.5591	7.171516	1.601356
2.606667	0.62092	15.87019	4.652258	296.7		260.2632	8.676844	1.769316
	0.652303	16.60486	5.176337	238.958		214.7083	9.676333	1.846241
	0.579961	15.15646	7.727496	913.2673		424.8	12.17759	2.140979
5.996666	0.495358	10.37883					4.966426	1.493935
5.843332	0.429028	9.004092					4.400963	1.314506
	0.451744	8.642005	4.254663				4.430375	1.183153
	0.427721	8.783534	4.208526				4.328389	1.14224
9.116667	0.831221	47.53246	316.2672		47.82715		11.19947	3.282243
8.202223	0.796664	45.42582	254.3038		63.68164		11.22041	2.929822
	0.765506	38.63403	174.4788		58.50208		14.14872	2.840493
	0.780858	36.76909	119.7106		41.81556		92.84309	3.044793
10.80889	0.78023	19.5001	1393				6.266268	3.595245
7.213333	0.778393	19.26265					6.589755	3.368547
	0.713924	15.80004					6.206768	3.182039
	0.665686	16.61504					5.847156	3.441399
4.443337	0.563245	1.138153	38.80008	603.2692	97.93658		5.155968	2.079765
3.981668	0.555297	1.111389	35.80451	561.0714	81.51555		5.308618	2.002265
	0.542758	1.077694	35.3534	582.1875	68.41577		4.860305	1.905849
	0.534629	1.061823	37.62985	568.5714	78.29399		4.379777	1.758429
17.16589	0.418547	8.505251	1.810717	230.454	60.53731	104.0712	4.125957	0.884147
4.501113	0.404242	6.948488	1.742363	216.1359	33.72366	330.7826	3.932246	0.826711
	0.43471	5.353245	1.82997	224.9995	32.3794		3.969304	0.849666
	0.439379	3.148772	1.955696	224.0105			4.09493	0.740017
19.16667	0.540052	3.78268	7.932231			772	2.991038	2.081641
13.5	0.564426	3.905731	7.890277			384.4957	3.111432	2.05802
	0.643964	4.428905	9.594224			1586.047	3.484969	2.33011
	0.554315	3.958589	9.072119			793.3333	2.931687	2.031807
13.60222	0.565133	7.616906	421.9286				4.049534	2.006663
13.70333	0.573758	7.514944	546.7783				3.875553	2.035163
	0.568431	8.084844	550.3323				3.989723	1.973311
	0.445439	7.582505	506.5	11396.32			3.247127	1.706594

For peer review only

18.02011	0.454148	6.514637	161.4876	977		42.61723	3.446052	2.072389
20.47611	0.456926	6.743698	108.8171	981.5		39.26	3.56303	1.996183
	0.472017	6.824187	52.77226	998		49.9	3.507319	2.002717
	0.433042	6.417286	54.44843	950	105.7513	64.40678	3.046325	1.859419
11.36124	0.599886	4.136403		1146.875			3.101763	3.229977
4.81763	0.608844	4.589276	25.63749	1212.5		33.83721	3.728577	3.151477
	0.617058	4.818186	24.52365	1822.5		43.84963	3.796912	2.945712
	0.629359	5.372809	25.42543			58.64423	3.684915	2.702827
7.244444	0.746457	7.476411					3.631397	3.002016
2.856667	0.729358	7.255408					3.408016	2.957912
	0.680477	6.672886			5436	1087.2	3.320587	2.73834
	0.640053	6.154049			310.9412	1762	3.26837	2.807733
10.91778	0.686281	4.829352	83.67512			95.71154	2.655537	3.428701
10.77556	0.669268	4.658401	71.38855			96.22642	2.622579	3.082189
	0.577237	4.565578	64.11334			69.30434	2.35575	2.777467
	0.525534	4.531467	210.4668			152.0625	2.35862	2.653431
51.68167	0.532774	5.859074					4.112247	2.1714
50.76667	0.56106	5.994607	334.1232				4.022865	2.129501
	0.591358	6.34204	204.7029				4.173802	2.047312
	0.580702	6.729553	96.634				4.340824	2.13931
14.14556	0.581479	7.067598		740			4.684408	2.404084
8.75	0.57435	7.519047		487.5			4.612917	2.29313
	0.575426	7.578822		452.6923			4.557697	2.265816
	0.569378	7.891267	219.717	376.875			4.46663	2.298546
0.81333	0.775619	6.81911	1.940082		93.86478	58.13684	3.038712	2.284134
1.81333	0.731107	5.799764	1.823521		61.35091	246.3617	3.056066	2.162012
	0.70478	5.632644	1.780018		64.11008	414.9171	3.058374	2.120095
	0.740809	5.206798	1.777036		59.0554	214.3633	3.109804	2.097578
2.716667	0.529457	6.808004	124.2451				8.597049	2.613136
6.638892	0.584146	6.366707	203.2186				8.006477	2.703563
	0.614801	6.221332					6.196059	2.672344
	0.596618	5.896872					5.907503	2.491149
1.383333	0.599218	7.307656					5.373358	3.184914
1.85	0.58997	7.581729					5.197728	2.99066
	0.567933	6.992147					4.969612	2.811894
	0.551956	6.954373	86.01227				5.02591	2.726324
0.75	0.725874	8.317625	6.233566				4.818296	2.815507
0.583333	0.717619	7.469378	3.122279				4.65939	1.873385
	0.762619	7.602462	2.923786				4.776581	1.826959
	0.735291	8.116139	2.861734				4.896042	1.75117
0	0.551128	6.352548	1.055761		316.9445		4.286239	1.015715
0	0.594777	6.010429	1.699753				4.308161	1.330685
	0.585915	5.274615	1.653749				3.959186	1.241831
	0.5872	5.424299	1.627662				4.15471	1.24547
0	0.627088	7.443902					4.232151	2.622917
0	0.647688	7.200452					4.569691	2.591031
	0.662528	8.334963					4.749927	2.684134
	0.645269	8.434721					4.704731	2.74376
14.14	0.739278	3.562154				126.579	3.589909	3.440058
18.45944	0.743828	3.517716				91.57317	3.585434	3.444782

For peer review only

	0.709869	3.47218				89.32143	3.581366	3.187709
	0.633851	3.455056				131.7931	3.631959	2.777593
10.25	0.779623	13.10626	4.959185				7.40751	2.178132
4.75	0.757882	12.34912	5.065621	10867.5			6.93621	1.997232
	0.779308	12.82492	7.093649	1814.583			6.930029	2.153292
	0.769546	12.49072	7.457734	1327.041			6.818134	2.302868
12.83333	0.559759	8.757236	1.158085			47.91608	4.099721	1.10356
13.36667	0.566157	8.839337	1.1162			41.96363	4.218659	1.11397
	0.531604	9.257508	0.850364	588.4605		22	4.010868	1.090109
	0.533672	8.952972	0.775048	134.0979		28.13278	4.058779	1.045821
5.903237	0.75608	8.234624	5.695945		435.9375		4.330326	2.010143
6.693243	0.729577	7.810288	6.376667		436.5625		4.593817	2.002169
	0.662202	6.878813	5.084332		390		4.406711	1.658164
	0.74883	7.007244	4.161633		235.4651		4.755233	1.750058
4	0.755659	7.599743			144.9512	152.3846	5.710616	3.63963
3.34234	0.799605	7.915638			182.2192	150.2195	5.630211	3.737396
	0.805629	7.703085			134.6019	253.5	6.062177	3.664241
	0.757877	7.892446			154.8995	880.7143	6.162371	3.85774
7.873889	0.654021	8.082704					4.277291	2.693879
5.344443	0.636659	7.297266					4.14305	2.399272
	0.670018	7.998321					4.48714	2.385104
	0.649231	8.034432					4.529729	2.313221
20.35418	0.716462	9.91574					4.640942	2.19956
21.95712	0.676321	9.013893					4.111407	2.063315
	0.728014	8.316018	1089.5				4.086477	2.044897
	0.726392	8.299271	564				4.260535	2.098831
11.93333	0.811167	5.617734					4.275759	3.890434
14.16111	0.786069	6.073292					4.854905	3.439109
	0.78131	6.161079					4.79827	3.532446
	0.794669	6.233634					4.359689	3.479558
12.87889	0.674194	7.501376					4.34336	4.069947
10.30778	0.699223	7.561562					4.669565	4.12295
	0.685139	7.163398					4.639773	3.874452
	0.635312	7.33235					4.473908	3.546603
10.92778	0.686101	6.051701	300.8898				4.932073	2.566232
15.13333	0.695063	5.792096	173.3344				4.816663	2.648736
	0.660861	8.018049	7.60072				5.049067	2.325721
	0.592597	7.831877	6.332509				4.950841	2.222581
16.52889	0.71888	9.692759	156.1794			5401.027	4.905663	3.817684
23.30334	0.69651	9.241086	40.46818			1117.5	4.914342	3.465615
	0.672145	9.162414	45.13848			1931.25	4.876762	3.245493
	0.61412	8.694382	52.08573			1276.5	4.452698	3.082941
2.210557	0.619136	805.2	61.78635				19.77082	2.876031
4.385558	0.621335	178.8667	69.44378				19.09276	2.794548
	0.620282	556.0417	72.97664				19.36147	2.636932
	0.607403	618	70.64718				20.02492	2.603625
12.80834	0.761179	6.642578	0.49322	11.76452	11.33927	164.5786	5.618772	1.511166
4.833337	0.756484	7.437141	0.548407	14.00741	10.37342	132.4404	5.612802	1.780929
	0.738787	6.727571	0.561684	13.57095	11.62598	367.2128	5.544571	1.727497
20.34333	0.689849	6.600595	76.49522	256.7734	2316.667		5.159445	2.187154

For peer review only

19.33779	0.69196	6.729641	115.2067	254.5569	1448.834		5.214958	2.200336
	0.676141	6.847405	114.9016	160.1903	1016.382		5.047255	2.119318
	0.646263	6.776846	197.2934	136.9028	811.5266		4.713433	2.245345
1.616666	0.72419						341.861	2.615948
1.85	0.731261						360.5732	2.654026
	0.746279						367.7562	2.705939
	0.601476	514.8	72.52065				698.1929	2.23407
6.002223	0.624419	6.591482	930.25			44.652	3.612173	2.770501
5.883333	0.598455	6.450168	882.25	2117.4	1058.7	40.10227	3.242971	2.511449
	0.585144	6.294802	866.75	742.9286	371.4643	35.74227	3.188314	2.494978
	0.583788	5.9195	1156	385.3333	270.0937	50.26087	3.283477	2.43232
46.62127	0.592755	5.329815			743.7097		3.453802	2.706113
51.14905	0.616112	5.448161			519.8936		3.601272	2.637525
	0.576702	5.105297			330.2055		3.49884	2.420827
	0.581998	5.404497		1644	411		3.497166	2.383115
19.27413	0.700553	7.015625	2.063683	813.8571		633	5.15964	1.688685
12.44666	0.713205	6.854715	1.972967	474.5		355.875	5.374399	1.58396
	0.692172	6.233681	1.843131	452.5		339.375	5.058218	1.516698
	0.66028	5.160464	1.902009	439.25		405.4616	4.766454	1.438008
22.42417	0.65566	6.768847	37.15532		461.5	1107.6	4.968554	2.443329
40.36833	0.642169	6.214104	18.05869		453.6083		4.730589	2.256661
	0.586879	5.787209	11.32936		464.2857		4.398026	2.045542
	0.619035	6.204866	16.91948		503.5714		4.547259	2.132941
26.68167	0.561693	8.794866		6858			4.451002	2.622291
18.86778	0.548177	11.75536		764.75			3.544589	2.683717
	0.554811	11.61716		4684.5			3.650384	2.630388
	0.579285	6.59021	228.0263				4.20187	2.955999
0.083333	0.664107	7.852009					6.367909	3.601498
0	0.656027	7.899263					6.244272	3.551547
	0.656526	7.926009					6.298574	3.306782
	0.683626	8.121388					6.771336	3.620745
9.907755	0.679792	15.21264	7.724294				5.026996	2.536481
11.2333	0.698887	16.25765	4.705185				5.359085	2.404876
	0.713051	18.05111	3.396152				5.189489	2.191876
0.230001	0.538307	8.803427	19.63951	640.2857			4.069021	2.327217
0.339202	0.561724	8.02776	5.335598	374			4.377284	1.682788
	0.564876	7.92318	2.885972	358			4.17701	1.286224
	0.515639	7.155232	2.702508	358			4.066949	1.192418
119.5529	0.462857	3.140584				116.3724	6.223231	2.319717
99.60443	0.473731	3.100044	5135			422.0548	6.002573	2.244156
	0.43989	3.052964	1290.313				6.074244	2.171853
	0.395082	2.859229	1271.25				5.666556	2.006676
43.24333	0.590064	103.2468	6.777983		927.5		6.11241	2.25503
35.79	0.595042	91.46119	3.415198		462.5		6.011015	1.768802
	0.575703	55.84324	3.205827		450.4167		5.989378	1.627615
	0.540978	16.02586	3.179083		487.8		7.369842	1.589191
0	0.478013	2.684982					5.950569	2.309903
0	0.465669	2.537192					5.481469	2.11899
	0.465103	2.46165					5.20408	2.032093
	0.440544	2.397263					5.091887	1.985536

For peer review only

115.7006	0.535705	13.72877	4.120045			3165	7.165566	2.077842
88.35389	0.532274	13.46398	4.040263			19038	7.182982	2.056073
	0.512619	13.24518	3.796009				6.826256	1.894335
	0.494238	14.0031	3.550673	1462.25			6.455771	1.7973
25.45055	0.57318	7.386356					7.196729	3.422791
39.27667	0.590409	7.392121					7.032316	3.334436
	0.604116	7.510125			637.7368		6.911262	3.222464
	0.580562	6.086349			4050		6.345778	3.094852
1.4	0.547176	6.40922	68.62276			135.9661	4.519081	2.744008
1.333333	0.548188	6.309813	70.14427			169.1087	4.452608	2.548873
	0.513057	5.943287	65.79089			108.2647	4.035758	2.33694
	0.485945	5.959815	85.50877			304.625	3.93052	2.159596
14.16667	0.323837	1.650307	2.46416	126.8836		241.6304	3.689099	0.994576
18.71322	0.327342	1.638342	2.609436	106.6605		642.3333	3.865906	0.948208
	0.31367	1.502569	2.587683	103.836		327.0833	3.890439	0.906701
	0.300294	1.405219	2.439509	115.3685		236.3878	3.71482	0.835588
0	0.678564	5.186515	5.55341			193.2857	3.39954	2.586753
0	0.6996	5.236533	7.505889			489.6818	3.257609	2.494288
	0.684627	5.25487	7.628163		2154	598.3333	3.211316	2.399041
	0.630943	5.109363	7.914865		639.353	1207.667	3.200305	2.316264
8.962511	0.750225	6.171621	3.614423	73.71536		95.95082	3.874963	2.058707
9.013335	0.770687	5.86712	3.622206	74.2803		52.52679	4.147634	2.01438
	0.778628	5.679929	3.3942	80.38997		45.80953	4.242872	2.024631
	0.760345	5.381078	3.201898	78.04878	316.4835	49.23077	3.941337	2.076497
44.21859	0.431094	4.951019	4.623869	196.8			5.149213	1.595298
45.72693	0.447422	4.815519	6.137854	188.7931			4.926985	1.572959
	0.418765	4.425887	12.15243	188.7778			4.587553	1.427863
	0.417088	4.020886	26.10127	559.1667			4.572991	1.458908
10.71253	0.783706	13.54564	1.398382	658.5226		360.5625	5.213609	1.269307
12.46386	0.806193	13.77828	1.410944	227.5804		814.8503	5.109008	1.263064
	0.791665	11.31112	1.239782	254.1427	2769	2769	4.497711	1.173851
	0.776812	11.48457	1.173661	508.3647	450.75	450.75	4.349367	1.111813
22.46867	0.55122	7.925724	787.207		2687	435.7297	5.816325	3.135586
23.26554	0.570437	7.9391	5216.25		1192.286	370.9333	6.108171	3.224918
	0.571008	7.61911			474.5143	2076	6.347362	3.100745
	0.567203	7.358385		20617.5	267.9337		6.359523	2.960934
3.990001	0.667978	9.629698	1200				4.618069	3.65699
3.330002	0.568704	7.5689					3.559704	2.895993
	0.491129	6.458278					3.085005	2.465068
30.82945	0.663182	5.725754	4.924602	4015.5		133.85	4.904447	1.958296
26.60556	0.650436	5.790169	4.988	659.25		608.5385	4.593027	1.961506
	0.682538	5.902175	5.166919	689	8268	318	4.529005	1.977497
	0.673223	5.584064	5.133739	690	828	285.5172	4.442139	1.968475
1.918887	0.792811	12.98227	1.55523	835.625	334.25		5.870673	1.399754
1.63333	0.779465	12.88825	2.438368		328.5		5.572521	2.147232
	0.775144	12.05455	2.511743		565.7143		5.653374	2.082241
	0.811504	11.967	2.944372				5.855573	2.024742
5.744444	0.641664	7.581367	498.5				5.519574	2.682026
9.916666	0.694477	8.41655	586.3637				5.493228	2.718049
	0.670135	7.940233	870.1754				5.364807	2.593896

	0.623511	7.325744					5.02022	2.357551
43.03111	0.463634	4.247895	354.375		1545.398	156.6	3.719266	2.666975
22.6	0.454914	4.348728	300.2632		1426.25	172.8788	3.941408	2.553571
	0.41494	3.715945	308.9455		2022.857	147.7565	3.759843	2.501343
	0.42847	4.007669	382.5	1955	2199.375	314.1964	4.114124	2.484409
22.31667	0.649793	9.763698	27.81517			435.5625	5.394628	3.077783
16.43889	0.600676	7.979311	185.3287			6657	5.224816	2.714102
	0.62198	8.199604	230.8158	1278.889			5.530493	2.670445
	0.590695	7.907393	227.8078	946.6667			5.301832	2.580808
60.36278	0.638564	6.353435	199.1929				6.471861	2.703264
49.51556	0.617518	6.151581	155.2294	604.2857			6.572924	2.548686
	0.598691	6.075487	147.8134	352.0833			6.73114	2.405555
	0.564986	5.764956	139.6566	252.9105	1868.927		6.512864	2.379348
42.56315	0.665929	6.644097	181.5684		3665.102	627.6605	6.466813	2.847872
53.44983	0.640311	6.493705	149.2089			754.3333	6.37015	2.655632
	0.614167	6.23004	143.4473			978.8571	6.503784	2.59184
	0.585464	6.159343	107.1563		2143.125	4572	5.894754	2.646565
4.772227	0.537328	2.634626	1.123958	24.36452	24.9403	32.4466	2.142009	0.70483
5.311118	0.586171	2.818685	1.308744	34.25409	30.39823	30.66964	2.034294	0.700218
	0.580626	3.080165	1.3549	49.35512	34.96447	40.04651	1.987297	0.672223
	0.54156	2.886064	1.318798	37.44926	31.47867	48.83823	1.872951	0.621583
12.64445	0.615348	7.525112	3.449242			171.1452	5.362768	2.271905
21.66764	0.588799	7.217984	3.305752			1150.333	5.01261	2.222258
	0.586966	6.537387	3.49332			10473	4.793862	2.220617
	0.56469	6.128513	3.479629	293.1667	458.8696	422.16	4.517662	2.151809
15.5812	0.493476	6.28927	56.57008				5.711257	2.02539
27.4123	0.483657	6.121574	54.91522				5.485891	1.833423
	0.481084	5.908804	66.71442				5.3194	1.835035
23.67721	0.504013	5.91474	2.619398	90.43774		442.7143	3.728075	1.307405
30.31389	0.482133	5.55896	2.745438	427.5862		248	3.720806	1.258535
	0.491273	5.467244	2.677693	6237.5		598.8	3.686163	1.289488
	0.449195	5.455947	2.43465				3.739401	1.251145
14.33	0.654153	9.422481	31.65424				3.942522	3.848435
14.28333	0.632337	8.7137	39.87387				3.753124	3.473211
	0.637588	8.699117	86.16118				3.690436	3.533879
	0.627054	8.920925	312.5				3.795982	3.647041
50.48333	0.67822	7.880881				93.40601	5.483623	2.678404
67.16666	0.60448	6.64772				84.74452	4.99501	2.386542
	0.584445	6.722658				143.5244	5.096924	2.290324
	0.578533	6.577725				492.84	5.442699	2.232431
29.21223	0.469429	6.960936	5.251942				4.583376	2.123696
58.56	0.451955	6.400344	5.1535				4.324465	1.895317
7.069444	0.492235	28.62484	2.833694	95.63781	699.75	524.8125		1.727362
7.736667	0.517777	25.97646	2.855295	76.77145	421	680.0769		1.721267
	0.514347	29.20822	2.72977	79.1958	377.5	906		1.675278
	0.487898	22.89639	1.326766	71.52582	400.6868	326.4643	9141	1.13161
10.3	0.621192	4.351406	1.875884	35.39362	142.7897	68.50377	2.664654	1.561188
12.5	0.597994	4.241208	1.902729	28.16638	110.6	100.5455	2.659825	1.511176
	0.553132	3.854276	1.949427	24.89583		129.06	2.688952	1.4186
	0.534946	3.232293	1.834449	26.56328		63.59406	2.535453	1.348871

For peer review only

For peer review only - http://bmjopen.bmj.com/site/about/guidelines.xhtml

20.0822	0.784271	11.3315	2.863376	47.59024	572.5162	328.7647	4.865416	2.106451
35.48887	0.706652	10.66021	2.586619	40.56989		286.3333	4.512661	1.821713
	0.673916	9.559506	2.489178	38.72409		290.2941	4.23	1.764662
	0.675837	8.757353	2.471138	37.37548		218.3182	3.986951	1.607787
48.32777	0.665619	12.83159	2.21474	42.32785			4.24415	1.592091
56.62778	0.698676	14.60355	1.7861	41.20548			4.209105	1.542806
	0.703855	14.12663	4.785965	7380			4.127763	2.83801
	0.682593	12.23312	4.538913				4.290428	2.783116
10.18334	0.66353	6.632839	102.5077				4.27824	2.863643
6.773338	0.630519	6.456167	121.9992				4.173721	2.656628
	0.620212	6.625337	134.8613		2886		4.232457	2.560654
	0.587567	6.864428	117.5131		881.8539		4.193764	2.593047
0	0.753367	6.856645	941.1429				3.717958	3.569775
0	0.725591	6.57261					3.711494	3.232831
	0.710555	6.566161					3.646321	2.993655
	0.691819	6.343525					3.48907	2.887074
0.25	0.625321	10.10204					4.789702	3.379355
0	0.615584	9.188817					4.603069	3.2414
	0.629907	9.395981					4.615303	3.252986
	0.674132	9.656511					5.005966	3.460571
9.055997								
11.79557								
	0.525993	3.383076	1.118868	14.24855	2.429206		4.577022	0.771823
16.93444	0.760033	12.29578					6.409543	6.008907
8.914437	0.747526	13.13471					6.016831	5.780542
	0.721832	11.11457					5.077766	5.403071
	0.653295	8.902					4.490675	4.680076
24.41666	0.759313	7.337953	73.49397	732			4.564133	3.905059
15.56333	0.770101	7.168364	86.23282	754.25			4.593884	3.863256
	0.730851	7.009142	97.47137	737.75			4.740245	3.660312
	0.716706	6.727558	93.92405	742			4.786157	3.738303
1.761114	0.746433	19.89255	35.83803				6.360039	3.316088
2.133335	0.798809	18.95045	35.64669				6.009925	3.152431
	0.797337	17.41748	36.06535				5.949179	3.083991
	0.705205	14.02016	35.66478				5.916107	2.853733
15.37196	0.631903	7.506745			814.75		5.123325	2.645769
19.14667	0.608871	6.904013			771.25		4.594077	2.448374
	0.601976	6.952428			755.5		4.487085	2.437496
	0.61242	7.624774			778.5		4.762986	2.521536
12.75	0.645904	6.420047	16.80709	88.9027		313.2632	3.133247	3.91737
6.583334	0.577259	5.770644	18.93918	96.31579	610	499.0909	2.969965	3.352037
	0.543523	6.491834	73.01452	163.1024	492.2727		2.888965	3.090871
	0.492055	3.47818	486.9388	141.2715			2.811002	2.810717
47.9211	0.50084	2617.5	747.8571					2.223403
60.72833	0.50618	1249.875	36.59956					2.042005
	0.507335	325.1	29.82083					2.002521
	0.512829	204.875	28.16151					2.036649
15.21666	0.501007	7.399872					3.758084	3.228179
9.736663	0.533865	7.79438					4.057877	3.277215

For peer review only

	0.560485	7.42371					4.185211	3.3868
	0.502759	7.045223					3.716709	3.11507
20.4116	0.62246	2.866222	62.03546	410.3572	766	183.84	2.461549	3.08676
27.15342	0.629355	3.024535	58.34842	605.5769	787.25	145.3385	2.381822	3.061046
	0.554842	2.860964	49.47939	960.3125	768.25	109.0748	2.318958	2.880271
	0.504737	2.706981	167.5742	957.5	766	237.6422	2.30039	2.950231
3	0.513447			186.5417			2.394835	3.587294
20.27334	0.887784	7.690531	666				3.640022	4.352474
5.15667	0.925617	7.921256	449.7878	1177.778			3.707817	4.532137
	0.789817	6.493424	232.7542	457.75			3.174226	3.818968
	0.694412	6.11755	227.0902	923.5			3.367247	3.864035
13.5	0.550042	2.751943	2.415775	755.4		21.9593	1.658189	0.813958
21.68333	0.490257	2.477845	2.199633			17.22727	1.459743	0.732855
	0.380437	1.902576	1.741879	922		15.45079	1.1977	0.595471
	0.330559	1.550117	1.648642	233.4545		20.38095	1.087344	0.540589
7.14667	0.620038	6.807958	8.812225	488.3133	506.625	79.47059	3.688374	2.602662
9.300608	0.665305	7.390129	10.17773	377.6957	374.4583	97.68478	3.918649	2.665851
42.83944	0.716219	15.65398	5.6319		10.34685	158.7	7.360797	2.716602
26.40778	0.708652	14.45542	2.896785		10.51147	2110.496	7.527459	2.207946
	0.694352	11.45953	2.814231		9.838726	1040.5	7.249524	2.127652
	0.659514	11.17964	2.773797		8.979645		6.161336	1.968646
53.25778	0.629209	3.102353	2340.726			5243.25	5.139141	2.087067
54.92944	0.637911	3.213672	14512.44			4334.4	5.30087	2.141693
	0.628014	3.082751			3897.273	21435	5.245903	2.03152
	0.610149	2.912384			3533.5	1766.75	4.996309	1.917953
2.083333	0.520146	12.26396	10.71962			116.8023	4.527535	2.163873
7.688883	0.512789	11.53214	10.55581			191.9	4.380234	2.02207
	0.519644	11.4246	10.69869			192.7	4.348971	2.089938
	0.511755	5.790302	1.980679	49.06303		261.8182	4.332369	1.215666
2.514445	0.518126		19.50109					2.690577
3.431215	0.47431	72.06529	14.01946	3257.857	3420.75	855.1875	38.42999	2.330647
	0.588715	14.10584	13.19664	571.3838	774.225	289.4299	7.295095	2.530505
	0.584298	13.70574	13.76485	466.9486	702.5455	412.16	7.377472	2.459101
22.96889	0.393129	5.313959			835.75	130.2468	4.340606	1.755215
20.43333	0.380496	5.394778			798.5	100.8632	4.01496	1.558205
	0.355254	4.886117			776.5	238.9231	4.110924	1.454327
	0.339995	4.709168			589.125	204.913	4.000966	1.361218
28.28742	0.56463	2.173526	5.625244		367.1219	226.9481	5.96144	2.178559
53.96252	0.524289	2.012274	4.89082		455.9677	282.7	5.70329	2.023353
	0.508729	1.930788	4.951915		418.7879	301.5273	5.629354	1.925461
	0.487355	1.852979	5.090811	985.6097	497.8648	475.4118	5.561481	1.841036
67.96778	0.647649	6.780635	478.25	390.4082	1434.75		4.200315	2.920387
81.26334	0.613348	6.240696	467.25	333.75			4.122707	2.771828
	0.604	6.326435	389.2708	333.6607			4.165373	2.74352
	0.606215	6.167875	333.75	378.8514			4.115088	2.684717
109.4193	0.701536	14.19635	1.666257	45.51553	12.77115		6.434131	1.70599
72.6408	0.656054	12.89509	1.71552	35.82843	11.87422		6.129129	1.591791
	0.624685	12.71785	1.649091	33.92873	12.65281		6.015731	1.490227
	0.618415	12.36822	1.613184	32.13273	13.52196		5.496751	1.490164
28.6926	0.550662	5.475951	101.1376	290.4951		782.4	4.523674	2.07825

92.59834	0.51569	5.317714		255.0582			4.324585	1.882639
	0.528912	5.936874	6113.75	284.8019			4.180669	1.947635
	0.510608	6.180999	1383.362	485.0972			4.126612	1.994785
14.73333	0.417281	3.625882	899.8077				4.136662	2.181127
25.4	0.420474	3.454765	311.875				4.201368	2.03142
	0.406712	3.224171	252.54	281.8527	1052.25		4.089647	1.884289
	0.389033	3.084107	772.875	139.8869	441.6429		3.982883	1.612573
3.083333	0.645474	4.739926					4.141628	2.62689
0.75	0.658502	4.568647	46.10839				4.087895	2.33
	0.719824	4.529412	50.8932				4.056202	2.288451
	0.721802	4.670561	56.30024	892.2727			4.16133	2.34027
10.36777	0.810939	8.070054					4.87719	3.723358
8.398889	0.819418	8.380673					4.948525	3.464569
	0.771912	7.881394					4.553519	3.465468
	0.662148	7.899071			2600.625		4.968936	3.580778
7.836671	0.517527	4.873711	3.459532	906.75		138.9774	4.833737	1.550642
4.087224	0.49233	4.570033	3.96052			157.8292	4.749623	1.513525
	0.470634	4.268003	4.018555			182.0893	4.583416	1.495285
	0.460109	3.774536	4.002536	3771.591		1422.429	4.471605	1.450261
22.12227	0.64628	7.26403					5.916709	2.952822
20.74038	0.616801	6.869967		496.784			4.744618	2.679272
	0.605768	6.311968	2.589311	310.8796	1398.958		4.523576	1.903622
	0.595751	6.520786	2.230112	356.75	1114.844		4.683754	1.864698
10.25556	0.571898	12.77284	504.2152	3268.605	1388.148		4.415819	2.746555
10.95556	0.568013	13.36034	1745.625				4.208892	2.77398
	0.537877	12.54512	1138				3.869247	2.386606
	0.510192	10.61511	498.9914				3.588014	2.14436
0.5	0.732972	9.033944	75.19608			163.1	4.451994	5.866633
1.077778	0.775481	9.238963	81.59992			102.7653	4.83051	5.12944
	0.745421	9.078811	78.1745			93	4.65609	4.671763
	0.680955	7.485069	10.66126	1287.083		138.3134	4.362674	2.501528
86.71443	0.564867	13.35815	11745				4.409908	2.181191
67.46527	0.559914	12.90834					4.234492	2.135646
	0.576117	13.97303					4.286838	2.089541
	0.545696	9.75623					4.147512	1.929436
0.166667	0.758365	6.718947	2.875797	953.4534	865.9091	177.2093	5.694017	1.707875
0.166667	0.674411	6.428838	2.834522		668.8889	126.7368	5.444843	1.56552
	0.64031	7.272163	2.947792		1450.625	211	5.228655	1.536717
	0.64291	7.625946	2.924005		1446.875	1736.25	5.143378	1.587399
5.53	0.638237	5.428673	1.74912				3.729161	1.490495
4.036667	0.688394	5.404988	9.604102				3.791826	2.123393
	0.682878	5.228936	15.9445				3.61976	2.244455
	0.676979	5.149725	17.69427		1140.75		3.840435	2.251473
5.28333	0.454037	2.189588	5.076388	41.37082	146.6492	97.01816	3.765853	1.782865
5.199997	0.420874	2.177699	5.432567	48.3037	83.01608	127.6181	3.556226	1.691544
7.583334	0.584235	5.441684	4.744144	1094.25	182.375	267.9796	3.295659	2.107242
12.25	0.55805	5.264144	4.180506	431	253.5294	587.7273	3.124787	1.988912
	0.54766	5.244546	6.403012	520.08	764.8236	371.4857	3.152026	2.112242
	0.541169	5.221553	7.436305	561.375	1684.125	328.6097	3.400533	2.305547
40.20956	0.576577	7.405039	392.4193		7603.125		6.147587	2.618014

50.11667	0.568351	6.947669	598.5		635.8002		6.359831	2.491526
	0.555086	7.033916	351.213		789.2973		6.201355	2.36675
	0.54989	7.231056	212.6749				6.307497	2.322851
11.87383	0.692603	9.572531	43.75791	1320		905.1429	5.602129	3.166253
13.91667	0.698076	10.09356	48.82167	1339.375			5.687056	3.114863
	0.818516	11.66216	48.09887	3066.875			6.564183	3.565743
	0.83306	11.30257	36.49589				6.507601	3.490639
75.62596	0.541914	3.679844	32.08961			5769	4.295798	2.590408
84.38393	0.552821	3.589756	34.50502				4.278487	2.410654
	0.563305	3.776925	37.07032				4.460869	2.414399
	0.547507	3.834563	35.40968				4.540079	2.433135
7.916667	0.706284	9.457504	1508.052		12165		4.162885	2.267374
4.466667	0.751658	8.57965	497.8802		1004.063		4.107783	2.318497
	0.710053	7.998732	466.1138		940		3.845221	2.194476
	0.649922	7.392316	458.5208		1232.924		3.977305	2.13651
52.20889	0.693643	7.16213				86.10638	4.962118	3.988806
83.10111	0.698569	7.115681				80.82	4.878233	3.590616
	0.646227	6.801723				92.17242	4.724134	3.444633
	0.586265	6.810072				158.1	4.579852	3.170267
50.7896	0.518452	5.439633	340.6945			99.44595	3.866823	2.954225
51.46722	0.556507	6.163896	383.1945			89	4.589226	3.52575
	0.545533	5.44783	381.7368			289.3929	4.245093	3.502413
	0.516242	5.387367	322.0379			120.2273	4.144529	2.893629
15.67222	0.728762	7.35039	3.232787			84.16599	5.593155	2.29144
18.38332	0.703359	7.111433	3.100296			176.9302	5.476533	2.163619
	0.688396	7.272144	2.756463			292.92	5.14299	1.991242
	0.695514	7.10117	2.533031			593.5	4.919256	1.861432
24.06	0.534113	8.107801	29.29786				3.372238	2.174932
18.48778	0.528471	8.203007	28.08				3.344115	2.0987
	0.549573	8.03445	26.93424				3.457352	2.125625
	0.546083	8.401967	23.95863	767.803			3.597205	2.102745
36.7582	0.77969	8.094878	155.9351				5.70204	2.358419
36.93611	0.747222	7.168338	907.6881				5.774314	2.295397
	0.720158	7.010852					5.81106	2.214589
	0.705605	7.138072					5.52517	2.027447
39.87666	0.614406	7.207685	4.325489		8493		4.405008	2.131374
69.18333	0.62174	6.19914	4.392294		8349		3.843419	1.975092
	0.589196	5.839629	4.067158				3.786889	1.788604
	0.563023	5.89691	4.054732				3.727779	1.662284
133.0908	0.497435	3.130708	59.94845				4.223702	2.366439
156.3761	0.500422	3.214741	60.10671				4.353361	2.372442
	0.478754	3.196316	75.88274				4.475903	2.265759
	0.470415	3.167109	171.0604				4.46138	2.187036
0.8	0.554935	15.39535	2.806677			1030.875	6.239226	1.980589
0.066667	0.549138	12.98156	2.676137				6.008514	1.79987
	0.520595	12.12339	2.513913				5.596964	1.633462
	0.521906	12.18224	2.455714				5.286013	1.611373
13.92667	0.856325	9.258868	4.974555	214.4181		1119.5	5.214918	2.260326
17.27667	0.883765	8.846456	5.847052	151.9928			5.405891	2.195081
	0.808798	8.057868	5.477373	124.4612			4.980499	1.946994

For peer review only

	0.794561	6.822229	5.761816	115.9545	6168		5.112536	1.913437
1.693336	0.43145	3.365108			801.75	1924.2	4.239102	2.30115
0.166667	0.440261	3.243633			1349.143		4.239342	2.205891
	0.445111	3.334455					4.144969	2.178216
	0.471879	3.589004					4.261924	2.245736
51.73222	0.576639	6.184988	3.023775			42.07538	3.51724	1.968505
41.22	0.539617	5.715035	2.603531			40.92632	3.230765	1.749165
	0.508355	5.485567	2.436014			46.40506	3.073295	1.672035
	0.521499	5.727634	2.35103			71.69444	3.244124	1.691182
5.880004	0.582153	10.28993	1.678207	159.82	636.875	140.8035	8.207201	1.922637
17.49841	0.607532	10.39576	1.40161	169.0512	613.75	194.9559	8.535115	1.720717
	0.537352	8.94771	1.358807	146.5762	807.1875	215.25	7.676734	1.539609
	0.527631	7.217064	1.532813	147.2142	787.1875	198.8684	7.538704	1.533083
27.88333	0.541945	5.959981			340.7806	158.25	5.791465	2.294433
24.75	0.521038	5.670709		1369.583	177.0377	896.4545	5.394695	2.147904
	0.496029	5.156898		445.4717	167.4468	410.6087	4.94568	1.938796
	0.499902	5.141508		696.3043	152.5236	218.3864	4.942223	1.867888
18.96667	0.717685	7.752551	1.911138			92.6639	4.624304	2.629587
29.3	0.671627	7.312414	2.000033			109.5638	4.273964	2.524107
	0.678325	7.080865	1.925284			136.56	4.361206	2.361387
	0.667687	6.541522	1.818772			333.8807	4.27491	2.151245
0.25	0.649626	7.754558	2.632485	93.45426		85.40563	4.636679	1.965116
1.166667	0.636491	6.899283	2.598929	97.88839		717.5455	4.485781	1.635402
	0.613867	6.872207	2.493312	92.28381			4.217351	1.528768
	0.552048	5.859577	2.29529	76.21165		575.75	3.864729	1.436733
16.84167	0.619744	5.283694	3.968584		42.82954		4.556163	1.91197
20.61556	0.618007	4.593385	3.844921		38.13305		4.309933	1.95962
	0.629995	4.476413	3.776745		36.99003	207.7091	4.050259	1.806988
	0.615406	4.496752	3.72522		30.66285	101.4425	3.916868	1.664694
0.25	0.641568	6.431699				186.8864	3.748163	2.623541
6	0.650815	6.209774				765.2778	3.674533	2.620192
	0.616715	5.942931				4355	3.35523	2.425018
	0.614836	6.063321					3.225504	2.385211
11.31667	0.389224	4.56742	619.95				3.175559	2.051065
20.12223	0.359271	4.092073	474.5238				3.121342	1.820633
	0.372188	4.113628	397.5938				3.252682	1.808515
	0.365642	3.934871	560.087				3.26249	1.729719
15.68556	0.718005	10.18711				825.2143	5.316111	2.802663
15.23333	0.723283	9.943505				677.1177	5.442354	2.746763
	0.700456	10.23421				943.75	5.268177	2.675981
	0.677232	9.847913				2257.2	5.062078	2.649972

For peer review only

ratio_bed_:	ratio_bed_:	ratio_bed_	ratio_bed_	ratio_bed_:	ratio_bed_:	ratio_bed_:	ratio_nurse	ratio_nurse
6.967948	5.175058	40.94118	0.987274	15.76168	7.098082	15.0951	1.236702	0.100572
6.748109	4.709292	274.9688	0.936631	14.14716	6.806367	13.65033	1.287941	0.106969
9.693797	4.628045	1759.8	0.958469	13.52086		12.42867	1.31092	0.11145
15.7453	4.299388		0.861746	10.39807		12.0043	1.423444	0.116921
2.40911	2.43407	426.6923	0.789121	13.69294	5.591869	6.012407	2.240854	0.33521
2.314504	2.329161	3249.857	0.768746	12.3439		6.017748	2.216561	0.331913
2.139977	2.084342		0.71007	10.78592		5.020691	2.359724	0.369582
12.17429	2.281571	1090.8	0.801709	11.73036		13.10993	1.87902	0.152997
6.09822	2.130808		0.716791	6.726713	4.74206	8.470817	1.633386	0.093625
6.139795	2.08023		0.684403	6.318039	4.657796	7.437956	1.750152	0.110415
5.637983	2.136775	2180.482	0.661732	6.088388		8.098723	1.637351	0.120862
4.992101	2.099335	24.45011	0.598801	6.137976		7.275791	1.626658	0.1305
3.156177	3.943144	1684.385	1.171099	15.59497	6.289596	11.04123	1.880572	0.201832
2.882222	3.935171	4035.6	1.111096	16.24846	6.185536	12.01115	2.181718	0.23296
2.850664	3.670663		1.119172	16.13986		10.83674	2.22162	0.234409
2.926253	3.285862		1.111767	16.07328		13.22991	2.230028	0.218388
14.51462	3.750716	49.01123	1.051658	9.688479	18.12366	12.31957	1.826381	0.078598
14.19535	4.261879	36.18646	1.182596	11.03463	22.22505	11.9341	1.610514	0.063011
16.74063	4.285947	33.54138	1.192956	11.58792		12.67187	1.53303	0.060223
19.28483	4.095509	31.3248	1.141046	11.56322		13.21921	1.724255	0.065978
2.729025	3.71543		1.396807	29.57085	6.411441	18.83871	2.018743	0.09635
2.333924	3.206687		1.158571	20.15816	5.572321	16.01277	2.330849	0.111061
2.361031	3.130921		1.068698	14.91027		13.1094	2.213643	0.115714
2.315803	2.79863		0.979679	10.40618		11.65484	2.337974	0.113849
7.96029	6.471368		1.369963	14.09809	12.32815	50.89233	1.203049	0.021038
7.306677	5.15944		1.086692	12.62197	12.33616	38.36164	1.255235	0.022014
6.956836	6.037163		0.994707	12.57387		30.79673	1.306325	0.025884
7.692865	6.326064		1.06235	12.22339		24.78691	1.280642	0.027197
25.70779	4.819793		1.063004	21.02021	10.52861	14.91474	1.281673	0.051282
22.76243	4.801032		0.91952	15.98032	10.70581	13.25384	1.284698	0.051914
19.5537	4.511707		0.956499	13.34534		9.068523	1.40071	0.063291
18.83453	4.985013		0.994314	14.10208		9.475832	1.50221	0.060186
3.037148	2.225309		0.6743	11.69165	6.482439	6.622304	1.775427	0.878617
3.008724	2.066253	3142	0.684692	12.60576	5.894393	6.512037	1.800837	0.899775
3.046527	1.983497	776.25	0.671099	13.14885		6.098453	1.842441	0.927907
2.994794	1.967122	868.3637	0.672469	13.30077		6.503622	1.870457	0.941777
3.763919	1.150379		0.447778	4.42267	3.488998	1.80503	2.389215	0.117574
3.573754	0.95584		0.396237	3.320086	11.83294	1.21532	2.473765	0.143916
3.686555	0.95133		0.404523	2.978837		1.183173	2.300385	0.186803
3.704464	0.947465		0.399281	3.020874		1.198356	2.27594	0.317584
7.189172	3.274344	270.2804	0.999748	10.23965	14.79978	15.47807	1.851675	0.264363
7.327323	3.196836	607.3729	1.001254	9.746354	15.97638	12.80492	1.771713	0.256034
8.538699	3.457185	13640	1.070067	10.32466		11.95419	1.552881	0.225789
7.037419	2.950116		0.864717	8.81135		8.524881	1.804029	0.252615
5.873455	4.210043		0.790151	6.696024	25.66403	6.139123	1.769496	0.131287
5.745118	4.047297		0.768736	6.623	25.9808	6.030728	1.742896	0.133068
5.462045	3.842849		0.737491	6.325999		5.752342	1.759227	0.123688
4.776813	3.498498	95.56604	0.60255	6.307911		4.578185	2.244978	0.131883

For peer review only

4.184002	2.971093	105.6851	1.013555	12.42237	5.821737	8.153485	2.201924	0.153501
5.190228	2.959804	334.8559	0.976587	11.94193	7.080567	7.64122	2.188539	0.148287
6.348771	2.950326		0.949893	12.06797		7.747544	2.118569	0.146538
5.265637	2.709979		0.856221	11.56993		8.608749	2.309245	0.155829
8.222031	4.724625	152.9167	1.192306	25.64459	10.75463	22.9375	1.666982	0.241756
8.216424	4.476744	306.3158	1.155641	28.27827	11.82942	29.69388	1.642457	0.217899
8.048386	4.221401		1.081482	29.84415		20.25	1.620593	0.207547
9.254688	4.491301		1.133834	21.9257		17.38597	1.588919	0.186122
8.923929	6.189874		1.415033	12.06115	7.859444	17.53957	1.339663	0.133754
29.32783	5.789671		1.347718	11.08197	12.43428	25.00901	1.371068	0.137828
21.35785	6.104502	5436	1.188025	11.19471		22.4091	1.469558	0.14986
18.58911	5.533126		0.991937	11.98795		14.65457	1.562371	0.162495
8.511473	4.443583	17.5601	1.265022	12.0179	13.80724	17.10009	1.457129	0.207067
7.863821	4.924989	19.12213	1.301908	13.11912	11.17776	18.17462	1.49417	0.214666
6.959762	4.466687	31.05195	1.162565	12.2094		16.33581	1.732391	0.21903
6.81067	4.19334	59.34147	1.172448	13.05542		12.37755	1.902828	0.220679
7.873411	4.439564		0.623888	12.41244	23.31765	13.07791	1.876969	0.170675
8.130658	4.2532		0.597015	10.19124	22.2412	7.263921	1.782342	0.166817
8.144128	4.145222		0.65878	10.29649		8.58344	1.691024	0.157678
8.750893	4.392026		0.670037	10.68604		8.617222	1.722053	0.148598
4.363432	3.598046	64.58182	0.658442	10.11854	7.205973	25.08332	1.719753	0.141491
4.08956	3.606524	53.18182	0.612899	9.894714	7.273326	12.50439	1.741099	0.132996
4.127804	3.642707	271.6154	0.59624	9.213192		12.77891	1.737844	0.131947
4.240098	3.774638	23.36404	0.580787	8.42215		13.58272	1.756302	0.126722
6.053994	4.289642	329.2729	0.769105	8.17581	7.42899	8.763494	1.289293	0.146647
5.648192	3.817767	164.3438	0.780224	8.014617	7.556328	9.190624	1.367789	0.172421
6.312995	3.708074	286.8333	0.801492	7.937013		9.052389	1.418883	0.177537
7.167575	3.719095	1057.8	0.860511	8.569855		10.25437	1.349875	0.192057
5.496731	3.561681		0.795926	13.01709	5.568854	14.13713	1.888728	0.146886
5.833725	3.625059		0.810377	14.03511	5.711174	14.54513	1.711901	0.157067
6.049282	3.315509		0.814423	14.77907		13.19686	1.626543	0.160737
6.11375	3.262322		0.785691	14.1878		11.03785	1.676113	0.169581
3.846828	3.787031		0.966337	12.42859	4.583607	10.98394	1.668841	0.136843
3.913672	3.637924		0.945937	11.0942	4.663537	9.999263	1.695001	0.131896
3.86606	3.309579		0.939567	10.95995		8.885921	1.760773	0.143018
3.915037	3.142605		0.915567	10.64354		8.750899	1.811739	0.143794
5.639182	4.999734		0.849448	13.14623	6.178235	8.7486	1.37765	0.120227
5.397698	3.809452		0.816039	8.550222	5.890987	8.179292	1.393497	0.13388
5.550282	3.577275		0.822304	8.081924		8.02597	1.311271	0.131536
5.801372	3.952761		0.796085	7.577853		8.769449	1.360006	0.123211
4.491946	2.768246	79.60465	0.517907	2.658027	8.501109	4.783871	1.81446	0.157417
4.25039	2.549237	647.4	0.533675	3.050918	8.212262	4.694201	1.681303	0.166377
4.07007	2.228697	603.6	0.466679	2.728265		3.713117	1.706731	0.189587
4.226841	2.294381	1569	0.449591	2.431399		3.494828	1.702997	0.184356
25.87786	4.351614		1.176834	10.14538	9.581922	14.37238	1.594672	0.134338
28.59431	4.322944		1.07264	9.234871	10.62519	13.82068	1.543953	0.13888
26.21176	4.407696		1.116468	9.467297		12.39405	1.50937	0.119977
26.39897	4.462047		1.003495	9.131846		10.67422	1.549742	0.118558
6.741646	6.044226		1.091598	18.66366	11.80658	24.81713	1.352671	0.280729
6.558155	5.616777		1.098685	19.57168	11.03767	24.83682	1.344396	0.284275

6.657758	5.178217		1.084361	19.41904		17.78468	1.408711	0.288004
6.287349	4.681919		0.991889	18.57232		15.75606	1.577657	0.289431
5.963695	5.106553		0.900622	11.68542	7.810595	6.699798	1.282672	0.076299
5.602436	4.831621		0.895119	10.84773	7.033542	6.056661	1.319466	0.080977
5.477144	4.610012		0.874198	11.81418		6.564535	1.283189	0.077973
4.945283	4.36486		0.867082	11.89322		6.16922	1.299467	0.080059
5.458183	2.348877		0.502812	5.773232	7.230303	14.50465	1.786483	0.114191
5.468032	2.373243		0.501856	5.657679	7.317809	16.47734	1.766294	0.113131
5.189003	2.419142		0.453376	5.513168		20.91549	1.881101	0.10802
5.39838	2.454811		0.431636	5.37528		17.59515	1.873811	0.111695
5.240892	5.379158	95.11364	1.051007	7.050743	6.59827	18.42855	1.322612	0.121438
3.61343	5.327791	135.1935	0.947327	6.650134	3.736449	14.91954	1.370656	0.128036
2.816902	4.566386	340.3637	0.832714	6.310369		11.62781	1.510112	0.145374
2.874457	4.411937		0.827446	6.527067		9.071451	1.335416	0.142709
16.67197	5.202838		1.192551	9.650794	17.69761	32.60369	1.323349	0.131583
17.80058	5.248878		1.173415	9.630737	20.25027	28.72132	1.250617	0.126332
18.37919	4.787358		1.149648	9.947768		23.3928	1.241267	0.129818
18.34822	4.754258		1.017109	10.02349		26.96379	1.319476	0.126703
4.524842	5.168393	1100.571	0.903863	12.38923	9.453445	11.87179	1.529002	0.123721
4.280816	4.529089	1473.6	0.877017	10.52439	7.406241	6.818687	1.570699	0.137038
4.460789	4.511565	7620	0.866857	8.240029		5.354106	1.492497	0.125026
4.650457	4.691966	7716	0.836451	7.60129		5.156892	1.540285	0.124464
5.363285	4.041989		1.063676	14.2656	7.396955	6.541423	1.395747	0.10085
5.120288	3.641238	366.0551	0.957947	13.1337	6.87597	6.319041	1.478587	0.11094
5.163794	3.581687	164.9092	0.95112	9.769488		6.669864	1.373601	0.12025
5.523847	3.664211	23.6115	0.966601	8.654012		6.623389	1.376667	0.120493
6.602001	6.148575		1.140012	7.542548	6.932499	20.86044	1.232791	0.178008
7.037178	6.015294		1.180403	6.831376	6.997563	18.08092	1.272153	0.164655
7.118974	5.666008		1.170078	7.300581		17.12677	1.279902	0.162309
7.305412	5.685731		1.177494	7.781002		17.58364	1.258385	0.16042
13.06393	6.377124	25.12779	0.946646	11.7358	18.42849	5.864822	1.483253	0.133309
14.64195	6.714107	21.46073	0.961195	12.57499	17.69802	6.362154	1.43016	0.132248
15.30032	6.166518	17.49705	0.975668	10.54722		7.903117	1.459558	0.139599
13.02837	6.627266	11.77961	0.94544	13.74674		9.784301	1.57403	0.136382
9.394515	5.009253	135.2571	1.218108	10.88354	61.06809	8.722409	1.457512	0.165243
3.914485	5.051521	28.50495	1.195159	10.66896	5.304418	8.66287	1.43872	0.172649
4.609754	4.343836	67.97387	1.069675	9.124714		5.756191	1.513177	0.124719
4.703027	4.180946	212.4951	1.022226	8.415439		5.243296	1.687487	0.127683
8.783055	5.928404	47.83344	1.075795	17.68883	6.793519	11.95571	1.391053	0.10317
8.361976	5.809921	63.17641	1.026955	17.29257	6.886618	11.22681	1.435729	0.108212
7.591409	5.555398	124.9596	0.990847	15.0555		10.93766	1.487775	0.109142
7.730395	5.627641	135.9909	0.990345	15.01904		11.47937	1.628346	0.115017
7.179627	5.437749		1.064318	16.56668	9.235357	7.664541	1.615153	0.001242
6.906942	5.005474		1.045699	16.1633	9.644717	6.7525	1.609437	0.005591
7.118903	4.856789		1.006363	15.65049		6.22135	1.612169	0.001798
7.605613	4.611992	57.79856	0.971298	15.96476		6.529555	1.646355	0.001618
19.10696	2.031748	124.6529	0.750597	10.78249	18.46699	11.11905	1.313752	0.150544
19.12337	1.857964	258.6516	0.737433	12.08107	18.11831	9.069128	1.321904	0.13446
18.96281	1.851257		0.750226	11.33808		8.646067	1.353571	0.148642
4.59539	4.194031		1.146112	8.974551	7.720722	7.824031	1.449594	0.151501

For peer review only

4.341147	4.025207		1.149334	8.234481	7.249176	7.457546	1.445169	0.148596
4.229981	3.73183		1.117212	7.591545		7.375162	1.478982	0.146041
4.324264	3.790072	697.1476	1.074789	6.941168		5.364965	1.547357	0.147561
12.8871	3.697964		1.513437	13.5708	25.73774	10.22134	1.380854	0
12.98072	3.872743	119.7805	1.420775	13.34233	27.1486	10.29509	1.367501	0
13.72886	3.897025	863	1.261869	14.00065		10.98174	1.339981	0
5.81992	3.326577		1.039446	12.57984		8.438207	1.662578	0.001943
5.30355	5.613491	32.9292	1.281721	14.44037	7.819473	7.988863	1.601489	0.151711
4.635604	5.196316	19.28415	1.193014	13.30346	6.679169	7.639322	1.670969	0.155035
4.346565	5.351667	24.13225	1.14606	12.1723		7.041472	1.70898	0.158861
4.070315	5.294696	31.1497	1.100918	12.1173		6.72909	1.712952	0.168933
4.731348	3.365065	130.9943	0.986723	11.03374	5.549634	9.081419	1.687038	0.187624
4.845346	3.48032	254.5313	0.978961	10.35578	5.587618	8.018485	1.623081	0.183548
4.708942	3.224321	251.0938	0.864218	8.190411		6.202716	1.733997	0.195875
4.600646	3.158737	513.75	0.820784	7.721268		6.087987	1.718218	0.185031
5.583072	3.349786	274.7746	0.818134	9.459708	4.786096	16.81755	1.427444	0.142539
5.440028	3.18519	959.6619	0.800805	8.826913	4.593909	16.02069	1.402121	0.145885
5.17275	3.103776		0.773286	8.052696		14.18798	1.444728	0.160419
4.568668	3.051593	11294.92	0.715609	7.88465		11.8312	1.51451	0.193781
5.502782	3.240245		0.790624	8.559638	5.955031	11.00163	1.525181	0.147736
5.222604	2.588273		0.687505	8.118581	5.554212	10.27477	1.557223	0.160924
7.513783	2.539304		0.588951	6.906887		9.095475	1.703929	0.172795
13.98703	2.564796	5499	0.681828	7.554194		10.58294	1.615417	0.161164
5.612954	3.968184	2286	1.034312	10.40264	8.149374	6.792977	1.780333	0.113703
5.635905	3.764789		0.914799	9.8423	9.285127	7.214932	1.82423	0.085068
5.41938	3.804825	11711.25	0.862462	9.939694		7.613864	1.802414	0.08608
5.910839	4.011987		0.901353	12.32802		7.972599	1.726266	0.15174
15.21447	4.401213	50.91177	1.193289	11.72811	21.57758	9.887663	1.505781	0.127356
15.34611	4.144682	50.79612	1.123936	11.40451	22.17327	9.874432	1.524328	0.126594
15.78181	3.87929	123.2701	0.978143	10.22821		9.479554	1.52317	0.126167
17.3231	3.946263	5448	0.895855	9.918537		8.358222	1.462788	0.123132
10.64313	3.540753	100.1278	1.226608	7.37781	59.27367	4.154265	1.471039	0.065735
10.53087	3.940228	114.9297	1.162538	7.745852	62.70316	3.978205	1.430846	0.06151
10.05002	4.123122	114.7059	1.152792	9.594302		3.966362	1.402424	0.055398
13.37272	3.399266		0.954502	7.930777	20.43839	15.29693	1.857676	0.113592
11.31999	2.976983		0.903625	6.915917	18.24391	8.486072	1.780234	0.124568
9.208871	2.352958		0.756658	6.045717		6.363691	1.770301	0.126212
8.870901	2.2395		0.672673	5.601156		5.652831	1.939342	0.139758
1.829533	3.434014		1.156235	11.6956	2.390856	21.1835	2.160492	0.318412
2.043362	3.457077		1.090212	11.16759	3.08415	20.41028	2.110904	0.322576
2.077947	3.354592		0.925586	10.541		18.02637	2.273297	0.327551
2.04145	3.136709		0.866941	9.97075		13.15455	2.53112	0.349745
5.021985	5.745559		0.75489	9.526201	9.354451	8.920864	1.694732	0.009686
4.744027	4.970892		0.647519	6.661468	8.486562	8.429593	1.680554	0.010934
4.358221	4.551204		0.605376	6.170803		7.651153	1.737007	0.017907
4.164323	4.435468		0.612479	5.118865		7.998253	1.848505	0.062399
4.018324	3.520576		0.823165	11.8499	6.871449	12.96066	2.091994	0.372442
3.897067	3.34697		0.727814	10.98298	6.773048	12.81555	2.147449	0.394137
3.700646	3.103281		0.681531	13.13463		11.84835	2.150063	0.406232
3.350554	2.902026		0.615047	12.23636		10.74065	2.26992	0.417142

For peer review only

3.062236	2.486877	210.4388	0.899948	7.477576	5.806337	12.14445	1.8667	0.07284
2.986764	2.428122	618.1169	0.857263	7.411092	5.799213	11.81502	1.878733	0.074272
2.715552	2.246184	452.25	0.825095	6.954251		11.25375	1.950766	0.075499
2.59133	2.163777	55.53551	0.749667	6.556603		9.529713	2.023318	0.071413
4.122399	3.534344	4739.063	1.201075	9.341024	4.737308	7.544395	1.744652	0.135385
4.105893	3.488401	1015.346	1.108842	8.5849	4.87001	7.891885	1.693741	0.135279
4.110892	3.528762	15669.21	1.050365	8.909166		8.030193	1.655311	0.133154
4.235119	3.533029		0.994449	8.809599		7.60003	1.722469	0.164302
4.310269	6.352666		1.201264	13.2575	6.979234	13.48235	1.827567	0.156025
4.77659	5.853467		1.11618	14.65333	7.286686	13.27686	1.824191	0.158483
4.36122	4.763111		1.054843	14.79343		11.95052	1.949103	0.168257
4.43991	4.213277		0.964511	13.24201		11.70474	2.057846	0.16779
2.823233	1.455839	103.165	1.09017	11.91197	18.68193	7.992352	3.087978	0.605948
2.753001	1.411395	80.47609	0.989246	10.65003	21.69967	7.325037	3.054906	0.610373
2.592473	1.328604	98.125	1.058819	9.174294		6.959301	3.188064	0.665527
2.464449	1.191713	133.1379	0.884774	8.525537		6.164354	3.330065	0.711633
6.406053	5.947518	36.08	1.208266	12.63374	5.897214	18.55713	1.473701	0.192808
6.479013	5.330514	121.0449	1.19551	11.62727	5.898122	17.17196	1.429389	0.190966
6.234676	4.843468	512.8571	1.11696	10.96916		18.15658	1.460649	0.1903
6.34671	4.126547	388.1786	1.017225	9.973099		21.09175	1.58493	0.195719
20.06382	4.983375	34.22807	1.254374	8.992638	22.86352	23.85733	1.332934	0.162032
20.21928	4.961319	65.36667	1.202588	9.127826	22.10237	20.40536	1.297544	0.170441
18.43187	4.77804	303.7895	1.102396	9.878261		19.22036	1.284311	0.176059
17.4782	4.320713	2880	1.017868	9.487832		16.63362	1.315193	0.185836
2.606745	3.18166		1.298126	12.72988	3.531306	7.66533	2.319682	0.201979
2.560817	2.964051		1.317227	13.99661	3.229211	7.228746	2.235024	0.207662
2.301652	2.439117		1.304642	11.99248		6.368924	2.387973	0.225943
2.187644	2.345149		1.256055	12.58731		5.558951	2.397579	0.248701
27.73695	4.552782	46.00478	0.827896	7.753495	61.12212	15.86517	1.275989	0.073824
30.67012	4.221672	438.6364	0.794094	7.429322	67.21631	14.22791	1.240397	0.072578
26.39803	3.858296	2769	0.696116	6.740998		10.41583	1.263161	0.088409
24.19916	3.799799		0.650347	5.682502		9.050935	1.287312	0.087073
4.853503	4.355447		0.765637	8.731917	5.044199	18.22307	1.814157	0.126171
5.031582	4.118622		0.793191	8.535118	5.109807	17.70742	1.753043	0.125959
4.97144	3.911057		0.79611	8.23538		16.56918	1.75129	0.131249
4.925628	3.735196		0.816425	7.886509		16.85828	1.763036	0.135899
18.85143	4.561433	39.48498	1.020603	7.481977	25	16.46975	1.497055	0.103845
14.92011	3.514499	32.24461	0.815595	6.149934	22.17934	12.36755	1.758384	0.13212
12.58376	2.974597	31.25806	0.696739	5.429675		10.96154	2.036126	0.15484
5.196725	4.385947	669.25	0.900585	6.62917	5.982848	18.10839	1.507882	0.174649
5.245748	4.321269	1582.2	0.92982	6.286687	5.862233	15.03127	1.53743	0.172707
5.503309	4.350971	8268	0.908655	5.889699		15.85641	1.46512	0.169429
5.534564	4.41998		0.873563	5.483686		15.32644	1.485392	0.179081
5.151814	4.917843		0.867923	6.979927	5.092054	13.15513	1.261334	0.077028
4.838931	4.543519		0.932639	10.40976	5.210432	13.25705	1.282931	0.07759
5.12038	4.435383		0.926738	10.43279		13.75095	1.290083	0.082956
5.187241	4.730841		0.918467	10.19408		15.49529	1.23228	0.083563
6.510273	3.945981		0.796986	28.38299	11.1466	6.26739	1.558448	0.131902
6.406185	3.903508		0.808944	30.04463	11.03408	6.079555	1.439932	0.118814
6.001607	3.471015		0.755435	31.62369		6.139636	1.492237	0.125941

For peer review only

5.822385	3.075445		0.629861	32.64249		7.354724	1.603821	0.136505
4.975081	2.919864	20.3	1.305281	18.45807	14.60589	10.53995	2.156874	0.235411
5.81517	2.914871	14.28631	1.237472	18.84466	15.69003	10.68911	2.19822	0.229952
5.808347	2.819342	18.3301	1.18044	17.04579		11.44366	2.409986	0.269111
5.750837	2.835074	35.19	1.191436	15.92361		12.93845	2.333885	0.249522
5.451974	4.351244		0.912256	13.33605	7.680209	7.785502	1.538951	0.10242
5.198862	4.075168		0.790119	13.74843	7.151463	6.644766	1.664792	0.125324
5.224807	4.084492		0.810611	14.87566		6.028732	1.60777	0.121957
4.790982	4.114675		0.777952	15.19822		5.421182	1.692922	0.126464
3.69274	3.899925	194.5962	0.969896	10.27776	4.09986	15.43107	1.566013	0.157395
3.627801	3.815718	676.8	0.887402	8.112487	4.134996	14.73176	1.619385	0.16256
3.486982	3.476566		0.831458	10.41781		12.78399	1.67031	0.164596
3.445509	3.183873		0.761627	10.44826		11.80364	1.769954	0.173462
3.713296	5.09169	44.88734	1.140902	9.339329	4.097027	15.18994	1.501661	0.15051
3.502533	4.618979	294.2153	1.069742	8.822692	3.761075	14.15041	1.561741	0.153995
3.456211	4.597989	1032.757	1.044895	8.904718		14.12605	1.62822	0.160513
3.45105	4.480303	313.4369	1.008355	9.341628		15.87337	1.708046	0.162355
5.787491	1.400814	26.19122	0.816208	7.706853	3.055247	7.853653	1.861059	0.379561
6.059923	1.440976	30.13158	0.822744	7.206192	3.115214	5.128745	1.705986	0.354775
6.474347	1.530073	75.54289	0.78555	7.100933		4.116887	1.722278	0.324658
6.306507	1.28382	105.5962	0.697071	6.612245		3.653595	1.846516	0.346493
3.762694	4.248958	53.05854	0.855968	6.609228	5.647921	22.20123	1.625096	0.132888
3.514312	3.995876	369.75	0.835094	5.987475	5.611249	20.50046	1.698374	0.138543
2.55872	3.953705	1496.143	0.802886	5.628085		21.00159	1.703676	0.152966
1.975958	4.02154	145.2651	0.735449	5.450732		21.62735	1.770884	0.163172
8.373023	3.063333	405	0.89658	10.04188	12.97371	25.44553	2.026442	0.159001
9.505353	2.928991	297	0.877386	10.29641	10.7876	23.99238	2.067581	0.163357
9.727121	2.877397	382.1786	0.847083	11.17653		19.48039	2.078638	0.169239
4.217331	4.224134		0.557163	4.884147	7.45968	5.077664	1.984077	0.169069
4.149971	3.576286		0.517387	4.187985	6.650352	5.05916	2.074115	0.17989
4.414973	3.153876		0.516718	3.78485		5.158392	2.035529	0.182908
3.990941	2.803868		0.48341	3.695329		4.709799	2.226205	0.183286
27.81825	3.356913	29.72269	1.098015	11.10239	27.97375	10.49306	1.528694	0.106129
33.9816	3.325109	32.19444	1.116317	11.11028	27.55151	5.852483	1.581435	0.114762
30.19597	3.21243	519.4286	1.13559	11.80008		5.921051	1.568412	0.114954
29.35802	3.091543	625	1.114018	11.3293		6.345463	1.594758	0.112096
4.25869	4.045131		1.448539	10.09108	5.341221	7.632582	1.474447	0.126889
3.925012	3.65115		1.282812	8.747045	4.737693	8.199172	1.654315	0.150428
3.953394	3.455302		1.215802	8.780649		8.093018	1.711025	0.148751
4.070753	3.372741		1.070867	8.30539		8.642617	1.728509	0.152028
3.608908	3.325989	137.2	0.864729	14.37838	7.829567	22.41498	2.13025	0.143659
3.519874	3.06851	234.3529	0.81528	13.45505	7.101611	20.08464	2.212612	0.156242
3.712617	2.254295	131.7245	0.672727	8.966386	5.015689	5.98065	2.031552	0.034935
3.806452	1.9142	421	0.635839	7.326201	4.765866	5.602631	1.931335	0.038496
3.701957	1.83752	3020	0.621406	6.695849		5.575405	1.944211	0.034237
3.428636	1.759299		0.558198	5.123273		5.601611	2.049609	0.043675
6.03873	4.065566	33.27	0.806061	8.04804	6.800508	6.786248	1.609809	0.229811
5.67007	4.029582	54.39344	0.718277	8.496464	7.196198	6.145853	1.672257	0.235782
5.738689	4.092221	134.4375	0.675297	8.623918		6.266522	1.807887	0.259452
5.41369	3.92552	917.5714	0.632823	8.063874		6.030773	1.869348	0.309378

8.356161	9.275374		0.922363	17.01002	11.13643	7.27943	1.27507	0.08825
8.848314	8.920867		0.733949	13.29641	10.28168	6.338444	1.415125	0.093807
8.104072	7.51439		0.679688	12.17571		5.941965	1.483864	0.104608
7.877734	5.554828		0.647638	10.32973		5.526068	1.479647	0.11419
6.796874	4.87859	255.1128	0.916358	12.97476	5.872107	29.43268	1.502361	0.077933
7.34055	5.561182	427.6744	0.86774	6.864883	6.039609	17.49429	1.431279	0.068476
7.224618	5.609764	615	1.082611	13.77313		11.46627	1.420746	0.070788
7.071745	5.939016	679.7368	1.027908	12.63608		10.82605	1.465002	0.081745
5.109602	4.980195		0.940273	8.098756	6.514539	8.843879	1.507091	0.150765
5.062958	4.497256		0.844032	6.965152	5.836115	7.814544	1.585996	0.154891
5.080677	4.358691		0.838697	7.147655		7.728462	1.612352	0.150936
4.945723	4.318598		0.766339	7.192739		7.433009	1.701933	0.145679
10.76471	5.949392		1.156965	16.68411	14.16181	8.929895	1.327374	0.145844
9.762952	5.279429		1.059694	16.7924	13.0018	8.253143	1.378186	0.152147
6.76277	5.026674		1.062007	13.89544		7.951298	1.40735	0.152296
6.642844	4.911842		1.052954	13.38695		7.85329	1.445465	0.157641
6.020372	6.294289		1.326201	8.977421	7.93245	24.04455	1.599179	0.09899
6.530737	5.898778		1.240939	8.674485	6.622439	20.36118	1.624474	0.108828
6.030669	5.562565		1.197913	8.31035		18.05128	1.587536	0.106428
6.244279	5.712659		1.229976	8.674428		19.44008	1.48339	0.103557
5.409681	1.519092	547.7143	0.49304	3.259403		2.960039	1.901167	0.295589
6.925598	7.084744		0.954199	24.44529	7.533594	20.86925	1.315732	0.081329
6.818538	6.675748		0.930654	22.62291	7.225057	19.94661	1.337747	0.076134
6.126936	6.08002		0.985055	20.27518		19.08665	1.385364	0.089972
5.96855	5.220232		0.915602	15.57264		18.20009	1.530701	0.112334
6.528609	5.304051	154.0692	1.226488	12.61142	8.345587	23.03921	1.31698	0.136278
6.739564	5.234921	266.2059	1.319486	12.16532	8.692308	17.04082	1.29853	0.139502
6.838449	5.009357		1.275637	11.50329		12.13078	1.368267	0.142671
6.437688	4.997308		1.17754	10.10793		10.85241	1.395273	0.148642
3.589879	5.442508		0.977645	9.492982	7.343757	19.18988	1.339706	0.05027
3.662867	5.220455		1.050016	8.189776	7.88415	17.15652	1.251864	0.052769
3.593767	4.951354		1.022418	9.302704		14.26421	1.254176	0.057414
3.569598	4.838892		0.984779	8.265443		13.81441	1.418028	0.071326
6.48915	4.705114	102.3555	0.995641	7.198215	5.519452	4.825114	1.582521	0.133214
6.303613	4.345113	287.0658	0.919718	6.792929	5.289258	4.627218	1.642384	0.144843
6.121777	4.302073	839.4445	0.878826	7.064869		4.30045	1.661195	0.143835
6.598218	4.369491		0.912631	6.800443		4.589459	1.632866	0.131151
6.199915	5.005858	27.94366	0.894106	17.44541	6.971636	9.372639	1.548218	0.155762
6.055619	5.224158	26.78049	0.789142	13.28783	6.923408	7.76023	1.732325	0.173291
10.46641	5.165045	47.08696	0.894754	10.51797		8.571654	1.839849	0.15404
10.74966	4.510761	107.0204	0.866302	9.212177		8.25844	2.032295	0.287507
2.996474	3.250414		1.247009	13.47608	4.368729	8.807537	1.996647	0.000382
2.916679	2.830074		1.09079	12.06047	4.070836	8.337218	1.975584	0.0008
2.953073	2.717827		1.03335	11.92562		8.064009	1.971083	0.003076
2.931518	2.606879		1.014297	11.67379		7.866998	1.949969	0.004881
5.32106	4.220505		0.93325	20.62578	6.8197	9.818746	1.995981	0.135137
5.267971	4.043872		0.958364	21.78642	7.635145	8.95328	1.873132	0.128298

For peer review only

5.197113	3.638509		0.950101	19.18455		8.580819	1.784168	0.134704
4.997576	3.487447		0.820588	15.45055		7.23242	1.989025	0.14194
14.54492	3.798418		1.325209	17.15992	17.05202	12.57284	1.606528	0.348891
19.07714	3.760947		1.334708	20.64539	20.94432	13.721	1.588928	0.330629
17.49181	3.745355		1.272583	21.69327		13.13435	1.802316	0.349533
18.5697	3.305297		1.264922	22.27114		13.01381	1.98123	0.369415
9.614999	3.122327		0.921317	9.187798	33.00972	11.73591	1.947621	0
6.537767	7.264832		1.284996	16.12736	9.288705	11.06912	1.126401	0.13003
6.603492	6.41674		1.362419	14.69727	9.335554	12.03027	1.08036	0.126243
5.629881	4.322928		1.008042	11.86342		19.86667	1.266116	0.154002
5.72051	4.129589		0.970875	11.79706		19.12801	1.440067	0.163464
3.688668	3.219209	35.29907	0.880748	7.25544	3.528522	4.282896	1.818044	0.36338
3.492968	2.793188	43.17722	0.786879	7.81524	3.105803	3.776238	2.039747	0.403576
2.911476	2.015687	23.64103	0.637445	7.543055		2.561095	2.628557	0.525603
2.57821	1.532541	57.06667	0.59869	7.056335		2.191575	3.025183	0.645113
5.597557	3.989703	60.04445	0.924393	9.951586	6.356588	18.54581	1.612805	0.146887
6.210929	4.311796	71.4875	0.951624	9.805213	6.284644	22.5336	1.503069	0.135316
3.322412	5.591696		1.123046	8.863357	4.692243	23.2698	1.396221	0.063882
3.28757	5.010788		1.03441	8.041708	4.342602	18.99869	1.411129	0.069178
3.134267	4.493797		1.014616	6.732204		18.77595	1.440191	0.087264
2.880467	4.517262		0.919344	6.044764		17.54802	1.516267	0.089448
2.596644	2.469862		1.00201	9.880343	5.20322	8.677729	1.589297	0.322336
2.703899	2.289578		0.936504	9.403351	5.318536	8.844749	1.567617	0.31117
2.659734	2.244614		0.890835	9.03526		8.033144	1.592321	0.324386
2.605222	2.087902		0.837492	8.421832		6.378568	1.638943	0.343361
14.28925	4.422292		0.543802	9.907087	17.85951	13.98455	1.922536	0.08154
13.71218	3.893088		0.523275	9.91198	15.51225	12.93721	1.950119	0.086714
13.70491	3.860071		0.52111	10.98648		12.01514	1.924395	0.08753
12.53664	3.364667	480	0.467062	6.656232		9.076684	1.954061	0.172703
3.165866	4.426432		1.189663	12.01652	6.864732	20.86915	1.930032	0
2.927237	3.466745	855.1875	1.073235	10.16803	5.704798	17.50996	2.108328	0.013876
4.125388	3.803149	224.413	1.002066	10.1441		19.72802	1.698616	0.070893
4.022487	3.595625	1932	0.939219	9.092567		19.28335	1.711455	0.072962
3.763587	2.08421	149.6866	1.316335	35.9102	6.586069	12.28523	2.543697	0.188184
3.545683	2.009151	119.775	1.193385	33.23698	5.513192	9.131056	2.628151	0.185364
3.294123	1.887128	78.30252	1.066431	25.92414		7.442976	2.814889	0.204661
3.260097	1.763485	138.6177	0.986016	22.33473		7.719316	2.941221	0.212352
2.580272	2.839042	52.09816	0.888528	10.7484	5.376861	6.874467	1.771071	0.460082
2.446353	2.640818	96.50294	0.853414	10.2422	5.006647	6.756238	1.907345	0.49695
2.311868	2.483987	186.7848	0.828024	9.093325		6.406632	1.965685	0.517923
2.220073	2.3517	55.9308	0.780735	8.184104		5.685102	2.051893	0.539671
4.075775	4.758834	20.74787	0.97635	12.13266	4.607013	10.52386	1.544046	0.147479
4.013124	4.241304	21.94005	0.977235	12.88546	4.646128	10.10146	1.630397	0.160239
3.937933	4.102164	76.25839	0.933337	14.06817		9.779684	1.655629	0.158067
3.866169	4.158965	119.9102	0.850984	11.94478		8.894242	1.649579	0.16213
6.738816	2.261038	50.02379	0.84873	8.314858	14.16392	16.27613	1.425444	0.070441
6.491098	1.989023	46.81132	0.829515	7.538636	12.95246	13.90452	1.524264	0.077549
5.936997	1.967747	114.4431	0.804682	7.0345		11.02796	1.600808	0.07863
5.968464	1.957326	433.2273	0.771889	7.051934		9.189556	1.617038	0.080852
4.551961	5.544463		1.039791	6.941802	4.589716	14.67086	1.815995	0.182617

For peer review only

4.258575	5.00795		0.994013	6.409547	4.32729	12.29348	1.939148	0.188051
4.423513	3.386553	236.6613	0.919019	5.930039		12.00285	1.890675	0.168439
4.528301	3.316736	802.35	0.859255	5.939668		12.2089	1.958449	0.161786
2.289074	3.063488		0.986971	12.28685	5.819386	7.602039	2.396465	0.275795
2.208381	2.771706		0.872562	11.64974	5.431328	7.378274	2.378269	0.289455
2.042037	2.56264		0.763581	10.0092		7.007391	2.458744	0.310157
1.983887	2.497409		0.726418	8.411363		6.783066	2.570478	0.324243
5.737968	7.439762	139.9756	1.196811	16.24944	7.869025	6.360374	1.54925	0.210974
5.475748	7.498375	78	1.033845	14.03738	7.126095	6.074784	1.518599	0.218883
5.364709	6.296228	468.5	1.027371	16.20104		8.407778	1.389229	0.220779
5.562353	4.767586	2944.5	0.996406	16.45793		13.38166	1.385422	0.214107
7.839628	5.927733	30.49398	1.197827	25.8675	10.18933	37.38273	1.233138	0.123915
7.97763	5.389111	32.77119	1.163819	24.90982	10.67541	36.35847	1.220378	0.119322
8.168528	5.106367	68.08696	1.098398	21.2926		33.10196	1.295484	0.126881
8.738901	5.25755	169.8367	1.094118	18.11914		31.84822	1.510236	0.126597
4.327938	3.664261		0.753896	5.206644	6.78705	9.848901	1.932266	0.205182
4.226051	3.336628		0.730856	5.004628	6.433854	8.987183	2.031156	0.218817
4.136962	3.279453		0.713763	4.930697		9.417715	2.124795	0.234302
4.06342	3.057222		0.675572	4.817706		8.221573	2.173398	0.264933
5.470254	10.35212		1.205431	16.66321	11.18982	12.05826	1.547317	0.137665
5.015019	7.49507		1.021941	17.70527	10.71216	10.73883	1.621269	0.145561
4.704951	5.487217		0.868272	16.46531		8.444948	1.650797	0.158429
4.92619	5.510526		0.912866	8.534693		8.98244	1.678554	0.153356
2.729957	4.405641		0.962745	7.737432	3.766407	14.60361	1.748563	0.078291
2.711345	4.666243		0.930279	7.502913	3.853621	14.887	1.760524	0.074848
2.501575	4.063453		0.849346	6.809478		13.49513	1.859162	0.079712
2.326098	3.59532		0.801053	5.934331		12.12324	1.960046	0.094205
5.562124	6.680185	257.5263	1.357962	22.90681	7.335469	17.56032	1.364308	0.110694
5.800042	6.654345		1.307878	22.80689	7.650671	15.31993	1.289522	0.108237
5.653794	5.863996		1.253964	23.46557		14.05752	1.341525	0.110147
5.598178	4.771309	4633.5	1.118309	14.48558		11.34651	1.468525	0.133599
4.597666	3.813239		0.957745	21.24758	5.816657	9.146521	1.77033	0.074861
4.381018	3.444662		0.885551	22.28766	5.886175	8.785762	1.785989	0.077469
4.416839	3.313629		0.853341	21.64458		7.927502	1.735758	0.071566
4.101207	3.103966		0.720906	14.65605		7.335857	1.832524	0.102499
5.724599	5.357583	1769.349	1.075338	4.533521	7.23381	16.79901	1.318626	0.148833
5.43148	5.001415		1.005293	4.323627	6.963649	13.7785	1.482776	0.155549
5.355807	4.983023		1.046261	4.412059		13.86114	1.561744	0.137511
5.679736	4.926794		1.030637	4.485954		10.76888	1.555429	0.131131
13.54051	3.649402		1.056456	6.299466	49.90826	5.125719	1.566815	0.184207
14.74343	3.82024		1.208329	9.35406	62.95185	5.221431	1.452657	0.185014
13.23374	3.540325		1.180681	9.886471		5.089056	1.46439	0.191243
14.0903	3.337465		1.113432	10.41955		4.897955	1.477151	0.194185
1.979183	2.361514	2722.2	0.815929	13.30102	4.576572	4.166208	2.202463	0.456707
1.878403	2.214433		0.751565	13.00502	4.660881	3.846525	2.376006	0.4592
5.867968	4.867686	312.6429	0.950302	10.31301	8.177049	8.43545	1.71164	0.183767
5.713301	4.569887	1847.143	0.875882	9.804565	7.704707	7.563053	1.791954	0.189964
5.782008	4.300307	13002	0.806825	9.45664		7.145011	1.825951	0.190674
5.942993	4.183783		0.789056	10.28386		7.197964	1.84785	0.191514
3.760453	4.674781	87.51798	1.067333	7.695684	6.859479	14.47971	1.734375	0.135043

For peer review only

3.678691	4.527031	920.7692	0.996055	7.468609	6.584779	14.31533	1.759476	0.143933
3.659267	4.054558	101.4615	1.020802	7.052664		8.729447	1.801524	0.142168
3.628374	3.852198	38.20447	1.099352	6.352298		6.558606	1.818545	0.138292
6.29598	4.959812	169.9273	0.885048	9.76156	8.455309	19.35626	1.443828	0.104466
6.388178	5.113171	169.2292	0.866364	9.886711	8.717917	18.72913	1.43251	0.099073
7.473239	5.87601	343.3614	1.004922	10.32299		23.0042	1.221723	0.085747
7.144804	5.608062	1463.396	0.928834	9.445474		18.55409	1.200394	0.088475
3.556997	3.735519		0.901257	11.19355	5.039315	13.7102	1.845311	0.271751
3.409434	3.520998		0.819275	10.62014	4.777648	13.3525	1.808905	0.278571
3.545027	3.597063		0.824456	11.3121		15.14214	1.775238	0.264766
3.649213	3.626179		0.807122	10.80911		13.63875	1.826462	0.260786
4.574863	5.759474	64.88	1.365766	9.719013	6.747258	18.53924	1.415862	0.105736
4.513456	6.112402	63	1.365804	9.581326	6.572835	17.25686	1.330392	0.116555
4.022224	5.825778	101.3933	1.304427	8.936408		15.80864	1.408346	0.12502
3.902172	5.267555	239.9189	1.16123	9.47605		15.47719	1.538646	0.135276
4.638147	5.820195		1.269807	22.62006	6.458047	16.08273	1.441664	0.139623
4.40972	5.44255		1.163516	19.42103	6.477651	15.76813	1.431498	0.140535
4.195422	5.335686		1.175282	18.46901		8.698848	1.547444	0.147022
4.252847	5.108045		1.142636	17.03565		8.769113	1.705712	0.146841
5.580355	3.75055		1.03946	8.514544	20.1506	17.91573	1.928817	0.183836
4.883229	4.307052		1.170591	9.867753	7.878887	14.9654	1.796923	0.162235
4.64219	4.063807		1.119191	12.7828		11.50625	1.83307	0.183559
4.485504	3.999906		1.0813	11.72592		8.828942	1.937076	0.185619
7.115024	4.309813	43.13736	0.925883	14.29639	14.4061	14.11396	1.37219	0.136047
7.330923	3.871638	101.44	0.837239	12.33145	13.72872	13.05814	1.42175	0.140619
7.042576	3.98603	348.7143	0.815964	17.9965		8.240445	1.452652	0.137511
6.86141	3.781219		0.773702	16.87616		6.350099	1.437785	0.140822
4.039249	3.931641	35.32627	0.828752	8.999242	7.761653	10.93238	1.872264	0.123338
3.962058	3.88388	41.95454	0.807855	8.269588	7.164378	10.34553	1.892253	0.121907
4.051185	3.871306	124.6522	0.839071	7.837201		10.42856	1.819593	0.124464
4.13431	3.727459	1452	0.781575	7.352788		9.631233	1.831222	0.11902
6.734673	4.046198		1.156429	9.553443	9.214613	11.70765	1.28256	0.123535
6.222815	4.088899		1.080775	9.575988	9.277995	11.93324	1.33829	0.139502
5.980843	3.966215		1.045347	8.142939		11.46124	1.388585	0.142636
6.05994	3.718199		0.977773	6.845302		12.15573	1.417223	0.140094
6.088359	4.023753	1698.6	0.962843	14.13778	5.778365	6.567697	1.62759	0.138741
6.460097	3.85482	834.9	0.98776	11.40473	5.564807	5.653922	1.608389	0.161313
5.942112	3.49581	1607.5	0.935041	8.559659		5.416833	1.697228	0.171244
5.68173	3.136652		0.855863	7.892644		5.083535	1.776126	0.16958
3.19289	2.97594		1.010586	10.36225	7.704022	12.08327	2.010314	0.319417
3.158346	2.851606		0.997683	10.51444	8.424048	11.89454	1.998313	0.311067
3.010998	2.728968		0.897595	10.12426		11.18495	2.088755	0.31286
2.829076	2.78503	4546.5	0.766142	10.12284		20.78305	2.125782	0.315745
5.31411	4.363571	30.43173	0.714824	9.321149	6.327966	14.90589	1.802013	0.064955
5.225323	4.177642	1027.5	0.655011	8.590879	6.240056	15.78166	1.821036	0.077032
4.763216	4.094338	1113.857	0.625746	7.60264		15.88205	1.920879	0.082485
4.610613	4.193409	716.7273	0.621586	7.719738		16.79897	1.916053	0.082087
8.079471	5.653588	569.2373	0.886134	10.24923	8.079198	16.56024	1.167781	0.108005
8.060442	5.605388	734.0323	0.847591	9.77357	7.424905	15.41792	1.131522	0.11304
7.472466	4.746741	980.3906	0.718894	9.163391		13.15887	1.236402	0.124102

7.138247	4.424028		0.669273	8.449241		11.08077	1.258556	0.14658
3.214451	3.484895	23.35043	1.00745	16.41906	6.600741	7.449208	2.317765	0.297167
3.048219	3.387949	27.35805	0.900025	14.45365	6.436772	7.757092	2.271379	0.308296
3.007398	3.323397	43	0.845407	14.20961		7.949657	2.24663	0.299899
3.13604	3.291932	77.1729	0.862093	15.73843		8.156496	2.119188	0.278629
2.959	4.250082		0.876265	8.209713	4.504678	17.961	1.734186	0.161682
2.765999	4.232897		0.789711	7.050239	3.880814	15.52364	1.853167	0.174977
3.547926	4.20696		0.74069	6.98511		14.00234	1.96713	0.182297
5.325617	4.147636		0.746314	6.834351		12.70511	1.91755	0.174592
4.195858	3.779454		0.805818	8.438236	7.010026	8.366157	1.71776	0.097182
4.106838	3.347105	1227.5	0.724253	10.48612	7.254662	7.482613	1.646005	0.096193
3.517457	2.777487		0.593407	9.140953		6.245731	1.860979	0.11176
3.432677	2.386071		0.546956	9.947635		7.097146	1.895264	0.138561
4.016921	3.686527	43.84415	1.051206	11.6083	5.991293	4.117723	1.845204	0.167786
3.804446	3.425384	55.08939	0.921944	10.83249	5.821998	4.115948	1.919245	0.176345
3.518954	3.447082	52.44725	0.802221	9.858964		4.41533	2.016013	0.193915
3.514379	3.336246		0.723955	10.39733		4.14659	2.000393	0.194495
6.124755	5.180051	6203.334	0.915596	8.420501	9.225569	17.49316	1.393369	0.12899
5.713823	4.870614		0.827599	7.957413	8.270905	15.23491	1.488921	0.136754
5.649422	4.715298		0.843069	8.821177		13.15934	1.47422	0.141226
5.580257	4.407499		0.851125	8.687055		15.38386	1.497709	0.15287
5.299823	4.749299	46.15513	0.868413	7.04485	9.200728	25.78564	1.539347	0.128956
5.351893	4.351557	154.7647	0.775437	6.38202	8.735471	26.17216	1.571113	0.144943
5.005854	3.748789	105.3803	0.712387	5.933712		18.05763	1.629019	0.145514
4.640077	3.181111	52.74046	0.649961	5.325281		14.22003	1.811438	0.170661
8.01232	5.118269		0.937552	6.388717	11.69512	16.87058	1.613568	0.189262
7.81034	4.737612		0.941311	6.340352	12.03382	18.54823	1.618104	0.217704
7.340684	4.251174		0.805829	7.911334		14.63407	1.587315	0.223393
7.178363	3.747222		0.739023	7.490614		18.26947	1.624944	0.222383
4.100984	4.214879	281.6096	0.772311	9.108667	5.972807	9.268067	1.558682	0.15548
4.132978	4.125981	341.5289	0.776895	9.148435	5.725377	9.773074	1.536535	0.161036
4.038193	3.885639	185.7583	0.727638	7.314707		8.900657	1.621494	0.168267
4.123973	3.924158	477	0.708097	7.229465		8.840959	1.626449	0.164926
1.870179	4.63854		1.270327	15.41385	4.583051	25.39414	2.569217	0.218942
1.586255	4.477063		1.115155	13.47107	3.763052	24.57414	2.783413	0.244375
1.626307	4.541946		1.165	14.30043		27.67537	2.686813	0.243094
1.68105	3.617531		1.131957	13.24228		28.1461	2.734916	0.254138
8.6434	4.234049	29.01015	1.178591	9.481288	5.062552	15.02471	1.392748	0.098163
7.661241	4.121	31.71074	1.118421	9.328714	4.905626	14.9758	1.382585	0.100568
7.573314	4.484493	54.23851	1.105652	8.952041		14.54135	1.427641	0.097712
7.818702	4.659696	88.03433	1.073463	8.601654		13.25706	1.4766	0.101544

For peer review only

ratio_nurse	ratio_nurse	ratio_nurse	ratio_nurse	ratio_midw	ratio_ahp_l	ratio_scien	ratio_stt_b	ratio_nurse
0.249443	0.001326	0	0.004863	0.188648	0.357028	0.143514	0.193235	0.024425
0.274267	0.001364	0	0.002728	0.200066	0.382617	0.14819	0.212346	0.003637
0.285916	0.001705	0	0.000682	0.1982	0.395827	0.103159	0.216074	0.000568
0.278687	0.003948	0	0	0.193263	0.422893	0.063511	0.232591	0
0.184569	0.01135	0.006649	0.007373	0.253702	0.539409	0.415091	0.410834	0.002344
0.238158	0.010824	0.007003	0.008132	0.245941	0.568981	0.432058	0.429339	0.000308
0.304301	0.012791	0.00828	0.008722	0.250355	0.660931	0.467295	0.479768	0
0.549283	0.024448	0.027056	0.000825	0.171351	0.533131	0.08214	0.438294	0.000917
0.367127	0.048416	0.427171	0	0.157524	0.574683	0.163982	0.469306	0
0.340547	0.058757	0.458892	0	0.170083	0.610745	0.162872	0.480716	0
0.350728	0.05305	0.479778	0	0.175664	0.565397	0.177368	0.467995	0.000459
0.394792	0.045487	0.478449	0.001678	0.202233	0.569133	0.200316	0.476341	0.0409
0.061964	0.002101	0	0.019681	0.295062	0.428705	0.316839	0.253605	0.000594
0.025526	0.000595	0	0.024433	0.323434	0.461376	0.346955	0.254119	0.000248
0.023772	0.000588	0	0.020938	0.316991	0.46874	0.350796	0.27243	0
0.023985	0.000574	0	0.015544	0.297109	0.457874	0.341734	0.304334	0
0.268419	0.002598	0.000306	0.00647	0.139441	0.624471	0.068896	0.266616	0.020403
0.214949	0.00337	0	0.003842	0.115249	0.56519	0.070446	0.234638	0.027635
0.193187	0.004185	0	0.004657	0.103345	0.541641	0.059735	0.233321	0.029814
0.129408	0.001095	0	0.002354	0.082118	0.467076	0.051854	0.24417	0.031924
0	0	0	0	0.201352	0.669373	0.366431	0.269148	0
0	0	0	0	0.227223	0.760742	0.428463	0.311848	0
0.235036	0	0	0	0.225715	0.845199	0.423544	0.319395	0
0.237613	0	0	0	0.231033	0.875473	0.431816	0.357318	0
0.003162	0	0.020909	0	0.08929	0.30467	0.125624	0.154527	0
0.003932	0	0.015703	0	0.089123	0.341318	0.136861	0.193819	0
0.005731	0	0.017093	0	0.070678	0.352052	0.143744	0.165641	0
0.008353	0	0.023915	0	0.010771	0.32843	0.129991	0.158076	0
0.000718	0	0	0	0.159585	0.278145	0.038899	0.207478	0
0	0	0	0	0.151751	0.296864	0.043932	0.208289	0
0	0	0	0	0.161114	0.314264	0.051141	0.221646	0
0	0	0	0	0.171023	0.29058	0.053094	0.200601	0
0.025773	0.001658	0.010211	0	0.19395	0.480824	0.329256	0.449376	0
0.027929	0.001782	0.012268	0	0.188373	0.499434	0.332367	0.483968	0.000318
0.028286	0.001718	0.014617	0	0.205748	0.524701	0.328243	0.50416	0.001288
0.026575	0.001759	0.012772	0	0.228322	0.56869	0.333913	0.508357	0.001152
0.552267	0.004339	0.016519	0.009609	0.242368	1.131033	0.265681	0.869279	0
0.573933	0.004627	0.029653	0.003023	0.254308	1.209612	0.279818	1.0462	0
0.546457	0.004444	0.030884	0	0.251933	1.176933	0.271256	1.05116	0
0.511327	0.004464	0	0	0.244204	1.351321	0.269945	1.055448	0
0.126068	0	0	0.001295	0.334332	0.48039	0.139098	0.305405	0.0037
0.126738	0	0	0.002601	0.321395	0.485904	0.136475	0.312809	0.001646
0.104229	0	0	0.00063	0.286947	0.429164	0.117114	0.289253	7.33E-05
0.110228	0	0	0.001261	0.341101	0.492173	0.142098	0.33897	0
0.00237	0	0	0	0.246942	0.49834	0.170258	0.237527	0
0.001829	0	0	0	0.258028	0.491361	0.174061	0.247078	0
0.001817	0	0	0	0.250644	0.506763	0.183082	0.260224	0
0.001974	8.77E-05	0	0	0.307965	0.585963	0.209345	0.285837	0.010464

For peer review only

0.006192	0.001024	0	0.023465	0.290187	0.482535	0.239006	0.336576	0.009462
0.00919	0.001019	0	0.025471	0.28066	0.500956	0.19267	0.33786	0.002986
0.018949	0.001002	0	0.02004	0.285118	0.499322	0.157511	0.338946	0
0.018366	0.001053	0.009456	0.015526	0.328264	0.537802	0.189911	0.369007	0
0	0.000872	0	0	0.322397	0.3096	0.121624	0.211657	0.00654
0.039005	0.000825	0	0.029553	0.268199	0.317312	0.121707	0.223377	0.003265
0.040777	0.000549	0	0.022805	0.263372	0.339476	0.124249	0.236888	0
0.039331	0	0	0.017052	0.271377	0.369983	0.108053	0.222653	0
0	0	0	0	0.275376	0.33311	0.112058	0.161554	0
0	0	0	0	0.293426	0.338076	0.034097	0.172721	0
0	0	0.000184	0.00092	0.301152	0.365185	0.046821	0.163814	0.000184
0	0	0.003216	0.000568	0.305963	0.356159	0.053795	0.18073	0
0.011951	0	0	0.010448	0.376572	0.291656	0.117488	0.225044	0.056947
0.014008	0	0	0.010392	0.381304	0.324445	0.127165	0.203046	0.052295
0.015597	0	0	0.014429	0.424493	0.36004	0.143683	0.22388	0.032204
0.004751	0	0	0.006576	0.423977	0.376871	0.146828	0.238473	0.016852
0	0	0	0	0.243176	0.460532	0.12701	0.225247	0
0.002993	0	0	0	0.248579	0.469594	0.122991	0.235117	0
0.004885	0	0	0	0.23959	0.488445	0.122788	0.241242	0
0.010348	0	0	0	0.230371	0.46744	0.114274	0.227685	0
0	0.001351	0	0	0.213474	0.415959	0.229177	0.277929	0.015484
0	0.002051	0	0	0.216783	0.436085	0.244525	0.277275	0.018803
0	0.002209	0	0	0.219409	0.441342	0.24226	0.274521	0.003682
0.004551	0.002653	0	0	0.223882	0.435058	0.235844	0.264926	0.042801
0.515442	0	0.010654	0.017201	0.329087	0.437803	0.16518	0.23312	0.003037
0.54839	0	0.0163	0.004059	0.327218	0.462532	0.177048	0.261933	0.006085
0.561792	0	0.015598	0.00241	0.326971	0.471677	0.158403	0.269682	0.003486
0.562735	0	0.016933	0.004665	0.321564	0.47674	0.139517	0.268883	0.000945
0.008049	0	0	0	0.116319	0.382682	0.181926	0.280766	0
0.004921	0	0	0	0.124899	0.369882	0.171417	0.275858	0
0	0	0	0	0.161393	0.374203	0.165309	0.301613	0
0	0	0	0	0.169276	0.401421	0.163566	0.30653	0
0	0	0	0	0.186103	0.31398	0.259954	0.264059	0
0	0	0	0	0.192392	0.334374	0.255514	0.274882	0
0	0	0	0	0.201223	0.355632	0.258661	0.302153	0
0.011626	0	0	0	0.198969	0.366794	0.255425	0.318207	0
0.160422	0	0	0	0.207542	0.355176	0.177331	0.200011	0
0.320279	0	0	0	0.21462	0.533793	0.185264	0.262505	0
0.342022	0	0	0	0.209355	0.547358	0.180171	0.279542	0
0.349438	0	0	0	0.204247	0.571047	0.172373	0.252988	0
0.947184	0	0.003155	0	0.233305	0.984529	0.222621	0.36124	0.012562
0.588321	0	0	0	0.232118	0.751493	0.235273	0.392274	0.001545
0.604687	0	0	0	0.252577	0.805263	0.245696	0.448693	0.001657
0.614378	0	0	0	0.240691	0.80291	0.236583	0.435847	0.000637
0	0	0	0	0.236286	0.381255	0.038643	0.2298	0
0	0	0	0	0.218833	0.385947	0.034972	0.231324	0
0	0	0	0	0.21053	0.37256	0.038151	0.226876	0
0	0	0	0	0.212552	0.364463	0.03788	0.224112	0
0	0	0	0.0079	0.278559	0.290693	0.148332	0.165447	0
0	0	0	0.01092	0.278906	0.290294	0.152482	0.178038	0

For peer review only

0	0	0	0.011196	0.279223	0.313705	0.150201	0.193117	0
0	0	0	0.007588	0.275333	0.360024	0.15905	0.213588	0
0.201646	0	0	0	0.134998	0.459109	0.167681	0.195827	0
0.197409	9.20E-05	0	0	0.144171	0.500693	0.178494	0.20697	0
0.140971	0.000551	0	0	0.1443	0.464405	0.182577	0.216919	0
0.134089	0.000754	0	0	0.146668	0.434241	0.202213	0.229102	0
0.863495	0	0	0.02087	0.243919	0.906159	0.183211	0.425735	0
0.895896	0	0	0.02383	0.237042	0.89769	0.182881	0.421364	0
1.175967	0.001699	0	0.045455	0.249323	0.917339	0.192715	0.41337	0
1.290243	0.007457	0	0.035546	0.24638	0.956186	0.185241	0.407363	0
0.175564	0	0.002294	0	0.23093	0.497477	0.190807	0.185903	0.010514
0.156822	0	0.002291	0	0.217684	0.499458	0.276745	0.187695	0.007397
0.196683	0	0.002564	0	0.226927	0.603077	0.355	0.218992	0.002938
0.24029	0	0.004247	0	0.210295	0.57141	0.347892	0.226658	0
0	0	0.006899	0.006562	0.175112	0.274753	0.059981	0.192203	0
0	0	0.005488	0.006657	0.177613	0.267566	0.056178	0.190517	0
0	0	0.007429	0.003945	0.164957	0.272908	0.054409	0.208883	0
0	0	0.006456	0.001135	0.162275	0.259219	0.054501	0.210338	0
0	0	0	0	0.233793	0.371212	0.221002	0.193484	0.000909
0	0	0	0	0.241368	0.416793	0.2336	0.220795	0.000679
0	0	0	0	0.222859	0.419269	0.224176	0.221653	0.000131
0	0	0	0	0.220764	0.432298	0.215033	0.21313	0.00013
0	0	0	0	0.215474	0.454636	0.186453	0.247403	0
0	0	0	0	0.243226	0.484657	0.195302	0.274632	0.002732
0.000918	0	0	0	0.24471	0.489022	0.193656	0.279198	0.006064
0.001773	0	0	0	0.234712	0.476456	0.181033	0.27291	0.042352
0	0	0	0	0.233877	0.257041	0.151469	0.162639	0
0	0	0	0	0.205977	0.290773	0.142102	0.166243	0
0	0	0	0	0.208408	0.28309	0.14047	0.176491	0
0	0	0	0	0.229374	0.287393	0.136885	0.175879	0
0	0	0	0	0.230237	0.245703	0.076547	0.15681	0.039797
0	0	0	0	0.214153	0.242545	0.068297	0.14894	0.046597
0	0	0	0	0.215528	0.258101	0.065358	0.162166	0.057153
0	0	0	0	0.223518	0.28196	0.076756	0.150892	0.084892
0.003323	0	0	0	0.202755	0.389676	0.106445	0.199631	0.007393
0.005769	0	0	0	0.207613	0.377539	0.255461	0.19796	0.035082
0.131566	0	0	0	0.198056	0.429974	0.216931	0.230211	0.014712
0.157915	0	0	0	0.201986	0.449927	0.212629	0.23918	0.004706
0.006403	0	0	0.000185	0.203846	0.261939	0.113856	0.168679	0.020906
0.024711	0	0	0.000895	0.203486	0.288549	0.119589	0.172119	0.015829
0.022154	0	0	0.000518	0.205054	0.30812	0.131728	0.180005	0.008003
0.019199	0	0	0.000783	0.224583	0.324366	0.129359	0.177694	0.007353
0.016185	0	0	0	0.05058	0.347701	0.139283	0.1839	0
0.0144	0	0	0	0.052376	0.35784	0.144782	0.199781	0
0.013703	0	0	0	0.051649	0.379229	0.140471	0.205897	0
0.014155	0	0	0	0.049938	0.38408	0.131482	0.216826	0.017301
2.027493	0.085001	0.088189	0.006076	0.177975	0.661741	0.052337	0.492187	0.008022
1.823464	0.071391	0.0964	0.007551	0.178164	0.561505	0.052292	0.538224	0.003866
1.78036	0.073687	0.086014	0.002723	0.180357	0.578872	0.052735	0.540174	0
0.013073	0.003894	0.000432	0	0.193819	0.457215	0.217609	0.238434	0

0.00868	0.003928	0.00069	0	0.191756	0.454476	0.230354	0.248434	0
0.008703	0.006243	0.000984	0	0.198127	0.47185	0.236408	0.267965	0
0.005069	0.007304	0.001232	0	0.21216	0.445366	0.231253	0.263847	0.001434
0	0	0	0	0.002925	0.382271	0.077597	0.270419	0
0	0	0	0	0.002773	0.376786	0.077037	0.258215	0.008349
0	0	0	0	0.002719	0.369557	0.072839	0.256606	0.001159
0.013789	0	0	0	0.001432	0.447614	0.171824	0.300609	0
0.001075	0	0	0.022395	0.276842	0.360946	0.188553	0.178142	0.030368
0.001133	0.000472	0.000945	0.024936	0.308359	0.398177	0.215722	0.192444	0.051856
0.001154	0.001346	0.002692	0.027978	0.313645	0.400805	0.230067	0.186858	0.041438
0.000865	0.002595	0.003702	0.019896	0.304555	0.41113	0.245681	0.188868	0.032103
0	0	0.001345	0	0.289536	0.369534	0.211356	0.297171	0.007634
0	0	0.001923	0	0.27768	0.379143	0.206384	0.28733	0.003929
0	0	0.003028	0	0.285809	0.413082	0.212362	0.310143	0.003983
0	0.000608	0.002433	0	0.285946	0.419619	0.217361	0.316582	0.001946
0.48457	0.001229	0	0.00158	0.193812	0.592177	0.179113	0.298527	0.003639
0.506851	0.002107	0	0.00281	0.186067	0.631329	0.183823	0.313953	0.001042
0.542555	0.00221	0	0.002947	0.197698	0.659327	0.193321	0.322188	0
0.52576	0.002277	0	0.002466	0.2098	0.695407	0.218882	0.327698	8.85E-05
0.026914	0	0.002167	0.000903	0.201266	0.409278	0.181726	0.308619	0
0.055375	0	0.002205	0	0.21139	0.443133	0.191475	0.386358	0
0.088266	0	0.002154	0	0.227375	0.488868	0.133089	0.393809	0
0.059103	0	0.001986	0	0.219913	0.468836	0.071495	0.389895	0.000182
0	0.000146	0	0	0.224669	0.381346	0.178159	0.252004	0.000437
0	0.001308	0	0	0.28212	0.372618	0.177434	0.265619	0
0	0.000213	0	0	0.273944	0.380172	0.184523	0.262824	8.54E-05
0.004385	0	0	0	0.237989	0.338295	0.169181	0.249253	0
0	0	0	0	0.157037	0.277662	0.065727	0.22721	0.019642
0	0	0	0	0.160147	0.281567	0.065163	0.241273	0.019687
0	0	0	0	0.158766	0.302409	0.063364	0.257779	0.008112
0	0	0	0	0.147681	0.276186	0.057726	0.253404	0.000184
0.129462	0	0	0	0.198926	0.394247	0.093957	0.282426	0.009987
0.212531	0	0	0	0.186599	0.415822	0.094959	0.253792	0.008701
0.294451	0	0	0	0.192697	0.45623	0.099502	0.242535	0.008718
0.050918	0.001562	0	0	0.245759	0.429698	0.074779	0.294181	0
0.18742	0.002674	0	0	0.228452	0.594252	0.088339	0.335911	0
0.346504	0.002793	0	0	0.239406	0.77747	0.108591	0.424997	0
0.370027	0.002793	0	0	0.245885	0.838632	0.112728	0.446528	0
0	0	0	0.008593	0.160688	0.431087	0.546588	0.291204	0
0.000195	0	0	0.002369	0.166595	0.445602	0.48939	0.289262	0
0.000775	0	0	0	0.16463	0.460436	0.481244	0.298099	0
0.000787	0	0	0	0.176474	0.498337	0.489848	0.318805	0
0.147537	0	0.001078	0	0.163602	0.443453	0.199124	0.174047	0
0.292809	0	0.002162	0	0.166361	0.565354	0.210791	0.201171	0
0.311932	0	0.00222	0	0.166962	0.614396	0.229451	0.219722	0
0.314556	0	0.00205	0	0.135688	0.629251	0.240135	0.225455	0
0	0	0	0	0.168051	0.432919	0.24886	0.284044	0
0	0	0	0	0.182433	0.471923	0.256603	0.298778	0
0	0	0	0	0.192157	0.492104	0.270223	0.32224	0
0	0	0	0	0.196391	0.503642	0.298458	0.344587	0

For peer review only

0.242716	0	0	0.000316	0.139556	0.481269	0.326559	0.402111	0.004752
0.247509	0	0	5.25E-05	0.139218	0.486364	0.33481	0.411841	0.001618
0.263435	0	0	0	0.146493	0.52789	0.368249	0.4452	0.002211
0.281637	0.000684	0	0	0.1549	0.55639	0.385902	0.462155	0.018006
0	0	0	0	0.138952	0.292159	0.242577	0.282938	0.000211
0	0	0	0	0.142201	0.299901	0.243552	0.286664	0.000985
0	0	0.001568	0	0.144691	0.310322	0.243256	0.283385	6.38E-05
0	0	0.000247	0	0.157585	0.323117	0.236121	0.283043	0
0.014572	0	0	0.007355	0.221284	0.36443	0.232004	0.157414	0
0.014256	0	0	0.005913	0.224587	0.39233	0.209354	0.170839	0
0.0152	0	0	0.009237	0.247785	0.42791	0.229294	0.209947	0
0.011695	0	0	0.003283	0.254419	0.46305	0.22523	0.237345	0
0.405818	0.007881	0	0.004139	0.271069	1.005454	0.354204	0.686889	0.009693
0.383225	0.009376	0	0.001557	0.258672	1.054621	0.36324	0.708519	0.012426
0.386446	0.009631	0	0.003057	0.25704	1.102899	0.385732	0.75267	0.010191
0.409919	0.008668	0	0.00423	0.269192	1.196762	0.40577	0.839128	0.007511
0.18007	0	0	0.005174	0.294157	0.386585	0.156102	0.168137	0.027716
0.133229	0	0	0.002042	0.306974	0.400916	0.154344	0.187599	0.008261
0.131093	0	0.000464	0.001671	0.311399	0.416833	0.160393	0.206464	0.00195
0.126345	0	0.001564	0.000828	0.31247	0.43173	0.157562	0.242333	0.002576
0.276669	0.013566	0	0.010422	0.258067	0.485742	0.049841	0.200667	0.029216
0.276075	0.013463	0	0.019038	0.241101	0.496431	0.049458	0.201559	0.015298
0.29462	0.012439	0	0.02183	0.235689	0.493917	0.054254	0.209291	0.003292
0.312315	0.012813	0.00316	0.020312	0.253721	0.48158	0.057214	0.231443	0.000347
0.216269	0.005081	0	0	0.194204	0.626842	0.38362	0.314301	0
0.162923	0.005297	0	0	0.202964	0.635745	0.3905	0.337376	0
0.082288	0.005297	0	0	0.217981	0.700347	0.434471	0.409985	0
0.038312	0.001788	0	0	0.218675	0.685444	0.457113	0.426412	0
0.715112	0.001519	0	0.002773	0.191806	0.787832	0.036053	0.219646	0.021737
0.708745	0.004394	0	0.001227	0.195733	0.791725	0.032605	0.236873	0.00228
0.806593	0.003935	0.000361	0.000361	0.222335	0.851897	0.037882	0.259182	0.000361
0.852035	0.001967	0.002219	0.002219	0.229919	0.899432	0.041324	0.263172	0
0.00127	0	0.000372	0.002295	0.17193	0.31892	0.206037	0.229598	0
0.000192	0	0.000839	0.002696	0.163715	0.310085	0.198745	0.2428	0
0	0	0.002107	0.000482	0.157546	0.322503	0.201149	0.255685	0
0	4.85E-05	0.003732	0	0.157245	0.337731	0.20302	0.267724	0
0.000833	0	0	0	0.216541	0.273449	0.053046	0.219229	0.025326
0	0	0	0	0.280922	0.345305	0.067024	0.284536	0.031013
0	0	0	0	0.324149	0.405668	0.079467	0.33618	0.031992
0.203062	0.000249	0	0.007471	0.203897	0.510648	0.192429	0.228001	0.001494
0.200481	0.001517	0	0.001643	0.217721	0.509812	0.190631	0.231414	0.000632
0.193539	0.001451	0.000121	0.003145	0.220799	0.50569	0.181709	0.229834	0.000121
0.19479	0.001449	0.001208	0.003502	0.225117	0.508007	0.180683	0.226245	0
0.642992	0.001197	0.002992	0	0.170338	0.714412	0.194106	0.203341	0
0.41011	0	0.003044	0	0.179452	0.465716	0.206657	0.220094	0
0.39813	0	0.001768	0	0.176886	0.480252	0.195298	0.22546	0
0.339631	0	0	0	0.170778	0.49389	0.192781	0.211379	0
0.002006	0	0	0	0.181173	0.372852	0.153603	0.253422	0
0.001705	0	0	0	0.182042	0.367911	0.156099	0.25618	0
0.001149	0	0	0	0.1864	0.385521	0.166622	0.2881	0

0	0	0	0	0.199194	0.424169	0.171751	0.325156	0
0.002822	0	0.000647	0.006386	0.26887	0.374957	0.201002	0.342482	0.049261
0.00333	0	0.000701	0.005784	0.253716	0.391608	0.171964	0.343068	0.069997
0.003237	0	0.000494	0.006768	0.265969	0.399785	0.172166	0.354693	0.054555
0.002614	0.000512	0.000455	0.003183	0.243065	0.40251	0.173888	0.352725	0.028417
0.035952	0	0	0.002296	0.18537	0.324909	0.18342	0.229819	0
0.005396	0	0	0.00015	0.191394	0.368446	0.19235	0.245389	0
0.004332	0.000782	0	0	0.180816	0.374469	0.191395	0.244829	0
0.00439	0.001056	0	0	0.188614	0.387475	0.208725	0.243033	0
0.00502	0	0	0	0.154515	0.369923	0.270802	0.256415	0.005139
0.006442	0.001655	0	0	0.152139	0.392359	0.275649	0.262074	0.001478
0.006765	0.00284	0	0	0.148563	0.415705	0.286781	0.28764	0
0.00716	0.003954	0.000535	0	0.153542	0.420283	0.290233	0.314083	0
0.005508	0	0.000273	0.001593	0.154636	0.351139	0.269303	0.196398	0.022278
0.006702	0	0	0.001326	0.156982	0.376558	0.285508	0.216498	0.003399
0.006971	0	0	0.001022	0.153757	0.385826	0.289334	0.217486	0.000968
0.009332	0	0.000467	0.000219	0.169642	0.377848	0.289767	0.223199	0.00319
0.889713	0.041043	0.040096	0.03082	0.466852	1.418782	0.172786	0.713871	0.038181
0.764092	0.029194	0.032897	0.032606	0.491571	1.428127	0.165019	0.693974	0.033188
0.738062	0.020261	0.0286	0.024971	0.503196	1.487602	0.154456	0.653564	0.013238
0.758266	0.026703	0.031768	0.020476	0.533917	1.608797	0.158566	0.778925	0.00947
0.289919	0	0	0.005843	0.186471	0.440159	0.265767	0.235352	0.018847
0.302503	0	0	0.000869	0.199497	0.449993	0.284551	0.250258	0.002705
0.286261	0	0	9.55E-05	0.2086	0.450325	0.39082	0.252927	0.000668
0.287387	0.003411	0.002179	0.002369	0.221353	0.464725	0.506084	0.248661	0.006884
0.017677	0	0	0	0.175093	0.493732	0.119431	0.326442	0.002469
0.01821	0	0	0	0.182286	0.545428	0.105204	0.341414	0.003367
0.014989	0	0	0	0.187991	0.544949	0.102805	0.347536	0.002617
0.381767	0.011057	0	0.002259	0.268235	0.764874	0.237117	0.236735	0
0.364241	0.002339	0	0.004032	0.268759	0.794575	0.240966	0.27962	0
0.373456	0.00016	0	0.00167	0.271285	0.775502	0.226502	0.31707	0
0.410737	0	0	0	0.267423	0.799268	0.250568	0.35665	0
0.031591	0	0	0	0.253645	0.259846	0.035948	0.297893	0.033644
0.025079	0	0	0	0.266445	0.287918	0.029428	0.300742	0.031061
0.011606	0	0	0	0.270971	0.282975	0.033117	0.311291	0.001925
0.0032	0	0	0	0.263436	0.274195	0.034062	0.323463	0.0016
0	0	0	0.010706	0.182361	0.373357	0.234814	0.247211	0
0	0	0	0.0118	0.2002	0.419016	0.254776	0.273886	0
0	0	0	0.006967	0.196197	0.436619	0.252947	0.28941	0
0	0	0	0.002029	0.183732	0.447942	0.245655	0.296495	0
0.190406	0	0	0	0.21818	0.470877	0.277092	0.300662	0.007289
0.194043	0	0	0	0.231242	0.527616	0.284101	0.325891	0.004267
0.352896	0.010456	0.001429	0.001905	0	0.578917	0.269352	0.443598	0.007592
0.350227	0.013026	0.002375	0.00147	0	0.580967	0.262712	0.522411	0.002375
0.366331	0.012627	0.002649	0.001104	0	0.596916	0.270127	0.544212	0.000331
0.753712	0.013981	0.002496	0.003063	0.000109	0.883697	0.291661	0.568408	0
0.533082	0.028254	0.007003	0.014598	0.375283	0.640538	0.165598	0.245968	0.030057
0.525561	0.035503	0.009042	0.009946	0.375965	0.661736	0.176365	0.248165	0.018385
0.512971	0.040167	0	0.007748	0.371892	0.704921	0.174256	0.244366	0.007438
0.545123	0.037646	0	0.015725	0.394407	0.741361	0.184717	0.254743	0.00109

For peer review only

0.349238	0.021013	0.001747	0.003042	0.205532	0.474732	0.119672	0.107812	0
0.386605	0.024649	0	0.003492	0.221599	0.548934	0.113016	0.112097	0
0.401739	0.025824	0	0.003445	0.236407	0.566681	0.123395	0.133078	0
0.404672	0.026756	0	0.00458	0.250818	0.621973	0.12694	0.180024	0
0.45152	0.023625	0	0	0.235618	0.628105	0.147126	0.204977	0.00392
0.559879	0.024269	0	0	0.23758	0.648169	0.13623	0.179818	0.002338
0.208944	0.000136	0	0	0.242262	0.35236	0.138416	0.178261	0.001626
0.220317	0	0	0	0.233077	0.35931	0.141408	0.168378	0.001471
0.009755	0	0	0	0.233741	0.349206	0.19571	0.200795	0
0.008197	0	0	0	0.239594	0.376417	0.197513	0.222358	0
0.007415	0	0.000347	0	0.236269	0.390525	0.196824	0.229427	0
0.00851	0	0.001134	0	0.238449	0.385647	0.202195	0.231557	0
0.001063	0	0	0	0.268965	0.28013	0.092896	0.168084	0
0	0	0	0	0.269433	0.309326	0.102428	0.189414	0
0	0	0	0	0.274249	0.33404	0.147868	0.198939	0
0	0	0	0	0.286609	0.346371	0.150538	0.20359	0
0	0	0	0	0.208781	0.295915	0.166103	0.158874	0
0	0	0	0	0.217246	0.308509	0.153122	0.169527	0
0	0	0	0	0.216671	0.30741	0.165819	0.179773	0
0	0	0	0	0.199762	0.28897	0.160147	0.17505	0

0.89376	0.070183	0.411657	0	0.218483	1.295633	0.184854	0.658288	0.001826
0	0	0	0	0.156017	0.16642	0.144392	0.141148	0
0	0	0	0	0.1662	0.172994	0.146659	0.149796	0
0	0	0	0	0.196937	0.18508	0.163214	0.164473	0
0	0	0	0	0.222684	0.213672	0.167545	0.191562	0
0.013607	0.001366	0	0	0.2191	0.256078	0.153172	0.188535	0.006491
0.011597	0.001326	0	0	0.217681	0.258849	0.148378	0.191025	0.003756
0.010259	0.001355	0	0	0.21096	0.273201	0.146232	0.199626	0
0.010647	0.001348	0	0	0.208936	0.267501	0.155335	0.200108	0
0.027903	0	0	0	0.157232	0.30156	0.278561	0.183739	0
0.028053	0	0	0	0.166391	0.317216	0.27301	0.191554	0
0.027727	0	0	0	0.16809	0.324255	0.27826	0.201965	0
0.028039	0	0	0	0.16903	0.350418	0.280144	0.206659	0
0	0	0.001227	0	0.195186	0.377962	0.154103	0.212535	0.00977
0	0	0.001297	0	0.217672	0.408434	0.158639	0.230144	0.003484
0	0	0.001324	0	0.222862	0.410257	0.163351	0.232446	0.001191
0	0	0.001285	0	0.209952	0.396584	0.151556	0.22886	0
0.059499	0.011248	0	0.003192	0.319158	0.255273	0.161293	0.199766	0.035786
0.052801	0.010383	0.001639	0.002004	0.336704	0.298326	0.165136	0.191418	0.037341
0.013696	0.006131	0.002031	0	0.346145	0.323533	0.095544	0.193609	0.021237
0.002054	0.007079	0	0	0.355745	0.355781	0.093026	0.221692	0.009344
0.001337	0	0	0	0	0.449761	0.333726	0.307653	0
0.027323	0	0	0	0	0.489715	0.342856	0.353348	0
0.033534	0	0	0	0	0.499371	0.33863	0.367941	0
0.035509	0	0	0	0	0.491003	0.34112	0.383601	0
0	0	0	0	0.266093	0.309772	0.187932	0.236938	0
0	0	0	0	0.246434	0.305137	0.189826	0.247288	0

For peer review only

0	0	0	0	0.238937	0.295264	0.192415	0.274838	0
0	0	0	0	0.269055	0.32102	0.200097	0.286743	0
0.01612	0.002437	0.001305	0.00544	0.406248	0.323964	0.068753	0.263268	0
0.017138	0.001651	0.00127	0.00688	0.419847	0.326686	0.052419	0.265891	0
0.02021	0.001041	0.001302	0.009168	0.431228	0.34719	0.05717	0.266997	0
0.005968	0.001044	0.001305	0.004208	0.434709	0.338957	0.053851	0.302545	0
0	0.005361	0	0	0.417565	0.278762	0.104004	0.320274	0
0.001502	0	0	0	0.274724	0.229754	0.152957	0.137649	0
0.002223	0.000849	0	0	0.269701	0.220646	0.151435	0.155842	0
0.004296	0.002185	0	0	0.315037	0.261851	0.177624	0.231325	0
0.004404	0.001083	0	0	0.296979	0.258797	0.17481	0.242155	0
0.413946	0.001324	0	0.045539	0.603068	1.228564	0.271101	0.310635	0.028329
0.454621	0	0	0.058047	0.685052	1.364527	0.286289	0.358014	0.02316
0.574093	0.001085	0	0.064722	0.834934	1.679344	0.343468	0.496109	0.042299
0.60656	0.004283	0	0.049065	0.919672	1.849833	0.387866	0.652511	0.017523
0.113479	0.002048	0.001974	0.012583	0.271122	0.384222	0.178649	0.250645	0.016654
0.098254	0.002648	0.002671	0.010237	0.25519	0.375115	0.161007	0.231922	0.013988
0.17756	0	0.096648	0.006301	0.135855	0.368107	0.300986	0.178837	0
0.34521	0	0.095134	0.000474	0.132847	0.45291	0.304176	0.199569	0
0.355337	0	0.101639	0.000961	0.13794	0.470002	0.319054	0.222529	0
0.360517	0	0.111363	0	0.162302	0.507963	0.347166	0.221373	0
0.000427	0	0	0.000191	0.194585	0.479141	0.385112	0.404881	0
6.89E-05	0	0	0.000231	0.188648	0.46692	0.369836	0.436762	0
0	0	0.000257	4.67E-05	0.190625	0.492242	0.375978	0.445511	0
0	0	0.000283	0.000566	0.200148	0.521389	0.383845	0.47895	0
0.093287	0	0	0.008561	0.220871	0.462134	0.069983	0.226127	0
0.094735	0	0	0.005211	0.228298	0.494543	0.072928	0.256865	0
0.093469	0	0	0.005189	0.229939	0.478483	0.072967	0.259063	0
0.504877	0.020382	0	0.003819	0.230821	0.822595	0.079766	0.297206	0.002083
0.051279	0	0	0	0	0.371667	0.315869	0.225916	0
0.071329	0.000307	0.000292	0.001169	0.026021	0.429065	0.341619	0.288455	0.001169
0.075777	0.00175	0.001292	0.003455	0.137078	0.395178	0.242401	0.26294	0.004456
0.072649	0.002142	0.001423	0.002426	0.135548	0.406653	0.248602	0.278116	0.000518
0	0	0.001197	0.007678	0.230383	0.569731	0.265704	0.479798	0.006681
0	0	0.001252	0.009914	0.249069	0.641764	0.282033	0.497723	0.008349
0	0	0.001288	0.004185	0.243254	0.687603	0.303571	0.529906	0.012771
0	0	0.001697	0.00488	0.24994	0.734636	0.306739	0.567059	0.007214
0.17777	0	0.002724	0.004406	0.167745	0.459019	0.387556	0.352232	0.019195
0.204465	0	0.002193	0.003537	0.175337	0.494229	0.408772	0.378671	0.010362
0.201942	0	0.002388	0.003316	0.17764	0.519356	0.432551	0.402579	0.005354
0.196432	0.001015	0.002009	0.002103	0.179808	0.543172	0.450436	0.425224	0.017879
0.002091	0.002561	0.000697	0	0.238077	0.34242	0.245352	0.210136	0.048198
0.00214	0.002996	0	0	0.242559	0.360773	0.249182	0.235777	0.045579
0.002569	0.002997	0	0	0.240075	0.364495	0.25394	0.243774	0.013113
0.002996	0.00264	0	0	0.243008	0.372479	0.258654	0.240444	0.00834
0.600147	0.021971	0.078301	0	0.155421	0.58617	0.148394	0.442275	0.01999
0.582914	0.027911	0.084216	0	0.163155	0.628223	0.154057	0.502759	0.021362
0.606395	0.029474	0.079034	0	0.166231	0.671039	0.168435	0.508195	0.008738
0.619892	0.031121	0.073954	0	0.181926	0.671067	0.167547	0.510901	0.002308
0.009888	0.003442	0	0.001278	0.221059	0.481174	0.219686	0.18036	0

For peer review only

0	0.003921	0	0	0.231236	0.531169	0.23482	0.199683	0
0.000164	0.003511	0	0	0.239196	0.513443	0.226065	0.295285	0.004225
0.000723	0.002061	0	0	0.24233	0.501307	0.220833	0.301501	0.001246
0.001111	0	0	0	0.241741	0.458479	0.436858	0.326425	0
0.003206	0	0	0	0.238018	0.492267	0.45282	0.360789	0
0.00396	0.003548	0.00095	0	0.24452	0.530704	0.489707	0.390223	0
0.001294	0.007149	0.002264	0	0.251074	0.620127	0.504061	0.400415	0
0	0	0	0	0.241451	0.380678	0.174278	0.134413	0.007144
0.021688	0	0	0	0.244625	0.429185	0.182623	0.133362	0.012821
0.019649	0	0	0	0.246536	0.436977	0.186403	0.158825	0.002134
0.017762	0.001121	0	0	0.240308	0.427301	0.17978	0.20975	0.00034
0	0	0	0	0.205036	0.268575	0.127557	0.168699	0.032793
0	0	0	0	0.20208	0.288636	0.125351	0.185559	0.030515
0	0	0	0	0.21961	0.288561	0.122421	0.195834	0.014687
0	0	0.000385	0	0.20125	0.279269	0.114431	0.190203	0.005888
0.289056	0.001103	0	0.007195	0.206879	0.644894	0.231057	0.272906	0
0.252492	0	0	0.006336	0.210543	0.660709	0.236628	0.299704	0
0.248846	0	0	0.005492	0.218178	0.668769	0.241723	0.304929	0
0.249842	0.000265	0	0.000703	0.223633	0.689531	0.246098	0.327094	0
0	0	0	0	0.169013	0.338659	0.182807	0.096599	0
0	0.002013	0	0	0.210765	0.373236	0.199401	0.133421	0
0.386203	0.003217	0.000715	0	0.221064	0.525314	0.212542	0.182242	0
0.448408	0.002803	0.000897	0	0.213504	0.53628	0.202997	0.181471	0
0.001983	0.000306	0.00072	0	0.226459	0.364092	0.366306	0.226982	0
0.000573	0	0	0	0.237592	0.360493	0.368821	0.214305	0
0.000879	0	0	0	0.258448	0.419005	0.399748	0.246096	0
0.002004	0	0	0	0.278706	0.46634	0.429905	0.278139	0
0.013299	0	0	0.006131	0.224618	0.170456	0.179787	0.149696	0.003883
0.012255	0	0	0.009731	0.207017	0.194953	0.172413	0.150278	0
0.012792	0	0	0.010753	0.214772	0.214052	0.176872	0.170532	0
0.093798	0.000777	0	0.00723	0.229217	0.399756	0.17863	0.209586	0.000216
8.51E-05	0	0	0	0.226762	0.458465	0.217502	0.262244	0
0	0	0	0	0.236156	0.468242	0.228257	0.290304	0
0	0	0	0	0.233272	0.478574	0.226406	0.301784	0
0	0	0	0	0.241108	0.518286	0.243831	0.322169	0
0.34773	0.001049	0.001155	0.005643	0.175623	0.585523	0.174685	0.186651	0.000565
0.352793	0	0.001495	0.00789	0.18366	0.638766	0.184112	0.199943	0
0.339237	0	0.000689	0.004739	0.191254	0.650738	0.186713	0.200681	0
0.341997	0	0.000691	0.000576	0.194425	0.629962	0.176065	0.202972	0
0.571716	0	0	0	0.268157	0.670918	0.073852	0.274018	0
0.104122	0	0	0	0.263725	0.470944	0.067827	0.261764	0
0.062718	0	0	0	0.276261	0.445542	0.075564	0.28246	0
0.056515	0	0.000877	0	0.260387	0.444154	0.070971	0.299629	0
0.19699	0.024172	0.006819	0.010307	0.265544	0.560895	0.505259	0.423457	0.000367
0.184075	0.020702	0.012046	0.007836	0.281197	0.591176	0.532367	0.451583	0
0.210786	0.000914	0.005483	0.003732	0.303429	0.474554	0.170417	0.205436	0.003199
0.239205	0.00232	0.003944	0.001701	0.320022	0.502787	0.17503	0.218824	0.000541
0.156176	0.001923	0.001307	0.002692	0.317256	0.473431	0.17295	0.232542	7.69E-05
0.134475	0.001781	0.000594	0.003043	0.294072	0.433737	0.168265	0.239018	0
0.002548	0	0.000132	0	0.162665	0.381969	0.265925	0.213914	0.011426

For peer review only

0.001671	0	0.001573	0	0.157237	0.40136	0.271836	0.220895	0.001086
0.002847	0	0.001267	0	0.161255	0.42252	0.273279	0.246636	0.009856
0.004702	0	0	0	0.158541	0.430506	0.275606	0.259592	0.026175
0.022853	0.000758	0	0.001105	0.178504	0.315831	0.158832	0.201621	0.005885
0.020483	0.000747	0	0	0.175838	0.321041	0.156539	0.195573	0.005909
0.020791	0.000326	0	0	0.152342	0.280446	0.133811	0.170184	0.002912
0.0274	0	0	0	0.153666	0.286481	0.139962	0.178315	0.000683
0.031163	0	0	0.000173	0.232786	0.38604	0.281136	0.2677	0
0.028981	0	0	0	0.233727	0.414825	0.293304	0.28401	0
0.026976	0	0	0	0.224172	0.414182	0.282085	0.278005	0
0.028241	0	0	0	0.22026	0.410992	0.274032	0.275772	0
0.000663	0	8.22E-05	0	0.240218	0.441039	0.218586	0.173627	0.015413
0.002009	0	0.000996	0	0.24344	0.431314	0.22156	0.163602	0.015873
0.002145	0	0.001064	0	0.260063	0.45569	0.248619	0.171651	0.009863
0.002181	0	0.000811	0	0.251427	0.468053	0.256268	0.189841	0.004168
0	0	0	0.011614	0.201527	0.250702	0.215603	0.171816	0
0	0	0	0.012373	0.204992	0.278504	0.226772	0.183737	0
0	0	0	0.010849	0.211679	0.290307	0.238355	0.187417	0
0	0	0	0.006325	0.218348	0.315431	0.235137	0.19577	0
0.002935	0	0	0.010056	0.25861	0.338498	0.1792	0.266628	0
0.00261	0	0	0.011236	0.217902	0.283628	0.204783	0.232177	0
0.00262	0	0	0.003456	0.235566	0.285517	0.215416	0.246075	0
0.003105	0	0	0.008318	0.241282	0.345587	0.22294	0.250006	0
0.309331	0	0	0.011881	0.17879	0.436407	0.140548	0.232029	0.023182
0.32255	0	0	0.005652	0.182597	0.462189	0.136408	0.258289	0.009858
0.362784	0	0	0.003414	0.194439	0.502199	0.141993	0.250876	0.002868
0.394784	0	0	0.001685	0.203283	0.537221	0.145743	0.264465	0
0.034132	0	0	0	0.296539	0.459785	0.247571	0.254347	0.028308
0.035613	0	0	0	0.299033	0.476485	0.252394	0.257475	0.023835
0.037127	0	0	0	0.289239	0.47045	0.246841	0.258311	0.008022
0.041739	0.001302	0	0	0.277994	0.475569	0.241878	0.268279	0.000689
0.006413	0	0	0	0.175376	0.424013	0.148485	0.247146	0
0.001102	0	0	0	0.173181	0.435654	0.160699	0.244565	0
0	0	0	0	0.172086	0.451551	0.167201	0.25213	0
0	0	0	0	0.18099	0.493231	0.165018	0.268947	0
0.231188	0	0.000118	0	0.227014	0.469181	0.164248	0.248524	0.000589
0.227671	0	0.00012	0	0.260185	0.506306	0.154796	0.259415	0.001198
0.245872	0	0	0	0.264069	0.559095	0.16829	0.286057	0.000622
0.246625	0	0	0	0.268256	0.601582	0.176003	0.318811	0
0.016681	0	0	0	0.236759	0.422576	0.313196	0.336028	0
0.016637	0	0	0	0.229708	0.421507	0.316621	0.35068	0
0.013178	0	0	0	0.223419	0.441353	0.332116	0.366439	0
0.005846	0	0	0	0.224146	0.45724	0.353472	0.359063	0.00022
0.356293	0	0	0.00097	0.160276	0.5049	0.188178	0.22917	0.03286
0.373673	0	0	0	0.166431	0.555596	0.191376	0.23937	0.000973
0.397786	0	0	0	0.178668	0.612197	0.209942	0.24424	0.000898
0.407214	0	0	0	0.189179	0.620589	0.216891	0.23847	0.001395
0.201023	0.004664	0	0.000893	0.191758	0.442414	0.12377	0.176879	0.001757
0.171026	0.006579	0	0	0.184983	0.455564	0.124063	0.1784	0.001362
0.182569	0.008035	0	0	0.200783	0.513612	0.133825	0.210671	0.00102

For peer review only

0.173556	0.008624	0.000162	0	0.195598	0.52262	0.14009	0.226038	0
0	0	0.001247	0.00052	0.235899	0.434565	0.311095	0.286953	0.042826
0	0	0.000741	0	0.235886	0.453332	0.32806	0.295164	0.036552
0	0	0	0	0.241256	0.459091	0.332513	0.300897	0.023256
0	0	0	0	0.234636	0.445288	0.318874	0.303773	0.012958
0.330712	0	0	0.023767	0.284314	0.508	0.337952	0.23529	0
0.384094	0	0	0.024434	0.309524	0.571701	0.361533	0.236245	0
0.410507	0	0	0.021549	0.325384	0.598074	0.281855	0.237701	0
0.425345	0	0	0.013948	0.30825	0.591302	0.187772	0.241101	0
0.595874	0.006257	0.00157	0.007102	0.121844	0.520119	0.23833	0.264589	0
0.713465	0.005915	0.001629	0.005129	0.117163	0.581153	0.243496	0.298766	0.000815
0.73594	0.006822	0.001239	0.004646	0.130264	0.649516	0.284296	0.360038	0
0.652395	0.006793	0.00127	0.005028	0.132649	0.652281	0.291318	0.419099	0
0	0	0.002934	0.006319	0.172668	0.435838	0.248947	0.271258	0.022808
0	0.00073	0.005649	0.001116	0.185367	0.46557	0.26285	0.291938	0.018152
0	0.002245	0.005972	0.002435	0.202197	0.515784	0.284175	0.2901	0.019067
0	0.001436	0.006556	0.004579	0.202338	0.535364	0.284545	0.299738	0
0.523249	0	0	0.010792	0.216249	0.380288	0.163272	0.193048	0.000161
0.499992	0	0	0.009127	0.233975	0.39618	0.175014	0.205313	0
0.519404	0	0	0.007323	0.229294	0.42348	0.177009	0.212076	0
0.549822	0	0	0.002995	0.233923	0.464847	0.179203	0.226886	0
0.379869	0.0107	0	0.011709	0.215672	0.508876	0.188686	0.210557	0.021666
0.384774	0.010216	0	0.001394	0.222927	0.61147	0.18685	0.229803	0.006461
0.401073	0.010836	0	0	0.237116	0.654122	0.199766	0.266753	0.009489
0.435675	0.013121	0	0.001737	0.25875	0.696023	0.215514	0.314356	0.018961
0.251979	0	0.023348	0	0.219483	0.523021	0.124808	0.195379	0
0.260083	0	0.026224	0	0.232022	0.510303	0.128035	0.211077	0
0.264778	0	0.027034	0.004814	0.246898	0.553407	0.136227	0.235229	0
0.268441	0	0.032613	0.009858	0.255306	0.600711	0.139308	0.266864	0
0	0	0	0.005351	0.266797	0.381164	0.243844	0.237255	0.003551
0	0	0	0.001307	0.272143	0.381651	0.241956	0.242367	0.002928
0	0	0	0.00023	0.298042	0.412368	0.247636	0.257358	0.005383
0	0	0	0	0.310029	0.41925	0.242485	0.254832	0.002096
0.001613	0	0	0	0.314905	0.487552	0.534708	0.215585	0
0.002107	0	0	0	0.320375	0.54926	0.630416	0.223361	0
0.002515	0	0	0	0.307439	0.55294	0.61489	0.22017	0
0.001785	0	0	0	0.306514	0.578129	0.594866	0.276432	0
0	0	0	0.001212	0.188107	0.356804	0.115695	0.236181	0.034471
0	0	0	0.001477	0.183744	0.364065	0.130527	0.24266	0.031535
0	0	0	0.00106	0.189819	0.373695	0.132043	0.222991	0.018437
0	0	0	0.000443	0.197547	0.377362	0.127898	0.214606	0.011359

ratio_nurse	ratio_supp	ratio_supp	ratio_supp	denomitor	od_banding	nhse_regio	nhse_regio	orgme
1.01289	0.063445	0.140883	0.066247	67082.34	2	Y60	Midlands	Dudley Gro
1.067656	0.070686	0.146921	0.073258	67082.34	2	Y60	Midlands	Dudley Gro
1.04333	0.07396		0.080459	67082.34	2	Y60	Midlands	Dudley Gro
1.160435	0.096172		0.083304	67082.34	2	Y60	Midlands	Dudley Gro
1.267233	0.07303	0.178831	0.166323			Y62	North West	Mancheste
1.30082	0.081012		0.166175			Y62	North West	Mancheste
1.408311	0.092713		0.199176			Y62	North West	Mancheste
						Y63	North East	South Tyne
1.247336	0.085249		0.076278			Y63	North East	South Tyne
1.395107	0.148661	0.210879	0.118052	19039.5	2	Y59	South East	Isle of Wigh
1.461128	0.158277	0.214694	0.134446	19039.5	2	Y59	South East	Isle of Wigh
1.511186	0.164247		0.123476	19039.5	2	Y59	South East	Isle of Wigh
1.670005	0.16292		0.137442	19039.5	2	Y59	South East	Isle of Wigh
0.853899	0.064123	0.158993	0.09057	161900.7	3	Y56	London	Barts Healt
0.900013	0.061544	0.161667	0.083256	161900.7	3	Y56	London	Barts Healt
0.893518	0.061958		0.092279	161900.7	3	Y56	London	Barts Healt
0.899469	0.062215		0.075586	161900.7	3	Y56	London	Barts Healt
0.950879	0.103215	0.055176	0.081172			Y56	London	London Nor
0.845597	0.090624	0.044994	0.083793			Y56	London	London Nor
0.838254	0.086297		0.078915			Y56	London	London Nor
0.876389	0.086481		0.075648			Y56	London	London Nor
0.715919	0.033817	0.155971	0.053082	46426.67	2.333333	Y59	South East	Royal Surre
0.863132	0.049608	0.179458	0.06245	46426.67	2.333333	Y59	South East	Royal Surre
0.935718	0.067068		0.076281	46426.67	2.333333	Y59	South East	Royal Surre
1.020743	0.096097		0.085801	46426.67	2.333333	Y59	South East	Royal Surre
0.729947	0.070932	0.081115	0.019649	17855.33	2	Y58	South West	Weston Are
0.920224	0.079227	0.081062	0.026068	17855.33	2	Y58	South West	Weston Are
1.005321	0.07953		0.032471	17855.33	2	Y58	South West	Weston Are
0.94131	0.08181		0.040344	17855.33	2	Y58	South West	Weston Are
0.94073	0.047573	0.094979	0.067048	23576	2	Y58	South West	Yeovil Distr
1.087524	0.062577	0.093407	0.07545	23576	2	Y58	South West	Yeovil Distr
1.04548	0.074933		0.110272	23576	2	Y58	South West	Yeovil Distr
1.005719	0.070912		0.105532	23576	2	Y58	South West	Yeovil Distr
1.48302	0.085531	0.154263	0.151005	73501	2	Y58	South West	University H
1.46051	0.079329	0.169653	0.153562	73501	2	Y58	South West	University H
1.490094	0.076052		0.163976	73501	2	Y58	South West	University H
1.487057	0.075184		0.15376	73501	2	Y58	South West	University H
2.233249	0.226108	0.286615	0.554007	38652.67	2	Y58	South West	Torbay and
2.52374	0.301197	0.08451	0.822829	38652.67	2	Y58	South West	Torbay and
2.47205	0.335702		0.845185	38652.67	2	Y58	South West	Torbay and
2.504501	0.33103		0.834476	38652.67	2	Y58	South West	Torbay and
1.000252	0.09766	0.067569	0.064608	76199.34	2	Y63	North East	Bradford Te
0.998747	0.102602	0.062592	0.078095	76199.34	2	Y63	North East	Bradford Te
0.934521	0.096855		0.083653	76199.34	2	Y63	North East	Bradford Te
1.156448	0.11349		0.117304	76199.34	2	Y63	North East	Bradford Te
1.265581	0.149342	0.038965	0.16289	49966	2	Y61	East of Engl	Southend U
1.300838	0.150989	0.03849	0.165817	49966	2	Y61	East of Engl	Southend U
1.355948	0.158078		0.173842	49966	2	Y61	East of Engl	Southend U
1.659615	0.158531		0.218427	49966	2	Y61	East of Engl	Southend U

0.986627	0.0805	0.17177	0.122647	40296	3	Y56	London	Royal Free
1.023975	0.083739	0.141232	0.130869	40296	3	Y56	London	Royal Free
1.05275	0.082864		0.129073	40296	3	Y56	London	Royal Free
1.167923	0.086431		0.116161	40296	3	Y56	London	Royal Free
0.838711	0.038995	0.092983	0.043597	30246.33	3	Y56	London	North Midc
0.86532	0.035363	0.084535	0.033677	30246.33	3	Y56	London	North Midc
0.924657	0.033507		0.049383	30246.33	3	Y56	London	North Midc
0.881963	0.045609		0.057518	30246.33	3	Y56	London	North Midc
0.706697	0.082911	0.127235	0.057014	38569	2.333333	Y56	London	Hillingdon H
0.741995	0.090237	0.080423	0.039986	38569	2.333333	Y56	London	Hillingdon H
0.841733	0.089328		0.044625	38569	2.333333	Y56	London	Hillingdon H
1.008129	0.083417		0.068238	38569	2.333333	Y56	London	Hillingdon H
0.7905	0.083209	0.072426	0.058479	47707.33	2.333333	Y56	London	Kingston Hc
0.768104	0.076225	0.089463	0.055022	47707.33	2.333333	Y56	London	Kingston Hc
0.860167	0.081904		0.061215	47707.33	2.333333	Y56	London	Kingston Hc
0.852916	0.076597		0.080791	47707.33	2.333333	Y56	London	Kingston Hc
1.602853	0.080564	0.042886	0.076465	52280.67	2	Y58	South West	Taunton an
1.674999	0.098124	0.044962	0.137667	52280.67	2	Y58	South West	Taunton an
1.517958	0.09712		0.116503	52280.67	2	Y58	South West	Taunton an
1.492454	0.09358		0.116047	52280.67	2	Y58	South West	Taunton an
1.518736	0.098829	0.138774	0.039867	29638.33	2	Y58	South West	Dorset Cou
1.631591	0.101064	0.137489	0.079972	29638.33	2	Y58	South West	Dorset Cou
1.677176	0.10854		0.078254	29638.33	2	Y58	South West	Dorset Cou
1.721801	0.118735		0.073623	29638.33	2	Y58	South West	Dorset Cou
1.300212	0.122312	0.134608	0.11411	40551.67	2	Y60	Midlands	Walsall Hea
1.281683	0.124772	0.132339	0.108807	40551.67	2	Y60	Midlands	Walsall Hea
1.247673	0.125992		0.110468	40551.67	2	Y60	Midlands	Walsall Hea
1.1621	0.116688		0.097519	40551.67	2	Y60	Midlands	Walsall Hea
1.256398	0.076822	0.17957	0.070736	63502.33	2	Y62	North West	Wirral Univ
1.233993	0.07125	0.175095	0.068752	63502.33	2	Y62	North West	Wirral Univ
1.227864	0.067663		0.075776	63502.33	2	Y62	North West	Wirral Univ
1.272764	0.070483		0.090597	63502.33	2	Y62	North West	Wirral Univ
1.034836	0.08046	0.218169	0.091042	65389.33	2	Y62	North West	St Helens a
1.057153	0.090137	0.21443	0.100007	65389.33	2	Y62	North West	St Helens a
1.06432	0.091241		0.112538	65389.33	2	Y62	North West	St Helens a
1.092219	0.093954		0.114274	65389.33	2	Y62	North West	St Helens a
1.177235	0.076067	0.161859	0.114304	40983.33	1.666667	Y62	North West	Mid Cheshi
1.225432	0.116956	0.169751	0.12226	40983.33	1.666667	Y62	North West	Mid Cheshi
1.216096	0.123733		0.124596	40983.33	1.666667	Y62	North West	Mid Cheshi
1.256147	0.131964		0.114032	40983.33	1.666667	Y62	North West	Mid Cheshi
1.93085	0.376219	0.117632	0.209036	29993.33	2	Y58	South West	Northern D
1.8738	0.32777	0.121769	0.213029	29993.33	2	Y58	South West	Northern D
2.142799	0.366533		0.269316	29993.33	2	Y58	South West	Northern D
2.224245	0.411286		0.286137	29993.33	2	Y58	South West	Northern D
0.849737	0.098567	0.104363	0.069578	27939	2	Y61	East of Engl	Bedford Ho
0.93228	0.108285	0.094116	0.072355	27939	2	Y61	East of Engl	Bedford Ho
0.895682	0.105627		0.080684	27939	2	Y61	East of Engl	Bedford Ho
0.996517	0.109507		0.093684	27939	2	Y56	London	Ealing Hosp
0.916088	0.05358	0.084699	0.040295	60118	2	Y61	East of Engl	Luton and I
0.910179	0.051094	0.090599	0.040263	60118	2	Y61	East of Engl	Luton and I

For peer review only

0.922202	0.051496		0.056228	60118	2	Y61	East of Engl	Luton and D
1.008177	0.053844		0.063468	60118	2	Y61	East of Engl	Luton and D
1.110344	0.085577	0.128031	0.149258	75215.66	2	Y63	North East	York Teachi
1.11717	0.092185	0.142176	0.165107	75215.66	2	Y63	North East	York Teachi
1.143905	0.084644		0.152334	75215.66	2	Y63	North East	York Teachi
1.153294	0.084082		0.162095	75215.66	2	Y63	North East	York Teachi
1.988816	0.173213	0.138307	0.068943	23951	2	Y63	North East	Harrogate a
1.992603	0.176751	0.136653	0.060689	23951	2	Y63	North East	Harrogate a
2.205674	0.181384		0.047811	23951	2	Y63	North East	Harrogate a
2.316769	0.186037		0.056834	23951	2	Y63	North East	Harrogate a
0.951468	0.141829	0.151555	0.054264	31386	2.333333	Y63	North East	Airedale NH
1.055601	0.150373	0.267634	0.067026	31386	2.333333	Y63	North East	Airedale NH
1.200893	0.158469		0.086001	31386	2.333333	Y63	North East	Airedale NH
1.208538	0.153208		0.110236	31386	2.333333	Y63	North East	Airedale NH
0.838539	0.103618	0.056505	0.030671	38448	2	Y61	East of Engl	Queen Eliza
0.852213	0.103834	0.049382	0.034817	38448	2	Y61	East of Engl	Queen Eliza
0.869832	0.100525		0.042748	38448	2	Y61	East of Engl	Queen Eliza
0.983179	0.099766		0.037087	38448	2	Y61	East of Engl	Queen Eliza
1.106363	0.080715	0.105782	0.084233	42109.67	2	Y58	South West	Royal Unite
1.140229	0.095017	0.135021	0.146656	42109.67	2	Y58	South West	Royal Unite
1.153593	0.121359		0.186773	42109.67	2	Y58	South West	Royal Unite
1.195527	0.131557		0.193915	42109.67	2	Y58	South West	Royal Unite
0.940136	0.070099	0.135191	0.152872	50770.67	2	Y58	South West	Poole Hosp
1.043899	0.07614	0.145434	0.158252	50770.67	2	Y58	South West	Poole Hosp
1.051392	0.10236		0.149928	50770.67	2	Y58	South West	Poole Hosp
1.034553	0.115553		0.15098	50770.67	2	Y58	South West	Poole Hosp
0.877184	0.132581	0.144248	0.047938	42119	2	Y61	East of Engl	Milton Keyn
0.847168	0.146383	0.142907	0.055307	42119	2	Y61	East of Engl	Milton Keyn
0.854644	0.136975		0.058388	42119	2	Y61	East of Engl	Milton Keyn
0.849261	0.128518		0.056871	42119	2	Y61	East of Engl	Milton Keyn
1.056361	0.085209	0.054264	0.170508	50706	1.666667	Y61	East of Engl	Basildon an
1.040371	0.079523	0.056503	0.157179	50706	1.666667	Y61	East of Engl	Basildon an
1.024939	0.094812		0.126532	50706	1.666667	Y61	East of Engl	Basildon an
1.057709	0.072745		0.102205	50706	1.666667	Y61	East of Engl	Basildon an
0.820945	0.091882	0.016375	0.114647	52110.33	1.333333	Y61	East of Engl	East Suffolk
0.836709	0.09373	0.188522	0.115435	52110.33	1.333333	Y61	East of Engl	East Suffolk
0.934863	0.109592		0.173726	52110.33	1.333333	Y61	East of Engl	East Suffolk
0.978258	0.118829		0.19072	52110.33	1.333333	Y61	East of Engl	East Suffolk
0.929545	0.056533	0.147199	0.083642	53586	2.333333	Y59	South East	Frimley Hea
0.973752	0.057828	0.145209	0.089072	53586	2.333333	Y59	South East	Frimley Hea
1.009238	0.066421		0.091427	53586	2.333333	Y59	South East	Frimley Hea
1.009749	0.066582		0.087113	53586	2.333333	Y59	South East	Frimley Hea
0.939568	0.060362	0.10828	0.130471	45060.33	2	Y58	South West	Royal Bourn
0.956298	0.061869	0.103684	0.148093	45060.33	2	Y58	South West	Royal Bourn
0.993678	0.063896		0.160737	45060.33	2	Y58	South West	Royal Bourn
1.02955	0.062638		0.15315	45060.33	2	Y58	South West	Royal Bourn
1.332273	0.092743	0.054151	0.089936	23606.33	1.666667	Y63	North East	South Tyne
1.356055	0.082774	0.055193	0.110264	23606.33	1.666667	Y63	North East	South Tyne
1.332932	0.088198		0.11566	23606.33	1.666667	Y63	North East	South Tyne
0.872515	0.111426	0.129522	0.127811	67915	2	Y58	South West	Royal Corn

0.870069	0.121441	0.137947	0.134092	67915	2	Y58	South West	Royal Corn
0.895086	0.131725		0.13559	67915	2	Y58	South West	Royal Corn
0.930416	0.144068		0.186395	67915	2	Y58	South West	Royal Corn
0.660748	0.073688	0.038853	0.097835	41757.33	1	Y62	North West	Liverpool U
0.703841	0.074949	0.036834	0.097134	41757.33	1	Y62	North West	Liverpool U
0.792475	0.071425		0.09106	41757.33	1	Y62	North West	Liverpool U
0.962051	0.079492		0.118509	41757.33	1	Y62	North West	Liverpool U
0.780201	0.06925	0.127886	0.125174	81963	2	Y56	London	Barking, Ha
0.838213	0.075168	0.149719	0.130902	81963	2	Y56	London	Barking, Ha
0.872555	0.082154		0.142016	81963	2	Y56	London	Barking, Ha
0.908333	0.082527		0.148608	81963	2	Y56	London	Barking, Ha
1.013456	0.090631	0.180192	0.110115	42355.67	2	Y63	North East	Barnsley Hc
1.021491	0.096564	0.178967	0.124712	42355.67	2	Y63	North East	Barnsley Hc
1.157115	0.122094		0.16122	42355.67	2	Y63	North East	Barnsley Hc
1.218347	0.129512		0.164258	42355.67	2	Y63	North East	Barnsley Hc
1.222294	0.105712	0.208939	0.059462	43599.33	2	Y63	North East	Rotherham
1.248743	0.11329	0.21768	0.062419	43599.33	2	Y63	North East	Rotherham
1.293182	0.124182		0.070482	43599.33	2	Y63	North East	Rotherham
1.39741	0.126829		0.084522	43599.33	2	Y63	North East	Rotherham
1.264823	0.116827	0.167925	0.090896	45864.67	2	Y60	Midlands	Chesterfiel
1.454534	0.123174	0.180044	0.097326	45864.67	2	Y60	Midlands	Chesterfiel
1.697933	0.144783		0.109945	45864.67	2	Y60	Midlands	Chesterfiel
1.466645	0.132377		0.094492	45864.67	2	Y60	Midlands	Chesterfiel
0.966827	0.096129	0.122709	0.147211	50338.33	2	Y61	East of Engl	North West
1.093137	0.101602	0.107699	0.138601	50338.33	2	Y61	East of Engl	North West
1.159471	0.100607		0.131339	50338.33	2	Y61	East of Engl	North West
1.109443	0.081116		0.12543	50338.33	2	Y61	East of Engl	North West
0.83802	0.085265	0.046344	0.101136	32205.33	2.666667	Y61	East of Engl	James Page
0.88973	0.087685	0.045099	0.101272	32205.33	2.666667	Y61	East of Engl	James Page
1.022346	0.097769		0.10549	32205.33	2.666667	Y61	East of Engl	James Page
1.116252	0.100821		0.119643	32205.33	2.666667	Y61	East of Engl	James Page
0.815256	0.135542	0.016871	0.240717	47073.67	2	Y61	East of Engl	Ipswich Hos
0.860187	0.129101	0.015948	0.25137	47073.67	2	Y61	East of Engl	Ipswich Hos
0.867459	0.104229		0.25212	47073.67	2	Y61	East of Engl	Ipswich Hos
1.047667	0.126091	0.048928	0.065373	32662.33	2	Y61	East of Engl	West Suffol
1.106654	0.144594	0.054813	0.11784	32662.33	2	Y61	East of Engl	West Suffol
1.3216	0.165406		0.157142	32662.33	2	Y61	East of Engl	West Suffol
1.486607	0.178535		0.176903	32662.33	2	Y61	East of Engl	West Suffol
0.864877	0.085502	0.41826	0.047207	70625.66	3	Y61	East of Engl	Cambridge
0.917252	0.089545	0.324238	0.048995	70625.66	3	Y61	East of Engl	Cambridge
1.080397	0.094868		0.055474	70625.66	3	Y61	East of Engl	Cambridge
1.153481	0.100293		0.076019	70625.66	3	Y61	East of Engl	Cambridge
1.324696	0.104974	0.106901	0.112097	59930	2.333333	Y58	South West	Royal Devo
1.544356	0.150117	0.117833	0.11863	59930	2.333333	Y58	South West	Royal Devo
1.651866	0.162053		0.130699	59930	2.333333	Y58	South West	Royal Devo
1.63271	0.195356		0.125027	59930	2.333333	Y58	South West	Royal Devo
1.214823	0.084389	0.14553	0.077157	90155.66	2	Y59	South East	University H
1.373978	0.09105	0.147644	0.07803	90155.66	2	Y59	South East	University H
1.467285	0.076135		0.0844	90155.66	2	Y59	South East	University H
1.625891	0.081724		0.093104	90155.66	2	Y59	South East	University H

For peer review only

1.111176	0.133733	0.172226	0.082342	105725.3	2.333333	Y63	North East	Sheffield Te
1.166503	0.134933	0.172437	0.084638	105725.3	2.333333	Y63	North East	Sheffield Te
1.211982	0.143797		0.088859	105725.3	2.333333	Y63	North East	Sheffield Te
1.333926	0.152518		0.104935	105725.3	2.333333	Y63	North East	Sheffield Te
0.832587	0.107055	0.21109	0.132549	87389	2	Y59	South East	Portsmouth
0.901842	0.116484	0.205338	0.126712	87389	2	Y59	South East	Portsmouth
0.95205	0.112244		0.12453	87389	2	Y59	South East	Portsmouth
1.005582	0.113513		0.131578	87389	2	Y59	South East	Portsmouth
0.832457	0.075429	0.143282	0.074171	61245.33	2	Y59	South East	Royal Berks
0.895913	0.068244	0.137237	0.075319	61245.33	2	Y59	South East	Royal Berks
0.948009	0.067598		0.083678	61245.33	2	Y59	South East	Royal Berks
1.036795	0.075517		0.085435	61245.33	2	Y59	South East	Royal Berks
0.917288	0.083949	0.053528	0.12512	85057.66	2.666667	Y56	London	Guy's and S
1.010871	0.093896	0.046084	0.136518	85057.66	2.666667	Y56	London	Guy's and S
0.944448	0.109		0.143693	85057.66	2.666667	Y56	London	Guy's and S
1.130232	0.117295		0.162223	85057.66	2.666667	Y56	London	Guy's and S
0.827632	0.079153	0.169572	0.053888	44914.33	2.333333	Y56	London	Lewisham a
0.836463	0.086005	0.169545	0.058234	44914.33	2.333333	Y56	London	Lewisham a
0.895287	0.091165		0.055076	44914.33	2.333333	Y56	London	Lewisham a
0.983067	0.10027		0.047412	44914.33	2.333333	Y56	London	Lewisham a
0.79721	0.111202	0.043738	0.041916	45429.33	2	Y56	London	Croydon He
0.83154	0.109555	0.045244	0.049007	45429.33	2	Y56	London	Croydon He
0.907115	0.101232		0.052028	45429.33	2	Y56	London	Croydon He
0.982446	0.105398		0.060119	45429.33	2	Y56	London	Croydon He
0.770342	0.078555	0.283181	0.130458	74048	3	Y56	London	St George's
0.75917	0.071446	0.309673	0.138337	74048	3	Y56	London	St George's
0.766494	0.083386		0.157012	74048	3	Y56	London	St George's
0.796144	0.079445		0.17989	74048	3	Y56	London	St George's
1.207882	0.128974	0.016361	0.063031	23517	2	Y60	Midlands	South Warv
1.259297	0.134602	0.014877	0.070284	23517	2	Y60	Midlands	South Warv
1.436542	0.148346		0.096008	23517	2	Y60	Midlands	South Warv
1.53764	0.175979		0.110486	23517	2	Y60	Midlands	South Warv
1.306102	0.114522	0.198248	0.054875	96981.66	2	Y60	Midlands	University H
1.26073	0.117163	0.195702	0.056474	96981.66	2	Y60	Midlands	University H
1.256107	0.121427		0.060353	96981.66	2	Y60	Midlands	University H
1.224852	0.126799		0.059318	96981.66	2	Y60	Midlands	University H
0.979813	0.133655	0.04	0.060717	41241.33	2	Y60	Midlands	Burton Hos
1.226099	0.162603	0.045087	0.080857	41241.33	2	Y60	Midlands	Burton Hos
1.435257	0.184173		0.091228	41241.33	2	Y60	Midlands	Burton Hos
1.11039	0.150848	0.167144	0.055223	58233	1.666667	Y63	North East	Northern Li
1.075477	0.159066	0.170583	0.066528	58233	1.666667	Y63	North East	Northern Li
1.100527	0.169788		0.063066	58233	1.666667	Y63	North East	Northern Li
1.144737	0.182359		0.065247	58233	1.666667	Y63	North East	Northern Li
1.152176	0.143268	0.196384	0.076016	23852.33	2	Y62	North West	East Cheshi
1.072226	0.096064	0.191923	0.075432	23852.33	2	Y62	North West	East Cheshi
1.079053	0.095852		0.072722	23852.33	2	Y62	North West	East Cheshi
1.088771	0.098096		0.064536	23852.33	2	Y62	North West	East Cheshi
1.254727	0.035232	0.089713	0.159556	47358.67	2	Y62	North West	Countess o
1.23618	0.033284	0.090628	0.164486	47358.67	2	Y62	North West	Countess o
1.32374	0.031622		0.162876	47358.67	2	Y62	North West	Countess o

For peer review only

1.587652	0.030635		0.135967	47358.67	2	Y62	North West	Countess o
0.766119	0.054177	0.068466	0.094877	75638.66	2.333333	Y56	London	King's Colle
0.808099	0.053065	0.063735	0.093553	75638.66	2.333333	Y56	London	King's Colle
0.847142	0.058666		0.087385	75638.66	2.333333	Y56	London	King's Colle
0.839324	0.0628		0.077289	75638.66	2.333333	Y56	London	King's Colle
1.096184	0.074985	0.130205	0.128444	46110	2	Y60	Midlands	Sherwood F
1.265633	0.072736	0.139832	0.150494	46110	2	Y60	Midlands	Sherwood F
1.233637	0.067224		0.165872	46110	2	Y60	Midlands	Sherwood F
1.285427	0.065797		0.184462	46110	2	Y60	Midlands	Sherwood F
1.031038	0.097297	0.243911	0.064804	70894.34	2	Y58	South West	University H
1.126886	0.123267	0.241838	0.067881	70894.34	2	Y58	South West	University H
1.202707	0.095989		0.078223	70894.34	2	Y58	South West	University H
1.312978	0.09571		0.08472	70894.34	2	Y58	South West	University H
0.876499	0.107074	0.244079	0.065833	84643.34	2	Y60	Midlands	University H
0.934805	0.113344	0.265881	0.070669	84643.34	2	Y60	Midlands	University H
0.957034	0.1123		0.070791	84643.34	2	Y60	Midlands	University H
0.991714	0.107048		0.062999	84643.34	2	Y60	Midlands	University H
1.225178	0.129755	0.327306	0.127329	29950	3	Y56	London	Whittington
1.215444	0.13877	0.321005	0.194979	29950	3	Y56	London	Whittington
1.272994	0.140827		0.242902	29950	3	Y56	London	Whittington
1.434575	0.151235		0.273703	29950	3	Y56	London	Whittington
1.168268	0.151304	0.177056	0.045043	60042	2	Y60	Midlands	Royal Wolv
1.197469	0.167015	0.178213	0.048779	60042	2	Y60	Midlands	Royal Wolv
1.245506	0.17768		0.047615	60042	2	Y60	Midlands	Royal Wolv
1.359714	0.183462		0.046238	60042	2	Y60	Midlands	Royal Wolv
1.11535	0.099583	0.077079	0.0393	60876	2	Y63	North East	City Hospita
1.139749	0.097121	0.092699	0.04168	60876	2	Y63	North East	City Hospita
1.180521	0.089473		0.051334	60876	2	Y63	North East	City Hospita
1.794807	0.204744	0.134054	0.196941	28121.33	1.333333	Y60	Midlands	Wye Valley
1.932787	0.238778	0.150368	0.197661	28121.33	1.333333	Y60	Midlands	Wye Valley
1.93529	0.264211		0.193859	28121.33	1.333333	Y60	Midlands	Wye Valley
2.068638	0.270612		0.212323	28121.33	1.333333	Y60	Midlands	Wye Valley
0.910734	0.090071	0.035748	0.095301	23891	1.666667	Y60	Midlands	George Elio
0.895803	0.090007	0.036296	0.170868	23891	1.666667	Y60	Midlands	George Elio
0.8806	0.084745		0.168889	23891	1.666667	Y60	Midlands	George Elio
0.897652	0.088267		0.157593	23891	1.666667	Y60	Midlands	George Elio
0.690351	0.099097	0.187223	0.131017	87265.34	2	Y61	East of Engl	Norfolk anc
0.779538	0.114324	0.211073	0.121964	87265.34	2	Y61	East of Engl	Norfolk anc
0.822502	0.113887		0.123563	87265.34	2	Y61	East of Engl	Norfolk anc
0.933823	0.120404		0.115706	87265.34	2	Y61	East of Engl	Norfolk anc
1.156432	0.069549	0.127721	0.044613	58159.67	2	Y62	North West	University H
1.226573	0.074322	0.140813	0.049789	58159.67	2	Y62	North West	University H
1.486487	0.111528	0.199374	0.167206	46192.67	2.333333	Y62	North West	Salford Roy
1.572725	0.136496	0.209825	0.178488	46192.67	2.333333	Y62	North West	Salford Roy
1.609255	0.149346		0.179359	46192.67	2.333333	Y62	North West	Salford Roy
1.79148	0.195188		0.17852	46192.67	2.333333	Y62	North West	Salford Roy
1.240601	0.124254	0.147048	0.147357	61434.33	2	Y62	North West	Bolton NHS
1.39222	0.117696	0.138962	0.162711	61434.33	2	Y62	North West	Bolton NHS
1.480831	0.115957		0.159578	61434.33	2	Y62	North West	Bolton NHS
1.580221	0.12401		0.165816	61434.33	2	Y62	North West	Bolton NHS

For peer review only

1.084171	0.058789	0.089795	0.137373	34733	1.333333	Y62	North West	Tameside a
1.362492	0.075208	0.09726	0.157767	34733	1.333333	Y62	North West	Tameside a
1.471262	0.082131		0.168294	34733	1.333333	Y62	North West	Tameside a
1.544073	0.096808		0.180961	34733	1.333333	Y62	North West	Tameside a
1.091277	0.077073	0.170297	0.033976	60316.33	2	Y58	South West	Great West
1.152419	0.145669	0.165574	0.057162	60316.33	2	Y58	South West	Great West
0.923693	0.072605		0.087212	60316.33	2	Y58	South West	Great West
0.972849	0.079138		0.09237	60316.33	2	Y58	South West	Great West
1.063521	0.123476	0.153503	0.113073	61133	2	Y59	South East	Hampshire
1.184789	0.143572	0.171347	0.127967	61133	2	Y59	South East	Hampshire
1.192325	0.139906		0.129392	61133	2	Y59	South East	Hampshire
1.304905	0.139029		0.134535	61133	2	Y59	South East	Hampshire
0.864331	0.059937	0.070612	0.111983	52160	2	Y59	South East	Dartford an
0.943668	0.059551	0.076912	0.121166	52160	2	Y59	South East	Dartford an
0.941613	0.071966		0.125766	52160	2	Y59	South East	Dartford an
0.949709	0.0747		0.127335	52160	2	Y59	South East	Dartford an
0.754033	0.111391	0.126064	0.041589	45245.33	2	Y63	North East	North Cuml
0.805841	0.115281	0.151002	0.049113	45245.33	2	Y63	North East	North Cuml
0.834786	0.120332		0.055398	45245.33	2	Y63	North East	North Cuml
0.813024	0.115281		0.05144	45245.33	2	Y63	North East	North Cuml
						Y63	North East	North Cuml
						Y63	North East	North Cuml
						Y63	North East	North Cuml
2.028233	0.306805		0.337833			Y63	North East	North Cuml
1.047999	0.040908	0.132739	0.047917	45038.33	2	Y60	Midlands	Kettering G
1.074513	0.044203	0.138407	0.050134	45038.33	2	Y60	Midlands	Kettering G
1.015171	0.049321		0.052393	45038.33	2	Y60	Midlands	Kettering G
1.092177	0.064215		0.054945	45038.33	2	Y60	Midlands	Kettering G
0.815336	0.079293	0.119824	0.043404	56089.67	1.666667	Y60	Midlands	Northampt
0.757871	0.082201	0.115044	0.058683	56089.67	1.666667	Y60	Midlands	Northampt
0.783922	0.086932		0.082435	56089.67	1.666667	Y60	Midlands	Northampt
0.849228	0.098932		0.092145	56089.67	1.666667	Y60	Midlands	Northampt
1.022867	0.105341	0.13617	0.052111	34894.33	2	Y58	South West	Salisbury N
0.952366	0.122103	0.126837	0.058287	34894.33	2	Y58	South West	Salisbury N
0.978074	0.107496		0.070106	34894.33	2	Y58	South West	Salisbury N
1.015456	0.120986		0.072388	34894.33	2	Y58	South West	Salisbury N
1.004378	0.138923	0.181177	0.207249	74691.34	2	Y63	North East	Doncaster a
1.08729	0.147212	0.189062	0.216113	74691.34	2	Y63	North East	Doncaster a
1.137882	0.141545		0.232534	74691.34	2	Y63	North East	Doncaster a
1.095733	0.147049		0.217891	74691.34	2	Y63	North East	Doncaster a
1.118435	0.057322	0.143438	0.106694	52360.33	1.333333	Y59	South East	Medway NI
1.267199	0.075257	0.144438	0.128862	52360.33	1.333333	Y59	South East	Medway NI
1.117625	0.095075		0.116664	52360.33	1.333333	Y59	South East	Medway NI
1.154332	0.108552		0.121088	52360.33	1.333333	Y59	South East	Medway NI
0.801919	0.074206	0.2289	0.113539	47590.67	2	Y62	North West	Royal Liver
0.916767	0.082916	0.24565	0.119944	47590.67	2	Y62	North West	Royal Liver
0.967727	0.083853		0.124008	47590.67	2	Y62	North West	Royal Liver
0.985905	0.085662		0.127113	47590.67	2	Y62	North West	Royal Liver
1.071524	0.048483	0.146634	0.101846	52732.33	2	Y61	East of Engl	Mid Essex H
1.043445	0.0459	0.130973	0.111691	52732.33	2	Y61	East of Engl	Mid Essex H

For peer review only

1.052519	0.052125		0.116539	52732.33	2	Y61	East of Engl	Mid Essex H
1.218638	0.064723		0.138266	52732.33	2	Y61	East of Engl	Mid Essex H
0.754598	0.058275	0.058644	0.079537	46023.67	3	Y56	London	Chelsea and
0.749228	0.048437	0.047746	0.072881	46023.67	3	Y56	London	Chelsea and
0.785803	0.046097		0.076136	46023.67	3	Y56	London	Chelsea and
0.790563	0.044901		0.076841	46023.67	3	Y56	London	Chelsea and
1.085402	0.10884	0.030294	0.085209	20491.33	2	Y61	East of Engl	Hinchingbro
0.778213	0.062006	0.107658	0.090341	39925.33	2	Y61	East of Engl	Princess Ale
0.733989	0.06804	0.107117	0.083124	39925.33	2	Y61	East of Engl	Princess Ale
0.992023	0.084293		0.050336	39925.33	2	Y61	East of Engl	Princess Ale
1.029999	0.084767		0.052279	39925.33	2	Y61	East of Engl	Princess Ale
1.135398	0.137828	0.283405	0.233487	39808	2.333333	Y56	London	Homerton U
1.270844	0.127955	0.321978	0.264814	39808	2.333333	Y56	London	Homerton U
1.568762	0.132572		0.390458	39808	2.333333	Y56	London	Homerton U
1.670313	0.141717		0.456293	39808	2.333333	Y56	London	Homerton U
1.08179	0.100487	0.157317	0.053921	149689	2	Y60	Midlands	Heart of En
1.050835	0.101987	0.159118	0.044378	149689	2	Y60	Midlands	Heart of En
0.890435	0.112824	0.213118	0.042974	34979.67	2	Y63	North East	Gateshead
0.966735	0.124352	0.230277	0.052635	34979.67	2	Y63	North East	Gateshead
0.985595	0.14854		0.05326	34979.67	2	Y63	North East	Gateshead
1.087732	0.165432		0.056986	34979.67	2	Y63	North East	Gateshead
0.997994	0.101211	0.192189	0.115238	133590.3	2	Y63	North East	Leeds Teach
1.067801	0.106345	0.188022	0.113061	133590.3	2	Y63	North East	Leeds Teach
1.122542	0.110678		0.124484	133590.3	2	Y63	North East	Leeds Teach
1.194041	0.118739		0.156775	133590.3	2	Y63	North East	Leeds Teach
1.838904	0.100938	0.055993	0.071507	45422.67	2	Y62	North West	Wrightingto
1.911043	0.100888	0.064465	0.077296	45422.67	2	Y62	North West	Wrightingto
1.91898	0.091021		0.083228	45422.67	2	Y62	North West	Wrightingto
2.141043	0.150235		0.110172	45422.67	2	Y62	North West	Wrightingto
0.840574	0.083219	0.145672	0.047918	57527.67	2	Y60	Midlands	University H
0.931762	0.098347	0.175291	0.05711	57527.67	2	Y60	Midlands	University H
0.997938	0.098579		0.050689	57527.67	2	Y60	Midlands	University H
1.064714	0.10998		0.051858	57527.67	2	Y60	Midlands	University H
0.759685	0.027847	0.151836	0.081399	68012.34	3	Y56	London	University C
0.837953	0.030087	0.181383	0.109516	68012.34	3	Y56	London	University C
0.937707	0.038574		0.134355	68012.34	3	Y56	London	University C
1.014182	0.044773		0.129545	68012.34	3	Y56	London	University C
1.125457	0.093037	0.185982	0.145466	105789.3	2	Y63	North East	Newcastle U
1.171765	0.097635	0.199734	0.148011	105789.3	2	Y63	North East	Newcastle U
1.207694	0.109971		0.156088	105789.3	2	Y63	North East	Newcastle U
1.280844	0.122188		0.175898	105789.3	2	Y63	North East	Newcastle U
1.024223	0.082422	0.21706	0.095022	83004.66	2	Y58	South West	Gloucesters
1.023295	0.077607	0.215233	0.098996	83004.66	2	Y58	South West	Gloucesters
1.071424	0.071082		0.102253	83004.66	2	Y58	South West	Gloucesters
1.175111	0.083719		0.112432	83004.66	2	Y58	South West	Gloucesters
1.178231	0.120267	0.070602	0.06144	65525.67	2	Y63	North East	Northumbr
1.205523	0.13265	0.077205	0.071919	65525.67	2	Y63	North East	Northumbr
1.242727	0.142157		0.090679	65525.67	2	Y63	North East	Northumbr
1.295524	0.141805		0.108819	65525.67	2	Y63	North East	Northumbr
0.961732	0.144055	0.217878	0.068162	81474	2	Y60	Midlands	University H

1.006023	0.156017	0.231092	0.081344	81474	2	Y60	Midlands	University H
1.088117	0.168633		0.083314	81474	2	Y60	Midlands	University H
1.163799	0.16836		0.081907	81474	2	Y60	Midlands	University H
1.013202	0.081388	0.171839	0.131544	107227.7	2	Y59	South East	Oxford Univ
1.146051	0.085839	0.184117	0.135533	107227.7	2	Y59	South East	Oxford Univ
1.309619	0.099908		0.142706	107227.7	2	Y59	South East	Oxford Univ
1.376618	0.118887		0.147426	107227.7	2	Y59	South East	Oxford Univ
0.835554	0.061541	0.127081	0.157223	44638	2	Y59	South East	Ashford and
0.967263	0.071238	0.140329	0.164615	44638	2	Y59	South East	Ashford and
0.973359	0.061724		0.118937	44638	2	Y59	South East	Ashford and
1.003607	0.060761		0.074729	44638	2	Y59	South East	Ashford and
0.834845	0.038659	0.098142	0.02675	49853.67	2	Y59	South East	Surrey and
0.85924	0.040145	0.093673	0.027504	49853.67	2	Y59	South East	Surrey and
0.910417	0.046965		0.03021	49853.67	2	Y59	South East	Surrey and
0.913978	0.05519		0.031399	49853.67	2	Y59	South East	Surrey and
1.326444	0.192062	0.147339	0.101534	90979.34	2	Y63	North East	South Tees
1.368259	0.199815	0.155428	0.11127	90979.34	2	Y63	North East	South Tees
1.401026	0.202811		0.106183	90979.34	2	Y63	North East	South Tees
1.480226	0.207568		0.121631	90979.34	2	Y63	North East	South Tees
0.829579	0.060012	0.089367	0.082931	52944.33	2	Y62	North West	University H
0.97853	0.05648	0.093352	0.09312	52944.33	2	Y62	North West	University H
1.151713	0.060734		0.118414	52944.33	2	Y62	North West	University H
1.095452	0.117169		0.111328	52944.33	2	Y62	North West	University H
1.038697	0.129242	0.265505	0.068476	71994.34	2	Y58	South West	North Bristo
1.074946	0.133282	0.259496	0.067173	71994.34	2	Y58	South West	North Bristo
1.177377	0.146854		0.074101	71994.34	2	Y58	South West	North Bristo
1.248356	0.168511		0.082486	71994.34	2	Y58	South West	North Bristo
0.736398	0.043655	0.136324	0.056947	56564	2	Y56	London	Epsom and
0.764598	0.043846	0.130708	0.065274	56564	2	Y56	London	Epsom and
0.797471	0.042616		0.071136	56564	2	Y56	London	Epsom and
0.894207	0.069034		0.088133	56564	2	Y56	London	Epsom and
1.044119	0.047064	0.17192	0.109331	102999.7	2	Y59	South East	East Kent H
1.12924	0.044868	0.16989	0.113821	102999.7	2	Y59	South East	East Kent H
1.171865	0.046201		0.126143	102999.7	2	Y59	South East	East Kent H
1.387144	0.068231		0.136317	102999.7	2	Y59	South East	East Kent H
0.92994	0.220579	0.13824	0.059527	48988.33	1.666667	Y63	North East	North Tees
0.994734	0.231287	0.143603	0.072577	48988.33	1.666667	Y63	North East	North Tees
0.955784	0.226652		0.072144	48988.33	1.666667	Y63	North East	North Tees
0.970274	0.222918		0.09286	48988.33	1.666667	Y63	North East	North Tees
0.946561	0.158744	0.020037	0.195095	35322	1.666667	Y62	North West	Southport a
0.82759	0.106905	0.015885	0.191518	35322	1.666667	Y62	North West	Southport a
0.846969	0.101148		0.1965	35322	1.666667	Y62	North West	Southport a
0.898124	0.095973		0.204167	35322	1.666667	Y62	North West	Southport a
1.225596	0.075182	0.218504	0.240026	99327.34	2	Y62	North West	Central Ma
1.330558	0.076893	0.214552	0.259975	99327.34	2	Y62	North West	Central Ma
1.052298	0.096965	0.122294	0.118547	132948	2	Y62	North West	Pennine Ac
1.141707	0.101993	0.129791	0.132222	132948	2	Y62	North West	Pennine Ac
1.239426	0.105746		0.139958	132948	2	Y62	North West	Pennine Ac
1.267337	0.09724		0.138928	132948	2	Y62	North West	Pennine Ac
0.936915	0.129943	0.145784	0.069062	90203.66	2	Y63	North East	Hull Univer

For peer review only

1.003961	0.133894	0.151865	0.069855	90203.66	2	Y63	North East	Hull Univer
0.979622	0.14179		0.114555	90203.66	2	Y63	North East	Hull Univer
0.909627	0.157423		0.152471	90203.66	2	Y63	North East	Hull Univer
1.129883	0.102443	0.118269	0.051663	89867	2	Y60	Midlands	United Linc
1.154249	0.101146	0.114706	0.053393	89867	2	Y60	Midlands	United Linc
0.995102	0.096871		0.04347	89867	2	Y60	Midlands	United Linc
1.076618	0.105871		0.053896	89867	2	Y60	Midlands	United Linc
1.109561	0.089337	0.19844	0.072938	139417.3	2	Y60	Midlands	University I
1.220592	0.094161	0.209308	0.074892	139417.3	2	Y60	Midlands	University I
1.212921	0.088401		0.066041	139417.3	2	Y60	Midlands	University I
1.23897	0.092515		0.07332	139417.3	2	Y60	Midlands	University I
0.73219	0.102891	0.148208	0.05394	61950	2	Y59	South East	Maidstone
0.73217	0.10437	0.152141	0.057948	61950	2	Y59	South East	Maidstone
0.76662	0.111902		0.063257	61950	2	Y59	South East	Maidstone
0.861156	0.105529		0.064611	61950	2	Y59	South East	Maidstone
0.787521	0.044209	0.154846	0.062179	57095	2	Y61	East of Engl	West Hertf
0.859464	0.051491	0.154377	0.063419	57095	2	Y61	East of Engl	West Hertf
0.85086	0.054145		0.114958	57095	2	Y61	East of Engl	West Hertf
0.87517	0.0587		0.114037	57095	2	Y61	East of Engl	West Hertf
0.962037	0.117446	0.049626	0.055817	61701.67	1.666667	Y61	East of Engl	East and No
0.854269	0.10134	0.126921	0.066821	61701.67	1.666667	Y61	East of Engl	East and No
0.893503	0.07823		0.086909	61701.67	1.666667	Y61	East of Engl	East and No
0.924813	0.085281		0.113264	61701.67	1.666667	Y61	East of Engl	East and No
1.08005	0.069948	0.069415	0.070852	58432	2	Y62	North West	Stockport N
1.194402	0.081093	0.07284	0.076581	58432	2	Y62	North West	Stockport N
1.225544	0.055566		0.121353	58432	2	Y62	North West	Stockport N
1.292487	0.059255		0.157478	58432	2	Y62	North West	Stockport N
1.206633	0.11112	0.128839	0.091471	74351	2	Y60	Midlands	Worcesters
1.237846	0.120925	0.139579	0.09666	74351	2	Y60	Midlands	Worcesters
1.191795	0.127597		0.095891	74351	2	Y60	Midlands	Worcesters
1.279468	0.136003		0.103829	74351	2	Y60	Midlands	Worcesters
0.864731	0.104674	0.108523	0.085414	50701.33	1.666667	Y62	North West	Warrington
0.925262	0.104428	0.107782	0.0838	50701.33	1.666667	Y62	North West	Warrington
0.95662	0.122806		0.087251	50701.33	1.666667	Y62	North West	Warrington
1.022732	0.146086		0.082266	50701.33	1.666667	Y62	North West	Warrington
1.038591	0.070732	0.173059	0.15226	76020.34	2	Y63	North East	Calderdale
1.012392	0.087683	0.179701	0.176868	76020.34	2	Y63	North East	Calderdale
1.069472	0.116827		0.18461	76020.34	2	Y63	North East	Calderdale
1.168412	0.1267		0.196714	76020.34	2	Y63	North East	Calderdale
0.989525	0.096504	0.129802	0.082759	130412.3	2	Y60	Midlands	Nottinghan
1.002322	0.095107	0.118708	0.084072	130412.3	2	Y60	Midlands	Nottinghan
1.114088	0.098773		0.089406	130412.3	2	Y60	Midlands	Nottinghan
1.305242	0.098787		0.048116	130412.3	2	Y60	Midlands	Nottinghan
1.398946	0.107283	0.158029	0.067088	58002.33	2	Y59	South East	East Sussex
1.526692	0.116403	0.160255	0.063365	58002.33	2	Y59	South East	East Sussex
1.598093	0.131533		0.062964	58002.33	2	Y59	South East	East Sussex
1.608787	0.129538		0.059527	58002.33	2	Y59	South East	East Sussex
1.128497	0.097568	0.123775	0.060386	98946.34	2	Y63	North East	Mid Yorksh
1.179815	0.102317	0.134682	0.06486	98946.34	2	Y63	North East	Mid Yorksh
1.391027	0.10913		0.075994	98946.34	2	Y63	North East	Mid Yorksh

For peer review only

1.494158	0.118354		0.090246	98946.34	2	Y63	North East	Mid Yorksh
0.992605	0.060905	0.151498	0.134242	69211.34	2	Y59	South East	Brighton ar
1.11108	0.069187	0.155357	0.128914	69211.34	2	Y59	South East	Brighton ar
1.182862	0.070375		0.125792	69211.34	2	Y59	South East	Brighton ar
1.159968	0.063539		0.122602	69211.34	2	Y59	South East	Brighton ar
1.141207	0.121807	0.221991	0.055676	73702.66	2	Y60	Midlands	Sandwell ar
1.266286	0.141839	0.257678	0.064418	73702.66	2	Y60	Midlands	Sandwell ar
1.350093	0.143162		0.071417	73702.66	2	Y60	Midlands	Sandwell ar
1.339919	0.14632		0.078709	73702.66	2	Y60	Midlands	Sandwell ar
1.240974	0.118508	0.142653	0.119529	54652	1	Y62	North West	Blackpool T
1.380733	0.095364	0.137842	0.133643	54652	1	Y62	North West	Blackpool T
1.685185	0.109398		0.160109	54652	1	Y62	North West	Blackpool T
1.828299	0.100526		0.140902	54652	1	Y62	North West	Blackpool T
0.951288	0.086145	0.166909	0.242853	74584.34	2	Y62	North West	Lancashire
1.084665	0.092315	0.171762	0.242957	74584.34	2	Y62	North West	Lancashire
1.246539	0.101431		0.226484	74584.34	2	Y62	North West	Lancashire
1.381302	0.096179		0.241162	74584.34	2	Y62	North West	Lancashire
1.092185	0.118758	0.108394	0.057165	85202.66	2	Y63	North East	County Dur
1.208314	0.125669	0.120906	0.065639	85202.66	2	Y63	North East	County Dur
1.186143	0.113364		0.075992	85202.66	2	Y63	North East	County Dur
1.174916	0.115114		0.065003	85202.66	2	Y63	North East	County Dur
1.151525	0.141948	0.108687	0.038781	49457.33	2	Y59	South East	Buckinghan
1.289595	0.15669	0.114476	0.038209	49457.33	2	Y59	South East	Buckinghan
1.403731	0.168529		0.055378	49457.33	2	Y59	South East	Buckinghan
1.538553	0.187784		0.070323	49457.33	2	Y59	South East	Buckinghan
1.066607	0.156526	0.085506	0.059275	80344.34	1.333333	Y62	North West	East Lancas
1.062348	0.15772	0.083099	0.053914	80344.34	1.333333	Y62	North West	East Lancas
1.240958	0.126401		0.068334	80344.34	1.333333	Y62	North West	East Lancas
1.353138	0.1335		0.054736	80344.34	1.333333	Y62	North West	East Lancas
1.294816	0.109786	0.167425	0.107897	63967.67	2	Y60	Midlands	Shrewsbury
1.287175	0.109308	0.174661	0.102322	63967.67	2	Y60	Midlands	Shrewsbury
1.374309	0.136711		0.112351	63967.67	2	Y60	Midlands	Shrewsbury
1.412236	0.138323		0.11311	63967.67	2	Y60	Midlands	Shrewsbury
0.787199	0.064877	0.218195	0.039379	104018	3	Y56	London	Imperial Co
0.896737	0.074233	0.265742	0.040693	104018	3	Y56	London	Imperial Co
0.858369	0.069928		0.036133	104018	3	Y56	London	Imperial Co
0.883426	0.075516		0.035529	104018	3	Y56	London	Imperial Co
0.848471	0.105471	0.197529	0.066557	71694.34	2	Y59	South East	Western Su
0.894118	0.107196	0.203848	0.066774	71694.34	2	Y59	South East	Western Su
0.904444	0.111706		0.068769	71694.34	2	Y59	South East	Western Su
0.931564	0.116257		0.075432	71694.34	2	Y59	South East	Western Su

clustergrou	benchmark	regionct	region	data_acute	data_acute	data_acute	data_acute	teaching
Acute	Acute - Me	Midlands	Other	0	1	0	0	0
Acute	Acute - Me	Midlands	Other	0	1	0	0	0
Acute	Acute - Me	Midlands	Other	0	1	0	0	0
Acute	Acute - Me	Midlands	Other	0	1	0	0	0
Acute	Acute - Tea	North West	Other	0	0	0	0	1
Acute	Acute - Tea	North West	Other	0	0	0	0	1
Acute	Acute - Tea	North West	Other	0	0	0	0	1
Acute	Acute - Larg	North East	Other	1	0	0	0	0
Acute	Acute - Larg	North East	Other	1	0	0	0	0
Community	Community	South East	Other	0	0	0	0	0
Community	Community	South East	Other	0	0	0	0	0
Community	Community	South East	Other	0	0	0	0	0
Community	Community	South East	Other	0	0	0	0	0
Acute	Acute - Tea	London	London	0	0	0	0	1
Acute	Acute - Tea	London	London	0	0	0	0	1
Acute	Acute - Tea	London	London	0	0	0	0	1
Acute	Acute - Tea	London	London	0	0	0	0	1
Acute	Acute - Larg	London	London	1	0	0	0	0
Acute	Acute - Larg	London	London	1	0	0	0	0
Acute	Acute - Larg	London	London	1	0	0	0	0
Acute	Acute - Larg	London	London	1	0	0	0	0
Acute	Acute - Me	South East	Other	0	1	0	0	0
Acute	Acute - Me	South East	Other	0	1	0	0	0
Acute	Acute - Me	South East	Other	0	1	0	0	0
Acute	Acute - Me	South East	Other	0	1	0	0	0
Acute	Acute - Sma	South West	Other	0	0	0	1	0
Acute	Acute - Sma	South West	Other	0	0	0	1	0
Acute	Acute - Sma	South West	Other	0	0	0	1	0
Acute	Acute - Sma	South West	Other	0	0	0	1	0
Acute	Acute - Sma	South West	Other	0	0	0	1	0
Acute	Acute - Sma	South West	Other	0	0	0	1	0
Acute	Acute - Sma	South West	Other	0	0	0	1	0
Acute	Acute - Sma	South West	Other	0	0	0	1	0
Acute	Acute - Tea	South West	Other	0	0	0	0	1
Acute	Acute - Tea	South West	Other	0	0	0	0	1
Acute	Acute - Tea	South West	Other	0	0	0	0	1
Acute	Acute - Tea	South West	Other	0	0	0	0	1
Acute	Acute - Larg	South West	Other	1	0	0	0	0
Acute	Acute - Larg	South West	Other	1	0	0	0	0
Acute	Acute - Larg	South West	Other	1	0	0	0	0
Acute	Acute - Larg	South West	Other	1	0	0	0	0
Acute	Acute - Tea	North East	Other	0	0	0	0	1
Acute	Acute - Tea	North East	Other	0	0	0	0	1
Acute	Acute - Tea	North East	Other	0	0	0	0	1
Acute	Acute - Tea	North East	Other	0	0	0	0	1
Acute	Acute - Me	East of Engl	Other	0	1	0	0	0
Acute	Acute - Me	East of Engl	Other	0	1	0	0	0
Acute	Acute - Me	East of Engl	Other	0	1	0	0	0
Acute	Acute - Me	East of Engl	Other	0	1	0	0	0

For peer review only

Acute	Acute - Tea	London	London	0	0	0	0	1
Acute	Acute - Tea	London	London	0	0	0	0	1
Acute	Acute - Tea	London	London	0	0	0	0	1
Acute	Acute - Tea	London	London	0	0	0	0	1
Acute	Acute - Me	London	London	0	1	0	0	0
Acute	Acute - Me	London	London	0	1	0	0	0
Acute	Acute - Me	London	London	0	1	0	0	0
Acute	Acute - Me	London	London	0	1	0	0	0
Acute	Acute - Sma	London	London	0	0	0	1	0
Acute	Acute - Sma	London	London	0	0	0	1	0
Acute	Acute - Sma	London	London	0	0	0	1	0
Acute	Acute - Sma	London	London	0	0	0	1	0
Acute	Acute - Sma	London	London	0	0	0	1	0
Acute	Acute - Sma	London	London	0	0	0	1	0
Acute	Acute - Sma	London	London	0	0	0	1	0
Acute	Acute - Sma	London	London	0	0	0	1	0
Acute	Acute - Me	South West	Other	0	1	0	0	0
Acute	Acute - Me	South West	Other	0	1	0	0	0
Acute	Acute - Me	South West	Other	0	1	0	0	0
Acute	Acute - Me	South West	Other	0	1	0	0	0
Acute	Acute - Sma	South West	Other	0	0	0	1	0
Acute	Acute - Sma	South West	Other	0	0	0	1	0
Acute	Acute - Sma	South West	Other	0	0	0	1	0
Acute	Acute - Sma	South West	Other	0	0	0	1	0
Acute	Acute - Sma	Midlands	Other	0	0	0	1	0
Acute	Acute - Sma	Midlands	Other	0	0	0	1	0
Acute	Acute - Sma	Midlands	Other	0	0	0	1	0
Acute	Acute - Sma	Midlands	Other	0	0	0	1	0
Acute	Acute - Tea	North West	Other	0	0	0	0	1
Acute	Acute - Tea	North West	Other	0	0	0	0	1
Acute	Acute - Tea	North West	Other	0	0	0	0	1
Acute	Acute - Tea	North West	Other	0	0	0	0	1
Acute	Acute - Tea	North West	Other	0	0	0	0	1
Acute	Acute - Tea	North West	Other	0	0	0	0	1
Acute	Acute - Tea	North West	Other	0	0	0	0	1
Acute	Acute - Tea	North West	Other	0	0	0	0	1
Acute	Acute - Sma	North West	Other	0	0	0	1	0
Acute	Acute - Sma	North West	Other	0	0	0	1	0
Acute	Acute - Sma	North West	Other	0	0	0	1	0
Acute	Acute - Sma	North West	Other	0	0	0	1	0
Acute	Acute - Sma	South West	Other	0	0	0	1	0
Acute	Acute - Sma	South West	Other	0	0	0	1	0
Acute	Acute - Sma	South West	Other	0	0	0	1	0
Acute	Acute - Sma	South West	Other	0	0	0	1	0
Acute	Acute - Sma	East of Engl	Other	0	0	0	1	0
Acute	Acute - Sma	East of Engl	Other	0	0	0	1	0
Acute	Acute - Sma	East of Engl	Other	0	0	0	1	0
Acute	Acute - Me	London	London	0	1	0	0	0
Acute	Acute - Me	East of Engl	Other	0	1	0	0	0
Acute	Acute - Me	East of Engl	Other	0	1	0	0	0

For peer review only

Acute	Acute - Me	East of Engl	Other	0	1	0	0	0
Acute	Acute - Me	East of Engl	Other	0	1	0	0	0
Acute	Acute - Tea	North East	Other	0	0	0	0	1
Acute	Acute - Tea	North East	Other	0	0	0	0	1
Acute	Acute - Tea	North East	Other	0	0	0	0	1
Acute	Acute - Tea	North East	Other	0	0	0	0	1
Acute	Acute - Sma	North East	Other	0	0	0	1	0
Acute	Acute - Sma	North East	Other	0	0	0	1	0
Acute	Acute - Sma	North East	Other	0	0	0	1	0
Acute	Acute - Sma	North East	Other	0	0	0	1	0
Acute	Acute - Sma	North East	Other	0	0	0	1	0
Acute	Acute - Sma	North East	Other	0	0	0	1	0
Acute	Acute - Sma	North East	Other	0	0	0	1	0
Acute	Acute - Sma	North East	Other	0	0	0	1	0
Acute	Acute - Sma	East of Engl	Other	0	0	0	1	0
Acute	Acute - Sma	East of Engl	Other	0	0	0	1	0
Acute	Acute - Sma	East of Engl	Other	0	0	0	1	0
Acute	Acute - Sma	East of Engl	Other	0	0	0	1	0
Acute	Acute - Me	South West	Other	0	1	0	0	0
Acute	Acute - Me	South West	Other	0	1	0	0	0
Acute	Acute - Me	South West	Other	0	1	0	0	0
Acute	Acute - Me	South West	Other	0	1	0	0	0
Acute	Acute - Sma	South West	Other	0	0	0	1	0
Acute	Acute - Sma	South West	Other	0	0	0	1	0
Acute	Acute - Sma	South West	Other	0	0	0	1	0
Acute	Acute - Sma	South West	Other	0	0	0	1	0
Acute	Acute - Sma	East of Engl	Other	0	0	0	1	0
Acute	Acute - Sma	East of Engl	Other	0	0	0	1	0
Acute	Acute - Sma	East of Engl	Other	0	0	0	1	0
Acute	Acute - Sma	East of Engl	Other	0	0	0	1	0
Acute	Acute - Me	East of Engl	Other	0	1	0	0	0
Acute	Acute - Me	East of Engl	Other	0	1	0	0	0
Acute	Acute - Me	East of Engl	Other	0	1	0	0	0
Acute	Acute - Me	East of Engl	Other	0	1	0	0	0
Acute	Acute - Me	East of Engl	Other	0	1	0	0	0
Acute	Acute - Me	East of Engl	Other	0	1	0	0	0
Acute	Acute - Me	East of Engl	Other	0	1	0	0	0
Acute	Acute - Me	East of Engl	Other	0	1	0	0	0
Acute	Acute - Larg	South East	Other	1	0	0	0	0
Acute	Acute - Larg	South East	Other	1	0	0	0	0
Acute	Acute - Larg	South East	Other	1	0	0	0	0
Acute	Acute - Larg	South East	Other	1	0	0	0	0
Acute	Acute - Me	South West	Other	0	1	0	0	0
Acute	Acute - Me	South West	Other	0	1	0	0	0
Acute	Acute - Me	South West	Other	0	1	0	0	0
Acute	Acute - Me	South West	Other	0	1	0	0	0
Acute	Acute - Sma	North East	Other	0	0	0	1	0
Acute	Acute - Sma	North East	Other	0	0	0	1	0
Acute	Acute - Sma	North East	Other	0	0	0	1	0
Acute	Acute - Larg	South West	Other	1	0	0	0	0

For peer review only

Acute	Acute - Larg	South West	Other	1	0	0	0	0
Acute	Acute - Larg	South West	Other	1	0	0	0	0
Acute	Acute - Larg	South West	Other	1	0	0	0	0
Acute	Acute - Tea	North West	Other	0	0	0	0	1
Acute	Acute - Tea	North West	Other	0	0	0	0	1
Acute	Acute - Tea	North West	Other	0	0	0	0	1
Acute	Acute - Tea	North West	Other	0	0	0	0	1
Acute	Acute - Larg	London	London	1	0	0	0	0
Acute	Acute - Larg	London	London	1	0	0	0	0
Acute	Acute - Larg	London	London	1	0	0	0	0
Acute	Acute - Larg	London	London	1	0	0	0	0
Acute	Acute - Sma	North East	Other	0	0	0	1	0
Acute	Acute - Sma	North East	Other	0	0	0	1	0
Acute	Acute - Sma	North East	Other	0	0	0	1	0
Acute	Acute - Sma	North East	Other	0	0	0	1	0
Acute	Acute - Sma	North East	Other	0	0	0	1	0
Acute	Acute - Sma	North East	Other	0	0	0	1	0
Acute	Acute - Sma	North East	Other	0	0	0	1	0
Acute	Acute - Sma	North East	Other	0	0	0	1	0
Acute	Acute - Sma	Midlands	Other	0	0	0	1	0
Acute	Acute - Sma	Midlands	Other	0	0	0	1	0
Acute	Acute - Sma	Midlands	Other	0	0	0	1	0
Acute	Acute - Sma	Midlands	Other	0	0	0	1	0
Acute	Acute - Me	East of Engl	Other	0	1	0	0	0
Acute	Acute - Me	East of Engl	Other	0	1	0	0	0
Acute	Acute - Me	East of Engl	Other	0	1	0	0	0
Acute	Acute - Me	East of Engl	Other	0	1	0	0	0
Acute	Acute - Sma	East of Engl	Other	0	0	0	1	0
Acute	Acute - Sma	East of Engl	Other	0	0	0	1	0
Acute	Acute - Sma	East of Engl	Other	0	0	0	1	0
Acute	Acute - Sma	East of Engl	Other	0	0	0	1	0
Acute	Acute - Me	East of Engl	Other	0	1	0	0	0
Acute	Acute - Me	East of Engl	Other	0	1	0	0	0
Acute	Acute - Me	East of Engl	Other	0	1	0	0	0
Acute	Acute - Sma	East of Engl	Other	0	0	0	1	0
Acute	Acute - Sma	East of Engl	Other	0	0	0	1	0
Acute	Acute - Sma	East of Engl	Other	0	0	0	1	0
Acute	Acute - Sma	East of Engl	Other	0	0	0	1	0
Acute	Acute - Tea	East of Engl	Other	0	0	0	0	1
Acute	Acute - Tea	East of Engl	Other	0	0	0	0	1
Acute	Acute - Tea	East of Engl	Other	0	0	0	0	1
Acute	Acute - Tea	East of Engl	Other	0	0	0	0	1
Acute	Acute - Larg	South West	Other	1	0	0	0	0
Acute	Acute - Larg	South West	Other	1	0	0	0	0
Acute	Acute - Larg	South West	Other	1	0	0	0	0
Acute	Acute - Larg	South West	Other	1	0	0	0	0
Acute	Acute - Tea	South East	Other	0	0	0	0	1
Acute	Acute - Tea	South East	Other	0	0	0	0	1
Acute	Acute - Tea	South East	Other	0	0	0	0	1
Acute	Acute - Tea	South East	Other	0	0	0	0	1

For peer review only

Acute	Acute - Tea	North East	Other	0	0	0	0	1
Acute	Acute - Tea	North East	Other	0	0	0	0	1
Acute	Acute - Tea	North East	Other	0	0	0	0	1
Acute	Acute - Tea	North East	Other	0	0	0	0	1
Acute	Acute - Larg	South East	Other	1	0	0	0	0
Acute	Acute - Larg	South East	Other	1	0	0	0	0
Acute	Acute - Larg	South East	Other	1	0	0	0	0
Acute	Acute - Larg	South East	Other	1	0	0	0	0
Acute	Acute - Larg	South East	Other	1	0	0	0	0
Acute	Acute - Larg	South East	Other	1	0	0	0	0
Acute	Acute - Larg	South East	Other	1	0	0	0	0
Acute	Acute - Larg	South East	Other	1	0	0	0	0
Acute	Acute - Tea	London	London	0	0	0	0	1
Acute	Acute - Tea	London	London	0	0	0	0	1
Acute	Acute - Tea	London	London	0	0	0	0	1
Acute	Acute - Tea	London	London	0	0	0	0	1
Acute	Acute - Larg	London	London	1	0	0	0	0
Acute	Acute - Larg	London	London	1	0	0	0	0
Acute	Acute - Larg	London	London	1	0	0	0	0
Acute	Acute - Larg	London	London	1	0	0	0	0
Acute	Acute - Me	London	London	0	1	0	0	0
Acute	Acute - Me	London	London	0	1	0	0	0
Acute	Acute - Me	London	London	0	1	0	0	0
Acute	Acute - Me	London	London	0	1	0	0	0
Acute	Acute - Tea	London	London	0	0	0	0	1
Acute	Acute - Tea	London	London	0	0	0	0	1
Acute	Acute - Tea	London	London	0	0	0	0	1
Acute	Acute - Tea	London	London	0	0	0	0	1
Acute	Acute - Me	Midlands	Other	0	1	0	0	0
Acute	Acute - Me	Midlands	Other	0	1	0	0	0
Acute	Acute - Me	Midlands	Other	0	1	0	0	0
Acute	Acute - Me	Midlands	Other	0	1	0	0	0
Acute	Acute - Tea	Midlands	Other	0	0	0	0	1
Acute	Acute - Tea	Midlands	Other	0	0	0	0	1
Acute	Acute - Tea	Midlands	Other	0	0	0	0	1
Acute	Acute - Tea	Midlands	Other	0	0	0	0	1
Acute	Acute - Sma	Midlands	Other	0	0	0	1	0
Acute	Acute - Sma	Midlands	Other	0	0	0	1	0
Acute	Acute - Sma	Midlands	Other	0	0	0	1	0
Acute	Acute - Me	North East	Other	0	1	0	0	0
Acute	Acute - Me	North East	Other	0	1	0	0	0
Acute	Acute - Me	North East	Other	0	1	0	0	0
Acute	Acute - Me	North East	Other	0	1	0	0	0
Acute	Acute - Sma	North West	Other	0	0	0	1	0
Acute	Acute - Sma	North West	Other	0	0	0	1	0
Acute	Acute - Sma	North West	Other	0	0	0	1	0
Acute	Acute - Sma	North West	Other	0	0	0	1	0
Acute	Acute - Sma	North West	Other	0	0	0	1	0
Acute	Acute - Sma	North West	Other	0	0	0	1	0
Acute	Acute - Sma	North West	Other	0	0	0	1	0

For peer review only

Acute	Acute - Sma	North West	Other	0	0	0	1	0
Acute	Acute - Tea	London	London	0	0	0	0	1
Acute	Acute - Tea	London	London	0	0	0	0	1
Acute	Acute - Tea	London	London	0	0	0	0	1
Acute	Acute - Tea	London	London	0	0	0	0	1
Acute	Acute - Me	Midlands	Other	0	1	0	0	0
Acute	Acute - Me	Midlands	Other	0	1	0	0	0
Acute	Acute - Me	Midlands	Other	0	1	0	0	0
Acute	Acute - Me	Midlands	Other	0	1	0	0	0
Acute	Acute - Tea	South West	Other	0	0	0	0	1
Acute	Acute - Tea	South West	Other	0	0	0	0	1
Acute	Acute - Tea	South West	Other	0	0	0	0	1
Acute	Acute - Tea	South West	Other	0	0	0	0	1
Acute	Acute - Tea	Midlands	Other	0	0	0	0	1
Acute	Acute - Tea	Midlands	Other	0	0	0	0	1
Acute	Acute - Tea	Midlands	Other	0	0	0	0	1
Acute	Acute - Tea	Midlands	Other	0	0	0	0	1
Acute	Acute - Me	London	London	0	1	0	0	0
Acute	Acute - Me	London	London	0	1	0	0	0
Acute	Acute - Me	London	London	0	1	0	0	0
Acute	Acute - Me	London	London	0	1	0	0	0
Acute	Acute - Larg	Midlands	Other	1	0	0	0	0
Acute	Acute - Larg	Midlands	Other	1	0	0	0	0
Acute	Acute - Larg	Midlands	Other	1	0	0	0	0
Acute	Acute - Larg	Midlands	Other	1	0	0	0	0
Acute	Acute - Larg	North East	Other	1	0	0	0	0
Acute	Acute - Larg	North East	Other	1	0	0	0	0
Acute	Acute - Larg	North East	Other	1	0	0	0	0
Acute	Acute - Mu	Midlands	Other	0	0	1	0	0
Acute	Acute - Mu	Midlands	Other	0	0	1	0	0
Acute	Acute - Mu	Midlands	Other	0	0	1	0	0
Acute	Acute - Mu	Midlands	Other	0	0	1	0	0
Acute	Acute - Sma	Midlands	Other	0	0	0	1	0
Acute	Acute - Sma	Midlands	Other	0	0	0	1	0
Acute	Acute - Sma	Midlands	Other	0	0	0	1	0
Acute	Acute - Sma	Midlands	Other	0	0	0	1	0
Acute	Acute - Tea	East of Engl	Other	0	0	0	0	1
Acute	Acute - Tea	East of Engl	Other	0	0	0	0	1
Acute	Acute - Tea	East of Engl	Other	0	0	0	0	1
Acute	Acute - Tea	East of Engl	Other	0	0	0	0	1
Acute	Acute - Tea	North West	Other	0	0	0	0	1
Acute	Acute - Tea	North West	Other	0	0	0	0	1
Acute	Acute - Tea	North West	Other	0	0	0	0	1
Acute	Acute - Tea	North West	Other	0	0	0	0	1
Acute	Acute - Tea	North West	Other	0	0	0	0	1
Acute	Acute - Tea	North West	Other	0	0	0	0	1
Acute	Acute - Me	North West	Other	0	1	0	0	0
Acute	Acute - Me	North West	Other	0	1	0	0	0
Acute	Acute - Me	North West	Other	0	1	0	0	0
Acute	Acute - Me	North West	Other	0	1	0	0	0

For peer review only

Acute	Acute - Sma	North West	Other	0	0	0	1	0
Acute	Acute - Sma	North West	Other	0	0	0	1	0
Acute	Acute - Sma	North West	Other	0	0	0	1	0
Acute	Acute - Sma	North West	Other	0	0	0	1	0
Acute	Acute - Me	South West	Other	0	1	0	0	0
Acute	Acute - Me	South West	Other	0	1	0	0	0
Acute	Acute - Me	South West	Other	0	1	0	0	0
Acute	Acute - Me	South West	Other	0	1	0	0	0
Acute	Acute - Larg	South East	Other	1	0	0	0	0
Acute	Acute - Larg	South East	Other	1	0	0	0	0
Acute	Acute - Larg	South East	Other	1	0	0	0	0
Acute	Acute - Larg	South East	Other	1	0	0	0	0
Acute	Acute - Sma	South East	Other	0	0	0	1	0
Acute	Acute - Sma	South East	Other	0	0	0	1	0
Acute	Acute - Sma	South East	Other	0	0	0	1	0
Acute	Acute - Sma	South East	Other	0	0	0	1	0
Acute	Acute - Sma	North East	Other	0	0	0	1	0
Acute	Acute - Sma	North East	Other	0	0	0	1	0
Acute	Acute - Sma	North East	Other	0	0	0	1	0
Acute	Acute - Sma	North East	Other	0	0	0	1	0
Acute	Acute - Sma	North East	Other	0	0	0	1	0
Acute	Acute - Sma	North East	Other	0	0	0	1	0
Acute	Acute - Sma	North East	Other	0	0	0	1	0
Acute	Acute - Sma	North East	Other	0	0	0	1	0
Acute	Acute - Sma	Midlands	Other	0	0	0	1	0
Acute	Acute - Sma	Midlands	Other	0	0	0	1	0
Acute	Acute - Sma	Midlands	Other	0	0	0	1	0
Acute	Acute - Sma	Midlands	Other	0	0	0	1	0
Acute	Acute - Me	Midlands	Other	0	1	0	0	0
Acute	Acute - Me	Midlands	Other	0	1	0	0	0
Acute	Acute - Me	Midlands	Other	0	1	0	0	0
Acute	Acute - Me	Midlands	Other	0	1	0	0	0
Acute	Acute - Sma	South West	Other	0	0	0	1	0
Acute	Acute - Sma	South West	Other	0	0	0	1	0
Acute	Acute - Sma	South West	Other	0	0	0	1	0
Acute	Acute - Sma	South West	Other	0	0	0	1	0
Acute	Acute - Tea	North East	Other	0	0	0	0	1
Acute	Acute - Tea	North East	Other	0	0	0	0	1
Acute	Acute - Tea	North East	Other	0	0	0	0	1
Acute	Acute - Tea	North East	Other	0	0	0	0	1
Acute	Acute - Me	South East	Other	0	1	0	0	0
Acute	Acute - Me	South East	Other	0	1	0	0	0
Acute	Acute - Me	South East	Other	0	1	0	0	0
Acute	Acute - Me	South East	Other	0	1	0	0	0
Acute	Acute - Tea	North West	Other	0	0	0	0	1
Acute	Acute - Tea	North West	Other	0	0	0	0	1
Acute	Acute - Tea	North West	Other	0	0	0	0	1
Acute	Acute - Tea	North West	Other	0	0	0	0	1
Acute	Acute - Me	East of Engl	Other	0	1	0	0	0
Acute	Acute - Me	East of Engl	Other	0	1	0	0	0

For peer review only

Acute	Acute - Me	East of Engl	Other	0	1	0	0	0
Acute	Acute - Me	East of Engl	Other	0	1	0	0	0
Acute	Acute - Tea	London	London	0	0	0	0	1
Acute	Acute - Tea	London	London	0	0	0	0	1
Acute	Acute - Tea	London	London	0	0	0	0	1
Acute	Acute - Tea	London	London	0	0	0	0	1
Acute	Acute - Sm	East of Engl	Other	0	0	0	1	0
Acute	Acute - Sm	East of Engl	Other	0	0	0	1	0
Acute	Acute - Sm	East of Engl	Other	0	0	0	1	0
Acute	Acute - Sm	East of Engl	Other	0	0	0	1	0
Acute	Acute - Sm	East of Engl	Other	0	0	0	1	0
Acute	Acute - Me	London	London	0	1	0	0	0
Acute	Acute - Me	London	London	0	1	0	0	0
Acute	Acute - Me	London	London	0	1	0	0	0
Acute	Acute - Me	London	London	0	1	0	0	0
Acute	Acute - Larg	Midlands	Other	1	0	0	0	0
Acute	Acute - Larg	Midlands	Other	1	0	0	0	0
Acute	Acute - Sm	North East	Other	0	0	0	1	0
Acute	Acute - Sm	North East	Other	0	0	0	1	0
Acute	Acute - Sm	North East	Other	0	0	0	1	0
Acute	Acute - Sm	North East	Other	0	0	0	1	0
Acute	Acute - Tea	North East	Other	0	0	0	0	1
Acute	Acute - Tea	North East	Other	0	0	0	0	1
Acute	Acute - Tea	North East	Other	0	0	0	0	1
Acute	Acute - Tea	North East	Other	0	0	0	0	1
Acute	Acute - Me	North West	Other	0	1	0	0	0
Acute	Acute - Me	North West	Other	0	1	0	0	0
Acute	Acute - Me	North West	Other	0	1	0	0	0
Acute	Acute - Me	North West	Other	0	1	0	0	0
Acute	Acute - Tea	Midlands	Other	0	0	0	0	1
Acute	Acute - Tea	Midlands	Other	0	0	0	0	1
Acute	Acute - Tea	Midlands	Other	0	0	0	0	1
Acute	Acute - Tea	Midlands	Other	0	0	0	0	1
Acute	Acute - Tea	London	London	0	0	0	0	1
Acute	Acute - Tea	London	London	0	0	0	0	1
Acute	Acute - Tea	London	London	0	0	0	0	1
Acute	Acute - Tea	London	London	0	0	0	0	1
Acute	Acute - Tea	North East	Other	0	0	0	0	1
Acute	Acute - Tea	North East	Other	0	0	0	0	1
Acute	Acute - Tea	North East	Other	0	0	0	0	1
Acute	Acute - Tea	North East	Other	0	0	0	0	1
Acute	Acute - Larg	South West	Other	1	0	0	0	0
Acute	Acute - Larg	South West	Other	1	0	0	0	0
Acute	Acute - Larg	South West	Other	1	0	0	0	0
Acute	Acute - Larg	South West	Other	1	0	0	0	0
Acute	Acute - Larg	North East	Other	1	0	0	0	0
Acute	Acute - Larg	North East	Other	1	0	0	0	0
Acute	Acute - Larg	North East	Other	1	0	0	0	0
Acute	Acute - Larg	North East	Other	1	0	0	0	0
Acute	Acute - Tea	Midlands	Other	0	0	0	0	1

For peer review only

Acute	Acute - Tea	Midlands	Other	0	0	0	0	1
Acute	Acute - Tea	Midlands	Other	0	0	0	0	1
Acute	Acute - Tea	Midlands	Other	0	0	0	0	1
Acute	Acute - Tea	South East	Other	0	0	0	0	1
Acute	Acute - Tea	South East	Other	0	0	0	0	1
Acute	Acute - Tea	South East	Other	0	0	0	0	1
Acute	Acute - Tea	South East	Other	0	0	0	0	1
Acute	Acute - Me	South East	Other	0	1	0	0	0
Acute	Acute - Me	South East	Other	0	1	0	0	0
Acute	Acute - Me	South East	Other	0	1	0	0	0
Acute	Acute - Me	South East	Other	0	1	0	0	0
Acute	Acute - Me	South East	Other	0	1	0	0	0
Acute	Acute - Me	South East	Other	0	1	0	0	0
Acute	Acute - Me	South East	Other	0	1	0	0	0
Acute	Acute - Me	South East	Other	0	1	0	0	0
Acute	Acute - Tea	North East	Other	0	0	0	0	1
Acute	Acute - Tea	North East	Other	0	0	0	0	1
Acute	Acute - Tea	North East	Other	0	0	0	0	1
Acute	Acute - Tea	North East	Other	0	0	0	0	1
Acute	Acute - Me	North West	Other	0	1	0	0	0
Acute	Acute - Me	North West	Other	0	1	0	0	0
Acute	Acute - Me	North West	Other	0	1	0	0	0
Acute	Acute - Me	North West	Other	0	1	0	0	0
Acute	Acute - Larg	South West	Other	1	0	0	0	0
Acute	Acute - Larg	South West	Other	1	0	0	0	0
Acute	Acute - Larg	South West	Other	1	0	0	0	0
Acute	Acute - Larg	South West	Other	1	0	0	0	0
Acute	Acute - Larg	London	London	1	0	0	0	0
Acute	Acute - Larg	London	London	1	0	0	0	0
Acute	Acute - Larg	London	London	1	0	0	0	0
Acute	Acute - Larg	London	London	1	0	0	0	0
Acute	Acute - Tea	South East	Other	0	0	0	0	1
Acute	Acute - Tea	South East	Other	0	0	0	0	1
Acute	Acute - Tea	South East	Other	0	0	0	0	1
Acute	Acute - Tea	South East	Other	0	0	0	0	1
Acute	Acute - Me	North East	Other	0	1	0	0	0
Acute	Acute - Me	North East	Other	0	1	0	0	0
Acute	Acute - Me	North East	Other	0	1	0	0	0
Acute	Acute - Me	North East	Other	0	1	0	0	0
Acute	Acute - Sma	North West	Other	0	0	0	1	0
Acute	Acute - Sma	North West	Other	0	0	0	1	0
Acute	Acute - Sma	North West	Other	0	0	0	1	0
Acute	Acute - Sma	North West	Other	0	0	0	1	0
Acute	Acute - Tea	North West	Other	0	0	0	0	1
Acute	Acute - Tea	North West	Other	0	0	0	0	1
Acute	Acute - Larg	North West	Other	1	0	0	0	0
Acute	Acute - Larg	North West	Other	1	0	0	0	0
Acute	Acute - Larg	North West	Other	1	0	0	0	0
Acute	Acute - Larg	North West	Other	1	0	0	0	0
Acute	Acute - Tea	North East	Other	0	0	0	0	1

For peer review only

Acute	Acute - Tea	North East	Other	0	0	0	0	1
Acute	Acute - Tea	North East	Other	0	0	0	0	1
Acute	Acute - Tea	North East	Other	0	0	0	0	1
Acute	Acute - Larg	Midlands	Other	1	0	0	0	0
Acute	Acute - Larg	Midlands	Other	1	0	0	0	0
Acute	Acute - Larg	Midlands	Other	1	0	0	0	0
Acute	Acute - Larg	Midlands	Other	1	0	0	0	0
Acute	Acute - Tea	Midlands	Other	0	0	0	0	1
Acute	Acute - Tea	Midlands	Other	0	0	0	0	1
Acute	Acute - Tea	Midlands	Other	0	0	0	0	1
Acute	Acute - Tea	Midlands	Other	0	0	0	0	1
Acute	Acute - Larg	South East	Other	1	0	0	0	0
Acute	Acute - Larg	South East	Other	1	0	0	0	0
Acute	Acute - Larg	South East	Other	1	0	0	0	0
Acute	Acute - Larg	South East	Other	1	0	0	0	0
Acute	Acute - Me	East of Engl	Other	0	1	0	0	0
Acute	Acute - Me	East of Engl	Other	0	1	0	0	0
Acute	Acute - Me	East of Engl	Other	0	1	0	0	0
Acute	Acute - Me	East of Engl	Other	0	1	0	0	0
Acute	Acute - Larg	East of Engl	Other	1	0	0	0	0
Acute	Acute - Larg	East of Engl	Other	1	0	0	0	0
Acute	Acute - Larg	East of Engl	Other	1	0	0	0	0
Acute	Acute - Larg	East of Engl	Other	1	0	0	0	0
Acute	Acute - Me	North West	Other	0	1	0	0	0
Acute	Acute - Me	North West	Other	0	1	0	0	0
Acute	Acute - Me	North West	Other	0	1	0	0	0
Acute	Acute - Me	North West	Other	0	1	0	0	0
Acute	Acute - Larg	Midlands	Other	1	0	0	0	0
Acute	Acute - Larg	Midlands	Other	1	0	0	0	0
Acute	Acute - Larg	Midlands	Other	1	0	0	0	0
Acute	Acute - Larg	Midlands	Other	1	0	0	0	0
Acute	Acute - Tea	North West	Other	0	0	0	0	1
Acute	Acute - Tea	North West	Other	0	0	0	0	1
Acute	Acute - Tea	North West	Other	0	0	0	0	1
Acute	Acute - Tea	North West	Other	0	0	0	0	1
Acute	Acute - Larg	North East	Other	1	0	0	0	0
Acute	Acute - Larg	North East	Other	1	0	0	0	0
Acute	Acute - Larg	North East	Other	1	0	0	0	0
Acute	Acute - Larg	North East	Other	1	0	0	0	0
Acute	Acute - Tea	Midlands	Other	0	0	0	0	1
Acute	Acute - Tea	Midlands	Other	0	0	0	0	1
Acute	Acute - Tea	Midlands	Other	0	0	0	0	1
Acute	Acute - Tea	Midlands	Other	0	0	0	0	1
Acute	Acute - Larg	South East	Other	1	0	0	0	0
Acute	Acute - Larg	South East	Other	1	0	0	0	0
Acute	Acute - Larg	South East	Other	1	0	0	0	0
Acute	Acute - Larg	South East	Other	1	0	0	0	0
Acute	Acute - Larg	North East	Other	1	0	0	0	0
Acute	Acute - Larg	North East	Other	1	0	0	0	0
Acute	Acute - Larg	North East	Other	1	0	0	0	0

Acute	Acute - Larg	North East	Other	1	0	0	0	0
Acute	Acute - Tea	South East	Other	0	0	0	0	1
Acute	Acute - Tea	South East	Other	0	0	0	0	1
Acute	Acute - Tea	South East	Other	0	0	0	0	1
Acute	Acute - Tea	South East	Other	0	0	0	0	1
Acute	Acute - Larg	Midlands	Other	1	0	0	0	0
Acute	Acute - Larg	Midlands	Other	1	0	0	0	0
Acute	Acute - Larg	Midlands	Other	1	0	0	0	0
Acute	Acute - Larg	Midlands	Other	1	0	0	0	0
Acute	Acute - Tea	North West	Other	0	0	0	0	1
Acute	Acute - Tea	North West	Other	0	0	0	0	1
Acute	Acute - Tea	North West	Other	0	0	0	0	1
Acute	Acute - Tea	North West	Other	0	0	0	0	1
Acute	Acute - Tea	North West	Other	0	0	0	0	1
Acute	Acute - Tea	North West	Other	0	0	0	0	1
Acute	Acute - Tea	North West	Other	0	0	0	0	1
Acute	Acute - Tea	North West	Other	0	0	0	0	1
Acute	Acute - Larg	North East	Other	1	0	0	0	0
Acute	Acute - Larg	North East	Other	1	0	0	0	0
Acute	Acute - Larg	North East	Other	1	0	0	0	0
Acute	Acute - Larg	North East	Other	1	0	0	0	0
Acute	Acute - Mu	South East	Other	0	0	1	0	0
Acute	Acute - Mu	South East	Other	0	0	1	0	0
Acute	Acute - Mu	South East	Other	0	0	1	0	0
Acute	Acute - Mu	South East	Other	0	0	1	0	0
Acute	Acute - Larg	North West	Other	1	0	0	0	0
Acute	Acute - Larg	North West	Other	1	0	0	0	0
Acute	Acute - Larg	North West	Other	1	0	0	0	0
Acute	Acute - Larg	North West	Other	1	0	0	0	0
Acute	Acute - Me	Midlands	Other	0	1	0	0	0
Acute	Acute - Me	Midlands	Other	0	1	0	0	0
Acute	Acute - Me	Midlands	Other	0	1	0	0	0
Acute	Acute - Me	Midlands	Other	0	1	0	0	0
Acute	Acute - Tea	London	London	0	0	0	0	1
Acute	Acute - Tea	London	London	0	0	0	0	1
Acute	Acute - Tea	London	London	0	0	0	0	1
Acute	Acute - Tea	London	London	0	0	0	0	1
Acute	Acute - Larg	South East	Other	1	0	0	0	0
Acute	Acute - Larg	South East	Other	1	0	0	0	0
Acute	Acute - Larg	South East	Other	1	0	0	0	0
Acute	Acute - Larg	South East	Other	1	0	0	0	0

For peer review only

data_comm	trust_size	trust_size_	trust_size_	trust_size_	trust_size_	ratio_bed_	med	surg
0	2	0	1	0	0	4.92092	153.2234	149.7669
0	2	0	1	0	0	4.587515	159.836	149.447
0	2	0	1	0	0	4.646518	157.8064	151.4545
0	2	0	1	0	0	4.470925	160.5372	155.8037
0	3	0	0	1	0	3.480691	531.2163	691.222
0	3	0	0	1	0	3.476124	545.3632	730.8928
0	3	0	0	1	0	3.228791	576.9961	785.838
0					1		199.7705	199.2811
0	3	0	0	1	0	4.182489	217.3347	211.679
1	1	1	0	0	0	5.052839	52.04995	61.38334
1	1	1	0	0	0	3.679162	68.15411	61.51667
1	1	1	0	0	0	3.469612	72.27033	63.04
1	1	1	0	0	0	3.632115	68.3486	61.66417
0	3	0	0	1	0	2.664883	684.7393	571.7467
0	3	0	0	1	0	2.396019	701.7891	583.7718
0	3	0	0	1	0	2.315907	733.8377	609.1982
0	3	0	0	1	0	2.349986	740.962	618.1524
0	3	0	0	1	0	2.832375	385.0125	273.066
0	3	0	0	1	0	3.000165	412.0606	278.4927
0	3	0	0	1	0	3.023003	426.1491	284.5317
0	3	0	0	1	0	2.78523	444.8466	293.6941
0	1	1	0	0	0	2.44109	180.657	154.8475
0	1	1	0	0	0	2.097989	181.245	155.2979
0	1	1	0	0	0	2.212544	176.4937	162.1258
0	1	1	0	0	0	2.159809	181.6133	173.9289
0	1	1	0	0	0	5.342856	53.66979	53.6625
0	1	1	0	0	0	4.632087	55.26667	58.72916
0	1	1	0	0	0	4.123782	60.92709	58.73333
0	1	1	0	0	0			
0	1	1	0	0	0	4.18934	83.12766	74.82083
0	1	1	0	0	0	4.380791	78.98117	75.30833
0	1	1	0	0	0	3.981726	83.75765	73.04584
0	1	1	0	0	0	4.144195	83.97288	79.66042
0	2	0	1	0	0	2.770837	283.0372	272.0913
0	2	0	1	0	0	2.672849	293.8811	274.75
0	2	0	1	0	0	2.481482	312.8172	280.5544
0	2	0	1	0	0	2.350432	338.6611	300.8007
0	1	1	0	0	0	2.629664	128.5335	103.6368
0	1	1	0	0	0	2.158798	146.841	108.6426
0	1	1	0	0	0	2.1457	153.796	115.1837
0	1	1	0	0	0	2.08368	163.0529	130.0259
0	2	0	1	0	0	3.060407	189.1905	188.7192
0	2	0	1	0	0	3.07598	194.1658	183.8262
0	2	0	1	0	0	3.432787	198.6724	183.3641
0	2	0	1	0	0	3.095046	192.2427	191.3405
0	1	1	0	0	0	2.98681	164.808	152.9136
0	1	1	0	0	0	2.785593	179.495	156.9553
0	1	1	0	0	0	2.74235	183.5105	166.1571
0	1	1	0	0	0	2.326722	217.6882	203.3866

For peer review only

0	3	0	0	1	0	2.11235	462.5181	395.1882
0	3	0	0	1	0	2.097434	467.9527	392.8896
0	3	0	0	1	0	2.105486	473.9999	398.94
0	3	0	0	1	0	1.98168	479.3912	411.582
0	1	1	0	0	0	3.059	149.9673	136.2594
0	1	1	0	0	0	3.196917	151.7087	141.0739
0	1	1	0	0	0	3.162555	153.6732	142.1122
0	1	1	0	0	0	3.298766	154.0728	144.0414
0	1	1	0	0	0	4.503096	104.9833	121.1264
0	1	1	0	0	0	4.454136	103.8361	126.2818
0	1	1	0	0	0	4.254969	106.4638	129.9732
0	1	1	0	0	0	3.85008	114.4132	130.7456
0	1	1	0	0	0	3.649581	113.6432	134.7569
0	1	1	0	0	0	3.348313	126.9296	137.2225
0	1	1	0	0	0	3.01946	131.9772	141.414
0	1	1	0	0	0	2.988228	135.6992	149.3586
0	1	1	0	0	0	3.284315	138.8417	139.5371
0	1	1	0	0	0	3.173288	148.1114	137.4361
0	2	0	1	0	0	3.099729	162.1109	149.8029
0	2	0	1	0	0			
0	1	1	0	0	0	3.604817	82.11234	97.34322
0	1	1	0	0	0	3.133897	93.33427	93.46954
0	1	1	0	0	0	3.147946	93.47365	97.71835
0	1	1	0	0	0	3.225535	93.47287	97.68002
0	1	1	0	0	0	4.141535	111.1303	109.6668
0	1	1	0	0	0	3.660055	119.7386	109.7803
0	1	1	0	0	0	3.693597	116.4854	111.3403
0	1	1	0	0	0	3.989693	110.4722	118.9917
0	2	0	1	0	0	3.697407	178.0978	154.3894
0	2	0	1	0	0	3.670897	190.621	155.4977
0	2	0	1	0	0	3.964081	184.7213	157.0067
0	2	0	1	0	0	4.04621	177.2029	153.6628
0	2	0	1	0	0	4.424022	150.4852	184.9767
0	2	0	1	0	0	4.433776	155.5672	197.9577
0	2	0	1	0	0	3.467294	199.1467	220.5997
0	2	0	1	0	0	3.533089	198.4099	207.8976
0	1	1	0	0	0	6.170159	81.44036	112.7875
0	1	1	0	0	0	5.581248	86.85333	112.6005
0	1	1	0	0	0	5.586073	88.1657	117.3635
0	1	1	0	0	0	5.714702	88.45606	123.9896
0	1	1	0	0	0	4.109161	69.41807	84.13442
0	1	1	0	0	0	3.00864	89.65845	84.39519
0	1	1	0	0	0	2.873391	87.52725	88.33846
0	1	1	0	0	0	3.13771	83.34103	94.12933
0	1	1	0	0	0	4.458573	87.69621	97.51666
0	1	1	0	0	0	4.265711	94.18125	103.6833
0	1	1	0	0	0	4.20681	99.125	109.6167
0					1			
0	2	0	1	0	0	4.259778	141.1459	149.1449
0	2	0	1	0	0	4.23739	147.6735	167.366

0	2	0	1	0	0	4.115541	151.9242	171.0741
0	2	0	1	0	0	3.755153	169.6336	193.8411
0	3	0	0	1	0	5.146567	216.5035	229.5789
0	3	0	0	1	0	4.813731	225.7605	221.185
0	3	0	0	1	0	4.629898	235.1564	223.3567
0	3	0	0	1	0	4.259214	254.4483	236.773
0	1	1	0	0	0	3.250871	87.82261	89.35201
0	1	1	0	0	0	3.133527	92.06877	92.70142
0	1	1	0	0	0	3.04242	92.19634	93.14735
0	1	1	0	0	0	3.02824	93.28851	96.83882
0	1	1	0	0	0	4.224988	82.54462	67.43483
0	1	1	0	0	0	4.297314	81.27171	70.04677
0	1	1	0	0	0	3.722507	83.81448	72.86648
0	1	1	0	0	0	4.056291	83.2041	74.29073
0	1	1	0	0	0	5.584074	88.68974	98.92925
0	1	1	0	0	0	5.569273	92.15745	108.113
0	1	1	0	0	0	4.490548	112.9038	117.0263
0	1	1	0	0	0	4.014895	127.961	122.8104
0	2	0	1	0	0	3.420479	187.693	136.5974
0	2	0	1	0	0	3.049183	201.3654	138.2671
0	2	0	1	0	0	3.068923	206.913	147.265
0	2	0	1	0	0	3.120338	206.0674	148.9262
0	2	0	1	0	0	4.085379	139.7055	99.38074
0	1	1	0	0	0	3.65719	145.4669	98.89962
0	1	1	0	0	0	3.687423	147.7319	95.38072
0	2	0	1	0	0	3.67493	153.4723	101.3648
0	1	1	0	0	0	4.301551	115.1329	121.0833
0	1	1	0	0	0	3.944123	126.8977	125.2372
0	1	1	0	0	0	3.998712	129.2916	120.8643
0	1	1	0	0	0	4.038064	131.251	123.9338
0	2	0	1	0	0	3.566622	191.4276	157.2045
0	2	0	1	0	0	3.617311	197.6606	170.0311
0	2	0	1	0	0	3.541277	199.7161	171.1756
0	2	0	1	0	0			
0	2	0	1	0	0	5.154797	114.796	161.5306
0	2	0	1	0	0	5.246083	113.5133	161.2002
0	3	0	0	1	0	4.937619	217.1593	289.8549
0	3	0	0	1	0	4.633715	257.6227	338.6174
0	3	0	0	1	0	4.680759	280.7771	320
0	3	0	0	1	0	4.415216	295.2857	331.4306
0	3	0	0	1	0	4.208961	305.895	339.9729
0	3	0	0	1	0	4.227488	301.9524	361.0883
0	2	0	1	0	0	3.523005	190.4624	114.9074
0	2	0	1	0	0	3.336154	201.0549	119.085
0	2	0	1	0	0	3.360967	198.5292	121.9648
0	2	0	1	0	0	3.332517	200.8992	134.0909
0	1	1	0	0	0	3.673475	83.64016	47.96667
0	1	1	0	0	0	3.595294	81.77356	50.71667
0	1	1	0	0	0			
0	2	0	1	0	0	3.858436	180.1248	165.8808

For peer review only

0	2	0	1	0	0	2.85636	241.2161	173.3278
0	2	0	1	0	0	3.059576	224.7861	179.2539
0	2	0	1	0	0	3.260528	212.3889	190.4375
0	2	0	1	0	0	4.026048	194.7319	157.6954
0	2	0	1	0	0	5.005513	163.5197	185.1469
0	3	0	0	1	0	4.587319	188.1273	207.6972
0	3	0	0	1	0	3.78339	340.1711	318.7337
0	3	0	0	1	0	3.547431	262.232	258.1004
0	3	0	0	1	0	3.522411	250.4676	254.9127
0	3	0	0	1	0	3.531966	245.4016	266.1211
0	3	0	0	1	0	3.356654	258.2929	280.9357
0	1	1	0	0	0	4.247323	90.46875	92.81952
0	1	1	0	0	0	4.266841	95.44532	98.6844
0	1	1	0	0	0	3.844457	104.5011	100.6703
0	1	1	0	0	0	3.747007	109.6876	102.0042
0	1	1	0	0	0	5.979303	79.39888	114.7308
0	1	1	0	0	0	6.571528	72.20543	113.6637
0	1	1	0	0	0	6.001255	75.4009	109.3932
0	1	1	0	0	0	5.336982	82.30307	111.3586
0	1	1	0	0	0	4.98591	92.56084	102.8867
0	1	1	0	0	0	4.307602	102.145	110.9162
0	1	1	0	0	0	3.575881	118.1527	116.5059
0	1	1	0	0	0	3.890921	117.7742	116.4412
0	2	0	1	0	0	4.501323	126.9627	149.3311
0	2	0	1	0	0	3.954152	193.4043	237.9455
0	2	0	1	0	0	3.883787	201.028	243.9483
0	3	0	0	1	0	4.11535	210.5532	246.5515
0	1	1	0	0	0	5.262692	82.22978	93.55833
0	1	1	0	0	0	5.126168	85.05378	97.00833
0	1	1	0	0	0	5.232019	82.85521	97.38229
0	1	1	0	0	0	4.916807	92.33636	101.8356
0	2	0	1	0	0	4.593118	127.9632	147.3808
0	2	0	1	0	0			
0	2	0	1	0	0			
0	1	1	0	0	0	3.609418	103.4793	103.9037
0	1	1	0	0	0	3.303105	113.2268	109.0884
0	1	1	0	0	0	2.950316	121.343	115.6991
0	1	1	0	0	0	2.768323	129.3202	120.9779
0	3	0	0	1	0	2.784472	362.9054	319.0535
0	3	0	0	1	0	2.567723	399.9653	333.2069
0	3	0	0	1	0	2.481422	415.9913	345.2268
0	3	0	0	1	0	2.347655	433.1982	371.6101
0	3	0	0	1	0	3.656903	228.2669	183.7044
0	3	0	0	1	0	3.332103	249.8422	198.185
0	3	0	0	1	0	3.156297	256.8675	203.7231
0	3	0	0	1	0	3.07054	264.7743	216.6538
0	3	0	0	1	0	2.888884	387.7796	373.7563
0	3	0	0	1	0	2.761539	394.798	359.0745
0	3	0	0	1	0	2.55622	423.3791	359.9188
0	3	0	0	1	0	2.436251	440.4309	365.8354

For peer review only

0	3	0	0	1	0	3.212155	492.6599	378.0803
0	3	0	0	1	0	3.083722	514.4757	393.8412
0	3	0	0	1	0	2.82987	532.71	407.0334
0	3	0	0	1	0	2.707785	540.0171	406.5959
0	3	0	0	1	0	3.129752	323.0288	222.7024
0	3	0	0	1	0	3.142035	321.209	227.5294
0	3	0	0	1	0	2.848764	354.4519	225.3278
0	3	0	0	1	0	2.78873	363.0685	236.2412
0	2	0	1	0	0	3.270126	204.4264	196.3917
0	2	0	1	0	0	3.034125	213.653	198.8125
0	2	0	1	0	0	2.825771	217.1089	198.7677
0	2	0	1	0	0	2.91238	209.1932	214.8153
0	3	0	0	1	0	1.610415	575.1622	410.1201
0	3	0	0	1	0	1.524868	631.8579	433.8001
0	3	0	0	1	0	1.546456	634.5154	462.7086
0	3	0	0	1	0	1.465847	658.493	465.813
0	3	0	0	1	0	4.219332	213.7779	207.6146
0	3	0	0	1	0	3.971804	226.0308	221.8716
0	3	0	0	1	0	3.857934	232.6375	232.5455
0	3	0	0	1	0	3.937913	230.0076	234.8776
0	1	1	0	0	0	3.669094	132.9347	117.225
0	1	1	0	0	0	3.500891	140.0358	123.2313
0	1	1	0	0	0	3.402944	141.3482	135.9167
0	1	1	0	0	0	3.215416	149.2809	137.8983
0	3	0	0	1	0	2.522369	357.6003	277.2323
0	3	0	0	1	0	2.390247	381.7597	289.3033
0	2	0	1	0	0	2.170256	391.4285	305.0761
0	3	0	0	1	0	2.110214	397.4716	319.4106
0	1	1	0	0	0	4.532524	106.0667	92.62952
0	1	1	0	0	0	4.882907	98.81409	96.7424
0	1	1	0	0	0	4.378387	105.4041	102.823
0	1	1	0	0	0	3.848592	117.1208	106.4206
0	3	0	0	1	0	4.458309	301.3474	305.3677
0	3	0	0	1	0	4.291972	324.0935	307.1
0	3	0	0	1	0	4.056757	341.1592	318.6501
0	3	0	0	1	0	3.868722	355.2853	331.6137
0	1	1	0	0	0	5.441925	84.52892	105.4114
0	1	1	0	0	0			
0	1	1	0	0	0			
0	2	0	1	0	0	5.167768	129.5047	173.14
0	2	0	1	0	0	5.397005	122.1511	171.5898
0	2	0	1	0	0	5.04438	136.5877	181.9843
0	2	0	1	0	0	4.435996	155.5457	197.3479
0	1	1	0	0	0	4.649181	71.89438	78.90163
0	1	1	0	0	0	4.971731	66.07357	77.24496
0	1	1	0	0	0	5.178083	63.73016	77.98883
0	1	1	0	0	0	5.242523	63.85285	72.42917
0	1	1	0	0	0	4.501411	110.7431	131.8159
0	1	1	0	0	0	4.609562	111.9412	138.6511
0	1	1	0	0	0	4.280515	115.8739	142.2102

For peer review only

0	1	1	0	0	0	4.084366	115.685	151.6095
0	3	0	0	1	0	2.681105	511.0765	490.8017
0	3	0	0	1	0	2.6234	543.6647	490.6735
0	3	0	0	1	0	2.510592	564.0104	504.566
0	3	0	0	1	0	2.559645	572.8334	526.661
0	2	0	1	0	0	4.572596	127.0066	132.0625
0	2	0	1	0	0	3.946041	140.5839	137.975
0	2	0	1	0	0	3.867944	148.7871	141.6858
0	2	0	1	0	0	3.647478	155.724	149.1443
0	3	0	0	1	0	3.833233	219.984	264.7877
0	3	0	0	1	0	3.130089	270.2799	268.5101
0	3	0	0	1	0	3.171129	266.4666	269.3822
0	3	0	0	1	0	3.088962	274.2831	285.3295
0	3	0	0	1	0	4.043414	282.0018	287.9216
0	3	0	0	1	0	3.820254	296.1845	300.365
0	3	0	0	1	0	3.848622	296.7296	304.0153
0	3	0	0	1	0	3.656776	312.5704	314.1197
0	1	1	0	0	0	2.189529	127.1963	110.7216
0	1	1	0	0	0	2.553775	112.089	106.1756
0	1	1	0	0	0	2.736732	104.8696	107.2554
0	1	1	0	0	0	2.582951	107.1449	109.5308
0	3	0	0	1	0	3.56719	247.8842	219.722
0	2	0	1	0	0	3.150757	273.8231	223.9695
0	3	0	0	1	0	2.80003	311.6931	232.5925
0	3	0	0	1	0	2.741111	320.8554	256.8637
0	2	0	1	0	0	3.872464	156.8769	177.6911
0	2	0	1	0	0	3.837989	154.7685	177.3293
0	2	0	1	0	0			
0	1	1	0	0	0	4.334514	59.57993	90.7125
0	1	1	0	0	0	3.910747	63.415	95.60834
0	1	1	0	0	0	3.600592	69.29416	101.1365
0	1	1	0	0	0	3.192866	76.65526	107.1729
0	1	1	0	0	0	4.206021	70.0781	74.05121
0	1	1	0	0	0	4.393933	65.94319	71.06
0	1	1	0	0	0	4.471569	67.76146	71.60625
0	1	1	0	0	0	4.225547	73.95493	74.40945
0	3	0	0	1	0	4.193359	246.8785	274.5881
0	3	0	0	1	0	3.44678	280.6968	287.8938
0	3	0	0	1	0	3.373646	290.7092	307.4114
0	3	0	0	1	0	3.328872	308.4379	319.0217
0	2	0	1	0	0	3.048222	225.0492	267.3065
0	2	0	1	0	0			
0	2	0	1	0	0	2.503916	279.4623	190.0364
0	2	0	1	0	0	2.389453	308.3342	190.0973
0	2	0	1	0	0	2.31194	326.5655	215.7953
0	2	0	1	0	0	2.271448	335.3588	231.7453
0	2	0	1	0	0	4.617182	120.0949	149.7182
0	2	0	1	0	0	4.549065	121.5635	147.7429
0	2	0	1	0	0	4.63695	115.9706	157.7369
0	2	0	1	0	0	4.843735	110.5036	165.3565

0	1	1	0	0	0	5.744209	81.08167	90.33041
0	1	1	0	0	0	5.371823	79.95424	91.90859
0	1	1	0	0	0	4.455378	92.30418	94.44138
0	1	1	0	0	0	4.437962	90.18779	100.6935
0	1	1	0	0	0	2.990061	189.1266	143.4131
0	2	0	1	0	0	3.180417	192.742	146.4959
0	2	0	1	0	0	2.990432	205.6559	147.8636
0	2	0	1	0	0	2.886748	223.6947	151.5325
0	2	0	1	0	0	4.032917	186.1556	211.5222
0	2	0	1	0	0	3.766166	194.7604	216.6797
0	2	0	1	0	0	3.549377	203.2751	220.743
0	2	0	1	0	0	3.504696	208.0066	219.3044
0	1	1	0	0	0	6.026771	91.09356	111.5091
0	1	1	0	0	0	5.61115	94.76667	116.9008
0	1	1	0	0	0	5.091724	104.1396	119.9708
0	1	1	0	0	0	4.693543	109.2458	123.3977
0	2	0	1	0	0	6.414288	90.89084	119.8889
0	2	0	1	0	0	5.347253	105.6617	120.558
0	2	0	1	0	0	4.663604	121.5262	138.7894
0	2	0	1	0	0			
0	1	1	0	0	0			
0	1	1	0	0	0			
0	1	1	0	0	0			
0	1	1	0	0	0	3.478399	91.85259	85.98042
0	2	0	1	0	0	5.293217	118.4063	129.5067
0	2	0	1	0	0	5.04988	125.3	131.5917
0	2	0	1	0	0	4.388969	135.795	129.1848
0	1	1	0	0	0	3.938726	140.9085	142.1075
0	2	0	1	0	0	5.499615	133.1002	156.6679
0	2	0	1	0	0	5.59577	134.7893	158.392
0	2	0	1	0	0	5.089925	144.9432	159.8073
0	2	0	1	0	0	4.707959	157.6055	158.1908
0	1	1	0	0	0	6.03047	79.63724	118.2097
0	1	1	0	0	0	5.532719	87.93146	126.3972
0	1	1	0	0	0	5.365339	91.55992	127.7414
0	1	1	0	0	0	5.333285	92.15708	131.7114
0	3	0	0	1	0	4.282682	190.243	159.2064
0	2	0	1	0	0	3.989397	193.325	159.8715
0	2	0	1	0	0	3.720683	203.0541	167.2479
0	2	0	1	0	0	3.639434	213.9069	173.7394
0	1	1	0	0	0	3.300239	150.2921	160.1381
0	1	1	0	0	0	2.881589	158.7665	157.5394
0	1	1	0	0	0	3.091465	145.9664	157.8291
0	1	1	0	0	0	3.372087	129.5933	155.2517
0	3	0	0	1	0	2.885572	302.3664	208.0434
0	2	0	1	0	0	2.767156	301.1214	201.5227
0	2	0	1	0	0	2.745413	296.0392	198.1106
0	2	0	1	0	0			
0	1	1	0	0	0	3.995273	126.7748	187.2119
0	1	1	0	0	0	3.984258	133.7138	194.7372

For peer review only

0	1	1	0	0	0	4.131827	131.1163	187.1719
0	1	1	0	0	0			
0	2	0	1	0	0	2.501198	306.2532	281.7473
0	2	0	1	0	0	2.359914	333.5927	278.0348
0	2	0	1	0	0	2.231572	344.264	290.4897
0	2	0	1	0	0	2.135916	358.6284	296.7248
0	1	1	0	0	0			
0	1	1	0	0	0	5.20869	95.89744	119.6406
0	1	1	0	0	0	4.584063	115.618	129.7721
0	1	1	0	0	0	3.650421	125.3965	132.7931
0	1	1	0	0	0	3.619937	127.5575	132.8258
0	1	1	0	0	0	2.396457	131.3397	110.1599
0	1	1	0	0	0	2.134809	133.1501	110.7635
0	1	1	0	0	0	1.765194	130.5806	118.9216
0	1	1	0	0	0	1.640425	130.454	119.9154
0	3	0	0	1	0	3.356749	402.4728	320.5654
0	3	0	0	1	0			
0	1	1	0	0	0	5.12111	103.2979	94.3353
0	1	1	0	0	0	5.119639	103.5229	100.4895
0	1	1	0	0	0	4.400496	118.2253	103.7669
0	1	1	0	0	0	3.844393	129.6694	105.7925
0	3	0	0	1	0	3.325634	525.5389	518.1277
0	3	0	0	1	0	3.351422	538.8757	531.0593
0	3	0	0	1	0	3.143393	568.2554	525.2651
0	3	0	0	1	0	3.005992	587.7427	544.5735
0	2	0	1	0	0	4.71225	106.5839	209.4458
0	1	1	0	0	0	3.768758	127.2966	212.8333
0	1	1	0	0	0	3.975924	121.1668	217.7083
0	1	1	0	0	0	4.062437	118.1557	207.5407
0	3	0	0	1	0	2.707069	419.2726	302.762
0	3	0	0	1	0	2.511091	454.0855	349.3226
0	3	0	0	1	0	3.218507	801.8471	635.0239
0	3	0	0	1	0	3.081028	836.0847	649.9219
0	3	0	0	1	0	2.485552	336.2432	284.0528
0	3	0	0	1	0	2.235251	357.2306	304.6453
0	3	0	0	1	0	2.023265	383.7856	331.218
0	3	0	0	1	0	1.921228	408.8531	350.5445
0	3	0	0	1	0	3.577903	407.0122	475.3083
0	3	0	0	1	0	3.405271	415.0918	477.2842
0	3	0	0	1	0	3.35693	411.6856	480.7842
0	3	0	0	1	0	3.107102	433.523	495.5747
0	3	0	0	1	0	3.854298	248.1645	218.0955
0	3	0	0	1	0	3.608443	258.976	217.9635
0	3	0	0	1	0	3.412453	273.7767	226.0936
0	3	0	0	1	0	3.250233	287.5178	230.3731
0	3	0	0	1	0	3.779374	231.8506	130.2354
0	3	0	0	1	0	3.672126	225.2101	129.6181
0	3	0	0	1	0	3.774882	208.682	141.2614
0	3	0	0	1	0	3.6814	215.7467	139.3722
0	3	0	0	1	0	3.717758	263.0617	256.6333

For peer review only

0	3	0	0	1	0	3.367957	281.5505	256.8043
0	3	0	0	1	0	3.379773	361.7847	349.1134
0	3	0	0	1	0	3.308232	404.219	393.9001
0	3	0	0	1	0	2.64035	443.0283	482.1257
0	3	0	0	1	0	2.560898	438.4204	506.3917
0	3	0	0	1	0	2.314095	454.7134	503.2606
0	3	0	0	1	0	2.130508	483.6875	521.0495
0	1	1	0	0	0	3.383234	141.3588	146.5597
0	1	1	0	0	0	3.245841	144.1845	158.3843
0	1	1	0	0	0	3.334547	140.4989	161.9612
0	1	1	0	0	0	3.270429	150.0568	166.1769
0	2	0	1	0	0	3.96941	159.4066	154.0381
0	2	0	1	0	0	3.622458	177.9179	154.0071
0	2	0	1	0	0	3.657653	178.3931	157.3972
0	2	0	1	0	0	4.014032	172.7689	157.3285
0	3	0	0	1	0	3.592083	252.4302	287.1207
0	3	0	0	1	0	3.160538	280.6485	290.5484
0	3	0	0	1	0	2.815516	301.8097	297.2246
0	3	0	0	1	0	2.614561	317.3573	307.8702
0	2	0	1	0	0	6.437484	114.3304	171.4549
0	2	0	1	0	0	5.325983	128.0984	174.3505
0	2	0	1	0	0	4.530112	148.2303	175.6124
0	2	0	1	0	0	4.165483	171.2887	183.6396
0	3	0	0	1	0	3.848692	243.4593	304.636
0	3	0	0	1	0	3.751949	248.1377	307.5727
0	2	0	1	0	0	3.317065	257.3058	316.0957
0	2	0	1	0	0	2.960377	278.6808	325.7808
0	3	0	0	1	0	4.380985	186.1453	171.608
0	3	0	0	1	0	4.033163	208.0873	175.9944
0	3	0	0	1	0	3.5445	229.5811	189.9879
0	2	0	1	0	0	3.152775	244.943	200.2584
0	3	0	0	1	0	3.26922	299.3833	255.9915
0	3	0	0	1	0	3.083758	304.4175	256.6448
0	3	0	0	1	0	3.276282	295.8384	268.3521
0	3	0	0	1	0	3.121272	302.601	294.7721
0	2	0	1	0	0	4.248409	149.4677	131.6333
0	2	0	1	0	0	4.007462	150.2198	136.3354
0	2	0	1	0	0	3.979213	145.8203	128.6053
0	2	0	1	0	0	4.011031	144.2896	131.9916
0	1	1	0	0	0	4.268801	84.97702	92.43769
0	1	1	0	0	0	4.547656	83.39462	83.19735
0	1	1	0	0	0	4.508204	82.84895	91.43751
0	1	1	0	0	0	4.526103	84.01268	93.65915
0	3	0	0	1	0	3.800895	298.4166	418.646
0	3	0	0	1	0			
0	3	0	0	1	0	3.959722	276.3452	292.4611
0	3	0	0	1	0	3.878502	277.8135	300.3955
0	3	0	0	1	0	4.205768	257.6224	280.9233
0	3	0	0	1	0	4.277626	262.4704	287.9814
0	3	0	0	1	0	3.133323	323.5383	296.2399

For peer review only

0	3	0	0	1	0	3.071476	324.7625	292.0587
0	3	0	0	1	0	2.69966	366.4351	295.0107
0	3	0	0	1	0	2.552902	390.3401	320.328
0	3	0	0	1	0	4.7162	223.9091	269.8207
0	3	0	0	1	0	4.750546	225.553	263.6938
0	3	0	0	1	0	5.493843	223.2954	260.0144
0	3	0	0	1	0	5.521955	222.6114	256.7703
0	3	0	0	1	0	2.760256	522.5059	482.293
0	3	0	0	1	0	2.651355	546.6074	482.1799
0	3	0	0	1	0	2.712675	559.5953	480.9851
0	3	0	0	1	0	2.724138	571.6487	487.5023
0	2	0	1	0	0	3.812528	212.7197	211.4878
0	2	0	1	0	0	3.62874	221.3579	202.2745
0	2	0	1	0	0	3.30825	227.3105	197.7466
0	2	0	1	0	0	3.245755	227.9131	219.5017
0	2	0	1	0	0	3.695148	182.5367	163.0559
0	2	0	1	0	0	3.288094	204.8299	175.2157
0	2	0	1	0	0	3.181695	210.0296	177.128
0	2	0	1	0	0	3.020592	218.0864	183.1924
0	2	0	1	0	0	2.483476	246.9321	200.7931
0	2	0	1	0	0	2.723236	253.2832	209.6463
0	2	0	1	0	0	2.62524	257.2146	221.1637
0	2	0	1	0	0	2.53338	261.0149	234.6791
0	2	0	1	0	0	5.043093	129.7319	140.642
0	2	0	1	0	0	4.476351	141.6332	145.0924
0	2	0	1	0	0	4.212382	144.8705	147.2617
0	2	0	1	0	0	4.024273	147.48	147.8025
0	2	0	1	0	0	4.581381	151.6464	178.5902
0	2	0	1	0	0	4.185483	165.3931	184.8937
0	2	0	1	0	0	4.353886	164.6231	194.1713
0	2	0	1	0	0	3.924596	184.9872	208.3357
0	2	0	1	0	0	5.166548	111.8735	122.4482
0	2	0	1	0	0	5.349274	104.8273	120.9191
0	2	0	1	0	0	5.237173	103.7296	117.9203
0	2	0	1	0	0	5.00797	107.1292	128.0827
0	2	0	1	0	0	4.537814	155.9672	164.9265
0	2	0	1	0	0	4.094433	169.9258	165.7422
0	2	0	1	0	0	3.683034	174.5843	164.4593
0	2	0	1	0	0	3.429924	181.5638	176.3718
0	3	0	0	1	0	3.11159	467.2048	456.8909
0	3	0	0	1	0	2.981804	500.4522	458.28
0	3	0	0	1	0	2.882585	513.1679	485.5585
0	3	0	0	1	0	2.768518	547.4049	514.3347
0	2	0	1	0	0	4.525129	151.8741	166.5739
0	2	0	1	0	0	4.18974	163.4946	169.1036
0	2	0	1	0	0	3.733215	174.0457	166.7868
0	2	0	1	0	0	3.583623	183.334	169.592
0	3	0	0	1	0	5.302072	211.1439	228.6928
0	3	0	0	1	0	5.116661	222.3618	233.9364
0	3	0	0	1	0	4.482142	233.3148	244.8172

For peer review only

0	3	0	0	1	0	4.540498	226.4069	250.0376
0	2	0	1	0	0	2.100772	381.6455	308.5611
0	2	0	1	0	0	2.070528	380.0963	296.8946
0	2	0	1	0	0	2.14337	371.1446	304.369
0	3	0	0	1	0	2.273958	363.1334	311.3148
0	2	0	1	0	0	3.18314	219.2018	245.4879
0	2	0	1	0	0	2.996321	216.2653	235.5396
0	2	0	1	0	0	2.861852	213.4981	237.9472
0	2	0	1	0	0	2.815753	229.1572	264.5434
0	2	0	1	0	0	4.975392	153.606	145.1904
0	2	0	1	0	0	4.664199	157.9049	150.1191
0	2	0	1	0	0	3.699483	174.5514	151.1666
0	2	0	1	0	0	3.648354	172.6121	152.9228
0	2	0	1	0	0	3.790518	222.6609	227.2279
0	2	0	1	0	0	3.763866	218.3261	238.1348
0	2	0	1	0	0	3.664839	214.7434	247.6246
0	2	0	1	0	0	3.438221	232.8966	254.1138
0	3	0	0	1	0	4.484759	207.4805	199.9755
0	3	0	0	1	0	4.203565	204.172	207.884
0	3	0	0	1	0	4.118693	207.2259	212.5798
0	3	0	0	1	0	3.950412	210.1679	218.1194
0	2	0	1	0	0	3.670139	187.7994	192.7616
0	2	0	1	0	0	3.411345	192.8125	200.3089
0	2	0	1	0	0	3.307045	188.5369	206.5
0	1	1	0	0	0	3.047934	188.8985	213.3792
0	3	0	0	1	0	5.63221	167.2967	212.5833
0	3	0	0	1	0	5.370132	177.6027	214.3135
0	3	0	0	1	0	4.987791	190.8661	218.2808
0	3	0	0	1	0	4.729073	201.9952	230.3526
0	2	0	1	0	0	4.872738	140.6294	177.1825
0	2	0	1	0	0	4.765455	144.5297	177.4278
0	2	0	1	0	0	4.209813	155.1732	185.4848
0	2	0	1	0	0	3.897094	173.3984	194.5987
0	3	0	0	1	0	1.858991	555.8122	370.5374
0	3	0	0	1	0	1.740668	572.4815	393.5145
0	3	0	0	1	0	1.847148	573.993	441.4177
0	3	0	0	1	0	1.910805	561.8052	479.4274
0	3	0	0	1	0	3.967742	242.6443	192.7806
0	3	0	0	1	0	3.993282	240.2159	192.1783
0	3	0	0	1	0	4.165196	226.58	195.4133
0	3	0	0	1	0	4.26356	220.5903	206.1094

For peer review only

ratio_bed_	others	ratio_bed_	science_stt	ratio_bed_	science_stt	ratio_bed_	nurse_supp	ratio_bed_
5.034492	131.5508	5.731625	253.9086	2.969573	156.1759	4.82789	767.3861	0.982556
4.906423	131.1962	5.58896	264.3629	2.77365	161.4467	4.541748	784.8587	0.934245
4.841389	133.3668	5.497996	234.0774	3.132511			765.5219	0.957843
4.606757	135.8021	5.285265	212.5274	3.377212			832.9024	0.861746
2.674973	628.7348	2.940827	1527.136	1.210763	638.1895	2.897259	2356.747	0.784556
2.593746	656.7365	2.886622	1632.994	1.160905			2481.445	0.76397
2.370718	692.3065	2.691005	1764.378	1.055896			2639.933	0.7057
	182.9256		450.1674				1102.197	
4.294238	191.605	4.744134	473.0751	1.921471			1134.579	0.801179
4.284551	56.01875	4.694856	166.5547	1.579061	86.5089	3.04015	366.9131	0.716791
4.076131	54.669	4.586695	161.3797	1.553789	87.54669	2.864186	366.3778	0.684403
3.977633	55.0065	4.558552	161.8249	1.549515			378.9298	0.661732
4.025839	56.9065	4.362419	167.9803	1.477852			414.9955	0.598199
3.191536	678.0547	2.691155	1040.917	1.753021	455.3889	4.007015	1594.065	1.144715
2.880406	724.6716	2.320361	1010.705	1.663691	411.8388	4.082908	1554.455	1.08173
2.789732	762.9307	2.227594	1059.172	1.604555			1554.117	1.093547
2.816862	793.3914	2.194692	1124.966	1.547825			1593.268	1.09288
3.993541	314.5375	3.466996	365.8756	2.980521	148.6877	7.334167	1043.989	1.044551
4.439075	322.2816	3.835931	377.1599	3.277787	159.2139	7.764712	1050.12	1.177247
4.527616	333.709	3.8604	377.5288	3.412322			1085.881	1.186364
4.218675	358.9683	3.451559	366.7739	3.378103			1088.762	1.13799
2.847963	140.2721	3.143889	280.2904	1.573368	92.19254	4.783468	315.7201	1.396807
2.44852	142.5135	2.668169	281.5034	1.350783	91.98575	4.133792	328.2061	1.158571
2.408624	152.7024	2.557262	290.1176	1.346006			365.3978	1.068698
2.255232	163.2482	2.402783	309.5375	1.267213			400.3864	0.979679
5.343583	39.69133	7.224499	80.33313	3.569511	28.89422	9.92413	209.3122	1.369963
4.358993	43.31216	5.91058	84.65424	3.024066	27.42533	9.334437	235.5773	1.086692
4.277809	46.90957	5.356051	77.73279	3.232227			252.5869	0.994707
			76.40971	3.471418			249.6824	1.06235
4.654452	67.81055	5.135631	85.80061	4.058829	56.42593	6.171807	327.6093	1.063004
4.594445	69.38902	4.98638	87.26833	3.964783	58.42454	5.922169	376.2832	0.91952
4.565626	71.25037	4.680677	90.9744	3.665866			348.6675	0.956499
4.368544	76.67792	4.538465	88.28596	3.941737			349.9902	0.994314
2.882305	330.718	2.371356	610.6422	1.284304	239.4063	3.275813	1163.059	0.6743
2.858963	344.834	2.277908	641.231	1.224988	253.885	3.093921	1147.23	0.684693
2.766844	350.9257	2.212007	646.1527	1.201342			1156.685	0.671099
2.646271	371.549	2.142382	670.4466	1.187268			1183.697	0.672469
3.261389	134.1867	2.518879	383.6163	0.881089	284.1304	1.189595	758.0861	0.44586
2.917826	127.1015	2.494071	420.3477	0.754138	287.6263	1.102125	800.984	0.395763
2.864989	137.371	2.402253	436.3973	0.756192			815.7766	0.404523
2.612941	134.3629	2.5286	450.302	0.754494			850.9042	0.399281
3.068051	174.5563	3.316981	257.3671	2.249705	76.52998	7.565662	579.896	0.998455
3.248993	176.855	3.37706	268.3353	2.22576	84.02554	7.107958	598.0553	0.998653
3.719376	179.4734	3.800007	277.142	2.460832			637.7733	1.069346
3.10964	186.8946	3.183612	286.2351	2.078711			688.8366	0.863775
3.219137	128.4553	3.832072	200.7321	2.452274	99.36301	4.954057	622.982	0.790151
3.185621	136.0403	3.675381	210.5696	2.374511	102.1537	4.894585	650.4188	0.768736
3.02876	148.2691	3.394167	223.0934	2.255782			682.3807	0.737491
2.490332	179.1623	2.827046	250.8095	2.019461			840.5948	0.60255

For peer review only

2.47224	451.2323	2.165182	562.3437	1.737372	287.6454	3.396543	986.8593	0.990009
2.498157	465.4626	2.108655	520.7152	1.884908	267.0669	3.675109	1030.031	0.952884
2.501629	479.8844	2.079668	495.4634	2.014276			1070.645	0.932149
2.308167	495.2058	1.918394	530.9712	1.789174			1124.277	0.844988
3.36674	131.7968	3.480737	152.8929	3.000467	62.65603	7.321722	384.7587	1.192306
3.437914	136.4928	3.553301	167.3658	2.897844	57.33281	8.459379	434.0137	1.117476
3.419833	141.8805	3.425418	175.5124	2.769035			460.4666	1.055451
3.5285	151.975	3.3443	168.0813	3.023834			456.9245	1.112328
3.902947	134.2989	3.520133	129.3503	3.654805	87.1039	5.427426	334.0911	1.415033
3.662443	127.5988	3.624641	95.65363	4.835154	55.6889	8.305067	343.1728	1.347718
3.485335	129.301	3.503452	95.41754	4.747555			381.7219	1.186728
3.369138	127.9577	3.442544	103.3081	4.263945			444.3308	0.991379
3.077765	120.6465	3.437729	142.0652	2.919434	54.29285	7.639127	332.1933	1.24852
3.097159	124.1728	3.422651	140.3396	3.028369	61.4062	6.921125	330.8607	1.284529
2.817967	128.9078	3.091358	146.4737	2.720624			348.5266	1.143385
2.714943	146.7221	2.763729	156.2399	2.595368			348.5242	1.163477
3.267949	124.4	3.665595	160.6292	2.838836	54.42395	8.378664	730.9009	0.623888
3.41977	123.4786	3.806328	168.3109	2.792451	85.8353	5.475603	787.2493	0.597016
3.354407	131.6511	3.816907	182.9248	2.747031			762.7737	0.65878
			191.7537	2.924324			836.8936	0.670037
3.040787	86.75833	3.411776	150.1034	1.971974	52.87771	5.597822	449.5458	0.658442
3.129362	90.47007	3.233114	152.6266	1.916441	63.60722	4.598535	477.2405	0.612899
3.011205	88.08395	3.340563	152.0627	1.935057			493.5092	0.59624
3.086609	94.02605	3.206558	150.9821	1.996926			519.1229	0.580787
4.196802	95.76392	4.80609	183.3175	2.510671	114.4723	4.020625	606.3392	0.759064
3.992065	89.55475	4.893654	192.3834	2.278003	105.6822	4.146866	563.4766	0.777761
3.864278	93.8531	4.584292	184.1837	2.335984			537.8483	0.799947
3.704041	91.78313	4.802081	180.0022	2.448581			514.2518	0.85707
4.265189	163.3068	4.032287	304.6831	2.161262	164.8264	3.995112	827.3378	0.795926
4.500066	159.8451	4.377677	312.9804	2.235763	170.6319	4.100934	863.4869	0.810377
4.663813	163.7184	4.472618	341.9034	2.141686			899.1032	0.814423
4.66606	170.7602	4.198872	337.0587	2.127226			912.5721	0.785691
3.599102	171.3431	3.88548	348.862	1.908348	205.8571	3.23404	688.9418	0.966337
3.48433	178.6804	3.860244	365.841	1.885382	216.8829	3.180288	729.1709	0.945937
3.130104	192.9967	3.577782	387.2424	1.783121			734.9133	0.939567
3.371852	188.3115	3.722556	402.1166	1.743276			765.6457	0.915567
4.455281	83.62542	6.008938	189.614	2.650121	138.7717	3.621056	591.5607	0.849448
4.305042	81.1634	5.97252	217.0561	2.233294	141.5522	3.424531	594.028	0.816039
4.196363	87.54948	5.625391	226.4088	2.175268			598.9271	0.822304
4.076955	90.80729	5.566734	215.0199	2.350946			634.9822	0.796085
3.390408	68.93896	4.137719	166.5461	1.712739	93.18189	3.061217	550.7749	0.517907
3.196272	70.26234	3.839183	169.2807	1.593507	90.31175	2.986876	505.4575	0.533675
2.847005	77.30046	3.253538	174.6387	1.440116			538.9139	0.466679
2.778093	86.76119	3.014021	175.8406	1.487142			581.6401	0.449591
4.009571	89.4125	4.372991	104.9612	3.725187	68.01097	5.749073	332.2473	1.176834
3.874779	97.71719	4.111354	106.9844	3.755222	66.87985	6.007041	374.5433	1.07264
3.804166	98.74558	4.222974	110.5161	3.773205			373.4994	1.116468
			107.417				408.5721	
4.031315	144.5848	4.158459	188.6596	3.186958	75.15221	8.00043	555.5481	1.082265
3.738811	153.7908	4.068838	206.8228	3.025536	81.88666	7.64166	576.3778	1.08566

3.654848	151.352	4.131099	214.6592	2.912756			583.6066	1.071355
3.286197	176.0276	3.618751	237.3699	2.683575			647.0422	0.98448
4.853452	181.465	6.140302	405.0389	2.75097	308.9698	3.60634	1237.2	0.900622
4.913307	192.7182	5.639064	418.9027	2.594278	333.9402	3.254325	1214.085	0.895119
4.874491	196.9396	5.528344	434.9514	2.503153			1245.427	0.874198
4.577169	209.5473	5.171863	467.438	2.318489			1249.882	0.867082
3.195228	88.33075	3.23217	173.8542	1.64218	59.16993	4.825086	573.7653	0.49759
3.112142	92.08525	3.132967	174.3248	1.654957	56.93327	5.067336	581.7409	0.495925
3.011358	94.16673	2.978759	170.0068	1.649934			631.4415	0.444222
2.917218	97.17259	2.907199	167.4106	1.687467			664.5291	0.425113
5.17166	70.52281	4.945209	131.3776	2.654562	71.77921	4.85865	331.8246	1.051007
4.985954	70.21056	4.974323	162.2058	2.153129	116.88	2.988107	368.6687	0.947327
4.281804	73.01058	4.273353	179.0854	1.742186			374.6787	0.832714
4.542963	70.90631	4.759802	193.9105	1.740494			407.8817	0.827446
5.006103	82.06308	6.034992	124.894	3.965363	43.174	11.47103	418.5362	1.183291
4.747349	89.98766	5.703559	126.6161	4.053591	43.21534	11.87657	440.815	1.164321
4.332358	99.06246	5.117983	133.4895	3.798052			443.0047	1.144457
4.183277	101.9171	5.040863	136.061	3.775879			505.6916	1.015936
4.699942	160.4576	4.001056	266.1	2.412627	121.9895	5.262747	710.2849	0.903863
4.440679	153.8887	3.989897	278.9987	2.200727	172.9497	3.550165	700.1007	0.877017
4.311955	156.0209	4.069967	283.1009	2.243017			732.5315	0.866857
4.317575	160.9593	3.994799	275.3088	2.335559			768.7236	0.836451
5.743065	118.9634	4.797696	247.6232	2.304913	164.4118	3.471466	536.5826	1.063676
5.379191	121.3826	4.382835	250.0046	2.127961	161.5609	3.292876	555.3543	0.957947
5.711322	122.8087	4.435762	257.5873	2.114817			572.7457	0.95112
5.564064	132.4571	4.257982	256.024	2.202918			583.488	0.966601
4.090158	108.0438	4.583789	155.5623	3.183613	95.18	5.203299	434.4253	1.140012
3.996415	115.6667	4.327087	154.3268	3.243117	99.20601	5.045057	424.0078	1.180403
4.277524	121.9093	4.24086	163.8687	3.154965			441.851	1.170078
4.276478	121.5902	4.358905	165.7647	3.197302			450.1083	1.177494
4.343068	129.1542	5.286318	159.3246	4.285277	153.463	4.448954	721.2306	0.946646
4.205114	137.7083	5.192133	155.3245	4.603266	152.7833	4.67983	743.8654	0.961195
4.131721	138.3969	5.110303	160.9165	4.395138			724.8883	0.975668
			159.9792	4.392758			743.3051	0.945439
3.663394	126.6994	4.670504	181.1203	3.267166	77.5325	7.632283	485.7943	1.218108
3.694163	128.2523	4.643193	270.0126	2.205453	181.0066	3.289936	498.2603	1.195159
3.699265	256.6025	4.178642	479.4487	2.236423			1002.407	1.069675
3.525365	311.8684	3.827736	539.3474	2.213323			1167.795	1.022226
4.107031	309.0129	4.253059	371.3217	3.539384	303.383	4.331983	1221.898	1.075581
3.933705	308.3253	4.228488	380.3148	3.428082	305.4447	4.268367	1270.696	1.026012
3.787067	321.4124	4.005758	401.3562	3.207874			1300.06	0.990339
3.535146	327.3549	3.899438	391.9542	3.256758			1289.945	0.989577
5.839484	115.8256	5.793192	216.8555	3.094226	160.2016	4.188473	630.4504	1.064318
5.632533	118.4156	5.664372	231.1158	2.902225	168.8794	3.971769	641.4371	1.045699
5.470842	129.4683	5.153771	231.1143	2.887099			663.0313	1.006363
4.992882	139.4298	4.801701	233.1921	2.871023			689.284	0.971298
6.40549	50.86667	6.040301	167.305	1.836466	44.27056	6.940278	411.2078	0.747189
5.796911	51.55	5.703201	173.6116	1.693435	48.64436	6.043867	400.9002	0.73335
			168.9789	1.686601			380.6616	0.748696
4.189756	172.8426	4.020999	316.9502	2.192774	178.8464	3.886017	606.3982	1.146112

For peer review only

3.975127	182.8755	3.767591	329.8851	2.088606	187.4349	3.675942	599.4775	1.149334
3.836737	190.1203	3.617447	346.8823	1.982661			615.5951	1.117212
3.636364	192.4458	3.598415	342.8571	2.019792			644.3127	1.074789
4.97161	174.1165	4.502733	272.8446	2.873431	107.1634	7.315932	518.0262	1.513437
4.420815	164.5707	4.973545	274.404	2.982828	109.6528	7.46447	576.0942	1.420775
4.155087	194.4537	4.438075	284.3113	3.035405			683.9063	1.261869
4.037854	367.654	3.500574	608.0212	2.116702			1238.159	1.039446
3.604217	256.24	3.630385	341.1182	2.72706	235.4092	3.95163	746.6154	1.245956
3.460989	248.7031	3.547402	360.1041	2.449986	247.5778	3.563527	761.5136	1.158548
3.256976	243.1723	3.564346	361.3692	2.398516			780.5369	1.110454
3.086115	259.9837	3.334824	376.7544	2.301234			804.7745	1.077321
4.139754	93.25959	4.12022	195.4016	1.966463	111.5505	3.444629	389.4203	0.986723
4.126792	92.37177	4.408814	201.0649	2.025466	123.6733	3.292951	416.0023	0.978961
3.990749	89.72329	4.477656	209.9163	1.913858			464.871	0.864218
4.029247	93.33574	4.403458	219.4506	1.872859			500.7405	0.820784
4.137946	87.0235	5.455423	226.7593	2.09363	127.423	3.725778	581.0338	0.817078
4.174597	79.88083	5.940099	236.1945	2.008937	132.9069	3.570169	593.8619	0.799007
4.136455	85.93005	5.265911	233.2678	1.93983			586.4982	0.771528
3.944465	96.08086	4.57167	240.0852	1.829559			614.8958	0.714349
4.485518	97.165	4.749652	226.2942	2.039381	119.4458	3.863676	584.1325	0.79006
3.966957	102.5692	4.289788	254.2467	1.730603	122.0425	3.605301	639.995	0.687505
3.626425	105.4192	4.00781	222.6142	1.897903			717.3767	0.588951
3.935465	111.855	4.096822	211.4317	2.167367			672.09	0.681828
3.827067	128.7044	4.440408	245.8386	2.324696	154.2591	3.704806	552.5413	1.034312
3.213971	186.8315	4.093259	338.8247	2.257067	188.3584	4.06008	835.9764	0.914799
3.200474	194.2346	4.019623	349.2663	2.2354			905.2572	0.862462
3.514478	203.7966	4.251789	362.5729	2.389864			961.3323	0.901353
4.625456	83.25833	5.197678	126.7685	3.413703	63.82221	6.780556	362.6531	1.193289
4.494459	81.82501	5.328445	133.6061	3.263323	63.81776	6.831955	387.9223	1.123936
4.451528	86.05798	5.037302	139.2156	3.113876			443.1869	0.978143
4.458167	91.85046	4.942817	141.2533	3.214083			506.7783	0.895855
3.987968	139.0495	4.226912	221.2192	2.656868	151.397	3.882178	479.1669	1.226608
			218.5799	2.867372	167.5414	3.740866	539.1224	1.162538
			222.324	2.923661			563.8484	1.152792
3.594676	91.1983	4.095471	137.8067	2.710319	42.6911	8.748897	391.3036	0.954502
3.428412	96.63273	3.870324	158.6694	2.357102	64.57221	5.791966	413.8884	0.903625
3.094233	98.53948	3.633061	191.0244	1.874106			473.1329	0.756658
2.959219	107.29	3.336751	200.2138	1.788089			532.2051	0.672673
3.167181	366.9475	2.7538	846.5888	1.193614	470.3543	2.148381	882.6411	1.14486
3.082169	383.9116	2.675095	799.6749	1.284272	383.3107	2.679289	944.4517	1.087403
2.990064	416.8958	2.476039	804.4769	1.283132			1115.24	0.925586
2.736739	428.0216	2.376048	822.4005	1.236624			1173.091	0.866941
4.543984	164.8267	5.064411	311.5053	2.67973	182.8084	4.566257	1105.79	0.75489
4.200621	174.1561	4.780193	342.9588	2.427405	196.8555	4.228991	1285.676	0.647519
3.979667	189.074	4.288004	364.1674	2.226311			1339.25	0.605376
3.752532	195.1895	4.165183	378.525	2.147811			1327.393	0.612479
2.997274	411.6375	2.721448	596.9861	1.876509	249.4643	4.490622	1360.905	0.823165
3.036278	419.15	2.601098	605.504	1.800566	246.0413	4.431167	1497.98	0.727814
3.006928	441.1818	2.453071	641.1928	1.68787			1587.969	0.681531
2.933013	461.9302	2.322862	689.9873	1.555101			1744.581	0.615047

For peer review only

4.185619	377.4886	4.19218	1153.12	1.372364	402.8534	3.928228	1758.936	0.899692
4.028274	409.9676	3.869818	1184.563	1.339313	407.8499	3.889912	1850.74	0.857225
3.703628	445.6111	3.382995	1226.274	1.229334			1827.063	0.825095
3.596323	468.8192	3.119006	1240.072	1.179166			1950.533	0.749667
4.53969	236.3727	4.277144	531.2958	1.902895	347.4191	2.91003	841.7457	1.201075
4.435691	245.1121	4.117504	535.1211	1.886022	335.1223	3.011587	910.1838	1.108842
4.48125	243.6195	4.144784	531.7764	1.898824			961.3321	1.050365
4.285874	241.9547	4.184668	525.6537	1.926173			1018.152	0.994449
3.403912	186.3857	3.586648	260.3261	2.567933	145.3675	4.59869	561.4139	1.190743
3.26061	187.6257	3.455017	246.4603	2.630241	137.7892	4.704651	584.6089	1.108861
3.086518	186.1457	3.295805	269.474	2.276658			587.2701	1.044664
2.836158	186.8358	3.260884	281.8237	2.161813			633.6673	0.961467
2.258485	465.8314	1.98838	964.3126	0.960529	165.4721	5.597622	853.4712	1.085274
2.221069	502.7838	1.916331	1032.64	0.933046	175.9368	5.476399	975.4741	0.987725
2.120665	556.9806	1.761731	1117.057	0.878424			929.7397	1.055403
2.072183	570.0717	1.693208	1201.638	0.803278			1095.04	0.881475
4.34459	227.7125	3.961135	292.4642	3.084138	201.5602	4.475089	751.1909	1.20076
4.04626	242.713	3.698813	306.9799	2.924459	204.4895	4.390202	752.7678	1.192599
3.85946	258.1462	3.476713	329.2541	2.725858			805.02	1.114879
3.856263	270.0012	3.354615	362.2052	2.500654			891.1628	1.016369
4.160802	122.0563	3.996108	122.1854	3.991885	41.77755	11.67493	393.9227	1.238187
3.978293	126.7953	3.866468	123.0611	3.983793	46.20644	10.60999	416.9957	1.175672
3.538932	124.5292	3.862549	126.765	3.794423			446.8223	1.076491
3.480827	132.3662	3.626305	138.5556	3.464314			481.324	0.997249
3.253589	399.4005	2.258385	629.5252	1.432826	373.1023	2.417568	694.8481	1.298126
3.154129	391.7394	2.329355	664.1874	1.373859	408.8089	2.232094	692.743	1.317227
2.784551	390.2062	2.177054	717.3646	1.184196			651.1367	1.304642
2.62593	387.4265	2.164927	741.0565	1.13183			667.7656	1.256055
5.19003	91.22475	5.269952	122.9273	3.910849	38.16763	12.59575	582.0225	0.825999
4.987472	91.39584	5.279234	130.0231	3.710878	41.09055	11.74236	608.2029	0.793321
4.488294	94.26605	4.895718	137.0948	3.366285			663.1309	0.695941
4.235553	104.1961	4.325976	137.2514	3.28412			694.0912	0.64941
4.399615	326.3849	4.116306	585.2747	2.295503	340.0708	3.950648	1757.832	0.764294
4.52947	326.7592	4.256957	614.1881	2.264778	350.7763	3.96549	1757.425	0.791499
4.343322	343.2787	4.03171	632.2587	2.188978			1739.119	0.795805
4.144883	354.4103	3.878274	647.0368	2.1243			1683.559	0.816425
4.363856	82.90417	5.548575	125.2468	3.672747	46.33	9.928771	450.7139	1.020603
			131.3074	2.844471	47.04	7.940051	457.9481	0.815595
			134.2541	2.405885			463.5879	0.696739
3.86537	123.9133	5.400955	281.3727	2.378518	148.8194	4.49706	748.1285	0.894566
3.842012	119.9025	5.498219	278.2326	2.369421	156.3157	4.217426	710.0912	0.928402
3.786042	129.3089	5.328328	283.5529	2.429882			760.4299	0.906066
3.496364	137.9309	5.002504	280.7804	2.457437			792.2853	0.870898
4.236288	59.075	5.658062	132.8469	2.516055	91.04982	3.671067	385.1149	0.867923
4.252705	59.40313	5.530012	140.1877	2.343287	87.82587	3.740356	352.2262	0.932639
4.231375	64.61875	5.106877	138.85	2.376665			356.0876	0.926738
4.621757	65.07584	5.143999	135.2924	2.47427			364.466	0.918467
3.78179	120.6513	4.131741	202.9023	2.456847	124.2609	4.011722	625.4816	0.796986
3.721571	120.1839	4.293422	212.736	2.425542	131.6389	3.919815	637.8688	0.808944
3.487794	123.4901	4.016516	225.5422	2.199145			656.5751	0.755435

For peer review only

3.11656	133.4312	3.541152	234.7886	2.012449			750.1657	0.629861
2.791861	553.379	2.476151	744.7083	1.839982	223.8203	6.122099	1058.524	1.294491
2.906719	570.4678	2.500141	734.5649	1.941626	224.3319	6.357767	1160.802	1.228677
2.806372	613.7418	2.307159	746.0319	1.898042			1209.136	1.171084
2.784049	634.6567	2.310304	772.1453	1.89893			1235.325	1.186935
4.397539	103.0498	5.635624	239.9886	2.419906	150.2102	3.866249	637.9422	0.910349
4.020656	112.9052	4.913414	242.8354	2.284469	161.0583	3.444405	702.1929	0.790025
4.061803	121.8894	4.721494	251.0464	2.292405			709.9578	0.810612
3.808393	125.0521	4.542108	256.5986	2.213574			730.1226	0.777952
3.184627	305.9369	2.756287	444.5756	1.896753	260.324	3.239233	869.4228	0.969896
3.150719	320.7376	2.63767	454.9136	1.859694	262.0221	3.228735	953.3451	0.887402
3.136807	317.0205	2.665443	485.3859	1.740883			1016.287	0.831458
2.969374	332.0927	2.551245	512.0065	1.654764			1112.421	0.761627
3.96028	247.4855	4.60734	531.0156	2.1473	353.3777	3.226718	1001.245	1.138832
3.767083	260.3909	4.345389	568.0195	1.992009	380.8072	2.97132	1059.232	1.068227
3.75639	289.7911	3.94077	578.7891	1.973085			1094.1	1.043781
3.638741	298.0774	3.834574	586.3202	1.949447			1133.779	1.008133
2.515318	102.371	2.720497	246.9341	1.127832	126.6159	2.199566	349.7955	0.796179
2.696005	117.266	2.441032	245.8867	1.164154	147.7006	1.938042	357.2543	0.80125
2.675857	126.6727	2.265681	231.9016	1.237594			372.516	0.770437
2.526687	132.5735	2.087522	259.4509	1.066676			402.6852	0.687262
4.024404	194.0312	4.557257	443.1143	1.995535	196.3909	4.5025	1038.207	0.851709
3.852087	206.6582	4.174768	461.4062	1.869827	195.8381	4.405425	1033.867	0.834489
3.752271	227.5382	3.83562	561.8309	1.553403			1087.099	0.802825
3.423996	264.1857	3.329098	663.798	1.324951			1197.952	0.73417
3.418855	157.5156	3.85676	270.8679	2.242791	70.69998	8.592648	677.5749	0.89658
3.349701	152.7244	3.88936	265.2913	2.239048	79.82111	7.441641	677.0112	0.877386
			267.7282	2.220536			701.8199	0.847083
2.846906	69.74667	3.702686	122.3722	2.110365	85.47945	3.021194	464.0921	0.556463
2.593916	76.01688	3.262434	129.1051	1.920915	86.31126	2.873321	480.3313	0.51631
2.466964	80.77552	3.088807	135.6213	1.839682			483.2715	0.516273
2.283693	87.62813	2.793053	148.6165	1.646856			506.2993	0.48341
3.980354	55.92	5.270923	98.39945	2.995444	38.62667	7.630739	268.4389	1.098015
4.07754	58.31208	4.968953	95.66668	3.028745	60.02556	4.82711	259.5589	1.116317
4.231474	60.30521	5.024442	104.3556	2.903534			266.8217	1.13559
4.199735	58.50156	5.341738	111.7267	2.797004			280.5161	1.114018
3.770192	287.4005	3.602116	499.0161	2.074582	329.4584	3.142279	725.7688	1.426418
3.360614	304.264	3.179805	511.4811	1.891565	322.213	3.002672	765.6194	1.263683
3.190351	319.2867	3.071691	531.9171	1.843802			813.5024	1.20559
3.218433	329.7307	3.113905	556.6521	1.844509			960.886	1.068545
2.566343	214.1301	3.20366	396.3397	1.730839	118.2211	5.802685	793.3125	0.864729
			405.0348	1.639365	126.56	5.246523	814.4445	0.81528
3.68219	248.1078	2.820347	498.8864	1.402624	256.5146	2.727915	1041.503	0.671866
3.875646	251.1998	2.932925	578.4396	1.273685	286.0897	2.575242	1159.789	0.635245
3.498686	263.1055	2.869572	614.8261	1.22799			1215.821	0.62098
3.287014	281.149	2.709417	655.158	1.162697			1366.993	0.557245
3.703624	138.3042	4.009279	228.2133	2.429744	163.2474	3.396685	696.0075	0.796687
3.742989	140.3771	3.939389	234.7647	2.35555	166.8255	3.314841	775.3975	0.713183
3.409158	144	3.734375	225.1139	2.388791			800.4835	0.671782
3.236945	150.091	3.566171	235.2211	2.275519			854.2297	0.626588

For peer review only

5.156071	71.87682	6.479837	105.9509	4.395903	105.8039	4.402013	506.3695	0.919783
4.673122	73.65121	5.83154	96.68587	4.442221	109.5344	3.921141	586.6903	0.732073
4.354553	79.7333	5.15782	105.4744	3.89905			606.4733	0.678101
3.974936	86.75622	4.613502	122.8622	3.257715			619.8485	0.645722
3.943156	131.4085	4.303375	199.1147	2.840072	115.5161	4.895423	617.1171	0.916358
4.184419	128.2875	4.778332	193.7371	3.164082	136.5366	4.489637	706.4326	0.86774
4.15924	131.2374	4.686166	194.7559	3.1578			568.0709	1.082611
4.261462	129.9813	4.968021	200.0442	3.228036			628.2175	1.027908
3.549273	195.1669	3.846708	297.6764	2.522034	200.1315	3.751284	798.4385	0.940273
3.385181	195.5708	3.750559	307.9752	2.381685	219.5464	3.34098	869.0425	0.844032
3.268506	197.771	3.648159	307.54	2.346036			860.2626	0.838697
3.324147	207.9373	3.505864	316.2049	2.305467			951.2759	0.766339
4.923365	110.7083	4.958976	143.2783	3.831703	100.2451	5.476575	474.5176	1.156965
4.54873	116.4667	4.565684	155.1872	3.426506	105.3282	5.048507	501.7956	1.059694
4.419825	114.9167	4.614214	183.8945	2.883447			499.2903	1.062007
4.155263	117.15	4.376867	181.5789	2.823841			486.9635	1.052954
4.862835	99.23959	5.874672	189.4615	3.077142	97.74223	5.964668	439.6015	1.326201
4.686542	107.8167	5.240377	182.2965	3.099346	113.0649	4.997131	455.3004	1.240939
4.083524	123.6778	4.582471	195.8644	2.893583			473.1147	1.197913
			198.7715	2.983325			482.1232	1.229976
			223.4322		82.81006		521.4717	
			197.2265		101.3694		504.5327	
			207.0684				451.8233	
3.715962	79.2265	4.032742	269.3839	1.18604			648.0206	0.49304
4.839519	111.0708	5.642796	178.9623	3.502133	113.2263	5.535377	656.8336	0.954199
4.808435	116.3188	5.439794	187.5819	3.373194	119.2994	5.303885	679.8983	0.930654
4.613546	123.5354	4.824527	195.3014	3.051694			605.0422	0.985055
3.905494	132.2292	4.197259	199.3045	2.784683			606.1583	0.915602
4.672303	137.3215	5.330557	250.1296	2.926483	119.483	6.126397	596.8259	1.226488
4.761919	144.6134	5.215631	255.9943	2.946355	131.0335	5.756163	571.6243	1.319485
4.616498	152.6252	4.833736	255.157	2.891357			578.3384	1.275637
4.69054	161.0562	4.607087	263.7387	2.813391			630.1275	1.17754
4.062696	94.59716	5.076791	222.0194	2.163099	90.4219	5.311214	491.2317	0.977645
3.848977	95.42424	5.098285	226.0106	2.152554	90.06265	5.401795	463.3261	1.050016
3.845659	103.694	4.737495	235.9103	2.082359			480.4788	1.022418
3.731643	102.7363	4.784095	239.2634	2.054221			499.0966	0.984779
5.11757	142.7407	5.707903	298.7184	2.727486	316.4704	2.57449	818.3173	0.995641
4.824188	146.2082	5.275013	299.8487	2.57213	312.4912	2.468069	838.5726	0.919718
4.517247	142.0774	5.317526	299.0249	2.526546			859.6696	0.878826
4.480849	143.8141	5.413238	296.1536	2.628704			853.0284	0.912631
3.097328	122.4605	4.050287	179.085	2.769634	124.0654	3.997891	556.3272	0.891562
2.904036	130.2437	3.512647	163.1236	2.804622	125.0346	3.658987	580.6602	0.787896
2.859106	157.7521	2.860501	130.4802	3.458378			504.3284	0.894754
2.814784	168.8437	2.588193	137.5319	3.177445			504.4429	0.866302
4.193836	266.0548	3.2794	559.6029	1.559141	298.7777	2.920231	699.674	1.247009
4.134771	284.2915	2.93097	580.1115	1.436362	304.6311	2.735276	763.8962	1.09079
4.102508	278.7064	2.916151	574.2658	1.415286			786.5198	1.03335
			593.9087	1.379842			807.9488	1.014297
2.70549	175.2707	2.889816	215.1971	2.353656	125.8551	4.024468	542.7271	0.93325
2.735738	179.5524	2.967101	232.8726	2.287732	129.2794	4.120921	555.8952	0.958364

For peer review only

2.894398	185.9032	2.914151	253.134	2.140171			570.2024	0.950101
			247.9231	2.054064			620.5914	0.820588
2.718748	252.0303	3.039317	254.3274	3.011866	105.8464	7.236905	582.1888	1.315724
2.83148	262.6988	2.996778	250.589	3.141599	94.9633	8.290045	595.2461	1.322562
2.644672	280.3958	2.739877	249.0413	3.08483			610.7368	1.257907
2.581517	305.0961	2.510684	272.9992	2.805869			608.7942	1.258225
			86.34061	2.356944	23.5048	8.657808	220.8794	0.921317
4.175004	143.9742	3.469373	145.1581	3.441075	98.90054	5.050529	388.7173	1.284996
4.084082	146.9572	3.606494	162.857	3.254388	100.8278	5.256489	389.014	1.362419
3.447092	133.2574	3.435081	187.1961	2.445297			454.0984	1.008042
3.476357	137.4012	3.360598	192.5333	2.398286			475.602	0.970875
2.85721	113.9317	2.762621	183.1014	1.718993	162.6917	1.934641	371.6998	0.846785
2.56628	118.4508	2.399731	183.1432	1.552064	166.7956	1.704182	377.7373	0.752507
1.938252	118.2478	1.949297	193.5226	1.191076			376.5181	0.612188
1.784591	121.7043	1.75836	222.6407	0.96119			367.947	0.581605
4.214429	287.6437	4.696783	579.977	2.329403	285.382	4.734006	1478.499	0.913765
			561.7894	2.544993	290.9487	4.914097	1517.067	0.942443
5.607657	109.2704	4.8412	253.8262	2.084103	135.4726	3.904849	474.3737	1.115155
5.274185	120.2858	4.406174	266.9851	1.985129	149.9433	3.534669	512.6205	1.033903
5.01364	115.7284	4.495439	281.7585	1.84644			513.2556	1.013628
4.712052	116.876	4.265204	283.4167	1.758894			542.2344	0.919344
3.373203	532.9363	3.279472	1380.711	1.265833	537.3042	3.252813	1744.578	1.001818
3.40075	578.6558	3.121026	1456.716	1.239775	543.7561	3.321342	1928.866	0.936302
3.400664	603.7239	2.95872	1467.384	1.217303			2005.225	0.890798
3.244282	624.6881	2.828211	1524.342	1.159025			2110.571	0.837096
2.397995	113.8992	4.409599	148.7211	3.377126	64.0369	7.843134	927.8895	0.541282
2.254111	122.8088	3.906479	158.2183	3.032202	68.01012	7.054097	919.3227	0.521852
2.212823	128.8225	3.739641	159.9551	3.011784			926.9687	0.519705
2.312799	130.7754	3.670416	180.9468	2.652714			1029.534	0.46623
3.748819	335.2563	3.38547	614.9259	1.845751	219.7244	5.165563	954.0516	1.189663
3.264175	371.9689	3.065444	718.442	1.587115	264.9957	4.3029	1063.775	1.07189
4.06402	660.1438	3.909375	1304.16	1.97886			2584.345	0.998609
3.963553	689.3292	3.736966	1356.826	1.898549			2748.953	0.937084
2.942235	458.7734	1.821706	623.0534	1.341378	194.9255	4.287536	641.3234	1.303165
2.621081	477.1666	1.67342	622.635	1.282453	232.2832	3.437614	677.0219	1.17943
2.344378	487.9373	1.591393	647.1947	1.199794			731.3798	1.061692
2.240799	506.4007	1.551143	686.3687	1.144429			800.4732	0.981295
3.063801	443.9083	3.28052	1077.316	1.351739	482.6711	3.017065	1645.363	0.885063
2.961548	443.8594	3.184567	1113.05	1.269934	491.5387	2.875664	1661.29	0.850845
2.874471	467.362	2.957023	1154.149	1.19742			1673.617	0.825757
2.718056	480.9432	2.800746	1179.514	1.141996			1728.13	0.779455
4.385693	221.3425	4.321357	435.674	2.195449	298.507	3.20428	979.6696	0.97635
4.287415	225.2139	4.149389	453.1942	2.06203	293.6466	3.182397	956.2694	0.977235
4.132137	226.8564	4.118244	464.9894	2.009186			1000.978	0.933337
4.056462	240.4292	3.886799	466.4075	2.003613			1098.141	0.850984
6.728204	206.4318	4.244742	517.5736	1.692996	115.7014	7.573372	1032.425	0.84873
6.380283	201.7016	4.100116	543.1873	1.522495	123.3259	6.705808	996.9677	0.829515
5.576542	188.9237	4.169673	533.0158	1.477911			978.9583	0.804682
5.698772	196.9466	4.03282	538.8575	1.473952			1028.97	0.771889
3.810885	235.5383	4.152192	391.2446	2.499715	279.7479	3.496005	941.8235	1.038411

For peer review only

3.692501	253.3741	3.742491	412.0173	2.301481	296.2669	3.200661	953.9617	0.994013
3.502444	337.7755	3.620008	637.4808	1.918097			1330.495	0.919019
3.394897	412.198	3.244193	698.4919	1.914482			1556.29	0.859255
2.426234	451.3833	2.591478	892.8507	1.31013	354.8824	3.296162	1185.192	0.986971
2.217157	470.5131	2.386225	913.4795	1.229092	358.8872	3.128421	1286.728	0.872562
2.090865	484.2118	2.173119	925.9061	1.136454			1378.047	0.763581
1.977739	500.7673	2.057842	932.0624	1.105613			1418.605	0.726418
3.263176	136.4799	3.504179	147.6313	3.23949	135.9684	3.517361	399.6036	1.196811
2.954838	140.3584	3.334323	147.8813	3.164701	142.7139	3.279288	452.679	1.033845
2.892668	138.8344	3.374523	161.7396	2.896631			456.0186	1.027371
2.953178	139.5198	3.517421	191.1617	2.567198			492.5199	0.996406
4.107749	149.1998	4.240957	187.4558	3.375463	79.02553	8.006906	528.2484	1.197827
4.184873	156.7802	4.110852	200.3814	3.216366	78.09867	8.252381	553.7803	1.163819
4.145563	158.1851	4.124915	207.6614	3.142134			594.0468	1.098398
4.407976	163.581	4.239489	211.2633	3.282633			633.8441	1.094118
3.158079	235.3504	3.852765	456.9687	1.984272	225.6661	4.018104	1209.277	0.749828
3.052847	236.4497	3.751326	475.7259	1.864519	236.5606	3.749568	1219.266	0.727487
2.858949	243.6696	3.487305	464.5177	1.829317			1195.188	0.710976
2.695129	244.47	3.394077	475.6065	1.744615			1228.801	0.675252
4.292674	123.0756	5.980063	205.6424	3.579028	126.8111	5.803909	610.57	1.205431
3.913094	124.0608	5.499322	227.0678	3.004609	127.2204	5.36274	667.602	1.021941
3.823761	138.0428	4.864435	265.0973	2.533032			773.3754	0.868272
3.885329	159.1276	4.483825	274.3176	2.601			781.6047	0.912866
3.075802	275.4363	3.401876	555.9107	1.685522	312.9404	2.99418	973.2591	0.962745
3.026927	283.0344	3.289353	542.8901	1.714896	304.1288	3.061203	1000.775	0.930279
2.700131	293.4318	2.908683	551.2282	1.548361			1004.891	0.849346
2.532378	306.5106	2.691587	584.1362	1.412342			1029.894	0.801053
4.752109	192.7284	4.231343	268.6941	3.03505	157.6121	5.174094	605.5322	1.346749
4.768619	196.2179	4.277133	270.8178	3.098947	164.4779	5.102511	649.8552	1.291442
4.283167	209.4194	3.885743	282.7005	2.878488			657.6921	1.237281
3.856268	222.0312	3.478115	299.7995	2.575888			696.135	1.10934
3.823369	248.1758	3.943778	469.5513	2.084437	275.2747	3.55554	1021.931	0.957745
3.657779	249.7708	3.758446	486.7997	1.928411	266.3329	3.524724	1060.074	0.885551
3.611859	275.9345	3.512609	511.9483	1.893258			1135.83	0.853341
3.20417	307.5747	3.070799	534.5862	1.766787			1310.158	0.720906
4.824006	123.7252	5.132343	229.4484	2.767507	125.5821	5.056454	594.0951	1.068852
4.41558	125.6227	4.792127	231.2013	2.603792	130.1402	4.625781	603.5801	0.997382
4.511865	128.5176	4.514947	224.7857	2.581347			557.3438	1.041099
4.384749	130.0296	4.450909	219.3673	2.63827			561.8792	1.030026
3.924265	76.73333	4.727411	126.1899	2.874637	78.0389	4.648323	343.3651	1.056456
4.558438	77.71667	4.879906	124.9972	3.034068	78.65779	4.821518	313.8633	1.208329
4.084757	78.46187	4.760274	133.7221	2.793106			316.3428	1.180681
4.059935	77.21079	4.92483	140.9205	2.698331			341.5117	1.113432
2.709329	418.3576	2.711197	1053.396	1.076755	520.0883	2.18088	1401.824	0.809125
			1063.158	1.016312	512.726	2.107363	1446.134	0.747164
3.741523	295.9624	3.69726	411.2773	2.660613	263.5401	4.15212	1155.56	0.946943
3.586938	330.5662	3.259559	424.3776	2.539012	282.3185	3.816612	1232.022	0.874578
3.856925	307.9162	3.518814	439.3504	2.466141			1345.834	0.805077
3.89869	291.5356	3.85116	457.2776	2.455292			1426.32	0.787166
3.422058	254.6683	3.980668	486.437	2.084031	217.8	4.654501	949.7974	1.067333

For peer review only

3.415409	256.1649	3.893976	491.4993	2.029504	221.1663	4.510181	1001.451	0.996055
3.353268	241.2078	4.101235	514.3257	1.923392			969.091	1.020802
3.110874	253.652	3.928611	533.3244	1.868469			906.4429	1.099352
3.91371	218.693	4.828687	380.6374	2.774294	179.4479	5.884715	1194.323	0.884183
4.063426	218.4396	4.905246	377.2885	2.840001	180.1181	5.948874	1236.778	0.866364
4.718007	216.9607	5.65425	372.925	3.289536			1220.741	1.004922
4.787354	234.2029	5.248653	391.2415	3.141922			1323.433	0.928834
2.990402	536.5709	2.687902	791.5594	1.822036	391.395	3.684897	1600.514	0.901117
3.005621	550.0866	2.634585	836.6726	1.732159	411.8774	3.518644	1768.942	0.819275
3.156023	566.5844	2.679213	850.2164	1.785428			1841.213	0.824456
3.194344	578.6448	2.691202	856.1824	1.81883			1929.386	0.807122
3.834737	180.6316	4.489801	318.0845	2.549637	163.942	4.946871	593.8062	1.365766
3.971088	185.1658	4.338005	309.381	2.596314	168.7543	4.75988	588.1152	1.365804
3.802847	183.3468	4.101516	316.0427	2.379425			576.4985	1.304427
3.370134	185.2565	3.993113	330.0091	2.241605			637.0402	1.16123
4.136618	140.6153	4.796774	261.314	2.581186	146.3827	4.607783	539.0165	1.251353
3.843835	151.8665	4.434815	276.478	2.435999	146.6856	4.591452	587.1822	1.147003
3.772696	152.9652	4.368642	284.5224	2.348673			575.8369	1.160485
3.595946	155.9002	4.22546	283.8595	2.320691			580.6846	1.134437
3.054139	171.8306	3.568921	273.4038	2.24302	64.66302	9.483783	596.1362	1.028708
3.290066	173.7428	3.96995	301.3931	2.288539	133.6337	5.161496	596.9823	1.155394
3.053169	176.2875	3.830392	311.6213	2.166893			605.671	1.114879
2.817678	189.8354	3.483281	312.7357	2.114405			617.0324	1.071662
4.651882	122.2428	5.352052	243.7581	2.684014	91.76961	7.129267	714.3963	0.915808
4.369629	129.406	4.899309	250.238	2.533589	94.73264	6.692519	760.834	0.833296
4.143982	130.4757	4.677115	239.7487	2.545373			749.9716	0.813697
4.015494	135.1581	4.391152	243.4582	2.43779			768.0911	0.772695
3.890191	181.0536	3.837261	348.7072	1.992359	153.0603	4.539061	838.3082	0.828752
3.744042	187.2	3.697916	352.9565	1.96129	163.5369	4.232991	856.8987	0.807855
3.691327	193.9159	3.696189	362.0678	1.979602			854.2188	0.839071
3.48476	199.204	3.644506	370.3745	1.960178			928.8939	0.781575
4.720365	115.0693	5.023061	228.6747	2.527609	112.0959	5.1563	499.8144	1.156429
4.637398	118.0918	4.748424	227.2516	2.46753	107.4293	5.219712	518.8405	1.080775
4.606926	120.5617	4.505991	227.8011	2.384756			519.6838	1.045347
4.188701	125.3252	4.280862	232.8225	2.30433			548.6957	0.977773
4.291306	138.5249	5.109191	292.1395	2.422644	230.245	3.073899	735.0625	0.962843
4.197784	138.6198	5.019125	288.1879	2.414223	248.083	2.804505	704.3717	0.98776
3.909783	150.2718	4.278915	292.1452	2.200961			687.6707	0.935041
3.530894	154.0291	4.043068	308.1454	2.020961			727.6283	0.855863
3.181832	435.8038	3.33579	943.8096	1.5403	309.0111	4.704524	1438.522	1.010586
3.256197	446.7628	3.340139	995.7798	1.498574	302.5984	4.931454	1495.715	0.997683
3.046492	472.2833	3.132124	1033.337	1.431527			1648.014	0.897595
2.946525	476.5174	3.180367	1079.847	1.40344			1978.094	0.766142
4.125796	153.0357	4.490783	286.8227	2.39608	154.7111	4.44215	962.092	0.714329
4.050773	158.8422	4.312457	295.0605	2.321558	153.1795	4.471879	1045.784	0.655011
3.895693	169.3693	3.836293	295.1047	2.201761			1038.361	0.625746
3.874004	179.729	3.655503	299.1718	2.196063			1056.973	0.621586
4.895214	197.1831	5.677464	336.5769	3.326134	206.1674	5.430053	1264.353	0.885433
4.863503	199.7987	5.694482	344.1267	3.306195	227.0283	5.01149	1342.334	0.847591
4.271555	207.1897	5.047308	360.2562	2.902796			1454.666	0.718894

For peer review only

4.111383	211.4997	4.860527	376.3803	2.73128			1535.995	0.669273
2.598351	309.9834	2.586429	479.4849	1.672107	229.0925	3.499678	796.238	1.006923
2.650772	332.9899	2.363435	490.4775	1.604559	223.7218	3.517762	874.4203	0.900025
2.613604	342.6002	2.321948	503.8779	1.578756			940.9667	0.845407
2.65246	352.4267	2.34304	514.1503	1.606048			957.8435	0.862093
2.842299	202.5509	3.444813	399.9793	1.744465	193.7426	3.601428	812.8604	0.858388
2.751129	208.4241	3.109046	387.3601	1.672862	208.7181	3.104667	836.3866	0.774761
2.567797	207.2909	2.947548	317.4489	1.92472			838.0735	0.729053
2.439108	222.2212	2.903639	276.7302	2.331693			873.5824	0.738625
5.263778	144.9656	5.27194	384.3557	1.988393	200.3726	3.814144	953.8425	0.801233
4.906105	143.5828	5.129445	399.3759	1.844127	199.9491	3.683438	1020.688	0.721572
4.271778	150.9861	4.276884	416.0787	1.55199			1091.209	0.591775
4.11809	163.9292	3.841598	447.385	1.407624			1154.538	0.545456
3.714333	224.1901	3.764663	439.053	1.922319	345.8388	2.440444	808.2204	1.04427
3.450777	241.6327	3.400823	455.8974	1.802489	340.7959	2.411267	892.2401	0.920996
3.178198	266.139	2.957102	451.9551	1.741324			982.943	0.800657
3.151148	274.0349	2.922074	467.8649	1.711498			1109.744	0.721563
4.653071	171.9085	5.412763	331.5559	2.806465	154.0532	6.04012	1026.319	0.906638
4.128504	163.2719	5.256569	326.4157	2.629316	160.1018	5.360652	1044.869	0.821395
4.014963	172.6455	4.943656	332.084	2.570133			1018.623	0.837896
3.806402	176.34	4.708233	337.1556	2.462513			977.9605	0.848961
3.575661	164.8517	4.181031	275.1782	2.504741	101.6426	6.781117	801.7592	0.859672
3.283678	166.3512	3.953985	274.0533	2.400081	100.4281	6.54946	849.1478	0.7746
3.019371	176.3726	3.53513	290.8745	2.143536			875.2264	0.712387
2.698247	188.9097	3.047753	305.0722	1.887258			886.8221	0.649228
4.43238	189.6271	4.968964	301.6956	3.123181	136.4195	6.907006	1005.011	0.937552
4.450255	189.7797	5.025564	323.4283	2.948877	130.6758	7.298596	1013.214	0.941311
4.361355	197.4349	4.821842	353.6263	2.692108			1185.975	0.802715
4.146903	211.8178	4.509772	387.9957	2.462012			1302.002	0.733678
3.867482	150.5123	4.552785	329.6729	2.078576	188.665	3.6321	890.9393	0.769132
3.88186	148.2677	4.645314	333.5774	2.064738	190.772	3.610331	887.4417	0.776107
3.521851	160.3339	4.074309	329.887	1.980224			897.9174	0.727517
3.472531	174.7639	3.866646	336.0615	2.010793			954.3185	0.708097
2.788517	535.5477	1.929333	775.2405	1.332812	266.1388	3.882373	813.3729	1.270327
2.532308	544.7237	1.829368	850.7882	1.171267	305.3624	3.263336	893.5981	1.115155
2.40192	570.0156	1.860037	885.3723	1.197519			910.0862	1.165
2.23913	591.5275	1.814793	935.3385	1.147713			948.3575	1.131957
4.994019	186.5991	5.159457	338.7684	2.841912	254.2487	3.786647	818.0322	1.17691
4.991458	193.6941	4.952397	357.9793	2.679624	259.5942	3.695191	859.099	1.116577
4.829507	205.1859	4.599487	335.0626	2.816638			854.569	1.104358
4.563111	210.1812	4.474711	322.1257	2.919668			876.5529	1.072953

For peer review only

nurse_support_train

For peer review only

STROBE Statement—Checklist of items that should be included in reports of ***cross-sectional studies***

	Item No	Recommendation	Page No
**Title and abstract**	1	(*a*) Indicate the study's design with a commonly used term in the title or the abstract	1
		(*b*) Provide in the abstract an informative and balanced summary of what was done and what was found	2
**Introduction**			
Background/rationale	2	Explain the scientific background and rationale for the investigation being reported	3
Objectives	3	State specific objectives, including any prespecified hypotheses	3
**Methods**			
Study design	4	Present key elements of study design early in the paper	3
Setting	5	Describe the setting, locations, and relevant dates, including periods of recruitment, exposure, follow-up, and data collection	3
Participants	6	(*a*) Give the eligibility criteria, and the sources and methods of selection of participants	3
Variables	7	Clearly define all outcomes, exposures, predictors, potential confounders, and effect modifiers. Give diagnostic criteria, if applicable	4-5
Data sources/ measurement	8*	For each variable of interest, give sources of data and details of methods of assessment (measurement). Describe comparability of assessment methods if there is more than one group	4-5
Bias	9	Describe any efforts to address potential sources of bias	3-5
Study size	10	Explain how the study size was arrived at	3
Quantitative variables	11	Explain how quantitative variables were handled in the analyses. If applicable, describe which groupings were chosen and why	3-5
Statistical methods	12	(*a*) Describe all statistical methods, including those used to control for confounding	5-6
		(*b*) Describe any methods used to examine subgroups and interactions	Not applicable
		(*c*) Explain how missing data were addressed	6
		(*d*) If applicable, describe analytical methods taking account of sampling strategy	Not applicable
		(*e*) Describe any sensitivity analyses	6, 8
**Results**			
Participants	13*	(a) Report numbers of individuals at each stage of study—eg numbers potentially eligible, examined for eligibility, confirmed eligible, included in the study, completing follow-up, and analysed	6
		(b) Give reasons for non-participation at each stage	Not applicable
		(c) Consider use of a flow diagram	Not applicable
Descriptive data	14*	(a) Give characteristics of study participants (eg demographic, clinical, social) and information on exposures and potential confounders	6

For peer review only

		(b) Indicate number of participants with missing data for each variable of interest	6
Outcome data	15*	Report numbers of outcome events or summary measures	6
Main results	16	(*a*) Give unadjusted estimates and, if applicable, confounder-adjusted estimates and their precision (eg, 95% confidence interval). Make clear which confounders were adjusted for and why they were included	7-8
		(*b*) Report category boundaries when continuous variables were categorized	7-8
		(*c*) If relevant, consider translating estimates of relative risk into absolute risk for a meaningful time period	Not applicable
Other analyses	17	Report other analyses done—eg analyses of subgroups and interactions, and sensitivity analyses	8
**Discussion**			
Key results	18	Summarise key results with reference to study objectives	8
Limitations	19	Discuss limitations of the study, taking into account sources of potential bias or imprecision. Discuss both direction and magnitude of any potential bias	9-10
Interpretation	20	Give a cautious overall interpretation of results considering objectives, limitations, multiplicity of analyses, results from similar studies, and other relevant evidence	8-10
Generalisability	21	Discuss the generalisability (external validity) of the study results	8-10
**Other information**			
Funding	22	Give the source of funding and the role of the funders for the present study and, if applicable, for the original study on which the present article is based	11

*Give information separately for exposed and unexposed groups.

Note: An Explanation and Elaboration article discusses each checklist item and gives methodological background and published examples of transparent reporting. The STROBE checklist is best used in conjunction with this article (freely available on the Web sites of PLoS Medicine at http://www.plosmedicine.org/, Annals of Internal Medicine at http://www.annals.org/, and Epidemiology at http://www.epidem.com/). Information on the STROBE Initiative is available at www.strobe-statement.org.

For peer review only

BMJ Open

Staffing levels and hospital mortality in England: a national panel study using routinely collected data

Journal:	*BMJ Open*
Manuscript ID	bmjopen-2022-066702.R3
Article Type:	Original research
Date Submitted by the Author:	13-Apr-2023
Complete List of Authors:	Rubbo, Bruna; University of Southampton, School of Health Sciences<br>Saville, Christina; University of Southampton, School of Health Sciences; National Institute for Health Research Applied Research Collaboration (Wessex), University of Southampton<br>Dall'Ora, Chiara; University of Southampton, School of Health Sciences; National Institute for Health Research Applied Research Collaboration (Wessex), University of Southampton<br>Turner, Lesley; University of Southampton, School of Health Sciences<br>Jones, Jeremy; University of Southampton, School of Health Sciences<br>Ball, Jane; University of Southampton, School of Health Sciences<br>Culliford, David; University of Southampton, Faculty of Medicine; National Institute for Health Research Applied Research Collaboration (Wessex), University of Southampton<br>Griffiths, Peter; University of Southampton, School of Health Sciences; National Institute for Health Research Applied Research Collaboration (Wessex), University of Southampton
**Primary Subject Heading**:	Health policy
Secondary Subject Heading:	Health services research
Keywords:	Organisation of health services < HEALTH SERVICES ADMINISTRATION & MANAGEMENT, Quality in health care < HEALTH SERVICES ADMINISTRATION & MANAGEMENT, Health policy < HEALTH SERVICES ADMINISTRATION & MANAGEMENT

SCHOLARONE™
Manuscripts

I, the Submitting Author has the right to grant and does grant on behalf of all authors of the Work (as defined in the below author licence), an exclusive licence and/or a non-exclusive licence for contributions from authors who are: i) UK Crown employees; ii) where BMJ has agreed a CC-BY licence shall apply, and/or iii) in accordance with the terms applicable for US Federal Government officers or employees acting as part of their official duties; on a worldwide, perpetual, irrevocable, royalty-free basis to BMJ Publishing Group Ltd ("BMJ") its licensees and where the relevant Journal is co-owned by BMJ to the co-owners of the Journal, to publish the Work in this journal and any other BMJ products and to exploit all rights, as set out in our licence.

The Submitting Author accepts and understands that any supply made under these terms is made by BMJ to the Submitting Author unless you are acting as an employee on behalf of your employer or a postgraduate student of an affiliated institution which is paying any applicable article publishing charge ("APC") for Open Access articles. Where the Submitting Author wishes to make the Work available on an Open Access basis (and intends to pay the relevant APC), the terms of reuse of such Open Access shall be governed by a Creative Commons licence – details of these licences and which Creative Commons licence will apply to this Work are set out in our licence referred to above.

Other than as permitted in any relevant BMJ Author's Self Archiving Policies, I confirm this Work has not been accepted for publication elsewhere, is not being considered for publication elsewhere and does not duplicate material already published. I confirm all authors consent to publication of this Work and authorise the granting of this licence.

For peer review only

Staffing levels and hospital mortality in England: a national panel study using routinely collected data

Bruna Rubbo[1], Christina Saville[1,2], Chiara Dall'Ora[1,2], Lesley Turner[1], Jeremy Jones[1], Jane Ball[1], David Culliford[2], Peter Griffiths[1,2]*

[1]University of Southampton, School of Health Sciences, Southampton, UK

[2]National Institute for Health Research Applied Research Collaboration, Wessex

*Corresponding author

peter.griffiths@soton.ac.uk (PG)

Short title: Staffing levels and hospital mortality

For peer review only - http://bmjopen.bmj.com/site/about/guidelines.xhtml

Abstract

Objectives

Examine the association between multiple clinical staff levels and case-mix adjusted patient mortality in English hospitals. Most studies investigating the association between hospital staffing levels and mortality have focused on single professional groups, in particular nursing. However, single staff group studies might overestimate effects or neglect important contributions to patient safety from other staff groups.

Design

Retrospective observational study of routinely available data.

Setting and participants

138 National Health Service hospital trusts that provided general acute adult services in England between 2015 and 2019.

Outcome measure

Standardised mortality rates were derived from the Summary Hospital level Mortality Indicator dataset, with observed deaths as outcome in our models and expected deaths as offset. Staffing levels were calculated as the ratio of occupied beds per staff group. We developed negative binomial random-effects models with trust as random effects.

Results

Hospitals with higher levels of medical and allied healthcare professional (AHP) staff (i.e. podiatry and chiropody, dietetics, occupational therapy, orthoptics and optics, physiotherapy, radiography, art, music and drama therapy, and speech and language therapy) had significantly lower mortality rates (rate ratio: 1.04, 95%CI 1.02 to 1.06, and 1.04, 95%CI 1.02 to 1.06, respectively), while those with higher support staff had higher mortality rates (0.85, 95%CI 0.79 to 0.91 for nurse support, and 1.00, 95%CI 0.99 to 1.00 for AHP support), after adjusting for multiple staff groups and hospital characteristics. Estimates of staffing levels on mortality were stronger between- than within-hospitals, which were not statistically significant in a within-between random effects model.

Conclusions

Considering multiple staff groups simultaneously when examining the association between hospital mortality and clinical staffing levels is crucial.

Article Summary

Strengths and limitations of the study

- We collected data from 138 hospitals over 4 years
- We included numerous clinical staffing groups simultaneously
- We did not have access to data at the ward level, so our associations were at the hospital level only.
- We used occupied beds-per-full time/contract staff, but this might not be a complete measure of staffing levels, as it does not take into account temporary staff or staff absence.

Keywords

Scheduling and staffing; Hospital Mortality; Workforce; Patient Safety; Delivery of Health Care

For peer review only - http://bmjopen.bmj.com/site/about/guidelines.xhtml

Introduction

Cost constraints and a quest for safer hospitals put pressure on health systems to find efficient staffing models to meet rising demand for care. Adequate nurse staffing levels in acute hospitals have important implications for positive outcomes for patients, including reducing patients' risk of death and costs.(1–5) Low registered nurse staffing levels are associated with failure to respond to patient abnormal changes in their physiological vital signs, which is in turn associated with failure to prevent death.(6) This link between registered nurses, failure to detect early deterioration and risk of death has meant the focus of much of the evidence and associated policy is on nurses. Nonetheless, the healthcare workforce is made up of many different professional groups, who must be mobilised to properly respond to or prevent deterioration and staffing levels of other staff groups are also likely to influence the quality and safety of care.

Studies focussing on the impact of clinical staff other than nurses on hospital mortality rates are relatively scarce. There are several studies on the effect of physician staffing levels, and they all indicate increased risk of patient death with lower medical staffing levels.(7–18) In-hospital patient care is delivered by multidisciplinary staff and no single staff group is solely responsible for patient outcomes. However, only a few studies considered multiple staff groups simultaneously, with some finding partial attenuation or complete absence of the effect of single staff levels on mortality when adjusted for other professionally qualified staff.(7,13,14,19) To our knowledge, no recent study has examined the potential effect of allied healthcare professionals (AHP) staffing levels on clinical outcomes, which include staff in the fields of podiatry and chiropody, dietetics, occupational therapy, orthoptics and optics, physiotherapy, radiography, art, music and drama therapy, and speech and language therapy. Only a few studies focused on in-hospital pharmacists.(19,20)

In the face of enduring staffing shortages in some professional groups it is important to understand the likely consequences on patient care and to identify priorities. A focus on single professional groups risks unintended consequences through neglect of other important contributions to patient safety and might lead to biased estimates. We therefore aimed to examine the associations between staffing levels of multiple staff groups, and case-mix adjusted patient mortality in hospitals.

Methods

Study design

This was a retrospective observational study using routinely available data on clinical healthcare staffing and hospital mortality.

Study setting

We included all 138 National Health Service (NHS) hospital trusts providing general acute adult inpatient services in England between April 2015 and March 2019. Hospital trusts are defined as organisational units within the NHS that service a defined geographical area or that provide a specialised function. One trust can therefore encompass several hospitals.

For peer review only - http://bmjopen.bmj.com/site/about/guidelines.xhtml

Data sources and linkage

We linked four data sources that provide trust-level datasets: a) NHS workforce, which contains detailed information on trust staffing; b) bed availability and occupancy, which contains data on trust-level available and occupied beds; c) Estates Returns Information Collection (ERIC), which contains data on trust organisation and structure; and d) Summary Hospital-level Mortality Indicator (SHMI), which contains data on observed and expected deaths (Table S1). These datasets are openly available and accessible on the NHS Digital platform, along with the data dictionary.(21,22) We linked the datasets using the unique hospital trust ID (*i.e.* "Org code").

Standardised mortality rates derived from the SHMI includes all deaths that occur in hospital or within 30-days of discharge among patients admitted to non-specialist acute trusts in the period of a year. SHMI data are derived from the Hospital Episode Statistics (HES) at the level of provider spells (*i.e.* total continuous stay of a patient using a hospital bed at an NHS organisation under the care of one or more consultants, or nursing episode or midwife episode), and the HES-Office of National Statistics (ONS) linked mortality data (Table S1).(23) The latter captures deaths that occur outside of hospital. Data used to calculate the SHMI are submitted by each hospital trust.

SHMI expected deaths is calculated based on individual patient characteristics that can affect the risk of mortality, including the patient's condition for hospitalisation, underlying conditions (Charlson Comorbidity Index),(24) age, gender, method of admission to hospital, and year of discharge. Logistic regression models estimate the risk for each provider spell, with binary variable as outcome (*i.e.* died or survived). The SHMI model performs well with early validation accounting for 81% of between-hospital variations.(25) The models are constructed using the preceding three years' data, with last year data used to calculate the standardised mortality rates (26). As SHMI data are published monthly, we used the dataset that contains mortality data from April to March the following year to report on annual hospital mortality level for each hospital trust included in this study. Additional information, including dataset and reports, can be found at the NHS digital website (https://digital.nhs.uk/data-and-information/publications/ci-hub/summary-hospital-level-mortality-indicator-shmi).

We obtained hospital staffing data by linking the medical and dental workforce dataset (physicians) with the non-medical workforce dataset (all other healthcare workers, including nurses, nurse assistants, physiotherapists, pharmacists, among others). Bed occupancy data are published quarterly as averages, with no estimate of monthly variation. For wards open overnight, occupied bed is defined as when it is occupied at midnight on the day in question; for day-only wards, occupied bed is a bed where at least one day case has taken place during the day. Trust teaching status was derived from the ERIC dataset, which contains variables on trust profile.

Staffing variables are published monthly. We used full-time equivalent (FTE) values to calculate the annual average available staff at each hospital trust from April to March the following year, to align with the SHMI data. FTE data are standardised measures of the workload that allow for the total workforce workload to be expressed in an equivalent number of full-time staff and are based on the proportion of time each staff are expected to work in a week, which would correspond to an FTE of 1 (*e.g.* 48 hours for doctors, 37.5h for nurses and AHP). Overtime and out-of-hour work are not recorded in these datasets.

For peer review only - http://bmjopen.bmj.com/site/about/guidelines.xhtml

Study outcome

The main study outcome was annual standardised all-cause mortality rate derived from observed and expected deaths in the SHMI dataset.(27) This is calculated and reported for all trusts providing acute adult services in NHS England.(25) Specialist trusts that do not provide general acute care do not report SHMI and, therefore, were not included in the study.

Staffing variables

Clinical staff are classified according to occupation codes. We grouped staffing variables by frequency and occupation as listed by NHS England, merging groups that had mean below 3 with others of a similar occupation in order to create 8 groups (*i.e.* medical, surgical, other medical specialties, nurses, support to nurses, AHP, support to AHP, and scientific, therapeutic, and technical (ST&T) staff). General medicine specialist doctors were grouped with clinical oncology doctors to form the general medicine group, surgeons were grouped with obstetricians and gynaecologists to form the surgical group, and all other medical specialties were grouped together to form the other medical group (*i.e.* radiologists, emergency medicine, anaesthetists, and pathologists). We excluded psychiatrists and paediatricians. The nurses' group was composed of nurses in adult services only; we did not include nurses from paediatric services. The nurse support group included support to adult and general nurses, and nurses in training. Scientific, therapeutic, and technical (ST&T) staff group was composed of multi-therapists, applied psychologist, psychological therapist, pharmacists, dentists, operating theatre staff, social services, and other ST&T staff.

We used the number of general acute occupied beds to calculate the bed-per-staff ratio. Beds assigned to maternity, mental illness, and learning disabilities services were excluded; however, general acute beds represented 96.2% of the total beds available across all hospital trusts included in the study. We calculated the average number of occupied beds overnight or day-only per trust per year, and then divided occupied beds per each staff group to obtain the average bed-per-staff levels for each hospital trust per year.

Hospital-level covariates

Teaching affiliation was coded as yes or no according to the trust type recorded in the ERIC dataset. Trust size was calculated based on the number of available general acute beds in each trust.

Statistical analysis

We initially explored the data using descriptive statistics. Continuous variables were described as mean and standard deviation (SD) or median and IQR, depending on the distribution of each variable. Categorical variables were described as frequencies and proportions. Annual mortality rates were calculated by dividing the number of observed deaths by the number of patient spells, across all hospital trusts each year. To explore the relationship between staffing levels and hospital mortality, statistical modelling was conducted at the hospital trust-level using multiple regression models on four years of data, with standardised mortality rates regressed onto hospital level staffing levels (expressed as the number of occupied beds-per-staff). We included expected deaths as an offset, as the number of expected deaths is the number of times the event could have happened (*i.e.* the exposure variable).

Multilevel models were adjusted for trust size and affiliation as a teaching hospital, with trust included as a random effect to adjust for clustering. We considered a range of potential

For peer review only - http://bmjopen.bmj.com/site/about/guidelines.xhtml

models, with model selection based on theoretical reasoning and model fit (minimising the Akaike information criterion (AIC), the Bayesian information criterion (BIC) and the likelihood ratios (see Table S2 for alternative frameworks considered)). We report exponentiated coefficients (rate ratios) with 95% confidence intervals (CI) for all estimates obtained from the models.

The best performing model (referred to as main model) was a negative binomial random effects model that included all 8 clinical staff groups and hospital characteristics (*i.e.* trust size and teaching status). To explore variations between and within hospital trusts, we also constructed a within-between random effects model (WBRE), with trusts as random effect, and staffing and hospital characteristics as fixed effects. These models can differentiate between effects arising from staffing differences between hospital trusts and those that are associated with annual changes of staffing levels within each hospital by group-mean centring each trust.(28,29)

We assessed potential collinearity between co-variates using correlation plots and Spearman's correlation coefficient, and the overall multicollinearity for all predictors in the models using generalised variance inflation factors (GVIF). All models had a GVIF<10. As a sensitivity analysis, we reran the models after excluding any variables that had a GVIF above 5.(30)

Observations with missing data were removed from the analyses; however, hospital trusts that reported at least one year of complete data were included.

We performed data linkage, cleaning, coding, and analyses in R statistical software, version 4.0.2 (R Core Team, R Foundation for Statistical Computing, Vienna, Austria), using the lme4 and glmer packages (v1.1-27.1, Bates et al, 2015), and the plm package (v2.4-3, Croissant et al, 2021).(31) The full dataset is available.(32)

Patient and public involvement

There was no patient and public involvement in this study.

Results

Descriptive statistics

There were 540 observations in the linked dataset for the 4-year study period, of which 519 (96%) contained data on all variables and were therefore included in our analyses. There were 2 hospital trusts that did not report on medical staffing in 2016, with 6 trusts not reporting these variables from 2017 onwards. For other clinical staff, only 1 trust did not report staff numbers in 2016 and 2017, and two trusts in 2018, with no missing data in 2019. One trust did not report the number of occupied general acute beds in 2018. The number of hospital trusts that reported on complete data varied, with 137 included in 2016 and 2017, 135 in 2018, and 130 in 2019.

The median number of acute general occupied beds per day was 674.0 (IQR 486.1 to 911.6). The mean annual observed deaths ± SD was 2229 ± 992 (range 525 to 7468), while the expected deaths were 2228 ± 987.2 (range 665 to 7591). Annual mortality rates across all hospital trusts were 3.44% for 2016, 3.46% for 2017, 3.23% for 2018, and 3.31% for 2019. There was a median of 744.2 available general acute beds per trust (interquartile range (IQR) 535.7 to 1009.4, range 143 to 2704). Hospital trusts in the upper tertile (*i.e.* the upper third of trusts, ranked by size) were classified as large, while those in the lower tertile were deemed small trusts, with the remaining classified as medium-sized hospital trusts. Forty-one (29.7%) hospital trusts were classified as teaching trusts.

For peer review only

Table 1 shows the distribution of staffing variables by their median, IQR, mean, and SD across all hospital trusts. There was considerable variation in staffing levels, with nurse staff levels showing least relative variation (SD 18.0% of the mean) and AHP support most (SD 44.4% of the mean). By contrast, variations within trusts as a percentage of the mean were relatively low, with the within trust variation ranging from 4.9% for nurse staffing to 10.9% for AHP support.

Table 1. Frequency of occupied bed-per-staff variables across 138 hospital trusts

Staffing level variables	Median (IQR)	Mean (SD)	SD as % of mean	Mean within-trust SD	Within-trust SD as % of mean
Bed per medical	3.66 (3.05 to 4.30)	3.70 (0.96)	25.9%	0.28	7.6%
Bed per surgical	3.79 (3.10 to 4.28)	3.73 (0.81)	21.7%	0.22	5.9%
Bed per other medical specialties	3.96 (3.26 to 4.69)	3.94 (1.01)	25.6%	0.27	6.9%
Bed per nurse adult service	0.61 (0.54 to 0.68)	0.61 (0.11)	18.0%	0.03	4.9%
Bed per nurse support	0.93 (0.80 to 1.08)	0.93 (0.21)	22.6%	0.07	7.5%
Bed per AHP	2.25 (1.88 to 2.74)	2.36 (0.89)	37.7%	0.18	7.6%
Bed per AHP support	10.03 (7.91 to 13.01)	11.07 (4.91)	44.4%	1.21	10.9%
Bed per ST&T	2.24 (1.80 to 2.82)	2.31 (0.75)	32.5%	0.16	6.9%

Q1: lower quartile, Q3: upper quartile, SD: standard deviation, AHP: allied healthcare professional, ST&T: scientific, therapeutic, and technical

All groups of medical staff were highly correlated with each other (range rho=0.68 to 0.85) (Figure S1). Nurse staffing levels were strongly correlated with staffing levels in all medical staff groups (rho>0.71). Nurse support staff levels were correlated to RN (rho=0.30), as were AHP and AHP support (rho=0.47), but neither support staff were correlated to medical staff (0.07 and -0.02, respectively). As the correlations between registered staff and their support were weak to moderate, there does not seem to be a substitution effect between these groups.

Staffing levels and hospital mortality

Table 2 shows results for the negative binomial random effect models, adjusted for hospital characteristics. In the single staff group models, hospital trusts that had lower staffing levels (*i.e.* more occupied beds per FTE staff) were associated with higher (standardised) mortality rates for all professionally qualified staff groups. The opposite effect was observed for

For peer review only - http://bmjopen.bmj.com/site/about/guidelines.xhtml

nursing support and AHP support staff, where hospital trusts with lower staffing levels (*i.e.* more occupied beds per FTE staff) had lower mortality.

Table 2. Exponentiated estimates (rate ratios) for hospital mortality from the multilevel negative binomial random effect models, adjusted for hospital characteristics (*n* = 138)

Predictors	Estimates for single staff models	95% CI	Estimates for multiple staff model	95% CI
Bed per medical	1.05***	1.04 to 1.07	1.04***	1.02 to 1.06
Bed per surgical	1.04***	1.02 to 1.06	0.98	0.96 to 1.01
Bed per other medical specialties	1.05***	1.03 to 1.06	1.03*	1.00 to 1.06
Bed per nurse adult service	1.33***	1.15 to 1.54	1.07	0.88 to 1.31
Bed per nurse support	0.92*	0.86 to 0.98	0.85***	0.79 to 0.91
Bed per AHP	1.02*	1.00 to 1.04	1.04***	1.02 to 1.06
Bed per AHP support	0.99*	0.99 to 0.99	1.00**	0.99 to 1.00
Bed per ST&T	1.02**	1.00 to 1.04	0.99	0.98 to 1.05
Teaching (ref not teaching)	0.96*#	0.92 to 0.99	1.01	0.98 to 1.05
Medium trust (ref small trust)	1.01#	0.97 to 1.05	1.01	0.98 to 1.04
Large trust (ref small trust)	1.00#	0.96 to 1.04	1.01	0.98 to 1.05

CI: confidence interval, AHP: allied healthcare professional, ST&T: scientific, therapeutic, and technical, # estimates for univariate models, without any staffing variables, * p<0.05, ** p<0.01, *** p<0.001. Estimates are a ratio of observed to expected deaths, all models that included one or multiple staffing levels have been adjusted for hospital characteristics (i.e. teaching status and trust size).

In the main model including all staff groups, associations between medical (rate ratio: 1.04, 95%CI 1.02 to 1.06), other medical specialties (1.03, 95%CI 1.00 to 1.06), AHP (1.04, 95%CI 1.02 to 1.06), nurse support (0.85, 95%CI 0.79 to 0.91), and AHP support (1.00, 95%CI 0.99 to 1.00) FTE staffing levels and hospital mortality remained statistically significant, with no change in direction of the effect compared to the single staff models (Table 2). Higher mortality rates were observed in hospital trusts with lower levels of medical and AHP staff. In contrast, hospital trusts with lower support staff levels per occupied bed (*i.e.* nursing support and AHP support) had lower mortality rates. The association with RN staffing was attenuated and no longer statistically significant (1.07, 95%CI 0.88 to 1.31), although the observed effect remained relatively large compared to other associations (Table S3). Similarly, the association between hospital mortality and surgical and ST&T staff, observed in the single staff groups models, were no longer statistically significant in the model adjusted for all staff groups.

Although we noted correlation between staffing variables, our GVIF for all variables was <10. The GVIF for beds per other medical specialties was 6.1 (Table S3), and therefore over our threshold of 5, so we ran the negative binomial random effects model omitting this variable as a sensitivity analysis and found similar results (not shown; available from authors upon request).

Within-between hospital trust variability

Between-hospital trust effects from the WBRE model were largely the same as those obtained from the main model (*i.e.* between only). For example, the between- rate ratio estimate from WBRE for the FTE medical group was 1.04 (95%CI 1.02 to 1.07), similar to the estimates obtained from the main model (1.04, 95%CI 1.02 to 1.06). However, even for staff groups where between-hospital trusts effects were significant, the within-hospital effects were small and not statistically significant. For example, the within-hospital estimate from the WBRE model for the medical group was 0.99 (95%CI 0.97 to 1.01) (Table 3). Despite potentially providing additional information by decomposing the variability into between- and

For peer review only - http://bmjopen.bmj.com/site/about/guidelines.xhtml

288 within-estimates, the WBRE model did not improve fit compared to the main model (Table
289 S2).

290 Table 3. Exponentiated estimates (rate ratios) for hospital mortality from the multilevel within-
291 between negative binomial random effect models, adjusted for hospital characteristics (*n* =
292 138).

Predictors	Observed deaths<br>Incidence Rate Ratios	95% confidence intervals	p-values
**Bed per medical**			
Within-hospital	0.98	0.97 – 1.00	0.129
Between-hospital	1.04	1.02 – 1.07	**<0.001**
**Bed per surgical**			
Within-hospital	1.03	0.99 – 1.06	0.106
Between-hospital	0.98	0.95 – 1.01	0.222
**Bed per other medical specialties**			
Within-hospital	1.02	0.99 – 1.04	0.21
Between-hospital	1.02	0.99 – 1.06	0.124
**Bed per nurse**			
Within-hospital	0.88	0.75 – 1.04	0.148
Between-hospital	1.1	0.88 – 1.37	0.393
**Bed per nurse support**			
Within-hospital	0.99	0.91 – 1.07	0.789
Between-hospital	0.84	0.77 – 0.90	**<0.001**
**Bed per AHP**			
Within-hospital	0.99	0.96 – 1.01	0.296
Between-hospital	1.04	1.02 – 1.07	**<0.001**
**Bed per AHP support**			
Within-hospital	1	1.00 – 1.00	0.352
Between-hospital	1	0.99 – 1.00	**0.017**
**Bed per ST&T**			
Within-hospital	1.01	0.99 – 1.04	0.329
Between-hospital	0.99	0.96 – 1.01	0.266
**Teaching hospital**			
Yes	1.02	0.98 – 1.05	0.323
**Trust size (reference: small)**			
Medium	1	0.98 – 1.03	0.706
Large	1.01	0.98 – 1.04	0.381

293
Discussion

294 After adjustment for hospital characteristics and including multiple clinical staff groups in our
295 models, we found that higher medical and AHP staffing levels were associated with lower
296 mortality rates in acute hospital trusts in England during the period of 2015 to 2019. In
297 contrast, hospitals with higher support staff per occupied bed had higher mortality rates.

298 Our study highlights the importance of simultaneously adjusting for multiple clinical staff
299 groups when investigating associations between staffing levels and mortality at the hospital-
300 level. In the single staff group models, significant effects on mortality rates were seen for all

For peer review only - http://bmjopen.bmj.com/site/about/guidelines.xhtml

staffing groups. However, when adjusted for multiple groups, effects for medical, other medical specialties, AHP, nurse support, and AHP support staffing levels remained statistically significant, but levels of surgical doctors, RN, and ST&T staff were no longer significant.

Our findings are in line with a previous study by Jarman *et al*,(7) who found that the association between RN staffing and mortality was no longer significant when adjusting for doctors-per-bed, with higher levels of physician staffing significantly reducing hospital standardised mortality ratios.

Nonetheless, other studies have shown a reduction in hospital mortality when both higher numbers of doctors and nurses per bed were observed.(11,13–15,33–36) The fact that the association between nurse staffing and mortality was not significant in the multivariable models should not be taken to indicate an absence of effect to nursing. Previous studies have reported that much of the variation in nurse staffing occurs between wards within hospitals and within wards over time (1,37) and Keogh noted that hospital nurse staffing levels often bore little relationship to staffing available to be deployed on wards in NHS hospitals,(38) therefore hospital nurse staffing per bed may be a poor indicator of the staffing levels experienced by inpatients. Griffiths *et al* (13) found that when medical staffing levels were accounted for, significant nurse staffing effects were only observed in models using ward-based staffing ratios as opposed to hospital-level staff-per-bed ratios. Longitudinal patient level studies of exposure to variation in nurse staffing confirm that there is an adverse effect of low nurse staffing when measured at this level.(2,39,40)

To our knowledge, this was the first study to include AHP and AHP support staff levels in analysis. Higher levels of AHP staff had a protective effect at the hospital trust level while more AHP support staff were detrimental to patient mortality, a finding that mirrors those obtained for nursing support staff. While causality cannot be assumed for any of these results, there is a potential that previous findings that have emphasised a link between nurse staffing and patient safety could divert attention from the role that other staff groups may play in delivering quality and preventing avoidable deaths.

In our study, high levels of support staff were associated with higher mortality rates. This finding appears to be paradoxical: increasing the number of some staff groups might lead to increased mortality, when holding all other staffing variables constant. This cannot be fully explored with the given data. One potential reason might be that raising levels of one staff group might affect the distribution of other staff groups to different departments and wards and this could negatively affect mortality. Another possible reason could be that increasing the levels of one staff group might alter the duties of other staff groups – for example, adding more nurse assistants while holding the number of registered nurses constant might increase the workload for the registered nurses as they are now responsible for the supervision of a larger number of nurse assistants, or create an implicit demand for delegation of work that is not suited to the assistants, which could negatively impact mortality.(39) Unfortunately, due to the fact that we do not have granular in-hospital data on deployment of staff and staff responsibility, we are unable to further investigate this, although this mechanism is consistent with evidence on skill mix in the nursing team which shows a tipping point where adding assistants has a positive effect when the number of assistant is low, but a negative one where the number is high.(39)

We found that variations between hospital trusts in the 4-year study period were higher in magnitude than within-hospital trusts estimates, which were not statistically significant for any of the clinical staff groups at the hospital level. Structural recruitment and retention difficulties, financial constraints, and other non-staffing resources available at each hospital could be main contributors to inter-hospital trust variations. There was relatively little year on year variation in staffing between trusts. Reduced variability of staffing levels within hospitals might be due to the limited number of funded posts in each organisation and could reflect

For peer review only - http://bmjopen.bmj.com/site/about/guidelines.xhtml

historic staffing levels. Bjerregaard *et al*,(36) using a WBRE model, found staffing levels estimates were significant between wards, while within-ward estimates had no significant effect on hospital mortality. Bjerregaard et al.(36) proposed that between hospital effects may reflect structural differences in staffing resource between hospitals while the limited within hospital variation we observed could be largely endogenous, reflecting change in staff in response to variation in patient populations and risk that is not reflected in the case mix adjustment model. This proposition remains untested.

Strengths and limitations

This was a national study using 4 years of routinely available data. The inclusion of numerous clinical staffing groups simultaneously in the models allowed us to estimate the effect of each staffing level on hospital mortality while considering hospital trust characteristics.

We used a variety of models and selected the one that performed best on our data. Most studies investigating clinical staffing levels and in-hospital mortality used fixed effects models, which only evaluate within-hospital effects. Such models might not appropriately assess the effect of different staffing levels based on variation between hospitals in hospital-level observational studies.

However, our study was limited by the relatively small sample size, as observations were clustered on 138 hospital trusts. We therefore had to group some of the medical specialties and were unable to unpack relatively heterogenous groups such as AHP. Furthermore, we were unable to explore the effects of grade and experience for medical (*e.g.* junior doctors, level of consultants) and RN (*e.g.* senior nurses) staff.

In our study, beds that were occupied by multiple patients in a single day were counted as one single occupied bed. Studies have shown that workload (*e.g.* number of admissions, discharges, additional tasks) in high volume hospitals can lead to higher mortality rates, even when levels of staffing are similar to those observed in low volume hospitals. Occupied beds-per-staff might not be a good measure of staffing levels, as these do not account for temporary staff or staff absence due to sickness, maternity leave, or long-term leave. The extent to which staff are deployed to deliver services such as ambulatory care or other services not delivered to inpatient beds is likely to vary.(41) FTE might not be an accurate representation of actual hours worked by the clinical staff as trusts with similar FTE staff might have a higher or lower rotation of staff responsible for a single bed during a shift, which could affect the quality of care, including mortality rates.(42) Additionally, our bed-to-staff rations might have been overestimated, as we could not include overtime and out-of-hour since these data are not captured in the datasets used for this study.

Future studies should aim to capture different staffing levels responsible for delivering patient care measured at the patient or ward-level, rather than at the hospital-level.

Although SHMI provides good control for patient risk and we controlled for hospital factors and multiple staff groups, the cross-sectional nature of our analysis means that findings need to be interpreted as demonstrating association but not direct evidence of causation.

Conclusion

In conclusion, our study highlights the importance of simultaneously considering multiple staff groups when investigating the effect of clinical staffing levels and mortality at the hospital-level. We showed that the number of AHP and AHP support per occupied beds have a significant impact on patient mortality, yet these groups have largely not been included in previous workforce studies, of which the majority focused exclusively on nursing

For peer review only - http://bmjopen.bmj.com/site/about/guidelines.xhtml

staff. We also found that hospitals with higher levels of medical staffing had lower mortality rates, while higher levels of support workers were associated with higher hospital mortality.

Data Statement

The dataset is available from the ePrints repository, DOI: https://doi.org/10.5258/SOTON/D2055

Authors' contributions

BR, CS, CDO, LT, JJ, JB, DC, and PG made substantial contributions to the conception or design of the work. BR, JJ, PG acquired the data, BR analysed the data, CS and DC independently checked the data analyses, BR, CS, CDO, LT, JJ, JB, DC, and PG discussed the interpretation of data for the work; BR drafted the work, and BR, CS, CDO, LT, JJ, JB, DC, and PG revised it critically for important intellectual content and gave final approval of the version to be published; PG is accountable for all aspects of the work in ensuring that questions related to the accuracy or integrity of any part of the work are appropriately investigated and resolved.

Conflicts of interest

All authors have completed the ICMJE uniform disclosure form at http://www.icmje.org/disclosure-of-interest/ and declare: all authors had financial support from the NIHR Health Services and Delivery Research for the submitted work; no financial relationships with any organisations that might have an interest in the submitted work in the previous three years; no other relationships or activities that could appear to have influenced the submitted work."

Funding

This study/project is funded by the NIHR [Health and Social Care Delivery Research (NIHR128056)]. The views expressed are those of the author(s) and not necessarily those of the NIHR or the Department of Health and Social Care.

Ethics Approval

We did not need ethics approval as this project relied on publicly available routinely collected data.

For peer review only

For peer review only - http://bmjopen.bmj.com/site/about/guidelines.xhtml

References

1 Griffiths P, Ball J, Bloor K, et al. Nurse staffing levels, missed vital signs and mortality in hospitals: retrospective longitudinal observational study. *Health Services and Delivery Research* 2018;6:38

2. Dall'Ora C, Saville C, Rubbo B, et al. Nurse staffing levels and patient outcomes: A systematic review of longitudinal studies. *Int J Nurs Stud*. 2022;134:104311.

3. Dietermann K, Winter V, Schneider U, et al. The impact of nurse staffing levels on nursing-sensitive patient outcomes: a multilevel regression approach. Eur J Health Econ. 2021;22(5):833–46.

4. Kane RL, Shamliyan TA, Mueller C, et al. The association of registered nurse staffing levels and patient outcomes: systematic review and meta-analysis. Med Care. 2007;45(12):1195–204.

5. Winter V, Dietermann K, Schneider U, et al Nurse staffing and patient-perceived quality of nursing care: a cross-sectional analysis of survey and administrative data in German hospitals. *BMJ Open*. 2021;11(11):e051133.

6. Hogan H, Healey F, Neale G, et al. Preventable deaths due to problems in care in English acute hospitals: a retrospective case record review study. *BMJ Qual Saf*. 2012;21(9):737–45.

7. Jarman B, Gault S, Alves B, et al. Explaining differences in English hospital death rates using routinely collected data. *BMJ*. 1999;318(7197):1515–20.

8. Pronovost PJ, Angus DC, Dorman T, et al. Physician staffing patterns and clinical outcomes in critically ill patients: a systematic review. *JAMA*. 2002;288(17):2151–62.

9. Lin HC, Lee HC. Caseload volume-outcome relation for pulmonary embolism treatment: association between physician and hospital caseload volume and 30-day mortality. *J Thromb Haemost*. 2008;6(10):1707–12.

10. Ong M, Bostrom A, Vidyarthi A, et al. House staff team workload and organization effects on patient outcomes in an academic general internal medicine inpatient service. *Arch Intern Med*. 2007;167(1):47–52.

11. Ricciardi R, Nelson J, Roberts PL, et al. Is the presence of medical trainees associated with increased mortality with weekend admission? *BMC Med Educ*. 2014;14:4.

12. Coit MH, Katz JT, McMahon GT. The effect of workload reduction on the quality of residents' discharge summaries. *J Gen Intern Med*. 2011;26(1):28–32.

13. Griffiths P, Ball J, Murrells T, et al. Registered nurse, healthcare support worker, medical staffing levels and mortality in English hospital trusts: a cross-sectional study. *BMJ Open*. 2016;6(2):e008751.

14. Bond CA, Raehl CL, Pitterle ME, et al. Health care professional staffing, hospital characteristics, and hospital mortality rates. *Pharmacotherapy.* 1999;19(2):130–8.

15. West E, Barron DN, Harrison D, et al. Nurse staffing, medical staffing and mortality in Intensive Care: An observational study. *Int J Nurs Stud.* 2014;51(5):781–94.

For peer review only - http://bmjopen.bmj.com/site/about/guidelines.xhtml

16. Dara SI, Afessa B. Intensivist-to-bed ratio: association with outcomes in the medical ICU. *Chest.* 2005;128(2):567–72.

17. Neuraz A, Guérin C, Payet C, et al. Patient Mortality Is Associated With Staff Resources and Workload in the ICU: A Multicenter Observational Study. *Crit Care Med*. 2015;43(8):1587–94.

18. Gershengorn HB, Harrison DA, Garland A, et al. Association of Intensive Care Unit Patient-to-Intensivist Ratios With Hospital Mortality. *JAMA Intern Med*. 2017;177(3):388–96.

19. Lee H, Ryu K, Sohn Y, et al. Impact on Patient Outcomes of Pharmacist Participation in Multidisciplinary Critical Care Teams: A Systematic Review and Meta-Analysis. *Crit Care Med*. 2019;47(9):1243–50.

20. Bond CA, Raehl CL, Franke T. Interrelationships among mortality rates, drug costs, total cost of care, and length of stay in United States hospitals: summary and recommendations for clinical pharmacy services and staffing. *Pharmacotherapy*. 2001;21(2):129–41.

21. [dataset] NHS Digital. National Workforce Data Set (NWD) and NHS occupational codes. [accessed 2022 Nov 02]. Available from: https://digital.nhs.uk/data-and-information/data-collections-and-data-sets/data-sets/national-workforce-data-set-nwd-and-nhs-occupation-codes#top

22. NHS Digital. NHS Data Model and Dictionary. NHS England; [accessed 2022 Nov 02]. Available from: https://datadictionary.nhs.uk/index.html

23. Aylin P, Williams S, Bottle A, Jarman B. Counting hospital activity: spells or episodes? *BMJ*. 2004;329(7476):1207.

24. Charlson ME, Pompei P, Ales KL, et al. A new method of classifying prognostic comorbidity in longitudinal studies: development and validation. *J Chronic Dis.* 1987;40(5):373–83.

25. Campbell MJ, Jacques RM, Fotheringham J, et al. Developing a summary hospital mortality index: retrospective analysis in English hospitals over five years. *BMJ.* 2012;344:e1001.

26. Campbell MJ, Jacques RM, Fotheringham J, et al. An evaluation of the Summary Hospital Mortality Index. University of Sheffield; 2011.

27. NHS Digital. The processing cycle and HES data quality NHS Digital: NHS England. [accessed 2022 Feb 11]. Available from: https://digital.nhs.uk/data-and-information/data-tools-and-services/data-services/hospital-episode-statistics/the-processing-cycle-and-hes-data-quality

28. Bell A, Fairbrother M, Jones K. Fixed and random effects models: making an informed choice. *Qual Quant.* 2019;53(2):1051–74.

29. Schunck R. Within and between Estimates in Random-Effects Models: Advantages and Drawbacks of Correlated Random Effects and Hybrid Models. *Stata J.* 2013;13(1):65–76.

For peer review only - http://bmjopen.bmj.com/site/about/guidelines.xhtml

30. O'brien RM. A Caution Regarding Rules of Thumb for Variance Inflation Factors. *Qual Quant*. 2007;41(5):673–90.

31. R Core Team (2018). R: A language and environment for statistical computing. R Foundation for Statistical Computing, Vienna, Austria. [accessed 2022 Nov 08]. Available online at https://www.R-project.org/.

32. [dataset] Rubbo B, Griffiths P, Saville C, et al. NHS England staffing levels and mortality dataset. University of Southampton; 2021. Available at DOI: 10.5258/SOTON/D2055

33. Griffiths P, Jones S, Bottle A. Is "failure to rescue" derived from administrative data in England a nurse sensitive patient safety indicator for surgical care? Observational study. *Int J Nurs Stud*. 2013;50(2):292–300.

34. Ozdemir BA, Sinha S, Karthikesalingam A, et al. Mortality of emergency general surgical patients and associations with hospital structures and processes. *Br J Anaesth*. 2016;116(1):54–62.

35. Chung W, Sohn M. The Impact of Nurse Staffing on In-Hospital Mortality of Stroke Patients in Korea. *J Cardiovasc Nurs*. 2018;33(1):47–54.

36. Bjerregaard U, Hølge-Hazelton B, Rud Kristensen S, et al. Nurse Staffing and Patient Outcomes: Analyzing Within- and Between-Variation. *DaCHE Discussion Papers.* 2020

37. Van den Heede K, Sermeus W, Diya L, et al. Nurse staffing and patient outcomes in Belgian acute hospitals: cross-sectional analysis of administrative data. *Int J Nurs Stud.* 2009;46(7):928–39.

38. Keogh B. Review into the Quality of Care and Treatment Provided by 14 Hospital Trusts in England: Overview Report. London, UK: National Health Service; 2013 [accessed 2022 Nov 2]. Available from: https://psnet.ahrq.gov/issue/review-quality-care-and-treatment-provided-14-hospital-trusts-england-overview-report

39. Griffiths P, Maruotti A, Recio Saucedo A, et al. Nurse staffing, nursing assistants and hospital mortality: retrospective longitudinal cohort study. *BMJ Qual Saf.* 2019;28(8):609–17.

40. Needleman J, Buerhaus P, Pankratz VS, et al. Nurse staffing and inpatient hospital mortality. *N Engl J Med.* 2011;364(11):1037–45.

41. Ball J. Nurse staffing, care left undone and patient mortality in acute hospitals. Doctoral thesis. Inst för lärande, informatik, management och etik / Dept of Learning, Informatics, Management and Ethics; 2017 [accessed 2022 Nov 2]. Available from: http://openarchive.ki.se/xmlui/handle/10616/45563

42. Denson JL, Jensen A, Saag HS. Associations between end-of-rotation resident transition in care and mortality among hospitalized patients. *JAMA* 2016;316(21):2204-2213.

Supporting information captions

Table S1. NHS digital data sources

Table S2. Comparison of goodness-of-fit for model selection

Table S3. Multicollinearity test in the main model (*i.e.* negative binomial random effect)

Figure S1. Spearman correlation between staffing level variables included in the study.

For peer review only - http://bmjopen.bmj.com/site/about/guidelines.xhtml

Supporting information

Tables

Table S1. NHS digital data sources

Data source	Datasets	Main variables available
NHS workforce data	Medical dataset	Professionally qualified clinical staff
	Non-medical dataset	Professionally qualified clinical staff
		Support to clinical staff
Bed occupancy data	Overnight beds dataset	Available beds
		Occupied beds
		% occupied beds
	Day only beds datasets	Available beds
		Occupied beds
		% occupied beds
ERIC data		Trust profile (e.g. type)
		Strategies and policies
		Finance
		Safety
		Fire and safety
		Business transport
		Medical records
		Key worker accommodation
		Facilities management services
		Areas
		Function and space
		Quality of buildings
		Combined heat and power energy
		Energy
		Water services
		Waste
SHMI data		SHMI value
		SHMI banding
		Number of spells*
		Observed deaths
		Expected deaths

For peer review only

NHS: National Health Service, ERIC: Estates Returns Information Collection, SHMI: Summary Hospital level Mortality Indicator, * spells: total continuous stay of a patient using a hospital bed at an NHS organisation under the care of one or more consultants, or nursing episode or midwife episode.

Table S2. Comparison of goodness-of-fit for model selection

Model	Correlations for trusts	Model degrees of freedom	AIC	BIC	Likelihood-ratio
**OLS**	No	17	8183.5	8255.8	-4074.7
**Poisson**	No	16	10567.6	10635.7	-5267.8
**Negative binomial**	No	17	6692.6	6764.9	-3329.3
**GLS**	Yes	26	7517.0	7627.6	-3732.5
**GLS exchangeable**	Yes	18	7867.5	7944.0	-3915.7
**GLS Toeplitz (2)**	Yes	19	7823.9	7904.7	-3892.9
**GLS AR**	Yes	18	7822.7	7899.2	-3893.3
**Poisson RE**	Yes	14	1997.8	2057.3	-984.9
**Negative binomial RE**	Yes	14	250.3	309.9	-111.2
**Poisson FE**	Yes (dummy)	146	6477.4	7098.2	-3092.7
**Negative binomial FE**	Yes (dummy)	147	6180.5	6805.6	-2943.3
**Negative binomial WBRE**	Yes	22	6418.3	6511.8	-3187.1

AIC: Akaike information criterion, BIC: Bayesian information criterion, OLS: ordinary least squares, GLS: generalised least squares, AR: autoregressive, RE: random effects, FE: fixed effects, WBRE: within-between random effects, (2) tridiagonal 2-Toeplitz matrix

Table S3. Multicollinearity test in the negative binomial random effects model

Variable	GVIF
Beds per medical	3.1
Beds per surgical	3.6
Beds per other medical	6.1
Beds per nurse	3.7
Beds per nurse support	1.7
Beds per AHP	2.0
Beds per AHP support	1.4

For peer review only

Beds per ST&T	2.6
Teaching status	1.8
Trust size	1.1*

GVIF: generalised variance inflation factor; AHP: allied health professionals; ST&T: scientific, therapeutic, and technical; *reported on GVIF^(1/(2*degrees of freedom)) instead of GVIF as variable has 2 degrees of freedom

Figure S1. Spearman correlation between staffing level variables included in the study.

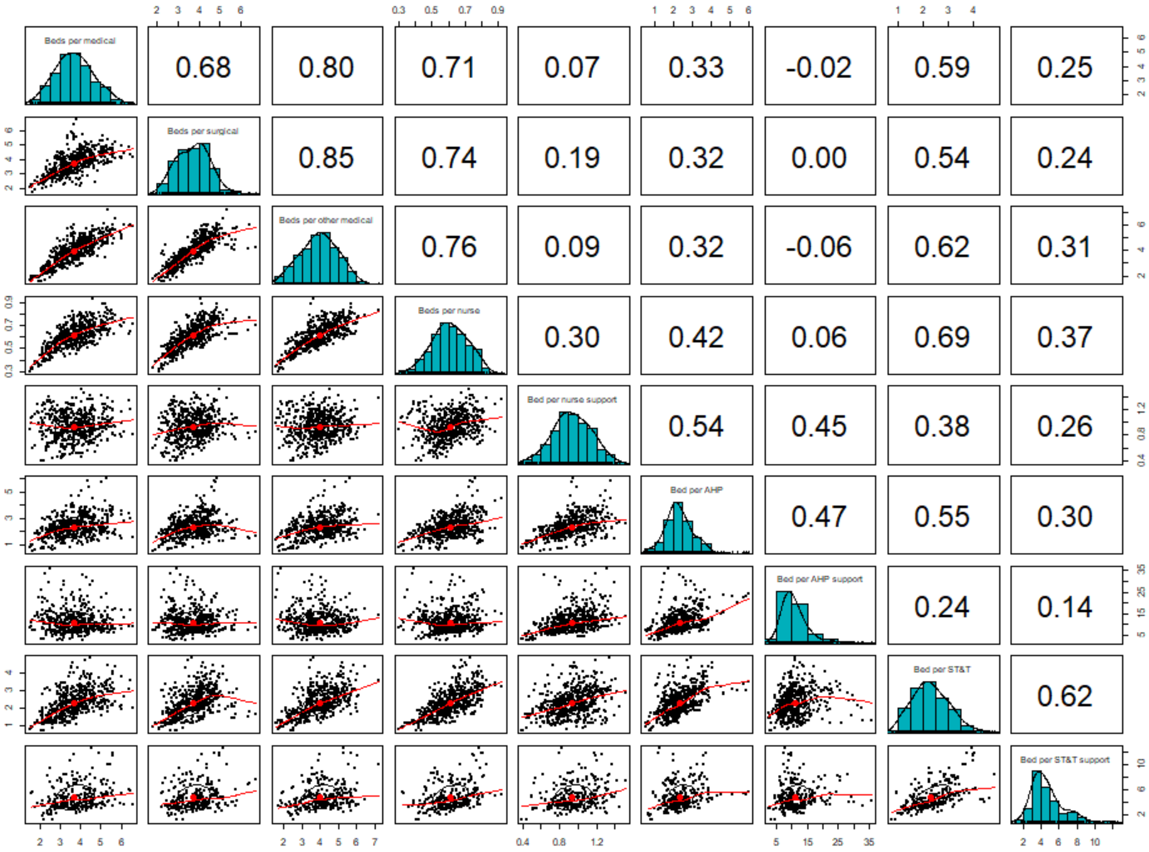

Supplement: Author's manuscript [file bmjopen-2022-066702.draft_revisions.pdf]
